# Global burden of skin cancer and its subtypes: a comprehensive analysis from 1990 to 2021 with projections to 2040

**DOI:** 10.3389/fpubh.2025.1610661

**Published:** 2025-09-04

**Authors:** Keyue Chen, Xiaoyi Liu

**Affiliations:** ^1^Research Centre of Basic Integrative Medicine, School of Basic Medical Sciences, Guangzhou University of Chinese Medicine, Guangzhou, China; ^2^State Key Laboratory of Traditional Chinese Medicine Syndrome, Guangzhou University of Chinese Medicine, Guangzhou, China; ^3^The Second Clinical Medical College, Guangzhou University of Chinese Medicine, Guangzhou, China

**Keywords:** Global Burden of Disease, skin cancer, melanoma, squamous-cell carcinoma, basal-cell carcinoma, prediction

## Abstract

**Background:**

Skin cancer represents a significant global public health concern. Comprehensive analysis of its global burden provides critical insights for evidence-based interventions. This study systematically evaluates the global disease burden of skin cancer and its subtypes.

**Methods:**

This study analyzed GBD 2021 data to assess ASIR and ASDR trends for total skin cancer and its subtypes (1990–2021), stratified by geography, age, and sex, using APC modeling, decomposition analysis, and inequality assessments, with projections through 2040.

**Results:**

First, skin cancer ASIR increased from 1990 to 2021, while ASDR significantly decreased. Second, geographical heterogeneity existed in distribution of histological subtypes. Third, skin cancer burden demonstrated age-dependent progression with compositional variance in subtypes across age groups. Fourth, sex disparities intensified beyond age 55, with increasing longitudinal divergence. Fifth, both the ASIR and ASDR of total skin cancer showed non‑linear associations with SDI. Sixth, international disparities in skin cancer burden demonstrated a decreasing trend. Finally, projections to 2040 indicate a continued increase in total skin cancer ASIR accompanied by a persistent decline in total skin cancer ASDR.

**Conclusion:**

ASIRs of total skin cancer and its subtypes showed increasing trends, while ASDRs demonstrated decreasing patterns, with significant heterogeneity across regions, age groups, and sex.

## 1 Introduction

Skin cancer comprises a spectrum of malignant neoplasms originating in the epidermis, affecting individuals across all racial, socioeconomic strata, and geographic regions (1–3). The global burden of skin cancer has demonstrated a significant increase (1, 2, 4). In England, the incidence rate of skin cancer was four-fold higher relative to other malignancies (5). According to the latest TNM classification system, skin cancer is categorized into several subtypes, including basal-cell carcinoma (BCC), squamous-cell carcinoma (SCC) (collectively designated as non-melanoma skin cancer, or NMSC, ICD-10: C44-C44.9), melanoma (ICD-10: C43-C43.9), and other rare subtypes (6). BCC constitutes the most prevalent form of skin cancer, accounting for 80% of NMSC cases (7), with an annual incidence growth rate of 3–10% (8). Recent epidemiological estimates indicate that the annual incidence of BCC in the United States approaches 2,000,000 cases (9), while in the United Kingdom, the incidence was documented at 3 cases per 1,000 individuals in 2015, with a projected annual increase of approximately 5% (5). SCC represents the second most prevalent variant of NMSC, with an incidence rate of approximately 1,035 cases in males and 472 cases in females per 100,000 person-years (10). Melanoma represents a highly aggressive form of cutaneous malignancy, characterized by a steadily increasing global incidence rate (11). From 1990 to 2017, the incidence of SCC increased by 310%, melanoma by 161%, and BCC by 77% (4), resulting in significant healthcare costs worldwide, with medical expenses expected to rise substantially in subsequent decades (12–15). Skin cancer represents a substantial global health concern with considerable socioeconomic implications (16, 17). To address the substantial healthcare burden associated with total skin cancer and its subtypes, comprehensive longitudinal analyses of their epidemiological patterns are imperative. Despite the fact that extant literature has documented a persistent increase in the disease burden associated with various skin cancer subtypes (2, 3), these investigations predominantly focus on individual skin cancer subtypes rather than adopting a collective analytical approach (2, 3, 7). Considerable deficiencies remain regarding the long-term epidemiological trends of skin cancer and its comprehensive impact on population health.

Although skin cancer encompasses several types (such as Merkel cell carcinoma, sebaceous carcinoma, and eccrine/apocrine carcinoma), the GBD 2021 database estimates the burden of only three major types: SCC, BCC and melanoma, which together account for more than 90% of all skin cancer cases (18). Therefore, this research focuses on these three types as the primary subjects for analyzing the global burden of skin cancer. Through conducting a comprehensive epidemiological analysis, we aim to inform evidence-based prevention and treatment strategies, ultimately contributing to optimized global health outcomes.

## 2 Methods

### 2.1 Data sources

Data for this investigation were obtained from the 2021 Global Burden of Disease (GBD) database (19), which represents the most comprehensive global health database currently available. Compared with GBD 2019, GBD 2021 not only incorporates new disease categories, refined age stratification, additional data sources, expanded subnational data coverage, and enhanced SDI analysis, but also demonstrates significant methodological advantages. Regarding uncertainty quantification, the number of uncertainty sampling iterations was adjusted to 500, which significantly improved computational efficiency while maintaining accuracy. In terms of modeling tools, the optimization of DisMod-MR 2.1/ST-GPR priors, the addition of count models to CODEm, and the introduction of MR-BRT calibration enhanced rare cause estimation and out-of-sample robustness. Additionally, completeness checks, re-mapping algorithms, and uncertainty propagation steps were employed by IHME for countries with sparse cancer-registry data (detailed procedures are provided in Supplementary material). These improvements render GBD 2021 superior to earlier versions in terms of data completeness and estimation precision; consequently, this study was conducted based on GBD 2021 (19).

The SDI represents a composite indicator of a country’s lag-distributed income per capita, average years of schooling, and the total fertility rate in females younger than 25 years of age (20). Countries and regions were classified by the GBD into different developmental levels based on the following SDI thresholds: low SDI regions [0–0.4658], low-middle SDI regions [0.4658–0.6188], middle SDI regions [0.6188–0.7120], high-middle SDI regions [0.7120–0.8103], and high SDI regions [0.8103–1.0000]. This classification system enables systematic analysis of the impact of socioeconomic development levels on health outcomes.

This research retrieved global epidemiological data for melanoma, BCC and SCC in males and females across all age groups from the Global Health Data Exchange query tool.^1^

### 2.2 Statistical analysis

Incidence and DALYs, along with their ASR and corresponding 95% uncertainty intervals (UIs), constituted the primary metrics for quantifying the burden of total skin cancer and its subtypes. Based on the GBD 2021 world population age standard per 100,000 persons, the ASRs of incidence and DALYs were typically described as follows.


$$\mathrm{ASR}=\frac{\sum_{i=0}^{n}a_{i}w_{i}}{\sum_{i=0}^{n}w_{i}}$$

$a_{i}$ represents the ASR for the i age group, $w_{i}$ represents the number of individuals in the corresponding age group in the GBD standard population and n represents the total number of age groups (21). The relative changes in skin cancer incidence and DALYs from 1990 to 2021 were calculated as follows (22).


$$\text{Relative change}=\frac{\text{Numbe}r_{2021}-\text{Numbe}r_{1990}}{\text{Numbe}r_{1990}}\times 100\%$$

To assess the long-term trends and calculate the average annual changes, the estimated annual percentage change (EAPC) for ASIR and ASDR from 1990 to 2021, along with their corresponding 95% confidence intervals (CIs) were calculated. The EAPC was described as follows (23).


In(ASR)=α+β(calendar year)+ε

EAPC=100×(exp(β)−1)

A negative EAPC indicates a decreasing trend, and vice versa (24).

A local regression smoothing model (LOESS) using the “geom_smooth” function from “ggplot2” (version 3.4.2) was applied to assess the relationship between skin cancer burden, SDI, and ASRs. Smoothing parameters were established as span = 0.5, degree = 2, and the tri-cube kernel. For the geom_smooth() function, the following parameters were specified: geom_smooth (method = “loess,” formula = y ~ x, span = 0.5, degree = 2, se = FALSE).

Age-period-cohort model analysis, decomposition analysis, cross-country inequality analysis and predictive analysis (Supplementary Data) were performed.

Age-standardized rates were calculated exclusively using the GBD 2021 world standard population, while 95% uncertainty intervals were obtained from the GBD 2021 database.

The above statistical analyses were conducted using Rstudio.

## 3 Results

### 3.1 Temporal patterns from 1990 to 2021

**Figure 1:**
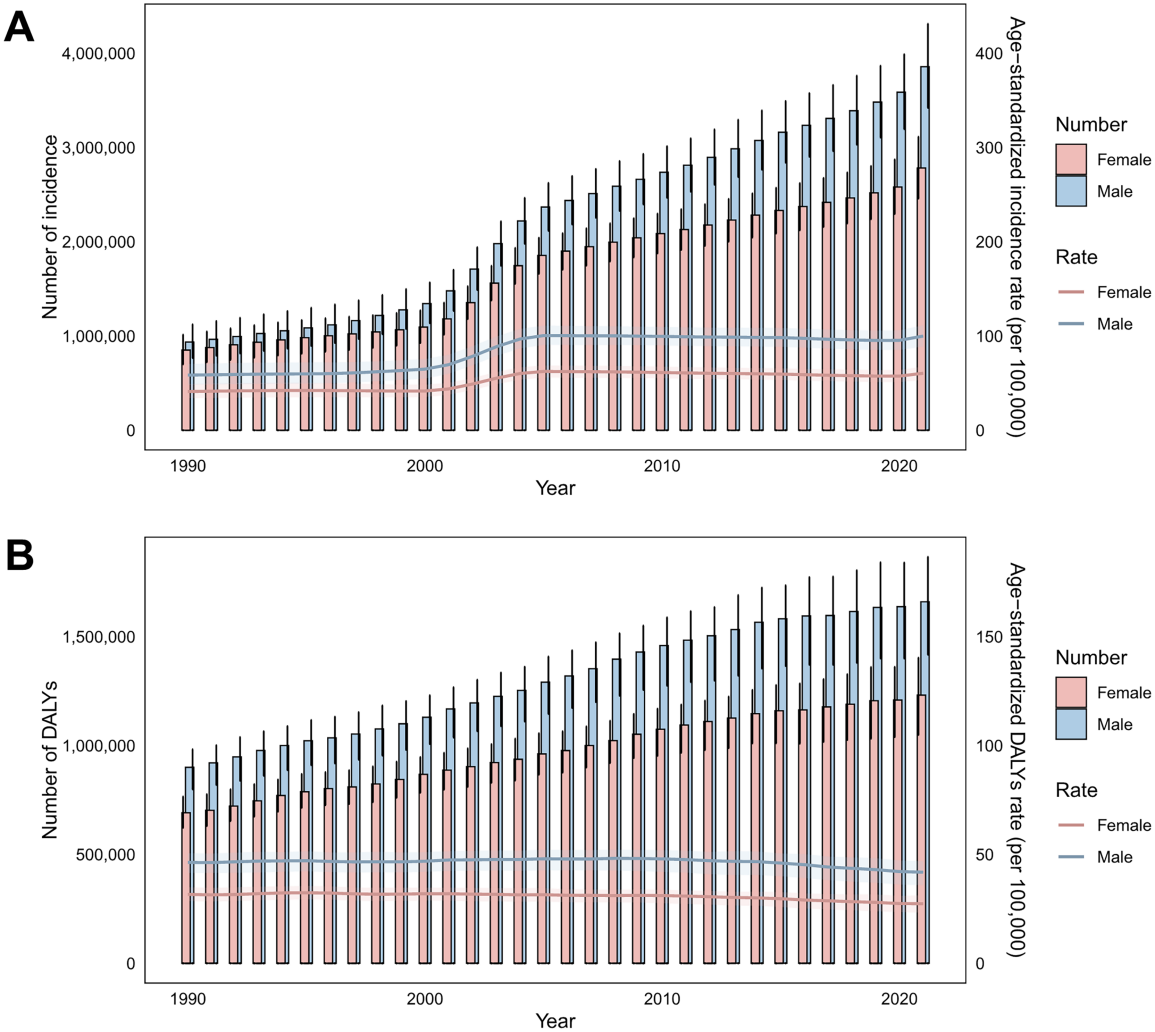
Temporal trends in incidence cases, ASIRs, DALYs, and ASDRs for total skin cancer by sex (1990–2020). **(A)** Incidence and ASIRs; **(B)** DALYs and ASDRs. Total skin cancer includes malignant skin melanoma, squamous-cell carcinoma and basal-cell carcinoma. The pink or blue regions around the curve represent the upper and lower limits of the 95% uncertainty interval (UI). DALYs, disability-adjusted life-years.

**Figure 2:**
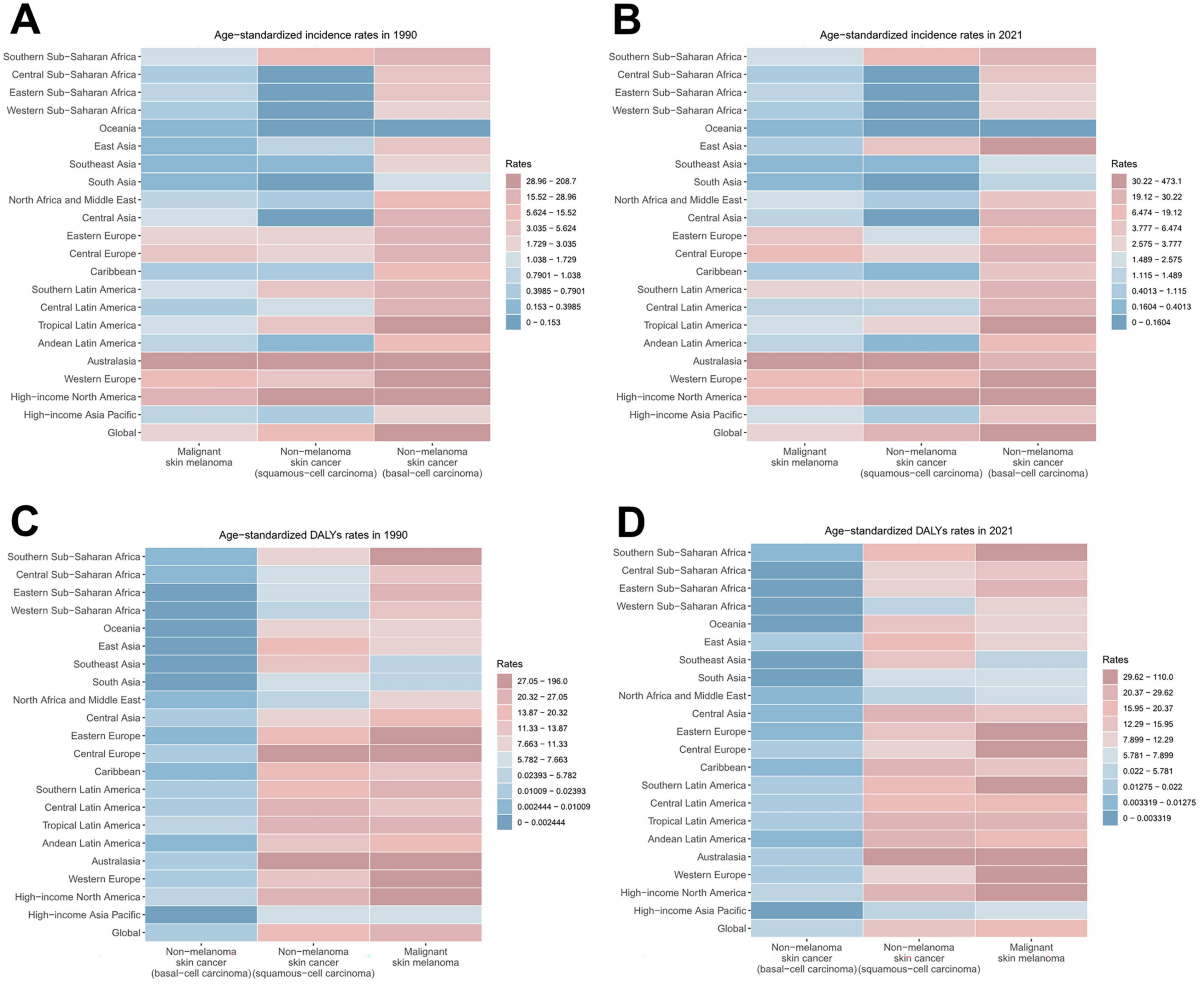
ASIRs and ASDRs for skin cancer subtypes by region, 1990 and 2021. **(A)** ASIRs in 1990; **(B)** ASIRs in 2021; **(C)** ASDRs in 1990; **(D)** ASDRs in 2021. ASIR, age-standardized rate of incidence; ASDR, age-standardized rate of DALYs; DALYs, disability-adjusted life-years.

From 1990 to 2021, the global ASIR of total skin cancer increased from 48.03 to 77.66 per 100,000 population (EAPC = 1.94). Conversely, the global ASDR of total skin cancer decreased from 38.35 to 33.96 per 100,000 population (EAPC = −0.32) (Table 1; Figures 1, 2).

**Table 1:** Global and regional incidence and DALYs of total skin cancer in 1990 and 2021, with age-standardized rates and EAPCs from 1990 to 2021.

Characteristic	Incidence					DALYs				
	All ages number (95% UI), 1990	Age-standardized rate (95% UI), 1990	All ages number (95% UI), 2021	Age-standardized rate (95% UI), 2021	EAPC (95% CI), 1990–2021	All ages number (95% UI), 1990	Age-standardized rate (95% UI), 1990	All ages number (95% UI), 2021	Age-standardized rate (95% UI), 2021	EAPC (95% CI), 1990–2021
Global										
Male	935,684 (765,959 to 1,123,410)	58.51 (48.41 to 70.34)	3,857,633 (3,419,815 to 4,311,762)	99.92 (88.87 to 111.43)	2.17 (1.73 to 2.61)	900,117 (798,500 to 982,623)	46.33 (41.47 to 50.54)	1,660,168 (1,416,740 to 1,866,300)	41.84 (35.88 to 46.9)	−0.22 (−0.34 to −0.09)
Female	850,279 (698,724 to 1,012,931)	41.05 (34.04 to 49.02)	2,782,317 (2,458,445 to 3,113,232)	60.49 (53.47 to 67.58)	1.57 (1.19 to 1.95)	691,224 (622,337 to 765,257)	31.64 (28.57 to 34.96)	1,231,540 (1,048,608 to 1,403,398)	27.37 (23.25 to 31.27)	−0.45 (−0.54 to −0.35)
Both	1,785,964 (1,468,745 to 2,133,473)	48.03 (39.76 to 57.35)	6,639,951 (5,876,877 to 7,424,890)	77.66 (68.91 to 86.6)	1.94 (1.54 to 2.35)	1,591,342 (1,455,828 to 1,706,278)	38.35 (35.18 to 40.99)	2,891,709 (2,543,935 to 3,175,526)	33.96 (29.91 to 37.32)	−0.32 (−0.43 to −0.22)
SDI region										
Low SDI	8,437 (6,182 to 10,561)	3.43 (2.51 to 4.27)	20,215 (14,721 to 25,452)	3.5 (2.53 to 4.38)	0.03 (0.01 to 0.06)	54,538 (34,010 to 69,978)	19.97 (12.45 to 25.66)	137,016 (78,318 to 183,680)	21.6 (12.38 to 28.72)	0.19 (0.14 to 0.25)
Low-middle SDI	34,555 (27,648 to 41,251)	5.72 (4.65 to 6.79)	77,433 (58,775 to 95,499)	5.24 (4.02 to 6.44)	0.26 (0.1 to 0.42)	103,289 (79,416 to 131,346)	15.2 (11.64 to 19.06)	289,358 (220,214 to 351,101)	19.09 (14.78 to 23.1)	0.8 (0.76 to 0.83)
Middle SDI	125,678 (103,742 to 146,411)	12.12 (10.13 to 14.12)	620,104 (494,278 to 737,331)	22.94 (18.48 to 27.18)	1.26 (1.02 to 1.49)	296,091 (238,959 to 337,591)	26.18 (21.4 to 29.76)	708,785 (559,555 to 819,020)	26.72 (21.22 to 30.75)	0.12 (0.05 to 0.19)
High-middle SDI	201,107 (173,130 to 230,553)	20.8 (18.07 to 23.76)	686,675 (565,014 to 814,876)	35.33 (29.2 to 41.72)	0.65 (0.41 to 0.9)	417,666 (384,669 to 450,223)	41.71 (38.44 to 44.86)	718,533 (636,639 to 804,671)	38.49 (34.06 to 43.1)	−0.24 (−0.38 to −0.11)
High SDI	1,415,176 (1,153,258 to 1,703,684)	128.94 (105.89 to 154.66)	5,233,719 (4,710,776 to 5,778,378)	256.81 (230.91 to 282.59)	2.91 (2.42 to 3.4)	717,264 (691,313 to 746,469)	68.65 (66.28 to 71.39)	1,034,494 (960,069 to 1,117,219)	55.68 (52.19 to 59.84)	−0.53 (−0.69 to −0.37)
GBD region										
Andean Latin America	3,420 (3,053 to 3,831)	16.81 (15.04 to 18.79)	7,825 (6,035 to 9,640)	13.16 (10.22 to 16.18)	−0.97 (−1.18 to −0.75)	7,121 (5,555 to 9,247)	31.98 (24.95 to 41.24)	24,348 (18,555 to 30,241)	40.47 (30.81 to 50.12)	0.8 (0.66 to 0.93)
Australasia	32,677 (26,811 to 39,261)	142.74 (117.81 to 170.72)	64,626 (52,207 to 78,930)	127.75 (103.61 to 154.68)	−0.55 (−0.77 to −0.34)	51,928 (48,896 to 55,008)	227.87 (214.51 to 241.45)	68,468 (61,916 to 75,384)	141.67 (129.41 to 154.81)	−1.46 (−1.76 to −1.16)
Caribbean	2,025 (1,597 to 2,430)	7.63 (6.02 to 9.2)	3,636 (2,840 to 4,452)	6.78 (5.29 to 8.3)	−0.54 (−0.63 to −0.45)	8,002 (7,311 to 9,341)	30.17 (27.62 to 35.1)	21,581 (18,463 to 24,979)	40.15 (34.29 to 46.54)	0.91 (0.76 to 1.06)
Central Asia	11,270 (8,813 to 13,744)	24.23 (19.13 to 29.2)	19,826 (15,105 to 24,613)	24.37 (19.02 to 29.61)	0.01 (−0.02 to 0.03)	14,214 (12,663 to 15,785)	28.7 (25.37 to 32.02)	28,935 (25,467 to 32,749)	35.62 (31.53 to 40.19)	0.66 (0.43 to 0.88)
Central Europe	41,967 (37,210 to 47,152)	28.7 (25.59 to 32.2)	76,909 (62,996 to 93,151)	36 (29.51 to 43.05)	1.05 (0.85 to 1.26)	112,130 (106,265 to 119,490)	79.59 (75.3 to 84.91)	134,732 (122,485 to 146,705)	70.13 (63.51 to 76.46)	−0.41 (−0.55 to −0.27)
Central Latin America	25,363 (20,038 to 30,329)	29.97 (24.04 to 36.03)	76,072 (60,080 to 92,078)	30.36 (24.26 to 36.74)	0.05 (0.04 to 0.06)	36,319 (35,279 to 37,258)	39.96 (38.56 to 41.07)	92,612 (82,497 to 103,472)	36.59 (32.62 to 40.85)	−0.36 (−0.45 to −0.26)
Central Sub-Saharan Africa	1,190 (874 to 1,515)	5.07 (3.76 to 6.37)	3,100 (2,264 to 3,993)	5.13 (3.79 to 6.52)	0.05 (0.02 to 0.07)	5,037 (3,103 to 7,647)	19.55 (11.78 to 29.77)	15,189 (8,196 to 24,375)	23.26 (11.94 to 36.69)	0.67 (0.55 to 0.78)
East Asia	43,412 (34,121 to 52,265)	4.89 (3.95 to 5.88)	806,098 (649,114 to 959,400)	36.93 (30.25 to 43.62)	4.42 (3.67 to 5.18)	237,183 (177,595 to 294,533)	25.29 (19.42 to 31.37)	530,820 (385,323 to 671,615)	25.69 (18.67 to 32.42)	0.25 (0.12 to 0.38)
Eastern Europe	55,003 (44,680 to 66,192)	20.15 (16.54 to 24.12)	91,507 (74,626 to 111,053)	26.97 (21.93 to 32.34)	0.98 (0.92 to 1.04)	141,734 (135,180 to 149,412)	53.74 (51.23 to 56.71)	217,492 (198,959 to 237,698)	69.71 (63.61 to 76.28)	0.63 (0.35 to 0.91)
Eastern Sub-Saharan Africa	4,110 (3,035 to 5,076)	4.96 (3.64 to 6.12)	9,956 (7,137 to 12,768)	5.01 (3.55 to 6.38)	−0.02 (−0.03 to −0.01)	31,829 (19,271 to 41,315)	33.36 (19.92 to 43.55)	82,118 (42,556 to 119,658)	35.61 (18.03 to 51.31)	0.13 (0.09 to 0.17)
High-income Asia Pacific	7,702 (6,423 to 9,096)	3.95 (3.31 to 4.64)	26,938 (22,186 to 32,379)	6.21 (5.14 to 7.33)	1.45 (1.38 to 1.51)	27,689 (24,514 to 29,672)	14.2 (12.56 to 15.25)	49,303 (41,739 to 57,424)	12.17 (10.45 to 14.16)	−0.47 (−0.58 to −0.36)
High-income North America	1,168,663 (933,936 to 1,423,378)	332.52 (266.5 to 403.33)	4,731,124 (4,279,928 to 5,203,539)	730.16 (660.78 to 801.2)	3.38 (2.78 to 3.99)	332,392 (317,003 to 349,903)	101.92 (97.45 to 107.09)	467,380 (423,817 to 519,287)	77.96 (71.22 to 86.04)	−0.73 (−0.9 to −0.55)
North Africa and Middle East	29,852 (23,527 to 35,953)	17.52 (14.06 to 20.97)	82,240 (58,407 to 100,728)	17.83 (12.78 to 21.91)	0.05 (−0.27 to 0.36)	49,707 (28,719 to 73,705)	27.02 (15.99 to 38.89)	117,408 (75,002 to 146,670)	25.01 (16.07 to 31.34)	−0.1 (−0.22 to 0.02)
Oceania	17 (10 to 30)	0.47 (0.31 to 0.81)	42 (26 to 69)	0.46 (0.3 to 0.75)	−0.01 (−0.03 to 0.01)	569 (378 to 907)	18.58 (12.67 to 28.75)	1,661 (1,081 to 2,485)	20.27 (13.65 to 29.83)	0.35 (0.32 to 0.38)
South Asia	21,214 (15,615 to 27,683)	3.24 (2.39 to 4.16)	58,766 (43,314 to 76,491)	3.7 (2.74 to 4.76)	0.45 (0.41 to 0.48)	163,224 (116,978 to 223,533)	25.13 (17.71 to 33.9)	444,482 (331,475 to 603,745)	28.64 (21.45 to 38.84)	0.38 (0.3 to 0.46)
Southeast Asia	6,776 (5,729 to 8,123)	2.61 (2.22 to 3.1)	12,837 (9,458 to 16,536)	1.97 (1.48 to 2.51)	−0.83 (−1.13 to −0.54)	51,975 (39,738 to 66,590)	18.03 (13.9 to 22.97)	134,194 (101,403 to 165,600)	19.76 (15.11 to 24.27)	0.16 (0.07 to 0.26)
Southern Latin America	12,350 (10,592 to 14,129)	27 (23.26 to 30.74)	24,114 (19,366 to 29,448)	27.9 (22.3 to 33.93)	0.14 (0.06 to 0.21)	18,620 (17,605 to 19,597)	40.44 (38.23 to 42.58)	38,476 (36,015 to 41,135)	45.97 (43.06 to 49.11)	0.42 (0.12 to 0.73)
Southern Sub-Saharan Africa	6,519 (5,032 to 8,042)	23.02 (17.59 to 28.56)	17,783 (13,390 to 22,104)	29.73 (22.34 to 36.82)	0.28 (−0.22 to 0.78)	12,707 (9,620 to 17,855)	41.33 (30.63 to 57.47)	34,486 (22,479 to 43,486)	54.36 (35.48 to 66.96)	1.1 (0.94 to 1.26)
Tropical Latin America	64,134 (55,757 to 71,940)	68.65 (60.02 to 77)	111,205 (91,225 to 131,785)	42.81 (35.24 to 50.46)	−0.09 (−0.55 to 0.38)	49,041 (47,316 to 50,687)	47.35 (45.54 to 49.1)	134,615 (126,312 to 140,802)	52.3 (48.97 to 54.75)	0.25 (0.12 to 0.37)
Western Europe	270,536 (241,886 to 302,748)	48.53 (43.55 to 53.93)	477,783 (392,911 to 574,466)	55.58 (46.23 to 65.9)	0.56 (0.35 to 0.78)	330,935 (318,446 to 341,079)	65.69 (63.53 to 67.63)	491,673 (453,962 to 522,987)	63.72 (59.83 to 67.4)	0.15 (−0.01 to 0.31)
Western Sub-Saharan Africa	3,298 (2,379 to 4,145)	3.4 (2.43 to 4.26)	8,067 (5,553 to 10,250)	3.47 (2.42 to 4.36)	0.06 (0.05 to 0.07)	15,451 (7,539 to 21,212)	14.35 (7.24 to 19.62)	42,682 (15,687 to 61,096)	15.93 (6.24 to 22.29)	0.34 (0.3 to 0.38)

Total skin cancer includes malignant skin melanoma, squamous-cell carcinoma and basal-cell carcinoma. SDI, socio-demographic index; DALYs, disability-adjusted life-years; EAPC, estimated annual percentage change; UI, uncertainty interval; CI, confidence interval.

Notably, although melanoma presented the lowest ASIR among all skin cancer types (2.98 in 1990, 3.56 in 2021) with the minimal annual growth rate (EAPC = 0.65), it constituted the highest disease burden in terms of DALYs (24.33 in 1990, 19.63 in 2021) (Figure 2; Supplementary Figures S1A,B; Supplementary Table S2). BCC demonstrated the highest ASIR among all skin cancer subtypes, rising from 31.67 in 1990 to 51.71 in 2021 per 100,000 population. However, it demonstrated the lowest disease burden in terms of DALYs, as evidenced by an ASDR of merely 0.02 in 2021. Notably, BCC represented the skin cancer subtype with the fastest-growing ASDR (EAPC = 1.64) (Figure 2; Supplementary Figures S1C,D; Supplementary Table S3). Although SCC did not demonstrate prominent ASIR or ASDR values, it exhibited the most accelerated increase in ASIR (EAPC = 2.06) (Figure 2; Supplementary Figures S1E,F; Supplementary Table S4).

A comprehensive longitudinal analysis of the ASIR and ASDR for total skin cancer revealed a general upward trajectory from 1990 to 2005, with the most pronounced acceleration occurring between 2000 and 2005. Subsequently, a declining trajectory emerged from 2006 to 2019. Notably, a resurgence in ASIR was observed from 2020 to 2021, predominantly attributable to the increase in BCC incidence (Figure 1; Supplementary Table S5).

### 3.2 Geographic disparities

The burden of total skin cancer demonstrated substantial geographical heterogeneity. Among 21 GBD regions, High-income North America presented the highest ASIR for total skin cancer (332.52 in 1990, 730.16 in 2021), followed by Australasia (142.74 in 1990, 127.75 in 2021). Notably, Australasia recorded the highest ASDR (227.87 in 1990, 141.67 in 2021), and also experienced the most significant reduction in ASDR (EAPC = −1.46) (Table 1; Figure 2). East Asia experienced the highest growth rate of ASIR (EAPC = 4.42), while High-income North America demonstrated a marginally lower growth rate (EAPC = 3.38). Conversely, other regions displayed relatively stable ASIR patterns (Table 1; Figure 2). In 2021, the highest ASIR for melanoma, BCC, and SCC were documented in Australasia (32.4), High-income North America (473.14), and High-income North America (240.41), respectively (Figure 2; Supplementary Tables S2–S4). In 2021, the highest ASDR for melanoma, BCC, and SCC across 21 regions were identified in Australasia (109.97), High-income North America (0.19), and Australasia (31.68), respectively (Figure 2; Supplementary Tables S2–S4). Notably, East Asia exhibited the most accelerated increases in ASIR (EAPC = 4.73) and ASDR (EAPC = 4.26) for BCC, followed by High-income North America (EAPC of ASIR = 3.65, EAPC of ASDR = 3.41) (Supplementary Table S3). These two regions also demonstrated the most accelerated growth in the ASIR for SCC (Supplementary Table S4).

**Figure 3:**
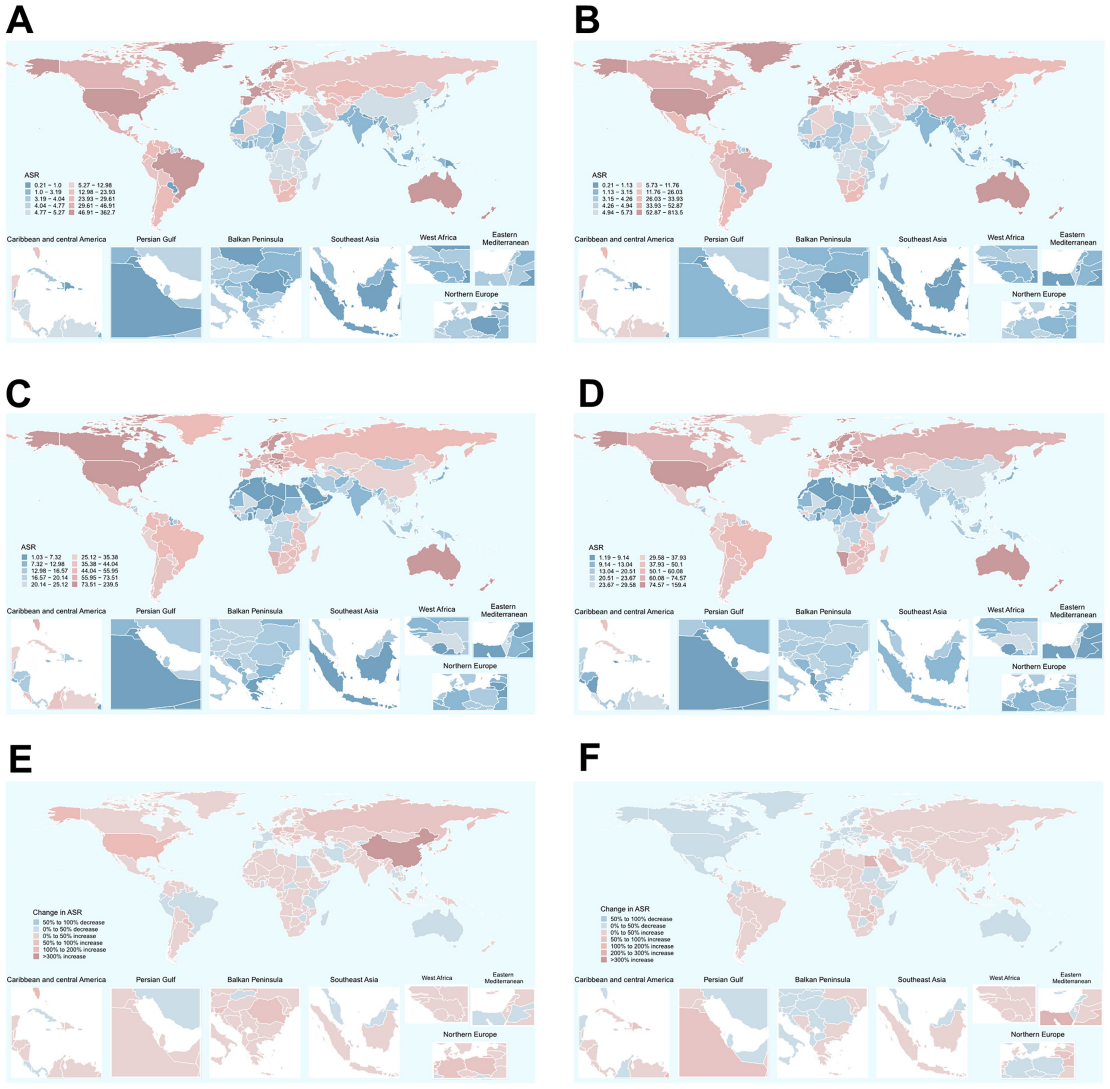
Global distribution of ASIRs and ASDRs of total skin cancer in 1990 and 2021, along with the changes observed from 1990 to 2021. **(A)** ASIRs in 1990; **(B)** ASIRs in 2021; **(C)** ASDRs in 1990; **(D)** ASDRs in 2021; **(E)** The changes in ASIR; **(F)** The changes in ASDR. Total skin cancer includes malignant skin melanoma, squamous-cell carcinoma and basal-cell carcinoma. ASIR, age-standardized rate of incidence; ASDR, age-standardized rate of DALYs; DALYs, disability-adjusted life-years.

Among 204 countries, the highest ASIR of total skin cancer was documented in the United States of America (362.66 in 1990, 813.53 in 2021), with its ASDR ranking 12th globally (Figure 3; Supplementary Table S9). New Zealand demonstrated the second highest ASIR for total skin cancer (121.45 in 1990, 136.04 in 2021), followed by Australia (146.9 in 1990, 126.21 in 2021). These two countries also exhibited the highest ASDR values (Figure 3; Supplementary Table S9). From 1990 to 2021, China experienced the most substantial change (662.31%) and the highest growth rate (EAPC = 4.47) in ASIR for total skin cancer. Saint Kitts and Nevis recorded the highest EAPC for ASDR (4.35), whereas Mauritius demonstrated the greatest relative increase in ASDR (312.88%) (Figure 3; Supplementary Table S9). Among 204 countries in 2021, the highest ASIR for melanoma, BCC, and SCC were observed in New Zealand (38.91), the United States of America (527.05), and the United States of America (269.15), respectively. The highest ASDR for melanoma, BCC, and SCC were identified in Australia (107.18), the United States of America (0.21), and Georgia (64.63), respectively (Supplementary Figure S3; Supplementary Tables S10–S12). For melanoma, Armenia experienced the most rapid increase in ASIR (EAPC = 2.86), and Lesotho experienced highest growth rate in ASDR (EAPC = 1.54). Mauritius demonstrated the most substantial changes in both ASIR (928.69%) and ASDR (822.38%) (Figure 3; Supplementary Table S10). For BCC, Canada (EAPC = 0.55) and the Russian Federation (EAPC = 0.82) demonstrated the most accelerated increases in ASIR and ASDR, respectively. Meanwhile, China exhibited the most pronounced changes in ASIR (740.31%) and ASDR (585.63%) (Figure 3; Supplementary Table S11). For SCC, while Mali (EAPC = 0.3) and Mongolia (EAPC = 9.38) exhibited the most accelerated increases in ASIR and ASDR, respectively. China (565.84%) and Georgia (2019.12%) demonstrated the most substantial changes in ASIR and ASDR, respectively, (Figure 3; Supplementary Table S12).

**Figure 4:**
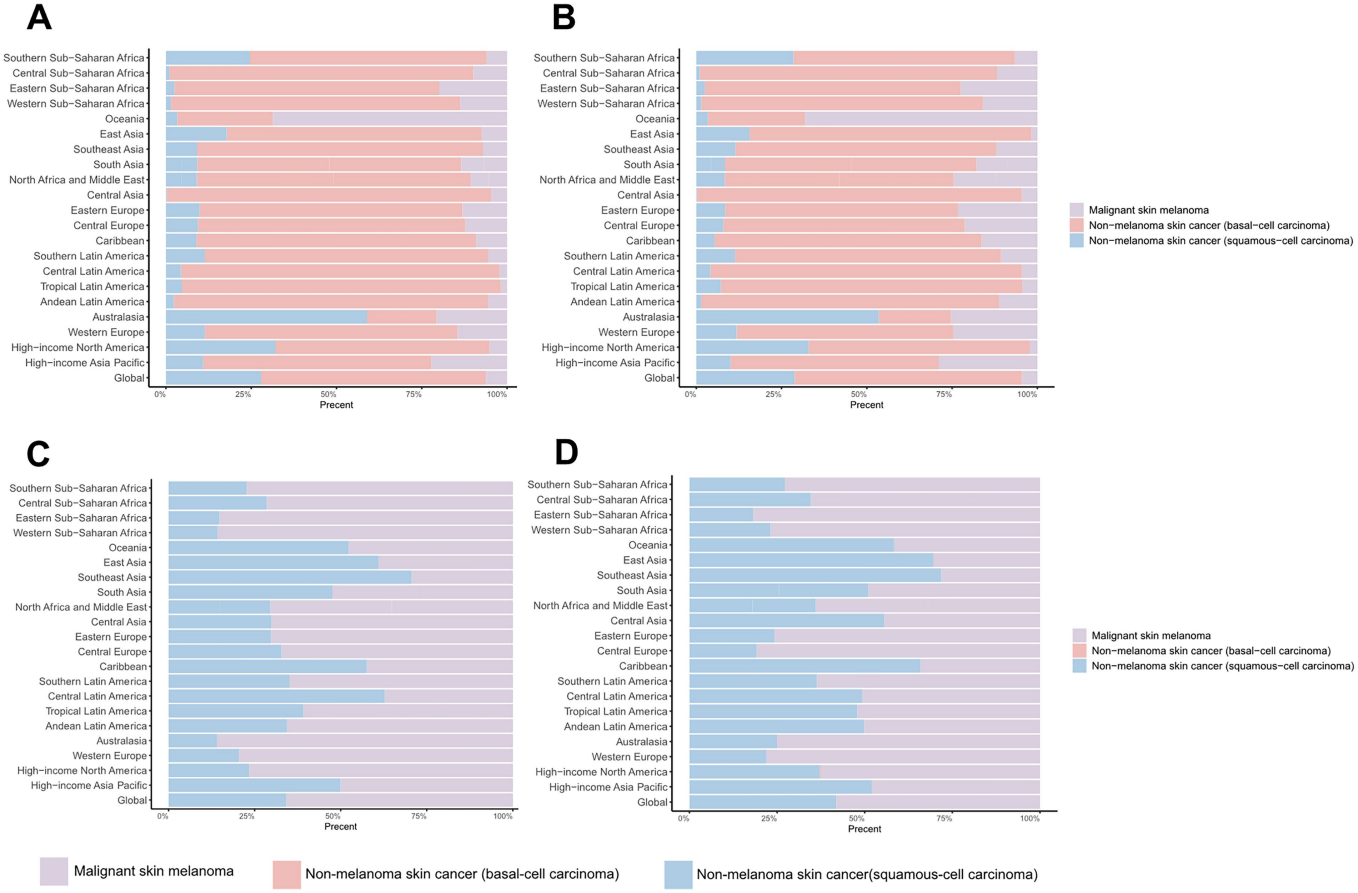
Distribution of skin cancer subtypes across 21 regions in 1990 and 2021. **(A)** ASIR in 1990; **(B)** ASIR in 2021; **(C)** ASDR in 1990; **(D)** ASDR in 2021. ASIR, age-standardized rate of incidence; ASDR, age-standardized rate of DALYs; DALYs, disability-adjusted life-years.

The composition of skin cancer subtypes exhibits significant heterogeneity across regions. Regarding the ASIRs of the three major skin cancer subtypes, BCC constituted the highest proportion of cases (65.94% in 1990, 66.60% in 2021) globally, followed by SCC (27.85% in 1990, 28.82% in 2021) (Figures 4A,B; Supplementary Table S13). Specifically, with the exception of Australia and Oceania, BCC constituted the principal prevalent subtype across all other geographical regions (Figures 4A,B; Supplementary Table S13). However, in terms of DALYs, melanoma contributed the largest proportion (65.71% in 1990, 58.05% in 2021) (Figures 4C,D; Supplementary Table S14). This pattern was consistent across most regions (Figures 4C,D; Supplementary Table S14).

### 3.3 The sex, age, and APC model analysis

**Figure 5:**
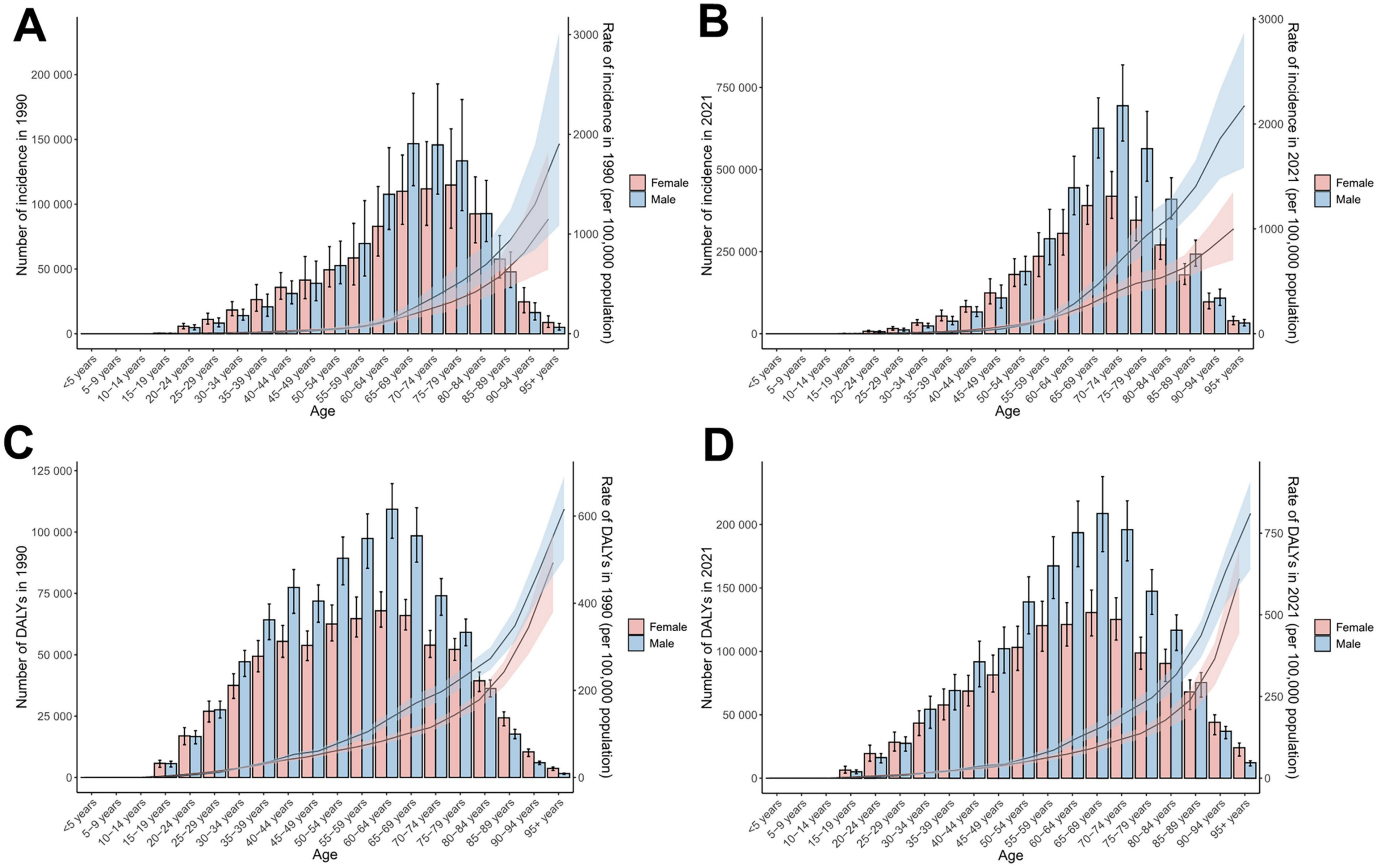
Incidence and DALYs of total skin cancer by sex and age group, with age-standardized rates and 95% uncertainty intervals for 1990 and 2021. **(A)** ASIRs in 1990; **(B)** ASIRs in 2021; **(C)** ASDRs in 1990; **(D)** ASDRs in 2021. Total skin cancer includes malignant skin melanoma, squamous-cell carcinoma and basal-cell carcinoma. The pink or blue regions around the curve represent the upper and lower limits of the 95% uncertainty interval (UI). ASIR, age-standardized rate of incidence; ASDR, age-standardized rate of DALYs; DALYs, disability-adjusted life-years.

Sex-specific differences in ASIR and ASDR for total skin cancer were consistently lower among females compared to males, with the ASIR progression rate in males (EAPC = 2.17) substantially exceeding that of females (EAPC = 1.57) (Table 1; Figures 1, 5; Supplementary Figure S3; Supplementary Tables S15–S18). Sex disparities in ASIRs and ASDRs for total skin cancer and its subtypes demonstrated a positive association with age (Figure 5; Supplementary Figure S3). Specifically, minimal sex variations were observed prior to 55 years of age, after which male ASIR and ASDR surpassed those of females, with the discrepancy progressively increasing with age (Figure 5; Supplementary Figure S3; Supplementary Tables S15–S18). Three skin cancer subtypes demonstrated analogous sex-specific distribution patterns.

**Figure 6:**
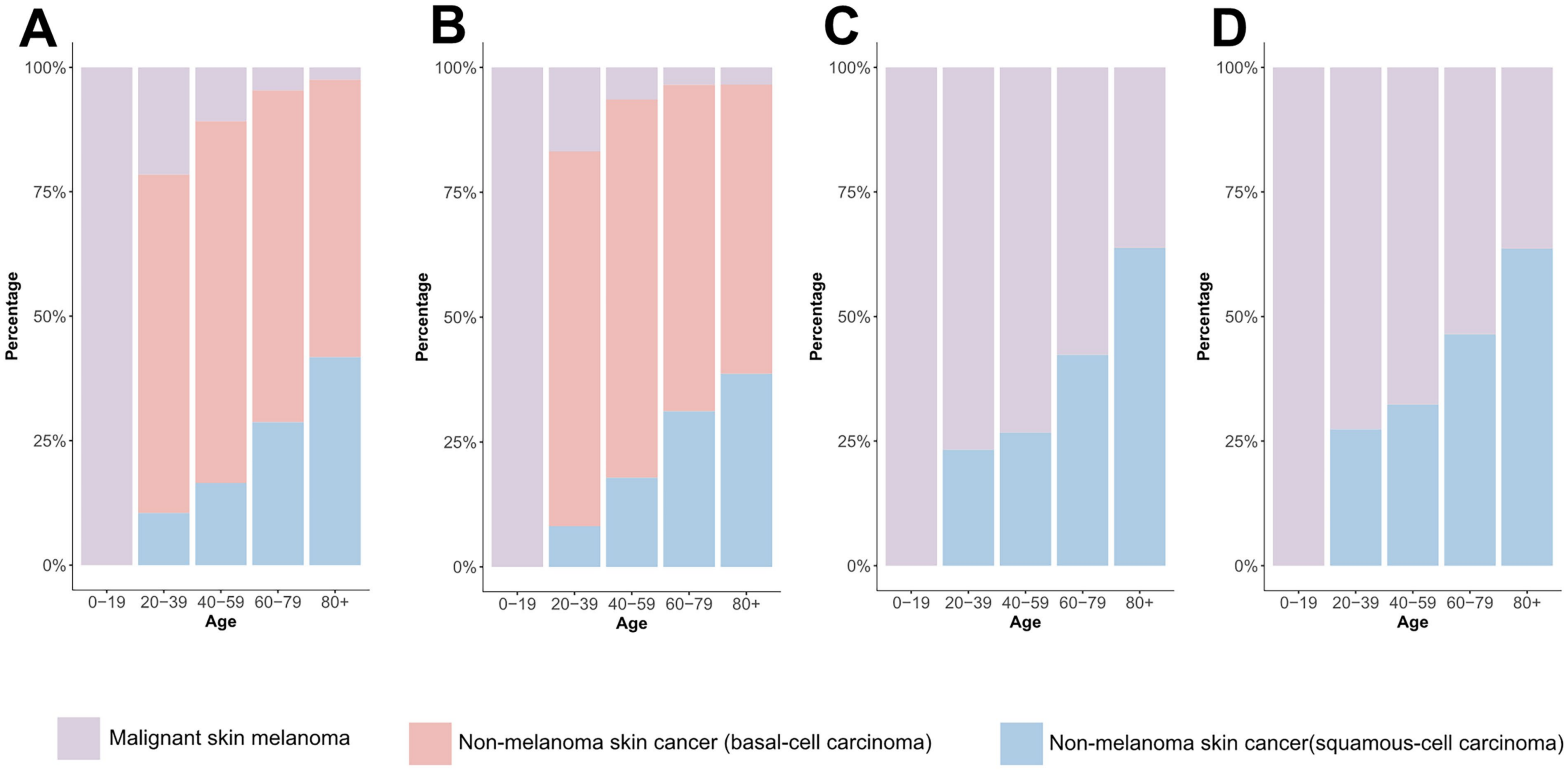
Comparison of age-specific distribution of skin cancer subtypes in 1990 and 2021. **(A)** ASIR in 1990; **(B)** ASIR in 2021; **(C)** ASDR in 1990; **(D)** ASDR in 2021. ASIR, age-standardized rate of incidence; ASDR, age-standardized rate of DALYs; DALYs, disability-adjusted life-years.

Global age distribution patterns of ASIRs and ASDRs for total skin cancer and its subtypes exhibited remarkable consistency across regions. Specifically, ASIRs and ASDRs of total skin cancer and its subtypes demonstrated a positive correlation with advancing age (Figure 5; Supplementary Figure S3; Supplementary Tables S15–S18). Notably, a significant reduction in melanoma ASIR was documented exclusively in males aged over 95 (Supplementary Figures S3A,B; Supplementary Table S16). BCC demonstrated the highest proportion of skin cancer cases and the lowest DALYs proportion across all age groups (Figure 6; Supplementary Tables S19, S20). With increasing age, the proportion of the incidence cases and DALYs for melanoma decreases, while those for BCC and SCC increase (Figure 6; Supplementary Tables S19, S20). In 2021, among individuals over 80, melanoma, BCC and SCC accounted for 3.47%, 57.88%, and 38.65% of all skin cancer cases, respectively. In terms of DALYs, the respective proportions were 36.31%, 0.06%, and 63.63% (Figure 6; Supplementary Tables S19, S20).

**Figure 7:**
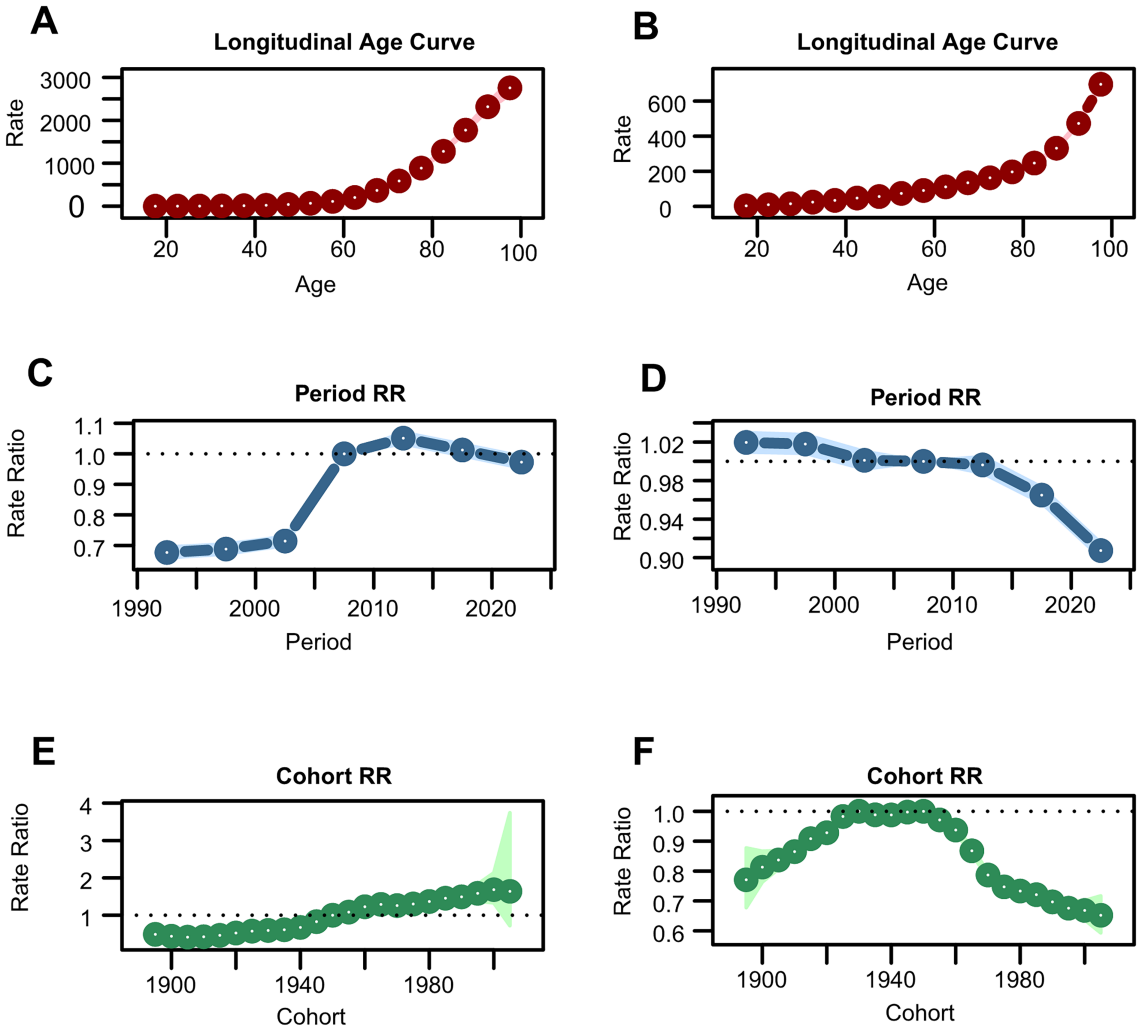
Estimates of age, period, and cohort effects on incidence and DALYs of total skin cancer. **(A)** Age effect on incidence; **(B)** Age effect on DALYs; **(C)** Period effect on incidence; **(D)** period effect on DALYs; **(E)** Cohort effect on incidence; **(F)** Cohort effect on DALYs. Total skin cancer includes malignant skin melanoma, squamous-cell carcinoma and basal-cell carcinoma. DALYs, disability-adjusted life-years.

Additionally, an APC analysis was conducted. Table 2 presents the net drift analysis for total skin cancer. The overall incidence of total skin cancer exhibited a significant annual increase of 1.617% primarily driven by BCC (1.783%) and SCC (1.704%), rather than melanoma (0.691%) (Supplementary Table S3). DALYs demonstrated divergent trends, with total skin cancer and melanoma declined by −0.33% and −0.502%, respectively, while BCC and SCC increased by 1.457% and 0.101% annually (Supplementary Table S3). These findings were consistent with the EAPC analysis results of ASIR and ASDR for total skin cancer and its subtypes (Table 1; Supplementary Tables S2–S4). APC analysis elucidated consistent epidemiological patterns across various metrics. The age-specific effect demonstrated an ascending trajectory in incidence and DALY rates with advancing age for total skin cancer and its subtypes (Figures 7A,B; Supplementary Figures S4A,B,G,H,M,N), corroborating the previously observed trends in Figure 5 and Supplementary Figure S3, with a notable acceleration beyond age 60. The period effect revealed an increasing risk of developing skin cancer after 2002, with a significantly higher risk after 2007 (RR = 1). In terms of DALYs, the total skin cancer risk demonstrated a declining trend, with the RR falling below 1 after 2007, indicating a reduced relative risk of DALYs for skin cancer (Figures 7C,D). Cohort effects analysis identified 1950 as the reference point (RR = 1), with subsequent birth cohorts exhibiting progressively increasing incidence risk and decreasing DALY risk (Figures 7E,F).

**Table 2:** Net drift percentages of incidence and DALYs of total skin cancer and its subtypes.

Disease	Measure name	Net drift		
		Net drift (%/year)	CI Lo	CI Hi
Total skin cancer	Incidence	1.617	1.519	1.716
	DALYs	−0.33	−0.357	−0.302
Malignant skin melanoma	Incidence	0.691	0.645	0.737
	DALYs	−0.502	−0.534	−0.469
Basal-cell carcinoma	Incidence	1.783	1.706	1.86
	DALYs	1.457	1.24	1.674
Squamous-cell carcinoma	Incidence	1.704	1.572	1.835
	DALYs	0.101	0.07	0.132

Total skin cancer includes malignant skin melanoma, squamous-cell carcinoma and basal-cell carcinoma. DALYs, disability-adjusted life-years.

To assess the validity of the APC analysis, deviance, AIC, and BIC were calculated for four nested models (Supplementary Table S21), and the relative performance of each model was evaluated accordingly. The full APC model exhibited substantially lower deviance, AIC, and BIC values compared with all reduced alternatives, thereby providing compelling statistical evidence for the concurrent inclusion of age, period, and cohort effects. This superior model fit confirmed that all three temporal dimensions contributed significantly to the observed trends in skin cancer burden, thereby emphasizing the critical importance of incorporating period and cohort influences—in addition to age effects—when projecting future incidence rates and developing targeted prevention strategies.

### 3.4 The relationship between SDI and the disease burden

**Figure 8:**
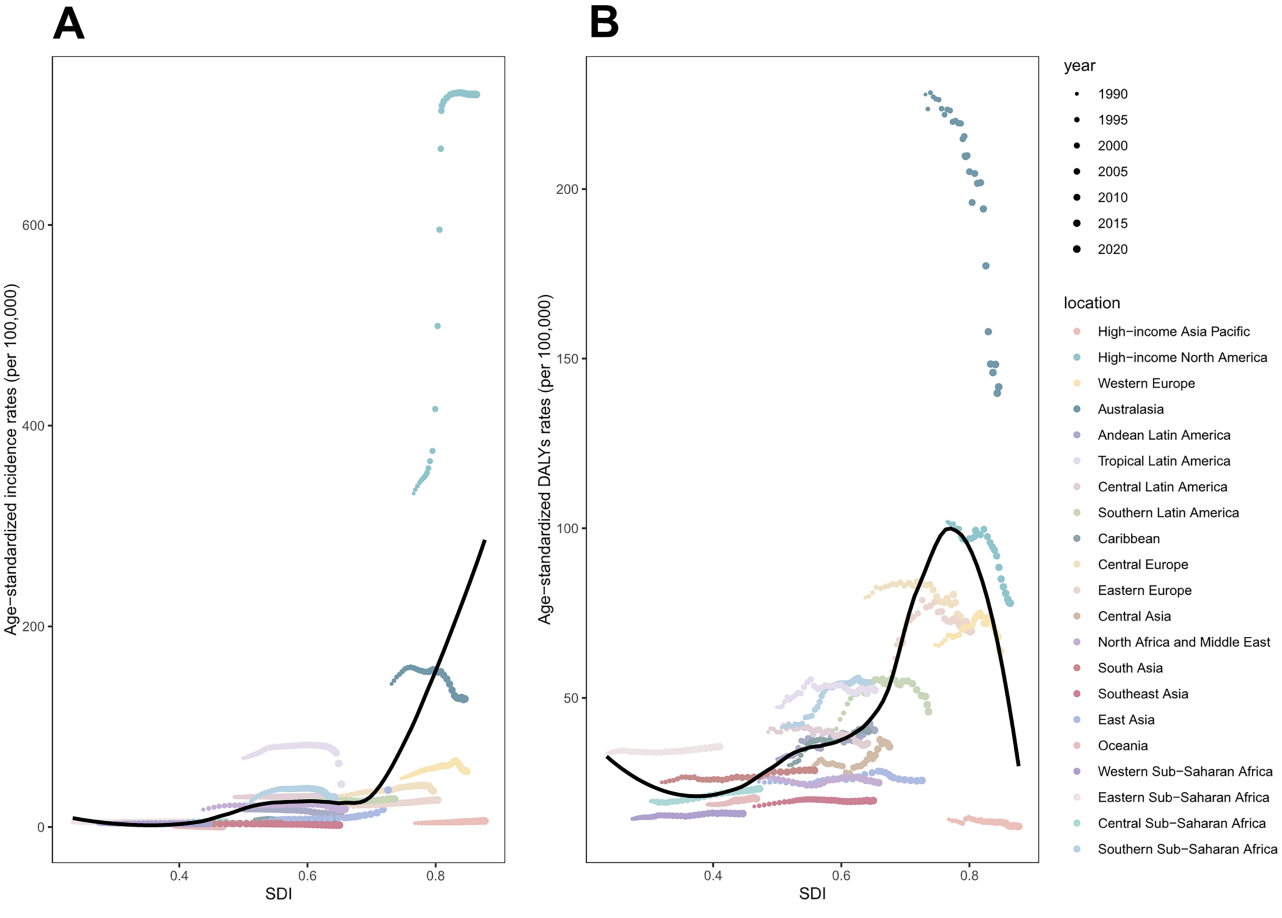
ASIRs and ASDRs of total skin cancer for 21 regions, by SDI (2021), from 1990 to 2021. **(A)** ASIRs; **(B)** ASDRs. Total skin cancer includes malignant skin melanoma, squamous-cell carcinoma and basal-cell carcinoma. 32 points are plotted for each region and show the observed ASIRs or ASDRs for each year from 1990 to 2021. Expected values are shown by the black line. Points above the black line represent a higher-than-expected burden, and those below the line show a lower-than-expected burden. DALYs, disability-adjusted life-years; ASIR, age-standardized rate of incidence; ASDR, age-standardized rate of DALYs; SDI, socio-demographic index.

**Figure 9:**
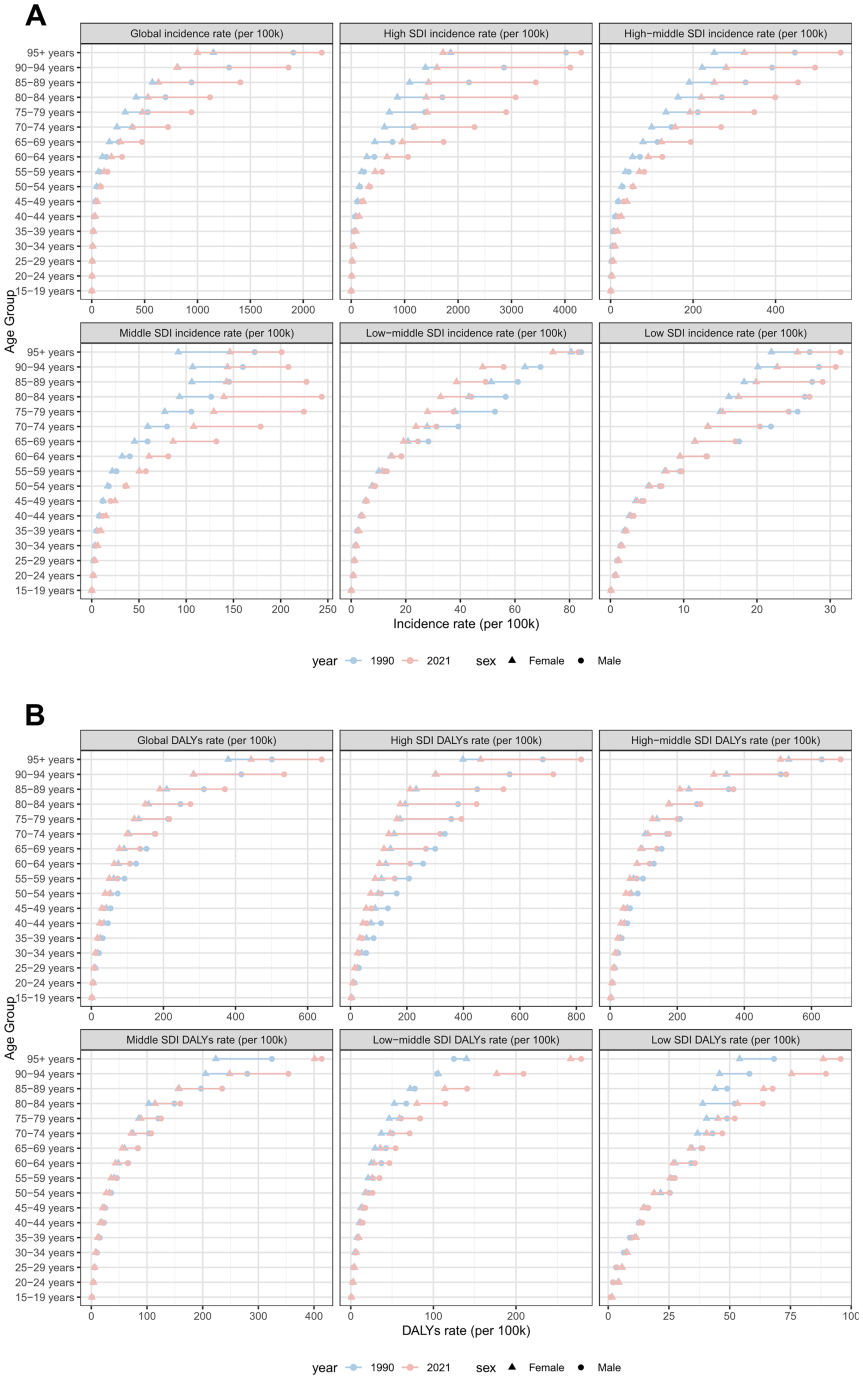
ASIRs and ASDRs of total skin cancer by sex, age group, and SDI, 1990 and 2021. **(A)** ASIRs; **(B)** ASDRs. Total skin cancer includes malignant skin melanoma, squamous-cell carcinoma and basal-cell carcinoma. ASIR, age-standardized rate of incidence; ASDR, age-standardized rate of DALYs; DALYs, disability-adjusted life-years; SDI, socio-demographic index.

From 1990 to 2021, total skin cancer ASIR exhibited a significant positive correlation with SDI across all 21 regions, with a pronounced point at SDI = 0.7 (Figure 8A; Supplementary Figures S6A,B; Supplementary Table S22). SCC and BCC exhibited similar patterns, while melanoma ASIR declined around SDI = 0.8 (Supplementary Figures S5A–C, S6E,F,I,J,M,N; Supplementary Table S22). The total skin cancer ASDR exhibited a U-shaped association with SDI, initially declining, then increasing, and finally decreasing after surpassing the SDI threshold of 0.8 (Figure 8B; Supplementary Figures S6D,E; Supplementary Table S22). The ASDRs for melanoma and SCC exhibited similar trends, whereas BCC ASDR continued increasing beyond SDI = 0.8 (Supplementary Figures S5D–F, S6G,H,K,L,O,P; Supplementary Table S22). Figure 9, Supplementary Figure S7, and Supplementary Tables S23–S26 illustrate ASIRs and ASDRs by SDI region and sex for 1990 and 2021, revealing significant heterogeneity across these parameters and distinct epidemiological patterns.

### 3.5 Decomposition analysis

**Figure 10:**
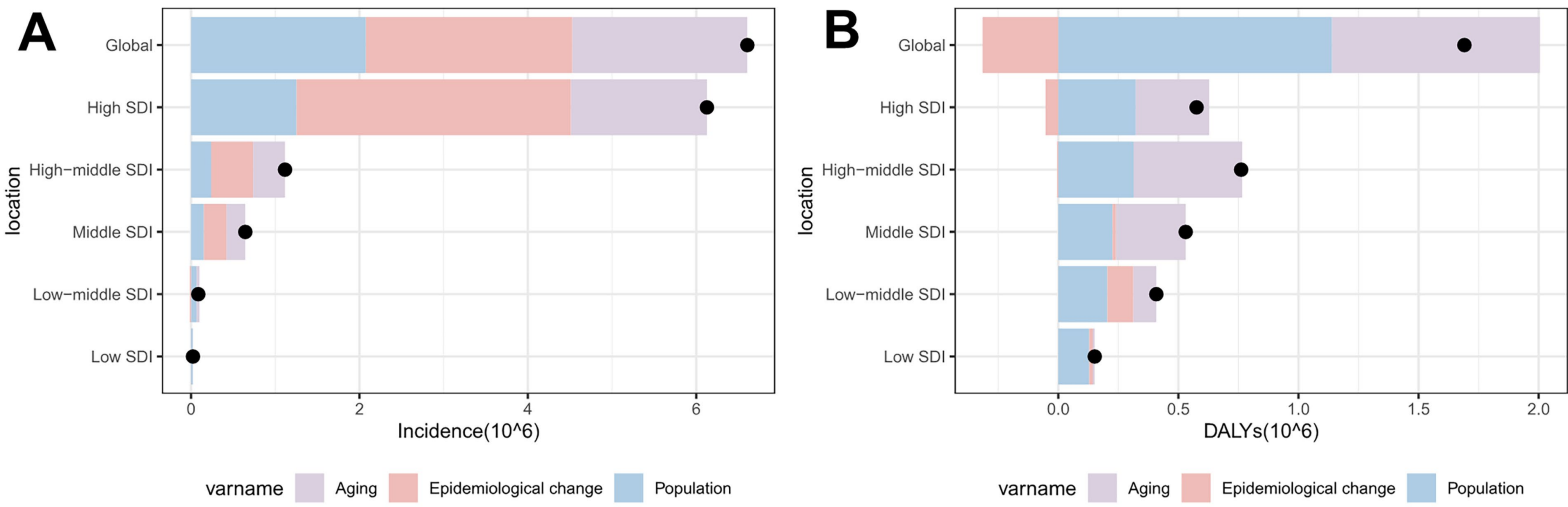
Population‑level determinants of changes in total skin cancer incidence and DALYs globally and across SDI regions from 1990 to 2021: the roles of aging, population growth, and epidemiological factors. **(A)** ASIR; **(B)** ASDR. Total skin cancer includes malignant skin melanoma, squamous-cell carcinoma and basal-cell carcinoma. Black dots represent the total change contributed by all three components. A positive value for each component indicates a corresponding positive contribution in incidence or DALYs, and a negative value indicates a corresponding negative contribution in incidence or DALYs. ASIR, age-standardized rate of incidence; ASDR, age-standardized rate of DALYs; DALYs, disability-adjusted life-years; SDI, socio-demographic index.

Aging (31.49%), population growth (31.43%), and epidemiological changes (37.08%) collectively contributed to the global elevation in total skin cancer ASIR (Figure 10A; Supplementary Table S27), while contributing 51.25%, 67.4%, and −18.64%, respectively, to the decrease in ASDR (Figure 10B; Supplementary Table S27). Epidemiological changes significantly influenced melanoma patterns, accounting for 49.72% of ASIR increase but −49.07% of ASDR change (Supplementary Table S28). Besides, particularly pronounced negative effect on ASDR change was observed in high‑middle SDI regions (−344.54%) (Supplementary Figure S8D; Supplementary Table S28). Three factors have significant effects on the ASIR and ASDR of BCC (Supplementary Figures S8B,E; Supplementary Table S29). In the case of SCC, epidemiological changes had only a minor effect on the increase in ASIR and ASDR (Supplementary Figure S8C,F; Supplementary Table S30), with population growth dominating ASIR increases (93.25%). Besides, population growth (48.92%) and aging (49.5%) significantly contributed to SCC ASDR increases (Supplementary Table S30).

### 3.6 Cross-country inequality analysis

**Figure 11:**
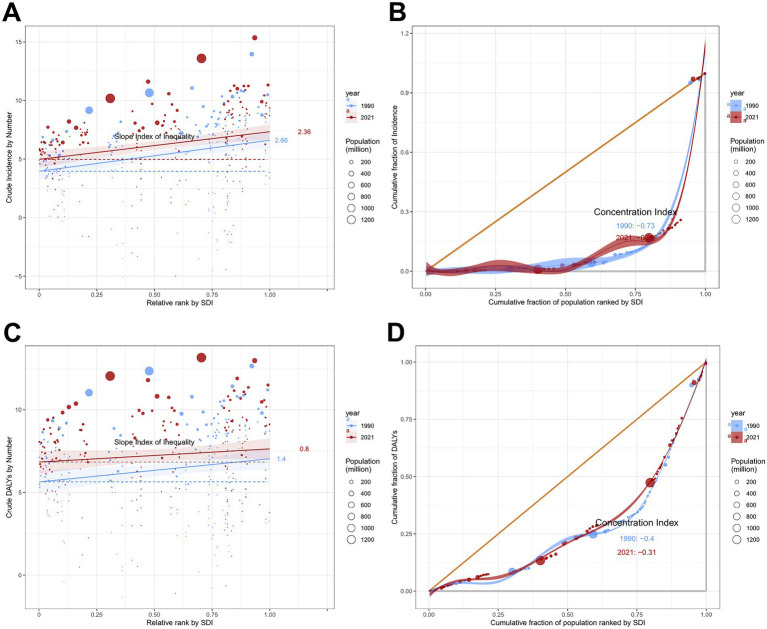
Absolute and relative cross-country inequality in ASIR and ASDR of total skin cancer, 1990–2021. **(A)** Health inequality regression curves for ASIRs; **(B)** Concentration curves for ASIRs; **(C)** Health inequality regression curves for ASDRs; **(D)** Concentration curves ASDRs. Total skin cancer includes malignant skin melanoma, squamous-cell carcinoma and basal-cell carcinoma. ASIR, age-standardized rate of incidence; ASDR, age-standardized rate of DALYs; DALYs, disability-adjusted life-years.

Total skin cancer incidence and DALYs demonstrated significant absolute and relative cross-country inequalities, with higher-SDI countries exhibiting greater burdens (Figure 11; Table 3). Slope Index of Inequalities (SIIs) indicate a diminishing disparity in skin cancer incidence and DALYs, reducing from 2.66 and 1.40 in 1990 to 2.36 and 0.80 in 2021, respectively (Figures 11A,C; Table 3). Concentration indexes (CIs) also indicated a reduction in the inequalities of skin cancer incidence and DALYs, reducing from −0.73 and −0.40 in 1990 to −0.69 and −0.31 in 2021 (Figures 11B,D; Table 3). With the exception of an increase in the absolute inequality of melanoma incidence (SII rose from 2.50 in 1990 to 2.71 in 2021) (Table 3; Supplementary Figure S9), declining trends were observed in the absolute and relative inequalities of other skin cancer subtypes.

**Table 3:** Slope index of inequality and concentration index in global ASIR and ASDR of total skin cancer and its subtypes in 1990 and 2021.

Disease	Incidence				DALYs			
	Slope index of inequality (SII)		Concentration index (CI)		Slope index of inequality (SII)		Concentration index (CI)	
	1990	2021	1990	2021	1990	2021	1990	2021
Total skin cancer	2.66 (1.33 to 3.99)	2.36 (1.11 to 3.62)	−0.73 (−0.79 to −0.67)	−0.69 (−0.75 to −0.63)	1.40 (0.17 to 2.63)	0.80 (−0.30 to 1.91)	−0.40 (−0.52 to −0.28)	−0.31 (−0.43 to −0.19)
Malignant skin melanoma	2.50 (1.24 to 3.77)	2.71 (1.53 to 3.90)	−0.72 (−0.83 to −0.61)	−0.64 (−0.75 to −0.53)	1.39 (0.13 to 2.65)	0.92 (−0.21 to 2.05)	−0.50 (−0.67 to −0.33)	−0.38 (−0.55 to −0.21)
Basal-cell carcinoma	2.58 (1.26 to 3.90)	2.16 (0.88 to 3.43)	−0.69 (−0.73 to −0.65)	−0.66 (−0.70 to −0.62)	2.73 (1.43 to 4.03)	2.31 (1.05 to 3.56)	−0.67 (−0.74 to −0.60)	−0.62 (−0.69 to −0.55)
Squamous-cell carcinoma	4.27 (2.68 to 5.87)	4.28 (2.76 to 5.80)	−0.82 (−0.89 to −0.75)	−0.77 (−0.84 to −0.70)	1.76 (0.47 to 3.05)	0.74 (−0.36 to 1.84)	−0.22 (−0.22 to −0.22)	−0.22 (−0.22 to −0.22)

Total skin cancer includes malignant skin melanoma, squamous-cell carcinoma and basal-cell carcinoma. ASIR, age-standardized rate of incidence; ASDR, age-standardized rate of DALYs; DALYs, disability-adjusted life-years.

### 3.7 Predictive analysis

**Figure 12:**
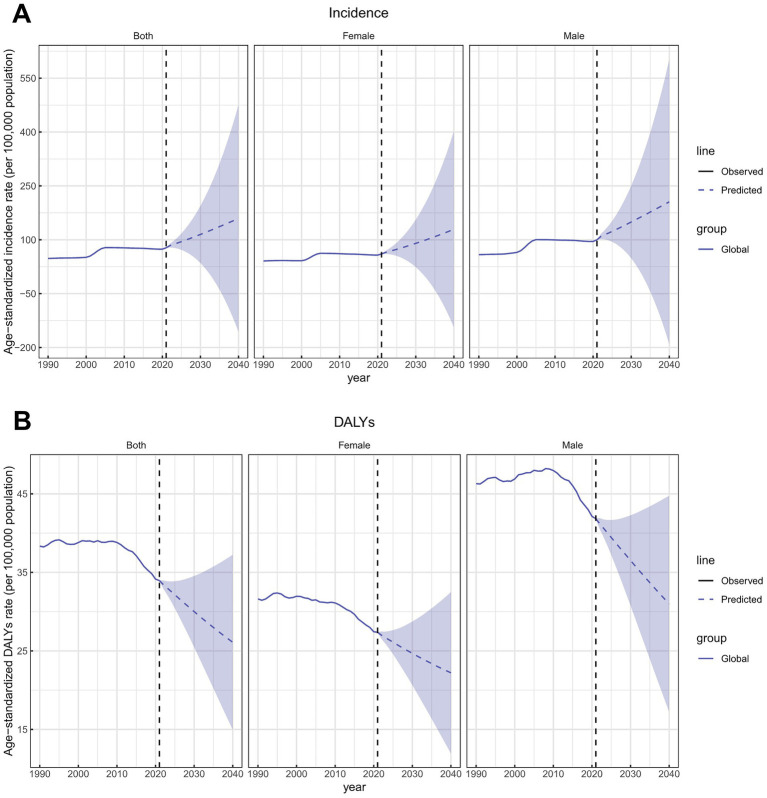
Future forecasts of ASIRs and ASDRs of total skin cancer. **(A)** ASIRs; **(B)** ASDRs. Total skin cancer includes malignant skin melanoma, squamous-cell carcinoma and basal-cell carcinoma. The purple region shows the upper and lower limits of the 95% uncertainty interval (UI). ASIR, age-standardized rate of incidence; ASDR, age-standardized rate of DALYs; DALYs, disability-adjusted life-years.

The global ASIR of total skin cancer was projected to increase to 159.51 per 100,000 population by 2040, whereas the ASDR was expected to decrease to 26.12 per 100,000 population (Figure 12; Supplementary Table S31). Specifically, the ASIRs for both BCC and SCC were projected to increase, while melanoma ASIR was expected to decline (Supplementary Figures S10A,C,E; Supplementary Tables S32–S34). ASDRs for both melanoma and SCC were projected to decrease, whereas BCC was expected to increase (Supplementary Figures S10B,D,F; Supplementary Tables S32–S34). The projected incidence and DALY rates also demonstrated sex-specific heterogeneity. The projected ASIRs and ASDRs for females are consistently lower than those for males, with sex disparities more prominent in ASDR than ASIR projections (Figure 12; Supplementary Figure S10; Supplementary Tables S31–S34).

## 4 Discussion

Skin cancer represents a substantial public health concern, imposing a significant burden on healthcare systems worldwide (25). Although multiple studies have documented the burden of melanoma, SCC, and BCC, these investigations predominantly have utilized GBD 2017 and GBD 2019 datasets (3, 4) and have lacked comprehensive global health inequality analyses or future projections. The present study provides a novel and comprehensive assessment of the global burden of total skin cancer, while simultaneously conducting a detailed comparative analysis of the three major subtypes. The main findings are as follows: First, skin cancer ASIR increased from 1990 to 2021, while ASDR significantly decreased. Second, geographical heterogeneity existed in distribution of histological subtypes. Third, skin cancer burden demonstrated age-dependent progression with compositional variance in subtypes across age groups. Fourth, sex disparities intensified beyond age 55, with increasing longitudinal divergence. Fifth, both the ASIR and ASDR of total skin cancer showed non‑linear associations with SDI. Sixth, international disparities in skin cancer burden demonstrated a decreasing trend. Finally, projections to 2040 indicate a continued increase in total skin cancer ASIR, accompanied by a persistent decline in total skin cancer ASDR.

From 1990 to 2021, the ASIR of total skin cancer demonstrated an upward trend, with comparable patterns observed across three major subtypes. This finding corroborates previous research (2–4, 16, 26) that documented an elevated incidence of melanoma, BCC and SCC. Ultraviolet radiation (UVR) is the major etiologic agent in the development of skin cancers. UVR contributes to skin cancer through a dual mechanism involving direct DNA damage and indirect immunosuppressive and oxidative damage. UVB radiation (280–320 nm) is directly absorbed by thymine bases in DNA, resulting in the formation of cyclobutane pyrimidine dimers (CPDs) and 6–4 photoproducts (27). UVA radiation (320–400 nm) primarily induces DNA damage through the excitation of endogenous chromophores, which subsequently generate reactive oxygen species (28). When UV-induced DNA damage is not efficiently repaired, mutations accumulate in key driver genes such as TP53 and BRAF, thereby promoting skin carcinogenesis (28). Furthermore, ultraviolet radiation has been shown to induce immunosuppression through the activation of Langerhans cell migration and the subsequent induction of IL-4 secretion by NKT cells (29). Advancing age is associated with compromised DNA repair capacity, including the decline of nucleotide excision repair (NER) and base excision repair (BER) mechanisms (30); diminished immune surveillance, characterized by reduced Langerhans cell populations (31) and impaired T-cell function; and the establishment of a pro-carcinogenic microenvironment resulting from the accumulation of senescent cells, such as fibroblasts (32), which secrete senescence-associated secretory phenotype (SASP) factors. These interconnected processes synergistically promote mutation accumulation in skin cells, ultimately facilitating malignant transformation and the development of skin cancer. Beyond the established impact of ultraviolet radiation exposure (33–35) and demographic aging (36), the extensive implementation of therapeutic modalities has been associated with the increased skin cancer prevalence. The use of immunosuppressive agents, including azathioprine (37) and calcineurin inhibitors (38), is associated with DNA damage while simultaneously inhibiting DNA repair mechanisms. Consequently, organ transplant recipients exhibit substantially elevated risks of BCC and SCC, with incidence rates exceeding those in the general population by orders of magnitude (39, 40). Furthermore, radiation therapy is characterized by the generation of free radicals and reactive oxygen intermediates, thereby damaging rapidly dividing cells in the epidermal basal layer and dermis (41). This process leads to reversible or irreversible DNA damage (41) and has been associated with a 6.3-fold increase in BCC and SCC risk among pediatric cancer survivors who have undergone radiation therapy (42, 43). Additionally, thiazide diuretics are characterized by photosensitizing properties that induce abnormal cutaneous reactions to ultraviolet radiation, thereby establishing a distinct dose–response relationship with BCC and SCC development (44). Previous studies have demonstrated that the risk of BCC and SCC exhibits a dose-dependent relationship with phototherapy sessions utilizing UVA, UVB, and occasionally visible light (45–47). These factors cumulatively account for the more substantial ASIR elevation observed in SCC and BCC relative to melanoma. Consequently, enhanced surveillance and systematic management of SCC and BCC burden are imperative in populations with exposure to these therapeutic interventions. Moreover, individuals with HIV who delay the initiation of antiretroviral therapy experience permanent loss of cutaneous memory T cells (48), thereby increasing their susceptibility to skin cancer development. An 80% elevated risk has been observed in comparison to HIV-negative individuals, with SCC being predominantly affected (49). Therefore, the global elevation in HIV prevalence (50) contributes to the increasing ASIR of skin cancers. Additionally, both human papillomavirus (HPV) (51) and diabetes (52) have been identified as contributory factors in skin cancer pathogenesis. The early proteins E6 and E7 of *β*-HPV have been shown to be capable of altering cellular responses to UV-induced stress and promoting the survival of DNA-damaged cells (51). In diabetic patients, elevated insulin and insulin-like growth factor (IGF) levels are associated with enhanced cellular proliferation and the upregulation of oncogenic epidermal growth factor receptors, subsequently inhibiting apoptosis and facilitating malignant transformation (53). Consequently, the increasing prevalence of both HPV (54) infection and diabetes mellitus (55) contributes to the rising incidence of skin cancer. Although previous investigations (56–58) have comprehensively summarized specific risk factors for skin cancers, the observed heterogeneity among these studies emphasizes the necessity for further research to elucidate risk factor profiles and develop targeted early prevention strategies. Furthermore, multiple factors have contributed to the improved detection rates of skin cancer: the global implementation of skin cancer screening programs (59–61), the promotion of self-skin examination (SSE) and total body skin examination (TBSE) protocols (62), and technological advancements including biosensors (63), computer-aided diagnosis systems (64), and teledermatoscopy platforms (65)—all enhancing diagnostic accuracy for skin malignancies. Notably, our temporal analysis of total skin cancer ASIR revealed a distinctive epidemiological pattern: a steady increase from 1990 to 2005, followed by a consistent decline from 2006 to 2019, and a significant resurgence from 2020 to 2021. Specifically, the ASIRs of BCC and SCC exhibited increasing trends from 2019 to 2021, whereas that of melanoma showed a declining trend, consistent with previous findings (66). The plateau in global skin cancer ASIR observed after the early 2000s can be attributed to the widespread implementation of public education and early detection programs, as supported by previous studies (67, 68). The mechanisms underlying the changes in skin cancer subtype ASIR between 2019 and 2021 encompass several key factors. Primarily, the COVID-19 pandemic has substantially influenced the differences in ASIRs for skin cancer subtypes between 2019 and 2021. Previous studies have demonstrated that severe and hospitalized COVID-19 cases are negatively correlated with melanoma risk (OR < 1) (69). Furthermore, the disruption of routine medical services and decreased screening compliance associated with COVID-19 have been detrimental to skin cancer diagnosis and detection. When combined with the suboptimal sensitivity of melanoma self-examination methods (70), melanoma diagnosis and detection have become increasingly challenging during the COVID-19 pandemic. In contrast, the distinctive clinical presentations of SCC and BCC (71, 72) have facilitated enhanced detection sensitivity, thereby mitigating the impact of healthcare access restrictions in 2020. Consequently, the ASIR of BCC and SCC has increased between 1990 and 2021, while melanoma ASIR has decreased.

From 1990 to 2021, the ASDR of total skin cancer demonstrated a declining trend, predominantly attributable to melanoma. This observation aligns with the findings of Sun et al., who documented a 16.8% reduction in melanoma ASDR during the corresponding period (2). The implementation of skin self-examination programs and public health education initiatives over recent decades (73–75) has facilitated early detection and intervention for skin cancers, resulting in a reduction in the overall disease burden. In contrast, BCC exhibited a significant increase in ASDR (EAPC = 1.64). This upward trend resulted from several contributing factors. The high recurrence rate (76), coupled with prolonged treatment and rehabilitation protocols associated with BCC, contributes to its elevated ASDR. Additionally, the escalating ASIR of BCC, in conjunction with demographic aging and the accumulation of untreated or delayed-treatment cases, has contributed substantially to the increasing disease burden. The extensive treatment duration and comprehensive rehabilitation regimen required for BCC emphasize the imperative for enhanced continuity of care. Consequently, healthcare systems should establish robust follow-up protocols to facilitate optimal post-treatment recovery and enable systematic surveillance. Such measures would expedite the identification of potential recurrences and mitigate the cumulative disease burden through prompt clinical intervention.

Given that DALYs can be decomposed into years lived with disability (YLD) and years of life lost (YLL), exploring the potential impact of both components on the observed findings is essential, although specific analysis of YLD and YLL changes in skin cancer was not conducted in this study. Since YLDs represent the health loss experienced by patients living with disease, and YLLs represent the years of life lost due to premature mortality caused by the disease, the increasing ASIRs of total skin cancer are associated with an elevated burden of YLDs. For skin cancer, the majority of cases are non-fatal (77, 78) when compared to other malignancies. Consequently, YLDs represent the principal component of DALYs, whereas YLLs contribute to a relatively smaller proportion. Both BCC and melanoma conform to this pattern. However, for SCC, both ASIRs and ASDRs demonstrated upward trends. Given that SCC exhibits greater local invasiveness and metastatic potential than BCC (79), this condition results in increased mortality, leading to elevated YLLs, while simultaneously indicating that substantial numbers of patients live with the disease long-term, thereby contributing to a greater YLD burden.

The epidemiological burden of skin cancer demonstrates marked geographical heterogeneity. Differential UVR exposure resulting from variations in stratospheric ozone depletion (80), urbanization processes (81), altitude (82), and latitude (83) contributes to this phenomenon. Consistent with previous epidemiological investigations (84), our analysis identified Australasia as the geographical region exhibiting the most substantial skin cancer burden, as evidenced by markedly elevated ASIR and ASDR. This phenomenon is predominantly attributable to the exceptionally high ambient UVR exposure levels prevalent on the Australian continent. Epidemiological investigation has estimated that approximately 63% of melanoma cases and virtually all keratinocyte carcinomas are attributable to cumulative solar radiation exposure (85). Notably, the demographic composition of this region is characterized by predominantly British (33.0%), Australian (29.9%), and Irish (9.5%) ancestries (86). These populations possess limited constitutive melanin levels and lack the physiological factors necessary for effective photoprotection against ultraviolet radiation (87), thereby exacerbating the skin cancer burden in this region. While regular sunscreen application has been demonstrated to prevent approximately 9.3% of NMSCs and 14% of melanoma cases (85), merely 15% of the population adheres to recommended sunscreen application protocols (88). Therefore, targeted regional public health initiatives should prioritize comprehensive sun protection education and awareness programs to enhance adherence to recommended photoprotective behaviors and optimize sunscreen utilization rates.

The burden of total skin cancer demonstrates a positive correlation with age, exhibiting a marked elevation after the age of 50, as evidenced by ASIRs and ASDRs. Analogous patterns were observed in melanoma, BCC, and SCC, which corroborates previous findings (1, 2, 89). Age constitutes a significant determinant in the pathogenesis of cutaneous malignancies. Previous investigations (90) have elucidated the underlying mechanisms, delineating age-related and ultraviolet (UV)-induced mutations as two distinct mutational pathways. These dual pathways mediate carcinogenesis not only in melanoma but also in BCC and SCC. Notably, UVB-induced mutations in keratinocytes during childhood and adolescence can accumulate temporally, contributing to carcinogenesis (91). This phenomenon is particularly significant considering that cumulative solar radiation exposure during these formative years accounts for the predominant proportion of an individual’s overall UV exposure throughout the lifespan (92). Although skin cancers are relatively infrequent in pediatric and adolescent populations, photoprotection remains imperative. Additionally, the ASIR of melanoma in males over 90 demonstrated a significant decline, consistent with previous epidemiological findings (4). Multiple age-associated biological processes contribute to this phenomenon, including stem cell senescence (93) and alterations in the microenvironmental milieu (94). Specifically, stem cell senescence is observed to promote tumor initiation through the Nupr1–lipocalin-2 pathway via the induction of functional iron deficiency (93). Age-induced senomorphics have been shown to suppress pro-tumorigenic signaling through the inhibition of SASP factor secretion, thereby alleviating chronic inflammation (95). Moreover, aging-associated stimuli, including DNA damage, telomere shortening, and oncogene activation, induce cellular senescence, resulting in stable growth arrest of these damaged cells (96, 97). Besides, the proportional distribution of skin cancer burden demonstrated age-dependent variations. With advancing age, the proportional contribution of melanoma to the total skin cancer burden diminished, whereas the proportion attributable to SCC increased. This phenomenon is attributed to differences in the etiological patterns of UVR exposure among skin cancer subtypes. Melanoma is primarily associated with intermittent and high-intensity solar radiation exposure (98, 99), which tends to decrease in geriatric populations due to health-related mobility and functional limitations (100). Furthermore, this phenomenon has been associated with a “plateau effect” in screening and self-examination frequencies. Although routine skin examinations remain capable of detecting early-stage melanoma, the marginal benefits of such screening are diminished in older adult populations (101). Consequently, the proportional contribution of melanoma to the overall disease burden exhibits a progressive decline with advancing age. Conversely, SCC is associated with cumulative and chronic low-dose ultraviolet radiation exposure (98, 99), which accumulates with advancing age, thereby resulting in an increased burden of SCC among older adult populations. These findings underscore the significance of ultraviolet radiation protection across all demographic age cohorts, while simultaneously emphasizing the necessity for age-stratified prevention strategies calibrated to address distinct patterns of ultraviolet exposure and cutaneous malignancy risk profiles.

Sex-specific analysis revealed significantly elevated ASIR and ASDR in males compared to females for total skin cancer, with this disparity progressively amplifying with advancing age. This pattern was consistent across all three histological subtypes of skin cancer, highlighting sex-based biological differences as critical determinants in skin cancer epidemiology. This observed disparity results from a multitude of factors. Behavioral differences are considered the primary drivers of sex- disparities in skin cancer (102); women demonstrate greater photoprotection awareness and protective behaviors than men (103, 104), and exhibit higher likelihood of performing skin self-examinations (105). Furthermore, skin biology is profoundly influenced by sex hormones, including estrogens, androgens, and progesterone (106–108). Among these hormones, androgens have been demonstrated to exert immunosuppressive effects, whereas estrogens are generally considered immunoenhancing agents (109). Thus, sex-specific differences in behavior and hormonal milieu are hypothesized to underlie sex-related disparities in skin cancer incidence.

The skin cancer burden demonstrates a significant association with SDI. A turning point was observed at an SDI of approximately 0.7 to 0.8, which represents the interactive effect between “health transition” and “public health saturation.” During the low to moderate SDI stage (0–0.8), both ASIR and ASDR demonstrated a general upward trend. This finding is consistent with previous epidemiological studies (110, 111), which have reported a positive correlation between cancer incidence and socioeconomic development. Multiple factors contribute to this epidemiological pattern, including demographic aging, elevated air pollution levels (112), increased light pollution (113), and enhanced accessibility to skin cancer screening protocols that facilitate early detection. Furthermore, economic development and improved living standards result in lifestyle changes, which lead to increased ultraviolet exposure through outdoor recreation, sunbathing, and artificial tanning, thereby contributing to the rise in skin cancer incidence. Additionally, the increase in ASIR at this stage potentially contributes to the observed trend in ASDR through its proportional impact on YLD calculations within the DALY framework. At this stage, health systems and public awareness of skin cancer have not been adequately developed, while sun protection awareness and early screening systems remain imperfect, resulting in continued growth of ASIRs and ASDRs. During the moderate-to-high and high SDI stages (≥0.8), ASDR began to decline, while ASIR continued to increase. At this stage, public health interventions achieve “critical density,” which is characterized by elevated health literacy and heightened disease awareness (114). In conjunction with advanced diagnostic modalities and therapeutic interventions (115, 116), these factors have precipitated the observed decline in ASDR. At this stage, prevention benefits begin to exceed risk growth rates, thereby establishing an inflection point. In conclusion, the inflection point—at an SDI of approximately 0.7 to 0.8— represents an equilibrium between lifestyle risks associated with economic and social development and the implementation of mature public health prevention and control systems. Prior to this inflection point, risk accumulation predominates; subsequently, large-scale prevention effects become evident. Therefore, low- and middle-SDI regions need to implement effective measures to address their continuously rising incidence and DALYs. However, the increasing cancer burden in these regions presents unique challenges to their already fragile healthcare and economic infrastructure. While previous research has demonstrated that early detection represents an effective strategy for controlling skin cancer (117), prioritizing early detection cannot be considered effective in low- and middle-income countries, where limited downstream resources will be overwhelmed by the inevitable increase in diagnostic volume (118). Therefore, optimal utilization of currently limited resources must be achieved. To achieve this objective, based on recommendations proposed by Timothy et al. (119), the following enhancements are required in low- and middle-SDI regions: (A) enhancement of skin cancer health services research and policy planning capacity; (B) establishment of high-quality health data sources; (C) strengthening of skin cancer-related economic evaluations; (D) exploration and localization of high-quality skin cancer control models; and (E) optimization of early detection strategies to prevent downstream resource overload.

Skin cancer burden exhibits significant health disparities, predominantly attributable to variations in socioeconomic status (SES). Substantial epidemiological evidence demonstrates that SES influences oncological outcomes through multiple mechanistic pathways (120–122), including health literacy (123), healthcare accessibility (124), and psychosocial resource allocation (125), which collectively modulate disease incidence, prognostic indicators, screening adherence, and therapeutic accessibility (126). Our analysis reveals narrowing disparities across socioeconomic strata, evidenced by decreasing SII metrics. Implemented public health policies, targeted interventions, and equitable social health services effectively ameliorated health outcomes among lower-SES populations, thereby diminishing the healthcare disparities between disadvantaged and advantaged socioeconomic cohorts. However, the skin cancer burden remains concentrated among disadvantaged populations. While high-SES cohorts exhibit higher melanoma and BCC incidence rates (127–129), lower-SES populations experience inferior clinical outcomes attributable to the limitations in health literacy, healthcare access, and prevention implementation. Consequently, healthcare policies should be strategically calibrated to address distinctive characteristics of diverse socioeconomic strata, prioritizing healthcare accessibility and quality to reduce disparities in skin cancer outcomes.

Based on predictive modeling, total skin cancer ASIR is projected to increase while ASDR is expected to continue declining through 2040, confirming previous findings (3, 4). This phenomenon results from a confluence of factors, including the escalation of high-risk behaviors concurrent with societal development, alongside advances in prevention, diagnosis, and treatment methodologies. Despite appreciable progress achieved through current healthcare management protocols and the projected continued decline in skin cancer ASDR, sustained vigilance toward the skin cancer burden remains imperative, particularly in the context of progressive population aging. While the ASDRs for melanoma and SCC were expected to decrease, BCC ASDR demonstrates an upward trend, indicating policymakers should allocate more resources toward BCC management.

Utilizing the latest GBD 2021 database, this investigation quantifies the burden of total skin cancer, comparatively analyzes its subtype-specific burden distribution, and generates empirical evidence crucial for evidence-based policy formulation. However, several methodological limitations exist. First, the quality and availability of data vary significantly across countries and regions, particularly in resource-limited settings, finally leading to underreporting or estimation bias. Second, this analysis relies on statistical models with inherent assumptions, which introduce uncertainty, particularly when data are sparse or heterogeneous. Third, regional variations in disease definitions, diagnostic criteria, and reporting practices may compromise the comparability of estimates among countries. Fourth, due to data limitations, our analysis was restricted to three major skin cancer subtypes, with other subtypes being excluded from the study.

In conclusion, skin cancer constitutes a substantial and emergent global public health challenge. From 1990 to 2021, the skin cancer ASIR increased while ASDR decreased. Substantial epidemiological disparities exist across countries, regions, age groups, and sex. Implementation of targeted healthcare interventions stratified by regional epidemiology, sex-specific risk profiles, and age-dependent vulnerability factors are imperative for optimizing therapeutic outcomes and mitigating the overall disease burden.

## Data Availability

Publicly available datasets were analyzed in this study. This data can be found at: http://www.healthdata.org/ and https://vizhub.healthdata.org/gbd-results/.

## Footnotes

^1^ http://ghdx.healthdata.org/gbd-results-tool

## References

[1] Zhang W Zeng W Jiang A He Z Shen X Dong X. Global, regional and National Incidence, mortality and disability-adjusted life-years of skin cancers and trend analysis from 1990 to 2019: an analysis of the global burden of disease study 2019. Cancer Med. (2021) 10:4905–22. doi: 10.1002/cam4.4046, PMID: 34105887 PMC8290243

[2] Sun Y Shen Y Liu Q Zhang H Jia L Chai Y. Global trends in melanoma burden: a comprehensive analysis from the global burden of disease study, 1990-2021. J Am Acad Dermatol. (2024) 92:100–7. doi: 10.1016/j.jaad.2024.09.035 39343306

[3] Hu W Fang L Ni R Zhang H Pan G. Changing trends in the disease burden of non-melanoma skin cancer globally from 1990 to 2019 and its predicted level in 25 years. BMC Cancer. (2022) 22:836. doi: 10.1186/s12885-022-09940-3, PMID: 35907848 PMC9339183

[4] Urban K Mehrmal S Uppal P Giesey RL Delost GR. The global burden of skin Cancer: a longitudinal analysis from the global burden of disease study, 1990-2017. JAAD Int. (2021) 2:98–108. doi: 10.1016/j.jdin.2020.10.013, PMID: 34409358 PMC8362234

[5] Venables ZC Nijsten T Wong KF Autier P Broggio J Deas A. Epidemiology of basal and cutaneous squamous cell carcinoma in the U.K. 2013-15: a cohort study. Br J Dermatol. (2019) 181:474–82. doi: 10.1111/bjd.17873, PMID: 30864158 PMC7379277

[6] Keohane SG Proby CM Newlands C Motley RJ Nasr I Mohd Mustapa MF. The new 8th edition of Tnm staging and its implications for skin Cancer: a review by the British Association of Dermatologists and the Royal College of pathologists, U.K. Br J Dermatol. (2018) 179:824–8. doi: 10.1111/bjd.16892, PMID: 29923189

[7] Rubin AI Chen EH Ratner D. Basal-Cell Carcinoma. N Engl J Med. (2005) 353:2262–9. doi: 10.1056/NEJMra044151, PMID: 16306523

[8] Roewert-Huber J Lange-Asschenfeldt B Stockfleth E Kerl H. Epidemiology and aetiology of basal cell carcinoma. Br J Dermatol. (2007) 157:47–51. doi: 10.1111/j.1365-2133.2007.08273.x 18067632

[9] Asgari MM Moffet HH Ray GT Quesenberry CP. Trends in basal cell carcinoma incidence and identification of high-risk subgroups, 1998-2012. JAMA Dermatol. (2015) 151:976–81. doi: 10.1001/jamadermatol.2015.1188, PMID: 26039887

[10] Lai V Cranwell W Sinclair R. Epidemiology of skin Cancer in the mature patient. Clin Dermatol. (2018) 36:167–76. doi: 10.1016/j.clindermatol.2017.10.008, PMID: 29566921

[11] Huang J Chan SC Ko S Lok V Zhang L Lin X. Global incidence, mortality, risk factors and trends of melanoma: a systematic analysis of registries. Am J Clin Dermatol. (2023) 24:965–75. doi: 10.1007/s40257-023-00795-3, PMID: 37296344

[12] Kao SZ Ekwueme DU Holman DM Rim SH Thomas CC Saraiya M. Economic burden of skin Cancer treatment in the USA: an analysis of the medical expenditure panel survey data, 2012-2018. Cancer Causes Control. (2023) 34:205–12. doi: 10.1007/s10552-022-01644-0, PMID: 36449145 PMC11001479

[13] Collins LG Minto C Ledger M Blane S Hendrie D. Cost-effectiveness analysis and return on Investment of Sunsmart Western Australia to prevent skin Cancer. Health Promot Int. (2024) 39:daae091. doi: 10.1093/heapro/daae091, PMID: 39110010 PMC11333957

[14] Gordon LG Rowell D. Health system costs of skin Cancer and cost-effectiveness of skin Cancer prevention and screening: a systematic review. Eur J Cancer Prev. (2015) 24:141–9. doi: 10.1097/cej.0000000000000056, PMID: 25089375

[15] Gordon LG Leung W Johns R McNoe B Lindsay D Merollini KMD. Estimated healthcare costs of melanoma and keratinocyte skin cancers in Australia and Aotearoa New Zealand in 2021. Int J Environ Res Public Health. (2022) 19:3178. doi: 10.3390/ijerph19063178, PMID: 35328865 PMC8948716

[16] Bray F Laversanne M Sung H Ferlay J Siegel RL Soerjomataram I. Global Cancer statistics 2022: Globocan estimates of incidence and mortality worldwide for 36 cancers in 185 countries. CA Cancer J Clin. (2024) 74:229–63. doi: 10.3322/caac.21834, PMID: 38572751

[17] Siegel RL Giaquinto AN Jemal A. Cancer statistics, 2024. CA Cancer J Clin. (2024) 74:12–49. doi: 10.3322/caac.21820, PMID: 38230766

[18] Roky AH Islam MM Ahasan AMF Mostaq MS Mahmud MZ Amin MN. Overview of skin cancer types and prevalence rates across continents. Cancer Pathog Ther. (2024) 3:89–100. doi: 10.1016/j.cpt.2024.08.002 40182119 PMC11963195

[19] Ferrari A Santomauro D Aali A Habtegiorgis Y Cristiana A Abbastabar H. Global incidence, prevalence, years lived with disability (Ylds), disability-adjusted life-years (Dalys), and healthy life expectancy (Hale) for 371 diseases and injuries in 204 countries and territories and 811 subnational locations, 1990-2021: a systematic analysis for the global burden of disease study 2021. Lancet. (2024) 403:2133–61. doi: 10.1016/S0140-6736(24)00757-8 38642570 PMC11122111

[20] GBD 2021 Demographics Collaborators. Global age-sex-specific mortality, life expectancy, and population estimates in 204 countries and territories and 811 subnational locations, 1950-2021, and the impact of the COVID-19 pandemic: a comprehensive demographic analysis for the global burden of disease study 2021. Lancet. (2024) 403:1989–2056. doi: 10.1016/s0140-6736(24)00476-8, PMID: 38484753 PMC11126395

[21] Fu L Tian T Wang B Lu Z Bian J Zhang W. Global, regional, and National Burden of Hiv and other sexually transmitted infections in older adults aged 60-89 years from 1990 to 2019: results from the global burden of disease study 2019. Lancet Healthy Longev. (2024) 5:e17–30. doi: 10.1016/s2666-7568(23)00214-3, PMID: 38183996

[22] Deng J Zhang H Wang Y Liu Q Du M Yan W. Global, regional, and National Burden of dengue infection in children and adolescents: an analysis of the global burden of disease study 2021. EClinicalMedicine. (2024) 78:102943. doi: 10.1016/j.eclinm.2024.102943, PMID: 39640938 PMC11617407

[23] Hankey BF Ries LA Kosary CL Feuer EJ Merrill RM Clegg LX. Partitioning linear trends in age-adjusted rates. Cancer Causes Control. (2000) 11:31–5. doi: 10.1023/a:1008953201688 10680727

[24] Jani CT Kareff SA Morgenstern-Kaplan D Salazar AS Hanbury G Salciccioli JD. Evolving trends in lung Cancer risk factors in the ten Most populous countries: an analysis of data from the 2019 global burden of disease study. EClinicalMedicine. (2025) 79:103033. doi: 10.1016/j.eclinm.2024.103033, PMID: 39968204 PMC11833020

[25] Yélamos O Geller S Tokez S. Skin Cancer special issue in skin health and disease. Skin Health Dis. (2023) 3:e224. doi: 10.1002/ski2.224, PMID: 37013119 PMC10066748

[26] Naeser Y Mikiver R Ingvar C Lambe M Ullenhag GJ. Survival in patients diagnosed with melanoma in situ compared to the general population. A Swedish population-based matched cohort study. EClinicalMedicine. (2023) 65:102284. doi: 10.1016/j.eclinm.2023.102284 38106551 PMC10725068

[27] Pfeifer GP. Mechanisms of UV-induced mutations and skin cancer. Genome Instab Dis. (2020) 1:99–113. doi: 10.1007/s42764-020-00009-8, PMID: 34589668 PMC8477449

[28] Yu ZW Zheng M Fan HY Liang XH Tang YL. Ultraviolet (UV) radiation: a double-edged sword in cancer development and therapy. Mol Biomed. (2024) 5:49. doi: 10.1186/s43556-024-00209-8, PMID: 39417901 PMC11486887

[29] Fukunaga A Khaskhely NM Ma Y Sreevidya CS Taguchi K Nishigori C. Langerhans cells serve as immunoregulatory cells by activating NKT cells. J Immunol. (2010) 185:4633–40. doi: 10.4049/jimmunol.1000246, PMID: 20844203 PMC2950871

[30] Goukassian D Gad F Yaar M Eller MS Nehal US Gilchrest BA. Mechanisms and implications of the age-associated decrease in DNA repair capacity. FASEB J. (2000) 14:1325–34. doi: 10.1096/fj.14.10.1325, PMID: 10877825

[31] Xu YP Qi RQ Chen W Shi Y Cui ZZ Gao XH. Aging affects epidermal Langerhans cell development and function and alters their miRNA gene expression profile. Aging. (2012) 4:742–54. doi: 10.18632/aging.100501, PMID: 23178507 PMC3560442

[32] Farsam V Basu A Gatzka M Treiber N Schneider LA Mulaw MA. Senescent fibroblast-derived Chemerin promotes squamous cell carcinoma migration. Oncotarget. (2016) 7:83554–69. doi: 10.18632/oncotarget.13446, PMID: 27907906 PMC5347788

[33] Zheng Q Capell BC Parekh V O’Day C Atillasoy C Bashir HM. Whole-exome and transcriptome analysis of Uv-exposed epidermis and carcinoma in situ reveals early drivers of carcinogenesis. J Invest Dermatol. (2021) 141:295–307.e13. doi: 10.1016/j.jid.2020.05.116, PMID: 32649944 PMC7790860

[34] Climstein M Doyle B Stapelberg M Rosic N Hertess I Furness J. Point prevalence of non-melanoma and melanoma skin cancers in Australian surfers and swimmers in Southeast Queensland and northern New South Wales. PeerJ. (2022) 10:e13243. doi: 10.7717/peerj.13243, PMID: 35505675 PMC9057286

[35] Wang Y Lipner SR. Retrospective study of ultraviolet indices and incidence of melanoma in the United States. Int J Dermatol. (2022) 61:e256–7. doi: 10.1111/ijd.15773, PMID: 34241892

[36] Beard JR Officer A de Carvalho IA Sadana R Pot AM Michel JP. The world report on ageing and health: a policy framework for healthy ageing. Lancet. (2016) 387:2145–54. doi: 10.1016/s0140-6736(15)00516-4, PMID: 26520231 PMC4848186

[37] O’Donovan P Perrett CM Zhang X Montaner B Xu YZ Harwood CA. Azathioprine and UVA light generate mutagenic oxidative DNA damage. Science. (2005) 309:1871–4. doi: 10.1126/science.1114233, PMID: 16166520 PMC2426755

[38] Yarosh DB Pena AV Nay SL Canning MT Brown DA. Calcineurin inhibitors decrease DNA repair and apoptosis in human keratinocytes following ultraviolet B irradiation. J Invest Dermatol. (2005) 125:1020–5. doi: 10.1111/j.0022-202X.2005.23858.x, PMID: 16297204

[39] Akdag D Rasmussen A Nielsen SD Møller DL Togsverd-Bo K Wenande E. Early results of a screening program for skin Cancer in liver transplant recipients: a cohort study. Cancers. (2024) 16:1224. doi: 10.3390/cancers16061224, PMID: 38539557 PMC10969135

[40] Mäkilä T Keinonen A Mäkisalo H Koskenmies S. Skin cancers and their precursors in Finnish liver transplantation recipients: a follow-up study. JEADV Clin Pract. (2024) 3:1027–34. doi: 10.1002/jvc2.386

[41] Hegedus F Mathew LM Schwartz RA. Radiation dermatitis: an overview. Int J Dermatol. (2017) 56:909–14. doi: 10.1111/ijd.13371, PMID: 27496623

[42] Perkins JL Liu Y Mitby PA Neglia JP Hammond S Stovall M. Nonmelanoma skin Cancer in survivors of childhood and adolescent Cancer: a report from the childhood Cancer survivor study. J Clin Oncol. (2005) 23:3733–41. doi: 10.1200/jco.2005.06.237, PMID: 15923570

[43] Cui J Wang T-J Zhang Y-X She L-Z Zhao Y-C. Molecular biological mechanisms of radiotherapy-induced skin injury occurrence and treatment. Biomed Pharmacother. (2024) 180:117470. doi: 10.1016/j.biopha.2024.117470, PMID: 39321513

[44] Azoulay L St-Jean A Dahl M Quail J Aibibula W Brophy JM. Hydrochlorothiazide use and risk of keratinocyte carcinoma and melanoma: a multisite population-based cohort study. J Am Acad Dermatol. (2023) 89:243–53. doi: 10.1016/j.jaad.2023.04.035, PMID: 37105517

[45] Raone B Patrizi A Gurioli C Gazzola A Ravaioli GM. Cutaneous carcinogenic risk evaluation in 375 patients treated with narrowband-Uvb phototherapy: a 15-year experience from our institute. Photodermatol Photo. (2018) 34:302–6. doi: 10.1111/phpp.12382, PMID: 29533483

[46] Osmancevic A Gillstedt M Wennberg AM Larkö O. The risk of skin Cancer in psoriasis patients treated with Uvb therapy. Acta Derm Venereol. (2014) 94:425–30. doi: 10.2340/00015555-1753, PMID: 24322826

[47] Maiorino A De Simone C Perino F Caldarola G Peris K. Melanoma and non-melanoma skin Cancer in psoriatic patients treated with high-dose phototherapy. J Dermatolog Treat. (2016) 27:443–7. doi: 10.3109/09546634.2015.1133882, PMID: 26822468

[48] Saluzzo S Pandey RV Gail LM Dingelmaier-Hovorka R Kleissl L Shaw L. Delayed antiretroviral therapy in HIV-infected individuals leads to irreversible depletion of skin- and mucosa-resident memory T cells. Immunity. (2021) 54:2842–58. doi: 10.1016/j.immuni.2021.10.021 34813775

[49] Martinez DAS Lupi O D’Ácri AM. The association between skin cancer and HIV infection. Indian J Dermatol Venereol Leprol. (2023) 89:725–8. doi: 10.25259/IJDVL_902_2021 37067140

[50] GBD 2021 HIV Collaborators. Global, regional, and national burden of HIV/AIDS, 1990-2021, and forecasts to 2050, for 204 countries and territories: the global burden of disease study 2021. Lancet HIV. (2024) 11:e807–22. doi: 10.1016/s2352-3018(24)00212-1, PMID: 39608393 PMC11612058

[51] Lambert PF Münger K Rösl F Hasche D Tommasino M. Beta human papillomaviruses and skin Cancer. Nature. (2020) 588:E20–1. doi: 10.1038/s41586-020-3023-0 33328661

[52] Chang W-C Hsieh T-C Hsu W-L Chang F-L Tsai H-R He M-S. Diabetes and further risk of Cancer: a Nationwide population-based study. BMC Med. (2024) 22:214. doi: 10.1186/s12916-024-03430-y, PMID: 38807177 PMC11134680

[53] Brownlee M. Biochemistry and molecular cell biology of diabetic complications. Nature. (2001) 414:813–20. doi: 10.1038/414813a., PMID: 11742414

[54] Yang M Du J Lu H Xiang F Mei H Xiao H. Global trends and age-specific incidence and mortality of cervical cancer from 1990 to 2019: an international comparative study based on the global burden of disease. BMJ Open. (2022) 12:e055470. doi: 10.1136/bmjopen-2021-055470, PMID: 35868828 PMC9316042

[55] GBD 2021 Diabetes Collaborators. Global, regional, and national burden of diabetes from 1990 to 2021, with projections of prevalence to 2050: a systematic analysis for the global burden of disease study 2021. Lancet. (2025) 405:202. doi: 10.1016/S0140-6736(25)00053-4 PMC10364581 37356446

[56] Saladi RN Persaud AN. The causes of skin cancer: a comprehensive review. Drugs Today (Barc). (2005) 41:37–53. doi: 10.1358/dot.2005.41.1.875777, PMID: 15753968

[57] Conforti C Zalaudek I. Epidemiology and risk factors of melanoma: a review. Dermatol Pract Concept. (2021) 11:e2021161S. doi: 10.5826/dpc.11S1a161S, PMID: 34447610 PMC8366310

[58] Artosi F Costanza G Di Prete M Garofalo V Lozzi F Dika E. Epidemiological and clinical analysis of exposure-related factors in non-melanoma skin Cancer: a retrospective cohort study. Environ Res. (2024) 247:118117. doi: 10.1016/j.envres.2024.118117, PMID: 38218521

[59] Smith B Smith JE Demanelis K Ferris LK. Changes in skin Cancer screening rates in the United States from 2005 to 2015. J Am Acad Dermatol. (2023) 89:142–5. doi: 10.1016/j.jaad.2023.02.011, PMID: 36804574 PMC11225604

[60] Katalinic A Eisemann N Waldmann A. Skin Cancer screening in Germany. Documenting melanoma incidence and mortality from 2008 to 2013. Dtsch Arztebl Int. (2015) 112:629–34. doi: 10.3238/arztebl.2015.0629, PMID: 26429634 PMC4593927

[61] Del Marmol V. Prevention and screening of melanoma in Europe: 20 years of the Euromelanoma campaign. J Eur Acad Dermatol Venereol. (2022) 36:5–11. doi: 10.1111/jdv.18195, PMID: 35738812

[62] Wang Y Derouin A Turner B Xu H. Improving skin cancer knowledge and screening among older Chinese Americans. J Nurse Practition. (2024) 20:105208. doi: 10.1016/j.nurpra.2024.105208

[63] Chatzilakou E Hu Y Jiang N Yetisen AKJB. Biosensors for melanoma skin Cancer diagnostics. Biosens Bioelectron. (2024) 250:116045. doi: 10.1016/j.bios.2024.116045 38301546

[64] Kumar Lilhore U Simaiya S Sharma YK Kaswan KS Rao KB Rao VM. A precise model for skin Cancer diagnosis using hybrid U-net and improved Mobilenet-V3 with Hyperparameters optimization. Sci Rep. (2024) 14:4299. doi: 10.1038/s41598-024-54212-8, PMID: 38383520 PMC10881962

[65] Uppal SK Beer J Hadeler E Gitlow H Nouri K. The clinical utility of Teledermoscopy in the era of telemedicine. Dermatol Ther. (2021) 34:e14766. doi: 10.1111/dth.14766, PMID: 33421232

[66] Barruscotti S Giorgini C Brazzelli V Vassallo C Michelerio A Klersy C. A significant reduction in the diagnosis of melanoma during the Covid-19 lockdown in a third-level center in the northern Italy. Dermatol Ther. (2020) 33:e14074. doi: 10.1111/dth.14074, PMID: 32713046

[67] Eisemann N Waldmann A Geller AC Weinstock MA Volkmer B Greinert R. Non-melanoma skin cancer incidence and impact of skin cancer screening on incidence. J Invest Dermatol. (2014) 134:43–50. doi: 10.1038/jid.2013.304, PMID: 23877569

[68] MacKie RM Bray CA Leman JA. Effect of public education aimed at early diagnosis of malignant melanoma: cohort comparison study. BMJ. (2003) 326:367. doi: 10.1136/bmj.326.7385.367, PMID: 12586670 PMC148894

[69] Li J Bai H Qiao H Du C Yao P Zhang Y. Causal effects of COVID-19 on cancer risk: a Mendelian randomization study. J Med Virol. (2023) 95:e28722. doi: 10.1002/jmv.28722, PMID: 37185860

[70] Climstein M Hudson J Stapelberg M Miller IJ Rosic N Coxon P. Patients poorly recognize lesions of concern that are malignant melanomas: is self-screening the correct advice? PeerJ. (2024) 12:e17674. doi: 10.7717/peerj.17674, PMID: 38974412 PMC11227272

[71] Naik PP Desai MB. Basal cell carcinoma: a narrative review on contemporary diagnosis and management. Oncol Ther. (2022) 10:317–35. doi: 10.1007/s40487-022-00201-8, PMID: 35729457 PMC9681969

[72] Wysong A. Squamous-cell carcinoma of the skin. N Engl J Med. (2023) 388:2262–73. doi: 10.1056/NEJMra2206348, PMID: 37314707

[73] Robinson JK Wahood S Ly S Kirk J Yoon J Sterritt J. Melanoma detection by skin self-examination targeting at-risk women: a randomized controlled trial with telemedicine support for concerning moles. Prev Med Rep. (2021) 24:101532. doi: 10.1016/j.pmedr.2021.101532, PMID: 34976609 PMC8683880

[74] Greene A Iloabuchi VC Stoos E Butterfield RJ Zhang N Mangold AR. Increase in melanoma knowledge in Latino patients after a targeted digital educational program. JAAD Int. (2024) 14:61–3. doi: 10.1016/j.jdin.2023.11.002, PMID: 38274397 PMC10809116

[75] Ferris LK. The value of behavioral counseling for skin Cancer prevention: actions we can take now and guidance for the future. JAMA Oncol. (2018) 4:630–2. doi: 10.1001/jamaoncol.2018.0469, PMID: 29558534

[76] Tomás-Velázquez A Sanmartin-Jiménez O Garcés JR Rodríguez-Prieto MA Ruiz-Salas V De Eusebio-Murillo E. Risk factors and rate of recurrence after Mohs surgery in basal cell and squamous cell carcinomas: a Nationwide prospective cohort (Regesmohs, Spanish registry of Mohs surgery). Acta Derm Venereol. (2021) 101:adv00602. doi: 10.2340/actadv.v101.544, PMID: 34694418 PMC9455311

[77] Barton V Armeson K Hampras S Ferris LK Visvanathan K Rollison D. Nonmelanoma skin cancer and risk of all-cause and cancer-related mortality: a systematic review. Arch Dermatol Res. (2017) 309:243–51. doi: 10.1007/s00403-017-1724-5, PMID: 28285366 PMC5396844

[78] Linos E Parvataneni R Stuart SE Boscardin WJ Landefeld CS Chren MM. Treatment of nonfatal conditions at the end of life: nonmelanoma skin cancer. JAMA Intern Med. (2013) 173:1006–12. doi: 10.1001/jamainternmed.2013.639, PMID: 23699934 PMC3726204

[79] Ashford BG Clark J Gupta R Iyer NG Yu B Ranson M. Reviewing the genetic alterations in high-risk cutaneous squamous cell carcinoma: a search for prognostic markers and therapeutic targets. Head Neck. (2017) 39:1462–9. doi: 10.1002/hed.24765, PMID: 28370784

[80] Sánchez CF. The relationship between the ozone layer and skin Cancer. Rev Med Chile. (2006) 134:1185–90. doi: 10.4067/s0034-98872006000900015, PMID: 17171222

[81] Grau-Pérez M Borrego L Carretero G Almeida P Cano J. Assessing the effect of environmental and socio-economic factors on skin melanoma incidence: an island-wide spatial study in gran Canaria (Spain), 2007-2018. Cancer Causes Control. (2022) 33:1261–72. doi: 10.1007/s10552-022-01614-6, PMID: 35925499 PMC9427872

[82] Del Fiore P Russo I Dal Monico A Tartaglia J Ferrazzi B Mazza M. Altitude effect on cutaneous melanoma epidemiology in the Veneto region (northern Italy): a pilot study. Life. (2022) 12:745. doi: 10.3390/life12050745, PMID: 35629411 PMC9146073

[83] Rivas M Rojas E Calaf GM Barberán M Liberman C De Paula Correa M. Association between non-melanoma and melanoma skin cancer rates, vitamin D and latitude. Oncol Lett. (2017) 13:3787–92. doi: 10.3892/ol.2017.5898, PMID: 28521479 PMC5431270

[84] Wang M Gao X Zhang L Guo L Pan X. Recent global patterns in skin cancer incidence, mortality, and prevalence. Chin Med J. (2025) 138:185–92. doi: 10.1097/CM9.0000000000003416 39682020 PMC11745855

[85] Olsen CM Wilson LF Green AC Bain CJ Fritschi L Neale RE. Cancers in Australia attributable to exposure to solar ultraviolet radiation and prevented by regular sunscreen use. Aust N Z J Public Health. (2015) 39:471–6. doi: 10.1111/1753-6405.12470, PMID: 26437734 PMC4606762

[86] Australian Bureau of Statistics. Cultural diversity: Census. Canberra: ABS. Available online at: https://www.abs.gov.au/statistics/people/people-and-communities/cultural-diversity-census/latest-release. (Accessed February 17, 2021).

[87] Brenner M Hearing VJ. The protective role of melanin against Uv damage in human skin. Photochem Photobiol. (2008) 84:539–49. doi: 10.1111/j.1751-1097.2007.00226.x, PMID: 18435612 PMC2671032

[88] Lee A Garbutcheon-Singh KB Dixit S Brown P Smith SD. The influence of age and gender in knowledge, behaviors and attitudes towards Sun protection: a cross-sectional survey of Australian outpatient clinic attendees. Am J Clin Dermatol. (2015) 16:47–54. doi: 10.1007/s40257-014-0106-4, PMID: 25516331

[89] Ciążyńska M Kamińska-Winciorek G Lange D Lewandowski B Reich A Sławińska M. The incidence and clinical analysis of non-melanoma skin Cancer. Sci Rep. (2021) 11:4337. doi: 10.1038/s41598-021-83502-8, PMID: 33619293 PMC7900109

[90] Lotz M Budden T Furney SJ Virós A. Molecular subtype, biological sex and age shape melanoma tumour evolution. Br J Dermatol. (2021) 184:328–37. doi: 10.1111/bjd.19128, PMID: 32282938 PMC7613609

[91] Lewis DA Travers JB Spandau DF. A new paradigm for the role of aging in the development of skin Cancer. J Invest Dermatol. (2009) 129:787–91. doi: 10.1038/jid.2008.293, PMID: 18818672 PMC3989529

[92] Laughlin-Richard N. Sun exposure and skin Cancer prevention in children and adolescents. J Sch Nurs. (2000) 16:20–6. doi: 10.1177/105984050001600204, PMID: 11151538

[93] Cañeque T Rodriguez R. Ageing of stem cells reduces their capacity to form tumours. Nature. (2024) 637:36–7. doi: 10.1038/d41586-024-03721-7, PMID: 39633118

[94] Ledford H. Why Cancer risk declines sharply in old age. Nature. (2024) 631:261–2. doi: 10.1038/d41586-024-02107-z, PMID: 38961208

[95] Qi X Jiang L Cao J. Senotherapies: a novel strategy for synergistic anti-tumor therapy. Drug Discov Today. (2022) 27:103365. doi: 10.1016/j.drudis.2022.103365, PMID: 36115631

[96] Lucas V Cavadas C Aveleira CA. Cellular senescence: from mechanisms to current biomarkers and Senotherapies. Pharmacol Rev. (2023) 75:675–713. doi: 10.1124/pharmrev.122.000622, PMID: 36732079

[97] Chiu FY Kvadas RM Mheidly Z Shahbandi A Jackson JG. Could senescence phenotypes strike the balance to promote tumor dormancy? Cancer Metastasis Rev. (2023) 42:143–60. doi: 10.1007/s10555-023-10089-z, PMID: 36735097 PMC10710690

[98] Walter SD King WD Marrett LD. Association of cutaneous malignant melanoma with intermittent exposure to ultraviolet radiation: results of a case-control study in Ontario, Canada. Int J Epidemiol. (1999) 28:418–27. doi: 10.1093/ije/28.3.418, PMID: 10405843

[99] MacKie RM. Long-term health risk to the skin of ultraviolet radiation. Prog Biophys Mol Biol. (2006) 92:92–6. doi: 10.1016/j.pbiomolbio.2006.02.008, PMID: 16616325

[100] Lin TC Liao YC. The impact of sunlight exposure on the health of older adults. Hu Li Za Zhi. (2016) 63:116–22. doi: 10.6224/jn.63.4.116, PMID: 27492302

[101] Ciocan D Barbe C Aubin F Granel-Brocard F Lipsker D Velten M. Distinctive features of melanoma and its management in elderly patients: a population-based study in France. JAMA Dermatol. (2013) 149:1150–7. doi: 10.1001/jamadermatol.2013.706 23945633

[102] Bellenghi M Puglisi R Pontecorvi G De Feo A Carè A Mattia G. Sex and gender disparities in melanoma. Cancers. (2020) 12:1819. doi: 10.3390/cancers12071819, PMID: 32645881 PMC7408637

[103] Patel AR Zaslow TL Wren TAL Daoud AK Campbell K Nagle K. A characterization of Sun protection attitudes and behaviors among children and adolescents in the United States. Prev Med Rep. (2019) 16:100988. doi: 10.1016/j.pmedr.2019.100988 31660287 PMC6807366

[104] Falk M Anderson CD. Influence of age, gender, educational level and self-estimation of skin type on Sun exposure habits and readiness to increase Sun protection. Cancer Epidemiol. (2013) 37:127–32. doi: 10.1016/j.canep.2012.12.006, PMID: 23295002

[105] Paddock LE Lu SE Bandera EV Rhoads GG Fine J Paine S. Skin self-examination and long-term melanoma survival. Melanoma Res. (2016) 26:401–8. doi: 10.1097/CMR.0000000000000255, PMID: 26990272

[106] Hall G Phillips TJ. Estrogen and skin: the effects of estrogen, menopause, and hormone replacement therapy on the skin. J Am Acad Dermatol. (2005) 53:555–68. doi: 10.1016/j.jaad.2004.08.039, PMID: 16198774

[107] Holzer G Riegler E Hönigsmann H Farokhnia S Schmidt JB. Effects and side-effects of 2% progesterone cream on the skin of peri- and postmenopausal women: results from a double-blind, vehicle-controlled, randomized study. Br J Dermatol. (2005) 153:626–34. doi: 10.1111/j.1365-2133.2005.06685.x, PMID: 16120154

[108] Wierckx K Van de Peer F Verhaeghe E Dedecker D Van Caenegem E Toye K. Short- and long-term clinical skin effects of testosterone treatment in trans men. J Sex Med. (2014) 11:222–9. doi: 10.1111/jsm.12366, PMID: 24344810

[109] Roved J Westerdahl H Hasselquist D. Sex differences in immune responses: hormonal effects, antagonistic selection, and evolutionary consequences. Horm Behav. (2017) 88:95–105. doi: 10.1016/j.yhbeh.2016.11.017, PMID: 27956226

[110] Lortet-Tieulent J Georges D Bray F Vaccarella S. Profiling global Cancer incidence and mortality by socioeconomic development. Int J Cancer. (2020) 147:3029–36. doi: 10.1002/ijc.33114, PMID: 32449164

[111] Fidler MM Soerjomataram I Bray F. A global view on Cancer incidence and National Levels of the human development index. Int J Cancer. (2016) 139:2436–46. doi: 10.1002/ijc.30382, PMID: 27522007

[112] Zhang X Han L Wei H Tan X Zhou W Li W. Linking urbanization and air quality together: a review and a perspective on the future sustainable Urban development. J Clean Prod. (2022) 346:130988. doi: 10.1016/j.jclepro.2022.130988

[113] Candolin U. Coping with light pollution in urban environments: patterns and challenges. iScience. (2024) 27:109244. doi: 10.1016/j.isci.2024.109244, PMID: 38433890 PMC10904992

[114] Stormacq C Van den Broucke S Wosinski J. Does health literacy mediate the relationship between socioeconomic status and health disparities? Integrative review. Health Promot Int. (2019) 34:e1–e17. doi: 10.1093/heapro/day062, PMID: 30107564

[115] Liu X Sangers TE Nijsten T Kayser M Pardo LM Wolvius EB. Predicting skin Cancer risk from facial images with an explainable artificial intelligence (Xai) based approach: a proof-of-concept study. EClinicalMedicine. (2024) 71:102550. doi: 10.1016/j.eclinm.2024.102550, PMID: 38545426 PMC10965465

[116] Smak Gregoor AM Sangers TE Eekhof JAH Howe S Revelman J Litjens RJM. Artificial intelligence in Mobile health for skin Cancer diagnostics at home (aim high): a pilot feasibility study. EClinicalMedicine. (2023) 60:102019. doi: 10.1016/j.eclinm.2023.102019, PMID: 37261324 PMC10227364

[117] Ersser SJ Effah A Dyson J Kellar I Thomas S McNichol E. Effectiveness of interventions to support the early detection of skin cancer through skin self-examination: a systematic review and meta-analysis. Br J Dermatol. (2019) 180:1339–47. doi: 10.1111/bjd.17529, PMID: 30561006

[118] Shah SC Kayamba V Peek RM Jr Heimburger D. Cancer control in Low- and middle-income countries: is it time to consider screening? J Glob Oncol. (2019) 5:1–8. doi: 10.1200/JGO.18.00200, PMID: 30908147 PMC6452918

[119] Hanna TP Kangolle AC. Cancer control in developing countries: using health data and health services research to measure and improve access, quality and efficiency. BMC Int Health Hum Rights. (2010) 10:24. doi: 10.1186/1472-698X-10-24, PMID: 20942937 PMC2978125

[120] Bammert P Schüttig W Novelli A Iashchenko I Spallek J Blume M. The role of Mesolevel characteristics of the health care system and socioeconomic factors on health care use – results of a scoping review. Int J Equity Health. (2024) 23:37. doi: 10.1186/s12939-024-02122-6, PMID: 38395914 PMC10885500

[121] Bryere J Tron L Menvielle G Launoy G Galateau-Salle F Bouvier A-M. The respective parts of incidence and lethality in socioeconomic differences in cancer mortality. An analysis of the French network cancer registries (Francim) data. Int J Equity Health. (2019) 18:189. doi: 10.1186/s12939-019-1087-y 31796079 PMC6891983

[122] Philips BU Jr Belasco E Markides KS Gong G. Socioeconomic deprivation as a determinant of Cancer mortality and the Hispanic paradox in Texas, USA. Int J Equity Health. (2013) 12:26. doi: 10.1186/1475-9276-12-26, PMID: 23587269 PMC3639133

[123] Ngiam NHW Yee WQ Teo N Yow KS Soundararajan A Lim JX. Building digital literacy in older adults of Low socioeconomic status in Singapore (project wire up): nonrandomized controlled trial. J Med Internet Res. (2022) 24:e40341. doi: 10.2196/40341, PMID: 36459398 PMC9758632

[124] McMaughan DJ Oloruntoba O Smith ML. Socioeconomic status and access to healthcare: interrelated drivers for healthy aging. Front Public Health. (2020) 8:231. doi: 10.3389/fpubh.2020.00231, PMID: 32626678 PMC7314918

[125] Taylor SE Seeman TE. Psychosocial resources and the Ses–health relationship. Ann N Y Acad Sci. (1999) 896:210–25. doi: 10.1111/j.1749-6632 10681899

[126] Li S He Y Liu J Chen K Yang Y Tao K. An umbrella review of socioeconomic status and Cancer. Nat Commun. (2024) 15:9993. doi: 10.1038/s41467-024-54444-2, PMID: 39557933 PMC11574020

[127] Li HO-Y Bailey AJ-M Grose E McDonald JT Quimby A Johnson-Obaseki S. Socioeconomic status and melanoma in Canada: a systematic review. J Cutan Med Surg. (2021) 25:87–94. doi: 10.1177/1203475420960426 32955341

[128] Van Hattem S Aarts MJ Louwman WJ Neumann HAM Coebergh JWW Looman CWN. Increase in basal cell carcinoma incidence steepest in individuals with high socioeconomic status: results of a Cancer registry study in the Netherlands. Br J Dermatol. (2009) 161:840–5. doi: 10.1111/j.1365-2133.2009.09222.x, PMID: 19438849

[129] Alfonso JH Martinsen JI Pukkala E Weiderpass E Tryggvadottir L Nordby KC. Occupation and relative risk of cutaneous squamous cell carcinoma (Cscc): a 45-year follow-up study in 4 Nordic countries. J Am Acad Dermatol. (2016) 75:548–55. doi: 10.1016/j.jaad.2016.03.033, PMID: 27262759

