# Supplementary material for: Global burden of skin cancer and its subtypes: a comprehensive analysis from 1990 to 2021 with projections to 2040
Source: Front Public Health. 2025 Sep 4;13:1610661. doi: 10.3389/fpubh.2025.1610661 (PMC12443545; doi:10.3389/fpubh.2025.1610661)
Supplement: Supplementary file 1 [file Supplementary_file_1.pdf]

Global Burden of Skin Cancer and its Subtypes: A Comprehensive Analysis from 1990 to 2021 with Projections to 2040

Contents

Materials and methods 3

Supplementary Figures and Tables 8

FIGURE S1 Temporal trends in incidence cases, ASIRs, DALYs, and ASDRs for skin cancers by sex (1990-2020). 8

FIGURE S2 Global distribution of ASIRs and ASDRs of skin cancers in 1990 and 2021, along with the changes observed from 1990 to 2021. 10

FIGURE S3 Incidence cases and DALYs of skin cancers by sex and age group, with age-standardized rates and 95% uncertainty intervals in 1990 and 2021. 11

FIGURE S4 Estimates of age, period, and cohort effects on incidence and DALYs of skin cancers. 12

FIGURE S5 ASIRs and ASDRs of skin cancers for 21 regions, by SDI (2021), from 1990 to 2021. 14

FIGURE S6 ASIRs and ASDRs of skin cancers for 204 countries and territories in 1990 and 2021. 15

FIGURE S7 ASIRs and ASDRs of skin cancers by sex, age group, and SDI, 1990 and 2021. 16

FIGURE S8 Population-level determinant changes in aging, population growth, and epidemiological changes for skin cancers incidence and DALYs globally and in various SDI regions from 1990 to 2021. ... 17

FIGURE S9 Absolute and relative cross-country inequalities in ASIRs and ASDRs of skin cancers, 1990-2021.. 18

FIGURE S10 Future forecasts of ASIRs and ASDRs of skin cancers. 19

TABLE S1 The disability weights for skin cancers. 20

TABLE S2 Global and regional incidence and DALYs of malignant skin melanoma in 1990 and 2021, with age-standardized rates and EAPCs from 1990 to 2021. 20

TABLE S3 Global and regional incidence and DALYs of non-melanoma skin cancer (basal-cell carcinoma) in 1990 and 2021, with age-standardized rates and EAPCs from 1990 to 2021. 23

TABLE S4 Global and regional incidence and DALYs of non-melanoma skin cancer (squamous-cell carcinoma) in 1990 and 2021, with age-standardized rates and EAPCs from 1990 to 2021. 27

TABLE S5 Global incidence and DALYs of total skin cancer from 1990 to 2021, by sex, with age-standardized rates and 95% uncertainty intervals. 30

TABLE S6 Global incidence and DALYs of malignant skin melanoma from 1990 to 2021, by sex, with age-standardized rates and 95% uncertainty intervals. 34

TABLE S7 Global incidence and DALYs of non-melanoma skin cancer (basal-cell carcinoma) from 1990 to 2021, by sex, with age-standardized rates and 95% uncertainty intervals. 37

TABLE S8 Global incidence and DALYs of non-melanoma skin cancer (squamous-cell carcinoma) from 1990 to 2021, by sex, with age-standardized rates and 95% uncertainty intervals. 41

TABLE S9 Incidence and DALYs of total skin cancer in 1990 and 2021 across 204 countries and territories, with age-standardized rates, 95% uncertainty intervals, and trends from 1990 to 2021. 45

TABLE S10 Incidence and DALYs of malignant skin melanoma in 1990 and 2021 across 204 countries and territories, with age-standardized rates, 95% uncertainty intervals, and trends from 1990 to 2021. 61

TABLE S11 Incidence and DALYs of non-melanoma skin cancer (basal-cell carcinoma) in 1990 and 2021 across 204 countries and territories, with age-standardized rates, 95% uncertainty intervals, and trends from 1990 to 2021. 75

TABLE S12 Incidence and DALYs of non-melanoma skin cancer (squamous-cell carcinoma) in 1990 and 2021 across 204 countries and territories, with age-standardized rates, 95% uncertainty intervals, and trends from 1990 to 2021. 88

TABLE S13 Proportions of age-standardized incidence rates of skin cancers globally and by region in 1990 and 2021. 98

TABLE S14 Proportions of age-standardized DALY rates of skin cancers globally and by region in 1990 and 2021. 99

TABLE S15 The number of cases and age-standardized rate of incidence and DALYs of total skin cancer by age group and sex in 1990 and 2021, with age-standardized rates and 95% uncertainty intervals. 100

TABLE S16 The number of cases and age-standardized rate of incidence and DALYs of malignant skin melanoma by age group and sex in 1990 and 2021, with age-standardized rates and 95% uncertainty intervals. 107

TABLE S17 The number of cases and age-standardized rate of incidence and DALYs of non-melanoma skin cancer (basal-cell carcinoma) by age group and sex in 1990 and 2021, with age-standardized rates and 95% uncertainty intervals. 110

TABLE S18 The number of cases and age-standardized rate of incidence and DALYs of non-melanoma skin cancer (squamous-cell carcinoma) by age group and sex in 1990 and 2021, with age-standardized rates and 95% uncertainty intervals. 114

TABLE S19 Proportions of age-standardized incidence rates of skin cancers across different age groups in 1990 and 2021. 118

TABLE S20 Proportions of age-standardized DALYs rates of skin cancers across different age groups in 1990 and 2021. 118

TABLE S21 Comparison of deviance, AIC, and BIC for four nested APC models on Incidence and DALYs for total skin cancer and its subtypes. 118

TABLE S22 Age-standardized incidence and DALYs rates of total skin cancer and its subtypes for 21 GBD regions, by year (1990-2021). 1190

TABLE S23 Age-standardized rates of incidence and DALYs of total skin cancer for global and SDI regions, by year (1990, 2021), age group and sex, with age-standardized rates and 95% uncertainty intervals. 181

TABLE S24 Age-standardized rates of incidence and DALYs of malignant skin melanoma for global and SDI regions, by year (1990, 2021), age group and sex, with age-standardized rates and 95% uncertainty intervals. 190

TABLE S25 Age-standardized rates of incidence and DALYs of non-melanoma skin cancer (basal-cell carcinoma) for global and SDI regions, by year (1990, 2021), age group and sex, with age-standardized rates and 95% uncertainty intervals. 200

TABLE S26 Age-standardized rates of incidence and DALYs of non-melanoma skin cancer (squamous-cell carcinoma) for global and SDI regions, by year (1990, 2021), age group and sex, with age-standardized rates and 95% uncertainty intervals. 209

TABLE S27 Changes in population-level determinants, including aging, population growth, and epidemiological factors, on the incidence and DALYs of total skin cancer globally and across different SDI regions from 1990 to 2021. 219

TABLE S28 Changes in population-level determinants, including aging, population growth, and epidemiological factors, on the incidence and DALYs of malignant skin melanoma globally and across different SDI regions from 1990 to 2021. 220

TABLE S29 Changes in population-level determinants, including aging, population growth, and epidemiological factors, on the incidence and DALYs of non-melanoma skin cancer (basal-cell carcinoma) globally and across different SDI regions from 1990 to 2021. 221

TABLE S30 Changes in population-level determinants, including aging, population growth, and epidemiological factors, on the incidence and DALYs of non-melanoma skin cancer (squamous-cell carcinoma) globally and across different SDI regions from 1990 to 2021. 222

TABLE S31 Predicated age-standardized rates of incidence and DALYs of total skin cancer by sex from 2022 to 2040, with 95% uncertainty intervals. 224

TABLE S32 Predicated age-standardized rates of incidence and DALYs of malignant skin melanoma by sex from 2022 to 2040, with 95% uncertainty intervals. 225

TABLE S33 Predicated age-standardized rates of incidence and DALYs of non-melanoma skin cancer (basal-cell carcinoma) by sex from 2022 to 2040, with 95% uncertainty intervals. 226

TABLE S34 Predicated age-standardized rates of incidence and DALYs of non-melanoma skin cancer (squamous-cell carcinoma) by sex from 2022 to 2040, with 95% uncertainty intervals. 227

1 Materials and methods

1.1 IHME methodological framework for managing sparse cancer registry data

IHME employs distinctive methodological approaches to address limitations arising from sparse data. For countries with sparse cancer registry data, a multi-step data processing framework is utilized, encompassing completeness assessment, spatial-temporal modeling, and uncertainty quantification. During the completeness assessment phase, only population‐ based cancer registries with well‐ defined population coverage and official source documentation are retained, while systems lacking adequate coverage documentation or those with prolonged update intervals are excluded. Although technical reviews incorporate indicators such as death certificate only percentage (DCO%) and morphological verification percentage (MV%), these metrics are not employed as triggers for completeness corrections or rigid exclusion thresholds. Regions with lower-quality registries undergo subsequent modeling and Bayesian "soft corrections," thereby minimizing direct data exclusion and preserving more comprehensive information. Subsequently, mortality-to-incidence (MI) ratios derived from high-quality registries with comparable epidemiological profiles are utilized by remapping algorithms to estimate incidence in data-sparse regions. These algorithms employ spatiotemporal Gaussian process regression (ST‐ GPR) incorporating covariates such as healthcare access, cancer screening coverage, and socio‐ demographic indices to model MI ratios across geographical and temporal dimensions, thereby enabling cancer incidence estimation in data‐ sparse

regions while enhancing estimate robustness. Multiple sources of variability are incorporated in uncertainty propagation: sampling uncertainty from sparse primary data, model uncertainty from MI ratio estimation, and systematic uncertainty from completeness corrections. Monte Carlo simulations are conducted using 500 posterior draws, generating point estimates with 95% uncertainty intervals that exhibit wider uncertainty ranges to reflect the high variability inherent in input data. These methodological considerations particularly affect skin cancer estimates in sub-Saharan Africa and parts of Asia, where registry coverage remains below 20% of the total population. Consequently, skin cancer burden estimates for these regions exhibit substantially larger uncertainty intervals (typically 2-3-fold wider) compared with countries possessing comprehensive registry systems, potentially resulting in an underestimation of the true disease burden due to conservative modeling assumptions in data-sparse environments.

1.2 The disability weights, age-weighting and discounting conventions

The GBD 2021 study employed standardized disability weights that were derived from population- based survey methodology (1). These disability weights were obtained from the "GBD 2021 Disability Weights" CSV file provided by the IHME GHDx database. The disability weights for skin cancers are presented in TABLE S1. Notably, GBD 2021 did not apply age-weighting or time discounting, thereby representing a departure from earlier GBD studies in which annual discounting and non-uniform age weights had been employed (2).

1.3 Age-period-cohort model analysis

The effect of age, period, and cohort (3) on the risk of total skin cancer and its subtypes was assessed using the age-period-cohort (APC) model. Age effect represents differences in the risk of total skin cancer and its subtypes across age groups resulting from age-related biological and physiological factors. Period effect reflects the impact of temporal changes from 1990 to 2021, including advancements in healthcare systems, diagnostic technologies, and public health interventions. Cohort effect refers to variations in risk across different birth cohorts, arising from differential exposure to environmental, behavioral, and socioeconomic risk factors over the course of their lives. The APC model is typically described as follows.

$$\log(\boldsymbol{M}) = \boldsymbol{\mu} + \boldsymbol{\alpha}(\boldsymbol{age})\boldsymbol{i} + \boldsymbol{\beta}(\boldsymbol{period})\boldsymbol{j} + \boldsymbol{\gamma}(\boldsymbol{cohort})\boldsymbol{k} + \boldsymbol{\varepsilon}.$$

In this equation, M represents the ASRs of total skin cancer and its subtypes, μ and ε denote the intercept and random error, respectively, while $\alpha(age)i$, $\beta(period)j$, and $\gamma(cohort)k$ represent the effects of age group α, time period β, and birth cohort γ, respectively. Using the web-based analytical tool (https://analysistools.cancer.gov/apc) (4), the APC analysis was conducted. Knots were established based on available age and period groups, implementing uniform 5-year intervals. The uniform knot placement strategy was advantageous for maintaining consistency across temporal analyses, facilitating comparability among age, period, and cohort effects, while effectively capturing long-term trends without over-parameterizing the model. Specifically, the reference settings for age, period, and cohort were determined according to the following median calculation formula:

$$\boldsymbol{Reference\ Age} = (\boldsymbol{Number\ of\ Age\ Groups} + \mathbf{1})/\mathbf{2}$$

$$\boldsymbol{Reference\ Period} = (\boldsymbol{Number\ of\ Periods} + \mathbf{1})/\mathbf{2}$$

$$\boldsymbol{Reference\ Cohort} = (\boldsymbol{Reference\ Period} - \boldsymbol{Reference\ Age} + \boldsymbol{Number\ of\ Age\ Groups})$$

As these variables represent ranges, reference points were calculated using the following formula:

$$\boldsymbol{Period\ and\ Age\ Points}: (\boldsymbol{Lower\ Value} + \boldsymbol{Upper\ Value} + \mathbf{1})/\mathbf{2}$$

$$\boldsymbol{Cohort\ Point}: [(\boldsymbol{Lower\ Period\ Value} + \boldsymbol{Upper\ Period\ Value} + \mathbf{1})/\mathbf{2}] - [(\boldsymbol{Lower\ Age\ Value} + \boldsymbol{Upper\ Age\ Value} + \mathbf{1})/\mathbf{2}],\ \boldsymbol{or\ more\ simply}$$

$$\boldsymbol{Cohort\ Point}: (\boldsymbol{Reference\ Period\ Point} - \boldsymbol{Reference\ Age\ Point})$$

The APC model was employed to estimate both the overall temporal trend and the incidence trend within each age group. The former was expressed as the annual percentage change in incidence, representing the net drift (% per year), which is influenced by calendar time and continuous birth cohorts. The latter represented the percentage change in annual incidence by age, designated as the local drift (% per year). This study primarily focused on examining the net drift of skin cancer incidence. In contrast to the EAPC, which emphasizes granular year-to-year variations, the net drift provided a macroscopic perspective on changes in disease burden. By incorporating period and cohort effects, the net drift eliminated the influence of demographic structure, thereby enabling an independent assessment of skin cancer incidence trends. This approach provided policymakers with a more precise tool for disease burden evaluation, facilitating the development and adjustment of effective health intervention policies without demographic structural bias, particularly for long-term disease trend prediction and response strategies.

Although the APC web tool is widely utilized for APC analysis, it did not directly report goodness-of-fit statistics. Its output was supplemented by comparing nested models—age only (A), age-period (AP), age-cohort (AC), and the full APC model—using deviance and information criteria including AIC and BIC. Following model fitting, deviance, the Akaike Information Criterion (AIC), and the Bayesian Information Criterion (BIC) were calculated to assess relative model performance.

1.4 Decomposition analysis

To identify the primary factors influencing the variations in the burden of total skin cancer and its subtypes from 1990 to 2021, the Das Gupta (5-7) decomposition method was employed to decompose changes in skin cancer incidence rates and DALYs into three components: population aging, population growth, and epidemiological changes. The calculation formula is presented as follows:

$$\boldsymbol{A_{ay,py,ey}} = \sum_{\boldsymbol{i=1}}^{\mathbf{20}} (\boldsymbol{a_{i,y}} \times \boldsymbol{p_y} \times \boldsymbol{e_{i,y}})$$

A indicates incidence or DALYs. $\mathbf{A_{ay,py,ey}}$ represents the incidence/DALYs number accumulated by population aging, population growth and epidemiological changes in year y; $\boldsymbol{a_{i,y}}$ represents the population proportion of the age group i in year y; $\boldsymbol{p_y}$ represents the total population in a given year y; $\boldsymbol{e_{i,y}}$ represented the incidence/DALYs rate for a specific age group i in year y. The contribution of each factor to the change in incidence/DALYs from 1990 to 2021 was defined as the effect of varying one factor while maintaining

the other factors at baseline levels. For example, the effect of age structure was calculated using the following formula:

$$\boldsymbol{Effect}_{2021}= \left(\frac{A_{a2021,p2021,e2021}+A_{a2021,p1990,e1990}}{3}+\frac{A_{a2021,p1990,e2021}+A_{a2021,p2021,e1990}}{6}\right) - \left(\frac{A_{a1990,p2021,e2021}+A_{a1990,p1990,e1990}}{3}+\frac{A_{a1990,p1990,e2021}+A_{a1990,p2021,e1990}}{6}\right)$$

A indicates incidence or DALYs.

1.5 Cross-country inequality analysis

To identify the cross-country inequalities in the burden of total skin cancer and its subtypes, SII and CI were calculated as the standardized measures (8). The SII was employed to quantify absolute inequality in health indicators between the most and least socioeconomically advantaged subgroups within a population. In contrast, the CI was employed to quantify relative inequality by measuring the extent to which health indicators were concentrated among socioeconomically disadvantaged or advantaged populations. A positive SII indicated a greater disease burden among high-SDI populations, while a negative SII indicated a greater burden among low-SDI populations. A positive CI indicated that the disease burden was primarily concentrated among high-SDI populations, whereas a negative CI indicated such concentration among low-SDI populations. The SII was derived from regression analysis, which modeled the association between national-level incidence and DALY rates with their relative positions on the SDI scale. These positions were determined by the midpoints of population distribution within the cumulative SDI ranking (9). Heteroscedasticity was controlled through the application of a weighted regression model. CI was calculated through numerical summation of the Lorenz curve, which depicts the cumulative percentage of the incidence and DALYs for total skin cancer and its subtypes against the cumulative population distribution, ranked by the SDI (9). A negative SII/CI indicates an inverse relationship between SDI and both incidence and DALYs, while a positive value suggests a direct relationship. The magnitude of the SII/CI reflects the level of inequality, with larger absolute values corresponding to greater disparities (9).

1.6 Predictive analysis

To conduct the Bayesian Age-Period-Cohort (BAPC) model, the BAPC packages (available from https://r-forge.r-project.org/) and the INLA packages (available from https://www.r-inla.org/) was used, along with global population projection data for 2017-2100. ASIR and ASDR of total skin cancer and its subtypes were predicted from 2022 through 2040.

1.7 Reference

1. Murray CJ, Ezzati M, Flaxman AD, Lim S, Lozano R, Michaud C, et al. GBD 2010: design, definitions, and metrics. Lancet. (2012) 380(9859):2063-6. doi:10.1016/S0140-6736(12)61899-6.
2. Salomon JA, Vos T, Hogan DR, Gagnon M, Naghavi M, Mokdad A, et al. Common values in assessing health outcomes from disease and injury: disability weights measurement study for the Global Burden of Disease Study 2010. Lancet. (2012) 380(9859):2129-43. doi:10.1016/S0140-6736(12)61680-8.
3. Rosenberg PS. A new age-period-cohort model for cancer surveillance research. Stat Methods Med Res. (2019) 28(10-11):3363-91. doi:10.1177/0962280218801121.

4．Rosenberg PS, Check DP, Anderson WF. A web tool for age-period-cohort analysis of cancer incidence and mortality rates. Cancer Epidemiol Biomarkers Prev. (2014) 23(11):2296-302. doi:10.1158/1055-9965.EPI-14-0300.

5．Das Gupta, P. Standardization and decomposition of rates：a user’s manual. D.C: U.S. Dept. of Commerce, Economics and Statistics Administration, Bureau of the Census (1993). Pages 19-36.

6．Das Gupta P. Standardization and decomposition of rates from cross-classified data. Genus. (1994) 50: 171-196.

7．Chevan A, Sutherland M. Revisiting Das Gupta: refinement and extension of standardization and decomposition. Demography. (2009) 46(3):429-49. doi:10.1353/dem.0.0060.

8．Luo Z, Shan S, Cao J, Zhou J, Zhou L, Jiang D, et al. Temporal trends in cross-country inequalities of stroke and subtypes burden from 1990 to 2021: a secondary analysis of the global burden of disease study 2021. EClinicalMedicine. (2024) 76:102829. doi: 10.1016/j.eclinm.2024.102829.

9．Ordunez P, Martinez R, Soliz P, Giraldo G, Mujica OJ, Nordet P. Rheumatic heart disease burden, trends, and inequalities in the Americas, 1990-2017: a population-based study. Lancet Glob Health. (2019) 7(10):e1388-e1397. doi: 10.1016/S2214-109X(19)30360-2.

2 Supplementary Figures and Tables

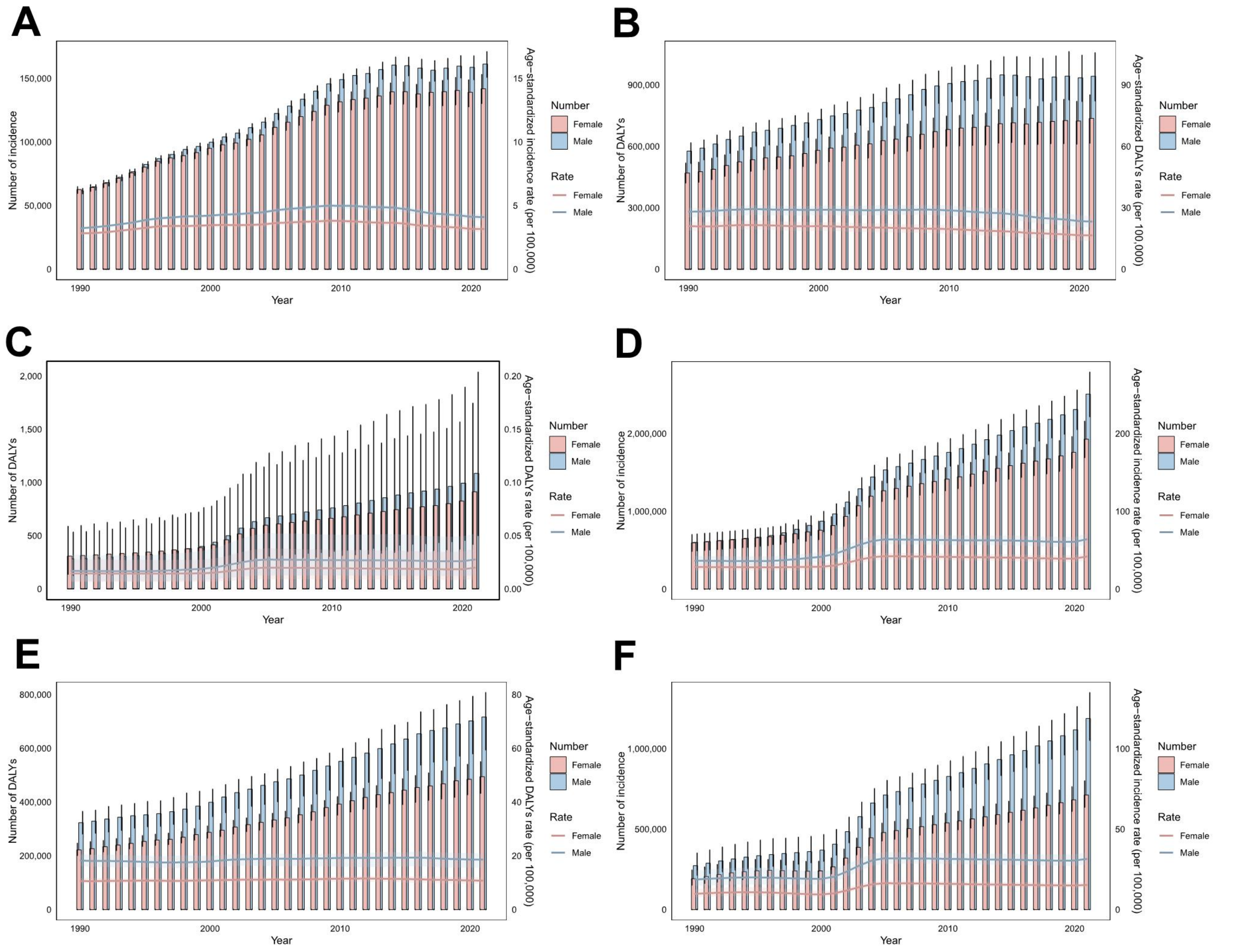

FIGURE S1

Temporal trends in incidence cases, ASIRs, DALYs, and ASDRs for skin cancers by sex (1990-2020). **(A)** Incidence cases and ASIRs for malignant skin melanoma. **(B)** DALYs and ASDRs for malignant skin melanoma. **(C)** Incidence cases and ASIRs for non-melanoma skin cancer (basal-cell carcinoma). **(D)** DALYs and ASDRs for non-melanoma skin cancer (basal-cell carcinoma). **(E)** Incidence cases and ASIRs for non-melanoma skin cancer (squamous-cell carcinoma). **(F)** DALYs and ASDRs for non-melanoma skin cancer (squamous-cell carcinoma). The pink or blue regions around the curve represent the upper and lower limits of the 95% uncertainty interval (UI). DALYs, disability-adjusted life-years; ASIR, age-standardized rates of incidence; ASDR, age-standardized rates of DALYs.

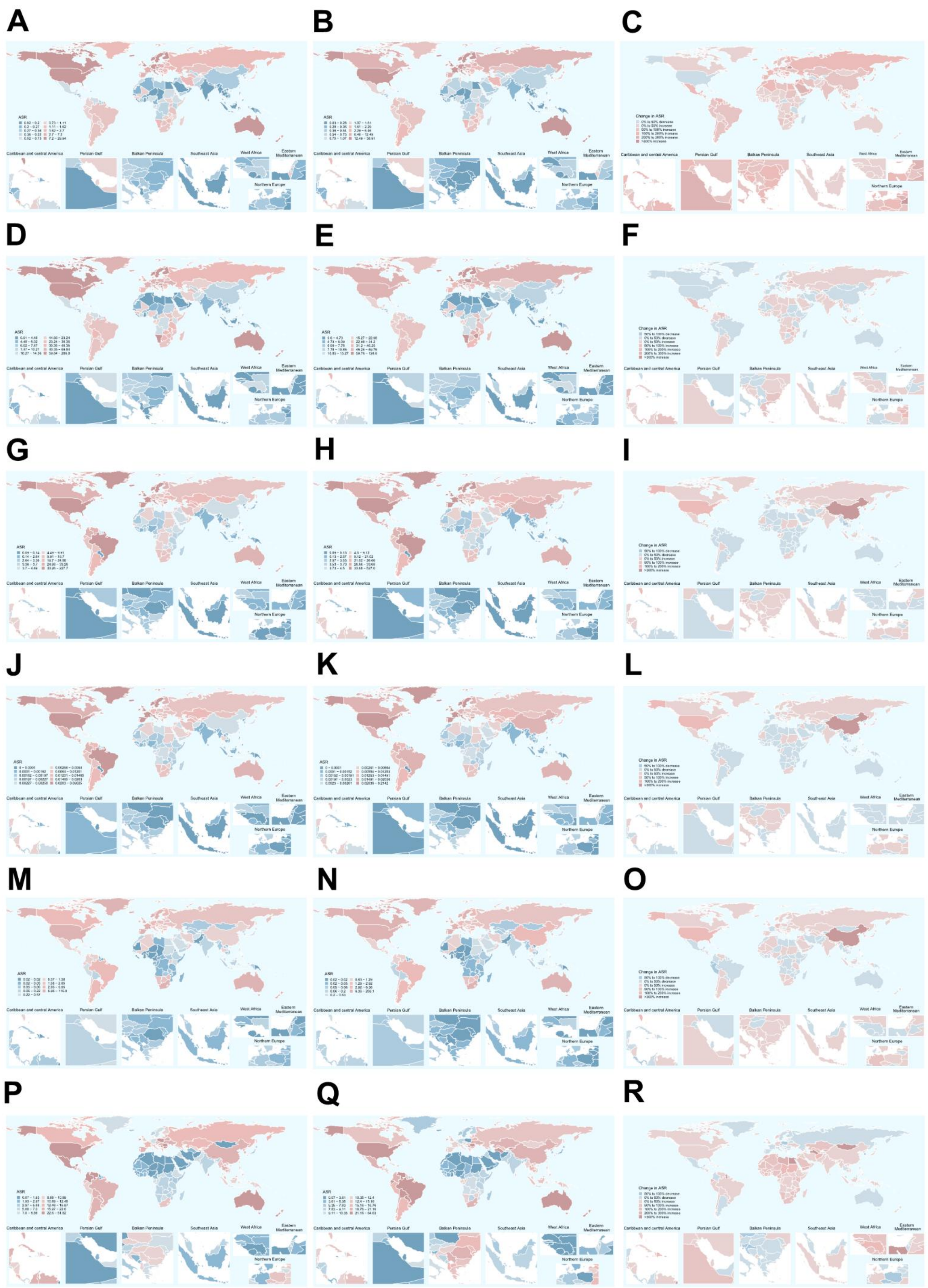
A
B
C
D
E
F
G
H
I
J
K
L
M
N
O
P
Q
R

FIGURE S2

Global distribution of ASIRs and ASDRs of skin cancers in 1990 and 2021, along with the changes observed from 1990 to 2021. **(A)** ASIRs of malignant skin melanoma in 1990; **(B)** ASIRs of malignant skin melanoma in 2021; **(C)** The changes in ASIR of malignant skin melanoma from 1990 to 2021; **(D)** ASDRs of malignant skin melanoma in 1990; **(E)** ASDRs of malignant skin melanoma in 2021; **(F)** The changes in ASDR of malignant skin melanoma from 1990 to 2021; **(G)** ASIRs of non-melanoma skin cancer (basal-cell carcinoma) in 1990; **(H)** ASIRs of non-melanoma skin cancer (basal-cell carcinoma) in 2021; **(I)** The changes in ASIR of non-melanoma skin cancer (basal-cell carcinoma) from 1990 to 2021; **(J)** ASDRs of non-melanoma skin cancer (basal-cell carcinoma) in 1990; **(K)** ASDRs of non-melanoma skin cancer (basal-cell carcinoma) in 2021; **(L)** The changes in ASDR of non-melanoma skin cancer (basal-cell carcinoma) from 1990 to 2021; **(M)** ASIRs of non-melanoma skin cancer (squamous-cell carcinoma) in 1990; **(N)** ASIRs of non-melanoma skin cancer (squamous-cell carcinoma) in 2021; **(O)** The changes in ASIR of non-melanoma skin cancer (squamous-cell carcinoma) from 1990 to 2021; **(P)** ASDRs of non-melanoma skin cancer (squamous-cell carcinoma) in 1990; **(Q)** ASDRs of non-melanoma skin cancer (squamous-cell carcinoma) in 2021; **(R)** The changes in ASDR of non-melanoma skin cancer (squamous-cell carcinoma) from 1990 to 2021; ASIR, age-standardized rate of incidence; ASDR, age-standardized rate of DALYs; DALYs, disability-adjusted life-years.

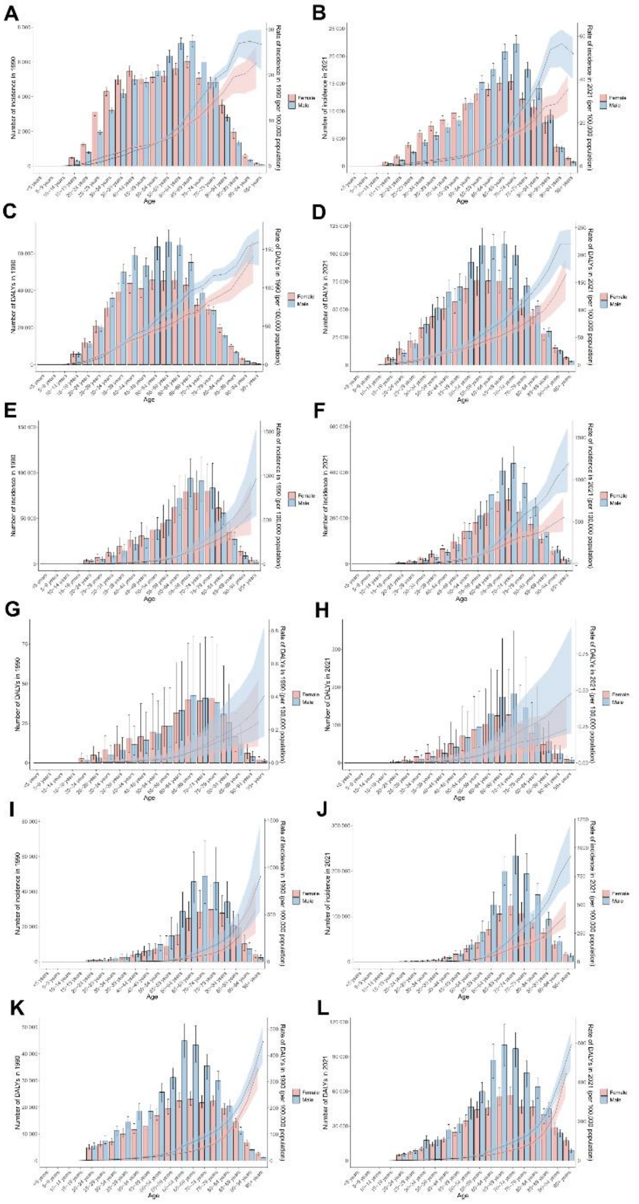

FIGURE S3

Incidence cases and DALYs of skin cancers by sex and age group, with age-standardized rates and 95% uncertainty intervals in 1990 and 2021. **(A)** Incidence cases and ASIRs of malignant skin melanoma in 1990; **(B)** Incidence cases and ASIRs of malignant skin melanoma in 2021; **(C)** DALYs and ASDRs of malignant skin melanoma in 1990; **(D)** DALYs and ASDRs of malignant skin melanoma in 2021; **(E)** Incidence cases and ASIRs of non-melanoma skin cancer (basal-cell carcinoma) in 1990; **(F)** Incidence cases and ASIRs of non-melanoma skin cancer (basal-cell carcinoma) in 2021; **(G)** DALYs and ASDRs of non-melanoma skin cancer (basal-cell carcinoma) in 1990; **(H)** DALYs and ASDRs of non-melanoma skin cancer (basal-cell carcinoma) in 2021; **(I)** Incidence cases and ASIRs of non-melanoma skin cancer (squamous-cell carcinoma) in 1990; **(J)** Incidence cases and ASIRs of non-melanoma skin cancer (squamous-cell carcinoma) in 2021; **(K)** DALYs and ASDRs of non-melanoma skin cancer (squamous-cell carcinoma) in 1990; **(L)** DALYs and ASDRs of non-melanoma skin cancer (squamous-cell carcinoma) in 2021. The pink or blue regions around the curve represent the upper and lower limits of the 95% uncertainty interval (UI). DALYs, disability-adjusted life-years; ASIR, age-standardized rate of incidence; ASDR, age-standardized rate of DALYs.

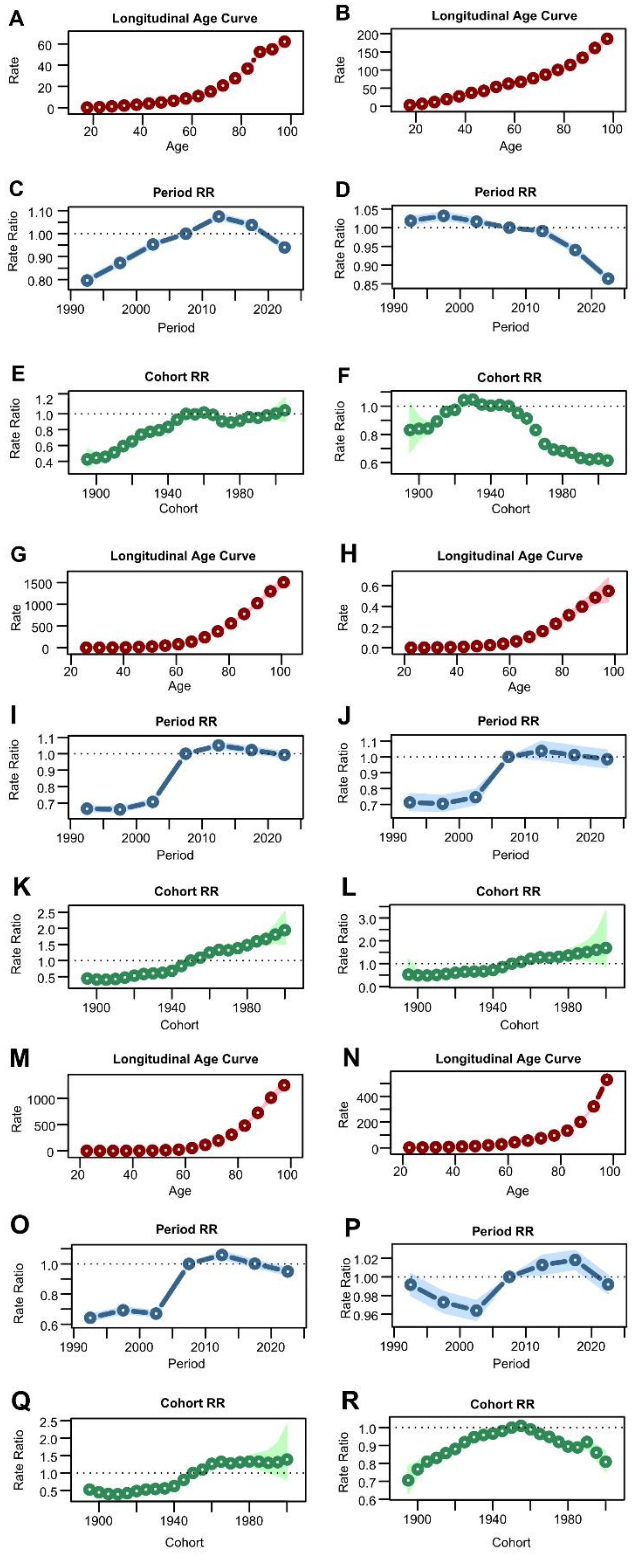

FIGURE S4

Estimates of age, period, and cohort effects on incidence and DALYs of skin cancers. **(A)** Estimates of age effect on incidence of malignant skin melanoma; **(B)** Estimates of age effect on DALYs of malignant skin melanoma; **(C)** Estimates of period effect on incidence of malignant skin melanoma; **(D)** Estimates of period effect on DALYs of malignant skin melanoma; **(E)** Estimates of cohort effect on incidence of

malignant skin melanoma; **(F)** Estimates of cohort effect on DALYs of malignant skin melanoma; **(G)** Estimates of age effect on incidence of non-melanoma skin cancer (basal-cell carcinoma); **(H)** Estimates of age effect on DALYs of non-melanoma skin cancer (basal-cell carcinoma); **(I)** Estimates of period effect on incidence of non-melanoma skin cancer (basal-cell carcinoma); **(J)** Estimates of period effect on DALYs of non-melanoma skin cancer (basal-cell carcinoma); **(K)** Estimates of cohort effect on incidence of non-melanoma skin cancer (basal-cell carcinoma); **(L)** Estimates of cohort effect on DALYs of non-melanoma skin cancer (basal-cell carcinoma); **(M)** Estimates of age effect on incidence of non-melanoma skin cancer (squamous-cell carcinoma); **(N)** Estimates of age effect on DALYs of non-melanoma skin cancer (squamous-cell carcinoma); **(O)** Estimates of period effect on incidence of non-melanoma skin cancer (squamous-cell carcinoma); **(P)** Estimates of period effect on DALYs of non-melanoma skin cancer (squamous-cell carcinoma); **(Q)** Estimates of cohort effect on incidence of non-melanoma skin cancer (squamous-cell carcinoma); **(R)** Estimates of cohort effect on DALYs of non-melanoma skin cancer (squamous-cell carcinoma); DALYs, disability-adjusted life-years.

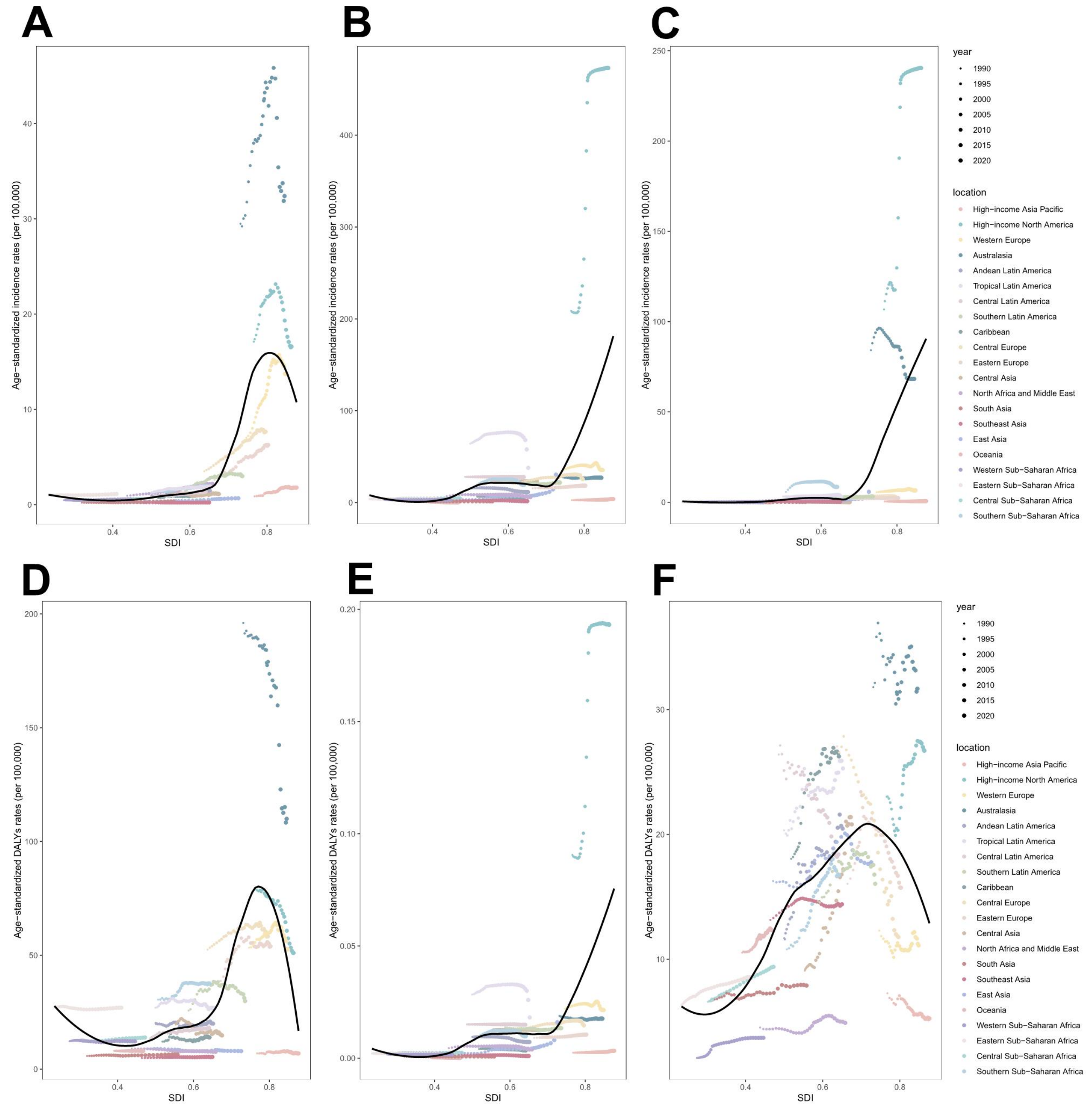

FIGURE S5

ASIRs and ASDRs of skin cancers for 21 regions, by SDI (2021), from 1990 to 2021. **(A)** ASIRs of malignant skin melanoma. **(B)** ASIRs of non-melanoma skin cancer (basal-cell carcinoma). **(C)** ASIRs of non-melanoma skin cancer (squamous-cell carcinoma). **(D)** ASDRs of malignant skin melanoma. **(E)** ASDRs of non-melanoma skin cancer (basal-cell carcinoma). **(F)** ASDRs of non-melanoma skin cancer (squamous-cell carcinoma). 32 points are plotted for each region and show the observed ASIRs and ASDRs for each year from 1990 to 2021. Expected values are shown as the black line. Points above the black line represent a higher-than-expected burden, and those below the line show a lower-than-expected burden. DALYs, disability-adjusted life-years; ASIR, age-standardized rates of incidence; ASDR, age-standardized rates of DALYs.

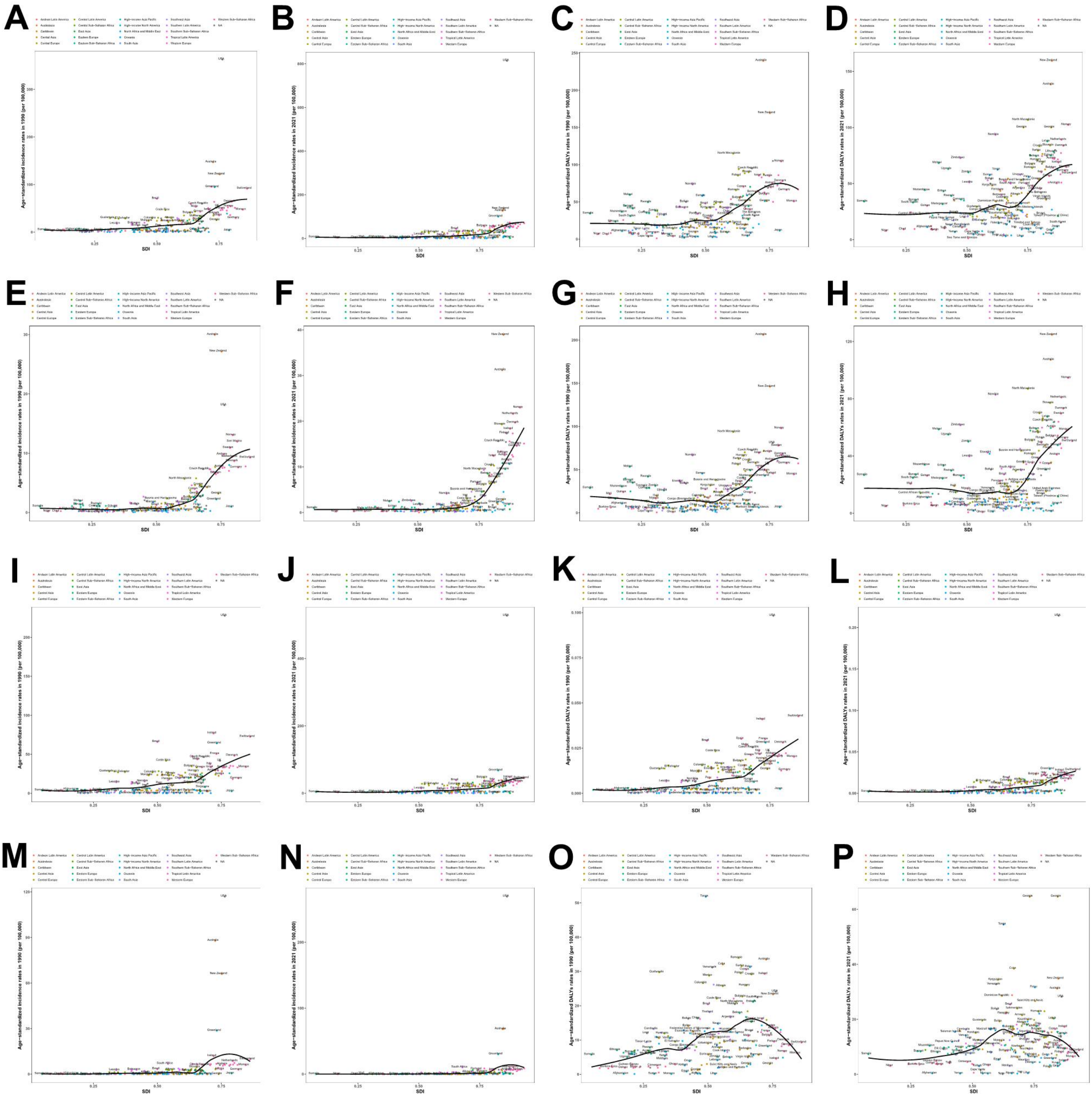

FIGURE S6

ASIRs and ASDRs of skin cancers for 204 countries and territories in 1990 and 2021. **(A)** ASIRs of total skin cancer in 1990; **(B)** ASIRs of total skin cancer in 2021; **(C)** ASDRs of total skin cancer in 1990; **(D)** ASDRs of total skin cancer in 2021; **(E)** ASIRs of malignant skin melanoma in 1990; **(F)** ASIRs of malignant skin melanoma in 2021; **(G)** ASDRs of malignant skin melanoma in 1990; **(H)** ASDRs of malignant skin melanoma in 2021; **(I)** ASIRs of non-melanoma skin cancer (basal-cell carcinoma) in 1990; **(J)** ASIRs of non-melanoma skin cancer (basal-cell carcinoma) in 2021; **(K)** ASDRs of non-melanoma skin cancer (basal-cell carcinoma) in 1990; **(L)** ASDRs of non-melanoma skin cancer (basal-cell carcinoma) in 2021; **(M)** ASIRs of non-melanoma skin cancer (squamous-cell carcinoma) in 1990; **(N)** ASIRs of non-melanoma skin cancer (squamous-cell carcinoma) in 2021; **(O)** ASDRs of non-melanoma skin cancer

(squamous-cell carcinoma) in 1990; **(P)** ASDRs of non-melanoma skin cancer (squamous-cell carcinoma) in 2021. Total skin cancer includes malignant skin melanoma, non-melanoma skin cancer (basal-cell carcinoma) and non-melanoma skin cancer (squamous-cell carcinoma). Expected values are shown as the black line. DALYs, disability-adjusted life-years; ASIR, age-standardized rate of incidence, ASDR: age-standardized rate of DALYs.

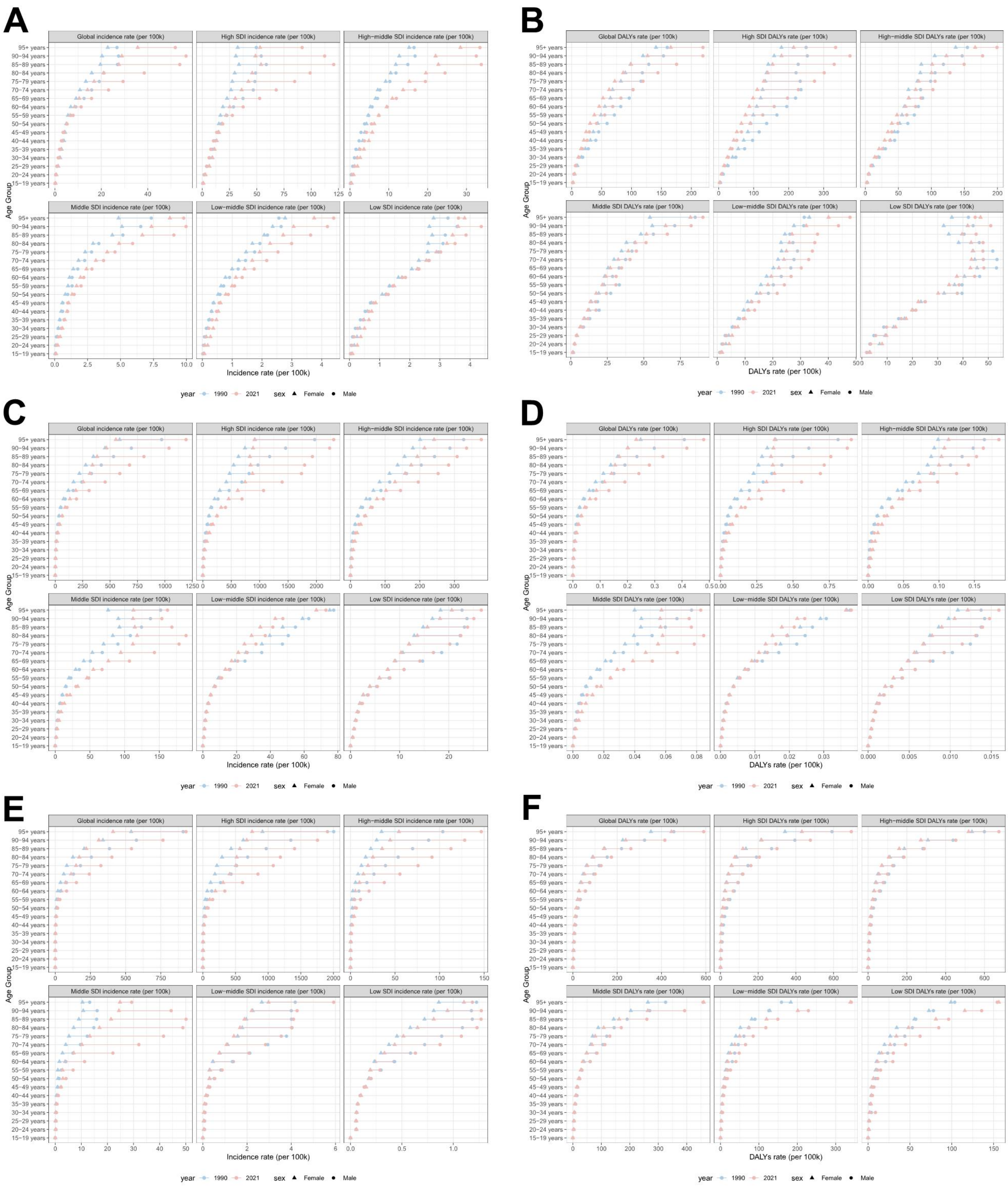

FIGURE S7

[i]ASIRs and ASDRs of skin cancers by sex, age group, and SDI, 1990 and 2021. **(A)** ASIRs of malignant skin melanoma; **(B)** ASDRs of malignant skin melanoma; **(C)** ASIRs of non-melanoma skin cancer (basal-

cell carcinoma); **(D)** ASDRs of non-melanoma skin cancer (basal-cell carcinoma); **(E)** ASIRs of non-melanoma skin cancer (squamous-cell carcinoma); **(F)** ASDRs of non-melanoma skin cancer (squamous-cell carcinoma); DALYs, disability-adjusted life-years; ASIR, age-standardized rate of incidence; ASDR, age-standardized rate of DALYs; SDI, socio-demographic index.

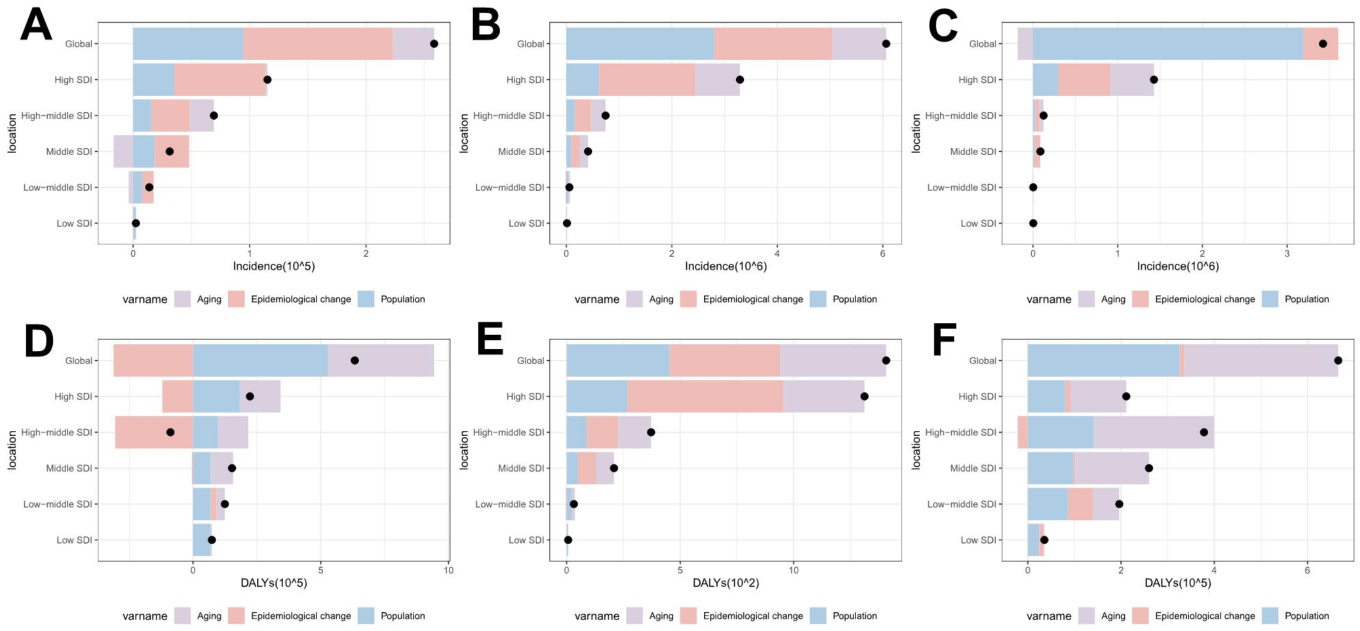

FIGURE S8

Population-level determinant changes in aging, population growth, and epidemiological changes for skin cancers incidence and DALYs globally and in various SDI regions from 1990 to 2021. **(A)** Incidence of malignant skin melanoma; **(B)** Incidence of non-melanoma skin cancer (basal-cell carcinoma); **(C)** Incidence of non-melanoma skin cancer (squamous-cell carcinoma); **(D)** DALYs of malignant skin melanoma; **(E)** DALYs of non-melanoma skin cancer (basal-cell carcinoma); **(F)** DALYs of non-melanoma skin cancer (squamous-cell carcinoma). Black dots represent the total change contributed by all three components. A positive value for each component indicates a corresponding positive contribution in incidence or DALYs, and a negative value indicates a corresponding negative contribution in incidence or DALYs. DALYs, disability-adjusted life-years; SDI, socio-demographic index.

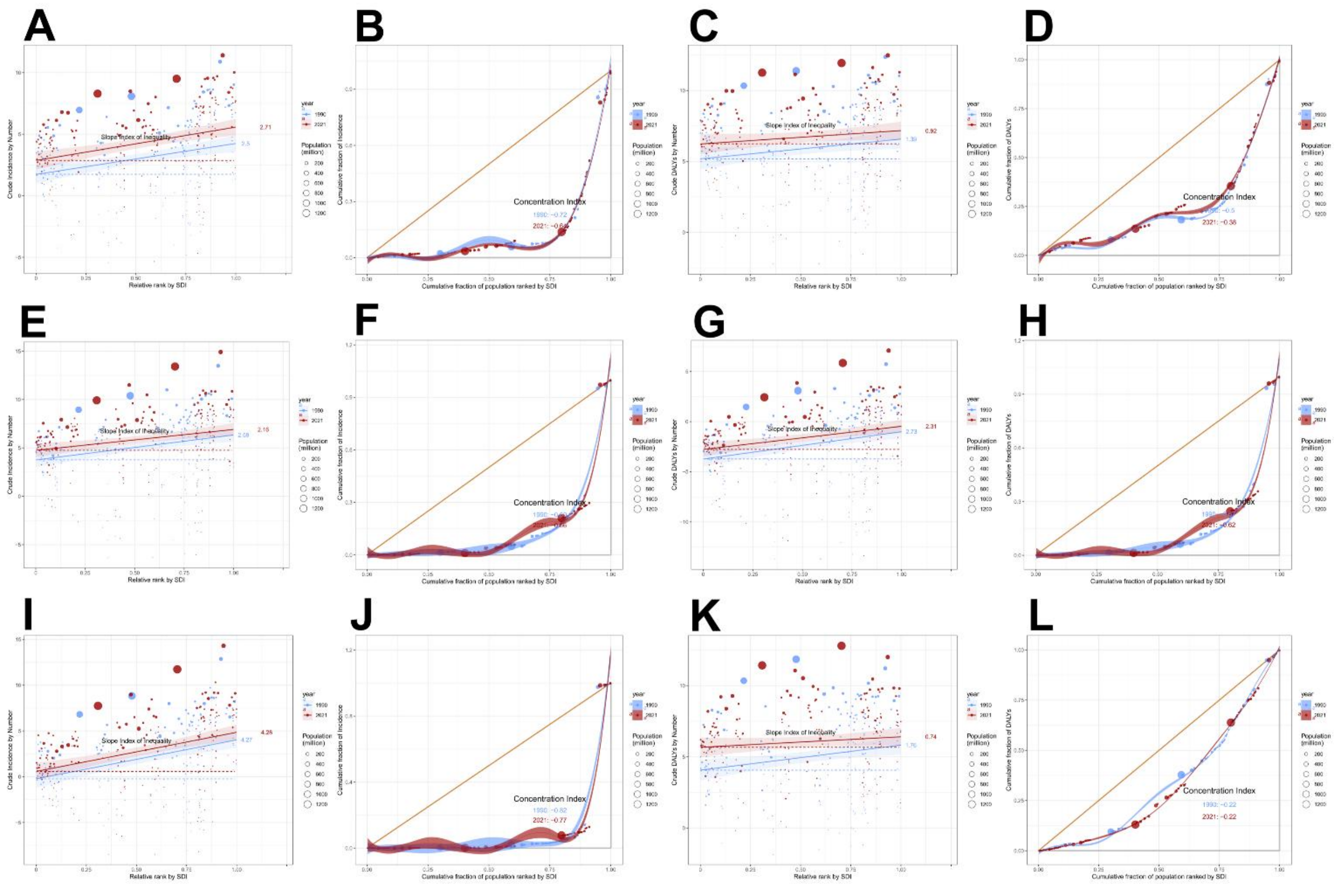

FIGURE S9

Absolute and relative cross-country inequalities in ASIRs and ASDRs of skin cancers, 1990-2021. **(A)** Health inequality regression curves for ASIRs of malignant skin melanoma; **(B)** Concentration curves for ASIRs of malignant skin melanoma; **(C)** Health inequality regression curves for ASDRs of malignant skin melanoma; **(D)** Concentration curves for ASDRs of malignant skin melanoma; **(E)** Health inequality regression curves for ASIRs of non-melanoma skin cancer (basal-cell carcinoma); **(F)** Concentration curves for ASIRs of non-melanoma skin cancer (basal-cell carcinoma); **(G)** Health inequality regression curves for ASDRs of non-melanoma skin cancer (basal-cell carcinoma); **(H)** Concentration curves for ASDRs of non-melanoma skin cancer (basal-cell carcinoma); **(I)** Health inequality regression curves for ASIRs of non-melanoma skin cancer (squamous-cell carcinoma); **(J)** Concentration curves for ASIRs of non-melanoma skin cancer (squamous-cell carcinoma); **(K)** Health inequality regression curves for ASDRs of non-melanoma skin cancer (squamous-cell carcinoma); **(L)** Concentration curves for ASDRs of non-melanoma skin cancer (squamous-cell carcinoma); DALYs, disability-adjusted life-years; ASIR, age-standardized rate of incidence; ASDR, age-standardized rate of DALYs.

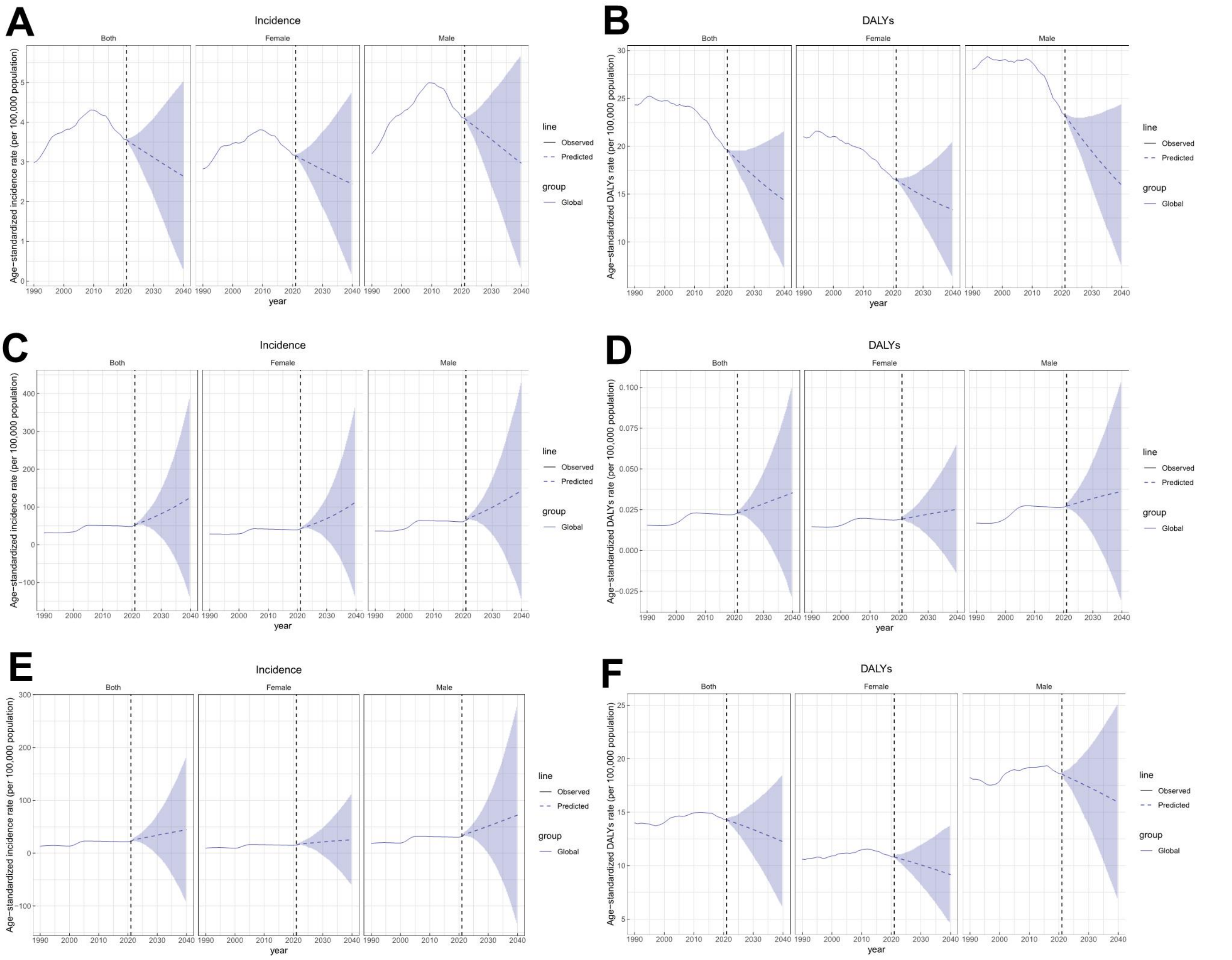

FIGURE S10

Future forecasts of ASIRs and ASDRs of skin cancers. (A) ASIRs of malignant skin melanoma; (B) ASDRs of malignant skin melanoma; (C) ASIRs of non-melanoma skin cancer (basal-cell carcinoma); (D) ASDRs of non-melanoma skin cancer (basal-cell carcinoma); (E) ASIRs of non-melanoma skin cancer (squamous-cell carcinoma); (F) ASDRs of non-melanoma skin cancer (squamous-cell carcinoma). The purple region shows the upper and lower limits of the 95% uncertainty interval (UI). DALYs, disability-adjusted life-years; ASIR, age-standardized rate of incidence; ASDR, age-standardized rate of DALYs

TABLE S1 The disability weights for skin cancers.

Cancer Type	Health State / Phase	Mean DW	95% UI Lower	95% UI Upper
Malignant melanoma	Diagnosis and primary therapy phase	0.2875	0.1926	0.3989
	Metastatic phase	0.4514	0.3072	0.6003
	Terminal phase	0.5396	0.3767	0.6871
	Controlled phase	0.049	0.0311	0.0721
Squamous cell carcinoma (SCC)	Disfigurement level 1 (mild)	0.011	0.0052	0.0206
	Disfigurement level 2 (moderate)	0.067	0.0444	0.0959
	Disfigurement level 3 (severe, with itch/pain)	0.5763	0.4014	0.7308
Basal cell carcinoma (BCC)	Asymptomatic	0	0	0
	Disfigurement level 1 (mild)	0.011	0.0052	0.0206

TABLE S2 Global and regional incidence and DALYs of malignant skin melanoma in 1990 and 2021, with age-standardized rates and EAPCs from 1990 to 2021.

	Incidence					DALYs				
	All ages number (95% UI), 1990	Age-standardized rate (95% UI), 1990	All ages number (95% UI), 2021	Age-standardized rate (95% UI), 2021	EAPC (95% CI), 1990-2021	All ages number (95% UI), 1990	Age-standardized rate (95% UI), 1990	All ages number (95% UI), 2021	Age-standardized rate (95% UI), 2021	EAPC (95% CI), 1990-2021
Global										
Male	61693 (59290 to 63466)	3.21 (3.08 to 3.3)	161316 (150890 to 171043)	4.1 (3.82 to 4.36)	0.89 (0.53 to 1.24)	576638 (517184 to 617161)	28.07 (25.29 to 29.95)	942404 (822098 to 1056864)	23.23 (20.33 to 26)	-0.54 (-0.72 to -0.37)
Female	62627 (59680	2.83 (2.69 to	141789 (130335	3.16 (2.9 to	0.41 (0.13 to	469140	21.01 (18.88 to	736432	16.55 (13.77 to	-0.81 (-0.92

	to 64845)	2.93)	to 153060)	3.41)	0.69)	(420903 to 517864)	23.18)	(615595 to 851312)	19.21)	to -0.69)
Both	124320 (119604 to 127610)	2.98 (2.87 to 3.06)	303105 (281718 to 318905)	3.56 (3.31 to 3.75)	0.65 (0.33 to 0.96)	1045778 (959373 to 1103849)	24.33 (22.39 to 25.58)	1678836 (1474534 to 1837369)	19.63 (17.25 to 21.5)	-0.67 (-0.82 to -0.53)
SDI region										
Low SDI	1428 (913 to 1817)	0.55 (0.36 to 0.7)	3942 (2372 to 5179)	0.63 (0.38 to 0.81)	0.31 (0.22 to 0.41)	42673 (27239 to 54251)	14.78 (9.49 to 18.73)	100368 (59921 to 134250)	14.6 (8.78 to 19.29)	-0.18 (-0.25 to -0.11)
Low-middle SDI	2027 (1474 to 2539)	0.3 (0.22 to 0.37)	7126 (4900 to 8763)	0.46 (0.32 to 0.56)	1.35 (1.28 to 1.41)	54546 (39683 to 68759)	7.29 (5.36 to 9.23)	140995 (96861 to 179626)	8.55 (5.94 to 10.86)	0.51 (0.48 to 0.55)
Middle SDI	5558 (4234 to 6325)	0.49 (0.38 to 0.56)	22391 (16320 to 26455)	0.84 (0.61 to 0.99)	1.65 (1.59 to 1.71)	128660 (97135 to 148647)	10.39 (7.95 to 12.05)	281237 (209528 to 329491)	10.28 (7.68 to 11.99)	-0.15 (-0.21 to -0.09)
High-middle SDI	21285 (20059 to 22132)	2.09 (1.97 to 2.17)	64796 (57763 to 69921)	3.5 (3.13 to 3.77)	1.85 (1.64 to 2.06)	264663 (242601 to 278416)	25.51 (23.38 to 26.81)	428050 (378978 to 463677)	23.27 (20.55 to 25.25)	-0.32 (-0.47 to -0.16)
High SDI	93902 (91264 to 95914)	8.97 (8.74 to 9.16)	204511 (191891 to 212087)	11.33 (10.8 to 11.7)	0.83 (0.45 to 1.21)	553624 (536873 to 572213)	53.44 (51.9 to 55.21)	725832 (682352 to 769618)	40.99 (38.9 to 43.36)	-0.74 (-0.96 to -0.52)
GBD region										
Andean Latin America	197 (152 to 256)	0.92 (0.71 to 1.19)	889 (679 to 1143)	1.48 (1.13 to 1.89)	1.68 (1.52 to 1.83)	4667 (3556 to 6172)	20.28 (15.39 to 26.71)	12219 (9211 to 15679)	19.99 (15.07 to 25.54)	0.02 (-0.1 to 0.13)
Australasia	6723 (6333 to 7113)	29.48 (27.83 to 31.21)	15279 (13732 to 16829)	32.4 (29.6 to 35.18)	0.5 (-0.01 to 1.01)	44594 (42041 to 47090)	196.05 (184.77 to 207.02)	51338 (46475 to 56880)	109.97 (100.62 to 120.58)	-1.76 (-2.14 to -1.38)
Caribbean	188 (173 to 210)	0.69 (0.63 to 0.77)	593 (519 to 681)	1.11 (0.97 to 1.28)	1.19 (0.95 to 1.43)	3394 (2974 to 4097)	12.12 (10.67 to 14.53)	7360 (6180 to 9020)	13.9 (11.61 to 17.08)	0.18 (-0.03 to 0.4)

Central Asia	541 (496 to 591)	1.11 (1.02 to 1.22)	925 (812 to 1059)	1.12 (0.99 to 1.27)	-0.3 (-0.67 to 0.07)	9956 (9135 to 10884)	19.59 (17.94 to 21.44)	12833 (11102 to 14829)	14.8 (12.91 to 17.05)	-1.29 (-1.55 to -1.03)
Central Europe	5051 (4797 to 5504)	3.51 (3.33 to 3.82)	14588 (13107 to 16008)	7.66 (6.89 to 8.41)	2.76 (2.53 to 2.99)	75340 (71228 to 81350)	52.52 (49.63 to 56.73)	108838 (98914 to 118198)	58.34 (52.78 to 63.49)	0.43 (0.27 to 0.58)
Central Latin America	616 (598 to 633)	0.67 (0.65 to 0.69)	3521 (3139 to 3902)	1.38 (1.23 to 1.53)	2.11 (1.95 to 2.26)	13484 (13145 to 13827)	13.55 (13.18 to 13.91)	46904 (42082 to 51764)	18.18 (16.32 to 20.06)	0.72 (0.62 to 0.83)
Central Sub-Saharan Africa	121 (87 to 186)	0.5 (0.36 to 0.76)	379 (253 to 606)	0.61 (0.4 to 0.95)	0.69 (0.53 to 0.85)	3599 (2565 to 5508)	12.95 (9.34 to 19.85)	9933 (6666 to 16194)	13.86 (9.12 to 21.99)	0.31 (0.2 to 0.42)
East Asia	3396 (2231 to 4276)	0.36 (0.25 to 0.47)	13831 (7571 to 18381)	0.68 (0.37 to 0.9)	2.22 (2.02 to 2.42)	92403 (58182 to 114970)	9.02 (5.86 to 11.31)	160830 (89349 to 212716)	7.9 (4.41 to 10.44)	-0.46 (-0.57 to -0.35)
Eastern Europe	6988 (6679 to 7415)	2.64 (2.52 to 2.8)	19495 (17958 to 21098)	6.27 (5.78 to 6.78)	2.9 (2.68 to 3.12)	99534 (95373 to 105418)	37.68 (36.11 to 39.96)	164701 (150657 to 180397)	53.97 (49.21 to 59.18)	0.99 (0.73 to 1.25)
Eastern Sub-Saharan Africa	886 (583 to 1108)	0.99 (0.66 to 1.23)	2524 (1454 to 3628)	1.13 (0.65 to 1.58)	0.3 (0.22 to 0.38)	27118 (17682 to 34250)	27.02 (17.77 to 33.92)	67197 (38352 to 96675)	27.03 (15.59 to 38.06)	-0.11 (-0.16 to -0.06)
High-income Asia Pacific	1752 (1631 to 1865)	0.88 (0.82 to 0.94)	6108 (5146 to 6843)	1.8 (1.5 to 2)	2.42 (2.05 to 2.8)	13865 (12586 to 14936)	6.91 (6.27 to 7.47)	23623 (20042 to 26423)	6.91 (5.76 to 7.68)	0.03 (-0.16 to 0.21)
High-income North America	56185 (54304 to 57370)	17.1 (16.58 to 17.46)	97139 (91218 to 100891)	16.61 (15.75 to 17.15)	-0.17 (-0.59 to 0.25)	254122 (245111 to 263281)	78.81 (76.1 to 81.65)	292143 (274912 to 310795)	51.06 (48.23 to 54.14)	-1.35 (-1.6 to -1.11)
North Africa and Middle East	1746 (899 to 2483)	0.94 (0.49 to 1.3)	10414 (5662 to 12844)	2.19 (1.16 to 2.7)	2.86 (2.78 to 2.95)	17511 (8480 to 26205)	8.97 (4.47 to 13.12)	37559 (19940 to 45929)	7.6 (3.97 to 9.3)	-0.57 (-0.65 to -0.5)
Oceania	9 (6 to 16)	0.32 (0.22 to	24 (16 to 38)	0.31 (0.22 to	-0.02 (-0.04	271 (183 to	8.05 (5.48 to	691 (469 to	7.92 (5.48 to	0.02 (0 to

		0.57)		0.51)	to 0)	483)	14.27)	1104)	12.68)	0.05)
South Asia	1470 (1032 to 1956)	0.22 (0.15 to 0.29)	5391 (3472 to 7543)	0.33 (0.21 to 0.46)	1.25 (1.1 to 1.4)	42751 (30023 to 57681)	5.78 (4.11 to 7.78)	108848 (72708 to 151823)	6.43 (4.28 to 8.93)	0.25 (0.16 to 0.33)
Southeast Asia	515 (400 to 731)	0.18 (0.15 to 0.26)	1582 (1089 to 2124)	0.24 (0.16 to 0.32)	0.76 (0.74 to 0.79)	15260 (11475 to 21600)	4.88 (3.75 to 6.95)	37794 (26158 to 50051)	5.35 (3.7 to 7.07)	0.23 (0.19 to 0.28)
Southern Latin America	684 (651 to 718)	1.47 (1.4 to 1.55)	2473 (2318 to 2648)	2.99 (2.8 to 3.21)	2.14 (1.73 to 2.54)	12051 (11410 to 12630)	25.75 (24.39 to 26.97)	24482 (23023 to 26192)	29.91 (28.1 to 32)	0.37 (0 to 0.74)
Southern Sub-Saharan Africa	410 (301 to 552)	1.38 (0.98 to 1.84)	1221 (722 to 1556)	1.96 (1.13 to 2.43)	1.27 (1.18 to 1.36)	9819 (7397 to 13488)	30.53 (22.44 to 41.35)	25073 (15225 to 32364)	37.61 (22.57 to 47.44)	0.82 (0.66 to 0.99)
Tropical Latin America	1305 (1261 to 1354)	1.26 (1.21 to 1.31)	4948 (4649 to 5185)	1.92 (1.8 to 2.01)	1.24 (1.09 to 1.39)	29819 (28860 to 30803)	26.99 (26.12 to 27.94)	70160 (66555 to 73107)	26.99 (25.58 to 28.13)	-0.14 (-0.3 to 0.02)
Western Europe	35074 (33846 to 36115)	7.03 (6.82 to 7.23)	100442 (94012 to 105087)	13.71 (13.06 to 14.23)	2.46 (2.06 to 2.86)	262978 (254028 to 270867)	53.37 (51.83 to 54.9)	383503 (357556 to 407460)	52.59 (49.66 to 55.65)	0.18 (-0.03 to 0.39)
Western Sub-Saharan Africa	459 (228 to 625)	0.47 (0.24 to 0.63)	1339 (517 to 1904)	0.55 (0.23 to 0.76)	0.49 (0.41 to 0.57)	13243 (6417 to 18321)	12.26 (6.13 to 16.92)	32806 (12757 to 46377)	12.22 (5.06 to 16.92)	-0.09 (-0.15 to -0.02)

DALYs, disability-adjusted life-years; EAPC, estimated annual percentage change; UI, uncertainty interval; CI, confidence interval.

TABLE S3 Global and regional incidence and DALYs of non-melanoma skin cancer (basal-cell carcinoma) in 1990 and 2021, with age-standardized rates and EAPCs from 1990 to 2021.

	Incidence					DALYs				
	All ages number (95% UI), 1990	Age-standardized rate (95% UI), 1990	All ages number (95% UI), 2021	Age-standardized rate (95% UI), 2021	EAPC (95% CI), 1990-2021	All ages number (95% UI), 1990	Age-standardized rate (95% UI), 1990	All ages number (95% UI), 2021	Age-standardized rate (95% UI), 2021	EAPC (95% CI), 1990-2021
Global										
Male	600858 (492849 to 708907)	36.64 (30.67 to 42.82)	2508840 (2216963 to 2791804)	64.29 (57.09 to 71.37)	2.32 (1.85 to 2.79)	282 (126 to 534)	0.02 (0.01 to 0.03)	1085 (500 to 2038)	0.03 (0.01 to 0.05)	2 (1.59 to 2.42)
Female	595675 (486939 to 701901)	28.47 (23.62 to 33.49)	1928099 (1691675 to 2159950)	42.03 (36.89 to 47)	1.59 (1.22 to 1.96)	308 (136 to 588)	0.01 (0.01 to 0.03)	912 (418 to 1745)	0.02 (0.01 to 0.04)	1.22 (0.92 to 1.52)
Both	1196532 (982434 to 1411452)	31.67 (26.34 to 37.03)	4436939 (3907157 to 4955955)	51.71 (45.7 to 57.58)	2.01 (1.6 to 2.43)	590 (262 to 1122)	0.02 (0.01 to 0.03)	1998 (921 to 3771)	0.02 (0.01 to 0.04)	1.64 (1.29 to 2)
SDI region										
Low SDI	6709 (5045 to 8336)	2.76 (2.06 to 3.42)	15537 (11797 to 19278)	2.74 (2.05 to 3.4)	-0.03 (-0.05 to -0.02)	4 (2 to 7)	0 (0 to 0)	8 (4 to 16)	0 (0 to 0)	-0.06 (-0.07 to -0.05)
Low-middle SDI	30608 (24679 to 36221)	5.11 (4.18 to 6.01)	65897 (50510 to 80878)	4.48 (3.47 to 5.48)	0.18 (0 to 0.37)	16 (7 to 31)	0 (0 to 0.01)	35 (15 to 67)	0 (0 to 0)	0.18 (0.04 to 0.33)
Middle SDI	109629 (90972 to 127076)	10.59 (8.91 to 12.26)	521967 (420005 to 612948)	19.23 (15.66 to 22.48)	1.18 (0.95 to 1.42)	59 (26 to 113)	0.01 (0 to 0.01)	267 (120 to 509)	0.01 (0 to 0.02)	1.14 (0.92 to 1.37)
High-middle	157701	16.33 (14.1 to	526671	27 (22.24 to	0.41 (0.13 to	96 (43 to 188)	0.01 (0 to 0.02)	293 (131 to	0.02 (0.01 to	0.32 (0.06 to

SDI	(134692 to 180963)	18.63)	(432442 to 621733)	31.77)	0.7)			551)	0.03)	0.57)
High SDI	891100 (724348 to 1056397)	81.28 (66.41 to 96.12)	3305587 (2978491 to 3641021)	165.5 (148.64 to 181.49)	3.12 (2.59 to 3.65)	415 (184 to 789)	0.04 (0.02 to 0.07)	1393 (649 to 2619)	0.07 (0.03 to 0.13)	2.75 (2.27 to 3.22)
GBD region										
Andean Latin America	3143 (2836 to 3475)	15.49 (14.01 to 17.11)	6818 (5267 to 8338)	11.49 (8.94 to 14.03)	-1.15 (-1.4 to -0.91)	2 (1 to 4)	0.01 (0 to 0.02)	4 (2 to 8)	0.01 (0 to 0.01)	-1 (-1.22 to -0.79)
Australasia	6724 (5249 to 8147)	29.07 (22.84 to 35.21)	13701 (10829 to 16773)	27.05 (21.34 to 33.05)	-0.12 (-0.2 to -0.05)	4 (2 to 8)	0.02 (0.01 to 0.04)	9 (4 to 16)	0.02 (0.01 to 0.03)	-0.15 (-0.22 to -0.08)
Caribbean	1653 (1272 to 1999)	6.25 (4.82 to 7.6)	2848 (2169 to 3523)	5.3 (4.03 to 6.56)	-0.65 (-0.75 to -0.56)	1 (0 to 2)	0 (0 to 0.01)	2 (1 to 3)	0 (0 to 0.01)	-0.66 (-0.75 to -0.57)
Central Asia	10696 (8294 to 13104)	23.06 (18.06 to 27.89)	18847 (14257 to 23474)	23.19 (17.99 to 28.25)	0.02 (0.01 to 0.03)	6 (3 to 11)	0.01 (0.01 to 0.02)	10 (5 to 20)	0.01 (0.01 to 0.02)	-0.03 (-0.04 to -0.02)
Central Europe	33023 (29078 to 36972)	22.48 (19.93 to 25.14)	55740 (44717 to 68500)	25.46 (20.33 to 30.89)	0.76 (0.54 to 0.97)	19 (9 to 37)	0.01 (0.01 to 0.03)	31 (13 to 60)	0.01 (0.01 to 0.03)	0.63 (0.47 to 0.8)
Central Latin America	23634 (18556 to 28287)	27.99 (22.35 to 33.68)	69423 (54524 to 84156)	27.74 (22.07 to 33.6)	-0.02 (-0.03 to -0.01)	13 (6 to 25)	0.02 (0.01 to 0.03)	38 (17 to 70)	0.01 (0.01 to 0.03)	-0.03 (-0.04 to -0.02)
Central Sub-Saharan Africa	1055 (778 to 1307)	4.52 (3.37 to 5.53)	2684 (1989 to 3331)	4.47 (3.36 to 5.5)	-0.03 (-0.04 to -0.02)	1 (0 to 1)	0 (0 to 0)	1 (1 to 3)	0 (0 to 0)	-0.07 (-0.08 to -0.05)
East Asia	33080 (26282 to 39250)	3.66 (2.99 to 4.31)	667867 (546251 to 780969)	30.5 (25.42 to 35.33)	4.73 (3.93 to 5.53)	22 (10 to 42)	0 (0 to 0)	347 (157 to 661)	0.02 (0.01 to 0.03)	4.26 (3.54 to 4.98)
Eastern Europe	42665 (33802 to 51953)	15.53 (12.44 to 18.81)	63943 (50493 to 79329)	18.41 (14.38 to 22.58)	0.56 (0.51 to 0.61)	25 (11 to 47)	0.01 (0 to 0.02)	36 (16 to 69)	0.01 (0 to 0.02)	0.49 (0.44 to 0.55)

Eastern Sub-Saharan Africa	3115 (2371 to 3820)	3.85 (2.89 to 4.72)	7156 (5476 to 8768)	3.75 (2.8 to 4.64)	-0.11 (-0.13 to -0.09)	2 (1 to 3)	0 (0 to 0)	4 (2 to 7)	0 (0 to 0)	-0.13 (-0.15 to -0.11)
High-income Asia Pacific	5125 (4158 to 6173)	2.64 (2.16 to 3.15)	17689 (14629 to 21302)	3.79 (3.16 to 4.51)	1.08 (0.95 to 1.2)	5 (2 to 9)	0 (0 to 0)	15 (6 to 30)	0 (0 to 0.01)	0.89 (0.77 to 1.01)
High-income North America	726238 (581451 to 867069)	208.67 (166.52 to 248.69)	3001114 (2725845 to 3285357)	473.14 (428.99 to 516.59)	3.65 (3 to 4.31)	314 (138 to 590)	0.09 (0.04 to 0.17)	1210 (571 to 2282)	0.19 (0.09 to 0.36)	3.41 (2.79 to 4.04)
North Africa and Middle East	11930 (9837 to 13958)	7.03 (5.89 to 8.2)	27533 (21040 to 33440)	5.98 (4.64 to 7.3)	-0.46 (-0.85 to -0.07)	8 (3 to 15)	0 (0 to 0.01)	18 (8 to 34)	0 (0 to 0.01)	-0.42 (-0.75 to -0.08)
Oceania	7 (4 to 12)	0.13 (0.07 to 0.22)	16 (9 to 27)	0.13 (0.07 to 0.22)	0.02 (0.01 to 0.04)	0 (0 to 0)	0 (0 to 0)	0 (0 to 0)	0 (0 to 0)	-0.01 (-0.02 to 0)
South Asia	8205 (6105 to 10580)	1.25 (0.93 to 1.58)	21635 (16460 to 27458)	1.36 (1.04 to 1.71)	0.31 (0.26 to 0.36)	5 (2 to 9)	0 (0 to 0)	12 (5 to 24)	0 (0 to 0)	0.31 (0.27 to 0.36)
Southeast Asia	5654 (4823 to 6654)	2.19 (1.87 to 2.55)	9839 (7258 to 12563)	1.5 (1.13 to 1.89)	-1.1 (-1.44 to -0.76)	4 (2 to 7)	0 (0 to 0)	6 (3 to 12)	0 (0 to 0)	-1.06 (-1.38 to -0.74)
Southern Latin America	10282 (8809 to 11655)	22.45 (19.34 to 25.32)	18837 (14899 to 23118)	21.75 (17.06 to 26.6)	-0.08 (-0.19 to 0.02)	6 (3 to 12)	0.01 (0.01 to 0.03)	12 (5 to 22)	0.01 (0.01 to 0.03)	-0.09 (-0.17 to 0)
Southern Sub-Saharan Africa	4540 (3485 to 5516)	15.98 (12.16 to 19.52)	11688 (8866 to 14300)	19.29 (14.59 to 23.53)	0.17 (-0.31 to 0.66)	2 (1 to 4)	0.01 (0 to 0.02)	6 (3 to 11)	0.01 (0 to 0.02)	0.17 (-0.24 to 0.6)
Tropical Latin America	59887 (52069 to 67056)	64.2 (56.17 to 71.8)	98371 (80263 to 116894)	37.82 (30.97 to 44.64)	-0.17 (-0.67 to 0.33)	27 (12 to 52)	0.03 (0.01 to 0.05)	47 (21 to 90)	0.02 (0.01 to 0.03)	-0.13 (-0.55 to 0.3)

Western Europe	203097 (180409 to 228236)	35.96 (31.93 to 40.13)	314622 (249958 to 386879)	35.31 (28.04 to 43.12)	0 (-0.2 to 0.21)	123 (55 to 238)	0.02 (0.01 to 0.04)	184 (78 to 343)	0.02 (0.01 to 0.04)	-0.04 (-0.21 to 0.12)
Western Sub-Saharan Africa	2779 (2109 to 3434)	2.88 (2.15 to 3.56)	6567 (4926 to 8120)	2.86 (2.15 to 3.54)	-0.02 (-0.04 to 0)	2 (1 to 3)	0 (0 to 0)	4 (2 to 7)	0 (0 to 0)	-0.07 (-0.08 to -0.05)

DALYs, disability-adjusted life-years; EAPC, estimated annual percentage change; UI, uncertainty interval; CI, confidence interval.

TABLE S4 Global and regional incidence and DALYs of non-melanoma skin cancer (squamous-cell carcinoma) in 1990 and 2021, with age-standardized rates and EAPCs from 1990 to 2021.

	Incidence					DALYs				
	All ages number (95% UI), 1990	Age-standardized rate (95% UI), 1990	All ages number (95% UI), 2021	Age-standardized rate (95% UI), 2021	EAPC (95% CI), 1990-2021	All ages number (95% UI), 1990	Age-standardized rate (95% UI), 1990	All ages number (95% UI), 2021	Age-standardized rate (95% UI), 2021	EAPC (95% CI), 1990-2021
Global										
Male	273134 (213821 to 351037)	18.65 (14.66 to 24.22)	1187477 (1051962 to 1348915)	31.53 (27.96 to 35.7)	2.1 (1.66 to 2.55)	323197 (281190 to 364928)	18.25 (16.17 to 20.55)	716679 (594142 to 807397)	18.58 (15.55 to 20.84)	0.26 (0.18 to 0.35)
Female	191978 (152105 to 246185)	9.76 (7.73 to 12.6)	712430 (636435 to 800222)	15.3 (13.67 to 17.17)	1.83 (1.35 to 2.32)	221777 (201297 to 246805)	10.61 (9.68 to 11.75)	494196 (432595 to 550340)	10.8 (9.47 to 12.02)	0.18 (0.1 to 0.26)
Both	465112 (366708 to	13.38 (10.55 to 17.26)	1899907 (1688003 to	22.38 (19.9 to 25.27)	2.06 (1.6 to 2.52)	544974 (496193 to	14.01 (12.79 to 15.37)	1210875 (1068481 to	14.31 (12.65 to 15.78)	0.24 (0.16 to 0.31)

	594410)		2150030)			601306)		1334386)		
SDI region										
Low SDI	299 (224 to 409)	0.12 (0.09 to 0.16)	735 (552 to 994)	0.13 (0.1 to 0.17)	0.27 (0.23 to 0.3)	11862 (6769 to 15720)	5.19 (2.96 to 6.93)	36640 (18394 to 49413)	7 (3.6 to 9.43)	1.1 (1.02 to 1.17)
Low-middle SDI	1920 (1495 to 2491)	0.32 (0.25 to 0.41)	4410 (3365 to 5858)	0.31 (0.24 to 0.4)	0.18 (0.1 to 0.27)	48727 (39726 to 62556)	7.91 (6.28 to 9.82)	148327 (123337 to 171408)	10.54 (8.84 to 12.24)	1.04 (0.98 to 1.1)
Middle SDI	10490 (8536 to 13010)	1.04 (0.84 to 1.3)	75747 (57953 to 97928)	2.88 (2.21 to 3.71)	1.7 (1.32 to 2.08)	167372 (141798 to 188831)	15.78 (13.44 to 17.7)	427281 (349907 to 489019)	16.43 (13.54 to 18.74)	0.29 (0.18 to 0.4)
High-middle SDI	22122 (18379 to 27459)	2.38 (2 to 2.95)	95207 (74809 to 123223)	4.82 (3.82 to 6.18)	0.87 (0.52 to 1.22)	152906 (142025 to 171619)	16.19 (15.05 to 18.03)	290190 (257531 to 340443)	15.21 (13.5 to 17.83)	-0.12 (-0.24 to 0)
High SDI	430174 (337646 to 551374)	38.69 (30.74 to 49.38)	1723620 (1540394 to 1925270)	79.99 (71.47 to 89.41)	2.91 (2.41 to 3.4)	163225 (154257 to 173467)	15.18 (14.36 to 16.1)	307269 (277067 to 344982)	14.62 (13.26 to 16.34)	0.17 (0.04 to 0.31)
GBD region										
Andean Latin America	80 (65 to 100)	0.39 (0.32 to 0.49)	118 (89 to 159)	0.2 (0.15 to 0.27)	-2.64 (-3 to -2.27)	2453 (1998 to 3071)	11.69 (9.55 to 14.51)	12124 (9342 to 14554)	20.47 (15.74 to 24.57)	1.79 (1.53 to 2.05)
Australasia	19230 (15228 to 24000)	84.18 (67.13 to 104.3)	35645 (27647 to 45328)	68.31 (52.67 to 86.46)	-1.15 (-1.37 to -0.92)	7330 (6854 to 7910)	31.8 (29.73 to 34.39)	17120 (15437 to 18488)	31.68 (28.78 to 34.2)	-0.13 (-0.31 to 0.05)
Caribbean	184 (152 to 221)	0.69 (0.57 to 0.83)	194 (152 to 248)	0.36 (0.29 to 0.46)	-2.31 (-2.45 to -2.17)	4607 (4337 to 5241)	18.05 (16.95 to 20.55)	14219 (12282 to 15955)	26.25 (22.67 to 29.45)	1.35 (1.1 to 1.6)
Central Asia	33 (22 to 49)	0.06 (0.04 to 0.09)	54 (35 to 80)	0.06 (0.04 to 0.09)	-0.17 (-0.24 to -0.1)	4252 (3526 to 4889)	9.1 (7.43 to 10.56)	16092 (14360 to 17900)	20.81 (18.62 to 23.12)	3.3 (3.01 to 3.58)
Central	3893 (3335 to	2.7 (2.33 to 3.24)	6580 (5172 to	2.87 (2.29 to	0.52 (0.37 to	36771 (35028	27.05 (25.66 to	25862 (23558	11.77 (10.72 to	-2.94 (-3.18

Europe	4675)		8643)	3.74)	0.66)	to 38103)	28.16)	to 28447)	12.95)	to -2.69)
Central Latin America	1114 (884 to 1410)	1.31 (1.04 to 1.66)	3128 (2417 to 4021)	1.24 (0.96 to 1.6)	-0.21 (-0.22 to -0.19)	22823 (22128 to 23406)	26.4 (25.38 to 27.14)	45670 (40398 to 51637)	18.4 (16.3 to 20.76)	-1.13 (-1.28 to -0.99)
Central Sub-Saharan Africa	14 (9 to 21)	0.05 (0.04 to 0.07)	37 (22 to 56)	0.05 (0.04 to 0.07)	-0.01 (-0.03 to 0.01)	1437 (538 to 2137)	6.59 (2.44 to 9.91)	5254 (1529 to 8178)	9.39 (2.83 to 14.69)	1.28 (1.17 to 1.39)
East Asia	6935 (5608 to 8738)	0.87 (0.71 to 1.1)	124400 (95292 to 160050)	5.75 (4.46 to 7.4)	3.73 (2.95 to 4.51)	144758 (119404 to 179521)	16.26 (13.56 to 20.06)	369643 (295817 to 458238)	17.77 (14.25 to 21.95)	0.61 (0.4 to 0.81)
Eastern Europe	5351 (4199 to 6825)	1.99 (1.58 to 2.52)	8070 (6175 to 10625)	2.29 (1.77 to 2.98)	0.52 (0.48 to 0.57)	42175 (39796 to 43947)	16.05 (15.11 to 16.74)	52754 (48286 to 57232)	15.73 (14.39 to 17.09)	-0.35 (-0.76 to 0.06)
Eastern Sub-Saharan Africa	108 (82 to 148)	0.12 (0.09 to 0.16)	275 (207 to 372)	0.13 (0.1 to 0.17)	0.09 (0.08 to 0.11)	4710 (1589 to 7062)	6.34 (2.15 to 9.63)	14917 (4202 to 22976)	8.58 (2.44 to 13.25)	1 (0.98 to 1.03)
High-income Asia Pacific	824 (634 to 1058)	0.43 (0.34 to 0.55)	3142 (2411 to 4233)	0.62 (0.48 to 0.81)	1.1 (1.01 to 1.2)	13819 (11926 to 14727)	7.28 (6.29 to 7.78)	25664 (21691 to 30971)	5.26 (4.68 to 6.47)	-1.06 (-1.18 to -0.93)
High-income North America	386240 (298182 to 498939)	106.75 (83.4 to 137.18)	1632871 (1462866 to 1817291)	240.41 (216.04 to 267.46)	3.3 (2.71 to 3.88)	77955 (71754 to 86032)	23.02 (21.31 to 25.27)	174027 (148334 to 206210)	26.7 (22.9 to 31.53)	0.96 (0.74 to 1.18)
North Africa and Middle East	1250 (1028 to 1536)	0.79 (0.64 to 0.98)	3173 (2501 to 4080)	0.74 (0.59 to 0.96)	-0.4 (-0.8 to 0.01)	7335 (5876 to 10632)	4.53 (3.52 to 6.31)	21127 (17553 to 27372)	4.9 (4.06 to 6.36)	0.74 (0.49 to 0.99)
Oceania	1 (0 to 1)	0.02 (0.01 to 0.03)	2 (1 to 3)	0.02 (0.01 to 0.03)	0 (-0.01 to 0.01)	298 (195 to 424)	10.53 (7.19 to 14.48)	970 (612 to 1381)	12.34 (8.16 to 17.15)	0.58 (0.53 to 0.63)
South Asia	932 (670 to 1306)	0.15 (0.11 to 0.2)	2358 (1725 to 3244)	0.16 (0.12 to 0.21)	0.2 (0.16 to 0.25)	38856 (28464 to 54077)	6.78 (4.75 to 9.17)	113381 (93024 to 150026)	7.9 (6.44 to 10.49)	0.49 (0.4 to 0.59)

Southeast Asia	606 (505 to 738)	0.24 (0.2 to 0.29)	1416 (1110 to 1849)	0.23 (0.18 to 0.29)	-0.1 (-0.34 to 0.14)	36711 (28260 to 44983)	13.15 (10.15 to 16.01)	96394 (75243 to 115537)	14.4 (11.41 to 17.2)	0.14 (0.03 to 0.25)
Southern Latin America	1384 (1131 to 1756)	3.07 (2.52 to 3.87)	2805 (2149 to 3682)	3.16 (2.44 to 4.12)	0.09 (-0.01 to 0.19)	6563 (6192 to 6955)	14.68 (13.84 to 15.59)	13982 (12986 to 14921)	16.05 (14.96 to 17.08)	0.55 (0.34 to 0.76)
Southern Sub-Saharan Africa	1568 (1245 to 1974)	5.66 (4.45 to 7.2)	4873 (3801 to 6249)	8.47 (6.62 to 10.86)	0.37 (-0.25 to 0.99)	2886 (2223 to 4363)	10.79 (8.19 to 16.1)	9407 (7251 to 11110)	16.74 (12.9 to 19.5)	1.79 (1.61 to 1.98)
Tropical Latin America	2941 (2427 to 3531)	3.19 (2.64 to 3.89)	7886 (6313 to 9706)	3.07 (2.46 to 3.81)	0.75 (0.55 to 0.95)	19195 (18443 to 19831)	20.33 (19.41 to 21.11)	64407 (59736 to 67605)	25.29 (23.38 to 26.58)	0.73 (0.59 to 0.86)
Western Europe	32364 (27630 to 38397)	5.54 (4.8 to 6.56)	62720 (48941 to 82500)	6.56 (5.13 to 8.55)	0.62 (0.48 to 0.77)	67834 (64363 to 69974)	12.3 (11.7 to 12.68)	107986 (96328 to 115184)	11.11 (10.15 to 11.71)	0 (-0.17 to 0.18)
Western Sub-Saharan Africa	60 (42 to 86)	0.05 (0.03 to 0.07)	161 (111 to 227)	0.05 (0.04 to 0.07)	0.26 (0.2 to 0.33)	2206 (1121 to 2888)	2.09 (1.11 to 2.7)	9873 (2929 to 14712)	3.71 (1.18 to 5.37)	2.21 (1.94 to 2.49)

DALYs, disability-adjusted life-years; EAPC, estimated annual percentage change; UI, uncertainty interval; CI, confidence interval.

TABLE S5 Global incidence and DALYs of total skin cancer from 1990 to 2021, by sex, with age-standardized rates and 95% uncertainty intervals.

Year	Incidence				DALYs			
	Male		Female		Male		Female	
	All ages number (95%	Age-standardized	All ages number (95%	Age-standardized	All ages number (95%	Age-standardized	All ages number (95%	Age-standardized

	UI)	rate (95% UI)	UI)	rate (95% UI)	UI)	rate (95% UI)	UI)	rate (95% UI)
1990	935684.38 (765959.39 to 1123409.75)	58.51 (48.41 to 70.34)	850279.42 (698723.96 to 1012930.57)	41.05 (34.04 to 49.02)	900117.08 (798500.23 to 982623.3)	46.33 (41.47 to 50.54)	691224.43 (622336.74 to 765256.87)	31.64 (28.57 to 34.96)
1991	964680.26 (789880.94 to 1158063.98)	58.84 (48.66 to 70.85)	877858.86 (721760.57 to 1046877.1)	41.4 (34.28 to 49.49)	920623.49 (818504.19 to 1001800.67)	46.26 (41.49 to 50.4)	702592.64 (630250 to 776145.61)	31.45 (28.4 to 34.66)
1992	995087.32 (815307.72 to 1193212.05)	59.17 (48.93 to 71.31)	906090.99 (746172.02 to 1080774.95)	41.74 (34.55 to 49.94)	948162.41 (838014.2 to 1038715.68)	46.6 (41.55 to 50.83)	721983.16 (652627.3 to 799028.45)	31.62 (28.64 to 34.91)
1993	1026220.34 (841284.58 to 1229604.64)	59.5 (49.23 to 71.72)	933646.04 (770487.45 to 1113381.03)	42.03 (34.79 to 50.28)	977300.72 (860506.11 to 1065898.86)	46.95 (41.68 to 51.23)	746202.3 (673582.23 to 822407.87)	31.97 (28.94 to 35.18)
1994	1055348.7 (866084.12 to 1263449.53)	59.66 (49.41 to 71.9)	958396.1 (792443.97 to 1142605.97)	42.16 (34.91 to 50.4)	999771.42 (887102.01 to 1088797.14)	47.04 (42.07 to 51.02)	770522.19 (696453.71 to 843848.56)	32.32 (29.26 to 35.32)
1995	1085276.11 (892804.9 to 1298633.92)	59.82 (49.62 to 72.02)	981502.31 (812616.8 to 1168843.96)	42.2 (34.99 to 50.38)	1022222.39 (909506.74 to 1117466.93)	47.12 (42.22 to 51.48)	787977 (713833.86 to 869286.88)	32.38 (29.38 to 35.55)
1996	1118603.32 (924818.8 to 1333998.08)	60.1 (49.97 to 71.99)	1002661.06 (833624.94 to 1187626.43)	42.1 (34.99 to 49.95)	1035501.68 (917448.44 to 1132494.09)	46.78 (41.9 to 50.91)	801930.25 (725588.31 to 878085.25)	32.26 (29.23 to 35.32)
1997	1162897.91 (967739.07 to 1378716.27)	60.84 (50.8 to 72.39)	1022491.96 (852397.96 to 1204180.62)	41.91 (34.94 to 49.35)	1052830.1 (934926.95 to 1153538.43)	46.56 (41.71 to 50.92)	809852.53 (731360.26 to 886596.12)	31.89 (28.79 to 34.82)
1998	1217685.68 (1018918.09 to 1435182.39)	62.05 (52 to 73.14)	1043224.42 (873488.86 to 1221323.7)	41.72 (34.89 to 48.89)	1076221.42 (953770.3 to 1184039.04)	46.64 (41.75 to 51.25)	823441.21 (739535.59 to 904027.71)	31.72 (28.55 to 34.71)
1999	1276935.42 (1074251.32 to 1496316.55)	63.34 (53.32 to 74.26)	1065985.08 (897038.23 to 1243949.52)	41.58 (34.98 to 48.44)	1099988.43 (979348.51 to 1203859.67)	46.59 (41.74 to 50.9)	844053.32 (760068.9 to 926115.06)	31.79 (28.69 to 34.74)

2000	1343208.28 (1134381.87 to 1568031.79)	64.89 (54.78 to 75.58)	1093801 (924191.72 to 1271966.51)	41.61 (35.19 to 48.36)	1130010.27 (1006131.27 to 1230444.12)	46.88 (42.01 to 51.03)	867863.1 (783273.26 to 946753.1)	31.96 (28.83 to 34.72)
2001	1478371.54 (1265388.69 to 1702926.12)	69.58 (59.97 to 80.14)	1181877.85 (1013887.99 to 1353423.14)	43.86 (37.65 to 50.28)	1168097.98 (1036745.46 to 1267719.41)	47.44 (42.43 to 51.48)	887221.04 (797288.83 to 966529.5)	31.94 (28.73 to 34.74)
2002	1709356.22 (1486271.06 to 1941465.2)	78.44 (68.93 to 89.25)	1353692.69 (1179623.34 to 1527162.64)	49.04 (42.81 to 55.43)	1195813.92 (1064976.12 to 1302599.21)	47.52 (42.51 to 51.67)	902973.33 (809858.38 to 986877.84)	31.76 (28.46 to 34.65)
2003	1979921.84 (1744564.12 to 2215749.85)	88.59 (78.93 to 99.4)	1560708.73 (1376903.94 to 1743489.27)	55.2 (48.78 to 61.72)	1226070.42 (1088585.01 to 1334840.46)	47.69 (42.65 to 51.79)	921834.34 (828792.98 to 1006457.83)	31.68 (28.48 to 34.53)
2004	2220749.85 (1977606.72 to 2465033.59)	96.79 (86.76 to 107.53)	1745660.51 (1553346.4 to 1934304.18)	60.23 (53.61 to 66.67)	1253263.29 (1109904.49 to 1361661.56)	47.68 (42.47 to 51.73)	936879.15 (839437.11 to 1031634.93)	31.44 (28.23 to 34.58)
2005	2368366.83 (2115905.16 to 2623259.39)	100.44 (90.55 to 111.02)	1854808.88 (1659738.1 to 2041689.86)	62.38 (55.85 to 68.78)	1290991.62 (1144458.95 to 1408559.94)	48 (42.7 to 52.21)	961518.42 (866269.25 to 1056368.36)	31.5 (28.39 to 34.52)
2006	2437557.3 (2182232.58 to 2696491.89)	100.34 (90.54 to 110.77)	1900126.68 (1702257.82 to 2090332.03)	62.22 (55.76 to 68.59)	1319403.88 (1172091.2 to 1437322.61)	47.89 (42.74 to 52.02)	976771.69 (875715.32 to 1065450.73)	31.23 (28.02 to 34.04)
2007	2511014.52 (2253349.35 to 2772407.11)	100.21 (90.46 to 110.47)	1947564.06 (1746668.89 to 2142927.44)	62.07 (55.73 to 68.44)	1352914.43 (1199236.84 to 1474015.35)	47.9 (42.69 to 52.06)	1000097.08 (898778.1 to 1088106.78)	31.19 (28.01 to 33.89)
2008	2588665.45 (2326419.79 to	100.18 (90.53 to 110.37)	1995273.69 (1790451.28 to 2195659.88)	61.89 (55.61 to 68.23)	1396543.32 (1228333.9 to 1515523.6)	48.21 (42.61 to 52.22)	1022870.37 (919451.15 to 1113871.41)	31.12 (27.98 to 33.86)

	2855427.97)							
2009	2662088.84 (2396353.07 to 2931777.35)	99.84 (90.29 to 109.94)	2041436.13 (1834739.32 to 2248906.04)	61.6 (55.38 to 67.86)	1429608.76 (1254874.3 to 1551042.43)	48.15 (42.4 to 52.08)	1051615.08 (941985.03 to 1145330.77)	31.2 (27.93 to 33.93)
2010	2737167.61 (2465999.75 to 3013981.67)	99.59 (90.02 to 109.61)	2086143.84 (1875462.41 to 2298275.27)	61.25 (55.13 to 67.49)	1459336.62 (1274721.11 to 1588375.11)	47.96 (41.97 to 51.99)	1075068.36 (952410.64 to 1169388.45)	31.11 (27.56 to 33.81)
2011	2811516.06 (2530518.91 to 3096610.4)	99.17 (89.55 to 109.18)	2129482.21 (1913152.76 to 2344862.97)	60.8 (54.66 to 66.99)	1483791.99 (1291081.53 to 1617440.8)	47.63 (41.64 to 51.72)	1094341.8 (966591.63 to 1188125.52)	30.88 (27.28 to 33.51)
2012	2895296.36 (2604892.18 to 3192016.49)	98.87 (89.23 to 108.88)	2177840.74 (1955711.4 to 2397919.27)	60.45 (54.31 to 66.62)	1504279.75 (1297970.27 to 1635804.62)	47.12 (40.9 to 51.16)	1110537.53 (979183.92 to 1206070.4)	30.57 (26.95 to 33.19)
2013	2986156.59 (2686186.85 to 3295064.61)	98.77 (89.07 to 108.86)	2229755.61 (2001309.7 to 2454830.14)	60.19 (54.04 to 66.3)	1532483.46 (1320687.69 to 1690953.45)	46.83 (40.56 to 51.57)	1126605.94 (987643.07 to 1224754.1)	30.26 (26.52 to 32.89)
2014	3074354.8 (2763839.85 to 3393269.11)	98.5 (88.76 to 108.66)	2282087.58 (2044652.82 to 2512822.26)	59.89 (53.7 to 66.01)	1566090.15 (1353432.99 to 1725516.35)	46.68 (40.6 to 51.36)	1146488.87 (1009595 to 1255725.67)	30.04 (26.45 to 32.92)
2015	3161408.42 (2837901.16 to 3492607.89)	98.29 (88.54 to 108.58)	2332255.33 (2086575.72 to 2571176.86)	59.56 (53.32 to 65.71)	1582265.63 (1364975.65 to 1735930.76)	46.04 (39.97 to 50.46)	1159619.5 (1010500.92 to 1277499.93)	29.66 (25.84 to 32.68)
2016	3235201.26 (2901763.01 to 3576588.27)	97.46 (87.65 to 107.71)	2373769.54 (2120508.61 to 2621306.33)	58.94 (52.69 to 65.13)	1595203.64 (1370543.27 to 1773466.34)	45.29 (39.13 to 50.17)	1163896.27 (1008824.4 to 1284887.62)	29.05 (25.16 to 32.09)
201	3308400.49	96.52 (86.65 to	2417318.03 (2156812.82	58.37 (52.1 to	1597255.29 (1370927.43	44.21 (38.17 to	1177389.65 (1016417.92	28.68 (24.74 to

7	(2961856.37 to 3662798.2)	106.76)	to 2676603.05)	64.65)	to 1775926.13)	49.02)	to 1304967.13)	31.82)
2018	3390526.99 (3029136.92 to 3761573.46)	95.83 (85.79 to 106.2)	2464035.18 (2194672.77 to 2735308.29)	57.86 (51.56 to 64.22)	1615543.68 (1379570.2 to 1805146.95)	43.61 (37.49 to 48.63)	1189909.64 (1025141.04 to 1328049.07)	28.29 (24.33 to 31.64)
2019	3481464.76 (3105342.53 to 3866804.18)	95.27 (85.13 to 105.81)	2518889.23 (2239256.69 to 2803894.28)	57.51 (51.15 to 63.98)	1634438 (1399128.58 to 1841421.28)	43 (36.99 to 48.3)	1205708.65 (1031552.08 to 1360253.31)	27.99 (23.9 to 31.64)
2020	3586177.31 (3193892.3 to 3989503.11)	95.42 (85.13 to 106.03)	2579968.48 (2294471.75 to 2872781.35)	57.41 (51.06 to 63.86)	1637904.13 (1399271.25 to 1840489.26)	42.13 (36.2 to 47.21)	1208973.24 (1038661.7 to 1361414.92)	27.43 (23.52 to 30.96)
2021	3857633.49 (3419814.56 to 4311761.69)	99.92 (88.87 to 111.43)	2782317.21 (2458444.68 to 3113231.76)	60.49 (53.47 to 67.58)	1660168.48 (1416740.37 to 1866299.56)	41.84 (35.88 to 46.9)	1231540.22 (1048607.6 to 1403397.63)	27.37 (23.25 to 31.27)

Total skin cancer includes malignant skin melanoma, non-melanoma skin cancer (basal-cell carcinoma) and non-melanoma skin cancer (squamous-cell carcinoma). DALYs, disability-adjusted life-years; UI, uncertainty interval.

TABLE S6 Global incidence and DALYs of malignant skin melanoma from 1990 to 2021, by sex, with age-standardized rates and 95% uncertainty intervals.

Year	Incidence				DALYs			
	Male		Female		Male		Female	
	All ages number (95% UI)	Age-standardized rate (95% UI)	All ages number (95% UI)	Age-standardized rate (95% UI)	All ages number (95% UI)	Age-standardized rate (95% UI)	All ages number (95% UI)	Age-standardized rate (95% UI)

1990	61693.14 (59289.78 to 63465.52)	3.21 (3.08 to 3.3)	62626.7 (59679.93 to 64845.27)	2.83 (2.69 to 2.93)	576637.91 (517184.34 to 617161.44)	28.07 (25.29 to 29.95)	469139.63 (420903.45 to 517864.33)	21.01 (18.88 to 23.18)
1991	64708.82 (62187.5 to 66420.59)	3.29 (3.16 to 3.39)	64289.12 (61228.24 to 66484.49)	2.84 (2.7 to 2.94)	591664.35 (534038.14 to 631165.84)	28.16 (25.49 to 29.99)	475773.05 (424638.54 to 522873.73)	20.86 (18.73 to 22.88)
1992	68113.5 (65471.61 to 70081.5)	3.4 (3.26 to 3.5)	67398.66 (64179.53 to 69846.5)	2.91 (2.77 to 3.02)	611159.46 (548793.26 to 655015.34)	28.48 (25.69 to 30.37)	487825.47 (437737.53 to 541492.13)	20.93 (18.85 to 23.2)
1993	72287.13 (69475.31 to 74252.75)	3.53 (3.39 to 3.63)	71435.3 (67993.86 to 73795.03)	3.02 (2.87 to 3.12)	633289.47 (565643.73 to 676230.69)	28.9 (25.97 to 30.79)	506140.34 (453779.29 to 558014.09)	21.27 (19.14 to 23.45)
1994	76700.7 (73632.31 to 78745.29)	3.67 (3.52 to 3.78)	75711.38 (72075.4 to 78270.99)	3.14 (2.98 to 3.24)	650418.41 (583689.03 to 693813.7)	29.11 (26.25 to 30.93)	524203.64 (470238.27 to 576621.86)	21.58 (19.44 to 23.71)
1995	82427.89 (79081.59 to 84633.44)	3.86 (3.71 to 3.97)	80238 (76352.05 to 82892.44)	3.25 (3.09 to 3.36)	669557.61 (600953.43 to 714212.27)	29.38 (26.5 to 31.25)	534284.86 (478754.87 to 593594.91)	21.55 (19.37 to 23.83)
1996	86916.81 (83402.95 to 89233.96)	4 (3.84 to 4.11)	84892.97 (80854.13 to 87569.84)	3.37 (3.21 to 3.48)	678429.19 (607432.36 to 726979.19)	29.21 (26.35 to 31.11)	543068.95 (486171.15 to 597277.66)	21.46 (19.28 to 23.65)
1997	90291.74 (86757.22 to 92657.23)	4.07 (3.91 to 4.18)	87453.12 (83190.72 to 90296.73)	3.4 (3.23 to 3.52)	688021.76 (617768.69 to 735938.61)	29.01 (26.25 to 30.95)	548002.51 (489609.98 to 601595.84)	21.22 (19 to 23.26)
1998	94107.02 (90581.96 to 96521.83)	4.15 (3.99 to 4.27)	89257.19 (84606.69 to 92335.27)	3.4 (3.22 to 3.52)	701963.18 (629199.12 to 749960.72)	29.03 (26.21 to 30.98)	553829.62 (491114.72 to 613219.39)	20.99 (18.68 to 23.17)
1999	96591.98 (92997.68 to 99141.36)	4.17 (4 to 4.28)	91585.04 (86875.06 to 94661.53)	3.41 (3.24 to 3.53)	714549.38 (643341.91 to 762974.41)	28.92 (26.15 to 30.82)	564722.16 (501720.97 to 624184.42)	20.94 (18.63 to 23.06)
2000	99803.8 (96032.78 to 102466.67)	4.22 (4.05 to 4.34)	94951.89 (90087.86 to 98413.98)	3.46 (3.28 to 3.59)	730726.67 (659905.55 to 781893.51)	28.98 (26.29 to 31)	579957.19 (517175.16 to 635192.12)	21.03 (18.79 to 22.93)
2001	103966.64 (99941.3 to 106699.57)	4.29 (4.13 to 4.42)	97798.55 (92817.01 to 101265.27)	3.49 (3.31 to 3.62)	748839 (676056.18 to 801847.24)	29.07 (26.42 to 31.1)	591644.3 (525239.3 to 646029.7)	20.99 (18.66 to 22.93)
2002	106995.56 (102947.03 to 109898.32)	4.32 (4.15 to 4.45)	99251.55 (94275.35 to 102722.54)	3.46 (3.28 to 3.59)	760240.79 (688181.62 to 817777.97)	28.87 (26.2 to 30.94)	595570.98 (528386.73 to 653973.39)	20.65 (18.36 to 22.64)

2003	111228.02 (107091.8 to 114439.97)	4.4 (4.23 to 4.53)	102160.75 (97023.43 to 105857.6)	3.48 (3.3 to 3.62)	777332.27 (703932.04 to 838391.55)	28.89 (26.3 to 31.05)	605306.62 (538493.34 to 664658.07)	20.53 (18.3 to 22.52)
2004	115649.97 (111022.45 to 119055.52)	4.47 (4.29 to 4.61)	105608 (100335.64 to 109724.01)	3.52 (3.34 to 3.66)	790358.58 (718069.89 to 855023.67)	28.74 (26.18 to 31.02)	611953.71 (543493.94 to 675668.63)	20.27 (18.05 to 22.37)
2005	122445.4 (117627.14 to 126164.91)	4.63 (4.44 to 4.78)	111553.98 (105782.19 to 116058.03)	3.63 (3.44 to 3.79)	814741.1 (742895.61 to 885170.24)	29 (26.53 to 31.34)	627279.66 (559030.14 to 697153.81)	20.31 (18.15 to 22.51)
2006	128218.08 (123092.56 to 132337.29)	4.74 (4.54 to 4.9)	115506.9 (109143.25 to 120466.66)	3.67 (3.47 to 3.83)	832249.95 (758919.78 to 903484.85)	28.96 (26.51 to 31.27)	634824.81 (564214.17 to 698564.26)	20.07 (17.89 to 22.07)
2007	133650.82 (128419.66 to 137882.68)	4.82 (4.6 to 4.97)	119978.17 (113207.81 to 125159.48)	3.72 (3.51 to 3.89)	851955.93 (776510.91 to 923240.7)	28.96 (26.53 to 31.27)	646769.47 (575914.22 to 708820.19)	19.97 (17.81 to 21.85)
2008	140026.94 (134107.82 to 144273.72)	4.92 (4.69 to 5.08)	123941.13 (116820.49 to 129017.32)	3.75 (3.54 to 3.91)	877719.68 (798171.58 to 952023.66)	29.13 (26.59 to 31.48)	658621.34 (587245.1 to 723238.13)	19.86 (17.74 to 21.78)
2009	145747.73 (139915.02 to 150038.5)	4.99 (4.77 to 5.15)	128846.06 (121044.83 to 134346.29)	3.81 (3.58 to 3.97)	894751.8 (810278.09 to 967694.31)	29.01 (26.35 to 31.23)	671660.99 (594417.53 to 738325.15)	19.77 (17.52 to 21.69)
2010	149102.85 (142446.68 to 153728.31)	4.98 (4.74 to 5.15)	131562.74 (123545.95 to 137294.87)	3.79 (3.56 to 3.96)	906823.12 (818269.44 to 987260.58)	28.73 (26.02 to 31.14)	682103.38 (600584.4 to 747929.93)	19.61 (17.29 to 21.47)
2011	152321.42 (145382.34 to 157506.19)	4.97 (4.73 to 5.14)	133354.42 (125265.99 to 139405.03)	3.75 (3.52 to 3.93)	916484.59 (825229.41 to 995279.43)	28.41 (25.71 to 30.69)	688546.95 (602760.25 to 750288.35)	19.33 (16.93 to 21.04)
2012	153881.08 (146592.52 to 159104.84)	4.9 (4.64 to 5.09)	134505.01 (125778.04 to 141034.01)	3.7 (3.45 to 3.88)	921291.15 (824600.24 to 998237.3)	27.9 (25.12 to 30.18)	692823.94 (605026.88 to 753649.01)	19 (16.6 to 20.65)
2013	156971.83 (149355.19 to 162802.61)	4.87 (4.59 to 5.06)	136221.69 (127247.54 to 142827.02)	3.65 (3.41 to 3.83)	932643.36 (832771.9 to 1019015.77)	27.57 (24.71 to 30.05)	698648.2 (606514.46 to 762159.96)	18.71 (16.25 to 20.42)
2014	160444.33 (152304.98 to 166807.76)	4.85 (4.57 to 5.05)	139430.77 (129786.84 to 146342.26)	3.65 (3.4 to 3.83)	948740.58 (847437.25 to 1037170.05)	27.38 (24.56 to 29.86)	710263.17 (615712.02 to 779080.09)	18.59 (16.12 to 20.4)
2015	160006.93 (152087.7 to 166684.26)	4.72 (4.45 to 4.92)	139616.1 (129864 to 147142.06)	3.57 (3.32 to 3.76)	947104.16 (846709.46 to 1037868.85)	26.72 (23.93 to 29.2)	714545.28 (619112.27 to 789442.46)	18.28 (15.83 to 20.21)

2016	158147.85 (149902.43 to 165139.71)	4.54 (4.28 to 4.75)	137721.07 (127294.72 to 145328.25)	3.44 (3.18 to 3.63)	940190.04 (832595.01 to 1036194.45)	25.89 (23.04 to 28.4)	709044.9 (609474.27 to 784774.34)	17.73 (15.22 to 19.66)
2017	156448.62 (147847.88 to 163903.37)	4.38 (4.13 to 4.59)	138935.39 (128557.67 to 147147.63)	3.39 (3.13 to 3.59)	929976.22 (821493.71 to 1030156.15)	25.01 (22.22 to 27.63)	716713.44 (609450.96 to 795301.55)	17.53 (14.88 to 19.48)
2018	158135.35 (149262.83 to 165755.15)	4.31 (4.05 to 4.52)	139582.74 (128565.04 to 148433.88)	3.33 (3.07 to 3.53)	938316.81 (823893.11 to 1040830.17)	24.67 (21.81 to 27.32)	721113.77 (613230.38 to 810253.52)	17.25 (14.62 to 19.43)
2019	159704.54 (150400.26 to 167883.17)	4.24 (3.98 to 4.46)	140549.82 (129293.34 to 150734.32)	3.27 (3.01 to 3.51)	943018.59 (827528.77 to 1062385.08)	24.21 (21.33 to 27.2)	725946.68 (613299.12 to 829018.84)	16.98 (14.3 to 19.45)
2020	158716.9 (149015.86 to 167685)	4.12 (3.86 to 4.36)	139124.45 (127698.53 to 148846.38)	3.17 (2.91 to 3.39)	934914.85 (819335.65 to 1044940.06)	23.49 (20.64 to 26.21)	723506.44 (610983.71 to 819524.68)	16.58 (13.94 to 18.83)
2021	161315.86 (150889.89 to 171042.66)	4.1 (3.82 to 4.36)	141788.75 (130334.77 to 153059.96)	3.16 (2.9 to 3.41)	942404.39 (822097.6 to 1056864.5)	23.23 (20.33 to 26)	736431.92 (615594.67 to 851312.29)	16.55 (13.77 to 19.21)

DALYs, disability-adjusted life-years; UI, uncertainty interval.

TABLE S7 Global incidence and DALYs of non-melanoma skin cancer (basal-cell carcinoma) from 1990 to 2021, by sex, with age-standardized rates and 95% uncertainty intervals.

Year	Incidence				DALYs			
	Male		Female		Male		Female	
	All ages number (95% UI)	Age-standardized rate (95% UI)	All ages number (95% UI)	Age-standardized rate (95% UI)	All ages number (95% UI)	Age-standardized rate (95% UI)	All ages number (95% UI)	Age-standardized rate (95% UI)
1990	600857.66 (492848.6 to 708907.09)	36.64 (30.67 to 42.82)	595674.75 (486938.62 to 701900.71)	28.47 (23.62 to 33.49)	282.21 (125.52 to 533.9)	0.02 (0.01 to 0.03)	308.06 (136.49 to 587.91)	0.01 (0.01 to 0.03)
1991	612129.53	36.41 (30.51 to	608104.72 (498503.9 to	28.39 (23.56 to 33.37)	287.39 (129.61 to	0.02 (0.01 to 0.03)	313.5 (138.11 to 594.7)	0.01 (0.01 to 0.03)

	(503036.22 to 721165.72)	42.55)	716074.04)		538.1)			
1992	625077.34 (514624.96 to 734783.71)	36.26 (30.4 to 42.37)	621148.3 (510606.26 to 730795.54)	28.34 (23.53 to 33.31)	292.98 (130.72 to 545.71)	0.02 (0.01 to 0.03)	319.94 (139.43 to 611.17)	0.01 (0.01 to 0.03)
1993	639067.84 (526976.25 to 750039.68)	36.16 (30.33 to 42.26)	634329.65 (522814.94 to 745951.94)	28.3 (23.51 to 33.27)	299.98 (133.78 to 560.76)	0.02 (0.01 to 0.03)	326.1 (145.41 to 625.47)	0.01 (0.01 to 0.03)
1994	652939.48 (539172.76 to 765028.04)	36.05 (30.24 to 42.13)	646924.37 (534534.59 to 760609.72)	28.22 (23.45 to 33.18)	305.8 (137.87 to 573.44)	0.02 (0.01 to 0.03)	332.17 (146.97 to 628.25)	0.01 (0.01 to 0.03)
1995	667750.91 (552396.89 to 782400.24)	35.98 (30.22 to 42.06)	659677.98 (545738.26 to 774818.83)	28.15 (23.4 to 33.09)	312.44 (140.37 to 591.78)	0.02 (0.01 to 0.03)	337.79 (148.65 to 648.98)	0.01 (0.01 to 0.03)
1996	689534.24 (572308.31 to 804073.33)	36.25 (30.45 to 42.12)	674106.95 (559073.9 to 789082.22)	28.12 (23.37 to 32.92)	322.14 (143.94 to 612.76)	0.02 (0.01 to 0.03)	345.97 (152.55 to 660.75)	0.01 (0.01 to 0.03)
1997	724451.34 (604299.21 to 841710.55)	37.15 (31.27 to 43.01)	692492.59 (575111 to 806838.68)	28.22 (23.48 to 32.86)	337.18 (148.99 to 642.37)	0.02 (0.01 to 0.03)	354.85 (156.16 to 670.36)	0.01 (0.01 to 0.03)
1998	769386.85 (645406.33 to 889254.82)	38.51 (32.44 to 44.34)	713635.61 (594445.68 to 827559.94)	28.41 (23.66 to 32.88)	356.35 (159.1 to 671.45)	0.02 (0.01 to 0.03)	365.83 (165.19 to 693.97)	0.01 (0.01 to 0.03)
1999	819819.83 (689827.72 to 942381.45)	40.02 (33.79 to 45.94)	735705.02 (615322.02 to 849918.63)	28.59 (23.91 to 32.99)	378.2 (167.45 to 715.99)	0.02 (0.01 to 0.03)	376.98 (171.97 to 706.47)	0.01 (0.01 to 0.03)

2000	873824.07 (738233.62 to 999382.4)	41.61 (35.18 to 47.61)	758263.97 (636824.45 to 873056.29)	28.76 (24.2 to 33.09)	400.02 (180.88 to 763.67)	0.02 (0.01 to 0.04)	387.59 (174.49 to 721.92)	0.01 (0.01 to 0.03)
2001	967461.74 (827045.84 to 1098010.67)	44.9 (38.7 to 50.91)	818769.63 (698886.19 to 931344.01)	30.29 (25.82 to 34.48)	439.2 (201.78 to 828.01)	0.02 (0.01 to 0.04)	413.68 (184.66 to 777.44)	0.02 (0.01 to 0.03)
2002	1118333.82 (968097.81 to 1257246.08)	50.56 (44.41 to 56.87)	934591.42 (808881.29 to 1049099.89)	33.74 (29.26 to 37.95)	500.39 (227.95 to 947.66)	0.02 (0.01 to 0.04)	461.13 (208.26 to 867.05)	0.02 (0.01 to 0.03)
2003	1290548.09 (1130868.62 to 1433392.48)	56.82 (50.57 to 63.24)	1072023.21 (939318.87 to 1193969.44)	37.76 (33.14 to 42.09)	571.26 (260.02 to 1078.54)	0.02 (0.01 to 0.05)	517.14 (233.7 to 983.55)	0.02 (0.01 to 0.03)
2004	1442303.96 (1280574.58 to 1594079.65)	61.8 (55.28 to 68.31)	1194261.72 (1056470.61 to 1319812.13)	41.03 (36.3 to 45.34)	632.76 (289.99 to 1188.94)	0.03 (0.01 to 0.05)	568.03 (260.8 to 1079.62)	0.02 (0.01 to 0.04)
2005	1533292.32 (1366533.91 to 1693582.95)	63.91 (57.68 to 70.42)	1264420.36 (1127073.24 to 1388691.48)	42.36 (37.79 to 46.67)	669.47 (306.81 to 1276.82)	0.03 (0.01 to 0.05)	597.78 (272.47 to 1145.8)	0.02 (0.01 to 0.04)
2006	1575703.77 (1407220.21 to 1738569.17)	63.77 (57.52 to 70.19)	1293921.12 (1154859.19 to 1421756.71)	42.21 (37.68 to 46.54)	686.64 (316.55 to 1290.33)	0.03 (0.01 to 0.05)	610.31 (277.09 to 1167.82)	0.02 (0.01 to 0.04)
2007	1620759.04 (1452137.01 to 1786279.71)	63.61 (57.36 to 69.93)	1324464.64 (1183294.92 to 1457357.02)	42.06 (37.6 to 46.41)	705.46 (325.95 to 1348.02)	0.03 (0.01 to 0.05)	623.97 (288.27 to 1191.41)	0.02 (0.01 to 0.04)
2008	1667706.95 (1495722.07 to	63.5 (57.29 to 69.79)	1355457.61 (1210472.03 to	41.91 (37.47 to 46.28)	723.96 (334.12 to 1372.88)	0.03 (0.01 to 0.05)	638.23 (295.33 to 1207.15)	0.02 (0.01 to 0.04)

	1837446.62)		1494368.71)					
2009	1712676.29 (1539104.57 to 1884147.89)	63.22 (57.07 to 69.52)	1385033.34 (1239314.51 to 1529924.24)	41.67 (37.28 to 46)	741.9 (341.43 to 1409.38)	0.03 (0.01 to 0.05)	651.68 (297.84 to 1233.78)	0.02 (0.01 to 0.04)
2010	1760188.44 (1583361.68 to 1935521.2)	63.06 (56.91 to 69.36)	1414873.66 (1266074.33 to 1562467.76)	41.43 (37.12 to 45.76)	761.36 (353.51 to 1435.98)	0.03 (0.01 to 0.05)	664.12 (304.48 to 1256.15)	0.02 (0.01 to 0.04)
2011	1808217.32 (1624369.19 to 1987949.64)	62.82 (56.62 to 69.1)	1445168.74 (1292065.8 to 1594438.24)	41.17 (36.84 to 45.44)	782.64 (362 to 1486.36)	0.03 (0.01 to 0.05)	677.81 (314.25 to 1279.33)	0.02 (0.01 to 0.04)
2012	1863923.14 (1674534.08 to 2051770.52)	62.72 (56.55 to 68.97)	1479818.84 (1322561.5 to 1631325.05)	41 (36.68 to 45.23)	806.51 (369.79 to 1540.01)	0.03 (0.01 to 0.05)	694.46 (320.01 to 1313.22)	0.02 (0.01 to 0.04)
2013	1923432.54 (1728063.66 to 2119054.4)	62.72 (56.54 to 69.02)	1516618.61 (1354933.2 to 1670472.59)	40.88 (36.54 to 45.05)	832.05 (381.48 to 1574.49)	0.03 (0.01 to 0.05)	711.38 (326.81 to 1352.46)	0.02 (0.01 to 0.04)
2014	1981158.1 (1778141.21 to 2182591.93)	62.61 (56.39 to 68.95)	1552766.19 (1384594.34 to 1709951.58)	40.71 (36.35 to 44.89)	856.93 (396.49 to 1639.63)	0.03 (0.01 to 0.05)	728.21 (334.43 to 1370.1)	0.02 (0.01 to 0.04)
2015	2039593.15 (1827403.05 to 2249008.87)	62.57 (56.31 to 68.97)	1588549.37 (1414441.98 to 1750932.46)	40.54 (36.13 to 44.73)	881.98 (410.08 to 1676.11)	0.03 (0.01 to 0.05)	745.07 (343.07 to 1412.89)	0.02 (0.01 to 0.04)
2016	2088430.53 (1869001.12 to 2305279.95)	62.11 (55.76 to 68.53)	1618517.48 (1438447.09 to 1787977.69)	40.19 (35.75 to 44.43)	901.85 (419.4 to 1714.13)	0.03 (0.01 to 0.05)	758.23 (349.33 to 1438.44)	0.02 (0.01 to 0.04)
2017	2134495.42	61.51 (55.12 to	1646295.7 (1460145.02	39.77 (35.3 to 44.08)	919.49 (425.56 to	0.03 (0.01 to 0.05)	770.88 (349.85 to	0.02 (0.01 to 0.04)

	(1905334.89 to 2358669.01)	67.92)	to 1823712.81)		1732.86)		1453.92)	
2018	2183833.74 (1944836.46 to 2418443.5)	61 (54.43 to 67.42)	1676679.8 (1484197.82 to 1862839.93)	39.41 (34.91 to 43.78)	938.51 (435.15 to 1778.46)	0.03 (0.01 to 0.05)	783.16 (360.84 to 1473.99)	0.02 (0.01 to 0.03)
2019	2241120.14 (1993478.01 to 2480946.38)	60.65 (54 to 67.1)	1714406.38 (1515080.8 to 1910017.38)	39.21 (34.66 to 43.65)	963.2 (442.98 to 1825.43)	0.03 (0.01 to 0.05)	801.53 (368.52 to 1508.26)	0.02 (0.01 to 0.03)
2020	2309951.96 (2052334.09 to 2560209.81)	60.8 (54.06 to 67.27)	1759118.2 (1556258.87 to 1960993.88)	39.23 (34.7 to 43.68)	993.85 (455.18 to 1895.54)	0.03 (0.01 to 0.05)	825.26 (374.9 to 1569.26)	0.02 (0.01 to 0.04)
2021	2508840.49 (2216962.69 to 2791803.82)	64.29 (57.09 to 71.37)	1928098.55 (1691674.69 to 2159949.8)	42.03 (36.89 to 47)	1085.45 (500.34 to 2037.81)	0.03 (0.01 to 0.05)	912.4 (418.2 to 1745.46)	0.02 (0.01 to 0.04)

DALYs, disability-adjusted life-years; UI, uncertainty interval.

TABLE S8 Global incidence and DALYs of non-melanoma skin cancer (squamous-cell carcinoma) from 1990 to 2021, by sex, with age-standardized rates and 95% uncertainty intervals.

Year	Incidence				DALYs			
	Male		Female		Male		Female	
	All ages number (95% UI)	Age-standardized rate (95% UI)	All ages number (95% UI)	Age-standardized rate (95% UI)	All ages number (95% UI)	Age-standardized rate (95% UI)	All ages number (95% UI)	Age-standardized rate (95% UI)
199	273133.58	18.65 (14.66 to	191977.97 (152105.41	9.76 (7.73 to 12.6)	323196.95 (281190.37	18.25 (16.17 to	221776.73 (201296.8 to	10.61 (9.68 to

0	(213821.02 to 351037.15)	24.22)	to 246184.59)		to 364927.96)	20.55)	246804.63)	11.75)
1991	287841.9 (224657.22 to 370477.66)	19.13 (14.99 to 24.91)	205465.02 (162028.44 to 264318.56)	10.18 (8.02 to 13.17)	328671.75 (284336.45 to 370096.72)	18.08 (15.99 to 20.38)	226506.09 (205473.35 to 252677.17)	10.58 (9.66 to 11.75)
1992	301896.48 (235211.15 to 388346.83)	19.52 (15.27 to 25.44)	217544.02 (171386.23 to 280132.92)	10.49 (8.25 to 13.6)	336709.97 (289090.22 to 383154.62)	18.1 (15.86 to 20.43)	233837.76 (214750.34 to 256925.15)	10.67 (9.78 to 11.68)
1993	314865.38 (244833.02 to 405312.21)	19.81 (15.51 to 25.83)	227881.09 (179678.65 to 293634.06)	10.71 (8.41 to 13.89)	343711.26 (294728.6 to 389107.4)	18.03 (15.7 to 20.41)	239735.85 (219657.53 to 263768.32)	10.69 (9.79 to 11.71)
1994	325708.53 (253279.04 to 419676.2)	19.94 (15.64 to 25.99)	235760.35 (185833.98 to 303725.26)	10.8 (8.48 to 13.98)	349047.21 (303275.11 to 394410)	17.91 (15.81 to 20.06)	245986.37 (226068.48 to 266598.45)	10.72 (9.81 to 11.58)
1995	335097.31 (261326.42 to 431600.24)	19.98 (15.7 to 25.99)	241586.33 (190526.48 to 311132.7)	10.8 (8.5 to 13.93)	352352.34 (308412.94 to 402662.87)	17.72 (15.71 to 20.2)	253354.35 (234930.35 to 275042.99)	10.81 (10.01 to 11.69)
1996	342152.28 (269107.54 to 440690.78)	19.85 (15.68 to 25.75)	243661.13 (193696.92 to 310974.37)	10.62 (8.41 to 13.55)	356750.35 (309872.15 to 404902.15)	17.56 (15.54 to 19.77)	258515.33 (239264.61 to 280146.84)	10.79 (9.95 to 11.64)
1997	348154.83 (276682.64 to 444348.49)	19.62 (15.63 to 25.2)	242546.24 (194096.24 to 307045.21)	10.28 (8.23 to 12.96)	364471.16 (317009.26 to 416957.45)	17.53 (15.45 to 19.94)	261495.18 (241594.13 to 284329.91)	10.66 (9.78 to 11.54)
1998	354191.8 (282929.81 to 449405.74)	19.39 (15.58 to 24.54)	240331.62 (194436.49 to 301428.49)	9.91 (8.01 to 12.48)	373901.9 (324412.08 to 433406.87)	17.59 (15.54 to 20.24)	269245.76 (248255.68 to 290114.35)	10.72 (9.87 to 11.52)
1999	360523.62 (291425.92 to	19.15 (15.53 to 24.04)	238695.02 (194841.16 to 299369.36)	9.57 (7.84 to 11.92)	385060.85 (335839.15 to 440169.27)	17.65 (15.59 to 20.04)	278954.18 (258175.95 to 301224.17)	10.84 (10.05 to 11.66)

	454793.74)							
2000	369580.41 (300115.48 to 466182.72)	19.06 (15.54 to 23.63)	240585.14 (197279.4 to 300496.24)	9.39 (7.71 to 11.68)	398883.58 (346044.83 to 447786.94)	17.88 (15.71 to 20)	287518.32 (265923.61 to 310839.07)	10.91 (10.03 to 11.76)
2001	406943.17 (338401.55 to 498215.88)	20.39 (17.14 to 24.81)	265309.68 (222184.79 to 320813.86)	10.08 (8.51 to 12.19)	418819.79 (360487.5 to 465044.16)	18.35 (16 to 20.35)	295163.06 (271864.87 to 319722.36)	10.93 (10.06 to 11.78)
2002	484026.83 (415226.22 to 574320.8)	23.56 (20.38 to 27.93)	319849.71 (276466.7 to 375340.22)	11.84 (10.27 to 13.89)	435072.74 (376566.55 to 483873.59)	18.63 (16.3 to 20.69)	306941.23 (281263.39 to 332037.4)	11.09 (10.09 to 11.98)
2003	578145.72 (506603.71 to 667917.4)	27.37 (24.13 to 31.63)	386524.77 (340561.64 to 443662.23)	13.95 (12.34 to 16.01)	448166.89 (384392.95 to 495370.37)	18.77 (16.34 to 20.7)	316010.59 (290065.94 to 340816.21)	11.13 (10.17 to 11.98)
2004	662795.93 (586009.69 to 751898.41)	30.52 (27.19 to 34.6)	445790.79 (396540.15 to 504768.04)	15.68 (13.98 to 17.67)	462271.95 (391544.61 to 505448.95)	18.92 (16.27 to 20.67)	324357.41 (295682.37 to 354886.67)	11.15 (10.17 to 12.18)
2005	712629.11 (631744.11 to 803511.54)	31.89 (28.43 to 35.81)	478834.54 (426882.67 to 536940.36)	16.4 (14.62 to 18.33)	475581.05 (401256.53 to 522112.88)	18.98 (16.16 to 20.81)	333640.98 (306966.64 to 358068.75)	11.18 (10.23 to 11.97)
2006	733635.45 (651919.81 to 825585.43)	31.83 (28.48 to 35.68)	490698.66 (438255.38 to 548108.66)	16.34 (14.61 to 18.22)	486467.28 (412854.86 to 532547.42)	18.9 (16.22 to 20.71)	341336.57 (311224.06 to 365718.65)	11.14 (10.13 to 11.94)
2007	756604.66 (672792.68 to 848244.73)	31.78 (28.51 to 35.58)	503121.25 (450166.16 to 560410.95)	16.28 (14.62 to 18.14)	500253.03 (422399.98 to 549426.63)	18.92 (16.15 to 20.74)	352703.63 (322575.61 to 378095.18)	11.2 (10.18 to 12)
200	780931.56 (696589.9	31.77 (28.55 to	515874.95 (463158.75	16.23 (14.6 to 18.04)	518099.68 (429828.2 to	19.05 (16.01 to	363610.8 (331910.72 to	11.24 (10.24 to

8	to 873707.63)	35.5)	to 572273.85)		562127.06)	20.68)	389426.12)	12.04)
2009	803664.82 (717333.48 to 897590.96)	31.63 (28.45 to 35.28)	527556.73 (474379.98 to 584635.52)	16.12 (14.52 to 17.88)	534115.05 (444254.78 to 581938.75)	19.11 (16.05 to 20.8)	379302.4 (347269.65 to 405771.84)	11.41 (10.4 to 12.21)
2010	827876.31 (740191.39 to 924732.16)	31.55 (28.37 to 35.1)	539707.44 (485842.12 to 598512.64)	16.02 (14.45 to 17.76)	551752.13 (456098.17 to 599678.54)	19.2 (15.94 to 20.8)	392300.85 (351521.76 to 420202.38)	11.48 (10.27 to 12.31)
2011	850977.32 (760767.38 to 951154.57)	31.38 (28.2 to 34.94)	550959.04 (495820.97 to 611019.7)	15.87 (14.29 to 17.61)	566524.76 (465490.11 to 620675.01)	19.19 (15.92 to 20.98)	405117.05 (363517.12 to 436557.84)	11.53 (10.34 to 12.44)
2012	877492.14 (783765.58 to 981141.13)	31.25 (28.05 to 34.83)	563516.89 (507371.86 to 625560.21)	15.75 (14.18 to 17.51)	582182.09 (473000.24 to 636027.31)	19.19 (15.76 to 20.93)	417019.13 (373837.02 to 451108.17)	11.55 (10.34 to 12.5)
2013	905752.22 (808768 to 1013207.6)	31.18 (27.93 to 34.77)	576915.31 (519128.95 to 641530.52)	15.65 (14.09 to 17.42)	599008.05 (487534.32 to 670363.18)	19.23 (15.85 to 21.47)	427246.36 (380801.8 to 461241.69)	11.52 (10.26 to 12.44)
2014	932752.38 (833393.66 to 1043869.41)	31.04 (27.8 to 34.66)	589890.62 (530271.65 to 656528.42)	15.53 (13.96 to 17.29)	616492.64 (505599.26 to 686706.67)	19.27 (16.02 to 21.45)	435497.48 (393548.55 to 475275.49)	11.43 (10.33 to 12.48)
2015	961808.34 (858410.4 to 1076914.75)	31 (27.79 to 34.69)	604089.86 (542269.74 to 673102.34)	15.44 (13.87 to 17.21)	634279.49 (517856.11 to 696385.8)	19.3 (16.03 to 21.2)	444329.16 (391045.57 to 486644.57)	11.35 (9.99 to 12.43)
2016	988622.88 (882859.46 to 1106168.61)	30.81 (27.61 to 34.42)	617530.99 (554766.8 to 688000.39)	15.32 (13.76 to 17.07)	654111.75 (537528.87 to 735557.75)	19.37 (16.08 to 21.72)	454093.14 (399000.81 to 498674.84)	11.29 (9.93 to 12.4)
2017	1017456.44 (908673.6 to 1140225.81)	30.63 (27.4 to 34.25)	632086.95 (568110.14 to 705742.61)	15.21 (13.67 to 16.98)	666359.58 (549008.16 to 744037.12)	19.17 (15.94 to 21.35)	459905.33 (406617.11 to 508211.66)	11.13 (9.85 to 12.3)

2018	1048557.91 (935037.62 to 1177374.81)	30.52 (27.31 to 34.26)	647772.64 (581909.91 to 724034.48)	15.12 (13.59 to 16.9)	676288.36 (555241.94 to 762538.31)	18.91 (15.68 to 21.26)	468012.71 (411549.83 to 516321.56)	11.03 (9.7 to 12.17)
2019	1080640.07 (961464.27 to 1217974.62)	30.38 (27.15 to 34.25)	663933.03 (594882.55 to 743142.57)	15.03 (13.47 to 16.82)	690456.22 (571156.83 to 777210.78)	18.77 (15.65 to 21.05)	478960.44 (417884.44 to 529726.21)	10.98 (9.59 to 12.15)
2020	1117508.45 (992542.35 to 1261608.31)	30.5 (27.21 to 34.4)	681725.83 (610514.35 to 762941.08)	15.01 (13.45 to 16.79)	701995.43 (579480.42 to 793653.65)	18.61 (15.54 to 20.95)	484641.54 (427303.09 to 540320.99)	10.84 (9.57 to 12.1)
2021	1187477.14 (1051961.99 to 1348915.21)	31.53 (27.96 to 35.7)	712429.91 (636435.22 to 800222)	15.3 (13.67 to 17.17)	716678.64 (594142.43 to 807397.25)	18.58 (15.55 to 20.84)	494195.89 (432594.73 to 550339.88)	10.8 (9.47 to 12.02)

DALYs, disability-adjusted life-years; UI, uncertainty interval.

TABLE S9 Incidence and DALYs of total skin cancer in 1990 and 2021 across 204 countries and territories, with age-standardized rates, 95% uncertainty intervals, and trends from 1990 to 2021.

Location name	Incidence				DALYs			
	Age-standardized rate (95% UI), 1990	Age-standardized rate (95% UI), 2021	EAPC (95% CI), 1990-2021	Change in age-standardized rate, 1990-2021	Age-standardized rate (95% UI), 1990	Age-standardized rate (95% UI), 2021	EAPC (95% CI),1990-2021	Change in age-standardized rate, 1990-2021
Afghanistan	5.55 (3.89 to 7.53)	5.66 (3.86 to 7.92)	0 (0 to 0)	1.95%	10.76 (3.24 to 25.97)	11.16 (3.4 to 26.32)	0 (0 to 0)	3.70%
Albania	29.54 (23.23 to	31.12 (24.08 to	0.23 (0.21 to	5.35%	49.02 (36.52 to	40.18 (24.91 to	0 (0 to 0)	-18.04%

	36.54)	39.02)	0.25)		67.25)	59.1)		
Algeria	7.09 (6.12 to 8.1)	7.11 (5.32 to 8.85)	0 (0 to 0)	0.24%	2.79 (1.86 to 4.46)	3.08 (1.59 to 5.81)	0 (0 to 0)	10.06%
American Samoa	0.59 (0.39 to 1)	0.71 (0.42 to 0.99)	0 (0 to 0)	20.45%	22.12 (16.27 to 38.47)	32.11 (20.41 to 41.62)	1.84 (1.34 to 2.34)	45.11%
Andorra	49.72 (38.02 to 64.98)	51.81 (38.87 to 67.39)	0 (0 to 0)	4.21%	74.98 (49.07 to 116.05)	50.27 (30.53 to 74.32)	-1.01 (-1.19 to -0.84)	-32.95%
Angola	5.13 (3.79 to 6.58)	5.16 (3.77 to 6.6)	0 (0 to 0)	0.68%	19.71 (11.3 to 30.29)	24.33 (12.01 to 38.72)	0 (0 to 0)	23.43%
Antigua and Barbuda	4.74 (3.75 to 5.71)	5.68 (4.65 to 6.73)	0 (0 to 0)	19.73%	21.16 (19.63 to 22.52)	41.18 (38.81 to 43.57)	0 (0 to 0)	94.61%
Argentina	27.86 (24.06 to 31.78)	28.2 (22.46 to 34.4)	0 (0 to 0)	1.21%	41.62 (39.09 to 44.26)	45.43 (42.41 to 48.96)	0 (0 to 0)	9.16%
Armenia	23.1 (17.87 to 28.21)	23.8 (18.23 to 29.56)	0.11 (0.09 to 0.12)	3.03%	14.26 (11.88 to 17.09)	30.56 (25.14 to 36.29)	0 (0 to 0)	114.32%
Australia	146.92 (121.16 to 175.99)	126.21 (101.77 to 152.84)	0 (0 to 0)	-14.10%	239.48 (224.46 to 253.81)	138.36 (125.64 to 152.13)	0 (0 to 0)	-42.22%
Austria	35.41 (32.24 to 38.58)	44.39 (36.8 to 53.12)	0 (0 to 0)	25.35%	84.77 (79.91 to 89.64)	72.4 (66.7 to 77.76)	-0.11 (-0.29 to 0.06)	-14.58%
Azerbaijan	23.77 (18.21 to 29.17)	24.08 (18.63 to 29.99)	0.06 (0.05 to 0.07)	1.33%	23.53 (15.28 to 34.73)	20.99 (12.63 to 33.42)	0 (0 to 0)	-10.81%
Bahamas	4.77 (3.77 to 5.75)	5.37 (4.1 to 6.71)	0 (0 to 0)	12.73%	35.42 (32.43 to 38.82)	41.74 (33.49 to 52.08)	0 (0 to 0)	17.83%
Bahrain	4.32 (3.04 to 5.55)	4.94 (3.23 to 6.65)	0 (0 to 0)	14.52%	8.8 (6.83 to 11.98)	9.38 (6.09 to 12.64)	0 (0 to 0)	6.63%
Bangladesh	0.33 (0.22 to 0.52)	0.4 (0.21 to 0.75)	0 (0 to 0)	21.06%	13.4 (8.62 to 19.87)	13.09 (7.14 to 22.65)	0 (0 to 0)	-2.27%
Barbados	4.22 (3.25 to 5.13)	4.77 (3.59 to 5.97)	0 (0 to 0)	12.95%	18.79 (17.56 to	20.32 (16.13 to	0 (0 to 0)	8.15%

					20.09)	25.18)		
Belarus	24.03 (22.92 to 25.14)	31.61 (24.72 to 39.08)	0 (0 to 0)	31.50%	45.36 (40 to 50.36)	74.41 (60.06 to 91.77)	0 (0 to 0)	64.05%
Belgium	39.92 (33.29 to 47.07)	48.59 (40 to 58.38)	0 (0 to 0)	21.70%	60.26 (56.7 to 64.15)	64.12 (58.57 to 70.3)	0 (0 to 0)	6.41%
Belize	4.1 (3.11 to 5.05)	4.42 (3.37 to 5.4)	0 (0 to 0)	7.84%	19.97 (18.1 to 21.91)	28.89 (25.19 to 32.85)	0 (0 to 0)	44.69%
Benin	3.19 (2.34 to 3.97)	3.24 (2.33 to 4.06)	0 (0 to 0)	1.50%	7.14 (4.09 to 9.26)	8.6 (3.09 to 13.21)	0 (0 to 0)	20.43%
Bermuda	6.63 (5.6 to 7.74)	10.03 (8.04 to 12.48)	0 (0 to 0)	51.28%	63.24 (58.41 to 68.6)	62.68 (52.81 to 76.42)	-0.29 (-0.52 to -0.07)	-0.89%
Bhutan	0.33 (0.19 to 0.55)	0.39 (0.21 to 0.76)	0 (0 to 0)	20.95%	11.9 (7.06 to 18.48)	12.79 (7.78 to 21.83)	0 (0 to 0)	7.47%
Bolivia (Plurinational State of)	13.83 (10.75 to 17.59)	14.25 (10.79 to 17.99)	0 (0 to 0)	3.07%	49.96 (31.82 to 84.28)	54.1 (33.59 to 80.4)	0 (0 to 0)	8.29%
Bosnia and Herzegovina	30.84 (24.24 to 38.68)	33.96 (25.38 to 42.6)	0 (0 to 0)	10.12%	48.37 (38.2 to 69.58)	53.03 (29.36 to 70.64)	0 (0 to 0)	9.62%
Botswana	18.88 (14.34 to 23.76)	18.94 (14.2 to 24.06)	0 (0 to 0)	0.31%	42.01 (28.23 to 75.26)	39.67 (22.25 to 71.72)	0 (0 to 0)	-5.58%
Brazil	70.39 (61.55 to 78.95)	43.8 (36.05 to 51.63)	0 (0 to 0)	-37.77%	47.66 (45.79 to 49.49)	52.5 (49.21 to 54.98)	0 (0 to 0)	10.15%
Brunei Darussalam	6.09 (4.68 to 7.85)	6.35 (4.87 to 8.15)	0 (0 to 0)	4.26%	26.91 (19.5 to 38.02)	23 (16.41 to 31.48)	0 (0 to 0)	-14.54%
Bulgaria	40.56 (32.4 to 49.57)	56.28 (45.45 to 66.39)	0 (0 to 0)	38.76%	55.37 (49.11 to 62.75)	67.17 (55.61 to 80.6)	0 (0 to 0)	21.32%
Burkina Faso	3.23 (2.38 to 4.01)	3.28 (2.36 to 4.12)	0 (0 to 0)	1.64%	8.15 (4.5 to 10.98)	9.55 (3.79 to 14.36)	0 (0 to 0)	17.21%

Burundi	5 (3.42 to 6.68)	4.83 (3.43 to 6.2)	0 (0 to 0)	-3.47%	43.98 (22.15 to 69.78)	35.1 (17.75 to 52.36)	0 (0 to 0)	-20.19%
Cabo Verde	2.92 (2.18 to 3.73)	3.31 (2.27 to 4.19)	0 (0 to 0)	13.35%	3.34 (2.2 to 6.03)	6.73 (2.3 to 10.09)	0 (0 to 0)	101.25%
Cambodia	1.23 (0.88 to 1.66)	1.34 (0.96 to 1.79)	0.25 (0.22 to 0.29)	8.41%	18.58 (12.56 to 26.63)	23.66 (15.7 to 32.96)	0 (0 to 0)	27.32%
Cameroon	3.26 (2.39 to 4.09)	3.33 (2.37 to 4.21)	0 (0 to 0)	1.88%	8.69 (4.73 to 11.59)	10.91 (4.1 to 17.09)	0 (0 to 0)	25.57%
Canada	39.17 (31.5 to 46.88)	49.42 (39.41 to 59.81)	0 (0 to 0)	26.15%	73.53 (69.46 to 77.49)	62.3 (57.37 to 66.86)	0 (0 to 0)	-15.27%
Central African Republic	5.14 (3.76 to 6.57)	5.11 (3.77 to 6.54)	0 (0 to 0)	-0.66%	22.04 (12.4 to 35.05)	22.87 (11.72 to 36.48)	0.14 (0.1 to 0.18)	3.80%
Chad	3.18 (2.35 to 3.95)	3.33 (2.42 to 4.19)	0 (0 to 0)	4.61%	6.27 (3.6 to 8.52)	9.15 (4.04 to 14.3)	0 (0 to 0)	45.82%
Chile	23.32 (19.22 to 27.84)	26.11 (20.93 to 31.6)	0 (0 to 0)	11.97%	36.78 (34.4 to 39.19)	45.15 (41.43 to 48.82)	0.61 (0.26 to 0.97)	22.75%
China	5.01 (4.04 to 6.03)	38.22 (31.31 to 45.14)	4.47 (3.72 to 5.23)	662.31%	25.33 (19.3 to 31.46)	25.78 (18.52 to 32.67)	0 (0 to 0)	1.78%
Colombia	29.16 (23.08 to 35.79)	29.91 (23.88 to 36.28)	0 (0 to 0)	2.56%	46.15 (43.54 to 48.68)	37.45 (31.33 to 44.53)	0 (0 to 0)	-18.84%
Comoros	4.7 (3.27 to 5.98)	4.76 (3.33 to 6.22)	0 (0 to 0)	1.14%	35.21 (16.72 to 50.82)	35.8 (16.92 to 56.76)	0 (0 to 0)	1.70%
Congo	5.11 (3.81 to 6.56)	5.33 (3.91 to 6.74)	0 (0 to 0)	4.39%	23.82 (14.45 to 36.66)	26.57 (13.99 to 40.4)	0.32 (0.22 to 0.42)	11.53%
Cook Islands	0.48 (0.29 to 0.69)	0.52 (0.33 to 0.8)	0 (0 to 0)	9.60%	14 (9.89 to 20.31)	12.8 (9.11 to 18.33)	-0.06 (-0.47 to 0.34)	-8.55%
Costa Rica	46.5 (43.95 to 49.05)	35.52 (28.73 to 42.73)	0 (0 to 0)	-23.61%	46.72 (43.06 to 50.05)	47.23 (41.02 to 53.39)	0 (0 to 0)	1.09%
Croatia	27.05 (21.98 to	33.91 (26.95 to	0 (0 to 0)	25.38%	89.89 (81.42 to	83.39 (71.06 to	0 (0 to 0)	-7.23%

	33.28)	41.65)			99.2)	96.13)		
Cuba	12.19 (9.66 to 14.74)	10.7 (8.47 to 12.94)	0 (0 to 0)	-12.23%	44.02 (41.65 to 46.51)	54.47 (46.75 to 61.74)	0 (0 to 0)	23.73%
Cyprus	29.56 (24.92 to 35.57)	35.18 (25.4 to 44.72)	0 (0 to 0)	19.03%	70.2 (50.52 to 105.69)	50.99 (30.74 to 69.21)	0 (0 to 0)	-27.37%
Czechia	59.8 (56.85 to 62.56)	60.31 (48.69 to 73.36)	0 (0 to 0)	0.85%	95.08 (88.34 to 102.41)	74.76 (63.66 to 86.67)	0 (0 to 0)	-21.37%
Côte d'Ivoire	2.45 (1.78 to 3.09)	2.55 (1.77 to 3.35)	0 (0 to 0)	3.81%	13.38 (7.79 to 17.71)	16.1 (7.01 to 24.44)	0 (0 to 0)	20.32%
Democratic People's Republic of Korea	0.76 (0.52 to 1.18)	0.83 (0.58 to 1.2)	0.35 (0.29 to 0.4)	9.06%	22.52 (15.5 to 34.24)	26.52 (19.1 to 37.68)	0 (0 to 0)	17.73%
Democratic Republic of the Congo	5.05 (3.75 to 6.31)	5.09 (3.79 to 6.51)	0 (0 to 0)	0.84%	18.8 (11.25 to 28.62)	22.59 (11.14 to 36.48)	0 (0 to 0)	20.11%
Denmark	64.2 (60.67 to 67.59)	64.78 (53.65 to 77.04)	0 (0 to 0)	0.91%	77.95 (73.95 to 82.23)	83.68 (76.25 to 90.95)	0 (0 to 0)	7.35%
Djibouti	4.7 (3.25 to 6.06)	4.85 (3.31 to 6.44)	0 (0 to 0)	3.35%	32.37 (16.14 to 49.9)	34.91 (15.16 to 59.83)	0 (0 to 0)	7.87%
Dominica	4.01 (2.98 to 5.1)	4.37 (3.13 to 5.49)	0 (0 to 0)	8.86%	19.68 (15.19 to 26.91)	22.44 (14.92 to 30.11)	0.42 (0.37 to 0.47)	14.03%
Dominican Republic	4.04 (3.06 to 5.06)	4.11 (3.08 to 5.21)	0 (0 to 0)	1.58%	18.19 (13.6 to 35.16)	34.09 (23.93 to 44.4)	0 (0 to 0)	87.46%
Ecuador	24.92 (22.76 to 27.39)	16.42 (12.69 to 20.35)	0 (0 to 0)	-34.11%	30.74 (28.66 to 32.81)	52.31 (41.83 to 64.5)	2.16 (1.8 to 2.52)	70.14%
Egypt	5.32 (4.41 to 6.27)	5.19 (3.93 to 6.52)	0 (0 to 0)	-2.58%	1.84 (1.32 to 4.16)	6.36 (3.83 to 8.06)	0 (0 to 0)	245.40%
El Salvador	28.7 (22.57 to 34.56)	28.91 (22.28 to 35.18)	0 (0 to 0)	0.74%	14.04 (11.77 to 18.86)	16.93 (11.9 to 21.74)	0 (0 to 0)	20.59%

Equatorial Guinea	5.01 (3.72 to 6.28)	5.26 (3.74 to 6.76)	0 (0 to 0)	4.81%	20.85 (11.78 to 33.34)	23.37 (10 to 39.09)	0 (0 to 0)	12.06%
Eritrea	4.77 (3.39 to 6.34)	4.88 (3.49 to 6.43)	0 (0 to 0)	2.33%	41.04 (22.41 to 64.68)	43.08 (23.56 to 65.69)	0 (0 to 0)	4.97%
Estonia	28.95 (27.01 to 30.98)	33.59 (26.67 to 40.69)	0 (0 to 0)	16.02%	70.25 (62.67 to 78.37)	75.06 (64.08 to 86.51)	0 (0 to 0)	6.83%
Eswatini	18.93 (14.54 to 24.16)	18.97 (14.43 to 24.45)	0 (0 to 0)	0.19%	43.28 (31.29 to 78.35)	52.91 (33.07 to 91.45)	0 (0 to 0)	22.24%
Ethiopia	4.52 (3.25 to 5.79)	4.53 (3.31 to 5.65)	0 (0 to 0)	0.17%	23.43 (11.77 to 38.16)	21.66 (11.05 to 32.52)	0 (0 to 0)	-7.55%
Fiji	0.46 (0.25 to 0.97)	0.5 (0.3 to 0.96)	0.39 (0.3 to 0.49)	10.10%	15.98 (10.29 to 28.37)	15.84 (10.5 to 26.41)	0 (0 to 0)	-0.82%
Finland	46.13 (37.63 to 55.43)	57.22 (47 to 68.45)	0 (0 to 0)	24.06%	69.49 (65.92 to 73.41)	71.99 (65.63 to 79.15)	0 (0 to 0)	3.60%
France	63.75 (57.49 to 69.65)	59.13 (48.16 to 71)	0 (0 to 0)	-7.24%	62.75 (59.61 to 65.94)	56.69 (52.06 to 61.7)	0 (0 to 0)	-9.66%
Gabon	5.21 (3.89 to 6.64)	5.37 (3.91 to 6.97)	0 (0 to 0)	3.19%	24.82 (14.98 to 38.01)	26.26 (12.32 to 40.49)	0 (0 to 0)	5.83%
Gambia	3.44 (2.61 to 4.3)	3.56 (2.58 to 4.51)	0 (0 to 0)	3.32%	7.8 (4.44 to 11.19)	11.54 (5.1 to 17.89)	1.11 (0.91 to 1.31)	47.85%
Georgia	25.74 (19.89 to 31.45)	25.45 (20.14 to 30.89)	-0.11 (-0.21 to -0.01)	-1.13%	51.21 (43.18 to 59.34)	100.12 (86.64 to 114.48)	0 (0 to 0)	95.49%
Germany	29.62 (26.55 to 32.56)	46.99 (38.98 to 56.23)	0 (0 to 0)	58.64%	65.97 (62.28 to 69.65)	61.37 (56.18 to 66.81)	0 (0 to 0)	-6.96%
Ghana	3.04 (2.25 to 3.78)	2.99 (2.23 to 3.71)	0 (0 to 0)	-1.54%	3.53 (2.36 to 4.93)	3.91 (2.36 to 5.76)	0 (0 to 0)	10.90%
Greece	43.11 (34.3 to	48.04 (39.19 to	0 (0 to 0)	11.44%	39.58 (37.44 to	54.09 (50.47 to	0 (0 to 0)	36.66%

	52.82)	58.17)			41.49)	57.71)		
Greenland	94.84 (74.21 to 117.43)	99.71 (77.63 to 123.44)	0 (0 to 0)	5.13%	51.03 (35.66 to 67.01)	36.12 (22.74 to 51.01)	0 (0 to 0)	-29.21%
Grenada	4.28 (3.26 to 5.31)	4.87 (3.74 to 6.04)	0 (0 to 0)	13.81%	15.27 (13.56 to 17.04)	34.72 (29.8 to 40.35)	0 (0 to 0)	127.43%
Guam	0.27 (0.17 to 0.47)	0.27 (0.17 to 0.43)	0 (0 to 0)	0.27%	8.15 (6.19 to 16.51)	9.81 (6.01 to 12.49)	0 (0 to 0)	20.28%
Guatemala	29.64 (23.46 to 35.95)	29.85 (23.63 to 36.37)	0 (0 to 0)	0.69%	37.73 (35.28 to 40.11)	29.15 (25.02 to 33.89)	0 (0 to 0)	-22.75%
Guinea	3.68 (2.77 to 4.68)	3.76 (2.66 to 4.91)	0 (0 to 0)	2.05%	25.75 (17.77 to 36.28)	30.08 (16.59 to 45.17)	0 (0 to 0)	16.81%
Guinea-Bissau	3.29 (2.35 to 4.13)	3.31 (2.38 to 4.23)	0 (0 to 0)	0.77%	9.65 (5.28 to 14.82)	12.21 (5.59 to 18.31)	0 (0 to 0)	26.48%
Guyana	4.18 (3.18 to 5.21)	4.35 (3.23 to 5.48)	0 (0 to 0)	4.14%	9.16 (8.08 to 10.32)	26.53 (20.08 to 33.83)	0 (0 to 0)	189.71%
Haiti	4.56 (3.3 to 6.1)	4.65 (3.29 to 6.2)	0 (0 to 0)	1.95%	30.03 (18.88 to 52.79)	34.9 (19.99 to 56.25)	0 (0 to 0)	16.22%
Honduras	29.61 (23.29 to 35.46)	29.76 (23.29 to 36.08)	0 (0 to 0)	0.52%	18.04 (12.78 to 22.91)	22.21 (15.02 to 31.55)	0 (0 to 0)	23.13%
Hungary	36.37 (33.15 to 39.86)	38.15 (30.52 to 46.71)	0 (0 to 0)	4.88%	91.94 (85.46 to 98.01)	71.11 (60.92 to 82.07)	0 (0 to 0)	-22.66%
Iceland	47.09 (38.17 to 56.71)	58.21 (47.45 to 69.53)	0 (0 to 0)	23.61%	50.07 (46.46 to 53.6)	68.83 (61.27 to 78.65)	0 (0 to 0)	37.47%
India	1.86 (1.36 to 2.39)	2.11 (1.56 to 2.68)	0 (0 to 0)	12.97%	11.86 (8.11 to 15.73)	13.67 (10.23 to 18.13)	0 (0 to 0)	15.23%
Indonesia	1.31 (0.97 to 1.7)	1.36 (0.99 to 1.76)	0.12 (0.12 to 0.13)	4.19%	15.03 (11.07 to 19.78)	19.5 (13.63 to 25.19)	0 (0 to 0)	29.75%

Iran (Islamic Republic of)	13.18 (9.49 to 16.51)	11.85 (8.46 to 14.69)	0 (0 to 0)	-10.07%	15.62 (7.19 to 21.27)	10.98 (6.24 to 13.77)	0 (0 to 0)	-29.68%
Iraq	4.85 (3.65 to 6.18)	5.51 (4.71 to 6.46)	0 (0 to 0)	13.58%	12.63 (8.78 to 17.77)	13.45 (9.27 to 18.85)	0 (0 to 0)	6.47%
Ireland	95.52 (86.27 to 105.04)	72.92 (59.68 to 87.05)	0 (0 to 0)	-23.66%	83.03 (77.92 to 88.21)	71.64 (64.55 to 78.98)	0 (0 to 0)	-13.71%
Israel	47.26 (37.69 to 57.58)	52.97 (43.31 to 63.33)	0 (0 to 0)	12.07%	85.37 (79.94 to 90.43)	74.64 (67.67 to 80.23)	0 (0 to 0)	-12.57%
Italy	50.65 (43.98 to 57.99)	59.29 (48.64 to 71.25)	0 (0 to 0)	17.05%	64.73 (62.26 to 67.17)	60.18 (56.04 to 64.19)	0 (-0.16 to 0.16)	-7.03%
Jamaica	5.95 (4.92 to 7)	5.4 (4.07 to 6.78)	0 (0 to 0)	-9.20%	13.22 (12.1 to 14.43)	19.19 (14.4 to 25.06)	0 (0 to 0)	45.16%
Japan	3.94 (3.24 to 4.7)	5.96 (4.89 to 7.18)	0 (0 to 0)	51.14%	11.33 (10.74 to 11.77)	11.62 (10.75 to 12.32)	0 (0 to 0)	2.60%
Jordan	8.46 (6.76 to 10.3)	7.67 (5.72 to 9.73)	0 (0 to 0)	-9.36%	7.84 (5.9 to 12.26)	9.13 (6.3 to 12.12)	0 (0 to 0)	16.49%
Kazakhstan	23.98 (18.76 to 29.24)	24.97 (19.63 to 30.55)	0.12 (0.09 to 0.14)	4.13%	33.9 (27.63 to 42.05)	41.68 (35.35 to 48.54)	0 (0 to 0)	22.93%
Kenya	4.33 (3.18 to 5.48)	4.24 (3.07 to 5.41)	0 (0 to 0)	-2.07%	16.7 (9.65 to 24.11)	22.46 (11.24 to 33.43)	0 (0 to 0)	34.48%
Kiribati	0.21 (0.11 to 0.34)	0.21 (0.11 to 0.35)	0 (0 to 0)	1.76%	4.27 (2.94 to 6.09)	4.61 (3.07 to 6.62)	0 (0 to 0)	8.16%
Kuwait	4.74 (3.94 to 5.55)	4.91 (3.73 to 6.09)	0 (0 to 0)	3.58%	4.16 (3.74 to 4.58)	5.35 (4.33 to 6.58)	1.55 (0.56 to 2.55)	28.67%
Kyrgyzstan	25.97 (23.3 to 28.6)	23.76 (18.56 to 29.28)	-0.32 (-0.4 to -0.23)	-8.53%	42.01 (35.37 to 49.93)	48.32 (39.64 to 57.92)	0 (0 to 0)	15.02%
Lao People's Democratic Republic	1.29 (0.89 to 1.8)	1.33 (0.95 to 1.79)	0.11 (0.09 to 0.12)	3.41%	18.46 (11.48 to 30.64)	19.99 (13.28 to 27.53)	0 (0 to 0)	8.28%

Latvia	21.65 (20.25 to 23.01)	28.72 (23.08 to 35.27)	0 (0 to 0)	32.65%	58.94 (52.37 to 65.38)	87.68 (75.69 to 101.92)	0 (0 to 0)	48.76%
Lebanon	10.26 (7.89 to 12.9)	11.42 (8.56 to 14.57)	0 (0 to 0)	11.23%	10.56 (6.94 to 16.38)	9.93 (6.95 to 14.36)	0.16 (0.03 to 0.3)	-6.00%
Lesotho	17.95 (13.68 to 22.71)	18.64 (14.05 to 23.91)	0 (0 to 0)	3.85%	33.9 (22.09 to 63.83)	50.98 (31.87 to 91.95)	1.79 (1.52 to 2.07)	50.37%
Liberia	3.29 (2.44 to 4.1)	3.34 (2.43 to 4.2)	0 (0 to 0)	1.37%	7.19 (3.89 to 9.79)	9.38 (3.49 to 15)	0 (0 to 0)	30.37%
Libya	3.7 (2.68 to 4.75)	3.9 (2.78 to 4.99)	0 (0 to 0)	5.65%	2.5 (1.5 to 4.28)	2.93 (1.56 to 6.61)	0 (0 to 0)	17.38%
Lithuania	29.86 (27.58 to 32.04)	35.52 (29.27 to 42.18)	0 (0 to 0)	18.96%	44.23 (39.65 to 48.7)	78.46 (67.43 to 90.19)	0 (0 to 0)	77.40%
Luxembourg	45.51 (36.8 to 55.21)	52.66 (42.69 to 63.78)	0 (0 to 0)	15.71%	71.94 (68.08 to 76.01)	58.27 (52.43 to 65.05)	0 (0 to 0)	-19.00%
Madagascar	4.79 (3.44 to 6.1)	4.67 (3.32 to 6.05)	0 (0 to 0)	-2.64%	34.71 (19.48 to 48.58)	31.66 (16.24 to 50)	-0.35 (-0.44 to -0.26)	-8.80%
Malawi	4.87 (3.46 to 6.24)	5.31 (3.38 to 7.34)	0 (0 to 0)	9.15%	59.32 (36.59 to 82.37)	68.3 (33.03 to 103.17)	0 (0 to 0)	15.14%
Malaysia	3.82 (3.03 to 4.59)	3.04 (2.21 to 3.8)	-0.85 (-1.12 to -0.58)	-20.48%	23.12 (15.57 to 28.73)	21.3 (15.07 to 27.11)	0 (0 to 0)	-7.88%
Maldives	1.22 (0.88 to 1.62)	1.19 (0.84 to 1.54)	-0.09 (-0.1 to -0.07)	-2.20%	7.05 (4.28 to 11.37)	4.76 (3.23 to 6.71)	0 (0 to 0)	-32.55%
Mali	5.72 (4.35 to 7.03)	5.77 (4.19 to 7.33)	0 (0 to 0)	0.92%	23.89 (15.22 to 31.29)	24.45 (11.28 to 37.39)	0 (0 to 0)	2.32%
Malta	53.37 (48.79 to 58.62)	51.33 (41.19 to 62.69)	0 (0 to 0)	-3.83%	40.81 (37.93 to 43.95)	45.27 (40.44 to 50.44)	0 (0 to 0)	10.94%
Marshall Islands	0.46 (0.28 to 0.8)	0.5 (0.3 to 0.81)	0.33 (0.26 to 0.39)	8.21%	20.38 (14.12 to 32.42)	25.39 (16.89 to 37.34)	0 (0 to 0)	24.59%

Mauritania	3.19 (2.34 to 3.98)	3.32 (2.41 to 4.21)	0 (0 to 0)	4.24%	7.72 (4.4 to 10.69)	9.17 (3.61 to 14.63)	0 (0 to 0)	18.79%
Mauritius	1.07 (0.78 to 1.37)	1.32 (1.03 to 1.65)	0 (0 to 0)	23.73%	2.55 (2.43 to 2.69)	10.54 (9.73 to 11.13)	0 (0 to 0)	312.88%
Mexico	30.35 (24.22 to 36.73)	31.1 (24.87 to 37.67)	0 (0 to 0)	2.48%	41.75 (40.51 to 42.83)	36.39 (32.17 to 40.75)	0 (0 to 0)	-12.84%
Micronesia (Federated States of)	0.48 (0.28 to 0.85)	0.48 (0.29 to 0.77)	0 (0 to 0)	-0.28%	22.11 (14.88 to 35.15)	25.35 (16.67 to 36.13)	0 (0 to 0)	14.67%
Monaco	47.28 (36.43 to 60.19)	55.32 (40.35 to 70.62)	0 (0 to 0)	17.00%	50.62 (35.46 to 77.57)	64.75 (36.8 to 88.79)	0 (0 to 0)	27.92%
Mongolia	24.08 (18.77 to 29.69)	24.13 (18.61 to 29.77)	0.01 (0 to 0.03)	0.23%	14.89 (7.62 to 25.77)	21.78 (14.45 to 31.08)	0 (0 to 0)	46.31%
Montenegro	33.21 (25.72 to 41.86)	35.19 (26.26 to 44.43)	0.28 (0.24 to 0.32)	5.95%	52.22 (37.42 to 70.91)	53.88 (34.24 to 75.09)	0 (0 to 0)	3.16%
Morocco	3.53 (2.46 to 4.71)	4.25 (2.63 to 5.71)	0 (0 to 0)	20.32%	6.46 (2.68 to 11.18)	7.61 (2.6 to 13.02)	0 (0 to 0)	17.81%
Mozambique	4.8 (3.37 to 6.16)	4.94 (3.43 to 6.44)	0 (0 to 0)	2.83%	36.63 (19.04 to 53.06)	44.16 (21.37 to 68.2)	0 (0 to 0)	20.55%
Myanmar	1.26 (0.88 to 1.73)	1.28 (0.91 to 1.7)	0.03 (0.02 to 0.04)	1.58%	17.8 (11.99 to 28.86)	17.92 (12.08 to 24.29)	-0.14 (-0.25 to -0.04)	0.67%
Namibia	20.14 (15.43 to 25.05)	21.68 (16.17 to 27.86)	0 (0 to 0)	7.65%	73.46 (52.73 to 105.17)	93.43 (59.74 to 140.07)	0 (0 to 0)	27.19%
Nauru	0.55 (0.3 to 1.03)	0.53 (0.3 to 0.9)	0 (0 to 0)	-3.91%	24.91 (14.82 to 42.79)	27.2 (16.07 to 45.97)	0 (0 to 0)	9.19%
Nepal	0.29 (0.18 to 0.49)	0.38 (0.24 to 0.69)	0 (0 to 0)	31.63%	11.17 (6.68 to 17.91)	12.96 (8.32 to 21.45)	0 (0 to 0)	15.97%
Netherlands	36.13 (30.35 to 42.32)	55.5 (46.41 to 65.57)	0 (0 to 0)	53.58%	75.9 (71.7 to 80.37)	89.07 (82.11 to 96.27)	0 (0 to 0)	17.35%

New Zealand	121.45 (97.38 to 150.12)	136.04 (110.99 to 164.92)	0 (0 to 0)	12.01%	169.31 (155.79 to 183.21)	159.45 (144.05 to 173.35)	0 (0 to 0)	-5.82%
Nicaragua	28.47 (22.28 to 34.85)	28.72 (22.72 to 35.38)	0 (0 to 0)	0.88%	17.76 (14.22 to 25.03)	20.44 (14.54 to 26.67)	0 (0 to 0)	15.09%
Niger	3.23 (2.38 to 3.98)	3.23 (2.33 to 4.03)	0 (0 to 0)	0.03%	6.6 (3.82 to 8.91)	7.85 (3.04 to 12.14)	0 (0 to 0)	18.93%
Nigeria	3.25 (2.22 to 4.2)	3.36 (2.19 to 4.4)	0 (0 to 0)	3.47%	18.44 (7.97 to 28)	20.67 (6.69 to 32.16)	0 (0 to 0)	12.10%
Niue	0.52 (0.32 to 0.87)	0.59 (0.37 to 0.92)	0 (0 to 0)	12.30%	21.56 (14.72 to 32.11)	25.16 (16.25 to 36.14)	0 (0 to 0)	16.72%
North Macedonia	35.18 (27.81 to 45.06)	38.99 (29.02 to 49.73)	0 (0 to 0)	10.84%	114.78 (91.94 to 173.45)	106.69 (66.23 to 148.87)	0 (0 to 0)	-7.05%
Northern Mariana Islands	0.42 (0.27 to 0.72)	0.45 (0.28 to 0.67)	0 (0 to 0)	7.76%	20.25 (14.67 to 33.36)	28.46 (16.39 to 35.64)	0 (0 to 0)	40.55%
Norway	54.21 (44.98 to 64.38)	64.92 (54.49 to 76.31)	0 (0 to 0)	19.76%	104.4 (99.93 to 108.41)	102.29 (95.23 to 109.7)	0 (0 to 0)	-2.02%
Oman	6.54 (5.26 to 7.88)	6.72 (5.53 to 8.11)	0 (0 to 0)	2.74%	6.02 (3.63 to 9.66)	6.31 (4.37 to 9.1)	0 (0 to 0)	4.86%
Pakistan	1.04 (0.76 to 1.35)	1.17 (0.82 to 1.6)	0 (0 to 0)	12.67%	17.78 (12.14 to 24.81)	21.07 (14.6 to 30.7)	0 (0 to 0)	18.52%
Palau	1.41 (0.93 to 2.05)	1.26 (0.8 to 1.76)	0 (0 to 0)	-10.97%	58.87 (42.14 to 80.1)	55.79 (38.47 to 76.97)	0 (0 to 0)	-5.24%
Palestine	5.21 (3.87 to 6.59)	5.71 (4.16 to 7.27)	0 (0 to 0)	9.59%	14.58 (9.39 to 20.2)	14.63 (8.81 to 18.86)	0 (0 to 0)	0.33%
Panama	21.38 (20.46 to 22.26)	20.8 (18.9 to 22.9)	0 (0 to 0)	-2.70%	20.25 (18.92 to 21.63)	26.85 (21.15 to 32.33)	0 (0 to 0)	32.56%
Papua New Guinea	0.4 (0.24 to 0.69)	0.43 (0.26 to 0.7)	0.28 (0.24 to 0.32)	6.59%	15.55 (9.19 to 25.13)	19.06 (11.07 to 29.47)	0 (0 to 0)	22.58%

Paraguay	0.99 (0.73 to 1.47)	1.59 (0.87 to 2.23)	0 (0 to 0)	60.59%	33.89 (25.35 to 48.2)	44.46 (28.24 to 60.23)	0 (0 to 0)	31.20%
Peru	13.98 (12.42 to 15.72)	11.28 (8.63 to 13.97)	0 (0 to 0)	-19.32%	27.88 (20.39 to 37.53)	31.54 (19.66 to 43.09)	0 (0 to 0)	13.15%
Philippines	3.23 (2.46 to 4.06)	2.5 (1.87 to 3.23)	0 (0 to 0)	-22.54%	18.59 (14.22 to 23.48)	19.5 (15.05 to 26.03)	0.18 (0.15 to 0.21)	4.87%
Poland	18.6 (15.23 to 22.53)	28.25 (22.95 to 34.34)	0 (0 to 0)	51.86%	85.39 (82.05 to 88.37)	69.05 (63.42 to 74.88)	-0.9 (-1.15 to -0.65)	-19.13%
Portugal	19.46 (16.59 to 23)	33.96 (27.37 to 41.03)	0 (0 to 0)	74.54%	53.65 (50.64 to 56.53)	49.96 (45.87 to 53.75)	0 (0 to 0)	-6.89%
Puerto Rico	4.67 (3.68 to 5.68)	5.51 (4.27 to 6.81)	0 (0 to 0)	18.05%	19.61 (18.41 to 21.01)	35.67 (29.57 to 42.51)	1.53 (1.03 to 2.03)	81.87%
Qatar	5.72 (3.56 to 7.64)	6.1 (3.74 to 8.65)	0 (0 to 0)	6.62%	13.54 (6.64 to 19.91)	8.22 (4.57 to 12.8)	0 (0 to 0)	-39.27%
Republic of Korea	3.69 (3.22 to 4.17)	6.98 (5.41 to 8.04)	0 (0 to 0)	89.29%	31.24 (22.28 to 36.6)	15.28 (9.26 to 22.47)	0 (0 to 0)	-51.08%
Republic of Moldova	23.81 (18.83 to 29.09)	25.75 (20.35 to 32)	0 (0 to 0)	8.16%	47.72 (44.63 to 50.82)	54.02 (47.55 to 61)	0 (0 to 0)	13.20%
Romania	16.85 (14.54 to 19.56)	26.03 (20.87 to 31.85)	0 (0 to 0)	54.47%	65.71 (61.73 to 69.73)	64.01 (55.83 to 72.95)	-0.11 (-0.26 to 0.04)	-2.58%
Russian Federation	17.39 (14.04 to 20.95)	26.19 (21.28 to 31.37)	0 (0 to 0)	50.59%	52.98 (51.48 to 54.31)	67.7 (62.02 to 72.86)	0 (0 to 0)	27.79%
Rwanda	5.08 (3.47 to 6.76)	4.88 (3.43 to 6.35)	0 (0 to 0)	-4.00%	49.58 (26.06 to 77.07)	38.96 (19.12 to 61.3)	0 (0 to 0)	-21.40%
Saint Kitts and Nevis	4.01 (2.99 to 4.99)	4.2 (3.14 to 5.22)	0 (0 to 0)	4.89%	9.8 (9.14 to 10.48)	33.04 (28.05 to 38.39)	4.35 (3.13 to 5.59)	237.08%

Saint Lucia	4.54 (3.56 to 5.48)	4.91 (3.75 to 6.07)	0 (0 to 0)	8.17%	21.69 (20.49 to 22.96)	25.58 (21.04 to 30.55)	0 (0 to 0)	17.95%
Saint Vincent and the Grenadines	4.41 (3.44 to 5.39)	5.23 (4.05 to 6.43)	0 (0 to 0)	18.60%	17.57 (16.16 to 19.02)	43.47 (37.9 to 49.69)	0 (0 to 0)	147.34%
Samoa	1.89 (1.17 to 3.53)	1.8 (1.06 to 3.61)	-0.23 (-0.28 to -0.17)	-5.00%	57.96 (36.9 to 99.56)	57.48 (36.22 to 104.81)	0 (0 to 0)	-0.82%
San Marino	51.31 (39.97 to 66.63)	51.89 (38.08 to 68.12)	0 (0 to 0)	1.12%	73.55 (55.33 to 105.92)	50.9 (27.53 to 78.22)	0 (0 to 0)	-30.79%
Sao Tome and Principe	2.98 (2.24 to 3.73)	3.02 (2.25 to 3.76)	0 (0 to 0)	1.40%	1.03 (0.64 to 1.33)	1.19 (0.74 to 1.61)	0 (0 to 0)	15.33%
Saudi Arabia	4.53 (3.38 to 5.8)	4.96 (4.36 to 5.72)	0 (0 to 0)	9.71%	3.27 (2.08 to 5.75)	4.97 (3.53 to 7.1)	0 (0 to 0)	51.83%
Senegal	5.47 (4.13 to 6.74)	5.51 (4.09 to 6.78)	0 (0 to 0)	0.64%	7.72 (4.22 to 10.66)	10.12 (4.12 to 15.7)	0 (0 to 0)	31.09%
Serbia	26.48 (24.44 to 28.99)	33.11 (24.9 to 41.82)	0 (0 to 0)	25.01%	93.95 (72.09 to 124.75)	79.36 (52.96 to 105.53)	0 (0 to 0)	-15.53%
Seychelles	1.29 (0.77 to 2.11)	1.72 (0.99 to 2.34)	0 (0 to 0)	32.78%	15.53 (10.89 to 32.1)	24.36 (15.36 to 30.52)	0 (0 to 0)	56.86%
Sierra Leone	3.25 (2.4 to 4.04)	3.3 (2.36 to 4.12)	0 (0 to 0)	1.56%	6.62 (3.42 to 9.15)	8.88 (3.4 to 13.37)	0 (0 to 0)	34.06%
Singapore	9.62 (8.97 to 10.46)	8.2 (6.64 to 9.92)	0 (0 to 0)	-14.71%	15.21 (14.25 to 16.08)	10.01 (9.14 to 10.87)	0 (0 to 0)	-34.17%
Slovakia	43.37 (40.59 to 47.74)	42.46 (30.58 to 53.89)	0 (0 to 0)	-2.08%	88.46 (67.33 to 126.96)	76.46 (44.06 to 105.46)	0 (0 to 0)	-13.57%
Slovenia	34.9 (32.45 to 37.5)	62.26 (49.81 to 76.37)	0 (0 to 0)	78.41%	87.4 (80.93 to 94.13)	84.04 (71.87 to 97.62)	0 (0 to 0)	-3.84%
Solomon Islands	0.44 (0.27 to 0.72)	0.46 (0.28 to 0.74)	0.2 (0.16 to 0.23)	5.27%	18.54 (11.43 to 30.54)	23.72 (14.84 to 36.2)	0.9 (0.84 to 0.96)	27.97%
Somalia	4.72 (3.42 to 6.06)	4.57 (3.36 to 5.91)	0 (0 to 0)	-3.04%	34.3 (18.88 to	34 (19.56 to 52.85)	0 (0 to 0)	-0.88%

					51.15)			
South Africa	24.84 (18.87 to 30.77)	32.81 (24.73 to 40.65)	0 (0 to 0)	32.08%	39.81 (30.09 to 54.25)	50.02 (31.46 to 62.72)	0 (0 to 0)	25.64%
South Sudan	4.79 (3.41 to 6.09)	4.81 (3.44 to 6.26)	0 (0 to 0)	0.36%	30.72 (16.33 to 45.29)	33.06 (17.28 to 52.14)	0 (0 to 0)	7.62%
Spain	59.21 (56.31 to 61.86)	55.02 (44.26 to 67.03)	0 (0 to 0)	-7.08%	56.83 (53.46 to 59.85)	47.54 (43.27 to 51.43)	0 (0 to 0)	-16.35%
Sri Lanka	1.3 (1.06 to 1.63)	1.11 (0.76 to 1.5)	-0.35 (-0.68 to -0.03)	-14.70%	29.73 (22.45 to 37.23)	18.22 (11.22 to 28.61)	0 (0 to 0)	-38.71%
Sudan	5.42 (3.87 to 7.1)	5.81 (4.04 to 7.51)	0 (0 to 0)	7.02%	7.85 (2.48 to 16.95)	6.34 (2.13 to 11.5)	0 (0 to 0)	-19.23%
Suriname	4.25 (3.19 to 5.31)	4.26 (3.11 to 5.39)	0 (0 to 0)	0.26%	16.51 (12.56 to 22.99)	18.99 (12.14 to 26.18)	0 (0 to 0)	15.01%
Sweden	52.33 (42.51 to 63.4)	67.44 (55.83 to 80.61)	0 (0 to 0)	28.87%	85.89 (81.11 to 91.07)	77.26 (68.66 to 88.6)	0 (0 to 0)	-10.05%
Switzerland	91.44 (87.85 to 94.94)	68.89 (56.27 to 82.69)	0 (0 to 0)	-24.66%	66.55 (62.32 to 70.76)	59.11 (53.26 to 65.08)	0 (0 to 0)	-11.18%
Syrian Arab Republic	5.16 (3.9 to 6.5)	5.89 (4.27 to 7.46)	0 (0 to 0)	14.34%	3.27 (2.36 to 5.03)	3.91 (2.17 to 5.66)	0 (0 to 0)	19.54%
Taiwan (Province of China)	3.23 (2.9 to 3.51)	1.32 (1.11 to 1.59)	-4.1 (-4.66 to -3.54)	-59.09%	26.37 (25.02 to 27.81)	21.01 (19.17 to 22.57)	0 (0 to 0)	-20.32%
Tajikistan	23.92 (18.41 to 29.93)	24.05 (18.51 to 29.86)	0 (0 to 0)	0.56%	26.92 (18.12 to 39.11)	20.72 (13.34 to 31.65)	-1.09 (-1.25 to -0.93)	-23.06%
Thailand	6.84 (6.27 to 7.63)	3.52 (2.66 to 4.57)	-1.86 (-2.44 to -1.29)	-48.57%	22.69 (17.46 to 33.23)	20.51 (14.53 to 31.12)	0 (0 to 0)	-9.57%
Timor-Leste	1.22 (0.89 to 1.6)	1.26 (0.92 to 1.66)	0.12 (0.1 to 0.14)	3.69%	13.33 (8.49 to 20.22)	17.1 (10.87 to 24.89)	0 (0 to 0)	28.26%

Togo	3.21 (2.36 to 3.99)	3.24 (2.3 to 4.07)	0 (0 to 0)	0.90%	7.61 (4.12 to 10.18)	10.2 (3.91 to 15.44)	0.94 (0.81 to 1.07)	33.98%
Tokelau	0.46 (0.29 to 0.78)	0.48 (0.29 to 0.82)	0 (0 to 0)	4.29%	20.95 (14.87 to 32)	22.81 (15.81 to 33.91)	0 (0 to 0)	8.91%
Tonga	0.46 (0.28 to 0.82)	0.49 (0.29 to 0.85)	0.27 (0.2 to 0.34)	7.52%	59.17 (43.47 to 82.03)	62.5 (45.73 to 87.61)	0 (0 to 0)	5.63%
Trinidad and Tobago	4.04 (3.43 to 4.66)	4.58 (3.4 to 5.75)	0 (0 to 0)	13.18%	14.82 (13.97 to 15.66)	14.69 (11.24 to 18.53)	0 (0 to 0)	-0.88%
Tunisia	7.04 (5.55 to 8.89)	7 (5.06 to 9.06)	0 (0 to 0)	-0.56%	3.42 (2.23 to 5.3)	3.8 (1.97 to 7.08)	0 (0 to 0)	11.18%
Turkmenistan	24.11 (18.9 to 29.19)	24 (18.69 to 28.94)	0 (0 to 0)	-0.44%	26.74 (23.82 to 29.82)	39.09 (29.98 to 51.89)	1.09 (0.82 to 1.36)	46.20%
Tuvalu	0.42 (0.25 to 0.71)	0.46 (0.29 to 0.72)	0 (0 to 0)	8.72%	20.12 (13.84 to 30.31)	24.5 (16.99 to 34.1)	0 (0 to 0)	21.77%
Türkiye	15.84 (12.91 to 18.62)	15.98 (10.85 to 20.31)	0 (0 to 0)	0.87%	36.09 (22.28 to 52.38)	30.01 (18.56 to 40.33)	0 (0 to 0)	-16.86%
Uganda	7.7 (6.3 to 9.41)	7.93 (5.87 to 10.13)	0 (0 to 0)	3.06%	44.83 (28.78 to 66.82)	63.01 (33 to 95.37)	0 (0 to 0)	40.54%
Ukraine	25.12 (19.76 to 31.17)	27.54 (21.31 to 34.45)	0 (0 to 0)	9.67%	57.82 (50.4 to 67.09)	75.17 (54.35 to 98.51)	0 (0 to 0)	30.00%
United Arab Emirates	7.61 (5.36 to 10.51)	10.19 (7.32 to 13.33)	0 (0 to 0)	34.03%	31.56 (20.02 to 49.27)	26.8 (18.8 to 36.2)	0 (0 to 0)	-15.07%
United Kingdom	55.02 (45.81 to 64.82)	62.92 (52.41 to 74.1)	0 (0 to 0)	14.36%	68.79 (67 to 70.58)	77.57 (73.73 to 81.22)	0 (0 to 0)	12.76%
United Republic of Tanzania	4.87 (3.48 to 6.08)	4.85 (3.35 to 6.33)	0 (0 to 0)	-0.36%	37.53 (21.44 to 51.49)	35.45 (16.91 to 55.5)	0 (0 to 0)	-5.54%
United States Virgin	5.84 (4.35 to 7.85)	5.92 (4.19 to 7.84)	0 (0 to 0)	1.32%	43.7 (32.3 to 69.36)	38.05 (24.82 to	0 (0 to 0)	-12.92%

Islands						54.6)		
United States of America	362.66 (290.46 to 439.66)	813.53 (736.54 to 892.64)	0 (0 to 0)	124.32%	105.02 (100.28 to 110.57)	79.89 (72.62 to 88.6)	0 (0 to 0)	-23.92%
Uruguay	29.71 (25.65 to 33.94)	33.54 (26.84 to 40.75)	0 (0 to 0)	12.89%	41.66 (38.6 to 44.74)	57.7 (52.85 to 62.51)	0 (0 to 0)	38.52%
Uzbekistan	23.89 (18.53 to 29.24)	24.26 (18.78 to 29.58)	0 (0 to 0)	1.54%	16.47 (11.97 to 21.33)	23.26 (18.47 to 28.35)	0 (0 to 0)	41.18%
Vanuatu	0.5 (0.3 to 0.86)	0.52 (0.33 to 0.8)	0 (0 to 0)	3.89%	19.58 (12.36 to 32.48)	23.65 (15.69 to 34.87)	0 (0 to 0)	20.81%
Venezuela (Bolivarian Republic of)	29.11 (22.91 to 35.43)	29.62 (23.65 to 36.06)	0 (0 to 0)	1.72%	42.58 (40.23 to 44.98)	50.08 (38.7 to 64.06)	0 (0 to 0)	17.60%
Viet Nam	2.59 (2.15 to 3.03)	1.67 (1.19 to 2.18)	-2.07 (-2.41 to -1.74)	-35.50%	16.58 (10.84 to 22.83)	20.61 (13.09 to 27.2)	0 (0 to 0)	24.36%
Yemen	5.12 (3.64 to 6.73)	5.51 (3.82 to 7.15)	0 (0 to 0)	7.70%	6.61 (2.22 to 13.61)	6.76 (2.09 to 13.23)	0 (-0.05 to 0.06)	2.26%
Zambia	4.93 (3.52 to 6.22)	5.75 (3.45 to 8.32)	0 (0 to 0)	16.64%	38.15 (21.59 to 52.07)	60.01 (19.55 to 106.52)	0 (0 to 0)	57.30%
Zimbabwe	15.78 (12.13 to 19.5)	15.01 (10.86 to 18.68)	0 (0 to 0)	-4.89%	44.27 (28.29 to 67.06)	72.7 (39.26 to 105.5)	0 (0 to 0)	64.22%

Total skin cancer includes malignant skin melanoma, non-melanoma skin cancer (basal-cell carcinoma) and non-melanoma skin cancer (squamous-cell carcinoma). DALYs, disability-adjusted life-years; EAPC, estimated annual percentage change; UI, uncertainty interval; CI, confidence interval.

TABLE S10 Incidence and DALYs of malignant skin melanoma in 1990 and 2021 across 204 countries and territories, with age-standardized rates, 95% uncertainty intervals, and trends from 1990 to 2021.

Location name	Incidence				DALYs			
	Age-standardized rate (95% UI), 1990	Age-standardized rate (95% UI), 2021	EAPC (95% CI), 1990-2021	Change in age-standardized rate,1990-2021	Age-standardized rate (95% UI), 1990	Age-standardized rate (95% UI), 2021	EAPC (95% CI), 1990-2021	Change in age-standardized rate, 1990-2021
Afghanistan	0.58 (0.18 to 1.32)	0.87 (0.27 to 1.91)	0 (0 to 0)	49.19%	10.62 (3.21 to 24.71)	10.84 (3.28 to 24.14)	0 (0 to 0)	2.14%
Albania	1.3 (0.95 to 1.88)	2.45 (1.41 to 3.7)	2.67 (2.28 to 3.06)	88.72%	23.3 (17.12 to 33.54)	21.39 (12.21 to 31.51)	0 (0 to 0)	-8.21%
Algeria	0.27 (0.18 to 0.35)	0.67 (0.34 to 0.95)	0 (0 to 0)	150.17%	2.62 (1.8 to 3.43)	2.77 (1.47 to 3.93)	0 (0 to 0)	5.53%
American Samoa	0.44 (0.31 to 0.76)	0.56 (0.34 to 0.74)	0 (0 to 0)	27.35%	10.03 (6.89 to 17.91)	12.94 (7.88 to 17.23)	1.48 (0.98 to 1.97)	29.05%
Andorra	9.78 (6.22 to 15.78)	11.45 (6.81 to 16.9)	0 (0 to 0)	17.05%	61.56 (39.8 to 97.63)	40.95 (24.26 to 61.03)	-1.04 (-1.21 to -0.86)	-33.47%
Angola	0.49 (0.33 to 0.73)	0.64 (0.4 to 0.99)	0 (0 to 0)	31.59%	12.98 (9.01 to 19.7)	14.55 (9.13 to 22.8)	0 (0 to 0)	12.05%
Antigua and Barbuda	1.11 (1.03 to 1.19)	1.92 (1.81 to 2.04)	0 (0 to 0)	72.28%	19.29 (17.91 to 20.52)	23.13 (21.89 to 24.35)	0 (0 to 0)	19.87%
Argentina	1.38 (1.3 to 1.46)	2.59 (2.41 to 2.81)	0 (0 to 0)	88.39%	25 (23.53 to 26.47)	29.97 (28.04 to 32.33)	0 (0 to 0)	19.88%
Armenia	0.41 (0.34 to 0.49)	0.87 (0.71 to 1.03)	2.86 (2.5 to 3.21)	112.91%	6.52 (5.53 to 7.79)	9.06 (7.48 to 10.7)	0 (0 to 0)	38.92%
Australia	29.94 (28.14 to 31.9)	31.18 (28.2 to 34.2)	0 (0 to 0)	4.13%	205.98 (193.14 to 217.6)	107.18 (97.26 to 118.38)	0 (0 to 0)	-47.97%

Austria	7.72 (7.21 to 8.24)	12.56 (11.5 to 13.67)	0 (0 to 0)	62.65%	70.64 (66.66 to 74.62)	60.34 (55.85 to 64.69)	-0.13 (-0.35 to 0.1)	-14.58%
Azerbaijan	0.72 (0.46 to 1.03)	0.78 (0.48 to 1.17)	0.42 (0.19 to 0.66)	8.30%	13.95 (9.22 to 20.02)	10.9 (6.49 to 17.12)	0 (0 to 0)	-21.91%
Bahamas	1.14 (1.05 to 1.23)	1.66 (1.33 to 2.05)	0 (0 to 0)	45.15%	23.03 (21.34 to 25.07)	26.51 (21.12 to 33.22)	0 (0 to 0)	15.11%
Bahrain	0.54 (0.35 to 0.71)	1.1 (0.56 to 1.56)	0 (0 to 0)	103.75%	3.97 (2.69 to 5.14)	3.14 (1.59 to 4.65)	0 (0 to 0)	-20.85%
Bangladesh	0.24 (0.17 to 0.36)	0.31 (0.16 to 0.58)	0 (0 to 0)	30.15%	6.35 (4.47 to 9.63)	5.55 (2.79 to 10.42)	0 (0 to 0)	-12.59%
Barbados	0.66 (0.62 to 0.71)	1.08 (0.85 to 1.35)	0 (0 to 0)	62.18%	11.38 (10.71 to 12.05)	13.19 (10.35 to 16.5)	0 (0 to 0)	15.91%
Belarus	2.11 (1.92 to 2.35)	8.52 (6.87 to 10.49)	0 (0 to 0)	302.88%	27.47 (25.05 to 30.3)	58.95 (47.23 to 73.14)	0 (0 to 0)	114.61%
Belgium	6.66 (6.13 to 7.23)	13.11 (11.86 to 14.52)	0 (0 to 0)	96.71%	48.93 (46.04 to 52.19)	53.77 (49.11 to 59.19)	0 (0 to 0)	9.89%
Belize	0.28 (0.27 to 0.3)	0.57 (0.5 to 0.65)	0 (0 to 0)	101.25%	5.89 (5.53 to 6.27)	9.65 (8.37 to 10.98)	0 (0 to 0)	63.79%
Benin	0.2 (0.12 to 0.25)	0.24 (0.1 to 0.34)	0 (0 to 0)	20.74%	5.07 (3.08 to 6.44)	5.05 (2.13 to 7.27)	0 (0 to 0)	-0.44%
Bermuda	4.24 (3.9 to 4.63)	7.75 (6.42 to 9.47)	0 (0 to 0)	82.64%	57.55 (53.13 to 62.49)	47.38 (39.83 to 57.98)	-0.97 (-1.32 to -0.63)	-17.69%
Bhutan	0.23 (0.14 to 0.38)	0.3 (0.16 to 0.59)	0 (0 to 0)	29.52%	6.17 (3.8 to 10.19)	5.68 (2.99 to 11.16)	0 (0 to 0)	-8.03%
Bolivia (Plurinational State of)	1.38 (0.83 to 2.47)	1.63 (0.98 to 2.52)	0 (0 to 0)	18.04%	33.76 (20.31 to 60.37)	30.44 (17.97 to 46.72)	0 (0 to 0)	-9.85%
Bosnia and Herzegovina	2.34 (1.84 to 3.39)	5.01 (2.6 to 6.63)	0 (0 to 0)	114.48%	37.67 (29.74 to 55.95)	43.58 (22.55 to 57.99)	0 (0 to 0)	15.69%
Botswana	1.46 (0.98 to 2.46)	1.56 (0.86 to 2.81)	0 (0 to 0)	6.49%	34.64 (23.31 to 58.12)	31.12 (16.26 to 56.79)	0 (0 to 0)	-10.16%

Brazil	1.27 (1.22 to 1.32)	1.93 (1.81 to 2.03)	0 (0 to 0)	52.20%	27.21 (26.32 to 28.2)	27.11 (25.69 to 28.3)	0 (0 to 0)	-0.36%
Brunei Darussalam	0.75 (0.53 to 1.15)	1.02 (0.69 to 1.4)	0 (0 to 0)	36.59%	14.25 (9.95 to 22.13)	13.46 (8.96 to 19.2)	0 (0 to 0)	-5.55%
Bulgaria	2.52 (2.21 to 2.92)	6.08 (4.91 to 7.38)	0 (0 to 0)	141.70%	32.68 (28.62 to 37.74)	50.75 (41.5 to 61.64)	0 (0 to 0)	55.30%
Burkina Faso	0.24 (0.14 to 0.32)	0.28 (0.13 to 0.39)	0 (0 to 0)	16.28%	6.04 (3.5 to 7.97)	6.08 (2.86 to 8.75)	0 (0 to 0)	0.65%
Burundi	1.31 (0.72 to 2.06)	1.03 (0.62 to 1.44)	0 (0 to 0)	-21.55%	37.49 (20.09 to 59.2)	26.81 (15.86 to 37.59)	0 (0 to 0)	-28.50%
Cabo Verde	0.07 (0.04 to 0.18)	0.41 (0.1 to 0.62)	0 (0 to 0)	487.83%	1.4 (0.81 to 3.49)	5.22 (1.34 to 7.79)	0 (0 to 0)	273.97%
Cambodia	0.19 (0.13 to 0.3)	0.3 (0.19 to 0.44)	1.44 (1.27 to 1.62)	55.64%	5.38 (3.41 to 8.37)	7.41 (4.69 to 11.18)	0 (0 to 0)	37.78%
Cameroon	0.24 (0.14 to 0.32)	0.31 (0.13 to 0.46)	0 (0 to 0)	26.16%	6.15 (3.46 to 8.07)	6.45 (2.86 to 9.68)	0 (0 to 0)	4.93%
Canada	8.01 (7.47 to 8.61)	10.63 (9.71 to 11.54)	0 (0 to 0)	32.66%	60.54 (57.2 to 63.73)	47.52 (43.86 to 51.01)	0 (0 to 0)	-21.51%
Central African Republic	0.55 (0.38 to 0.92)	0.53 (0.36 to 0.85)	0 (0 to 0)	-3.80%	15.02 (9.87 to 24.65)	14.01 (9 to 22.79)	-0.25 (-0.3 to -0.19)	-6.76%
Chad	0.17 (0.11 to 0.23)	0.23 (0.12 to 0.33)	0 (0 to 0)	31.45%	4.56 (2.74 to 5.91)	5.47 (3.01 to 8.07)	0 (0 to 0)	19.97%
Chile	1.61 (1.51 to 1.73)	3.58 (3.28 to 3.92)	0 (0 to 0)	121.71%	26.58 (24.87 to 28.41)	26.9 (24.66 to 29.27)	-0.08 (-0.29 to 0.12)	1.19%
China	0.36 (0.24 to 0.46)	0.68 (0.37 to 0.91)	2.27 (2.05 to 2.48)	89.21%	8.99 (5.74 to 11.23)	7.8 (4.21 to 10.37)	0 (0 to 0)	-13.25%
Colombia	0.97 (0.91 to 1.04)	1.85 (1.55 to 2.2)	0 (0 to 0)	90.89%	19.53 (18.38 to 20.77)	20.27 (16.91 to 24.2)	0 (0 to 0)	3.77%
Comoros	1.04 (0.55 to 1.43)	1.1 (0.59 to 1.67)	0 (0 to 0)	6.06%	28.43 (14.59 to 39.73)	26.08 (13.83 to 40.35)	0 (0 to 0)	-8.26%

Congo	0.6 (0.43 to 0.97)	0.72 (0.48 to 1.1)	0 (0 to 0)	19.38%	15.49 (10.8 to 25.17)	15.57 (10.2 to 24.07)	-0.05 (-0.17 to 0.08)	0.54%
Cook Islands	0.33 (0.21 to 0.45)	0.38 (0.25 to 0.56)	0 (0 to 0)	13.85%	7.23 (4.58 to 10.19)	6.85 (4.62 to 10.29)	0.01 (-0.23 to 0.24)	-5.30%
Costa Rica	1.65 (1.51 to 1.77)	3 (2.6 to 3.39)	0 (0 to 0)	81.76%	24.62 (22.87 to 26.3)	29.6 (25.59 to 33.72)	0 (0 to 0)	20.21%
Croatia	4.7 (4.21 to 5.3)	10.34 (8.68 to 12.19)	0 (0 to 0)	120.05%	60.76 (54.39 to 67.64)	69.86 (59.27 to 81.13)	0 (0 to 0)	14.99%
Cuba	0.82 (0.77 to 0.88)	1.67 (1.43 to 1.9)	0 (0 to 0)	102.91%	11.95 (11.21 to 12.7)	16.04 (13.84 to 18.28)	0 (0 to 0)	34.21%
Cyprus	3.95 (2.93 to 6.34)	8.37 (4.29 to 11.46)	0 (0 to 0)	111.69%	39.64 (28.85 to 64.15)	34.52 (17.86 to 47.84)	0 (0 to 0)	-12.91%
Czechia	7.31 (6.63 to 7.94)	15.62 (13.41 to 18.24)	0 (0 to 0)	113.85%	72.31 (67.25 to 78.18)	64.8 (55.08 to 75.19)	0 (0 to 0)	-10.38%
Côte d'Ivoire	0.43 (0.27 to 0.55)	0.53 (0.28 to 0.78)	0 (0 to 0)	23.70%	11.09 (6.75 to 14.5)	11.86 (5.91 to 17.63)	0 (0 to 0)	6.90%
Democratic People's Republic of Korea	0.33 (0.22 to 0.56)	0.37 (0.26 to 0.56)	0.57 (0.49 to 0.66)	14.44%	9.16 (6.04 to 15.46)	9.95 (6.72 to 15.53)	0 (0 to 0)	8.61%
Democratic Republic of the Congo	0.49 (0.34 to 0.73)	0.58 (0.37 to 0.93)	0 (0 to 0)	20.04%	12.46 (8.9 to 18.76)	13.45 (8.43 to 21.55)	0 (0 to 0)	7.90%
Denmark	8.66 (8.18 to 9.21)	19.68 (17.88 to 21.41)	0 (0 to 0)	127.32%	67.97 (64.59 to 71.56)	73.07 (66.67 to 79.48)	0 (0 to 0)	7.51%
Djibouti	0.98 (0.53 to 1.41)	1.08 (0.54 to 1.74)	0 (0 to 0)	9.71%	25.8 (13.88 to 38.54)	24.68 (12.38 to 40.59)	0 (0 to 0)	-4.35%
Dominica	0.51 (0.38 to 0.73)	0.61 (0.36 to 0.82)	0 (0 to 0)	18.07%	10.17 (7.6 to 14.46)	10.92 (6.42 to 15.23)	0.21 (0.15 to 0.27)	7.40%

Dominican Republic	0.25 (0.18 to 0.35)	0.31 (0.21 to 0.45)	0 (0 to 0)	22.48%	5.71 (4.27 to 8.06)	5.54 (3.88 to 7.81)	0 (0 to 0)	-3.02%
Ecuador	0.95 (0.88 to 1.01)	1.7 (1.32 to 2.12)	0 (0 to 0)	80.05%	19.33 (18.03 to 20.66)	23.5 (18.24 to 29.36)	0.95 (0.63 to 1.28)	21.59%
Egypt	0.09 (0.07 to 0.13)	0.2 (0.13 to 0.29)	0 (0 to 0)	115.28%	1.02 (0.77 to 1.47)	1.05 (0.68 to 1.43)	0 (0 to 0)	2.54%
El Salvador	0.2 (0.15 to 0.29)	0.42 (0.24 to 0.55)	0 (0 to 0)	109.84%	4.51 (3.53 to 6.28)	5.65 (3.24 to 7.4)	0 (0 to 0)	25.39%
Equatorial Guinea	0.52 (0.35 to 0.81)	0.75 (0.38 to 1.26)	0 (0 to 0)	45.25%	13.91 (9.33 to 22.61)	13.8 (7.06 to 23.17)	0 (0 to 0)	-0.73%
Eritrea	1.2 (0.71 to 1.89)	1.28 (0.8 to 1.9)	0 (0 to 0)	6.64%	34.62 (20.05 to 54.79)	33.22 (20.21 to 49.95)	0 (0 to 0)	-4.04%
Estonia	4.35 (3.85 to 4.94)	10.81 (8.95 to 12.51)	0 (0 to 0)	148.49%	49.12 (44.14 to 55.07)	59.61 (50.5 to 68.84)	0 (0 to 0)	21.36%
Eswatini	1.54 (1.1 to 2.7)	1.93 (1.23 to 3.37)	0 (0 to 0)	25.17%	36.21 (26.3 to 63.8)	42.39 (26.63 to 75.04)	0 (0 to 0)	17.07%
Ethiopia	0.57 (0.33 to 0.91)	0.59 (0.39 to 0.8)	0 (0 to 0)	3.14%	16.37 (9.38 to 26.67)	13.66 (8.94 to 18.89)	0 (0 to 0)	-16.54%
Fiji	0.31 (0.17 to 0.72)	0.36 (0.22 to 0.72)	0.56 (0.42 to 0.69)	15.10%	7.51 (4.17 to 17.3)	8.01 (4.71 to 15.65)	0 (0 to 0)	6.61%
Finland	7.52 (7.05 to 8.01)	17.38 (15.76 to 19.13)	0 (0 to 0)	131.10%	59.94 (57.05 to 63.34)	63.98 (58.41 to 70.51)	0 (0 to 0)	6.74%
France	6.45 (6.05 to 6.88)	13.38 (12.31 to 14.62)	0 (0 to 0)	107.46%	50.16 (47.73 to 52.73)	45.5 (41.97 to 49.57)	0 (0 to 0)	-9.29%
Gabon	0.69 (0.5 to 1.08)	0.8 (0.46 to 1.25)	0 (0 to 0)	16.20%	16.81 (12.07 to 26.65)	15.54 (8.95 to 24.35)	0 (0 to 0)	-7.51%
Gambia	0.22 (0.14 to 0.3)	0.31 (0.17 to 0.43)	0 (0 to 0)	40.17%	5.53 (3.39 to 7.77)	6.86 (3.68 to 9.85)	0.39 (0.19 to 0.6)	23.97%
Georgia	3.2 (2.66 to 3.74)	2.79 (2.4 to 3.22)	-1.12 (-2.02 to	-12.96%	48.15 (40.56 to	35.47 (30.81 to	0 (0 to 0)	-26.33%

			-0.21)		55.72)	40.75)		
Germany	7.59 (7.08 to 8.13)	14.58 (13.27 to 16.04)	0 (0 to 0)	92.22%	57.35 (54.15 to 60.62)	52.44 (48.09 to 57.22)	0 (0 to 0)	-8.56%
Ghana	0.03 (0.02 to 0.04)	0.03 (0.02 to 0.06)	0 (0 to 0)	23.81%	0.68 (0.42 to 0.95)	0.69 (0.35 to 1.13)	0 (0 to 0)	1.15%
Greece	3.69 (3.46 to 3.93)	8.15 (7.57 to 8.76)	0 (0 to 0)	120.89%	23.34 (22.29 to 24.38)	38.46 (36.26 to 40.92)	0 (0 to 0)	64.78%
Greenland	2.2 (1.57 to 2.91)	2.28 (1.43 to 3.25)	0 (0 to 0)	3.91%	42.9 (30.15 to 57.68)	30.45 (18.63 to 43.98)	0 (0 to 0)	-29.01%
Grenada	0.64 (0.57 to 0.71)	1.13 (0.96 to 1.31)	0 (0 to 0)	76.92%	14.03 (12.44 to 15.65)	18.26 (15.57 to 21.37)	0 (0 to 0)	30.13%
Guam	0.13 (0.09 to 0.22)	0.13 (0.08 to 0.18)	0 (0 to 0)	0.61%	2.57 (1.87 to 4.77)	2.85 (1.88 to 4.14)	0 (0 to 0)	10.97%
Guatemala	0.33 (0.31 to 0.34)	0.52 (0.45 to 0.61)	0 (0 to 0)	61.36%	7.96 (7.65 to 8.26)	9.59 (8.24 to 11.07)	0 (0 to 0)	20.59%
Guinea	0.88 (0.63 to 1.22)	1.03 (0.62 to 1.53)	0 (0 to 0)	17.82%	23.84 (16.72 to 33.46)	26.11 (15.4 to 38.58)	0 (0 to 0)	9.50%
Guinea-Bissau	0.25 (0.15 to 0.38)	0.31 (0.16 to 0.47)	0 (0 to 0)	25.32%	6.9 (3.96 to 10.74)	7.41 (4.09 to 11.09)	0 (0 to 0)	7.32%
Guyana	0.31 (0.28 to 0.35)	0.52 (0.39 to 0.66)	0 (0 to 0)	65.90%	7.88 (6.95 to 8.89)	11.44 (8.56 to 14.6)	0 (0 to 0)	45.16%
Haiti	0.71 (0.42 to 1.28)	0.76 (0.4 to 1.27)	0 (0 to 0)	6.58%	19.47 (11.23 to 36.15)	19 (10.08 to 32.96)	0 (0 to 0)	-2.40%
Honduras	0.25 (0.16 to 0.34)	0.37 (0.22 to 0.52)	0 (0 to 0)	48.35%	6.1 (3.93 to 8.25)	7.14 (4.24 to 9.94)	0 (0 to 0)	16.91%
Hungary	4.6 (4.22 to 4.94)	8.25 (7.07 to 9.68)	0 (0 to 0)	79.16%	66.02 (61.21 to 70.47)	57.76 (49.22 to 66.88)	0 (0 to 0)	-12.51%
Iceland	7.59 (6.96 to 8.29)	18.31 (16.03 to 20.75)	0 (0 to 0)	141.39%	45.53 (42.28 to 48.71)	63.92 (56.92 to 73.18)	0 (0 to 0)	40.40%
India	0.19 (0.13 to 0.25)	0.31 (0.2 to 0.41)	0 (0 to 0)	60.16%	5.15 (3.38 to 6.66)	5.8 (3.86 to 7.75)	0 (0 to 0)	12.61%
Indonesia	0.15 (0.11 to 0.22)	0.19 (0.13 to 0.25)	0.76 (0.7 to 0.82)	28.46%	4.16 (2.93 to 6.08)	4.68 (3.12 to 6.25)	0 (0 to 0)	12.44%

Iran (Islamic Republic of)	2.07 (0.99 to 2.78)	3.67 (2.16 to 4.65)	0 (0 to 0)	77.30%	15.06 (7.06 to 20.52)	10.29 (6.11 to 12.88)	0 (0 to 0)	-31.68%
Iraq	0.45 (0.32 to 0.66)	1.02 (0.68 to 1.47)	0 (0 to 0)	125.01%	4.64 (3.34 to 6.81)	4.43 (2.95 to 6.28)	0 (0 to 0)	-4.53%
Ireland	6.87 (6.39 to 7.46)	15.58 (14.07 to 17.09)	0 (0 to 0)	126.64%	53.71 (50.31 to 57.32)	54.36 (49.41 to 59.43)	0 (0 to 0)	1.21%
Israel	7.28 (6.74 to 7.78)	12.38 (11.11 to 13.48)	0 (0 to 0)	70.20%	69.22 (64.85 to 73.41)	59.82 (54.48 to 64.17)	0 (0 to 0)	-13.58%
Italy	7.29 (6.87 to 7.71)	12.54 (11.6 to 13.52)	0 (0 to 0)	72.14%	52.44 (50.75 to 54.35)	49.36 (46.28 to 52.64)	0.06 (-0.15 to 0.27)	-5.87%
Jamaica	0.47 (0.43 to 0.51)	0.93 (0.7 to 1.24)	0 (0 to 0)	97.98%	8.1 (7.53 to 8.75)	13.03 (9.69 to 17.2)	0 (0 to 0)	60.92%
Japan	0.96 (0.9 to 1.02)	1.8 (1.67 to 1.91)	0 (0 to 0)	87.35%	6.46 (6.24 to 6.69)	6.76 (6.35 to 7.16)	0 (0 to 0)	4.54%
Jordan	0.36 (0.25 to 0.5)	0.69 (0.35 to 1.02)	0 (0 to 0)	92.12%	2.79 (1.97 to 3.74)	2.15 (1.06 to 3.14)	0 (0 to 0)	-22.88%
Kazakhstan	1.13 (0.91 to 1.45)	1.88 (1.58 to 2.21)	1.45 (1.21 to 1.69)	65.79%	20.84 (17.18 to 26.14)	21.81 (18.31 to 25.51)	0 (0 to 0)	4.62%
Kenya	0.47 (0.32 to 0.66)	0.63 (0.39 to 0.92)	0 (0 to 0)	34.71%	11.34 (7.68 to 15.66)	13.57 (8.39 to 19.87)	0 (0 to 0)	19.68%
Kiribati	0.06 (0.03 to 0.1)	0.07 (0.03 to 0.1)	0 (0 to 0)	5.81%	1.66 (0.98 to 2.6)	1.68 (0.82 to 2.61)	0 (0 to 0)	1.27%
Kuwait	0.32 (0.29 to 0.35)	0.54 (0.45 to 0.65)	0 (0 to 0)	70.76%	1.27 (1.14 to 1.4)	1.33 (1.1 to 1.63)	-0.9 (-2.95 to 1.2)	5.20%
Kyrgyzstan	1.51 (1.27 to 1.78)	0.99 (0.79 to 1.23)	-1.73 (-2.33 to -1.13)	-34.15%	31.56 (26.6 to 37.64)	13.97 (11.03 to 17.27)	0 (0 to 0)	-55.75%
Lao People's Democratic Republic	0.22 (0.13 to 0.42)	0.26 (0.17 to 0.39)	0.46 (0.4 to 0.52)	16.69%	6.46 (3.55 to 12.29)	6.82 (4.37 to 10.29)	0 (0 to 0)	5.72%
Latvia	2.94 (2.62 to 3.29)	8.35 (7.2 to 9.75)	0 (0 to 0)	183.72%	37.37 (33.17 to 41.45)	67.38 (57.82 to 78.95)	0 (0 to 0)	80.31%
Lebanon	0.88 (0.59 to 1.38)	2.04 (1.3 to 3.07)	0 (0 to 0)	130.90%	6.45 (4.24 to 9.98)	5.72 (3.74 to 8.53)	-0.12 (-0.24 to	-11.36%

							-0.01)	
Lesotho	1.19 (0.77 to 2.15)	1.68 (1.07 to 2.97)	0 (0 to 0)	41.22%	27.99 (18.01 to 51.24)	39.74 (24.42 to 74.61)	1.54 (1.28 to 1.8)	42.00%
Liberia	0.2 (0.12 to 0.26)	0.27 (0.12 to 0.4)	0 (0 to 0)	36.67%	5.15 (2.91 to 6.77)	5.58 (2.46 to 8.34)	0 (0 to 0)	8.39%
Libya	0.29 (0.18 to 0.39)	0.55 (0.32 to 0.78)	0 (0 to 0)	92.11%	2.38 (1.49 to 3.25)	2.52 (1.47 to 3.6)	0 (0 to 0)	5.73%
Lithuania	2.75 (2.43 to 3.06)	9.49 (8.03 to 11.05)	0 (0 to 0)	245.12%	28.92 (25.86 to 32)	63.63 (54.37 to 73.57)	0 (0 to 0)	120.03%
Luxembourg	7.02 (6.51 to 7.54)	12.58 (11.15 to 14.18)	0 (0 to 0)	79.31%	59.6 (56.53 to 62.91)	48.8 (43.94 to 54.6)	0 (0 to 0)	-18.12%
Madagascar	1.04 (0.64 to 1.43)	0.96 (0.56 to 1.47)	0 (0 to 0)	-7.76%	28.72 (17.4 to 39.2)	24.19 (14 to 37.78)	-0.64 (-0.74 to -0.55)	-15.79%
Malawi	1.92 (1.28 to 2.59)	2.43 (1.28 to 3.66)	0 (0 to 0)	26.46%	53.27 (34.59 to 72.57)	58.54 (30.46 to 86.81)	0 (0 to 0)	9.89%
Malaysia	0.38 (0.23 to 0.49)	0.45 (0.26 to 0.57)	0.46 (0.34 to 0.59)	16.28%	9.74 (5.69 to 12.43)	9.36 (5.27 to 12.08)	0 (0 to 0)	-3.91%
Maldives	0.1 (0.06 to 0.17)	0.09 (0.06 to 0.13)	-0.39 (-0.48 to -0.31)	-9.61%	2.6 (1.5 to 4.44)	1.78 (1.08 to 2.72)	0 (0 to 0)	-31.65%
Mali	0.8 (0.53 to 1.07)	0.86 (0.44 to 1.32)	0 (0 to 0)	8.18%	21.61 (14.01 to 28.14)	20.53 (10.05 to 30.77)	0 (0 to 0)	-4.97%
Malta	3.56 (3.27 to 3.88)	8.32 (7.35 to 9.45)	0 (0 to 0)	133.92%	29.59 (27.46 to 31.87)	35.38 (31.6 to 39.49)	0 (0 to 0)	19.59%
Marshall Islands	0.32 (0.2 to 0.55)	0.36 (0.22 to 0.56)	0.47 (0.38 to 0.56)	12.01%	8.38 (5.07 to 14.95)	9.11 (5.63 to 14.43)	0 (0 to 0)	8.68%
Mauritania	0.22 (0.12 to 0.3)	0.3 (0.13 to 0.44)	0 (0 to 0)	38.07%	5.44 (3.14 to 7.6)	5.36 (2.32 to 8.05)	0 (0 to 0)	-1.44%
Mauritius	0.03 (0.02 to 0.03)	0.26 (0.25 to 0.28)	0 (0 to 0)	928.69%	0.6 (0.57 to 0.63)	5.53 (5.12 to 5.81)	0 (0 to 0)	822.38%
Mexico	0.63 (0.62 to 0.65)	1.38 (1.22 to 1.54)	0 (0 to 0)	117.78%	12.95 (12.68 to	19.47 (17.22 to	0 (0 to 0)	50.28%

					13.2)	21.79)		
Micronesia (Federated States of)	0.34 (0.2 to 0.6)	0.33 (0.21 to 0.52)	0 (0 to 0)	-0.32%	8.86 (5.11 to 16.07)	8.51 (5.3 to 13.21)	0 (0 to 0)	-3.86%
Monaco	7.77 (5.36 to 11.72)	15.08 (8.46 to 20.76)	0 (0 to 0)	94.13%	44.48 (30.94 to 68.82)	58.4 (32.63 to 80.04)	0 (0 to 0)	31.28%
Mongolia	0.59 (0.3 to 0.84)	0.6 (0.37 to 0.93)	-0.24 (-0.61 to 0.14)	1.88%	13.46 (6.86 to 19.25)	9.61 (6.01 to 14.41)	0 (0 to 0)	-28.62%
Montenegro	4.5 (3.21 to 6.23)	6.4 (3.74 to 8.92)	1.62 (1.43 to 1.81)	42.12%	42.64 (30.1 to 58.4)	43.68 (26.31 to 61.76)	0 (0 to 0)	2.44%
Morocco	0.53 (0.22 to 0.86)	1.26 (0.42 to 1.94)	0 (0 to 0)	137.56%	6.36 (2.66 to 10.4)	7.37 (2.52 to 11.38)	0 (0 to 0)	15.85%
Mozambique	1.09 (0.64 to 1.52)	1.3 (0.74 to 1.92)	0 (0 to 0)	19.33%	30.32 (17.19 to 42.6)	33.8 (18.93 to 50.67)	0 (0 to 0)	11.51%
Myanmar	0.2 (0.11 to 0.35)	0.23 (0.15 to 0.33)	0.36 (0.31 to 0.42)	16.10%	5.63 (3.18 to 10.52)	5.72 (3.66 to 8.45)	-0.1 (-0.17 to -0.04)	1.55%
Namibia	2.67 (1.99 to 3.73)	4.11 (2.62 to 6.28)	0 (0 to 0)	53.80%	66.45 (47.72 to 90.02)	82.8 (52.23 to 122.29)	0 (0 to 0)	24.60%
Nauru	0.4 (0.22 to 0.78)	0.38 (0.22 to 0.65)	0 (0 to 0)	-5.34%	10.18 (5.39 to 20.6)	9.55 (5.12 to 17.1)	0 (0 to 0)	-6.28%
Nepal	0.2 (0.13 to 0.33)	0.29 (0.19 to 0.53)	0 (0 to 0)	47.16%	5.3 (3.45 to 9.01)	5.85 (3.72 to 10.94)	0 (0 to 0)	10.40%
Netherlands	9.39 (8.84 to 9.97)	21.59 (19.74 to 23.24)	0 (0 to 0)	129.96%	67.09 (63.47 to 71.01)	80.86 (74.72 to 87.37)	0 (0 to 0)	20.53%
New Zealand	27.15 (24.82 to 29.54)	38.91 (35.21 to 42.43)	0 (0 to 0)	43.33%	145.98 (134.65 to 157.32)	124.64 (113.07 to 135.24)	0 (0 to 0)	-14.62%
Nicaragua	0.31 (0.23 to 0.47)	0.53 (0.31 to 0.71)	0 (0 to 0)	73.38%	6.57 (5.07 to 9.83)	7.88 (4.68 to 10.47)	0 (0 to 0)	19.91%
Niger	0.18 (0.11 to 0.23)	0.21 (0.1 to 0.3)	0 (0 to 0)	17.76%	4.78 (2.96 to 6.21)	4.81 (2.23 to 6.77)	0 (0 to 0)	0.50%
Nigeria	0.62 (0.28 to 0.93)	0.78 (0.27 to 1.19)	0 (0 to 0)	25.35%	16.51 (7.01 to 25.39)	17.07 (5.74 to 26.42)	0 (0 to 0)	3.36%

Niue	0.37 (0.24 to 0.62)	0.44 (0.29 to 0.67)	0 (0 to 0)	17.13%	9.12 (5.7 to 15.41)	10 (6.51 to 15.58)	0 (0 to 0)	9.63%
North Macedonia	5.68 (4.57 to 8.82)	9.46 (5.55 to 12.92)	0 (0 to 0)	66.55%	93.65 (75.03 to 147.68)	86.89 (50.63 to 122.74)	0 (0 to 0)	-7.22%
Northern Mariana Islands	0.27 (0.19 to 0.48)	0.3 (0.2 to 0.42)	0 (0 to 0)	12.10%	5.97 (4.09 to 10.97)	6.38 (4.14 to 9.04)	0 (0 to 0)	6.78%
Norway	13.03 (12.33 to 13.76)	22.97 (21.21 to 24.75)	0 (0 to 0)	76.25%	97.74 (93.68 to 101.43)	94.59 (88.4 to 101.38)	0 (0 to 0)	-3.23%
Oman	0.25 (0.16 to 0.44)	0.59 (0.37 to 0.99)	0 (0 to 0)	131.86%	2.17 (1.42 to 3.77)	1.89 (1.15 to 3.17)	0 (0 to 0)	-12.93%
Pakistan	0.4 (0.28 to 0.56)	0.54 (0.34 to 0.8)	0 (0 to 0)	33.57%	10.66 (7.53 to 14.64)	12.37 (7.9 to 18.7)	0 (0 to 0)	15.99%
Palau	1.27 (0.85 to 1.81)	1.11 (0.72 to 1.51)	0 (0 to 0)	-12.22%	27.6 (18.8 to 38.36)	24.16 (15.54 to 32.92)	0 (0 to 0)	-12.49%
Palestine	0.57 (0.38 to 0.88)	1.01 (0.64 to 1.43)	0 (0 to 0)	75.53%	4.85 (3.24 to 7.19)	4.11 (2.65 to 5.93)	0 (0 to 0)	-15.26%
Panama	0.63 (0.59 to 0.67)	1.64 (1.29 to 1.99)	0 (0 to 0)	160.08%	11.09 (10.48 to 11.66)	18.78 (14.8 to 22.65)	0 (0 to 0)	69.43%
Papua New Guinea	0.26 (0.16 to 0.44)	0.28 (0.18 to 0.45)	0.41 (0.35 to 0.47)	10.39%	6.61 (4.06 to 11.43)	7.28 (4.46 to 11.73)	0 (0 to 0)	10.22%
Paraguay	0.87 (0.67 to 1.27)	1.48 (0.81 to 2.06)	0 (0 to 0)	70.81%	17.73 (13.54 to 26.36)	22.37 (12.39 to 31.06)	0 (0 to 0)	26.11%
Peru	0.8 (0.58 to 1.08)	1.34 (0.85 to 1.89)	0 (0 to 0)	67.82%	17.08 (12.45 to 23.23)	15.43 (10 to 21.95)	0 (0 to 0)	-9.69%
Philippines	0.26 (0.18 to 0.35)	0.29 (0.2 to 0.42)	0 (0 to 0)	10.56%	6.55 (4.68 to 9)	7.06 (5.03 to 10.44)	0.31 (0.26 to 0.36)	7.85%
Poland	2.83 (2.7 to 2.95)	6.55 (5.97 to 7.18)	0 (0 to 0)	131.04%	55.9 (54.2 to 57.62)	66.43 (61.06 to 72.02)	0.55 (0.32 to 0.79)	18.85%
Portugal	2.51 (2.35 to 2.68)	6.57 (6 to 7.15)	0 (0 to 0)	162.02%	31.57 (29.86 to	34.3 (31.67 to	0 (0 to 0)	8.64%

					33.32)	36.86)		
Puerto Rico	0.98 (0.91 to 1.06)	1.86 (1.55 to 2.25)	0 (0 to 0)	90.20%	14.2 (13.28 to 15.28)	15.27 (12.68 to 18.31)	-0.3 (-0.66 to 0.07)	7.54%
Qatar	1.82 (0.81 to 2.51)	2.19 (1.08 to 3.51)	0 (0 to 0)	19.86%	11.2 (4.98 to 15.6)	5.3 (2.62 to 8.58)	0 (0 to 0)	-52.69%
Republic of Korea	0.59 (0.39 to 0.78)	1.9 (0.78 to 2.5)	0 (0 to 0)	220.43%	8.8 (5.64 to 11.18)	7.52 (3.07 to 9.94)	0 (0 to 0)	-14.57%
Republic of Moldova	1.68 (1.57 to 1.81)	3.5 (3.05 to 3.96)	0 (0 to 0)	107.61%	27.24 (25.68 to 28.9)	36.29 (31.82 to 40.96)	0 (0 to 0)	33.19%
Romania	1.78 (1.66 to 1.92)	4.64 (3.99 to 5.35)	0 (0 to 0)	160.05%	31.76 (29.53 to 33.98)	41.22 (35.82 to 47.38)	0.93 (0.71 to 1.15)	29.79%
Russian Federation	2.66 (2.59 to 2.74)	6.59 (6.03 to 7.12)	0 (0 to 0)	147.61%	37.5 (36.46 to 38.51)	52.56 (48.06 to 56.62)	0 (0 to 0)	40.17%
Rwanda	1.43 (0.8 to 2.21)	1.27 (0.72 to 1.92)	0 (0 to 0)	-10.74%	41.81 (23.23 to 65.39)	29.72 (16.44 to 45.3)	0 (0 to 0)	-28.91%
Saint Kitts and Nevis	0.31 (0.29 to 0.33)	0.42 (0.35 to 0.5)	0 (0 to 0)	36.78%	7.1 (6.62 to 7.58)	6.4 (5.36 to 7.63)	-0.44 (-0.75 to -0.12)	-9.82%
Saint Lucia	0.93 (0.88 to 0.98)	1.13 (0.93 to 1.36)	0 (0 to 0)	22.04%	18.67 (17.69 to 19.72)	16.75 (13.75 to 19.99)	0 (0 to 0)	-10.27%
Saint Vincent and the Grenadines	0.76 (0.7 to 0.83)	1.31 (1.13 to 1.51)	0 (0 to 0)	71.32%	14.88 (13.66 to 16.11)	20.88 (18.06 to 24.14)	0 (0 to 0)	40.38%
Samoa	1.73 (1.08 to 3.27)	1.65 (0.98 to 3.37)	-0.22 (-0.28 to -0.16)	-4.66%	45.39 (28.32 to 81.78)	41.54 (24.97 to 82.21)	0 (0 to 0)	-8.48%
San Marino	11.94 (8.76 to 18.05)	12.23 (6.58 to 18.94)	0 (0 to 0)	2.46%	65.09 (48.68 to 94.8)	45.5 (24.16 to 70.34)	0 (0 to 0)	-30.09%
Sao Tome and Principe	0.02 (0.01 to 0.03)	0.03 (0.02 to 0.05)	0 (0 to 0)	45.77%	0.51 (0.27 to 0.68)	0.6 (0.33 to 0.82)	0 (0 to 0)	16.21%
Saudi Arabia	0.13 (0.07 to 0.26)	0.47 (0.3 to 0.8)	0 (0 to 0)	267.28%	1.35 (0.79 to 2.63)	1.62 (1.05 to 2.76)	0 (0 to 0)	20.29%

Senegal	0.21 (0.12 to 0.29)	0.28 (0.13 to 0.4)	0 (0 to 0)	31.59%	5.47 (3.11 to 7.47)	5.87 (2.75 to 8.48)	0 (0 to 0)	7.26%
Serbia	3.75 (2.82 to 5.18)	6.34 (3.82 to 8.73)	0 (0 to 0)	68.96%	62.38 (46.89 to 86.02)	56.4 (34.43 to 76.3)	0 (0 to 0)	-9.59%
Seychelles	0.36 (0.22 to 0.8)	0.71 (0.4 to 0.91)	0 (0 to 0)	97.98%	9.06 (5.6 to 20.18)	15.26 (8.74 to 19.55)	0 (0 to 0)	68.46%
Sierra Leone	0.19 (0.11 to 0.25)	0.24 (0.11 to 0.34)	0 (0 to 0)	29.47%	4.74 (2.6 to 6.36)	5.26 (2.43 to 7.41)	0 (0 to 0)	10.85%
Singapore	0.59 (0.55 to 0.64)	1.5 (1.37 to 1.63)	0 (0 to 0)	152.17%	6.69 (6.28 to 7.13)	6.31 (5.82 to 6.86)	0 (0 to 0)	-5.69%
Slovakia	5.77 (4.48 to 8.76)	10.27 (5.36 to 14.05)	0 (0 to 0)	78.10%	70.06 (53.47 to 105.28)	64.73 (34.6 to 89.5)	0 (0 to 0)	-7.61%
Slovenia	7.66 (7 to 8.37)	19.29 (16.33 to 22.75)	0 (0 to 0)	151.97%	71.53 (66.22 to 77.03)	76.99 (65.82 to 89.5)	0 (0 to 0)	7.64%
Solomon Islands	0.29 (0.19 to 0.48)	0.32 (0.2 to 0.5)	0.29 (0.24 to 0.34)	7.94%	7.64 (4.76 to 12.99)	8.33 (5.13 to 13.22)	0.35 (0.3 to 0.4)	9.01%
Somalia	1 (0.61 to 1.43)	0.96 (0.64 to 1.41)	0 (0 to 0)	-3.54%	28.59 (17.07 to 41.16)	26.67 (17.77 to 39.84)	0 (0 to 0)	-6.71%
South Africa	1.29 (0.91 to 1.67)	1.81 (0.99 to 2.3)	0 (0 to 0)	40.98%	27.89 (21.01 to 37.72)	31.95 (18.5 to 42.12)	0 (0 to 0)	14.55%
South Sudan	0.92 (0.55 to 1.27)	1 (0.62 to 1.46)	0 (0 to 0)	8.58%	24.83 (14.45 to 34.91)	24.64 (15.1 to 37.13)	0 (0 to 0)	-0.74%
Spain	4.59 (4.3 to 4.89)	9.32 (8.43 to 10.22)	0 (0 to 0)	103.29%	36.02 (34.14 to 37.87)	35.15 (32.07 to 38.02)	0 (0 to 0)	-2.43%
Sri Lanka	0.15 (0.11 to 0.25)	0.24 (0.12 to 0.35)	2.28 (1.94 to 2.63)	62.79%	3.57 (2.7 to 6.16)	4.35 (2.23 to 6.51)	0 (0 to 0)	21.82%
Sudan	0.53 (0.18 to 1.07)	0.82 (0.27 to 1.34)	0 (0 to 0)	54.27%	7.73 (2.45 to 16.07)	6.09 (2.04 to 9.86)	0 (0 to 0)	-21.27%
Suriname	0.47 (0.35 to 0.64)	0.54 (0.33 to 0.74)	0 (0 to 0)	13.92%	10.78 (7.94 to 14.49)	10.48 (6.43 to 14.64)	0 (0 to 0)	-2.80%

Sweden	10.89 (10.19 to 11.65)	17.33 (15.21 to 19.48)	0 (0 to 0)	59.16%	77.68 (73.36 to 82.41)	69.56 (61.88 to 79.96)	0 (0 to 0)	-10.45%
Switzerland	9.25 (8.62 to 9.96)	15.11 (13.5 to 16.71)	0 (0 to 0)	63.39%	57.25 (53.62 to 60.87)	50.35 (45.51 to 55.53)	0 (0 to 0)	-12.05%
Syrian Arab Republic	0.34 (0.25 to 0.54)	1.07 (0.58 to 1.53)	0 (0 to 0)	215.85%	3.2 (2.31 to 4.94)	3.84 (2.12 to 5.57)	0 (0 to 0)	19.93%
Taiwan (Province of China)	0.52 (0.49 to 0.55)	0.71 (0.64 to 0.77)	1.07 (0.7 to 1.44)	37.28%	10.96 (10.32 to 11.68)	11.65 (10.56 to 12.48)	0 (0 to 0)	6.31%
Tajikistan	0.78 (0.53 to 1.06)	0.63 (0.42 to 0.9)	0 (0 to 0)	-18.58%	15.3 (10.98 to 21.1)	11.62 (7.5 to 17.82)	-1.17 (-1.29 to -1.05)	-24.02%
Thailand	0.19 (0.13 to 0.37)	0.22 (0.13 to 0.47)	-0.1 (-0.29 to 0.1)	16.20%	4.58 (3.14 to 8.93)	3.96 (2.32 to 8.18)	0 (0 to 0)	-13.54%
Timor-Leste	0.14 (0.1 to 0.21)	0.18 (0.12 to 0.25)	0.92 (0.77 to 1.08)	27.86%	3.94 (2.76 to 5.86)	4.7 (3.03 to 6.68)	0 (0 to 0)	19.19%
Togo	0.21 (0.12 to 0.28)	0.29 (0.13 to 0.43)	0 (0 to 0)	38.45%	5.35 (2.99 to 7.12)	6.12 (2.7 to 8.89)	0.3 (0.21 to 0.39)	14.40%
Tokelau	0.31 (0.21 to 0.54)	0.33 (0.21 to 0.58)	0 (0 to 0)	6.27%	7.7 (5.1 to 13.45)	7.66 (4.79 to 13.36)	0 (0 to 0)	-0.54%
Tonga	0.31 (0.2 to 0.57)	0.35 (0.21 to 0.6)	0.39 (0.29 to 0.49)	11.39%	7.34 (4.64 to 13.3)	8 (4.58 to 14.09)	0 (0 to 0)	8.92%
Trinidad and Tobago	0.38 (0.36 to 0.4)	0.51 (0.38 to 0.65)	0 (0 to 0)	33.18%	7.66 (7.3 to 8.01)	7.7 (5.84 to 9.75)	0 (0 to 0)	0.50%
Tunisia	0.45 (0.3 to 0.6)	1.11 (0.57 to 1.7)	0 (0 to 0)	147.17%	3.28 (2.19 to 4.37)	3.54 (1.88 to 5.3)	0 (0 to 0)	8.06%
Turkmenistan	1.2 (1.08 to 1.35)	0.94 (0.72 to 1.22)	0 (0 to 0)	-22.33%	24.66 (22.03 to 27.39)	15.16 (11.61 to 20.08)	-2.74 (-3.42 to -2.05)	-38.52%
Tuvalu	0.28 (0.17 to 0.47)	0.31 (0.21 to 0.47)	0 (0 to 0)	13.41%	7.23 (4.37 to 12.49)	7.79 (5.06 to 12.1)	0 (0 to 0)	7.79%
Türkiye	1.91 (0.96 to 2.88)	5.21 (2.55 to 7.07)	0 (0 to 0)	172.25%	19.74 (9.52 to 30.22)	16.47 (7.95 to 22.13)	0 (0 to 0)	-16.56%

Uganda	1.52 (1.07 to 2.24)	2.35 (1.37 to 3.5)	0 (0 to 0)	54.60%	39.86 (27.33 to 58.39)	54.92 (30.83 to 81.62)	0 (0 to 0)	37.80%
Ukraine	2.62 (2.29 to 3.14)	4.53 (3.26 to 6)	0 (0 to 0)	72.60%	41.08 (36.15 to 48.5)	57.72 (41.54 to 75.96)	0 (0 to 0)	40.50%
United Arab Emirates	2.51 (1.52 to 4.2)	4.52 (3.04 to 6.29)	0 (0 to 0)	79.99%	21.43 (13.25 to 34.82)	16.94 (11.42 to 23.06)	0 (0 to 0)	-20.96%
United Kingdom	7.38 (7.2 to 7.54)	15.45 (14.67 to 15.93)	0 (0 to 0)	109.36%	56.72 (55.45 to 58.13)	63.93 (61.07 to 66.97)	0 (0 to 0)	12.71%
United Republic of Tanzania	1.17 (0.74 to 1.52)	1.17 (0.64 to 1.72)	0 (0 to 0)	-0.52%	31.05 (19.23 to 40.78)	26.98 (14.42 to 41.05)	0 (0 to 0)	-13.11%
United States Virgin Islands	2.12 (1.6 to 3.14)	2.14 (1.4 to 3.13)	0 (0 to 0)	1.12%	38.75 (28.68 to 57.65)	30.5 (20.22 to 44.58)	0 (0 to 0)	-21.29%
United States of America	18.09 (17.53 to 18.46)	17.33 (16.44 to 17.92)	0 (0 to 0)	-4.20%	80.81 (78 to 83.84)	51.51 (48.57 to 54.71)	0 (0 to 0)	-36.26%
Uruguay	1.95 (1.8 to 2.1)	4.57 (4.12 to 4.98)	0 (0 to 0)	133.54%	30.68 (28.39 to 33.07)	45.05 (41.16 to 48.94)	0 (0 to 0)	46.81%
Uzbekistan	0.39 (0.28 to 0.53)	0.61 (0.48 to 0.73)	0 (0 to 0)	57.51%	7.48 (5.68 to 9.8)	9.44 (7.43 to 11.44)	0 (0 to 0)	26.24%
Vanuatu	0.35 (0.22 to 0.61)	0.37 (0.25 to 0.56)	0 (0 to 0)	5.65%	9.06 (5.49 to 16.26)	9.63 (6.19 to 14.78)	0 (0 to 0)	6.37%
Venezuela (Bolivarian Republic of)	0.57 (0.54 to 0.59)	1.19 (0.92 to 1.51)	0 (0 to 0)	110.13%	11.41 (10.93 to 11.94)	17.34 (13.37 to 22.13)	0 (0 to 0)	51.95%
Viet Nam	0.17 (0.12 to 0.23)	0.29 (0.17 to 0.38)	1.85 (1.77 to 1.93)	67.93%	4.47 (2.95 to 6.08)	5.81 (3.3 to 7.86)	0 (0 to 0)	30.00%
Yemen	0.45 (0.16 to 0.88)	0.72 (0.22 to 1.24)	0 (0 to 0)	57.40%	6.51 (2.19 to 12.83)	6.52 (2.01 to 11.66)	-0.08 (-0.14 to -0.02)	0.17%
Zambia	1.11 (0.69 to 1.47)	2.02 (0.71 to 3.62)	0 (0 to 0)	82.06%	31.17 (19.16 to	50.01 (16.54 to	0 (0 to 0)	60.42%

					41.5)	91.03)		
Zimbabwe	1.66 (1.11 to 2.3)	2.54 (1.37 to 3.49)	0 (0 to 0)	52.59%	37.3 (23.86 to 51.1)	61.46 (30.89 to 86.78)	0 (0 to 0)	64.76%

DALYs, disability-adjusted life-years; EAPC, estimated annual percentage change; UI, uncertainty interval; CI, confidence interval.

TABLE S11 Incidence and DALYs of non-melanoma skin cancer (basal-cell carcinoma) in 1990 and 2021 across 204 countries and territories, with age-standardized rates, 95% uncertainty intervals, and trends from 1990 to 2021.

Location name	Incidence				c			
	Age-standardized rate (95% UI), 1990	Age-standardized rate (95% UI), 2021	EAPC (95% CI), 1990-2021	Change in age-standardized rate, 1990-2021	Age-standardized rate (95% UI), 1990	Age-standardized rate (95% UI), 2021	EAPC (95% CI), 1990-2021	Change in age-standardized rate, 1990-2021
Afghanistan	4.7 (3.51 to 5.85)	4.55 (3.41 to 5.68)	-0.11 (-0.11 to -0.1)	-3.22%	0 (0 to 0)	0 (0 to 0)	0 (0 to 0)	-4.06%
Albania	25.39 (20.1 to 30.83)	25.66 (20.37 to 31.31)	0 (0 to 0)	1.07%	0.02 (0.01 to 0.03)	0.02 (0.01 to 0.03)	0 (0 to 0)	-0.33%
Algeria	5.8 (5.1 to 6.48)	5.4 (4.17 to 6.55)	-0.37 (-0.56 to -0.19)	-6.92%	0 (0 to 0.01)	0 (0 to 0.01)	0 (0 to 0)	-7.55%
American Samoa	0.13 (0.07 to 0.22)	0.13 (0.07 to 0.22)	0 (0 to 0)	-0.06%	0 (0 to 0)	0 (0 to 0)	0 (0 to 0)	-0.06%
Andorra	33.43 (26.76 to 40.67)	33.74 (26.97 to 41.97)	0 (-0.02 to 0.01)	0.93%	0.02 (0.01 to 0.04)	0.02 (0.01 to 0.04)	-0.06 (-0.08 to -0.04)	-1.60%
Angola	4.59 (3.42 to 5.78)	4.47 (3.33 to 5.53)	0 (0 to 0)	-2.55%	0 (0 to 0)	0 (0 to 0)	0 (0 to 0)	-3.50%
Antigua and Barbuda	3.58 (2.69 to 4.44)	3.7 (2.8 to 4.62)	0.13 (0.12 to 0.15)	3.58%	0 (0 to 0)	0 (0 to 0)	0 (0 to 0)	1.92%

Argentina	23.36 (20.24 to 26.33)	22.34 (17.52 to 27.33)	0 (0 to 0)	-4.34%	0.01 (0.01 to 0.03)	0.01 (0.01 to 0.03)	0 (0 to 0)	-4.42%
Armenia	22.63 (17.48 to 27.62)	22.87 (17.48 to 28.44)	0 (0 to 0)	1.06%	0.01 (0.01 to 0.03)	0.01 (0.01 to 0.03)	0 (0 to 0)	0.34%
Australia	29.2 (23.05 to 35.49)	26.81 (21.1 to 32.89)	0 (0 to 0)	-8.20%	0.02 (0.01 to 0.04)	0.02 (0.01 to 0.03)	0 (0 to 0)	-7.52%
Austria	24.54 (22.2 to 26.82)	27.87 (22.25 to 34.3)	0 (0 to 0)	13.58%	0.02 (0.01 to 0.03)	0.02 (0.01 to 0.04)	0 (0 to 0)	9.25%
Azerbaijan	22.99 (17.71 to 28.06)	23.25 (18.11 to 28.73)	0 (0 to 0)	1.10%	0.01 (0.01 to 0.02)	0.01 (0.01 to 0.02)	0 (0 to 0)	0.10%
Bahamas	3.57 (2.68 to 4.44)	3.66 (2.74 to 4.58)	0 (0 to 0)	2.52%	0 (0 to 0)	0 (0 to 0)	0 (0 to 0)	1.10%
Bahrain	3.76 (2.67 to 4.81)	3.83 (2.65 to 5.06)	0.08 (0.04 to 0.12)	1.89%	0 (0 to 0)	0 (0 to 0)	0 (0 to 0)	1.02%
Bangladesh	0.09 (0.05 to 0.15)	0.09 (0.05 to 0.15)	0 (0 to 0)	-0.89%	0 (0 to 0)	0 (0 to 0)	0 (0 to 0)	-1.66%
Barbados	3.51 (2.59 to 4.35)	3.64 (2.71 to 4.54)	0.15 (0.14 to 0.17)	3.76%	0 (0 to 0)	0 (0 to 0)	0 (0 to 0)	2.46%
Belarus	19.57 (18.83 to 20.25)	20.46 (15.83 to 25.17)	0 (0 to 0)	4.56%	0.01 (0.01 to 0.02)	0.01 (0.01 to 0.02)	0 (0 to 0)	2.57%
Belgium	29.17 (23.76 to 34.83)	30.89 (24.57 to 38.01)	0 (0 to 0)	5.91%	0.02 (0.01 to 0.04)	0.02 (0.01 to 0.04)	0 (0 to 0)	2.16%
Belize	3.76 (2.8 to 4.67)	3.79 (2.83 to 4.66)	0 (0 to 0)	0.87%	0 (0 to 0)	0 (0 to 0)	0 (0 to 0)	-0.27%
Benin	2.98 (2.21 to 3.68)	2.98 (2.22 to 3.69)	0 (0 to 0)	0.23%	0 (0 to 0)	0 (0 to 0)	0 (0 to 0)	-1.25%
Bermuda	2.38 (1.69 to 3.09)	2.27 (1.61 to 3)	0 (0 to 0)	-4.48%	0 (0 to 0)	0 (0 to 0)	-0.15 (-0.22 to -0.08)	-5.05%
Bhutan	0.09 (0.05 to 0.15)	0.09 (0.05 to 0.15)	0 (0 to 0)	0.31%	0 (0 to 0)	0 (0 to 0)	0 (0 to 0)	-0.35%
Bolivia	12.25 (9.77 to	12.43 (9.66 to	0 (0 to 0)	1.41%	0.01 (0 to 0.01)	0.01 (0 to 0.01)	0.07 (0.05 to	0.76%

(Plurinational State of)	14.85)	15.21)					0.09)	
Bosnia and Herzegovina	25.67 (20.22 to 31.53)	26.01 (20.53 to 32.02)	0 (0 to 0)	1.31%	0.02 (0.01 to 0.03)	0.02 (0.01 to 0.03)	0 (0 to 0)	0.59%
Botswana	14.59 (11.18 to 17.61)	14.52 (11.11 to 17.61)	0 (0 to 0)	-0.45%	0.01 (0 to 0.01)	0.01 (0 to 0.01)	0 (0 to 0)	-1.10%
Brazil	65.84 (57.62 to 73.65)	38.73 (31.72 to 45.71)	0 (0 to 0)	-41.18%	0.03 (0.01 to 0.06)	0.02 (0.01 to 0.04)	0 (0 to 0)	-36.01%
Brunei Darussalam	4.58 (3.58 to 5.7)	4.58 (3.61 to 5.74)	0 (0 to 0)	-0.06%	0 (0 to 0.01)	0 (0 to 0.01)	0 (0 to 0)	-1.46%
Bulgaria	34.67 (27.66 to 42.13)	46.49 (37.37 to 54.78)	0 (0 to 0)	34.10%	0.02 (0.01 to 0.03)	0.02 (0.01 to 0.04)	0 (0 to 0)	28.39%
Burkina Faso	2.97 (2.23 to 3.67)	2.99 (2.22 to 3.7)	0 (0 to 0)	0.48%	0 (0 to 0)	0 (0 to 0)	0 (0 to 0)	-0.51%
Burundi	3.64 (2.67 to 4.55)	3.74 (2.77 to 4.69)	0 (0 to 0)	2.92%	0 (0 to 0)	0 (0 to 0)	0 (0 to 0)	2.01%
Cabo Verde	2.83 (2.13 to 3.52)	2.88 (2.16 to 3.54)	0 (0 to 0)	1.63%	0 (0 to 0)	0 (0 to 0)	0 (0 to 0)	-0.46%
Cambodia	0.98 (0.71 to 1.27)	0.98 (0.73 to 1.27)	0 (0 to 0)	-0.11%	0 (0 to 0)	0 (0 to 0)	0 (0 to 0)	-0.75%
Cameroon	3 (2.24 to 3.74)	3 (2.23 to 3.72)	0 (0 to 0)	-0.08%	0 (0 to 0)	0 (0 to 0)	0 (0 to 0)	-1.24%
Canada	25.7 (19.76 to 31.38)	31.02 (23.87 to 37.99)	0.55 (0.39 to 0.72)	20.71%	0.02 (0.01 to 0.03)	0.02 (0.01 to 0.04)	0 (0 to 0)	16.69%
Central African Republic	4.53 (3.34 to 5.58)	4.52 (3.37 to 5.61)	0 (0 to 0)	-0.27%	0 (0 to 0)	0 (0 to 0)	-0.05 (-0.06 to -0.04)	-1.09%
Chad	2.99 (2.24 to 3.69)	3.08 (2.28 to 3.83)	0 (0 to 0)	3.06%	0 (0 to 0)	0 (0 to 0)	0 (0 to 0)	2.18%
Chile	19.05 (15.62 to 22.7)	19.79 (15.56 to 24.07)	0.17 (0.04 to 0.3)	3.88%	0.01 (0.01 to 0.02)	0.01 (0.01 to 0.02)	0.11 (0.01 to 0.2)	2.02%
China	3.76 (3.07 to 4.43)	31.58 (26.33 to 36.58)	0 (0 to 0)	740.31%	0 (0 to 0)	0.02 (0.01 to 0.03)	0 (0 to 0)	585.63%
Colombia	26.97 (21.23 to	26.85 (21.4 to 32.5)	0 (0 to 0)	-0.43%	0.02 (0.01 to 0.03)	0.02 (0.01 to 0.03)	0 (0 to 0)	-1.14%

	33.17)							
Comoros	3.62 (2.68 to 4.47)	3.61 (2.7 to 4.47)	0 (0 to 0)	-0.23%	0 (0 to 0)	0 (0 to 0)	0 (0 to 0)	-1.29%
Congo	4.45 (3.35 to 5.51)	4.56 (3.39 to 5.56)	0 (0 to 0)	2.38%	0 (0 to 0)	0 (0 to 0)	0 (0 to 0)	1.76%
Cook Islands	0.13 (0.07 to 0.22)	0.13 (0.07 to 0.22)	0 (0 to 0)	0.01%	0 (0 to 0)	0 (0 to 0)	0 (0 to 0)	1.10%
Costa Rica	41.75 (39.9 to 43.58)	31.23 (25.13 to 37.67)	0 (0 to 0)	-25.20%	0.02 (0.01 to 0.05)	0.02 (0.01 to 0.04)	0 (0 to 0)	-22.12%
Croatia	20.46 (16.3 to 25.47)	21.59 (16.74 to 26.87)	0 (0 to 0)	5.51%	0.01 (0.01 to 0.02)	0.01 (0.01 to 0.03)	0 (0 to 0)	2.39%
Cuba	9.79 (7.58 to 11.97)	8.12 (6.3 to 9.9)	0 (0 to 0)	-17.09%	0.01 (0 to 0.01)	0.01 (0 to 0.01)	-0.72 (-0.85 to -0.58)	-15.86%
Cyprus	23.24 (20.09 to 26.22)	24.37 (19.25 to 30.01)	0 (0 to 0)	4.86%	0.02 (0.01 to 0.03)	0.02 (0.01 to 0.03)	0 (0 to 0)	1.61%
Czechia	46.45 (44.6 to 48.15)	38 (30.08 to 46.25)	0 (0 to 0)	-18.20%	0.03 (0.01 to 0.05)	0.02 (0.01 to 0.04)	0 (0 to 0)	-16.46%
Côte d'Ivoire	2.02 (1.5 to 2.52)	2.01 (1.48 to 2.56)	0 (0 to 0)	-0.35%	0 (0 to 0)	0 (0 to 0)	0 (0 to 0)	-1.53%
Democratic People's Republic of Korea	0.22 (0.14 to 0.33)	0.22 (0.14 to 0.32)	0 (0 to 0)	1.14%	0 (0 to 0)	0 (0 to 0)	0 (0 to 0)	-0.16%
Democratic Republic of the Congo	4.51 (3.37 to 5.51)	4.46 (3.39 to 5.5)	0 (0 to 0)	-1.20%	0 (0 to 0)	0 (0 to 0)	0 (0 to 0)	-2.39%
Denmark	48.18 (45.73 to 50.43)	38.17 (30.42 to 46.6)	0 (0 to 0)	-20.78%	0.03 (0.01 to 0.05)	0.02 (0.01 to 0.04)	0 (0 to 0)	-20.78%
Djibouti	3.66 (2.68 to 4.57)	3.72 (2.74 to 4.61)	0 (0 to 0)	1.66%	0 (0 to 0)	0 (0 to 0)	0 (0 to 0)	0.22%
Dominica	3.45 (2.57 to 4.29)	3.71 (2.74 to 4.59)	0 (0 to 0)	7.48%	0 (0 to 0)	0 (0 to 0)	0.22 (0.2 to 0.25)	5.53%
Dominican Republic	3.74 (2.84 to 4.63)	3.75 (2.82 to 4.68)	0 (0 to 0)	0.21%	0 (0 to 0)	0 (0 to 0)	0 (0 to 0)	-1.42%
Ecuador	23.16 (21.22 to	14.31 (11.05 to	0 (0 to 0)	-38.21%	0.01 (0.01 to 0.03)	0.01 (0 to 0.02)	-1.63 (-1.89 to	-34.19%

	25.36)	17.7)					-1.38)	
Egypt	4.79 (4 to 5.57)	4.57 (3.48 to 5.68)	0 (0 to 0)	-4.69%	0 (0 to 0.01)	0 (0 to 0)	0 (0 to 0)	-4.35%
El Salvador	27.28 (21.48 to 32.68)	27.3 (21.13 to 33.09)	0 (0 to 0)	0.08%	0.01 (0.01 to 0.03)	0.01 (0.01 to 0.03)	0 (0 to 0)	-0.08%
Equatorial Guinea	4.45 (3.34 to 5.4)	4.45 (3.33 to 5.43)	0 (0 to 0)	0.18%	0 (0 to 0)	0 (0 to 0)	0 (0 to 0)	-0.66%
Eritrea	3.52 (2.65 to 4.38)	3.55 (2.66 to 4.45)	0 (0 to 0)	0.88%	0 (0 to 0)	0 (0 to 0)	0 (0 to 0)	-0.47%
Estonia	22.01 (20.83 to 23.17)	20.19 (15.74 to 24.83)	0 (0 to 0)	-8.28%	0.01 (0.01 to 0.03)	0.01 (0.01 to 0.02)	0 (0 to 0)	-7.57%
Eswatini	14.61 (11.24 to 17.85)	14.32 (11.06 to 17.59)	0 (0 to 0)	-1.99%	0.01 (0 to 0.01)	0.01 (0 to 0.01)	0 (0 to 0)	-2.91%
Ethiopia	3.89 (2.88 to 4.78)	3.88 (2.88 to 4.76)	0 (0 to 0)	-0.25%	0 (0 to 0)	0 (0 to 0)	0 (0 to 0)	-0.85%
Fiji	0.13 (0.07 to 0.22)	0.13 (0.07 to 0.22)	0 (0 to 0)	-0.50%	0 (0 to 0)	0 (0 to 0)	0 (0 to 0)	-0.68%
Finland	33.04 (26.24 to 40.21)	33.72 (26.52 to 41.35)	0 (0 to 0)	2.07%	0.02 (0.01 to 0.04)	0.02 (0.01 to 0.04)	0 (0 to 0)	0.76%
France	50.46 (45.42 to 55.04)	39.06 (30.69 to 47.68)	0 (0 to 0)	-22.59%	0.03 (0.01 to 0.06)	0.02 (0.01 to 0.05)	0 (0 to 0)	-20.55%
Gabon	4.47 (3.35 to 5.49)	4.52 (3.42 to 5.65)	0 (0 to 0)	1.20%	0 (0 to 0)	0 (0 to 0)	0 (0 to 0)	-0.12%
Gambia	2.84 (2.19 to 3.48)	2.84 (2.12 to 3.52)	0 (0 to 0)	-0.10%	0 (0 to 0)	0 (0 to 0)	-0.01 (-0.03 to 0.01)	-0.80%
Georgia	22.48 (17.19 to 27.63)	22.6 (17.7 to 27.59)	0 (0 to 0)	0.54%	0.01 (0.01 to 0.03)	0.01 (0.01 to 0.03)	0 (0 to 0)	-1.58%
Germany	19.13 (16.88 to 21.19)	27.89 (22.2 to 34.21)	0 (0 to 0)	45.77%	0.01 (0.01 to 0.02)	0.02 (0.01 to 0.03)	0 (0 to 0)	33.84%
Ghana	2.99 (2.22 to 3.71)	2.94 (2.2 to 3.63)	0 (0 to 0)	-1.77%	0 (0 to 0)	0 (0 to 0)	0 (0 to 0)	-3.04%
Greece	33.24 (26.06 to 40.92)	33.53 (26.63 to 41.18)	0 (0 to 0)	0.86%	0.02 (0.01 to 0.04)	0.02 (0.01 to 0.04)	0 (0 to 0)	-1.65%

Greenland	64.09 (50.77 to 77.45)	67.44 (53.14 to 80.59)	0 (0 to 0)	5.23%	0.03 (0.01 to 0.05)	0.03 (0.01 to 0.06)	0 (0 to 0)	4.55%
Grenada	3.6 (2.65 to 4.52)	3.7 (2.75 to 4.66)	0 (0 to 0)	2.77%	0 (0 to 0)	0 (0 to 0)	0 (0 to 0)	1.76%
Guam	0.13 (0.07 to 0.22)	0.13 (0.07 to 0.22)	0 (0 to 0)	-0.01%	0 (0 to 0)	0 (0 to 0)	0 (0 to 0)	0.58%
Guatemala	28.06 (22.19 to 34)	28.08 (22.22 to 34.16)	0 (0 to 0)	0.07%	0.01 (0.01 to 0.03)	0.01 (0.01 to 0.03)	0 (0 to 0)	0.18%
Guinea	2.79 (2.13 to 3.45)	2.71 (2.03 to 3.36)	0 (0 to 0)	-2.87%	0 (0 to 0)	0 (0 to 0)	0 (0 to 0)	-3.62%
Guinea-Bissau	3.02 (2.2 to 3.73)	2.99 (2.21 to 3.73)	0 (0 to 0)	-1.22%	0 (0 to 0)	0 (0 to 0)	0 (0 to 0)	-2.29%
Guyana	3.81 (2.86 to 4.78)	3.78 (2.8 to 4.74)	0 (-0.02 to 0.02)	-0.84%	0 (0 to 0)	0 (0 to 0)	0 (0 to 0)	-2.31%
Haiti	3.8 (2.84 to 4.74)	3.84 (2.85 to 4.85)	0 (0 to 0)	1.10%	0 (0 to 0)	0 (0 to 0)	0 (0 to 0)	0.34%
Honduras	28.09 (22.17 to 33.5)	28.14 (22.09 to 33.94)	0 (0 to 0)	0.16%	0.01 (0.01 to 0.03)	0.01 (0.01 to 0.03)	0 (0 to 0)	-0.94%
Hungary	28.5 (26.12 to 31.03)	26.86 (21.1 to 33.02)	0 (0 to 0)	-5.77%	0.02 (0.01 to 0.03)	0.01 (0.01 to 0.03)	0 (0 to 0)	-5.42%
Iceland	33.37 (26.45 to 40.47)	33.57 (26.55 to 40.54)	0 (0 to 0)	0.62%	0.02 (0.01 to 0.04)	0.02 (0.01 to 0.04)	0 (0 to 0)	1.56%
India	1.49 (1.1 to 1.89)	1.61 (1.21 to 2.01)	0.29 (0.24 to 0.33)	8.07%	0 (0 to 0)	0 (0 to 0)	0 (0 to 0)	8.50%
Indonesia	1.08 (0.81 to 1.38)	1.09 (0.81 to 1.4)	0 (0 to 0)	1.07%	0 (0 to 0)	0 (0 to 0)	0 (0 to 0)	-0.31%
Iran (Islamic Republic of)	9.94 (7.59 to 12.2)	7.29 (5.6 to 8.89)	-0.71 (-0.93 to -0.49)	-26.63%	0.01 (0 to 0.01)	0 (0 to 0.01)	0 (0 to 0)	-24.16%
Iraq	4.03 (3.06 to 5.04)	4.05 (3.67 to 4.46)	-0.2 (-0.36 to -0.03)	0.60%	0 (0 to 0)	0 (0 to 0)	0 (0 to 0)	0.79%
Ireland	76.82 (69.31 to 84.55)	47.67 (38.06 to 57.43)	0 (0 to 0)	-37.95%	0.04 (0.02 to 0.08)	0.03 (0.01 to 0.05)	0 (0 to 0)	-32.94%

Israel	33.43 (25.9 to 41.18)	33.99 (27.12 to 41.21)	0 (0 to 0)	1.68%	0.02 (0.01 to 0.04)	0.02 (0.01 to 0.04)	0 (0 to 0)	-2.42%
Italy	37.52 (32.55 to 42.63)	40.15 (31.89 to 49.11)	0 (0 to 0)	7.02%	0.02 (0.01 to 0.04)	0.02 (0.01 to 0.05)	0 (0 to 0)	3.50%
Jamaica	5.44 (4.46 to 6.44)	4.45 (3.35 to 5.5)	0 (0 to 0)	-18.27%	0 (0 to 0.01)	0 (0 to 0.01)	0 (0 to 0)	-18.28%
Japan	2.56 (2.02 to 3.14)	3.55 (2.77 to 4.45)	0 (0 to 0)	38.73%	0 (0 to 0)	0 (0 to 0.01)	0 (0 to 0)	32.28%
Jordan	6.6 (5.3 to 7.9)	5.81 (4.45 to 7.2)	-0.08 (-0.27 to 0.1)	-12.04%	0 (0 to 0.01)	0 (0 to 0.01)	0 (0 to 0)	-11.61%
Kazakhstan	22.78 (17.8 to 27.7)	23.03 (18 to 28.26)	0 (0 to 0)	1.06%	0.01 (0.01 to 0.02)	0.01 (0.01 to 0.02)	0 (0 to 0)	0.44%
Kenya	3.63 (2.7 to 4.52)	3.41 (2.53 to 4.22)	0 (0 to 0)	-6.15%	0 (0 to 0)	0 (0 to 0)	0 (0 to 0)	-6.28%
Kiribati	0.13 (0.07 to 0.22)	0.13 (0.07 to 0.22)	0 (0 to 0)	0.04%	0 (0 to 0)	0 (0 to 0)	0 (0 to 0)	0.10%
Kuwait	4.12 (3.42 to 4.8)	4.08 (3.06 to 5.05)	0.14 (-0.14 to 0.42)	-0.99%	0 (0 to 0.01)	0 (0 to 0.01)	0.11 (-0.17 to 0.39)	-1.07%
Kyrgyzstan	24.33 (21.93 to 26.64)	22.7 (17.73 to 27.96)	0 (0 to 0)	-6.69%	0.01 (0.01 to 0.03)	0.01 (0.01 to 0.02)	0 (0 to 0)	-6.32%
Lao People's Democratic Republic	1 (0.71 to 1.29)	1.01 (0.74 to 1.31)	0 (0 to 0)	0.49%	0 (0 to 0)	0 (0 to 0)	0 (0 to 0)	-0.67%
Latvia	16.68 (15.83 to 17.44)	18.26 (14.25 to 22.78)	0 (0 to 0)	9.49%	0.01 (0 to 0.02)	0.01 (0 to 0.02)	0 (0 to 0)	7.73%
Lebanon	8.11 (6.32 to 9.89)	8.11 (6.27 to 9.87)	-0.01 (-0.77 to 0.76)	-0.09%	0.01 (0 to 0.01)	0.01 (0 to 0.01)	0 (0 to 0)	-0.11%
Lesotho	14.14 (10.85 to 17.22)	14.27 (10.86 to 17.44)	0 (0 to 0)	0.90%	0.01 (0 to 0.01)	0.01 (0 to 0.01)	0.02 (0 to 0.03)	0.07%
Liberia	3.07 (2.32 to 3.81)	3.05 (2.3 to 3.77)	0 (0 to 0)	-0.90%	0 (0 to 0)	0 (0 to 0)	0 (0 to 0)	-1.58%
Libya	3.38 (2.48 to 4.32)	3.32 (2.44 to 4.16)	-0.04 (-0.05 to -0.02)	-1.67%	0 (0 to 0)	0 (0 to 0)	0 (0 to 0)	-2.64%

Lithuania	24.25 (22.6 to 25.82)	23.28 (19.13 to 27.55)	0 (0 to 0)	-4.01%	0.01 (0.01 to 0.03)	0.01 (0.01 to 0.03)	0 (0 to 0)	-4.49%
Luxembourg	32.77 (25.79 to 40.12)	33.72 (26.66 to 41.29)	0 (0 to 0)	2.92%	0.02 (0.01 to 0.04)	0.02 (0.01 to 0.04)	0 (0 to 0)	0.20%
Madagascar	3.69 (2.76 to 4.59)	3.65 (2.72 to 4.5)	0 (0 to 0)	-1.22%	0 (0 to 0)	0 (0 to 0)	-0.07 (-0.08 to -0.06)	-2.45%
Malawi	2.93 (2.17 to 3.62)	2.87 (2.09 to 3.65)	0 (0 to 0)	-2.09%	0 (0 to 0)	0 (0 to 0)	0 (0 to 0)	-2.84%
Malaysia	3.11 (2.55 to 3.69)	2.31 (1.75 to 2.87)	0 (0 to 0)	-25.66%	0 (0 to 0)	0 (0 to 0)	0 (0 to 0)	-23.78%
Maldives	1.03 (0.76 to 1.34)	1.02 (0.74 to 1.31)	0 (0 to 0)	-0.94%	0 (0 to 0)	0 (0 to 0)	-0.04 (-0.06 to -0.02)	-0.98%
Mali	4.28 (3.32 to 5.16)	4.23 (3.22 to 5.15)	0 (0 to 0)	-1.27%	0 (0 to 0)	0 (0 to 0)	0 (0 to 0)	-1.81%
Malta	43.5 (40.08 to 47.43)	36.7 (28.96 to 44.95)	0 (0 to 0)	-15.64%	0.03 (0.01 to 0.05)	0.02 (0.01 to 0.05)	0 (0 to 0)	-13.94%
Marshall Islands	0.13 (0.07 to 0.22)	0.13 (0.07 to 0.22)	0 (0 to 0)	-0.08%	0 (0 to 0)	0 (0 to 0)	0 (0 to 0)	-0.32%
Mauritania	2.95 (2.2 to 3.65)	3 (2.27 to 3.74)	0 (0 to 0)	1.77%	0 (0 to 0)	0 (0 to 0)	0 (0 to 0)	0.58%
Mauritius	0.98 (0.71 to 1.26)	0.99 (0.74 to 1.29)	0 (0 to 0)	1.33%	0 (0 to 0)	0 (0 to 0)	0 (0 to 0)	0.24%
Mexico	28.44 (22.62 to 34.45)	28.45 (22.67 to 34.49)	0 (0 to 0)	0.04%	0.01 (0.01 to 0.03)	0.01 (0.01 to 0.03)	0 (0 to 0)	0.09%
Micronesia (Federated States of)	0.13 (0.07 to 0.22)	0.13 (0.07 to 0.22)	0 (0 to 0)	-0.01%	0 (0 to 0)	0 (0 to 0)	0 (0 to 0)	-0.55%
Monaco	33.6 (26.49 to 40.8)	34.03 (27.05 to 41.63)	0 (0 to 0)	1.29%	0.02 (0.01 to 0.04)	0.02 (0.01 to 0.04)	0 (0 to 0)	0.48%
Mongolia	23.43 (18.42 to 28.76)	23.47 (18.2 to 28.75)	0 (0 to 0)	0.19%	0.01 (0.01 to 0.02)	0.01 (0.01 to 0.02)	0 (-0.01 to 0.01)	-0.31%
Montenegro	25.87 (20.35 to 31.87)	25.89 (20.28 to 31.64)	0 (0 to 0)	0.09%	0.02 (0.01 to 0.03)	0.02 (0.01 to 0.03)	0 (-0.01 to 0.02)	-0.47%

Morocco	2.91 (2.18 to 3.74)	2.9 (2.15 to 3.65)	0.03 (-0.02 to 0.09)	-0.31%	0 (0 to 0)	0 (0 to 0)	0 (0 to 0)	-1.27%
Mozambique	3.66 (2.7 to 4.56)	3.59 (2.65 to 4.45)	0 (0 to 0)	-2.01%	0 (0 to 0)	0 (0 to 0)	0 (0 to 0)	-3.05%
Myanmar	1 (0.72 to 1.29)	0.99 (0.72 to 1.29)	0 (0 to 0)	-0.85%	0 (0 to 0)	0 (0 to 0)	-0.05 (-0.06 to -0.05)	-1.63%
Namibia	15.31 (11.73 to 18.57)	15.21 (11.7 to 18.53)	0 (0 to 0)	-0.66%	0.01 (0 to 0.02)	0.01 (0 to 0.01)	0 (0 to 0)	-1.40%
Nauru	0.13 (0.07 to 0.22)	0.13 (0.07 to 0.22)	0 (0 to 0)	0.03%	0 (0 to 0)	0 (0 to 0)	0 (0 to 0)	0.25%
Nepal	0.09 (0.05 to 0.15)	0.09 (0.05 to 0.15)	0 (0 to 0)	0.19%	0 (0 to 0)	0 (0 to 0)	0 (0 to 0)	-0.47%
Netherlands	18.16 (14.51 to 21.65)	23.46 (18.55 to 28.71)	0 (0 to 0)	29.18%	0.01 (0.01 to 0.02)	0.02 (0.01 to 0.03)	0 (0 to 0)	22.15%
New Zealand	28.38 (22.1 to 34.79)	28.34 (22.43 to 34.6)	0 (0 to 0)	-0.14%	0.02 (0.01 to 0.03)	0.02 (0.01 to 0.03)	0 (0 to 0)	-1.37%
Nicaragua	26.96 (21.13 to 32.84)	26.98 (21.49 to 33.1)	0 (0 to 0)	0.07%	0.02 (0.01 to 0.03)	0.02 (0.01 to 0.03)	0 (0 to 0)	0.20%
Niger	3.03 (2.26 to 3.72)	3 (2.22 to 3.7)	0 (0 to 0)	-1.01%	0 (0 to 0)	0 (0 to 0)	0 (0 to 0)	-2.13%
Nigeria	2.61 (1.93 to 3.25)	2.56 (1.92 to 3.18)	0 (0 to 0)	-1.73%	0 (0 to 0)	0 (0 to 0)	0 (0 to 0)	-2.91%
Niue	0.13 (0.07 to 0.22)	0.13 (0.07 to 0.22)	0 (0 to 0)	0.05%	0 (0 to 0)	0 (0 to 0)	0 (0 to 0)	0.13%
North Macedonia	26.53 (21.03 to 32.25)	26.56 (21.18 to 32.82)	0 (0 to 0)	0.09%	0.01 (0.01 to 0.03)	0.01 (0.01 to 0.03)	0 (0 to 0)	-0.20%
Northern Mariana Islands	0.13 (0.07 to 0.22)	0.13 (0.07 to 0.22)	0 (0 to 0)	-0.24%	0 (0 to 0)	0 (0 to 0)	0 (0 to 0)	-0.15%
Norway	34.95 (27.78 to 42.37)	35.35 (28.15 to 42.81)	0 (0 to 0)	1.15%	0.02 (0.01 to 0.04)	0.02 (0.01 to 0.04)	0 (0 to 0)	1.25%
Oman	5.34 (4.34 to 6.25)	5.2 (4.41 to 5.92)	0.05 (-0.03 to 0.14)	-2.66%	0 (0 to 0.01)	0 (0 to 0.01)	0 (0 to 0)	-3.88%

Pakistan	0.61 (0.46 to 0.76)	0.62 (0.47 to 0.78)	-0.06 (-0.1 to -0.01)	0.26%	0 (0 to 0)	0 (0 to 0)	0 (0 to 0)	-0.34%
Palau	0.13 (0.07 to 0.22)	0.13 (0.07 to 0.22)	0 (0 to 0)	-0.22%	0 (0 to 0)	0 (0 to 0)	0 (0 to 0)	-1.35%
Palestine	4.4 (3.32 to 5.41)	4.47 (3.36 to 5.53)	0.04 (0.02 to 0.05)	1.40%	0 (0 to 0.01)	0 (0 to 0.01)	0 (0 to 0)	0.70%
Panama	18.17 (17.5 to 18.8)	18.01 (16.72 to 19.42)	0 (0 to 0)	-0.86%	0.01 (0.01 to 0.02)	0.01 (0.01 to 0.02)	0 (0 to 0)	-3.17%
Papua New Guinea	0.13 (0.07 to 0.22)	0.13 (0.07 to 0.22)	0 (0 to 0)	-0.02%	0 (0 to 0)	0 (0 to 0)	0.03 (0.02 to 0.05)	-0.31%
Paraguay	0.11 (0.06 to 0.18)	0.1 (0.06 to 0.16)	0 (0 to 0)	-12.82%	0 (0 to 0)	0 (0 to 0)	0 (0 to 0)	-13.52%
Peru	12.93 (11.65 to 14.32)	9.85 (7.71 to 11.95)	0 (0 to 0)	-23.85%	0.01 (0 to 0.02)	0.01 (0 to 0.01)	0 (0 to 0)	-21.62%
Philippines	2.58 (1.99 to 3.2)	1.84 (1.37 to 2.32)	0 (0 to 0)	-28.81%	0 (0 to 0)	0 (0 to 0)	-0.87 (-1.31 to -0.43)	-27.96%
Poland	13.91 (11.1 to 17.09)	19.68 (15.43 to 24.46)	0 (0 to 0)	41.51%	0.01 (0 to 0.02)	0.01 (0.01 to 0.02)	0 (0 to 0)	32.45%
Portugal	13.82 (11.78 to 16.31)	20.08 (15.79 to 24.33)	0 (0 to 0)	45.29%	0.01 (0 to 0.02)	0.01 (0.01 to 0.03)	0 (0 to 0)	35.26%
Puerto Rico	3.64 (2.73 to 4.54)	3.6 (2.68 to 4.49)	0 (0 to 0)	-1.09%	0 (0 to 0.01)	0 (0 to 0)	0 (0 to 0)	-1.42%
Qatar	3.85 (2.72 to 5.06)	3.87 (2.63 to 5.06)	-0.03 (-0.1 to 0.04)	0.48%	0 (0 to 0)	0 (0 to 0)	0 (0 to 0)	-0.19%
Republic of Korea	2.66 (2.46 to 2.87)	4.44 (4.1 to 4.78)	0 (0 to 0)	66.93%	0 (0 to 0)	0 (0 to 0.01)	0 (0 to 0)	57.69%
Republic of Moldova	19.67 (15.39 to 24.02)	19.75 (15.39 to 24.69)	0 (0 to 0)	0.38%	0.01 (0 to 0.02)	0.01 (0 to 0.02)	0 (0 to 0)	-0.29%
Romania	13.6 (11.7 to 15.79)	19.79 (15.64 to 24.37)	0 (0 to 0)	45.55%	0.01 (0 to 0.02)	0.01 (0 to 0.02)	0 (0 to 0)	36.14%

Russian Federation	13.02 (10.14 to 15.99)	17.47 (13.62 to 21.48)	0 (0 to 0)	34.14%	0.01 (0 to 0.01)	0.01 (0 to 0.02)	0.82 (0.72 to 0.92)	29.87%
Rwanda	3.61 (2.64 to 4.48)	3.56 (2.67 to 4.36)	0 (0 to 0)	-1.37%	0 (0 to 0)	0 (0 to 0)	0 (0 to 0)	-2.15%
Saint Kitts and Nevis	3.65 (2.67 to 4.58)	3.73 (2.75 to 4.65)	0 (0 to 0)	2.24%	0 (0 to 0)	0 (0 to 0)	0 (0 to 0)	2.25%
Saint Lucia	3.57 (2.65 to 4.42)	3.73 (2.79 to 4.63)	0 (0 to 0)	4.61%	0 (0 to 0)	0 (0 to 0)	0 (0 to 0)	2.93%
Saint Vincent and the Grenadines	3.6 (2.71 to 4.49)	3.87 (2.88 to 4.84)	0 (0 to 0)	7.56%	0 (0 to 0)	0 (0 to 0)	0 (0 to 0)	6.10%
Samoa	0.14 (0.08 to 0.23)	0.13 (0.07 to 0.22)	0 (0 to 0)	-9.18%	0 (0 to 0)	0 (0 to 0)	0 (0 to 0)	-9.45%
San Marino	33.27 (26.43 to 40.61)	33.32 (26.57 to 40.8)	0 (0 to 0)	0.17%	0.02 (0.01 to 0.04)	0.02 (0.01 to 0.04)	0 (0 to 0)	1.06%
Sao Tome and Principe	2.94 (2.22 to 3.67)	2.97 (2.22 to 3.69)	0 (0 to 0)	1.06%	0 (0 to 0)	0 (0 to 0)	0 (0 to 0)	0.12%
Saudi Arabia	3.96 (2.97 to 4.95)	3.92 (3.56 to 4.24)	-0.1 (-0.38 to 0.18)	-1.16%	0 (0 to 0)	0 (0 to 0.01)	0 (0 to 0)	-1.16%
Senegal	5.17 (3.97 to 6.28)	5.14 (3.92 to 6.21)	0 (0 to 0)	-0.64%	0 (0 to 0.01)	0 (0 to 0.01)	0 (0 to 0)	-1.39%
Serbia	20.19 (19.3 to 21.02)	24.11 (19.06 to 29.51)	0 (0 to 0)	19.44%	0.01 (0.01 to 0.02)	0.01 (0.01 to 0.03)	0 (0 to 0)	12.92%
Seychelles	0.92 (0.54 to 1.3)	0.99 (0.58 to 1.41)	0 (0 to 0)	7.60%	0 (0 to 0)	0 (0 to 0)	0 (0 to 0)	5.17%
Sierra Leone	3.04 (2.28 to 3.76)	3.04 (2.24 to 3.75)	0 (0 to 0)	-0.15%	0 (0 to 0)	0 (0 to 0)	0 (0 to 0)	-1.06%
Singapore	7.93 (7.48 to 8.56)	5.65 (4.47 to 6.93)	0 (0 to 0)	-28.76%	0.01 (0 to 0.01)	0 (0 to 0.01)	0 (0 to 0)	-26.68%
Slovakia	33.08 (31.88 to 34.18)	28.35 (22.25 to 34.75)	0 (0 to 0)	-14.31%	0.02 (0.01 to 0.04)	0.02 (0.01 to 0.03)	-0.49 (-0.79 to -0.2)	-13.23%
Slovenia	24.02 (22.51 to 25.62)	37.29 (28.98 to 46.79)	0 (0 to 0)	55.26%	0.02 (0.01 to 0.03)	0.02 (0.01 to 0.04)	0 (0 to 0)	41.92%
Solomon Islands	0.13 (0.07 to 0.22)	0.13 (0.07 to 0.22)	0 (0 to 0)	-0.16%	0 (0 to 0)	0 (0 to 0)	-0.01 (-0.01 to 0)	-0.39%

Somalia	3.67 (2.77 to 4.55)	3.56 (2.68 to 4.43)	0 (0 to 0)	-2.90%	0 (0 to 0)	0 (0 to 0)	0 (0 to 0)	-3.65%
South Africa	16.82 (12.69 to 20.54)	20.96 (15.88 to 25.5)	0 (0 to 0)	24.60%	0.01 (0 to 0.02)	0.01 (0 to 0.02)	0 (0 to 0)	22.10%
South Sudan	3.81 (2.82 to 4.74)	3.76 (2.78 to 4.72)	0 (0 to 0)	-1.54%	0 (0 to 0)	0 (0 to 0)	0 (0 to 0)	-2.35%
Spain	47.83 (45.58 to 49.85)	37.13 (29.14 to 45.74)	0 (0 to 0)	-22.37%	0.03 (0.01 to 0.06)	0.02 (0.01 to 0.05)	0 (0 to 0)	-21.74%
Sri Lanka	1.07 (0.88 to 1.26)	0.81 (0.6 to 1.06)	0 (0 to 0)	-23.61%	0 (0 to 0)	0 (0 to 0)	-0.78 (-1.12 to -0.43)	-24.38%
Sudan	4.62 (3.49 to 5.68)	4.7 (3.56 to 5.8)	0 (0 to 0)	1.62%	0 (0 to 0.01)	0 (0 to 0.01)	0 (0 to 0)	0.98%
Suriname	3.72 (2.81 to 4.59)	3.67 (2.75 to 4.58)	0 (0 to 0)	-1.43%	0 (0 to 0)	0 (0 to 0)	0 (0 to 0)	-2.53%
Sweden	35.16 (27.41 to 43.44)	43.46 (35.41 to 52.36)	0 (0 to 0)	23.58%	0.02 (0.01 to 0.04)	0.02 (0.01 to 0.05)	0 (0 to 0)	12.73%
Switzerland	72.39 (69.88 to 74.72)	45.09 (36 to 54.95)	0 (0 to 0)	-37.71%	0.04 (0.02 to 0.08)	0.03 (0.01 to 0.05)	0 (0 to 0)	-34.21%
Syrian Arab Republic	4.55 (3.45 to 5.59)	4.55 (3.48 to 5.57)	0.01 (-0.01 to 0.04)	0.00%	0 (0 to 0.01)	0 (0 to 0.01)	0 (0 to 0)	-0.09%
Taiwan (Province of China)	2.3 (2.03 to 2.51)	0.11 (0.06 to 0.18)	0 (0 to 0)	-95.15%	0 (0 to 0)	0 (0 to 0)	0 (0 to 0)	-94.49%
Tajikistan	23.08 (17.84 to 28.77)	23.36 (18.05 to 28.87)	0 (0 to 0)	1.20%	0.01 (0.01 to 0.02)	0.01 (0.01 to 0.02)	0 (0 to 0)	0.66%
Thailand	5.71 (5.3 to 6.2)	2.57 (1.95 to 3.18)	0 (0 to 0)	-54.92%	0 (0 to 0.01)	0 (0 to 0)	0 (0 to 0)	-51.81%
Timor-Leste	1.01 (0.74 to 1.3)	1.01 (0.75 to 1.31)	0 (0 to 0)	0.49%	0 (0 to 0)	0 (0 to 0)	0 (0 to 0)	-0.29%
Togo	2.98 (2.23 to 3.68)	2.92 (2.17 to 3.62)	0 (0 to 0)	-1.77%	0 (0 to 0)	0 (0 to 0)	-0.11 (-0.12 to -0.09)	-2.57%
Tokelau	0.13 (0.07 to 0.22)	0.13 (0.07 to 0.22)	0 (0 to 0)	0.07%	0 (0 to 0)	0 (0 to 0)	0 (0 to 0)	0.20%
Tonga	0.13 (0.08 to 0.23)	0.13 (0.07 to 0.22)	0 (0 to 0)	-0.79%	0 (0 to 0)	0 (0 to 0)	0 (0 to 0)	-1.41%

Trinidad and Tobago	3.16 (2.66 to 3.64)	3.54 (2.59 to 4.44)	0 (0 to 0)	11.93%	0 (0 to 0)	0 (0 to 0)	0 (0 to 0)	9.25%
Tunisia	6.11 (4.87 to 7.65)	5.47 (4.16 to 6.79)	-0.46 (-0.66 to -0.25)	-10.52%	0 (0 to 0.01)	0 (0 to 0.01)	0 (0 to 0)	-10.58%
Turkmenistan	22.84 (17.78 to 27.76)	23.01 (17.93 to 27.63)	0 (0 to 0)	0.71%	0.01 (0.01 to 0.02)	0.01 (0.01 to 0.03)	0 (-0.01 to 0.02)	0.05%
Tuvalu	0.13 (0.07 to 0.22)	0.13 (0.07 to 0.22)	0 (0 to 0)	-0.11%	0 (0 to 0)	0 (0 to 0)	0 (0 to 0)	-0.37%
Türkiye	12.3 (10.57 to 13.83)	9.37 (7.21 to 11.43)	-0.82 (-1.56 to -0.07)	-23.86%	0.01 (0 to 0.02)	0.01 (0 to 0.01)	0 (0 to 0)	-20.20%
Uganda	5.59 (4.77 to 6.44)	4.94 (4 to 5.84)	0 (0 to 0)	-11.63%	0 (0 to 0.01)	0 (0 to 0.01)	0 (0 to 0)	-11.45%
Ukraine	19.99 (15.53 to 24.75)	20.36 (16.01 to 24.95)	0 (0 to 0)	1.87%	0.01 (0 to 0.02)	0.01 (0 to 0.02)	0 (0 to 0)	0.49%
United Arab Emirates	4.81 (3.62 to 5.94)	5.3 (4 to 6.54)	0.33 (0.3 to 0.36)	10.08%	0 (0 to 0.01)	0 (0 to 0.01)	0 (0 to 0)	10.25%
United Kingdom	41.67 (33.82 to 49.64)	40.45 (32.24 to 49.01)	0 (0 to 0)	-2.93%	0.02 (0.01 to 0.04)	0.02 (0.01 to 0.04)	0 (0 to 0)	-2.24%
United Republic of Tanzania	3.65 (2.7 to 4.49)	3.64 (2.68 to 4.54)	0 (0 to 0)	-0.31%	0 (0 to 0)	0 (0 to 0)	0 (0 to 0)	-1.14%
United States Virgin Islands	3.67 (2.71 to 4.63)	3.73 (2.76 to 4.64)	0 (0 to 0)	1.44%	0 (0 to 0)	0 (0 to 0)	0 (0 to 0)	1.02%
United States of America	227.67 (181.57 to 271.04)	527.05 (478.06 to 575.22)	0 (0 to 0)	131.50%	0.1 (0.04 to 0.18)	0.21 (0.1 to 0.4)	0 (0 to 0)	117.99%
Uruguay	24.07 (20.7 to 27.53)	24.96 (19.66 to 30.41)	0.19 (-0.11 to 0.5)	3.70%	0.01 (0.01 to 0.03)	0.01 (0.01 to 0.03)	0 (0 to 0)	2.38%
Uzbekistan	23.45 (18.2 to 28.62)	23.59 (18.25 to 28.76)	0 (0 to 0)	0.62%	0.01 (0.01 to 0.02)	0.01 (0.01 to 0.02)	0 (0 to 0)	0.50%
Vanuatu	0.13 (0.07 to 0.22)	0.13 (0.07 to 0.22)	0 (0 to 0)	-0.28%	0 (0 to 0)	0 (0 to 0)	0 (-0.01 to 0)	-0.33%

Venezuela (Bolivarian Republic of)	27.34 (21.44 to 33.28)	27.21 (21.79 to 32.98)	0 (0 to 0)	-0.48%	0.01 (0.01 to 0.03)	0.01 (0.01 to 0.03)	-0.02 (-0.04 to -0.01)	-0.55%
Viet Nam	2.38 (2.01 to 2.74)	1.36 (1.01 to 1.76)	0 (0 to 0)	-42.79%	0 (0 to 0)	0 (0 to 0)	0 (0 to 0)	-42.20%
Yemen	4.44 (3.31 to 5.56)	4.55 (3.41 to 5.56)	0 (0 to 0)	2.41%	0 (0 to 0)	0 (0 to 0)	0.07 (0.05 to 0.09)	2.02%
Zambia	3.77 (2.79 to 4.67)	3.68 (2.7 to 4.63)	0 (0 to 0)	-2.40%	0 (0 to 0)	0 (0 to 0)	0 (0 to 0)	-3.26%
Zimbabwe	12.33 (9.61 to 14.93)	11.16 (8.47 to 13.49)	0 (0 to 0)	-9.49%	0.01 (0 to 0.01)	0.01 (0 to 0.01)	0 (0 to 0)	-9.53%

DALYs, disability-adjusted life-years; EAPC, estimated annual percentage change; UI, uncertainty interval; CI, confidence interval.

TABLE S12 Incidence and DALYs of non-melanoma skin cancer (squamous-cell carcinoma) in 1990 and 2021 across 204 countries and territories, with age-standardized rates, 95% uncertainty intervals, and trends from 1990 to 2021.

Location name	Incidence				DALYs			
	Age-standardized rate (95% UI), 1990	Age-standardized rate (95% UI), 2021	EAPC (95% CI), 1990-2021	Change in age-standardized rate, 1990-2021	Age-standardized rate (95% UI), 1990	Age-standardized rate (95% UI), 2021	EAPC (95% CI), 1990-2021	Change in age-standardized rate, 1990-2021
Afghanistan	0.27 (0.2 to 0.36)	0.24 (0.18 to 0.32)	0 (0 to 0)	-10.14%	0.15 (0.03 to 1.25)	0.32 (0.11 to 2.18)	0 (0 to 0)	117.86%
Albania	2.85 (2.18 to 3.83)	3.01 (2.29 to 4.01)	0 (0 to 0)	5.45%	25.71 (19.39 to 33.68)	18.78 (12.69 to 27.55)	0 (0 to 0)	-26.96%
Algeria	1.02 (0.84 to 1.27)	1.04 (0.81 to 1.35)	0 (0 to 0)	1.71%	0.17 (0.06 to 1.02)	0.3 (0.12 to 1.87)	0 (0 to 0)	81.61%

American Samoa	0.02 (0.01 to 0.03)	0.02 (0.01 to 0.03)	0 (0 to 0)	-0.01%	12.1 (9.38 to 20.55)	19.16 (12.52 to 24.4)	0 (0 to 0)	58.43%
Andorra	6.5 (5.04 to 8.53)	6.62 (5.09 to 8.52)	0 (0 to 0)	1.78%	13.4 (9.27 to 18.38)	9.3 (6.25 to 13.25)	-0.91 (-1.08 to -0.74)	-30.62%
Angola	0.05 (0.04 to 0.08)	0.05 (0.04 to 0.07)	0 (0 to 0)	-3.48%	6.73 (2.29 to 10.58)	9.78 (2.88 to 15.91)	0 (0 to 0)	45.39%
Antigua and Barbuda	0.05 (0.03 to 0.07)	0.05 (0.04 to 0.07)	0 (0 to 0)	3.13%	1.86 (1.72 to 1.99)	18.04 (16.92 to 19.22)	0 (0 to 0)	869.92%
Argentina	3.12 (2.53 to 3.99)	3.26 (2.54 to 4.25)	0 (0 to 0)	4.34%	16.61 (15.55 to 17.76)	15.45 (14.36 to 16.6)	0 (0 to 0)	-6.98%
Armenia	0.06 (0.04 to 0.09)	0.06 (0.04 to 0.09)	0 (0 to 0)	0.81%	7.72 (6.34 to 9.27)	21.48 (17.65 to 25.56)	0 (0 to 0)	178.17%
Australia	87.78 (69.97 to 108.6)	68.22 (52.47 to 85.74)	0 (0 to 0)	-22.28%	33.48 (31.31 to 36.17)	31.17 (28.38 to 33.72)	0 (0 to 0)	-6.89%
Austria	3.15 (2.83 to 3.52)	3.95 (3.05 to 5.15)	0 (0 to 0)	25.63%	14.11 (13.25 to 14.99)	12.05 (10.84 to 13.04)	0 (0 to 0)	-14.64%
Azerbaijan	0.06 (0.04 to 0.09)	0.06 (0.04 to 0.09)	0 (0 to 0)	2.82%	9.56 (6.05 to 14.69)	10.08 (6.14 to 16.28)	0 (0 to 0)	5.36%
Bahamas	0.05 (0.03 to 0.07)	0.05 (0.04 to 0.08)	0 (0 to 0)	2.35%	12.39 (11.09 to 13.74)	15.23 (12.37 to 18.85)	0 (0 to 0)	22.89%
Bahrain	0.02 (0.01 to 0.03)	0.02 (0.01 to 0.03)	0 (0 to 0)	-9.29%	4.83 (4.14 to 6.84)	6.24 (4.5 to 7.98)	0 (0 to 0)	29.23%
Bangladesh	0.01 (0 to 0.02)	0.01 (0 to 0.02)	0 (0 to 0)	-0.47%	7.04 (4.15 to 10.24)	7.54 (4.35 to 12.23)	0 (0 to 0)	7.05%
Barbados	0.05 (0.03 to 0.07)	0.05 (0.04 to 0.07)	0 (0 to 0)	4.68%	7.4 (6.85 to 8.04)	7.12 (5.78 to 8.68)	0 (0 to 0)	-3.77%
Belarus	2.35 (2.17 to 2.53)	2.63 (2.02 to 3.41)	0 (0 to 0)	11.66%	17.88 (14.95 to 20.04)	15.45 (12.83 to 18.61)	0 (0 to 0)	-13.59%
Belgium	4.09 (3.4 to 5.01)	4.59 (3.57 to 5.85)	0 (0 to 0)	12.10%	11.31 (10.66 to 11.92)	10.34 (9.45 to 11.07)	0 (0 to 0)	-8.62%
Belize	0.05 (0.04 to 0.08)	0.05 (0.04 to 0.08)	0 (0 to 0)	1.53%	14.07 (12.57 to 15.63)	19.23 (16.83 to 21.87)	0 (0 to 0)	36.70%
Benin	0.02 (0.01 to 0.03)	0.02 (0.01 to 0.03)	0 (0 to 0)	0.20%	2.07 (1.01 to 2.82)	3.55 (0.97 to 5.94)	0 (0 to 0)	71.59%
Bermuda	0.01 (0 to 0.02)	0.01 (0 to 0.02)	-0.4 (-0.51 to -0.29)	-10.75%	5.69 (5.28 to 6.11)	15.31 (12.98 to 18.44)	3.28 (2.13 to 4.44)	169.13%
Bhutan	0.01 (0 to 0.02)	0.01 (0 to 0.02)	0 (0 to 0)	0.76%	5.73 (3.26 to 8.29)	7.12 (4.78 to 10.67)	0 (0 to 0)	24.16%
Bolivia (Plurinational State of)	0.2 (0.15 to 0.27)	0.2 (0.15 to 0.27)	0 (0 to 0)	1.16%	16.19 (11.5 to 23.9)	23.66 (15.62 to 33.66)	1.29 (1.11 to 1.47)	46.11%
Bosnia and Herzegovina	2.83 (2.17 to 3.76)	2.94 (2.25 to 3.95)	0 (0 to 0)	3.95%	10.69 (8.45 to 13.6)	9.43 (6.81 to 12.62)	0 (0 to 0)	-11.79%
Botswana	2.83 (2.17 to 3.68)	2.86 (2.22 to 3.64)	0 (0 to 0)	1.03%	7.37 (4.92 to 17.13)	8.55 (5.98 to 14.92)	0 (0 to 0)	15.92%

Brazil	3.28 (2.71 to 3.99)	3.14 (2.52 to 3.89)	0 (0 to 0)	-4.19%	20.42 (19.46 to 21.24)	25.37 (23.51 to 26.65)	0 (0 to 0)	24.23%
Brunei Darussalam	0.76 (0.57 to 1)	0.75 (0.57 to 1)	0 (0 to 0)	-1.48%	12.65 (9.55 to 15.89)	9.53 (7.45 to 12.27)	0 (0 to 0)	-24.66%
Bulgaria	3.37 (2.53 to 4.52)	3.7 (3.18 to 4.22)	0 (0 to 0)	9.82%	22.67 (20.49 to 24.97)	16.4 (14.09 to 18.92)	0 (0 to 0)	-27.68%
Burkina Faso	0.02 (0.01 to 0.03)	0.02 (0.01 to 0.03)	0 (0 to 0)	0.01%	2.1 (1 to 3)	3.47 (0.92 to 5.61)	0 (0 to 0)	64.77%
Burundi	0.05 (0.04 to 0.08)	0.06 (0.04 to 0.08)	0 (0 to 0)	6.76%	6.48 (2.06 to 10.58)	8.29 (1.88 to 14.77)	0 (0 to 0)	27.80%
Cabo Verde	0.02 (0.01 to 0.03)	0.02 (0.01 to 0.03)	0 (0 to 0)	1.69%	1.95 (1.38 to 2.53)	1.51 (0.96 to 2.29)	0 (0 to 0)	-22.64%
Cambodia	0.06 (0.04 to 0.08)	0.06 (0.04 to 0.08)	0 (0 to 0)	-2.09%	13.2 (9.15 to 18.26)	16.25 (11 to 21.77)	0 (0 to 0)	23.07%
Cameroon	0.02 (0.01 to 0.03)	0.02 (0.01 to 0.03)	0 (0 to 0)	-0.16%	2.54 (1.27 to 3.52)	4.46 (1.24 to 7.4)	0 (0 to 0)	75.55%
Canada	5.47 (4.28 to 6.89)	7.77 (5.83 to 10.28)	0 (0 to 0)	42.20%	12.96 (12.25 to 13.73)	14.76 (13.51 to 15.81)	0 (0 to 0)	13.81%
Central African Republic	0.05 (0.03 to 0.07)	0.05 (0.03 to 0.08)	0 (0 to 0)	-0.84%	7.01 (2.53 to 10.4)	8.87 (2.72 to 13.69)	0.85 (0.81 to 0.9)	26.42%
Chad	0.02 (0.01 to 0.03)	0.02 (0.01 to 0.03)	0 (0 to 0)	2.68%	1.72 (0.85 to 2.6)	3.68 (1.03 to 6.22)	0 (0 to 0)	114.47%
Chile	2.66 (2.09 to 3.41)	2.75 (2.09 to 3.61)	0 (0 to 0)	3.39%	10.19 (9.52 to 10.76)	18.24 (16.76 to 19.53)	1.86 (1.13 to 2.6)	79.03%
China	0.89 (0.73 to 1.14)	5.95 (4.61 to 7.66)	0 (0 to 0)	565.84%	16.34 (13.56 to 20.23)	17.97 (14.31 to 22.27)	0 (0 to 0)	9.96%
Colombia	1.23 (0.94 to 1.58)	1.21 (0.93 to 1.58)	0 (0 to 0)	-1.27%	26.6 (25.15 to 27.88)	17.17 (14.42 to 20.29)	0 (0 to 0)	-35.45%
Comoros	0.05 (0.04 to 0.08)	0.05 (0.04 to 0.08)	0 (0 to 0)	-1.11%	6.77 (2.14 to 11.09)	9.72 (3.09 to 16.41)	0 (0 to 0)	43.51%
Congo	0.05 (0.03 to 0.07)	0.05 (0.04 to 0.08)	0 (0 to 0)	4.10%	8.33 (3.65 to 11.49)	11 (3.79 to 16.32)	0 (0 to 0)	31.94%
Cook Islands	0.02 (0.01 to 0.03)	0.02 (0.01 to 0.03)	-0.01 (-0.01 to -0.01)	-0.22%	6.76 (5.31 to 10.12)	5.95 (4.49 to 8.04)	0 (0 to 0)	-12.03%
Costa Rica	3.1 (2.54 to 3.71)	1.29 (1 to 1.66)	0 (0 to 0)	-58.37%	22.08 (20.17 to 23.69)	17.62 (15.41 to 19.63)	0 (0 to 0)	-20.21%
Croatia	1.88 (1.47 to 2.51)	1.98 (1.54 to 2.59)	0 (0 to 0)	5.08%	29.12 (27.03 to 31.54)	13.52 (11.78 to 14.98)	0 (0 to 0)	-53.59%
Cuba	1.58 (1.31 to 1.89)	0.91 (0.74 to 1.14)	0 (0 to 0)	-42.02%	32.06 (30.45 to 33.79)	38.42 (32.91 to 43.45)	0.72 (0.62 to 0.83)	19.83%
Cyprus	2.36 (1.9 to 3.01)	2.44 (1.86 to 3.25)	0 (0 to 0)	3.36%	30.54 (21.66 to 41.51)	16.45 (12.87 to 21.35)	0 (0 to 0)	-46.14%
Czechia	6.04 (5.62 to 6.46)	6.69 (5.2 to 8.87)	0 (0 to 0)	10.65%	22.75 (21.07 to 24.18)	9.94 (8.57 to 11.44)	0 (0 to 0)	-56.32%
Côte d'Ivoire	0.01 (0.01 to 0.02)	0.01 (0 to 0.02)	0 (0 to 0)	-8.05%	2.28 (1.04 to 3.21)	4.23 (1.1 to 6.8)	0 (0 to 0)	85.62%

Democratic People's Republic of Korea	0.22 (0.16 to 0.29)	0.24 (0.18 to 0.32)	0 (0 to 0)	9.02%	13.36 (9.46 to 18.77)	16.57 (12.38 to 22.16)	0 (0 to 0)	23.98%
Democratic Republic of the Congo	0.05 (0.04 to 0.07)	0.05 (0.03 to 0.08)	0 (0 to 0)	-1.00%	6.34 (2.35 to 9.86)	9.13 (2.71 to 14.93)	0 (0 to 0)	44.13%
Denmark	7.36 (6.77 to 7.95)	6.93 (5.35 to 9.04)	0 (0 to 0)	-5.85%	9.95 (9.34 to 10.61)	10.59 (9.57 to 11.43)	0 (0 to 0)	6.37%
Djibouti	0.06 (0.04 to 0.08)	0.06 (0.04 to 0.08)	0 (0 to 0)	2.71%	6.56 (2.27 to 11.35)	10.23 (2.78 to 19.23)	0 (0 to 0)	55.95%
Dominica	0.05 (0.03 to 0.07)	0.05 (0.04 to 0.08)	0 (0 to 0)	9.62%	9.51 (7.59 to 12.45)	11.52 (8.5 to 14.87)	0 (0 to 0)	21.12%
Dominican Republic	0.05 (0.04 to 0.08)	0.05 (0.04 to 0.08)	0 (0 to 0)	-1.36%	12.47 (9.33 to 27.09)	28.55 (20.05 to 36.59)	0 (0 to 0)	128.92%
Ecuador	0.82 (0.66 to 1.02)	0.41 (0.31 to 0.53)	0 (0 to 0)	-50.02%	11.4 (10.62 to 12.13)	28.8 (23.59 to 35.13)	3.57 (3 to 4.15)	152.56%
Egypt	0.44 (0.35 to 0.57)	0.42 (0.32 to 0.54)	0 (0 to 0)	-4.72%	0.82 (0.55 to 2.68)	5.3 (3.15 to 6.62)	0 (0 to 0)	550.86%
El Salvador	1.22 (0.93 to 1.59)	1.19 (0.91 to 1.54)	0 (0 to 0)	-2.38%	9.52 (8.23 to 12.55)	11.27 (8.65 to 14.32)	0 (0 to 0)	18.35%
Equatorial Guinea	0.05 (0.03 to 0.07)	0.05 (0.03 to 0.07)	0 (0 to 0)	-0.85%	6.94 (2.45 to 10.72)	9.56 (2.93 to 15.92)	0 (0 to 0)	37.67%
Eritrea	0.05 (0.04 to 0.08)	0.05 (0.04 to 0.07)	0 (0 to 0)	0.67%	6.42 (2.36 to 9.89)	9.86 (3.35 to 15.73)	0 (0 to 0)	53.55%
Estonia	2.59 (2.32 to 2.87)	2.59 (1.98 to 3.35)	0 (0 to 0)	0.10%	21.12 (18.52 to 23.28)	15.43 (13.57 to 17.64)	0 (0 to 0)	-26.95%
Eswatini	2.79 (2.19 to 3.62)	2.72 (2.14 to 3.5)	0 (0 to 0)	-2.20%	7.07 (4.99 to 14.54)	10.51 (6.43 to 16.4)	0 (0 to 0)	48.79%
Ethiopia	0.07 (0.05 to 0.09)	0.06 (0.04 to 0.09)	0 (0 to 0)	-1.06%	7.07 (2.39 to 11.48)	8 (2.11 to 13.63)	0 (0 to 0)	13.26%
Fiji	0.02 (0.01 to 0.03)	0.02 (0.01 to 0.03)	0 (0 to 0)	-0.78%	8.46 (6.12 to 11.07)	7.84 (5.8 to 10.76)	0 (0 to 0)	-7.41%
Finland	5.57 (4.34 to 7.22)	6.12 (4.72 to 7.96)	0 (0 to 0)	9.93%	9.52 (8.87 to 10.04)	7.98 (7.22 to 8.6)	0 (0 to 0)	-16.18%
France	6.84 (6.02 to 7.73)	6.7 (5.16 to 8.7)	0 (0 to 0)	-2.10%	12.55 (11.87 to 13.16)	11.16 (10.08 to 12.09)	0 (0 to 0)	-11.08%
Gabon	0.05 (0.03 to 0.07)	0.05 (0.04 to 0.08)	0 (0 to 0)	1.78%	8.01 (2.91 to 11.36)	10.72 (3.37 to 16.14)	0 (0 to 0)	33.83%
Gambia	0.38 (0.28 to 0.52)	0.41 (0.29 to 0.56)	0 (0 to 0)	7.70%	2.27 (1.05 to 3.42)	4.68 (1.42 to 8.04)	2.47 (2.14 to 2.8)	106.16%
Georgia	0.06 (0.04 to 0.09)	0.06 (0.04 to 0.09)	0 (0 to 0)	1.64%	3.05 (2.62 to 3.6)	64.63 (55.83 to 73.7)	0 (0 to 0)	2019.12%
Germany	2.9 (2.59 to 3.25)	4.52 (3.51 to 5.98)	0 (0 to 0)	55.75%	8.6 (8.12 to 9.01)	8.91 (8.08 to 9.56)	0 (0 to 0)	3.59%
Ghana	0.02 (0.01 to 0.03)	0.02 (0.01 to 0.03)	0 (0 to 0)	-2.10%	2.85 (1.93 to 3.98)	3.22 (2.01 to 4.63)	0 (0 to 0)	13.23%
Greece	6.17 (4.78 to 7.97)	6.36 (4.99 to 8.22)	0 (0 to 0)	3.01%	16.22 (15.14 to 17.07)	15.61 (14.2 to 16.74)	0 (0 to 0)	-3.74%

Greenland	28.55 (21.88 to 37.08)	29.98 (23.06 to 39.61)	0.26 (0.21 to 0.31)	5.01%	8.1 (5.5 to 9.28)	5.64 (4.1 to 6.97)	0 (0 to 0)	-30.41%
Grenada	0.05 (0.03 to 0.07)	0.05 (0.04 to 0.07)	0 (0 to 0)	3.73%	1.24 (1.12 to 1.38)	16.46 (14.23 to 18.97)	0 (0 to 0)	1232.56%
Guam	0.02 (0.01 to 0.03)	0.02 (0.01 to 0.03)	0 (-0.01 to 0)	-0.16%	5.58 (4.32 to 11.74)	6.95 (4.13 to 8.35)	0 (0 to 0)	24.57%
Guatemala	1.25 (0.96 to 1.6)	1.24 (0.96 to 1.6)	0 (0 to 0)	-1.28%	29.76 (27.63 to 31.83)	19.54 (16.77 to 22.8)	0 (0 to 0)	-34.34%
Guinea	0.01 (0 to 0.02)	0.01 (0 to 0.02)	-0.33 (-0.4 to -0.27)	-7.10%	1.91 (1.05 to 2.82)	3.97 (1.19 to 6.59)	0 (0 to 0)	108.32%
Guinea-Bissau	0.02 (0.01 to 0.03)	0.02 (0.01 to 0.03)	-0.03 (-0.04 to -0.02)	-1.10%	2.75 (1.32 to 4.08)	4.8 (1.5 to 7.21)	0 (0 to 0)	74.50%
Guyana	0.05 (0.04 to 0.07)	0.05 (0.03 to 0.08)	0 (0 to 0)	-0.97%	1.27 (1.13 to 1.42)	15.09 (11.52 to 19.23)	0 (0 to 0)	1083.73%
Haiti	0.05 (0.04 to 0.08)	0.05 (0.04 to 0.08)	0 (0 to 0)	0.84%	10.56 (7.65 to 16.64)	15.9 (9.91 to 23.28)	0 (0 to 0)	50.53%
Honduras	1.26 (0.96 to 1.62)	1.25 (0.98 to 1.62)	0 (0 to 0)	-0.80%	11.92 (8.85 to 14.63)	15.06 (10.76 to 21.58)	0 (0 to 0)	26.34%
Hungary	3.27 (2.8 to 3.89)	3.05 (2.35 to 4.01)	0 (0 to 0)	-6.85%	25.91 (24.24 to 27.5)	13.34 (11.69 to 15.17)	0 (0 to 0)	-48.52%
Iceland	6.14 (4.76 to 7.96)	6.33 (4.88 to 8.24)	0 (0 to 0)	3.07%	4.52 (4.17 to 4.85)	4.88 (4.34 to 5.43)	0 (0 to 0)	8.05%
India	0.19 (0.14 to 0.25)	0.19 (0.14 to 0.25)	0 (0 to 0)	2.93%	6.71 (4.73 to 9.07)	7.87 (6.38 to 10.38)	0 (0 to 0)	17.24%
Indonesia	0.08 (0.06 to 0.11)	0.08 (0.06 to 0.11)	0 (0 to 0)	0.36%	10.87 (8.14 to 13.7)	14.83 (10.51 to 18.93)	0 (0 to 0)	36.37%
Iran (Islamic Republic of)	1.17 (0.91 to 1.54)	0.89 (0.7 to 1.15)	0 (0 to 0)	-23.98%	0.56 (0.12 to 0.74)	0.69 (0.12 to 0.88)	0 (0 to 0)	24.38%
Iraq	0.37 (0.27 to 0.48)	0.43 (0.36 to 0.53)	0 (0 to 0)	17.84%	7.99 (5.44 to 10.95)	9.01 (6.32 to 12.57)	0 (0 to 0)	12.87%
Ireland	11.82 (10.56 to 13.03)	9.68 (7.55 to 12.53)	0 (0 to 0)	-18.13%	29.28 (27.59 to 30.81)	17.26 (15.12 to 19.49)	0 (0 to 0)	-41.06%
Israel	6.56 (5.05 to 8.62)	6.59 (5.07 to 8.64)	0 (0 to 0)	0.52%	16.13 (15.09 to 16.98)	14.8 (13.18 to 16.02)	0 (0 to 0)	-8.23%
Italy	5.85 (4.56 to 7.64)	6.6 (5.15 to 8.62)	0 (0 to 0)	12.72%	12.26 (11.5 to 12.78)	10.79 (9.75 to 11.51)	0 (0 to 0)	-12.03%
Jamaica	0.04 (0.02 to 0.05)	0.02 (0.02 to 0.04)	0 (0 to 0)	-31.93%	5.11 (4.57 to 5.67)	6.15 (4.71 to 7.86)	0 (0 to 0)	20.24%
Japan	0.42 (0.32 to 0.55)	0.61 (0.45 to 0.82)	0 (0 to 0)	44.04%	4.86 (4.5 to 5.08)	4.86 (4.4 to 5.15)	0 (0 to 0)	0.00%
Jordan	1.5 (1.21 to 1.91)	1.17 (0.92 to 1.5)	0 (0 to 0)	-21.76%	5.05 (3.93 to 8.51)	6.98 (5.24 to 8.97)	0 (0 to 0)	38.25%
Kazakhstan	0.06 (0.04 to 0.09)	0.06 (0.04 to 0.09)	0 (0 to 0)	1.75%	13.05 (10.45 to 15.89)	19.86 (17.03 to 23)	0 (0 to 0)	52.18%
Kenya	0.23 (0.17 to 0.3)	0.2 (0.15 to 0.27)	0 (0 to 0)	-12.20%	5.36 (1.97 to 8.45)	8.89 (2.85 to 13.55)	0 (0 to 0)	65.79%

Kiribati	0.02 (0.01 to 0.03)	0.02 (0.01 to 0.03)	0 (0 to 0)	-0.09%	2.61 (1.96 to 3.49)	2.93 (2.24 to 4.01)	0 (0 to 0)	12.55%
Kuwait	0.3 (0.23 to 0.4)	0.29 (0.22 to 0.38)	0 (0 to 0)	-5.09%	2.89 (2.59 to 3.18)	4.02 (3.22 to 4.94)	3.39 (1.46 to 5.37)	38.98%
Kyrgyzstan	0.13 (0.1 to 0.18)	0.06 (0.04 to 0.09)	0 (0 to 0)	-54.71%	10.44 (8.76 to 12.26)	34.35 (28.6 to 40.62)	0 (0 to 0)	228.94%
Lao People's Democratic Republic	0.06 (0.04 to 0.09)	0.07 (0.05 to 0.09)	0 (0 to 0)	2.66%	12.01 (7.93 to 18.35)	13.17 (8.91 to 17.23)	0 (0 to 0)	9.66%
Latvia	2.03 (1.79 to 2.28)	2.11 (1.63 to 2.75)	0 (0 to 0)	3.86%	21.56 (19.2 to 23.91)	20.28 (17.86 to 22.95)	0 (0 to 0)	-5.91%
Lebanon	1.27 (0.98 to 1.64)	1.27 (0.99 to 1.63)	0 (0 to 0)	0.16%	4.1 (2.7 to 6.39)	4.2 (3.2 to 5.82)	0 (0 to 0)	2.43%
Lesotho	2.62 (2.05 to 3.34)	2.69 (2.12 to 3.49)	0 (0 to 0)	2.84%	5.91 (4.07 to 12.58)	11.23 (7.45 to 17.33)	2.82 (2.46 to 3.17)	90.08%
Liberia	0.02 (0.01 to 0.03)	0.02 (0.01 to 0.03)	-0.01 (-0.02 to 0.01)	-0.80%	2.04 (0.97 to 3.02)	3.8 (1.03 to 6.66)	0 (0 to 0)	85.79%
Libya	0.03 (0.02 to 0.04)	0.03 (0.02 to 0.04)	0 (0 to 0)	-2.14%	0.11 (0.01 to 1.02)	0.41 (0.09 to 3)	0 (0 to 0)	263.44%
Lithuania	2.86 (2.56 to 3.16)	2.75 (2.12 to 3.58)	0 (0 to 0)	-3.76%	15.29 (13.79 to 16.67)	14.81 (13.05 to 16.59)	0 (0 to 0)	-3.13%
Luxembourg	5.72 (4.5 to 7.55)	6.35 (4.88 to 8.31)	0 (0 to 0)	10.95%	12.32 (11.54 to 13.06)	9.45 (8.48 to 10.4)	0 (0 to 0)	-23.31%
Madagascar	0.05 (0.04 to 0.08)	0.05 (0.04 to 0.08)	0 (0 to 0)	-1.18%	5.99 (2.08 to 9.37)	7.47 (2.23 to 12.22)	0.83 (0.74 to 0.93)	24.74%
Malawi	0.02 (0.01 to 0.03)	0.02 (0.01 to 0.03)	0 (0 to 0)	-10.70%	6.05 (1.99 to 9.79)	9.76 (2.57 to 16.36)	0 (0 to 0)	61.33%
Malaysia	0.32 (0.25 to 0.42)	0.28 (0.21 to 0.36)	0 (0 to 0)	-14.36%	13.37 (9.88 to 16.3)	11.93 (9.8 to 15.03)	0 (0 to 0)	-10.77%
Maldives	0.08 (0.06 to 0.11)	0.07 (0.05 to 0.1)	0 (0 to 0)	-8.73%	4.45 (2.79 to 6.93)	2.98 (2.15 to 4)	-1.57 (-1.68 to -1.47)	-33.09%
Mali	0.64 (0.49 to 0.81)	0.68 (0.53 to 0.87)	0.3 (0.26 to 0.34)	6.53%	2.28 (1.21 to 3.15)	3.91 (1.22 to 6.61)	0 (0 to 0)	71.26%
Malta	6.31 (5.45 to 7.3)	6.31 (4.89 to 8.3)	0 (0 to 0)	-0.07%	11.2 (10.46 to 12.03)	9.87 (8.84 to 10.9)	0 (0 to 0)	-11.88%
Marshall Islands	0.02 (0.01 to 0.03)	0.02 (0.01 to 0.03)	0 (0 to 0)	0.15%	12 (9.05 to 17.47)	16.29 (11.27 to 22.91)	0 (0 to 0)	35.70%
Mauritania	0.02 (0.01 to 0.03)	0.02 (0.01 to 0.03)	0 (-0.01 to 0.01)	0.06%	2.28 (1.26 to 3.09)	3.8 (1.29 to 6.58)	0 (0 to 0)	67.15%
Mauritius	0.06 (0.04 to 0.08)	0.06 (0.04 to 0.09)	0 (0 to 0)	3.86%	1.95 (1.85 to 2.06)	5.01 (4.61 to 5.32)	0 (0 to 0)	156.49%
Mexico	1.27 (0.99 to 1.64)	1.27 (0.98 to 1.64)	0 (0 to 0)	-0.20%	28.78 (27.83 to 29.6)	16.91 (14.94 to 18.93)	0 (0 to 0)	-41.25%

Micronesia (Federated States of)	0.02 (0.01 to 0.03)	0.02 (0.01 to 0.03)	0 (0 to 0)	-1.51%	13.25 (9.77 to 19.08)	16.84 (11.37 to 22.92)	0 (0 to 0)	27.06%
Monaco	5.92 (4.59 to 7.67)	6.21 (4.84 to 8.23)	0.16 (0.15 to 0.17)	5.00%	6.12 (4.51 to 8.71)	6.34 (4.16 to 8.71)	0 (0 to 0)	3.62%
Mongolia	0.06 (0.04 to 0.09)	0.06 (0.04 to 0.09)	0 (0 to 0)	-2.23%	1.42 (0.75 to 6.5)	12.17 (8.43 to 16.64)	9.38 (8.36 to 10.41)	757.70%
Montenegro	2.85 (2.17 to 3.76)	2.91 (2.24 to 3.87)	0 (0 to 0)	2.02%	9.57 (7.32 to 12.48)	10.18 (7.92 to 13.3)	0.14 (-0.06 to 0.34)	6.38%
Morocco	0.09 (0.06 to 0.12)	0.09 (0.06 to 0.11)	0 (0 to 0)	-2.21%	0.09 (0.02 to 0.78)	0.24 (0.08 to 1.64)	0 (0 to 0)	150.91%
Mozambique	0.05 (0.04 to 0.08)	0.05 (0.04 to 0.08)	0 (0 to 0)	-2.10%	6.31 (1.85 to 10.46)	10.35 (2.43 to 17.53)	0 (0 to 0)	63.95%
Myanmar	0.06 (0.04 to 0.09)	0.06 (0.04 to 0.08)	0 (0 to 0)	-5.02%	12.16 (8.8 to 18.34)	12.2 (8.42 to 15.84)	-0.16 (-0.29 to -0.03)	0.26%
Namibia	2.16 (1.71 to 2.76)	2.36 (1.85 to 3.06)	0 (0 to 0)	9.42%	7 (5.02 to 15.13)	10.63 (7.51 to 17.77)	0 (0 to 0)	51.86%
Nauru	0.02 (0.01 to 0.03)	0.02 (0.01 to 0.03)	-0.01 (-0.02 to -0.01)	-0.41%	14.73 (9.43 to 22.19)	17.66 (10.95 to 28.87)	0 (0 to 0)	19.88%
Nepal	0.01 (0 to 0.02)	0.01 (0 to 0.02)	0 (0 to 0)	0.19%	5.87 (3.23 to 8.9)	7.11 (4.59 to 10.51)	0 (0 to 0)	20.99%
Netherlands	8.59 (7 to 10.7)	10.45 (8.13 to 13.62)	0 (0 to 0)	21.69%	8.81 (8.22 to 9.33)	8.2 (7.38 to 8.87)	0 (0 to 0)	-6.88%
New Zealand	65.93 (50.46 to 85.79)	68.8 (53.35 to 87.9)	0 (0 to 0)	4.35%	23.31 (21.13 to 25.85)	34.8 (30.98 to 38.07)	0 (0 to 0)	49.28%
Nicaragua	1.2 (0.92 to 1.54)	1.21 (0.91 to 1.57)	0 (0 to 0)	0.47%	11.17 (9.14 to 15.17)	12.54 (9.85 to 16.17)	0 (0 to 0)	12.27%
Niger	0.02 (0.01 to 0.03)	0.02 (0.01 to 0.03)	-0.04 (-0.05 to -0.04)	-1.69%	1.81 (0.86 to 2.7)	3.04 (0.81 to 5.37)	0 (0 to 0)	67.56%
Nigeria	0.02 (0.01 to 0.03)	0.02 (0.01 to 0.03)	0.06 (0.04 to 0.09)	1.16%	1.93 (0.96 to 2.61)	3.6 (0.95 to 5.73)	0 (0 to 0)	86.94%
Niue	0.02 (0.01 to 0.03)	0.02 (0.01 to 0.03)	0 (0 to 0)	0.04%	12.44 (9.03 to 16.71)	15.16 (9.74 to 20.56)	0 (0 to 0)	21.92%
North Macedonia	2.97 (2.22 to 3.99)	2.97 (2.3 to 3.99)	0 (0 to 0)	0.32%	21.12 (16.91 to 25.74)	19.78 (15.59 to 26.1)	0 (0 to 0)	-6.31%
Northern Mariana Islands	0.02 (0.01 to 0.03)	0.02 (0.01 to 0.03)	-0.01 (-0.01 to 0)	-0.27%	14.27 (10.58 to 22.39)	22.08 (12.25 to 26.6)	0 (0 to 0)	54.68%
Norway	6.23 (4.87 to 8.25)	6.61 (5.14 to 8.75)	0 (0 to 0)	6.05%	6.64 (6.24 to 6.94)	7.68 (6.82 to 8.28)	0 (0 to 0)	15.70%
Oman	0.95 (0.76 to 1.19)	0.93 (0.74 to 1.19)	0 (0 to 0)	-1.48%	3.84 (2.21 to 5.88)	4.41 (3.22 to 5.93)	0 (0 to 0)	14.94%
Pakistan	0.02 (0.01 to 0.03)	0.02 (0.01 to 0.03)	0 (0 to 0)	-26.68%	7.11 (4.61 to 10.17)	8.7 (6.7 to 11.99)	0 (0 to 0)	22.32%

Palau	0.02 (0.01 to 0.03)	0.02 (0.01 to 0.03)	0.01 (0 to 0.01)	0.21%	31.27 (23.34 to 41.75)	31.63 (22.92 to 44.05)	0 (0 to 0)	1.15%
Palestine	0.23 (0.17 to 0.3)	0.23 (0.17 to 0.32)	0 (0 to 0)	1.71%	9.73 (6.16 to 13)	10.52 (6.15 to 12.93)	0 (0 to 0)	8.10%
Panama	2.58 (2.37 to 2.8)	1.15 (0.89 to 1.49)	0 (0 to 0)	-55.40%	9.16 (8.44 to 9.94)	8.06 (6.34 to 9.65)	0 (0 to 0)	-12.03%
Papua New Guinea	0.02 (0.01 to 0.03)	0.02 (0.01 to 0.03)	0 (0 to 0)	-0.27%	8.95 (5.13 to 13.71)	11.78 (6.62 to 17.75)	0.97 (0.92 to 1.01)	31.71%
Paraguay	0.01 (0 to 0.02)	0.01 (0 to 0.02)	0 (0 to 0)	-11.00%	16.15 (11.81 to 21.84)	22.1 (15.85 to 29.17)	0 (0 to 0)	36.79%
Peru	0.25 (0.2 to 0.32)	0.09 (0.07 to 0.13)	0 (0 to 0)	-63.40%	10.79 (7.94 to 14.28)	16.11 (9.66 to 21.13)	0 (0 to 0)	49.36%
Philippines	0.39 (0.3 to 0.51)	0.38 (0.29 to 0.49)	0 (0 to 0)	-2.81%	12.04 (9.54 to 14.47)	12.43 (10.03 to 15.59)	0.11 (0.08 to 0.14)	3.25%
Poland	1.86 (1.43 to 2.49)	2.02 (1.55 to 2.7)	0 (0 to 0)	8.61%	29.48 (27.84 to 30.74)	2.61 (2.35 to 2.84)	0 (0 to 0)	-91.15%
Portugal	3.13 (2.46 to 4.02)	7.31 (5.59 to 9.55)	0 (0 to 0)	133.58%	22.07 (20.77 to 23.19)	15.65 (14.19 to 16.86)	0 (0 to 0)	-29.12%
Puerto Rico	0.05 (0.04 to 0.08)	0.05 (0.04 to 0.07)	-0.03 (-0.04 to -0.02)	-1.23%	5.41 (5.12 to 5.73)	20.39 (16.89 to 24.2)	0 (0 to 0)	277.13%
Qatar	0.05 (0.03 to 0.08)	0.05 (0.03 to 0.08)	0 (0 to 0)	-2.42%	2.34 (1.66 to 4.3)	2.92 (1.95 to 4.21)	0 (0 to 0)	24.92%
Republic of Korea	0.44 (0.37 to 0.52)	0.64 (0.54 to 0.76)	0 (0 to 0)	47.83%	22.44 (16.64 to 25.42)	7.76 (6.19 to 12.52)	0 (0 to 0)	-65.41%
Republic of Moldova	2.45 (1.87 to 3.26)	2.51 (1.91 to 3.34)	0 (0 to 0)	2.25%	20.47 (18.95 to 21.9)	17.72 (15.73 to 20.02)	0 (0 to 0)	-13.40%
Romania	1.47 (1.17 to 1.85)	1.59 (1.23 to 2.12)	0 (0 to 0)	8.74%	33.94 (32.19 to 35.73)	22.78 (20.01 to 25.55)	0 (0 to 0)	-32.88%
Russian Federation	1.7 (1.31 to 2.23)	2.12 (1.63 to 2.77)	0 (0 to 0)	24.72%	15.47 (15.01 to 15.79)	15.13 (13.96 to 16.23)	-0.5 (-1.04 to 0.04)	-2.23%
Rwanda	0.05 (0.04 to 0.07)	0.05 (0.04 to 0.07)	0 (0 to 0)	-1.15%	7.77 (2.83 to 11.68)	9.24 (2.67 to 15.99)	0 (0 to 0)	19.03%
Saint Kitts and Nevis	0.05 (0.03 to 0.07)	0.05 (0.04 to 0.08)	0.05 (0.03 to 0.06)	1.94%	2.7 (2.53 to 2.89)	26.64 (22.69 to 30.76)	8.18 (5.68 to 10.74)	885.95%
Saint Lucia	0.05 (0.03 to 0.07)	0.05 (0.04 to 0.08)	0 (0 to 0)	4.30%	3.01 (2.8 to 3.24)	8.83 (7.28 to 10.56)	0 (0 to 0)	192.72%
Saint Vincent and the Grenadines	0.05 (0.03 to 0.07)	0.05 (0.04 to 0.08)	0 (0 to 0)	8.09%	2.7 (2.49 to 2.9)	22.58 (19.84 to 25.54)	0 (0 to 0)	737.76%
Samoa	0.02 (0.01 to 0.03)	0.02 (0.01 to 0.03)	0 (0 to 0)	-4.49%	12.57 (8.58 to 17.78)	15.94 (11.25 to 22.6)	0 (0 to 0)	26.81%
San Marino	6.1 (4.78 to 7.97)	6.33 (4.93 to 8.38)	0.14 (0.12 to 0.15)	3.67%	8.44 (6.64 to 11.07)	5.38 (3.36 to 7.84)	0 (0 to 0)	-36.32%

Sao Tome and Principe	0.02 (0.01 to 0.03)	0.02 (0.01 to 0.03)	0.01 (0.01 to 0.02)	1.05%	0.52 (0.37 to 0.65)	0.59 (0.41 to 0.79)	0 (0 to 0)	14.51%
Saudi Arabia	0.44 (0.33 to 0.59)	0.58 (0.5 to 0.67)	0 (0 to 0)	33.35%	1.93 (1.29 to 3.11)	3.35 (2.48 to 4.34)	0 (0 to 0)	73.96%
Senegal	0.09 (0.04 to 0.16)	0.09 (0.04 to 0.16)	0.05 (0.03 to 0.06)	0.41%	2.25 (1.11 to 3.19)	4.25 (1.37 to 7.21)	0 (0 to 0)	89.14%
Serbia	2.54 (2.31 to 2.8)	2.65 (2.02 to 3.58)	0 (0 to 0)	4.34%	31.55 (25.19 to 38.7)	22.94 (18.53 to 29.21)	0 (0 to 0)	-27.29%
Seychelles	0.01 (0 to 0.02)	0.01 (0 to 0.02)	0 (0 to 0)	3.42%	6.47 (5.3 to 11.92)	9.1 (6.62 to 10.97)	0 (0 to 0)	40.63%
Sierra Leone	0.02 (0.01 to 0.03)	0.02 (0.01 to 0.03)	0 (-0.02 to 0.03)	-0.09%	1.88 (0.82 to 2.79)	3.62 (0.97 to 5.96)	0 (0 to 0)	92.73%
Singapore	1.09 (0.94 to 1.26)	1.05 (0.8 to 1.37)	0 (0 to 0)	-3.37%	8.51 (7.97 to 8.94)	3.69 (3.31 to 4)	0 (0 to 0)	-56.58%
Slovakia	4.51 (4.23 to 4.8)	3.84 (2.97 to 5.09)	0 (0 to 0)	-14.99%	18.39 (13.86 to 21.64)	11.72 (9.46 to 15.92)	-1.47 (-1.58 to -1.37)	-36.28%
Slovenia	3.22 (2.94 to 3.51)	5.68 (4.49 to 6.83)	0 (0 to 0)	76.22%	15.86 (14.7 to 17.08)	7.03 (6.04 to 8.08)	0 (0 to 0)	-55.65%
Solomon Islands	0.02 (0.01 to 0.03)	0.02 (0.01 to 0.03)	0 (0 to 0)	0.32%	10.9 (6.67 to 17.56)	15.39 (9.71 to 22.97)	1.24 (1.18 to 1.3)	41.27%
Somalia	0.05 (0.04 to 0.08)	0.05 (0.04 to 0.07)	0 (0 to 0)	-3.08%	5.71 (1.81 to 9.99)	7.33 (1.79 to 13)	0 (0 to 0)	28.28%
South Africa	6.73 (5.27 to 8.56)	10.03 (7.86 to 12.85)	0 (0 to 0)	49.10%	11.91 (9.07 to 16.51)	18.06 (12.96 to 20.58)	0 (0 to 0)	51.63%
South Sudan	0.06 (0.04 to 0.08)	0.06 (0.04 to 0.08)	0 (0 to 0)	-5.23%	5.88 (1.88 to 10.38)	8.41 (2.18 to 15.01)	0 (0 to 0)	42.90%
Spain	6.79 (6.42 to 7.13)	8.56 (6.7 to 11.06)	0 (0 to 0)	26.16%	20.78 (19.3 to 21.93)	12.37 (11.18 to 13.37)	0 (0 to 0)	-40.48%
Sri Lanka	0.09 (0.07 to 0.12)	0.06 (0.04 to 0.08)	0 (0 to 0)	-34.46%	26.16 (19.75 to 31.07)	13.87 (8.99 to 22.11)	-2.64 (-2.95 to -2.33)	-46.98%
Sudan	0.27 (0.2 to 0.35)	0.29 (0.22 to 0.38)	0 (0 to 0)	6.35%	0.11 (0.02 to 0.88)	0.25 (0.08 to 1.63)	0 (0 to 0)	123.36%
Suriname	0.05 (0.04 to 0.08)	0.05 (0.03 to 0.07)	0 (0 to 0)	-2.65%	5.73 (4.62 to 8.49)	8.51 (5.71 to 11.54)	0 (0 to 0)	48.51%
Sweden	6.28 (4.91 to 8.31)	6.65 (5.2 to 8.77)	0 (0 to 0)	5.97%	8.19 (7.74 to 8.62)	7.68 (6.77 to 8.59)	0 (0 to 0)	-6.30%
Switzerland	9.8 (9.34 to 10.25)	8.69 (6.77 to 11.04)	0 (0 to 0)	-11.32%	9.26 (8.68 to 9.81)	8.73 (7.74 to 9.5)	0 (0 to 0)	-5.71%
Syrian Arab Republic	0.27 (0.2 to 0.36)	0.27 (0.2 to 0.36)	0 (0 to 0)	2.09%	0.07 (0.05 to 0.08)	0.07 (0.05 to 0.08)	0 (0 to 0)	1.61%
Taiwan (Province of China)	0.41 (0.38 to 0.44)	0.5 (0.4 to 0.63)	0 (0 to 0)	20.04%	15.41 (14.7 to 16.13)	9.36 (8.61 to 10.09)	0 (0 to 0)	-39.25%
Tajikistan	0.06 (0.04 to 0.09)	0.06 (0.04 to 0.09)	0 (0 to 0)	4.00%	11.61 (7.13 to 17.99)	9.08 (5.84 to 13.81)	0 (0 to 0)	-21.81%
Thailand	0.94 (0.84 to 1.06)	0.73 (0.58 to 0.92)	0 (0 to 0)	-22.99%	18.1 (14.32 to 24.29)	16.55 (12.22 to 22.93)	0 (0 to 0)	-8.56%

Timor-Leste	0.07 (0.05 to 0.09)	0.07 (0.05 to 0.09)	0 (0 to 0)	-0.20%	9.39 (5.73 to 14.36)	12.41 (7.83 to 18.2)	0 (0 to 0)	32.06%
Togo	0.02 (0.01 to 0.03)	0.02 (0.01 to 0.03)	-0.06 (-0.07 to -0.05)	-1.29%	2.26 (1.13 to 3.06)	4.08 (1.21 to 6.55)	2.15 (1.85 to 2.45)	80.25%
Tokelau	0.02 (0.01 to 0.03)	0.02 (0.01 to 0.03)	0 (0 to 0.01)	0.09%	13.25 (9.77 to 18.55)	15.16 (11.02 to 20.54)	0 (0 to 0)	14.40%
Tonga	0.02 (0.01 to 0.03)	0.02 (0.01 to 0.03)	0 (0 to 0)	0.50%	51.82 (38.83 to 68.72)	54.5 (41.14 to 73.52)	0 (0 to 0)	5.16%
Trinidad and Tobago	0.5 (0.41 to 0.62)	0.53 (0.42 to 0.66)	0 (0 to 0)	5.80%	7.16 (6.67 to 7.65)	6.99 (5.4 to 8.78)	0 (0 to 0)	-2.37%
Tunisia	0.49 (0.37 to 0.65)	0.43 (0.33 to 0.58)	0 (0 to 0)	-11.54%	0.14 (0.03 to 0.92)	0.25 (0.08 to 1.77)	0 (0 to 0)	86.98%
Turkmenistan	0.06 (0.04 to 0.09)	0.06 (0.04 to 0.09)	0 (0 to 0)	1.86%	2.07 (1.78 to 2.41)	23.92 (18.37 to 31.79)	10.9 (9.32 to 12.52)	1055.47%
Tuvalu	0.02 (0.01 to 0.03)	0.02 (0.01 to 0.03)	0.01 (0.01 to 0.01)	0.27%	12.89 (9.48 to 17.82)	16.71 (11.93 to 22)	0 (0 to 0)	29.60%
Türkiye	1.62 (1.38 to 1.92)	1.4 (1.09 to 1.81)	0 (0 to 0)	-13.70%	16.34 (12.76 to 22.15)	13.53 (10.61 to 18.19)	0 (0 to 0)	-17.22%
Uganda	0.58 (0.46 to 0.73)	0.64 (0.51 to 0.8)	0 (0 to 0)	9.27%	4.97 (1.45 to 8.42)	8.08 (2.16 to 13.75)	0 (0 to 0)	62.51%
Ukraine	2.5 (1.95 to 3.27)	2.66 (2.04 to 3.51)	0 (0 to 0)	6.08%	16.73 (14.24 to 18.56)	17.44 (12.81 to 22.53)	0 (0 to 0)	4.23%
United Arab Emirates	0.28 (0.21 to 0.37)	0.38 (0.28 to 0.5)	0 (0 to 0)	33.71%	10.13 (6.77 to 14.44)	9.86 (7.38 to 13.14)	0 (0 to 0)	-2.62%
United Kingdom	5.97 (4.78 to 7.64)	7.02 (5.5 to 9.15)	0 (0 to 0)	17.62%	12.05 (11.53 to 12.41)	13.61 (12.65 to 14.21)	0 (0 to 0)	13.00%
United Republic of Tanzania	0.05 (0.04 to 0.08)	0.05 (0.04 to 0.08)	0 (0 to 0)	0.01%	6.48 (2.21 to 10.7)	8.47 (2.5 to 14.45)	0 (0 to 0)	30.70%
United States Virgin Islands	0.05 (0.04 to 0.08)	0.05 (0.04 to 0.08)	0.03 (0.02 to 0.03)	0.85%	4.95 (3.62 to 11.71)	7.55 (4.6 to 10.01)	0 (0 to 0)	52.63%
United States of America	116.9 (91.36 to 150.17)	269.15 (242.05 to 299.5)	0 (0 to 0)	130.23%	24.1 (22.24 to 26.54)	28.17 (23.95 to 33.48)	0 (0 to 0)	16.86%
Uruguay	3.68 (3.15 to 4.32)	4.01 (3.06 to 5.36)	0 (0 to 0)	8.98%	10.96 (10.2 to 11.65)	12.64 (11.69 to 13.54)	0 (0 to 0)	15.33%
Uzbekistan	0.06 (0.04 to 0.09)	0.06 (0.04 to 0.09)	0 (0 to 0)	2.42%	8.98 (6.29 to 11.51)	13.8 (11.04 to 16.89)	0 (0 to 0)	53.69%
Vanuatu	0.02 (0.01 to 0.03)	0.02 (0.01 to 0.03)	0 (0 to 0)	-0.84%	10.52 (6.88 to 16.22)	14.02 (9.5 to 20.08)	0.89 (0.8 to 0.98)	33.24%
Venezuela (Bolivarian Republic of)	1.21 (0.93 to 1.56)	1.22 (0.94 to 1.57)	0 (0 to 0)	0.75%	31.16 (29.3 to 33.01)	32.72 (25.32 to 41.9)	-0.02 (-0.19 to 0.14)	5.02%
Viet Nam	0.04 (0.03 to 0.06)	0.02 (0.01 to 0.04)	0 (0 to 0)	-44.58%	12.11 (7.89 to 16.74)	14.8 (9.78 to 19.33)	0 (0 to 0)	22.30%

Yemen	0.22 (0.17 to 0.3)	0.25 (0.19 to 0.34)	0 (0 to 0)	11.78%	0.1 (0.02 to 0.77)	0.24 (0.08 to 1.56)	3.52 (3.15 to 3.88)	135.27%
Zambia	0.06 (0.04 to 0.08)	0.05 (0.04 to 0.08)	0 (0 to 0)	-2.63%	6.98 (2.43 to 10.57)	10.01 (3 to 15.49)	0 (0 to 0)	43.34%
Zimbabwe	1.79 (1.41 to 2.27)	1.32 (1.02 to 1.69)	0 (0 to 0)	-26.50%	6.96 (4.43 to 15.95)	11.23 (8.36 to 18.71)	0 (0 to 0)	61.39%

DALYs, disability-adjusted life-years; EAPC, estimated annual percentage change; UI, uncertainty interval; CI, confidence interval.

TABLE S13 Proportions of age-standardized incidence rates of skin cancers globally and by region in 1990 and 2021.

Location name	Malignant skin melanoma		Non-melanoma skin cancer (basal-cell carcinoma)		Non-melanoma skin cancer (squamous-cell carcinoma)	
	proportion, 1990	proportion, 2021	proportion, 1990	proportion, 2021	proportion, 1990	proportion, 2021
Global	6.21%	4.58%	65.94%	66.60%	27.85%	28.82%
Andean Latin America	5.50%	11.23%	92.18%	87.28%	2.32%	1.49%
Australasia	20.66%	25.35%	20.37%	21.18%	58.97%	53.47%
Caribbean	9.07%	16.42%	81.94%	78.21%	8.99%	5.36%
Central Asia	4.60%	4.60%	95.14%	95.15%	0.26%	0.25%
Central Europe	12.24%	21.29%	78.34%	70.73%	9.43%	7.98%
Central Latin America	2.24%	4.56%	93.38%	91.35%	4.38%	4.09%
Central Sub-Saharan Africa	9.87%	11.82%	89.10%	87.18%	1.03%	1.01%
East Asia	7.42%	1.84%	74.81%	82.58%	17.77%	15.58%
Eastern Europe	13.08%	23.24%	77.07%	68.28%	9.85%	8.48%
Eastern Sub-Saharan Africa	19.89%	22.48%	77.65%	74.99%	2.46%	2.52%
High-income Asia Pacific	22.29%	28.92%	66.81%	61.08%	10.90%	10.00%
High-income North America	5.14%	2.27%	62.76%	64.80%	32.10%	32.92%
North Africa and Middle East	10.70%	24.58%	80.28%	67.10%	9.02%	8.32%

Oceania	68.61%	68.15%	28.04%	28.44%	3.36%	3.41%
South Asia	13.48%	17.86%	77.28%	73.70%	9.24%	8.44%
Southeast Asia	7.05%	12.07%	83.82%	76.49%	9.13%	11.44%
Southern Latin America	5.46%	10.72%	83.17%	77.96%	11.37%	11.31%
Southern Sub-Saharan Africa	5.99%	6.60%	69.43%	64.90%	24.58%	28.50%
Tropical Latin America	1.83%	4.49%	93.52%	88.35%	4.65%	7.16%
Western Europe	14.49%	24.66%	74.09%	63.54%	11.42%	11.80%
Western Sub-Saharan Africa	13.73%	15.95%	84.85%	82.60%	1.42%	1.45%

TABLE S14 Proportions of age-standardized DALY rates of skin cancers globally and by region in 1990 and 2021.

Location name	Malignant skin melanoma		Non-melanoma skin cancer (basal-cell carcinoma)		Non-melanoma skin cancer (squamous-cell carcinoma)	
	proportion, 1990	proportion, 2021	proportion, 1990	proportion, 2021	proportion, 1990	proportion, 2021
Global	65.71%	58.05%	0.04%	0.07%	34.25%	41.88%
Andean Latin America	65.53%	50.17%	0.03%	0.02%	34.44%	49.81%
Australasia	85.88%	74.97%	0.01%	0.01%	14.11%	25.02%
Caribbean	42.40%	34.07%	0.01%	0.01%	57.59%	65.92%
Central Asia	70.05%	44.34%	0.04%	0.04%	29.91%	55.62%
Central Europe	67.19%	80.78%	0.02%	0.02%	32.80%	19.20%
Central Latin America	37.12%	50.65%	0.04%	0.04%	62.84%	49.31%
Central Sub-Saharan Africa	71.46%	65.35%	0.01%	0.01%	28.52%	34.64%
East Asia	38.95%	30.31%	0.01%	0.07%	61.04%	69.63%
Eastern Europe	70.22%	75.72%	0.02%	0.02%	29.76%	24.26%
Eastern Sub-Saharan Africa	85.19%	81.80%	0.01%	0.00%	14.80%	18.20%
High-income Asia Pacific	50.06%	47.89%	0.02%	0.03%	49.93%	52.08%

High-income North America	76.46%	62.53%	0.09%	0.26%	23.45%	37.21%
North Africa and Middle East	70.44%	63.96%	0.04%	0.04%	29.52%	36.00%
Oceania	47.66%	41.59%	0.00%	0.00%	52.34%	58.41%
South Asia	52.38%	48.98%	0.00%	0.00%	47.62%	50.02%
Southeast Asia	29.35%	28.17%	0.01%	0.00%	70.64%	71.83%
Southern Latin America	64.72%	63.62%	0.03%	0.03%	35.25%	36.35%
Southern Sub-Saharan Africa	77.26%	72.68%	0.02%	0.02%	22.72%	27.30%
Tropical Latin America	60.80%	52.11%	0.05%	0.03%	39.14%	47.85%
Western Europe	79.46%	77.98%	0.04%	0.04%	20.50%	21.98%
Western Sub-Saharan Africa	85.71%	76.86%	0.01%	0.01%	14.28%	23.13%

DALYs, disability-adjusted life-years.

TABLE S15 The number of cases and age-standardized rate of incidence and DALYs of total skin cancer by age group and sex in 1990 and 2021, with age-standardized rates and 95% uncertainty intervals.

Age group	Sex	Incidence				DALYs			
		Number of cases (95% UI), 1990	Age-standardized rate (95% UI), 1990	Number of cases (95% UI), 2021	Age-standardized rate (95% UI), 2021	Number of cases (95% UI), 1990	Age-standardized rate (95% UI), 1990	Number of cases (95% UI), 2021	Age-standardized rate (95% UI), 2021
<5 years	Female	0 (0 to 0)	0 (0 to 0)	0 (0 to 0)	0 (0 to 0)	0 (0 to 0)	0 (0 to 0)	0 (0 to 0)	0 (0 to 0)

<5 years	Male	0 (0 to 0)	0 (0 to 0)	0 (0 to 0)	0 (0 to 0)	0 (0 to 0)	0 (0 to 0)	0 (0 to 0)	0 (0 to 0)
5-9 years	Female	0 (0 to 0)	0 (0 to 0)	0 (0 to 0)	0 (0 to 0)	0 (0 to 0)	0 (0 to 0)	0 (0 to 0)	0 (0 to 0)
5-9 years	Male	0 (0 to 0)	0 (0 to 0)	0 (0 to 0)	0 (0 to 0)	0 (0 to 0)	0 (0 to 0)	0 (0 to 0)	0 (0 to 0)
10-14 years	Female	0 (0 to 0)	0 (0 to 0)	0 (0 to 0)	0 (0 to 0)	0 (0 to 0)	0 (0 to 0)	0 (0 to 0)	0 (0 to 0)
10-14 years	Male	0 (0 to 0)	0 (0 to 0)	0 (0 to 0)	0 (0 to 0)	0 (0 to 0)	0 (0 to 0)	0 (0 to 0)	0 (0 to 0)
15-19 years	Female	480.24 (426.01 to 524.03)	0.19 (0.17 to 0.21)	684.9 (543.53 to 820.37)	0.23 (0.18 to 0.27)	5789.37 (4127.06 to 6996.05)	2.27 (1.62 to 2.74)	6438.87 (4018.29 to 9483.42)	2.12 (1.32 to 3.12)
15-19 years	Male	314.02 (276.35 to 340.43)	0.12 (0.1 to 0.13)	403.91 (338.8 to 461.63)	0.13 (0.11 to 0.14)	5692.1 (4303.94 to 6627.45)	2.16 (1.63 to 2.51)	5137.85 (3574.23 to 6572.98)	1.6 (1.12 to 2.05)
20-24 Female		5755.8 (3773.97 to	2.36 (1.55 to 3.31)	7692.98 (5346.09 to	2.62 (1.82 to 3.62)	16952.75 (13351.9	6.94 (5.47 to 8.33)	19481.96 (13423.15	6.63 (4.57 to 8.86)

years	e	8071.89)		10642.17)		to 20334.27)		to 26023.58)	
20-24 years	Male	4718.74 (2983.79 to 6981.93)	1.9 (1.2 to 2.82)	6318.14 (4129.17 to 9111.98)	2.08 (1.36 to 3)	16677.07 (13707.44 to 19048.22)	6.73 (5.53 to 7.68)	16225.09 (12139 to 19465.77)	5.35 (4 to 6.42)
25-29 years	Female	11062.27 (7439.76 to 15813.96)	5.03 (3.38 to 7.18)	15855.44 (11022.81 to 21874.65)	5.45 (3.79 to 7.52)	27006.23 (22603.14 to 31122.5)	12.27 (10.27 to 14.14)	28376.08 (21417.38 to 36388.86)	9.75 (7.36 to 12.51)
25-29 years	Male	8237.43 (5323.96 to 12218.8)	3.7 (2.39 to 5.49)	11781.61 (7686.96 to 17144.13)	3.96 (2.59 to 5.77)	27627.16 (24241.88 to 31120.07)	12.42 (10.89 to 13.99)	27505.99 (21355.2 to 32536.22)	9.25 (7.18 to 10.94)
30-34 years	Female	18303.51 (13887.82 to 24725.69)	9.63 (7.31 to 13.01)	33291.47 (26138.88 to 42858.55)	11.14 (8.74 to 14.34)	37524.12 (32203.91 to 42253.39)	19.74 (16.94 to 22.23)	43404.13 (33600.89 to 53160.55)	14.52 (11.24 to 17.78)
30-34 years	Male	14011.6 (10427.47 to 18957.29)	7.17 (5.34 to 9.71)	23973.6 (18208.25 to 32005.29)	7.85 (5.96 to 10.47)	47163.69 (41148.69 to 51783.45)	24.15 (21.07 to 26.51)	54443.21 (39342.15 to 64729.85)	17.82 (12.88 to 21.18)
35-39 years	Female	26381.41 (17296.48 to 37948.96)	15.21 (9.97 to 21.88)	53777.03 (39236.74 to 72143.08)	19.36 (14.12 to 25.97)	49436.67 (43042.57 to 55826.72)	28.5 (24.82 to 32.19)	57765.11 (46055.11 to 70446.16)	20.79 (16.58 to 25.36)

35-39 years	Male	20795.25 (13529.86 to 30602.49)	11.63 (7.57 to 17.12)	38219.15 (27643.98 to 52897.79)	13.5 (9.77 to 18.69)	64275.93 (56156.1 to 70701.41)	35.95 (31.41 to 39.54)	69214.39 (53929.91 to 81806.52)	24.45 (19.05 to 28.9)
40-44 years	Female	35861.98 (26610.74 to 47225.25)	25.57 (18.98 to 33.68)	82488.95 (65897.32 to 101306.88)	33.25 (26.56 to 40.83)	55470.07 (48962.01 to 61958.23)	39.56 (34.92 to 44.18)	68733.87 (56954.16 to 81105.62)	27.71 (22.96 to 32.69)
40-44 years	Male	31084.52 (23103.74 to 40833.49)	21.25 (15.8 to 27.92)	66288.09 (52064.49 to 82702.52)	26.29 (20.65 to 32.8)	77431.61 (66904.57 to 84759.98)	52.94 (45.74 to 57.95)	91751.69 (72053.24 to 108166.7)	36.39 (28.57 to 42.9)
45-49 years	Female	41434.92 (27014.26 to 59696.36)	36.41 (23.74 to 52.46)	124498.65 (90935.27 to 167267.34)	52.83 (38.59 to 70.98)	53819.95 (47623.54 to 59724.87)	47.29 (41.85 to 52.48)	81365.87 (67994.55 to 97066.64)	34.53 (28.85 to 41.19)
45-49 years	Male	38961.62 (25462.86 to 56076.62)	32.91 (21.51 to 47.36)	109158.2 (78394.51 to 148337.63)	45.89 (32.96 to 62.36)	71876.93 (63234.06 to 78447.03)	60.71 (53.41 to 66.26)	102178.8 (82899.58 to 119251.55)	42.96 (34.85 to 50.13)
50-54 years	Female	49404.61 (36127 to 67285.88)	47.09 (34.43 to 64.13)	180689.09 (144084.27 to 228412)	81.05 (64.63 to 102.45)	62540.71 (55653.75 to 70274.11)	59.61 (53.05 to 66.98)	103167.88 (84979.56 to 119844.84)	46.28 (38.12 to 53.76)
50-54 years	Male	52605.86 (38553.67 to 71473.77)	48.87 (35.81 to 66.39)	189706.3 (149352.43 to	85.46 (67.28 to 106.29)	89343.32 (78493.69 to	82.99 (72.91 to 91.04)	139053.32 (113769.41 to	62.64 (51.25 to 71.54)

				235934.37)		98012.52)		158803.09)	
55-59 years	Female	58547.46 (37697.63 to 85203.53)	63.42 (40.84 to 92.3)	235953.74 (174115.6 to 307512.94)	117.39 (86.62 to 152.99)	64723.93 (57254.51 to 73531.49)	70.11 (62.02 to 79.66)	120271.34 (100097.67 to 139532.26)	59.84 (49.8 to 69.42)
55-59 years	Male	69592.11 (44524.58 to 102720.9)	74.92 (47.93 to 110.59)	289334.22 (210376.66 to 378746.84)	148.59 (108.04 to 194.5)	97391.13 (85257.81 to 107447.78)	104.85 (91.79 to 115.67)	167457.46 (141641.55 to 190453.54)	86 (72.74 to 97.81)
60-64 years	Female	82881.68 (59904.85 to 113714.66)	101 (73 to 138.57)	305399.21 (239788.82 to 378582.37)	185.64 (145.76 to 230.13)	67935.49 (61267.03 to 75656.5)	82.78 (74.66 to 92.19)	121282.48 (104121.17 to 138428.41)	73.72 (63.29 to 84.15)
60-64 years	Male	107636.21 (80454.36 to 143589.61)	137.04 (102.43 to 182.81)	444664.85 (362156.38 to 540460.96)	285.89 (232.84 to 347.48)	109332.25 (97463.31 to 119769.9)	139.19 (124.08 to 152.48)	193637.25 (166818.59 to 218546.22)	124.5 (107.25 to 140.51)
65-69 years	Female	109968.81 (84316.18 to 137904.64)	165.92 (127.22 to 208.07)	390544.13 (333137.02 to 451578.24)	271.19 (231.33 to 313.58)	65992.07 (60132.38 to 72548.16)	99.57 (90.73 to 109.46)	130639.24 (112190.99 to 148384.55)	90.72 (77.91 to 103.04)
65-69 years	Male	146701.81 (114298.09 to 185630.57)	255.88 (199.36 to 323.79)	626138.27 (535310.28 to 718500.87)	474.95 (406.05 to 545.01)	98490.65 (87718.25 to 109975.1)	171.79 (153 to 191.82)	208708.28 (178614.12 to 237880.56)	158.31 (135.48 to 180.44)

70-74 years	Female	111853.74 (83634.38 to 148361.79)	237.77 (177.78 to 315.38)	418809.89 (351066.06 to 493811.33)	382.66 (320.76 to 451.18)	53918.97 (48900.75 to 59912.66)	114.62 (103.95 to 127.36)	125230.8 (108903.44 to 142256.44)	114.42 (99.5 to 129.98)
70-74 years	Male	145750.53 (107680.35 to 192886.83)	387.44 (286.24 to 512.74)	694489.9 (586984.84 to 819031.21)	720.49 (608.96 to 849.69)	74095.69 (66069.9 to 81051.25)	196.97 (175.63 to 215.46)	195972.44 (171336.21 to 218724.04)	203.31 (177.75 to 226.91)
75-79 years	Female	114794.59 (81428.53 to 158127.08)	316.04 (224.18 to 435.34)	346060.19 (282837.76 to 416648.02)	479.99 (392.3 to 577.89)	52203.82 (47748.87 to 56636)	143.72 (131.46 to 155.92)	98720.74 (85956.89 to 111202.05)	136.93 (119.22 to 154.24)
75-79 years	Male	133498.68 (94955.85 to 180786.32)	529.07 (376.32 to 716.47)	563507.1 (464415.41 to 677247.74)	942.53 (776.79 to 1132.77)	59167.86 (53665.35 to 64577.99)	234.49 (212.68 to 255.93)	147470.02 (129137.8 to 164558.53)	246.66 (216 to 275.24)
80-84 years	Female	92620.01 (70109.54 to 121035.71)	419.24 (317.35 to 547.86)	270592.98 (226289.7 to 318036.84)	531.29 (444.3 to 624.44)	39420.19 (35015.15 to 42947.04)	178.43 (158.49 to 194.4)	90514.91 (76290.78 to 101712.17)	177.72 (149.79 to 199.71)
80-84 years	Male	92670.27 (71098.61 to 118342.26)	697.64 (535.24 to 890.9)	409952.47 (348981.68 to 475103.1)	1118.5 (952.15 to 1296.26)	36274.34 (32830.37 to 39686.23)	273.08 (247.15 to 298.76)	116718.16 (100732.24 to 128893.84)	318.45 (274.84 to 351.67)
85-89	Femal	57619.04 (43032.49	573.49 (428.31 to	179477.92 (150252.19 to	630.43 (527.77 to	24327.81 (21002.57 to	242.14 (209.04 to	68026.77 (54147.2	238.95 (190.2 to 272)

years	e	to 75799.88)	754.45)	213924.76)	751.43)	26771.89)	266.47)	to 77434.78)	
85-89 years	Male	47793.22 (35675.33 to 63073.88)	943.77 (704.48 to 1245.52)	242399.71 (205860.48 to 285283.19)	1405 (1193.21 to 1653.56)	17665.97 (15802.54 to 19632.44)	348.85 (312.05 to 387.68)	75453.12 (63886.51 to 83313.17)	437.34 (370.3 to 482.9)
90-94 years	Female	24595.07 (16207.25 to 35628.51)	812.73 (535.56 to 1177.32)	97149.1 (76112.63 to 123828.89)	805.49 (631.07 to 1026.7)	10424.47 (8562.63 to 11600.47)	344.47 (282.95 to 383.33)	44079.11 (34268.97 to 49984)	365.47 (284.13 to 414.43)
90-94 years	Male	16351.99 (10614.99 to 23891.95)	1298.85 (843.15 to 1897.75)	108420.64 (86315.79 to 135673.75)	1860.18 (1480.93 to 2327.76)	6008.88 (5238.26 to 6637.96)	477.29 (416.08 to 527.26)	36991.2 (31164.25 to 40851.29)	634.66 (534.69 to 700.89)
95+ years	Female	8714.3 (4885.4 to 13819.25)	1149.86 (644.63 to 1823.47)	39351.53 (27722.5 to 53364.97)	999.21 (703.93 to 1355.04)	3737.78 (2876.14 to 4263.4)	493.21 (379.51 to 562.56)	24041.05 (17456.84 to 27675.09)	610.45 (443.26 to 702.72)
95+ years	Male	4960.53 (2829.86 to 7873.29)	1906.2 (1087.44 to 3025.5)	32877.35 (23957.42 to 43493.43)	2174.37 (1584.44 to 2876.47)	1602.49 (1303.22 to 1797.79)	615.79 (500.79 to 690.84)	12250.22 (9660.61 to 13743.8)	810.18 (638.91 to 908.96)

Total skin cancer includes malignant skin melanoma, non-melanoma skin cancer (basal-cell carcinoma) and non-melanoma skin cancer (squamous-cell carcinoma). DALYs, disability-adjusted life-years; UI, uncertainty interval.

TABLE S16 The number of cases and age-standardized rate of incidence and DALYs of malignant skin melanoma by age group and sex in 1990 and 2021, with age-standardized rates and 95% uncertainty intervals.

Age group	Sex	Incidence				DALYs			
		Number of cases (95% UI), 1990	Age-standardized rate (95% UI), 1990	Number of cases (95% UI), 2021	Age-standardized rate (95% UI), 2021	Number of cases (95% UI), 1990	Age-standardized rate (95% UI), 1990	Number of cases (95% UI), 2021	Age-standardized rate (95% UI), 2021
<5 years	Female	0 (0 to 0)	0 (0 to 0)	0 (0 to 0)	0 (0 to 0)	0 (0 to 0)	0 (0 to 0)	0 (0 to 0)	0 (0 to 0)
<5 years	Male	0 (0 to 0)	0 (0 to 0)	0 (0 to 0)	0 (0 to 0)	0 (0 to 0)	0 (0 to 0)	0 (0 to 0)	0 (0 to 0)
5-9 years	Female	0 (0 to 0)	0 (0 to 0)	0 (0 to 0)	0 (0 to 0)	0 (0 to 0)	0 (0 to 0)	0 (0 to 0)	0 (0 to 0)
5-9 years	Male	0 (0 to 0)	0 (0 to 0)	0 (0 to 0)	0 (0 to 0)	0 (0 to 0)	0 (0 to 0)	0 (0 to 0)	0 (0 to 0)
10-14 years	Female	0 (0 to 0)	0 (0 to 0)	0 (0 to 0)	0 (0 to 0)	0 (0 to 0)	0 (0 to 0)	0 (0 to 0)	0 (0 to 0)
10-14 years	Male	0 (0 to 0)	0 (0 to 0)	0 (0 to 0)	0 (0 to 0)	0 (0 to 0)	0 (0 to 0)	0 (0 to 0)	0 (0 to 0)
15-19 years	Female	480.24 (426.01 to 524.03)	0.19 (0.17 to 0.21)	684.9 (543.53 to 820.37)	0.23 (0.18 to 0.27)	5789.37 (4127.06 to 6996.05)	2.27 (1.62 to 2.74)	6438.87 (4018.29 to 9483.42)	2.12 (1.32 to 3.12)
15-19 years	Male	314.02 (276.35 to 340.43)	0.12 (0.1 to 0.13)	403.91 (338.8 to 461.63)	0.13 (0.11 to 0.14)	5692.1 (4303.94 to 6627.45)	2.16 (1.63 to 2.51)	5137.85 (3574.23 to 6572.98)	1.6 (1.12 to 2.05)
20-24 years	Female	1231.04 (1120.52 to 1326.14)	0.5 (0.46 to 0.54)	1785.15 (1492.83 to 2060.75)	0.61 (0.51 to 0.7)	12083.4 (9188.62 to 14289.19)	4.95 (3.76 to 5.85)	14661.49 (9221.56 to 20557.14)	4.99 (3.14 to 7)
20-24	Male	796.38 (734.06 to	0.32 (0.3 to 0.34)	1064.13 (945.44 to	0.35 (0.31 to	11132.43 (9310.73 to	4.49 (3.76 to 4.96)	10675.68 (8139.3 to	3.52 (2.68 to

years		848.42)		1184.43)	0.39)	12296.14)		13091.97)	4.31)
25-29 years	Female	3086.07 (2896.92 to 3284.52)	1.4 (1.32 to 1.49)	3826.41 (3363.19 to 4239.69)	1.31 (1.16 to 1.46)	20907.15 (17424.97 to 23584.66)	9.5 (7.92 to 10.72)	21407.19 (15541.58 to 28407.81)	7.36 (5.34 to 9.76)
25-29 years	Male	1948.52 (1841.98 to 2059.38)	0.88 (0.83 to 0.93)	2515.12 (2245.3 to 2740.36)	0.85 (0.76 to 0.92)	20095.89 (17881.68 to 21932.79)	9.03 (8.04 to 9.86)	18901.91 (14973.36 to 22596.07)	6.36 (5.04 to 7.6)
30-34 years	Female	4298.57 (4062.24 to 4546.2)	2.26 (2.14 to 2.39)	5940.78 (5291.42 to 6477.72)	1.99 (1.77 to 2.17)	30472.43 (26123.89 to 33789.11)	16.03 (13.74 to 17.77)	33362.49 (24898.67 to 41564.34)	11.16 (8.33 to 13.9)
30-34 years	Male	3204.3 (3026.01 to 3377.57)	1.64 (1.55 to 1.73)	4296.73 (3787.94 to 4731.44)	1.41 (1.24 to 1.55)	35805.74 (31721.65 to 38769.5)	18.33 (16.24 to 19.85)	36662.49 (28865.05 to 43038.24)	12 (9.45 to 14.09)
35-39 years	Female	4981.37 (4710.41 to 5242.14)	2.87 (2.72 to 3.02)	7302.46 (6474.35 to 8083.35)	2.63 (2.33 to 2.91)	39410.24 (34225.34 to 43767.08)	22.72 (19.73 to 25.23)	43426.24 (33444.8 to 54355.75)	15.63 (12.04 to 19.57)
35-39 years	Male	4184.26 (3956.56 to 4403.11)	2.34 (2.21 to 2.46)	5530.08 (4877.96 to 6113.74)	1.95 (1.72 to 2.16)	49856.03 (43731.42 to 54148.72)	27.89 (24.46 to 30.29)	51114.35 (40639.93 to 60728.37)	18.06 (14.36 to 21.45)
40-44 years	Female	5484.1 (5180.86 to 5759.54)	3.91 (3.69 to 4.11)	8362.72 (7508.26 to 9153.27)	3.37 (3.03 to 3.69)	43868.04 (38713.11 to 48014.02)	31.28 (27.61 to 34.24)	50486.89 (40763.83 to 60673.36)	20.35 (16.43 to 24.46)
40-44 years	Male	4980.07 (4707.11 to 5236.45)	3.41 (3.22 to 3.58)	7010.57 (6191.14 to 7664.19)	2.78 (2.46 to 3.04)	58835.34 (51537.61 to 63259.45)	40.23 (35.24 to 43.25)	65257.28 (52497.72 to 76791.51)	25.88 (20.82 to 30.45)
45-49 years	Female	4992.67 (4749.31 to 5244.55)	4.39 (4.17 to 4.61)	9615.75 (8721.48 to 10486.04)	4.08 (3.7 to 4.45)	41014.91 (36200.59 to 44978.71)	36.04 (31.81 to 39.52)	56752.84 (46711.42 to 68739.82)	24.08 (19.82 to 29.17)
45-49 years	Male	4810.97 (4580.46 to 5053.82)	4.06 (3.87 to 4.27)	8285.04 (7437.82 to 9035.04)	3.48 (3.13 to 3.8)	53437.4 (47497.84 to 57523.23)	45.13 (40.12 to 48.59)	70401.64 (58597.77 to 82175.24)	29.6 (24.64 to 34.55)
50-54 years	Female	5110 (4812.07 to 5397.91)	4.87 (4.59 to 5.14)	11318.26 (10210.91 to 12362.1)	5.08 (4.58 to 5.54)	45654.71 (40463.69 to 50701.78)	43.52 (38.57 to 48.33)	68585.01 (55559.75 to 80031.16)	30.76 (24.92 to 35.9)
50-54 years	Male	5500.75 (5217.36 to 5808.05)	5.11 (4.85 to 5.4)	11443.56 (10363.29 to 12401.24)	5.16 (4.67 to 5.59)	63674.66 (56572.18 to 69141.68)	59.15 (52.55 to 64.23)	92378.93 (77745.66 to 104823.11)	41.62 (35.02 to 47.22)
55-59	Female	5189.57 (4877.66 to	5.62 (5.28 to 5.92)	13072.54 (11955.75	6.5 (5.95 to	45196.18 (39757.54 to	48.96 (43.07 to	76125.97 (62572.66	37.87 (31.13 to

years		5461.67)		to 14137.27)	7.03)	50435.91)	54.64)	to 88481.22)	44.02)
55-59 years	Male	6346.11 (5970.8 to 6676.05)	6.83 (6.43 to 7.19)	15217.06 (13882.4 to 16434.51)	7.81 (7.13 to 8.44)	66231.38 (58327.59 to 72732.79)	71.3 (62.79 to 78.3)	107499.99 (91505.74 to 122639.11)	55.21 (46.99 to 62.98)
60-64 years	Female	5602.85 (5274.12 to 5926.59)	6.83 (6.43 to 7.22)	13908.01 (12813.49 to 14987.06)	8.45 (7.79 to 9.11)	45484.88 (41098.49 to 50360.12)	55.43 (50.08 to 61.37)	75641.09 (64440.71 to 86261.1)	45.98 (39.17 to 52.43)
60-64 years	Male	7063.84 (6730.61 to 7383.23)	8.99 (8.57 to 9.4)	17528.54 (16426.63 to 18716.31)	11.27 (10.56 to 12.03)	64393.35 (58857.93 to 68626.54)	81.98 (74.93 to 87.37)	106715.01 (95351.63 to 117288.17)	68.61 (61.3 to 75.41)
65-69 years	Female	6029.39 (5676.35 to 6337.37)	9.1 (8.56 to 9.56)	15066.1 (13783.83 to 16236.58)	10.46 (9.57 to 11.27)	42884.87 (39356.39 to 46608.1)	64.7 (59.38 to 70.32)	75277.85 (64415.23 to 85197.94)	52.27 (44.73 to 59.16)
65-69 years	Male	7190.86 (6865.78 to 7536.93)	12.54 (11.98 to 13.15)	20776.1 (19455.93 to 22177.19)	15.76 (14.76 to 16.82)	55149.98 (50834.59 to 59393.8)	96.2 (88.67 to 103.6)	108393.1 (96745.77 to 119487.15)	82.22 (73.38 to 90.64)
70-74 years	Female	5059.74 (4714.72 to 5370.83)	10.76 (10.02 to 11.42)	15347.04 (13935.34 to 16672.69)	14.02 (12.73 to 15.23)	32166.51 (29220.18 to 35409.31)	68.38 (62.11 to 75.27)	68544.68 (59841.94 to 77953.12)	62.63 (54.68 to 71.22)
70-74 years	Male	5949.52 (5646.7 to 6253.07)	15.82 (15.01 to 16.62)	22238.93 (20753.81 to 23766.82)	23.07 (21.53 to 24.66)	38547.65 (35581 to 41134.71)	102.47 (94.58 to 109.35)	98813.51 (89897.03 to 107274.26)	102.51 (93.26 to 111.29)
75-79 years	Female	4833.07 (4428.21 to 5127.19)	13.31 (12.19 to 14.12)	12159.41 (10547.43 to 13327.4)	16.87 (14.63 to 18.49)	29858.2 (27193.83 to 32099.1)	82.2 (74.87 to 88.37)	51843.77 (44868.18 to 58547.78)	71.91 (62.23 to 81.21)
75-79 years	Male	4824.04 (4535.54 to 5076.29)	19.12 (17.97 to 20.12)	17544.16 (16178.16 to 18852.15)	29.34 (27.06 to 31.53)	29233.29 (27168.46 to 31018.72)	115.85 (107.67 to 122.93)	71266.7 (65003.32 to 77945.08)	119.2 (108.73 to 130.37)
80-84 years	Female	3498.49 (3045.73 to 3800.75)	15.84 (13.79 to 17.2)	10674.93 (8717.22 to 11984.84)	20.96 (17.12 to 23.53)	19796.52 (17491.3 to 21557.16)	89.61 (79.17 to 97.58)	43850.42 (37085.12 to 49894.29)	86.1 (72.81 to 97.96)
80-84 years	Male	2799.28 (2591.88 to 2971.95)	21.07 (19.51 to 22.37)	14105.4 (12161.68 to 15405.49)	38.48 (33.18 to 42.03)	15681.01 (14478.14 to 16752.76)	118.05 (108.99 to 126.12)	52839.31 (46787.01 to 57641.2)	144.17 (127.65 to 157.27)
85-89	Female	1963.39 (1593.16 to	19.54 (15.86 to	7844.3 (5941 to	27.55 (20.87 to	9880.53 (8336.19 to	98.34 (82.97 to	28205.87 (22071.13	99.08 (77.53 to

years		2166.13)	21.56)	8963.72)	31.49)	10851.59)	108.01)	to 32383.34)	113.75)
85-89 years	Male	1365.53 (1211.43 to 1465.45)	26.97 (23.92 to 28.94)	9280.03 (7828.29 to 10233.41)	53.79 (45.37 to 59.31)	6524.7 (5870.52 to 7023.76)	128.84 (115.93 to 138.7)	30248.04 (25797.59 to 33166.74)	175.32 (149.53 to 192.24)
90-94 years	Female	613.77 (480.8 to 688.03)	20.28 (15.89 to 22.74)	3475.95 (2593.59 to 3988.37)	28.82 (21.5 to 33.07)	3604.04 (2878.57 to 4008.07)	119.09 (95.12 to 132.44)	15300.44 (11607.2 to 17398.17)	126.86 (96.24 to 144.25)
90-94 years	Male	345.13 (291.4 to 374.73)	27.41 (23.15 to 29.77)	3292.42 (2647.16 to 3672.11)	56.49 (45.42 to 63)	1931.13 (1669.77 to 2086.23)	153.39 (132.63 to 165.71)	12781.2 (10587.58 to 14111.8)	219.29 (181.65 to 242.12)
95+ years	Female	172.38 (128.79 to 198.04)	22.75 (16.99 to 26.13)	1404.04 (995.13 to 1644.85)	35.65 (25.27 to 41.77)	1067.63 (804.56 to 1222.84)	140.88 (106.16 to 161.35)	6520.81 (4655.41 to 7532.93)	165.58 (118.21 to 191.28)
95+ years	Male	69.57 (54.51 to 77.98)	26.73 (20.95 to 29.96)	784.09 (607.55 to 893.95)	51.86 (40.18 to 59.12)	415.83 (332.68 to 461.18)	159.79 (127.84 to 177.22)	3317.4 (2584.48 to 3725.62)	219.4 (170.93 to 246.4)

DALYs, disability-adjusted life-years; UI, uncertainty interval.

TABLE S17 The number of cases and age-standardized rate of incidence and DALYs of non-melanoma skin cancer (basal-cell carcinoma) by age group and sex in 1990 and 2021, with age-standardized rates and 95% uncertainty intervals.

Age group	Sex	Incidence				DALYs			
		Number of cases (95% UI), 1990	Age-standardized rate (95% UI), 1990	Number of cases (95% UI), 2021	Age-standardized rate (95% UI), 2021	Number of cases (95% UI), 1990	Age-standardized rate (95% UI), 1990	Number of cases (95% UI), 2021	Age-standardized rate (95% UI), 2021
<5 years	Female	0 (0 to 0)	0 (0 to 0)	0 (0 to 0)	0 (0 to 0)	0 (0 to 0)	0 (0 to 0)	0 (0 to 0)	0 (0 to 0)
<5 years	Male	0 (0 to 0)	0 (0 to 0)	0 (0 to 0)	0 (0 to 0)	0 (0 to 0)	0 (0 to 0)	0 (0 to 0)	0 (0 to 0)

5-9 years	Female	0 (0 to 0)	0 (0 to 0)	0 (0 to 0)	0 (0 to 0)	0 (0 to 0)	0 (0 to 0)	0 (0 to 0)	0 (0 to 0)
5-9 years	Male	0 (0 to 0)	0 (0 to 0)	0 (0 to 0)	0 (0 to 0)	0 (0 to 0)	0 (0 to 0)	0 (0 to 0)	0 (0 to 0)
10-14 years	Female	0 (0 to 0)	0 (0 to 0)	0 (0 to 0)	0 (0 to 0)	0 (0 to 0)	0 (0 to 0)	0 (0 to 0)	0 (0 to 0)
10-14 years	Male	0 (0 to 0)	0 (0 to 0)	0 (0 to 0)	0 (0 to 0)	0 (0 to 0)	0 (0 to 0)	0 (0 to 0)	0 (0 to 0)
15-19 years	Female	0 (0 to 0)	0 (0 to 0)	0 (0 to 0)	0 (0 to 0)	0 (0 to 0)	0 (0 to 0)	0 (0 to 0)	0 (0 to 0)
15-19 years	Male	0 (0 to 0)	0 (0 to 0)	0 (0 to 0)	0 (0 to 0)	0 (0 to 0)	0 (0 to 0)	0 (0 to 0)	0 (0 to 0)
20-24 years	Female	3840.61 (2279.37 to 5636.45)	1.57 (0.93 to 2.31)	5361.29 (3543.12 to 7706.44)	1.83 (1.21 to 2.62)	2.61 (1.02 to 5.91)	0 (0 to 0)	3.43 (1.38 to 7.62)	0 (0 to 0)
20-24 years	Male	3064.17 (1788.94 to 4751)	1.24 (0.72 to 1.92)	4459.77 (2765.51 to 6649.09)	1.47 (0.91 to 2.19)	1.8 (0.67 to 4.15)	0 (0 to 0)	2.55 (0.97 to 5.73)	0 (0 to 0)
25-29 years	Female	7043.25 (4058.8 to 10978.89)	3.2 (1.84 to 4.99)	11180.74 (7141.86 to 16314.39)	3.84 (2.45 to 5.61)	4.86 (1.84 to 10.3)	0 (0 to 0)	7.42 (2.95 to 15.83)	0 (0 to 0.01)
25-29 years	Male	5191.37 (2907.22 to 8312.7)	2.33 (1.31 to 3.74)	8133.32 (4816.12 to 12490.62)	2.74 (1.62 to 4.2)	3.09 (1.16 to 6.67)	0 (0 to 0)	4.81 (1.84 to 10.2)	0 (0 to 0)
30-34 years	Female	12481.03 (8882.44 to 17848.94)	6.57 (4.67 to 9.39)	25393.29 (19400.91 to 33699.36)	8.49 (6.49 to 11.27)	8.09 (2.97 to 17.09)	0 (0 to 0.01)	16.04 (6.09 to 32.6)	0.01 (0 to 0.01)
30-34 years	Male	9118.56 (6344.65 to 12971.04)	4.67 (3.25 to 6.64)	17259.2 (12737.27 to 23839.07)	5.65 (4.17 to 7.8)	5.21 (1.89 to 11.16)	0 (0 to 0.01)	9.85 (3.65 to 20.43)	0 (0 to 0.01)
35-39 years	Female	19116.51 (11414.44 to 28524.32)	11.02 (6.58 to 16.44)	42847.27 (30273.46 to 58550.34)	15.42 (10.9 to 21.08)	11.82 (4.21 to 23.29)	0.01 (0 to 0.01)	25.34 (9.51 to 48.38)	0.01 (0 to 0.02)

35-39 years	Male	14134.68 (8336.84 to 21613.57)	7.91 (4.66 to 12.09)	28483.65 (19946.91 to 40219.99)	10.06 (7.05 to 14.21)	8.01 (2.82 to 16.07)	0 (0 to 0.01)	15.79 (5.84 to 30.92)	0.01 (0 to 0.01)
40-44 years	Female	26344.73 (18844.42 to 35493.69)	18.79 (13.44 to 25.31)	65576.17 (51856.34 to 80967.48)	26.43 (20.9 to 32.64)	15.23 (6.39 to 30.22)	0.01 (0 to 0.02)	35.51 (15.08 to 68.71)	0.01 (0.01 to 0.03)
40-44 years	Male	21707.75 (15651.71 to 29000.49)	14.84 (10.7 to 19.83)	50005.24 (38922.37 to 62731.31)	19.83 (15.44 to 24.88)	11.63 (4.91 to 22.99)	0.01 (0 to 0.02)	25.37 (10.86 to 50.9)	0.01 (0 to 0.02)
45-49 years	Female	30714.14 (18844.84 to 45360.04)	26.99 (16.56 to 39.86)	98043.77 (70165.25 to 132843.71)	41.61 (29.78 to 56.38)	16.49 (6.89 to 37.42)	0.01 (0.01 to 0.03)	50.52 (22.25 to 107.69)	0.02 (0.01 to 0.05)
45-49 years	Male	27909.78 (17384.54 to 40814.58)	23.57 (14.68 to 34.47)	83247.65 (58422.08 to 114191.07)	35 (24.56 to 48.01)	14.3 (6.12 to 32.08)	0.01 (0.01 to 0.03)	41.12 (18.01 to 88.85)	0.02 (0.01 to 0.04)
50-54 years	Female	37140.45 (26645.31 to 51439.64)	35.4 (25.4 to 49.03)	140853.47 (111514.88 to 179608.76)	63.18 (50.02 to 80.56)	19.51 (8.72 to 43.58)	0.02 (0.01 to 0.04)	70.57 (32.3 to 149.18)	0.03 (0.01 to 0.07)
50-54 years	Male	37288.55 (27026.52 to 50974.33)	34.64 (25.1 to 47.35)	141365.38 (110641.52 to 175946.67)	63.68 (49.84 to 79.26)	18.44 (8.44 to 39.2)	0.02 (0.01 to 0.04)	66.67 (30.35 to 135.24)	0.03 (0.01 to 0.06)
55-59 years	Female	44717.18 (28704.17 to 64969.52)	48.44 (31.09 to 70.38)	180703.18 (131535.98 to 235610.99)	89.9 (65.44 to 117.22)	23.22 (10.14 to 47.55)	0.03 (0.01 to 0.05)	87.22 (38.34 to 175.03)	0.04 (0.02 to 0.09)
55-59 years	Male	48345.78 (31199.94 to 70706.08)	52.05 (33.59 to 76.12)	209652.92 (151533.2 to 272475.11)	107.67 (77.82 to 139.93)	23.24 (10.06 to 47.45)	0.03 (0.01 to 0.05)	95.2 (40.76 to 189.65)	0.05 (0.02 to 0.1)
60-64 years	Female	62080.32 (44827.76 to 85675.57)	75.65 (54.63 to 104.4)	220410.17 (168860.76 to 276319.61)	133.98 (102.64 to 167.96)	31.45 (12.77 to 63.16)	0.04 (0.02 to 0.08)	102.43 (44.56 to 195.56)	0.06 (0.03 to 0.12)
60-64	Male	71801.11 (54072.06	91.41 (68.84 to	301608.2	193.91 (155.42 to	33.18 (14.22 to	0.04 (0.02 to 0.08)	130.34 (59.3 to	0.08 (0.04 to

years		to 96572.6)	122.95)	(241732.63 to 368707.39)	237.05)	64.16)		248.24)	0.16)
65-69 years	Female	79145.98 (60835.91 to 97420.72)	119.41 (91.79 to 146.99)	270341.54 (229962.42 to 312898.87)	187.73 (159.69 to 217.28)	39.84 (16.76 to 75.58)	0.06 (0.03 to 0.11)	124.45 (57.73 to 244.28)	0.09 (0.04 to 0.17)
65-69 years	Male	93893.53 (73988.68 to 115468.3)	163.77 (129.05 to 201.41)	406732.1 (347380.46 to 464751.09)	308.52 (263.5 to 352.53)	42.57 (18 to 79.74)	0.07 (0.03 to 0.14)	174.07 (81.08 to 329.05)	0.13 (0.06 to 0.25)
70-74 years	Female	78360.62 (59644.54 to 102326.43)	166.57 (126.79 to 217.52)	279986.79 (234238.78 to 329380.99)	255.82 (214.02 to 300.95)	39.15 (17.01 to 75.82)	0.08 (0.04 to 0.16)	126.85 (57.89 to 247.59)	0.12 (0.05 to 0.23)
70-74 years	Male	90971.06 (68759.28 to 117391.59)	241.83 (182.78 to 312.06)	439063.29 (372916.62 to 513749.49)	455.5 (386.88 to 532.98)	40.79 (17.55 to 79.45)	0.11 (0.05 to 0.21)	183.73 (83.3 to 349.55)	0.19 (0.09 to 0.36)
75-79 years	Female	80132.11 (58014.21 to 109143.51)	220.61 (159.72 to 300.48)	227360.18 (185512.71 to 272854.84)	315.35 (257.31 to 378.45)	40.57 (16.71 to 79.29)	0.11 (0.05 to 0.22)	102.17 (44.42 to 204.89)	0.14 (0.06 to 0.28)
75-79 years	Male	83453.37 (60707.76 to 110356.66)	330.73 (240.59 to 437.35)	352152.65 (291519.95 to 420577.32)	589.01 (487.6 to 703.46)	37.91 (16.5 to 74.21)	0.15 (0.07 to 0.29)	144.64 (64.94 to 278.54)	0.24 (0.11 to 0.47)
80-84 years	Female	61378.51 (46867.34 to 79460.21)	277.83 (212.14 to 359.67)	173056.1 (143401.69 to 203321.87)	339.78 (281.56 to 399.21)	30.73 (13.1 to 61.21)	0.14 (0.06 to 0.28)	78.85 (35.37 to 159.16)	0.15 (0.07 to 0.31)
80-84 years	Male	55778.56 (43295.57 to 69583.51)	419.91 (325.94 to 523.84)	248467.46 (209659.46 to 287327.33)	677.91 (572.03 to 783.94)	25.34 (11.04 to 49.96)	0.19 (0.08 to 0.38)	102.81 (45.25 to 204.01)	0.28 (0.12 to 0.56)

85-89 years	Female	34988.19 (27042.51 to 44876.81)	348.24 (269.16 to 446.67)	108853.7 (91485.26 to 131769.16)	382.36 (321.35 to 462.85)	16.49 (7.21 to 31.57)	0.16 (0.07 to 0.31)	48.27 (21.98 to 91.89)	0.17 (0.08 to 0.32)
85-89 years	Male	26926.91 (20872.46 to 34797.92)	531.73 (412.17 to 687.15)	139637.3 (119064.58 to 166045.44)	809.37 (690.12 to 962.43)	11.89 (5.22 to 22.88)	0.23 (0.1 to 0.45)	56.91 (27.03 to 110.14)	0.33 (0.16 to 0.64)
90-94 years	Female	13737.99 (9394.57 to 19521.87)	453.96 (310.44 to 645.09)	56355.47 (44042.91 to 73685.85)	467.26 (365.17 to 610.95)	6.1 (2.57 to 11.68)	0.2 (0.08 to 0.39)	24.2 (10.56 to 46.49)	0.2 (0.09 to 0.39)
90-94 years	Male	8746.49 (5823.13 to 12683.77)	694.74 (462.53 to 1007.48)	60527.26 (48391.97 to 76858.23)	1038.47 (830.26 to 1318.66)	3.76 (1.61 to 7.2)	0.3 (0.13 to 0.57)	24.34 (10.73 to 45.31)	0.42 (0.18 to 0.78)
95+ years	Female	4453.13 (2461.05 to 7127.98)	587.6 (324.74 to 940.55)	21775.41 (14800.35 to 30711.48)	552.92 (375.81 to 779.82)	1.88 (0.77 to 3.81)	0.25 (0.1 to 0.5)	9.14 (3.76 to 17.37)	0.23 (0.1 to 0.44)
95+ years	Male	2526 (1412.22 to 4026.74)	970.68 (542.68 to 1547.37)	18045.08 (12887.84 to 24492.69)	1193.42 (852.35 to 1619.84)	1.06 (0.41 to 2.15)	0.41 (0.16 to 0.83)	7.25 (3.03 to 14.22)	0.48 (0.2 to 0.94)

DALYs, disability-adjusted life-years; UI, uncertainty interval.

TABLE S18 The number of cases and age-standardized rate of incidence and DALYs of non-melanoma skin cancer (squamous-cell carcinoma) by age group and sex in 1990 and 2021, with age-standardized rates and 95% uncertainty intervals.

Age group	Sex	Incidence				DALYs			
		Number of cases (95% UI), 1990	Age-standardized rate (95% UI), 1990	Number of cases (95% UI), 2021	Age-standardized rate (95% UI), 2021	Number of cases (95% UI), 1990	Age-standardized rate (95% UI), 1990	Number of cases (95% UI), 2021	Age-standardized rate (95% UI), 2021
<5 years	Female	0 (0 to 0)	0 (0 to 0)	0 (0 to 0)	0 (0 to 0)	0 (0 to 0)	0 (0 to 0)	0 (0 to 0)	0 (0 to 0)
<5 years	Male	0 (0 to 0)	0 (0 to 0)	0 (0 to 0)	0 (0 to 0)	0 (0 to 0)	0 (0 to 0)	0 (0 to 0)	0 (0 to 0)

5-9 years	Female	0 (0 to 0)	0 (0 to 0)	0 (0 to 0)	0 (0 to 0)	0 (0 to 0)	0 (0 to 0)	0 (0 to 0)	0 (0 to 0)
5-9 years	Male	0 (0 to 0)	0 (0 to 0)	0 (0 to 0)	0 (0 to 0)	0 (0 to 0)	0 (0 to 0)	0 (0 to 0)	0 (0 to 0)
10-14 years	Female	0 (0 to 0)	0 (0 to 0)	0 (0 to 0)	0 (0 to 0)	0 (0 to 0)	0 (0 to 0)	0 (0 to 0)	0 (0 to 0)
10-14 years	Male	0 (0 to 0)	0 (0 to 0)	0 (0 to 0)	0 (0 to 0)	0 (0 to 0)	0 (0 to 0)	0 (0 to 0)	0 (0 to 0)
15-19 years	Female	0 (0 to 0)	0 (0 to 0)	0 (0 to 0)	0 (0 to 0)	0 (0 to 0)	0 (0 to 0)	0 (0 to 0)	0 (0 to 0)
15-19 years	Male	0 (0 to 0)	0 (0 to 0)	0 (0 to 0)	0 (0 to 0)	0 (0 to 0)	0 (0 to 0)	0 (0 to 0)	0 (0 to 0)
20-24 years	Female	684.16 (374.09 to 1109.3)	0.28 (0.15 to 0.45)	546.54 (310.14 to 874.97)	0.19 (0.11 to 0.3)	4866.74 (4162.26 to 6039.18)	1.99 (1.7 to 2.47)	4817.03 (4200.21 to 5458.82)	1.64 (1.43 to 1.86)
20-24 years	Male	858.19 (460.79 to 1382.51)	0.35 (0.19 to 0.56)	794.23 (418.22 to 1278.47)	0.26 (0.14 to 0.42)	5542.85 (4396.04 to 6747.93)	2.24 (1.77 to 2.72)	5546.86 (3998.73 to 6368.07)	1.83 (1.32 to 2.1)
25-29 years	Female	932.95 (484.04 to 1550.54)	0.42 (0.22 to 0.7)	848.29 (517.76 to 1320.57)	0.29 (0.18 to 0.45)	6094.22 (5176.32 to 7527.54)	2.77 (2.35 to 3.42)	6961.46 (5872.86 to 7965.22)	2.39 (2.02 to 2.74)
25-29 years	Male	1097.54 (574.76 to 1846.71)	0.49 (0.26 to 0.83)	1133.17 (625.53 to 1913.14)	0.38 (0.21 to 0.64)	7528.18 (6359.05 to 9180.61)	3.38 (2.86 to 4.13)	8599.26 (6380 to 9929.95)	2.89 (2.15 to 3.34)
30-34 years	Female	1523.91 (943.14 to 2330.55)	0.8 (0.5 to 1.23)	1957.41 (1446.55 to 2681.47)	0.65 (0.48 to 0.9)	7043.6 (6077.05 to 8447.19)	3.71 (3.2 to 4.44)	10025.6 (8696.13 to 11563.61)	3.35 (2.91 to 3.87)
30-34 years	Male	1688.74 (1056.81 to 2608.68)	0.86 (0.54 to 1.34)	2417.67 (1683.03 to 3434.78)	0.79 (0.55 to 1.12)	11352.74 (9425.15 to 13002.78)	5.81 (4.83 to 6.66)	17770.87 (10473.45 to 21671.18)	5.82 (3.43 to 7.09)
35-39 years	Female	2283.53 (1171.63 to 4182.51)	1.32 (0.68 to 2.41)	3627.3 (2488.93 to 5509.39)	1.31 (0.9 to 1.98)	10014.61 (8813.02 to 12036.35)	5.77 (5.08 to 6.94)	14313.53 (12600.8 to 16042.03)	5.15 (4.54 to 5.77)
35-39 years	Male	2476.31 (1236.46 to 4585.82)	1.39 (0.69 to 2.56)	4205.41 (2819.12 to 6564.06)	1.49 (1 to 2.32)	14411.9 (12421.86 to 16536.62)	8.06 (6.95 to 9.25)	18084.25 (13284.14 to 21047.23)	6.39 (4.69 to 7.44)
40-44 years	Female	4033.15 (2585.46 to 5972.02)	2.88 (1.84 to 4.26)	8550.06 (6532.72 to 11186.13)	3.45 (2.63 to 4.51)	11586.8 (10242.5 to 13913.98)	8.26 (7.3 to 9.92)	18211.47 (16175.25 to 20363.54)	7.34 (6.52 to 8.21)
40-44 years	Male	4396.71 (2744.91 to 6596.54)	3.01 (1.88 to 4.51)	9272.28 (6950.98 to 12307.02)	3.68 (2.76 to 4.88)	18584.64 (15362.05 to 21477.55)	12.71 (10.5 to 14.68)	26469.04 (19544.66 to 31324.29)	10.5 (7.75 to 12.42)

45-49 years	Female	5728.11 (3420.11 to 9091.77)	5.03 (3.01 to 7.99)	16839.13 (12048.55 to 23937.59)	7.15 (5.11 to 10.16)	12788.54 (11416.07 to 14708.74)	11.24 (10.03 to 12.93)	24562.52 (21260.88 to 28219.14)	10.42 (9.02 to 11.98)
45-49 years	Male	6240.87 (3497.86 to 10208.22)	5.27 (2.95 to 8.62)	17625.51 (12534.6 to 25111.53)	7.41 (5.27 to 10.56)	18425.23 (15730.1 to 20891.72)	15.56 (13.29 to 17.65)	31736.03 (24283.8 to 36987.46)	13.34 (10.21 to 15.55)
50-54 years	Female	7154.16 (4669.62 to 10448.33)	6.82 (4.45 to 9.96)	28517.36 (22358.48 to 36441.13)	12.79 (10.03 to 16.35)	16866.49 (15181.34 to 19528.75)	16.08 (14.47 to 18.61)	34512.3 (29387.51 to 39664.5)	15.48 (13.18 to 17.79)
50-54 years	Male	9816.56 (6309.79 to 14691.39)	9.12 (5.86 to 13.65)	36897.36 (28347.62 to 47586.46)	16.62 (12.77 to 21.44)	25650.22 (21913.08 to 28831.64)	23.83 (20.35 to 26.78)	46607.72 (35993.4 to 53844.73)	21 (16.21 to 24.26)
55-59 years	Female	8640.71 (4115.8 to 14772.34)	9.36 (4.46 to 16)	42178.02 (30623.87 to 57764.67)	20.98 (15.24 to 28.74)	19504.53 (17486.83 to 23048.03)	21.13 (18.94 to 24.97)	44058.15 (37486.67 to 50876.01)	21.92 (18.65 to 25.31)
55-59 years	Male	14900.22 (7353.83 to 25338.77)	16.04 (7.92 to 27.28)	64464.23 (44961.05 to 89837.22)	33.11 (23.09 to 46.14)	31136.51 (26920.15 to 34667.53)	33.52 (28.98 to 37.32)	59862.27 (50095.05 to 67624.78)	30.74 (25.73 to 34.73)
60-64 years	Female	15198.51 (9802.98 to 22112.5)	18.52 (11.95 to 26.95)	71081.03 (58114.57 to 87275.7)	43.21 (35.33 to 53.05)	22419.16 (20155.76 to 25233.23)	27.32 (24.56 to 30.75)	45538.96 (39635.9 to 51971.76)	27.68 (24.09 to 31.59)
60-64 years	Male	28771.26 (19651.68 to 39633.78)	36.63 (25.02 to 50.46)	125528.11 (103997.12 to 153037.26)	80.71 (66.86 to 98.39)	44905.72 (38591.16 to 51079.2)	57.17 (49.13 to 65.03)	86791.9 (71407.67 to 101009.81)	55.8 (45.91 to 64.94)
65-69 years	Female	24793.45 (17803.92 to 34146.55)	37.41 (26.86 to 51.52)	105136.5 (89390.77 to 122442.8)	73.01 (62.07 to 85.02)	23067.36 (20759.23 to 25864.48)	34.8 (31.32 to 39.02)	55236.94 (47718.03 to 62942.33)	38.36 (33.14 to 43.71)
65-69 years	Male	45617.43 (33443.63 to 62625.34)	79.57 (58.33 to 109.23)	198630.07 (168473.88 to 231572.59)	150.67 (127.79 to 175.66)	43298.1 (36865.65 to 50501.57)	75.52 (64.3 to 88.09)	100141.11 (81787.28 to 118064.36)	75.96 (62.04 to 89.56)
70-74 years	Female	28433.38 (19275.11 to 40664.53)	60.44 (40.97 to 86.44)	123476.07 (102891.93 to 147757.65)	112.82 (94.01 to 135)	21713.31 (19663.55 to 24427.53)	46.16 (41.8 to 51.93)	56559.27 (49003.6 to 64055.73)	51.68 (44.77 to 58.53)
70-74 years	Male	48829.95 (33274.37	129.8 (88.45 to	233187.68	241.92 (200.55 to	35507.24 (30471.35	94.39 (81 to 105.9)	96975.2 (81355.87	100.61 (84.4 to

		to 69242.17)	184.06)	(193314.41 to 281514.89)	292.05)	to 39837.08)		to 111100.24)	115.26)
75-79 years	Female	29829.4 (18986.11 to 43856.38)	82.12 (52.27 to 120.74)	106540.59 (86777.61 to 130465.78)	147.77 (120.36 to 180.96)	22305.06 (20538.34 to 24457.62)	61.41 (56.54 to 67.33)	46774.8 (41044.29 to 52449.38)	64.88 (56.93 to 72.75)
75-79 years	Male	45221.28 (29712.55 to 65353.37)	179.22 (117.75 to 259)	193810.29 (156717.31 to 237818.28)	324.17 (262.13 to 397.78)	29896.65 (26480.39 to 33485.06)	118.48 (104.94 to 132.7)	76058.69 (64069.54 to 86334.91)	127.22 (107.16 to 144.4)
80-84 years	Female	27743.02 (20196.48 to 37774.75)	125.58 (91.42 to 170.98)	86861.95 (74170.78 to 102730.12)	170.55 (145.63 to 201.7)	19592.94 (17510.75 to 21328.67)	88.69 (79.26 to 96.54)	46585.64 (39170.3 to 51658.72)	91.47 (76.91 to 101.43)
80-84 years	Male	34092.43 (25211.16 to 45786.79)	256.65 (189.79 to 344.69)	147379.6 (127160.54 to 172370.27)	402.11 (346.94 to 470.29)	20567.99 (18341.19 to 22883.51)	154.84 (138.08 to 172.27)	63776.03 (53899.97 to 71048.62)	174 (147.06 to 193.85)
85-89 years	Female	20667.46 (14396.82 to 28756.94)	205.71 (143.29 to 286.22)	62779.92 (52825.93 to 73191.88)	220.52 (185.56 to 257.09)	14430.78 (12659.17 to 15888.73)	143.63 (126 to 158.14)	39772.64 (32054.09 to 44959.56)	139.7 (112.59 to 157.92)
85-89 years	Male	19500.77 (13591.43 to 26810.51)	385.08 (268.39 to 529.43)	93482.39 (78967.61 to 109004.34)	541.84 (457.71 to 631.81)	11129.39 (9926.8 to 12585.81)	219.77 (196.02 to 248.53)	45148.17 (38061.89 to 50036.28)	261.69 (220.61 to 290.02)
90-94 years	Female	10243.31 (6331.88 to 15418.6)	338.48 (209.23 to 509.5)	37317.67 (29476.13 to 46154.67)	309.41 (244.39 to 382.68)	6814.33 (5681.49 to 7580.72)	225.18 (187.74 to 250.5)	28754.47 (22651.22 to 32539.34)	238.41 (187.81 to 269.79)
90-94 years	Male	7260.37 (4500.46 to 10833.46)	576.7 (357.47 to 860.51)	44600.95 (35276.66 to 55143.41)	765.22 (605.24 to 946.1)	4074 (3566.87 to 4544.53)	323.6 (283.32 to 360.97)	24185.66 (20565.94 to 26694.18)	414.96 (352.85 to 457.99)
95+ years	Female	4088.78 (2295.56 to 6493.24)	539.52 (302.9 to 856.79)	16172.07 (11927.01 to 21008.64)	410.64 (302.85 to 533.45)	2668.27 (2070.82 to 3036.75)	352.08 (273.25 to 400.7)	17511.1 (12797.67 to 20124.79)	444.64 (324.96 to 511.01)
95+ years	Male	2364.96 (1363.13 to 3768.58)	908.79 (523.82 to 1448.17)	14048.17 (10462.03 to 18106.79)	929.09 (691.91 to 1197.51)	1185.6 (970.13 to 1334.46)	455.59 (372.79 to 512.8)	8925.58 (7073.1 to 10003.96)	590.3 (467.78 to 661.62)
<5 years	Female	0 (0 to 0)	0 (0 to 0)	0 (0 to 0)	0 (0 to 0)	0 (0 to 0)	0 (0 to 0)	0 (0 to 0)	0 (0 to 0)

<5 years	Male	0 (0 to 0)	0 (0 to 0)	0 (0 to 0)	0 (0 to 0)	0 (0 to 0)	0 (0 to 0)	0 (0 to 0)	0 (0 to 0)

DALYs, disability-adjusted life-years; UI, uncertainty interval.

TABLE S19 Proportions of age-standardized incidence rates of skin cancers across different age groups in 1990 and 2021.

Age group	Malignant skin melanoma		Non-melanoma skin cancer (basal-cell carcinoma)		Non-melanoma skin cancer (squamous-cell carcinoma)	
	proportion, 1990	proportion, 2021	proportion, 1990	proportion, 2021	proportion, 1990	proportion, 2021
0-19	100.00%	100.00%	0.00%	0.00%	0.00%	0.00%
20-39	21.56%	16.78%	67.97%	75.07%	10.47%	8.15%
40-59	10.85%	6.43%	72.68%	75.71%	16.47%	17.86%
60-79	4.61%	3.48%	66.70%	65.36%	28.70%	31.16%
≥80	2.49%	3.47%	55.74%	57.88%	41.78%	38.65%

TABLE S20 Proportions of age-standardized DALYs rates of skin cancers across different age groups in 1990 and 2021.

Age group	Malignant skin melanoma		Non-melanoma skin cancer (basal-cell carcinoma)		Non-melanoma skin cancer (squamous-cell carcinoma)	
	proportion, 1990	proportion, 2021	proportion, 1990	proportion, 2021	proportion, 1990	proportion, 2021
0-19	100.00%	100.00%	0.00%	0.00%	0.00%	0.00%
20-39	76.70%	72.63%	0.02%	0.03%	23.28%	27.35%
40-59	73.28%	67.57%	0.02%	0.05%	26.70%	32.38%
60-79	57.64%	53.46%	0.05%	0.09%	42.31%	46.45%
≥80	36.13%	36.31%	0.06%	0.06%	63.80%	63.63%

DALYs, disability-adjusted life-years.

TABLE S21 Comparison of deviance, AIC, and BIC for four nested APC models on Incidence and DALYs for total skin cancer and its subtypes.

Disease	Model	Incidence			DALYs		
		Deviance	AIC	BIC	Deviance	AIC	BIC
Total skin cancer	A	2162510.23	2164211.03	2164258.27	177360.49	179057.01	179104.26
	AP	302944.83	304657.63	304721.55	112541.20	114249.72	114313.64
	AC	517022.24	518767.04	518875.43	15287.99	17028.51	17136.90
	APC	24598.38	24686.38	24808.66	3366.30	3454.30	3576.58
Malignant skin melanoma	A	46002.79	47416.42	47463.66	200986.69	202622.29	202669.53
	AP	18601.47	20027.10	20091.02	83833.44	85481.04	85544.96
	AC	15089.66	16547.29	16655.68	19018.39	20697.98	20806.37
	APC	724.11	812.11	934.39	2420.37	2508.37	2630.65
basal-cell carcinoma	A	1452307.92	1453893.22	1453936.72	479.92	1213.49	1256.99
	AP	174009.72	175607.02	175666.83	74.87	820.44	880.25
	AC	350466.33	352093.63	352194.22	120.06	895.63	996.21
	APC	16807.81	16891.81	17005.99	8.41	92.41	206.58
squamous-cell carcinoma	A	755064.77	756516.11	756559.61	21187.31	22687.14	22730.64
	AP	130752.19	132215.54	132275.34	14787.04	16298.87	16358.68
	AC	191553.44	193046.79	193147.37	4241.83	5783.67	5884.25
	APC	10438.54	10522.54	10636.71	1561.39	1645.39	1759.57

Total skin cancer includes malignant skin melanoma, squamous-cell carcinoma and basal-cell carcinoma. DALYs, disability-adjusted life-years. A, age model; AP, age-period model; AC, age-cohort model; APC, age-period-cohort model.

TABLE S22 Age-standardized incidence and DALYs rates of total skin cancer and its subtypes for 21 GBD regions, by year (1990-2021).

Location name	Year	SDI	Total skin cancer		Malignant skin melanoma		Non-melanoma skin cancer (basal-cell carcinoma)		Non-melanoma skin cancer (squamous-cell carcinoma)	
			Age-standardized incidence rate	Age-standardized DALY rate	Age-standardized incidence rate	Age-standardized DALY rate	Age-standardized incidence rate	Age-standardized DALY rate	Age-standardized incidence rate	Age-standardized DALY rate
Andean Latin America	1990	0.500011489518683	16.8088429	31.9787631511608	0.924611812	20.2790445709021	15.49380243	0.00943876384842728	0.390428656	11.6902798164103
Andean Latin America	1991	0.501652202488628	16.92699371	31.4471334766286	0.909926309	19.8330585616893	15.61234188	0.00947901996450817	0.404725522	11.6045958949748
Andean Latin America	1992	0.503655860402823	17.05243823	32.056244871917	0.915912249	19.9593550727017	15.71947685	0.00952665337549636	0.417049125	12.0873631458398
Andean Latin America	1993	0.506388431143355	17.15629024	32.2192019570147	0.917805016	19.8004435355214	15.81151651	0.00952495713648762	0.426968713	12.4092334643568
Andean Latin America	1994	0.51030557441471	17.25270127	32.6593166440087	0.93401844	19.8553412094715	15.88494811	0.00959713869821046	0.433734718	12.794378295839
Andean Latin America	1995	0.514896344363455	17.31937747	32.9962825723348	0.95272761	19.7530021672789	15.93037164	0.00960458448208069	0.436278217	13.2336758205738
Andean Latin America	1996	0.519376447388139	17.34969721	32.8314113912202	0.960142855	19.4424353901097	15.95438903	0.00960792804644091	0.435165324	13.3793680730641
Andean Latin	1997	0.52403649	17.36252771	32.91042896824	0.966464196	19.15228849000	15.96409888	0.009603587662	0.431964628	13.74853689058

America		0435374		94		02		43198		68
Andean Latin America	1998	0.528443908833288	17.35329114	33.5159846644962	0.966547359	18.8748369173385	15.95945092	0.00958910873111254	0.427292863	14.6315586384266
Andean Latin America	1999	0.532743716538652	17.35424615	35.2649883132119	0.989440899	19.1151350666889	15.94320892	0.00959187782403324	0.421596332	16.140261368699
Andean Latin America	2000	0.537560512436655	17.36626681	37.4114848903265	1.035949052	19.7623938552121	15.91484559	0.00956031321132969	0.415472173	17.6395307219031
Andean Latin America	2001	0.54257234974372	17.36645415	37.0767335821024	1.067807859	20.0880575315653	15.89309877	0.00958532226638193	0.405547516	16.9790907282707
Andean Latin America	2002	0.547911071290629	17.3734187	38.1485428811158	1.105927816	20.5654120130912	15.87733347	0.00956568476231285	0.390157408	17.5735651832623
Andean Latin America	2003	0.553108983629686	17.34225663	38.1612514292871	1.117553117	20.5884776890359	15.85174235	0.00955566973549011	0.372961169	17.5632180705157
Andean Latin America	2004	0.558235444027735	17.27810651	37.2534335612308	1.110974791	20.2295210045191	15.80914264	0.00953052378760384	0.357989074	17.0143820329241
Andean Latin America	2005	0.563091516777997	17.12581205	35.2585380094203	1.041080092	18.7486610610358	15.73573493	0.00949421834460322	0.348997033	16.5003827300399
Andean Latin America	2006	0.567987399260658	17.01836655	35.1013169113073	1.040297703	18.4450963907837	15.63334849	0.0094558656198161	0.344720359	16.6467646549038
Andean Latin America	2007	0.572998971717573	16.92965259	36.699765863692	1.083546546	18.9346309044841	15.50550575	0.00938292972407743	0.340600293	17.7557520294838
Andean Latin America	2008	0.578564192250676	16.81874205	37.0049341391048	1.127219466	19.3316611368466	15.35549528	0.00929456329401573	0.336027306	17.6639784389642
Andean Latin America	2009	0.583998941887463	16.68182843	38.3739950885989	1.15682823	19.4567071619891	15.19377899	0.00924505041813176	0.331221209	18.9080428761917
Andean Latin	2010	0.59020118	16.52453329	38.25401703939	1.174326178	19.41462079883	15.02445061	0.009161360347	0.325756497	18.83023488021

America		0290442		04		04		5822		24
Andean Latin America	2011	0.596944619668801	16.15203964	37.9630905367035	1.189830256	19.2683131942807	14.64611431	0.00896873134098613	0.316095079	18.6858086110818
Andean Latin America	2012	0.603839225091452	15.45667082	37.0171230846656	1.177530739	18.6256283188077	13.97855166	0.00860708985025818	0.300588417	18.3828876760076
Andean Latin America	2013	0.610705168909346	14.73197488	37.681598957306	1.240198325	19.0964470877499	13.2095977	0.00818897966376153	0.282178852	18.5769628898923
Andean Latin America	2014	0.617059985208393	14.08261963	37.4630562638217	1.28621094	19.364463906185	12.53177488	0.00781860837068635	0.264633808	18.090773749266
Andean Latin America	2015	0.622760754184778	13.71281662	37.5819844068797	1.32680724	19.59938059	12.13475797	0.00759875428163319	0.251251407	17.9750050668981
Andean Latin America	2016	0.62816126326043	13.52967083	38.2772096042251	1.364081891	19.8103907827527	11.92838371	0.00747943580314508	0.237205231	18.4593393856693
Andean Latin America	2017	0.633537897231924	13.33629376	39.2586395024194	1.39728051	19.837687416974	11.71999225	0.00735901044836446	0.219021	19.413593074997
Andean Latin America	2018	0.638860163722721	13.23870949	41.1289246351715	1.476734633	20.5861734193032	11.55876365	0.00726570422832895	0.203211205	20.53548551
Andean Latin America	2019	0.643921324827191	13.21999845	42.5222357853725	1.527870988	20.9226547725083	11.49556292	0.00722837659027016	0.196564538	21.5923526362739
Andean Latin America	2020	0.647806818889059	13.16139849	40.7890027065953	1.473186202	20.1002832287289	11.49172539	0.0072268664670088	0.196486905	20.6814926113994
Andean Latin America	2021	0.651602455956311	13.16142783	40.4668940264095	1.478348063	19.9937060239103	11.48659023	0.00722468239977908	0.196489537	20.4659633200994
Australasia	1990	0.73123439665081	142.73605798536	227.866060982968	29.48383558	196.048849200191	29.06838534	0.0188561382320915	84.18383706	31.7983556445453
Australasia	1991	0.73491529	145.93709996875	223.5903776959	29.22155512	191.4320747562	28.65470605	0.018594320674	88.0608388	32.13970861901

		2940003		13		2		0238		87
Australasia	1992	0.739005622229086	149.63558241711	228.403713604792	30.02691073	192.555418377949	28.26788598	0.0184512746102219	91.34078571	35.8298439522327
Australasia	1993	0.743426014100367	152.16569941886	227.18347341334	30.35678692	190.221239370565	27.91955857	0.0182651866268918	93.88935392	36.9439688561477
Australasia	1994	0.747726041121644	154.98746073622	226.553278002523	31.75759031	190.436430888755	27.62602346	0.0181739253682593	95.60384696	36.0986731883994
Australasia	1995	0.752015071895455	157.68010790294	226.336239562844	33.89112875	190.764060320361	27.40329834	0.0180592118750535	96.38568081	35.5541200306079
Australasia	1996	0.756387301681455	158.9714171	223.692093200171	35.58773155	189.415372008834	27.19263426	0.0178749790838084	96.19105128	34.2588462122529
Australasia	1997	0.760990092580495	159.30334124423	221.925374401963	37.05931338	189.525790305515	26.95644059	0.0178272405155488	95.28758728	32.381756855932
Australasia	1998	0.765299501797176	158.6917537	223.45844020175	37.94193168	189.840853287822	26.73652008	0.0175127907766481	94.01330199	33.600074123151
Australasia	1999	0.769521271780446	157.59084671542	223.155230305157	38.30775833	189.034798661408	26.5764879	0.0173528925555494	92.70660048	34.1030787511935
Australasia	2000	0.773791156697847	156.38917504821	219.814713576366	38.14560397	185.948757021578	26.52167249	0.0173377351768875	91.72189858	33.8486188196114
Australasia	2001	0.778073920719092	155.75155557368	220.120888750656	38.42987182	185.945638464776	26.61601043	0.0173730810281502	90.70567332	34.1578772048514
Australasia	2002	0.782480023897558	154.85985710995	219.428492377999	38.74001158	185.01918505286	26.82285318	0.0175011776067756	89.29699235	34.3918061475327
Australasia	2003	0.786285288288186	154.79222597583	219.351885029777	39.89457877	186.352155141931	27.06359468	0.0176462213745303	87.83405253	32.9820836664711
Australasia	2004	0.78942762	154.72379253994	214.7606578357	40.78749933	184.2836769926	27.26048244	0.017742620814	86.67581077	30.45923822227

		4702187		82		9		9602		74
Australasia	2005	0.791721728700336	155.91554576262	215.538917079634	42.40355922	184.121702049634	27.33514176	0.017646732514372	86.17684478	31.3995682974854
Australasia	2006	0.792856546831287	155.97810760182	209.787721695639	42.59511825	178.538754132596	27.21459338	0.017676620992074	86.16839597	31.2312909420506
Australasia	2007	0.793949581488712	156.38846348912	209.504122000136	43.24627879	177.450821548695	26.94507915	0.0175150922075681	86.19710556	32.0357853592333
Australasia	2008	0.796401956546833	157.16034273679	209.862249049569	44.31409416	178.987265820575	26.62752242	0.0173369092575459	86.21872615	30.8576463197368
Australasia	2009	0.799931529585471	156.28706964906	205.121066103661	43.72123912	173.677785434932	26.36578231	0.0171989063404914	86.20004822	31.4260817623881
Australasia	2010	0.804112332299495	154.23233876933	196.026597601856	41.86322799	163.815580300468	26.26347087	0.0170903739675838	86.10563991	32.1939269274204
Australasia	2011	0.808160632647829	154.99567930367	204.58622253205	44.3722337	170.797501457925	26.35618059	0.0172252985073479	84.26726502	33.7714957756177
Australasia	2012	0.81246356265617	151.42832362523	201.679456239835	44.81281148	168.466532993104	26.56089831	0.0173904589481043	80.05461384	33.1955327877824
Australasia	2013	0.817349222974342	147.67869380739	201.900047059415	45.84330957	167.648852542548	26.80569574	0.0174474192843026	75.0296885	34.2337470975824
Australasia	2014	0.821647278182694	142.50624008719	194.145417119792	44.72562974	159.827962252458	27.01994982	0.0175712750757142	70.76066052	34.2998835922578
Australasia	2015	0.825611025242067	136.51733310051	177.381305896145	40.58703064	142.385829475784	27.13018714	0.017659750382838	68.80011532	34.9778166699777
Australasia	2016	0.829456873590488	131.10207274798	157.963850369268	35.40554287	122.8898823	27.14847248	0.0177100554474336	68.5480574	35.0562580269207
Australasia	2017	0.83292442	128.84989819651	148.4281322067	33.35191331	114.5786405790	27.14198199	0.017541311432	68.35600289	33.83195031632

		0350647		87		27		4339		73
Australasia	2018	0.83661889 2913327	128.29587907302	145.9140823601 32	32.92856275	112.6162636203 65	27.12540806	0.017541204503 1933	68.24190826	33.28027753526 35
Australasia	2019	0.84058157 6884194	129.08414020788	148.2895002717 57	33.74181746	115.1259931397 09	27.1143884	0.017645461662 2521	68.22793434	33.14586167038 56
Australasia	2020	0.84333342 7149089	127.19649906428	139.8315745496 94	31.88413936	108.3664024342 59	27.04542501	0.017577379086 3558	68.2669347	31.44759473634 83
Australasia	2021	0.84551406 3311111	127.7519665	141.6682454303 61	32.39610513	109.9734547489 81	27.05046281	0.017617274731 1269	68.30539857	31.67717340664 91
Caribbean	1990	0.51811138 1453294	7.629421361	30.17491060899 61	0.692207366	12.12115317245 05	6.251291381	0.004100983026 12703	0.685922614	18.04965645351 95
Caribbean	1991	0.52278858 5669872	7.862534125	30.33228057268 63	0.71716585	12.13422802137 19	6.445361095	0.004211704121 34731	0.700007179	18.19384084719 31
Caribbean	1992	0.52698419 1623192	8.049196599	30.99523355445 15	0.75251717	12.55468009308 56	6.589633558	0.004293426453 79153	0.707045871	18.43626003491 21
Caribbean	1993	0.53050678 5857999	8.147052206	31.16351989467 29	0.752397887	12.52208046148 26	6.68588868	0.004347524689 08183	0.708765638	18.63709190850 12
Caribbean	1994	0.53358431 0833862	8.231481959	31.58654758067 66	0.788892968	12.78877372662 37	6.735700003	0.004374984420 30653	0.706888989	18.79339886963 26
Caribbean	1995	0.53665059 2516603	8.336287184	33.15699886202 29	0.890752407	14.14815874908 33	6.742074597	0.004377763076 705	0.70346018	19.00446234986 29
Caribbean	1996	0.53977593 1989678	8.373293283	34.86923571558 23	1.020296279	15.55872845680 69	6.666210167	0.004333270701 08862	0.686786838	19.30617398807 43
Caribbean	1997	0.54330288 5513977	8.067642985	34.77732046725 94	0.904559355	13.88472604817 92	6.5097051	0.004242002111 69173	0.653378529	20.88835241696 85
Caribbean	1998	0.54736506	7.862736555	36.22804072272	0.919763569	14.04343720367	6.328159956	0.004136008840	0.61481303	22.18046751020

		5845421		68		78		30451		87
Caribbean	1999	0.552060171937124	7.643460489	36.8978281758356	0.886472648	13.5641830725478	6.174792345	0.00404581097485076	0.582195496	23.329599292313
Caribbean	2000	0.557368943291687	7.585610812	37.3883223221135	0.915921022	13.7925610564175	6.103065054	0.00400220327951764	0.566624736	23.5917590624165
Caribbean	2001	0.563110488199015	7.528824986	37.599397032312	0.873902436	13.2187875995904	6.091636585	0.00399267319262455	0.563285965	24.376616759529
Caribbean	2002	0.569068591798538	7.524580868	37.9640184219367	0.877138482	13.0839802008986	6.086491867	0.00398686999634885	0.560950519	24.8760513510418
Caribbean	2003	0.574888622062261	7.537295796	37.4356799270217	0.893538601	13.0286721066391	6.084532285	0.00398289375887817	0.55922491	24.4030249266237
Caribbean	2004	0.580533314534419	7.502762836	37.2934040088476	0.86434531	12.4772174675172	6.081018784	0.00397791917292266	0.557398742	24.8122086221575
Caribbean	2005	0.585757989506811	7.508192656	37.4666365034936	0.881403411	12.5461875077048	6.071745292	0.0039693016486431	0.555043952	24.9164796941402
Caribbean	2006	0.590602336380845	7.502700164	37.8041772431822	0.9148081	12.7720025573915	6.046956308	0.00395111864853014	0.540935757	25.0282235671422
Caribbean	2007	0.594709606468949	7.425074562	38.1767920730925	0.901879418	12.5815728957847	6.011266428	0.00392581757118205	0.511928717	25.5912933597366
Caribbean	2008	0.59829691009498	7.328226831	38.6419702085082	0.877248243	12.1751436241827	5.97276422	0.00389868563367664	0.478214369	26.4629278986918
Caribbean	2009	0.601654827953653	7.322409306	39.5846568004444	0.93285312	12.7460387648853	5.939610279	0.00387499437959856	0.449945908	26.8347430411795
Caribbean	2010	0.605480076292026	7.293046151	39.3249604934216	0.930370933	12.4653473852759	5.924561315	0.00386395128788873	0.438113903	26.8557491568578
Caribbean	2011	0.60952504	7.312552598	39.07287530527	0.95665966	12.82863125782	5.918319456	0.003858845411	0.437573482	26.24038520203

		5948231		44		76		96049		48
Caribbean	2012	0.61325472126086	7.27901603	38.4079781372168	0.933484225	12.6362788072823	5.908600048	0.00385036171599581	0.436931757	25.7678489682185
Caribbean	2013	0.616876849803668	7.333204846	39.100443590269	0.998132981	13.3374336539139	5.898601384	0.00384159421407023	0.436470482	25.759168342141
Caribbean	2014	0.620442645171873	7.350926771	39.7412127034332	1.026783575	13.6447249068459	5.888407261	0.00383252121261976	0.435735936	26.0926552753747
Caribbean	2015	0.624074667101969	7.317925993	39.9780683776713	1.006200064	13.3866509977854	5.877452901	0.00382278268113701	0.434273028	26.5875945972048
Caribbean	2016	0.627582073766478	7.259022119	40.61215595	1.049901963	13.6697610090929	5.786186353	0.00376386716799888	0.422933803	26.9386310726491
Caribbean	2017	0.630788065739452	7.11246419	40.7156333277758	1.108644065	14.1917699565452	5.602570951	0.00364766591169118	0.401249174	26.5202157053189
Caribbean	2018	0.633972165730267	6.904149138	40.7713942535897	1.10437707	14.1408531039731	5.420016957	0.00353211457108151	0.379755111	26.6270090350455
Caribbean	2019	0.637321229780475	6.826569787	40.9350222854825	1.127485062	14.2959336457609	5.330425583	0.00347416213261546	0.368659142	26.635614477589
Caribbean	2020	0.639708532040586	6.798802328	40.5407984561649	1.11378347	14.0137605733922	5.318473904	0.00346393322049803	0.366544954	26.5235739495522
Caribbean	2021	0.642003054597226	6.775575125	40.149921263234	1.11283616	13.8997740918672	5.299282369	0.00344981566083829	0.363456596	26.246697355706
Central Asia	1990	0.553361945570561	24.23458715	28.7019740291946	1.11465168	19.5942243785517	23.05588692	0.0127140080997527	0.064048553	9.09503564254315
Central Asia	1991	0.555147774282528	24.29482902	29.3867487462351	1.153545468	20.1577909702096	23.07734431	0.0127165750861091	0.063939244	9.21624120093937
Central Asia	1992	0.55726954	24.34691193	29.99813714977	1.185695305	20.65387281517	23.09736181	0.012740357079	0.063854821	9.331523977517

		1264459		35		65		3737		64
Central Asia	1993	0.560082891879757	24.38430853	30.5295582430093	1.206934596	21.1017969194395	23.11357188	0.012753388404658	0.063802051	9.41500793516511
Central Asia	1994	0.562699530509851	24.40644317	30.8501522481727	1.216955474	21.3494334976071	23.12572109	0.012762459119191	0.063766606	9.48795629144637
Central Asia	1995	0.565585601634758	24.45494693	31.6492022286579	1.256907293	22.0589547239022	23.13426944	0.0127447653292801	0.06377019	9.5775027394264
Central Asia	1996	0.568760584200855	24.47314847	31.8670090484258	1.279357809	22.2802878600734	23.13045795	0.0127533577908234	0.063332707	9.57396783056162
Central Asia	1997	0.571766483451116	24.42918219	31.2452157932427	1.253174357	21.7566503536683	23.11370608	0.0127316350535313	0.062301759	9.47583380452089
Central Asia	1998	0.574659386316177	24.38301712	30.7664835681339	1.231474235	21.3551376559363	23.09045528	0.0126994388257211	0.061087605	9.39864647337186
Central Asia	1999	0.578120700874648	24.35228353	30.4450372107492	1.222836236	21.0904917760675	23.0693595	0.0126733298625277	0.060087791	9.34187210481915
Central Asia	2000	0.58215553627779	24.30209839	30.5374165488716	1.181995153	20.5999116215389	23.06037484	0.0126629664801177	0.059728392	9.9248419608526
Central Asia	2001	0.586645798374273	24.29049341	30.7526897223616	1.183524121	20.5838298305572	23.04714946	0.0126375717046837	0.059819835	10.1562223200997
Central Asia	2002	0.591457113244564	24.21591094	30.2802980880949	1.137582664	19.812162660499	23.01842423	0.0126281909362833	0.059904051	10.4555072366596
Central Asia	2003	0.596529769407501	24.1033195	30.1462778150632	1.058044614	18.6007431328137	22.9852927	0.0126156971048364	0.059982188	11.5329189851447
Central Asia	2004	0.601736719181198	24.01326661	29.8904905004595	0.992643281	17.3849918010535	22.96056841	0.0125882191813532	0.060054924	12.4929104802246
Central Asia	2005	0.60729382	23.8929846	27.98020971225	0.87617957	15.44926432	22.95667645	0.012587017320	0.060128577	12.51835837350

		0151786		67				7935		59
Central Asia	2006	0.613078276344782	23.94926847	27.7811145935122	0.90061004	15.5989189691115	22.98844499	0.0125945934298089	0.060213448	12.1696010309709
Central Asia	2007	0.619104577585881	24.06084159	29.4832758389073	0.949078506	16.2107209215785	23.05145405	0.0126016434639884	0.06030904	13.2599532738648
Central Asia	2008	0.624930378379196	24.15708491	29.8522157748547	0.971000421	16.1398534679671	23.12567803	0.0126443256334031	0.060406457	13.6997179812542
Central Asia	2009	0.630102885311406	24.21553988	30.0011742187297	0.963514583	15.7559460757138	23.19153198	0.0126308448921902	0.060493316	14.2325972981237
Central Asia	2010	0.635295596785045	24.2875022	30.6887726105188	0.998252914	15.9773130203385	23.22869512	0.0126271691764446	0.060554168	14.6988324210039
Central Asia	2011	0.640313560990136	24.35040456	31.7883959443823	1.051318992	16.3233063076065	23.23852362	0.0126487544321941	0.060561947	15.4524408823436
Central Asia	2012	0.644673516154135	24.41128186	33.4745099135777	1.113011188	16.8916746757226	23.23774582	0.0126528053976185	0.06052485	16.5701824324575
Central Asia	2013	0.648888141196487	24.31741797	32.1317545193864	1.02528191	15.3334473378802	23.23166043	0.0126536873552767	0.060475628	16.7856534941509
Central Asia	2014	0.652999879929734	24.37110959	33.0965000535541	1.085118758	15.8692747928704	23.22555382	0.0126763804761351	0.060437019	17.2145488802076
Central Asia	2015	0.656817827293337	24.43110875	34.9191342381001	1.14680132	16.4307319969119	23.22387423	0.0126644727668508	0.060433201	18.4757377684214
Central Asia	2016	0.660312421102967	24.47413721	37.7315042680608	1.194128355	16.6884060753926	23.21952227	0.0126523710924752	0.060486585	21.0304458215757
Central Asia	2017	0.663576782037169	24.42397859	36.5404388964759	1.158915234	15.8254482253825	23.20449012	0.0126372885150944	0.060573233	20.7023533825783
Central Asia	2018	0.66666644	24.44426134	37.01290492395	1.193992256	16.16370103370	23.1896064	0.012618074579	0.060662686	20.83658581567

		8854357		73		03		4303		76
Central Asia	2019	0.669837303617765	24.39653764	36.751117453113	1.150560448	15.3621742072997	23.18526093	0.0126090707762603	0.06071626	21.376334175037
Central Asia	2020	0.672486710434048	24.38903703	36.4768770121616	1.14122357	15.0768306448008	23.18710198	0.0126075295445747	0.060711474	21.3874388378162
Central Asia	2021	0.675163978206939	24.37046024	35.6223437850976	1.12085803	14.799811671277	23.18891977	0.0125917936954985	0.06068244	20.8099403201251
Central Europe	1990	0.637272173782064	28.69697143	79.5874803212441	3.512545895	52.5201777694159	22.47944349	0.0131488097224365	2.704982044	27.0541537421058
Central Europe	1991	0.64308262232945	29.45278077	79.9457977126518	3.633517088	53.3619299837899	23.07353454	0.0134054970451059	2.745729142	26.5704622318168
Central Europe	1992	0.648772145233911	30.18513634	81.0594066057819	3.757346942	54.0283960932789	23.64293371	0.013610289754977	2.784855685	27.017400222748
Central Europe	1993	0.654469911707356	30.92631151	82.6548486678533	3.916814683	54.7829103568502	24.18681964	0.013819808921674	2.822677185	27.8581185020814
Central Europe	1994	0.661219387961725	31.69354925	82.2867298283185	4.129270078	55.5054899883754	24.70388907	0.0140758109628144	2.860390101	26.7671640289803
Central Europe	1995	0.667954946822872	32.40370048	82.7304073091384	4.308926652	56.3701703770135	25.19636446	0.0142761022478728	2.898409367	26.345960829877
Central Europe	1996	0.674121166869274	33.1456255	83.0871338105633	4.488240918	57.1121726775407	25.71752766	0.0145111654268393	2.939856928	25.9604499675958
Central Europe	1997	0.680037255188276	33.91162287	83.571995130194	4.698196184	58.1860037587853	26.23251712	0.0147357850589204	2.980909568	25.3712555863498
Central Europe	1998	0.685968373728779	34.52631378	83.3992347698041	4.819241351	58.6002755848025	26.68935333	0.0149573563378066	3.017719106	24.7840018286638
Central	1999	0.69205618	35.13533574	84.08358390156	5.037037001	59.85069534565	27.04924155	0.015153277323	3.049057187	24.21773527859

Europe		4230124		72		01		0882		4
Central Europe	2000	0.698779713226691	35.48509415	83.1484373789581	5.14832361	59.74325328	27.26470637	0.0152631895968905	3.072064179	23.3899209094012
Central Europe	2001	0.705656782889881	35.84428739	83.9117287422147	5.3718249	60.7126792870678	27.38487017	0.0153010716273988	3.087592316	23.1837483835195
Central Europe	2002	0.712000596699111	36.02177098	83.0125088689797	5.45108178	60.0835445185689	27.47183906	0.0153608373333236	3.098850138	22.9136035130775
Central Europe	2003	0.717823643978472	36.38707391	84.3512600969565	5.734573748	61.7719220532727	27.5441091	0.015350486682237	3.108391067	22.5639875570016
Central Europe	2004	0.723494250327768	36.60860971	84.2930901239661	5.868650258	61.846368749522	27.6208371	0.0153198730284346	3.119122353	22.4314015014157
Central Europe	2005	0.728674093307365	36.90115611	83.4161792297686	6.047280437	61.6670275528266	27.72006577	0.0153687830526822	3.133809899	21.7337828938893
Central Europe	2006	0.733476440600071	37.43392069	83.2367908774113	6.252195211	62.1127081401081	28.02795964	0.0155158919755757	3.153765844	21.1085668453276
Central Europe	2007	0.738076674759704	38.34280406	83.4620374248654	6.576681152	63.4969823902779	28.59006272	0.0158163966828329	3.176060181	19.9492386379047
Central Europe	2008	0.743147843017925	39.14459448	81.9273369631359	6.696061984	63.3622311688711	29.24819495	0.0161786783879221	3.200337546	18.5489271158769
Central Europe	2009	0.748765718421887	39.80601089	79.8829732877711	6.744383817	62.6529978049154	29.83676779	0.0164444459037164	3.224859287	17.213531036952
Central Europe	2010	0.754919494877203	40.2328412	78.3459235692239	6.806468804	61.9495465167846	30.17903658	0.0165559263308768	3.24733582	16.3798211261084
Central Europe	2011	0.760349287943668	40.65639521	78.12716007	7.0714061	62.7223051180136	30.31796311	0.0166413975162233	3.267026007	15.3882135503002
Central	2012	0.76480327	41.03703087	79.35674147824	7.351128417	63.66505120659	30.40393834	0.016733125301	3.281964111	15.67495714635

Europe		0577235		96		62		1525		22
Central Europe	2013	0.768567712494674	41.24102591	78.0072967103549	7.481379924	63.6065768670141	30.46543791	0.0167527793963163	3.294208077	14.3839670639445
Central Europe	2014	0.771755473295727	41.36021871	77.5305358133812	7.528873729	62.8464286319205	30.52582484	0.0167577679083518	3.305520134	14.6673494135524
Central Europe	2015	0.774844012185043	41.73486801	80.5117054687514	7.815201334	64.3215395140444	30.60306592	0.0167681397089774	3.316600756	16.173397814998
Central Europe	2016	0.777817246515258	41.73125076	78.0860776973939	7.804923787	63.010842128867	30.601029	0.0167976619256385	3.32529798	15.0584379066013
Central Europe	2017	0.781359319970327	41.4876304	74.6287034833814	7.754133246	61.3731171946465	30.409928	0.0167292241655615	3.323569157	13.2388570645693
Central Europe	2018	0.785476334303034	41.29021017	73.9246415877069	7.894339616	61.5798832292337	30.08535296	0.0165834835148406	3.310517597	12.3281748749584
Central Europe	2019	0.789748976082869	40.82072288	72.4304117427372	7.850991233	60.4085028953769	29.68448712	0.016425442576336	3.285244526	12.005483404784
Central Europe	2020	0.793111122536361	37.65152157	70.1701985040982	7.604671557	58.2680170008182	26.9429687	0.0152206755830046	3.103881313	11.886960827697
Central Europe	2021	0.796244447535816	35.99950197	70.1253956729715	7.66422354	58.3410066176619	25.46256882	0.0145977412820709	2.872709605	11.7697913140275
Central Latin America	1990	0.48578741023851	29.97346123	39.9637652909753	0.670378023	13.5460650488784	27.98846157	0.0150581181531821	1.314621639	26.4026421239437
Central Latin America	1991	0.489558534042101	29.9943026	41.0147208221264	0.694303979	13.867501523554	27.98457104	0.01509465630137	1.315427584	27.132124642271
Central Latin America	1992	0.494110188081027	30.02356803	40.8264779927891	0.725434126	14.3755019814453	27.9827147	0.0150628897330054	1.315419206	26.4359131216108
Central Latin	1993	0.49941796	30.05732513	39.94818948886	0.760198059	14.81115700943	27.98235338	0.015091853473	1.314773695	25.12194062595

America		5912113		64		57		9789		67
Central Latin America	1994	0.505060641375706	30.10798916	39.9470061473166	0.811641989	15.483068247274	27.98263134	0.0150808354439085	1.313715825	24.4488570645987
Central Latin America	1995	0.509874245782476	30.16334476	41.9012988271056	0.868464451	16.1182177238992	27.9825485	0.0150978598947829	1.312331818	25.7679832433116
Central Latin America	1996	0.514693467179789	30.16615731	40.9675951672639	0.871615424	15.6417992513885	27.98522723	0.0150887106961303	1.309314649	25.3107072051793
Central Latin America	1997	0.520170670818824	30.21821734	41.7458851532183	0.922357369	16.1569344965872	27.99158877	0.0150892344249779	1.304271196	25.5738614222061
Central Latin America	1998	0.526131813323221	30.22436612	40.8936531641989	0.9278359	15.9916598580484	27.99826019	0.0151031307858262	1.29827003	24.8868901753647
Central Latin America	1999	0.531807022718199	30.23616871	40.9776154628289	0.94009785	15.9663357574255	28.0033588	0.0151134564127947	1.292712055	24.9961662489906
Central Latin America	2000	0.53730037286636	30.24796082	41.4619783955446	0.9548715	16.0085860237277	28.00432754	0.0151081132713731	1.28876178	25.4382842585455
Central Latin America	2001	0.542439835431694	30.26891926	41.1199904138767	0.984557636	16.3404691109813	27.99768514	0.0151093790058965	1.286676481	24.7644119238895
Central Latin America	2002	0.547134354053144	30.2697681	41.4189838190927	0.998160638	16.4014794751971	27.98557645	0.0151092585072342	1.286031012	25.0023950853884
Central Latin America	2003	0.551446097890775	30.23955698	40.8328674218563	0.981365958	16.0681375454872	27.97206617	0.0150794981343312	1.286124851	24.7496503782348
Central Latin America	2004	0.556011499493668	30.2146608	39.3202268348121	0.966901274	15.7063301908659	27.96148476	0.0150644705381649	1.286274763	23.598832173408
Central Latin America	2005	0.56074953113176	30.26631771	40.5022692785444	1.022653922	16.5416595057765	27.95793828	0.0150611083554527	1.285725513	23.9455486644124
Central Latin	2006	0.56564869	30.29396311	40.68704281470	1.043706274	16.66475347105	27.96750339	0.015052403525	1.282753452	24.00723694012

America		134557		76		88		2294		36
Central Latin America	2007	0.570707267870529	30.31666281	39.4497897354556	1.050234139	16.5808268599912	27.98889535	0.0150612093591524	1.277533314	22.8539016661052
Central Latin America	2008	0.575839326486226	30.38258385	39.9786937313851	1.09701981	17.0715321687557	28.01413742	0.0150410922387105	1.271426621	22.8921204703907
Central Latin America	2009	0.579940059456967	30.41936761	40.2523323874135	1.118906772	17.0682090135508	28.03478282	0.0150606881984585	1.265678022	23.1690626856642
Central Latin America	2010	0.584253169513966	30.44987021	40.0459110553422	1.145772163	17.1929872466723	28.04248724	0.0150752733691301	1.261610811	22.8378485353008
Central Latin America	2011	0.589307552450467	30.4394626	38.8182122822418	1.140136291	16.8878432801154	28.04049237	0.0150720766778415	1.258833943	21.9152969254486
Central Latin America	2012	0.594827165232211	30.47031519	39.0078954345702	1.176662136	17.1587702046292	28.03736164	0.0150772432394908	1.25629141	21.8340479867015
Central Latin America	2013	0.600731021704094	30.48777265	39.2779107844443	1.200578837	17.3147072332967	28.0331712	0.0150703845920555	1.254022611	21.9481331665555
Central Latin America	2014	0.606509289751879	30.50545947	39.0028478295753	1.224731752	17.427798155355	28.02852603	0.0150674985774227	1.252201688	21.5599821756429
Central Latin America	2015	0.611815328903404	30.53111803	38.987570689933	1.257406262	17.6133150412685	28.02304083	0.0150705544460501	1.250670945	21.3591850942184
Central Latin America	2016	0.61708609493694	30.51790243	38.8797924607461	1.291294529	17.9396225228267	27.97700767	0.0150121591861773	1.249600232	20.9251577787332
Central Latin America	2017	0.622442428610684	30.40120391	37.2619252318648	1.267750929	17.4116751942594	27.88413604	0.0149948075227938	1.249316938	19.8352552300826
Central Latin America	2018	0.627764641878286	30.35999019	36.7676694729009	1.316866097	17.8658472411815	27.79377779	0.0149217017684434	1.249346305	18.886900529951
Central Latin	2019	0.63284707	30.32326565	36.06852479702	1.320205063	17.72695335509	27.75399257	0.014897611185	1.249068015	18.32667383074

America		0506264		71		37		0138		84
Central Latin America	2020	0.636995887380403	30.3472682	36.1731318296201	1.350145437	17.9510326722594	27.75009958	0.0149124311668022	1.247023184	18.2071867261939
Central Latin America	2021	0.640685100088415	30.36450405	36.5900905883436	1.384172075	18.1772567325404	27.73667168	0.0148986270338395	1.243660297	18.3979352287694
Central Sub-Saharan Africa	1990	0.302374953023092	5.07323916	19.5492203132611	0.50089789	12.953496600107	4.520055042	0.00244656296314826	0.052286228	6.59327715019098
Central Sub-Saharan Africa	1991	0.305220590903611	5.070248819	19.6105534330314	0.501474843	12.9701241074865	4.51666015	0.00244311274972018	0.052113826	6.63798621279514
Central Sub-Saharan Africa	1992	0.307803568747444	5.063434868	19.5204184932585	0.498736471	12.8686541560086	4.512747058	0.00243941512107169	0.051951338	6.64932492212886
Central Sub-Saharan Africa	1993	0.309082037447476	5.055437882	19.4133651356154	0.495434943	12.7556267306037	4.508201779	0.00243556115988614	0.05180116	6.65530284385186
Central Sub-Saharan Africa	1994	0.309897977588989	5.04817791	19.3726302387884	0.49385549	12.6798815088187	4.502660275	0.00243149567941873	0.051662144	6.69031723429032
Central Sub-Saharan Africa	1995	0.311059245394322	5.04263791	19.4534660939331	0.495680846	12.6964880435972	4.495429994	0.00242698559204821	0.051527071	6.75455106474388
Central Sub-Saharan Africa	1996	0.31267439617356	5.03459339	19.4677574015202	0.495754221	12.6525051725165	4.487426584	0.00242248255072982	0.051412586	6.81282974645299

Central Sub-Saharan Africa	1997	0.314501684648562	5.015893234	19.0755328275057	0.485752229	12.3494331061994	4.478833712	0.0024174335068046	0.051307293	6.72368228779952
Central Sub-Saharan Africa	1998	0.316506275524783	5.007500128	19.1562919511688	0.485884403	12.3791108861407	4.47040321	0.00241234432662003	0.051212515	6.7747687207015
Central Sub-Saharan Africa	1999	0.318568996518438	4.999752909	19.2359373598819	0.48569515	12.4113607313061	4.462928065	0.00240773488855911	0.051129695	6.82216889368723
Central Sub-Saharan Africa	2000	0.320841477036976	4.992211169	19.2558327604863	0.484402622	12.3877701254765	4.456755129	0.00240391120322246	0.051053418	6.86565872380655
Central Sub-Saharan Africa	2001	0.323749194337746	4.985917784	19.1554177569014	0.480020594	12.2799939136115	4.454869809	0.00240179860238922	0.051027381	6.87302204468753
Central Sub-Saharan Africa	2002	0.32756690434276	4.98480131	19.1360090988889	0.477140102	12.189732169707	4.456620315	0.00240093764369159	0.051040894	6.94387599153818
Central Sub-Saharan Africa	2003	0.331934652228618	4.992399584	19.3890087971202	0.481187678	12.2871173233928	4.460135585	0.00240075679518226	0.051076321	7.09949071693224
Central Sub-Saharan Africa	2004	0.337420016836254	4.996838318	19.4707927650946	0.48242518	12.2759231358488	4.46330178	0.00240055630719172	0.051111358	7.19246907293864
Central Sub-Saharan	2005	0.344099266833661	4.9994438	19.539547740779	0.484531603	12.2704870760194	4.463795007	0.00239951051270757	0.051117189	7.26666115424691

Africa										
Central Sub-Saharan Africa	2006	0.351459736075685	5.005015966	19.7823491895321	0.491182978	12.379090812286	4.462706076	0.0023980356939508	0.051126912	7.40086034155211
Central Sub-Saharan Africa	2007	0.359565506393019	5.007770607	19.9499677139446	0.496465918	12.4443462640274	4.460178601	0.00239521069566414	0.051126088	7.5032262392215
Central Sub-Saharan Africa	2008	0.368488175281803	5.01414037	20.2686722892614	0.505686233	12.6077853635841	4.457329571	0.00239208263458204	0.051124566	7.65849484304268
Central Sub-Saharan Africa	2009	0.376669289335938	5.020810566	20.5738083358428	0.514602572	12.7683302528379	4.455079239	0.00238959315338969	0.051128754	7.80308848985155
Central Sub-Saharan Africa	2010	0.385380946482775	5.030450982	20.9330596906713	0.525517316	12.9642810121111	4.453798767	0.00238847055701729	0.051134899	7.96639020800314
Central Sub-Saharan Africa	2011	0.394691758703726	5.042035536	21.2905672667493	0.535504894	13.1437579055699	4.455339118	0.00238923495707521	0.051191524	8.14442012622235
Central Sub-Saharan Africa	2012	0.404233879200515	5.052107074	21.604851607108	0.544066325	13.2872831889521	4.456779654	0.00238995426427641	0.051261094	8.31517846389167
Central Sub-Saharan Africa	2013	0.413522206991658	5.061898939	21.8973446022651	0.552324966	13.4129140144822	4.458237948	0.00239071033110611	0.051336025	8.48203987745178
Central Sub-	2014	0.42257865	5.071084999	22.13734443515	0.56001333	13.50684121768	4.459665266	0.002391490982	0.051406403	8.628111726480

Saharan Africa		0795931		08		82		26373		32
Central Sub-Saharan Africa	2015	0.430987530665413	5.078143706	22.3002051509884	0.566481744	13.5424148264473	4.460212342	0.00239182291137738	0.05144962	8.75539850162968
Central Sub-Saharan Africa	2016	0.438745916786925	5.086539432	22.4394821329633	0.571629785	13.5683397063899	4.463382647	0.00239290263325003	0.051526999	8.86874952394013
Central Sub-Saharan Africa	2017	0.446178584983408	5.095931277	22.5918787919514	0.577169067	13.6090091901738	4.467147533	0.00239366246184947	0.051614677	8.98047593931572
Central Sub-Saharan Africa	2018	0.453249769940327	5.105558773	22.7383867598914	0.583035641	13.6475682918323	4.470827	0.00239439187110726	0.051696131	9.08842407618803
Central Sub-Saharan Africa	2019	0.460159558738537	5.115397557	22.8951856962661	0.590095995	13.6900732215962	4.47354967	0.00239521709126666	0.051751893	9.20271725757865
Central Sub-Saharan Africa	2020	0.466456368419447	5.124671363	23.1592907099901	0.600247155	13.8154385950917	4.472679215	0.00239480907142754	0.051744993	9.34145730582696
Central Sub-Saharan Africa	2021	0.472255650923077	5.128206967	23.2585808721152	0.606147831	13.8620530221995	4.470339948	0.0023936757900948	0.051719188	9.39413417412564
East Asia	1990	0.471179162202213	4.892777669	25.290433749471	0.363304275	9.02366701432715	3.659892018	0.00236200282995453	0.869581377	16.2644047323139
East Asia	1991	0.47993558	4.700353159	25.12481877379	0.364424302	8.955362170626	3.495495497	0.002262040755	0.84043336	16.16719456241

		4224314		69		96		82723		41
East Asia	1992	0.488431396416205	4.589708809	25.1834366573725	0.371105922	8.97900394879452	3.395510602	0.00220090539752749	0.823092286	16.2022318031805
East Asia	1993	0.496859472966304	4.544490731	25.3772453361187	0.383727803	9.1386129971339	3.345630189	0.00217018069215603	0.81513274	16.2364621582926
East Asia	1994	0.504885881304375	4.535024121	25.2997058725681	0.38996638	9.1452391961559	3.330808467	0.00216103038481469	0.814249274	16.1523056460274
East Asia	1995	0.513530274810462	4.550977608	25.0064774388411	0.394905863	9.10736208776421	3.337525534	0.00216535718224819	0.81854621	15.8969499938946
East Asia	1996	0.523757699756695	4.907152195	24.7808588408576	0.395844107	8.97835438295517	3.654402511	0.0023436430946216	0.856905577	15.8001608148078
East Asia	1997	0.53381551348184	5.734849457	24.5841162702355	0.395774806	8.84084882521135	4.398114539	0.00276050592763876	0.940960112	15.7405069390965
East Asia	1998	0.542652548201818	6.735132709	24.7458721542449	0.398126429	8.74523547581171	5.295385883	0.00326271363319647	1.041620397	15.99737396
East Asia	1999	0.551367027531984	7.605351665	24.7767441063205	0.404755962	8.73898369300165	6.071060562	0.00369605080592812	1.129535141	16.0340643625129
East Asia	2000	0.558815666160014	8.029559323	25.0055309573728	0.405832369	8.59793490313739	6.448723993	0.00390556699530737	1.175002961	16.4036904872401
East Asia	2001	0.564234747702678	8.109684661	25.5193431257907	0.407402243	8.45744893772199	6.51552157	0.00394099184475542	1.186760848	17.057953196224
East Asia	2002	0.570798376631889	8.154563741	25.7328831741878	0.405310871	8.27842713261148	6.553809259	0.00396105330824144	1.195443611	17.4504949882681
East Asia	2003	0.578904729001887	8.191127787	26.0441857235977	0.407532323	8.12622922919867	6.580787945	0.00397488428061494	1.20280752	17.9139816101184
East Asia	2004	0.58768266	8.236551828	26.26144433175	0.415343553	7.9786180277731	6.610600779	0.003990072929	1.210607496	18.27883623109

		7850312		67		29		35169		61
East Asia	2005	0.597096172077907	8.30876949	26.2178479200581	0.431001951	7.88495360401275	6.657222941	0.00401409026145142	1.220544598	18.3288802257839
East Asia	2006	0.607273415014958	8.407757698	25.9126972352856	0.447991844	7.82823174978559	6.726502218	0.00405121195586596	1.233263636	18.0804142735441
East Asia	2007	0.617523835613458	8.531130066	26.1400831208928	0.468215526	7.86078221216212	6.814019335	0.00409916658049885	1.248895205	18.2752017421502
East Asia	2008	0.626955795235393	8.67236222	26.8057202541334	0.486075966	7.92574595242901	6.919077417	0.00415709556182911	1.267208836	18.8758172061426
East Asia	2009	0.636271150980068	8.828702365	27.4137240891463	0.502448803	8.01573464851699	7.038309965	0.00422267958915318	1.287943597	19.3937667610402
East Asia	2010	0.64798500987872	9.006437132	27.9831582349151	0.525373652	8.17487895643489	7.170018507	0.00429453170961835	1.311044972	19.8039847467706
East Asia	2011	0.657502070448635	9.235371396	28.4447877608664	0.55687835	8.35726495368002	7.335603889	0.00438591169657268	1.342889157	20.0831368954898
East Asia	2012	0.66292987254691	9.521475943	28.3307504840059	0.576580771	8.37177136428211	7.556392335	0.00450922816437757	1.388502837	19.9544698915594
East Asia	2013	0.668681910994893	9.863272429	28.0223993472668	0.586675368	8.25435134164438	7.831555955	0.00466270598779819	1.445041107	19.7633852996346
East Asia	2014	0.674062749580226	10.26343332	27.4160051930101	0.598101151	8.17244344927661	8.156090217	0.00484244388704055	1.509241955	19.2387192998464
East Asia	2015	0.677306858113326	10.70852361	26.8582867539116	0.604417342	8.11647397621012	8.526236495	0.00504536709240847	1.577869778	18.7367674106091
East Asia	2016	0.68152833229251	11.24240129	26.4819349221541	0.61161796	8.09086940191655	8.980790151	0.00529122267006679	1.649993177	18.3857742975675
East Asia	2017	0.68974757	11.96741443	26.09196618048	0.624382303	8.021836340064	9.602696119	0.005623073319	1.740336005	18.06450676710

		1986607		52		15		87322		12
East Asia	2018	0.698625829276073	12.97123726	25.8800859974254	0.643594011	7.99986594431466	10.45853508	0.00607499292310972	1.869108167	17.8741450601876
East Asia	2019	0.708327812697457	14.32788803	25.7222990275029	0.66095552	7.99231353805059	11.61071469	0.00667906982748828	2.05621782	17.7233064196248
East Asia	2020	0.717709308430576	17.66371158	25.5958898588423	0.670694365	7.94890897921725	14.37760179	0.00808898745955304	2.615415426	17.6388918921655
East Asia	2021	0.725704902048078	36.92988449	25.6852869138037	0.678832715	7.90246496350397	30.49721575	0.0160155279084244	5.753836027	17.7668064223913
Eastern Europe	1990	0.664250164553239	20.14575028	53.7382842658568	2.635017442	37.6839406550299	15.5256627	0.00905888859960931	1.985070138	16.0452847222273
Eastern Europe	1991	0.671452374691644	20.44317966	55.2134285302101	2.766769279	38.7445553959061	15.6770565	0.0091187723627953	1.999353889	16.4597543619412
Eastern Europe	1992	0.678752886948831	20.80051186	57.7590360032118	2.979734319	40.599412413732	15.80767083	0.00917318027564933	2.01310671	17.1504504092042
Eastern Europe	1993	0.683359195466776	21.19388696	61.7317781661688	3.245908862	44.0498169876019	15.92211821	0.00921607482573189	2.025859886	17.6727451037412
Eastern Europe	1994	0.686011586087513	21.48062796	65.6710036128708	3.416786331	47.207043149395	16.02519545	0.00925548039130434	2.038646184	18.4547049830845
Eastern Europe	1995	0.68945934404488	21.58548785	67.2325970363483	3.420151175	48.2861899919666	16.1132478	0.00930199870517088	2.052088882	18.9371050456765
Eastern Europe	1996	0.692791353191819	21.548678	66.5336095405791	3.304498395	47.4554198069661	16.17751138	0.00932763274177224	2.066668223	19.0688621008712
Eastern Europe	1997	0.695202953355902	21.50287452	65.9113328062628	3.204231842	46.523992913824	16.21621343	0.00934330136042208	2.082429245	19.3779965910784
Eastern	1998	0.69693983	21.52017591	66.45786123454	3.181089956	46.05540539468	16.24161994	0.009356254533	2.097466015	20.39309958532

Europe		3144754		94		91		64226		67
Eastern Europe	1999	0.698813649567542	21.59180218	70.0691268997205	3.216446378	46.6409016623123	16.26457269	0.00937603087450006	2.110783112	23.4188492065337
Eastern Europe	2000	0.701011179547123	21.74766486	71.8280575252268	3.341043653	48.7142754059532	16.28661837	0.00937934446517849	2.120002834	23.1044027748084
Eastern Europe	2001	0.70350621709587	21.90733344	72.5172226078479	3.472722521	50.3541591984205	16.30852023	0.00938716037930114	2.126090689	22.1536762490481
Eastern Europe	2002	0.706727141014359	22.01613744	72.8619615871303	3.564120781	51.4143581655611	16.32154308	0.00938745107499684	2.130473569	21.4382159704942
Eastern Europe	2003	0.712025170062336	22.19750593	73.8813528427016	3.722707665	52.6157783192698	16.34073719	0.00938887498631627	2.134061082	21.2561856484455
Eastern Europe	2004	0.71921543629325	22.47302894	74.9158813540557	3.961891013	53.9415168901105	16.37231326	0.00939795315691318	2.138824665	20.9649665107883
Eastern Europe	2005	0.726265709436458	23.00686784	78.8391778010066	4.447945681	57.569820993731	16.41377575	0.0094158006751534	2.145146408	21.2599410066004
Eastern Europe	2006	0.732617451974551	23.20690794	76.6861495147895	4.554651128	56.0362545055949	16.49435265	0.00944733029435013	2.157904165	20.6404476789003
Eastern Europe	2007	0.738786858434947	23.56581933	77.150408356894	4.770606374	56.7267534970908	16.6179603	0.00950892069834827	2.177252657	20.4141459391049
Eastern Europe	2008	0.745450424042438	24.00027164	78.223238437854	5.021555408	57.9538325051591	16.77935713	0.00959506513196553	2.199359097	20.2598108675629
Eastern Europe	2009	0.751070158196508	24.13893055	75.351542201989	4.954047515	55.3895479365754	16.96239523	0.00968965767627105	2.222487806	19.9523046077373
Eastern Europe	2010	0.756606480895099	24.46267852	75.5154071191096	5.08162822	55.5371422305495	17.13797532	0.00977870908652057	2.24307498	19.9684861794736
Eastern	2011	0.76102530	24.60772605	73.39108883616	5.034307311	53.93131744234	17.31095442	0.009863841804	2.262464321	19.44990755201

Europe		4802355		57		39		52265		73
Eastern Europe	2012	0.765422749752295	24.89856504	72.7013968348461	5.125034519	53.6497179521131	17.49109201	0.0099645330644268	2.282438518	19.0417143496686
Eastern Europe	2013	0.770338292575998	25.30216389	72.4593314767806	5.325072628	53.9131552130024	17.6765508	0.0100693338631878	2.300540456	18.536106929915
Eastern Europe	2014	0.775091703655184	25.76273686	73.2517398698288	5.595469951	55.2086677983623	17.85104981	0.0101576337635359	2.316217098	18.032914437703
Eastern Europe	2015	0.779663483531772	26.00680675	72.6497479214725	5.687151161	54.8215881503659	17.99203881	0.010240583221112	2.327616776	17.8179191878855
Eastern Europe	2016	0.784804691244677	26.17803785	71.375677321536	5.729522589	54.0136770538078	18.12281248	0.0103165057704612	2.325702784	17.3516837619577
Eastern Europe	2017	0.790014867226964	26.51701544	71.6500048128426	5.947892251	54.7136980807029	18.25894493	0.0103956121397557	2.310178259	16.9259111199999
Eastern Europe	2018	0.794485575424964	26.8634639	72.499968301632	6.205501272	55.8019070687828	18.366818	0.0104723395610534	2.29114463	16.6875888932881
Eastern Europe	2019	0.798138377230752	26.96074319	71.3529185175674	6.265155572	55.2070415794054	18.41208847	0.010503894106993	2.28349915	16.135373044055
Eastern Europe	2020	0.800505061050603	26.88321325	69.5607644499737	6.185205963	53.8054705037021	18.41248845	0.010505311067787	2.285518843	15.7447886352038
Eastern Europe	2021	0.802851008869645	26.97109161	69.7097703312535	6.270168502	53.9655218896528	18.4134031	0.0105052059200138	2.287520004	15.7337432356807
Eastern Sub-Saharan Africa	1990	0.233622123414569	4.961771362	33.3599513609688	0.987077628	27.0186609888251	3.852612656	0.0021130341275395	0.122081078	6.33917733801615
Eastern Sub-Saharan	1991	0.236340461470335	4.978604408	33.5750271985041	0.992456908	27.165633243021	3.863909591	0.00211859026139856	0.122237909	6.40727536522175

Africa										
Eastern Sub-Saharan Africa	1992	0.238615291385449	4.991245746	33.669679510665	0.994266894	27.1942959729296	3.874571547	0.00212378890854877	0.122407305	6.47325974882689
Eastern Sub-Saharan Africa	1993	0.240816973360 95	5.006638551	33.9280005107682	1.001089864	27.3748456930877	3.883032822	0.00212780259164797	0.122515865	6.55102701508889
Eastern Sub-Saharan Africa	1994	0.242901813912446	5.015251854	34.0354800751909	1.003302129	27.420647583916	3.889162291	0.00213066837657314	0.122787435	6.61270182289832
Eastern Sub-Saharan Africa	1995	0.245423541954266	5.017593398	34.0764877363398	1.00315779	27.3965078960417	3.891386737	0.00213166103929349	0.123048871	6.6778481792588
Eastern Sub-Saharan Africa	1996	0.2485848600935	5.020724225	34.2253911684284	1.007124857	27.473209199552	3.890579335	0.00213081376006737	0.123020032	6.75005115511633
Eastern Sub-Saharan Africa	1997	0.252144544352426	5.022166267	34.3271453318989	1.009831686	27.4955534281398	3.889559244	0.00212936726198346	0.122775336	6.82946253649716
Eastern Sub-Saharan Africa	1998	0.25589050866728	5.026734407	34.5078988991691	1.015564201	27.5976885750042	3.88860045	0.00212778189938647	0.122569756	6.9080825422655
Eastern Sub-Saharan Africa	1999	0.2599571216546	5.024111271	34.4309256733036	1.014110638	27.4704663184638	3.887512409	0.00212622742192966	0.122488224	6.95833312741788
Eastern Sub-	2000	0.26416531	5.024567244	34.47252200364	1.016945448	27.44085699317	3.885147876	0.002124404789	0.122473919	7.029540605671

Saharan Africa		1546351		01		9		37004		72
Eastern Sub-Saharan Africa	2001	0.268772259985925	5.015760475	34.1999746112018	1.010680011	27.1736988815607	3.882487445	0.00212275757794816	0.122593019	7.02415297206315
Eastern Sub-Saharan Africa	2002	0.273484445461516	5.009500966	34.090140059169	1.006841048	26.9349718469991	3.879907897	0.00212102162053983	0.122752022	7.15304719054938
Eastern Sub-Saharan Africa	2003	0.278433674725514	5.006328699	34.1258862197256	1.006192644	26.8409141415322	3.877195386	0.00211913253151475	0.12294067	7.28285294566189
Eastern Sub-Saharan Africa	2004	0.283936614019918	5.004010989	34.1901873054649	1.006212822	26.7869025556323	3.874612766	0.00211727691278645	0.123185401	7.40116747291977
Eastern Sub-Saharan Africa	2005	0.289970390395006	4.99639268	34.0951034763393	1.001501329	26.5976337938846	3.871455614	0.00211518247519191	0.123435736	7.49535449997954
Eastern Sub-Saharan Africa	2006	0.296455257278441	4.990629663	34.0044512314441	0.998239601	26.4290751985947	3.868669322	0.00211289353696799	0.123720741	7.57326313931242
Eastern Sub-Saharan Africa	2007	0.303575909976254	4.987543611	33.9705949837326	0.998119362	26.33329558	3.865433686	0.00210988655921981	0.123990564	7.63518952095338
Eastern Sub-Saharan Africa	2008	0.310918495032424	4.985562577	33.904550560318	0.999282513	26.202554855319	3.862020122	0.00210664524342701	0.124259942	7.69988905975553

Eastern Sub-Saharan Africa	2009	0.318456773439147	4.988523356	33.9639890585942	1.004909389	26.1803976742184	3.859023675	0.00210384205633064	0.124590292	7.78148754231948
Eastern Sub-Saharan Africa	2010	0.326295972475511	4.996167051	34.1154841905325	1.014907065	26.2427872010392	3.856301511	0.00210175792787363	0.124958476	7.87059523156539
Eastern Sub-Saharan Africa	2011	0.334253400533177	4.993940813	34.1880263696306	1.02023064	26.2551276873661	3.848656708	0.00209770402863254	0.125053465	7.93080097823583
Eastern Sub-Saharan Africa	2012	0.341628076204938	4.980737927	34.2196151701168	1.023271647	26.2511698326401	3.832848846	0.00208951555165368	0.124617433	7.96635582192501
Eastern Sub-Saharan Africa	2013	0.349207299648926	4.966265326	34.3323177774532	1.02830311	26.3161765792091	3.814004064	0.00207974553462679	0.123958151	8.01406145270945
Eastern Sub-Saharan Africa	2014	0.357009520008588	4.958579316	34.5207760353192	1.037418103	26.4458054638159	3.797682908	0.00207129999744921	0.123478305	8.07289927150581
Eastern Sub-Saharan Africa	2015	0.364907040695028	4.959598266	34.6390938059844	1.046777001	26.4868708671175	3.789310477	0.00206701308230627	0.123510789	8.15015592578457
Eastern Sub-Saharan Africa	2016	0.372758831690512	4.968440432	34.8220204167624	1.058601815	26.602047357916	3.785685619	0.00206484610813595	0.124152998	8.21790821273829
Eastern Sub-Saharan	2017	0.380499990741678	4.976088995	35.017178551478	1.070962853	26.719876995206	3.78021871	0.00206135118296105	0.124907432	8.29524020508899

Africa										
Eastern Sub-Saharan Africa	2018	0.388355112204887	4.98363697	35.1709233242186	1.08381518	26.7926287034028	3.774240418	0.00205759599822683	0.125581372	8.37623702481761
Eastern Sub-Saharan Africa	2019	0.396344656080834	4.993463922	35.352189522675	1.098477051	26.8868784065166	3.769006678	0.00205462207828372	0.125980193	8.46325649408015
Eastern Sub-Saharan Africa	2020	0.403480212306543	4.993369686	35.5493819705219	1.114421429	26.9955207567119	3.752797291	0.00204636459575022	0.126150966	8.55181484921429
Eastern Sub-Saharan Africa	2021	0.409720982688906	5.005679597	35.6061721314914	1.125570243	27.0264161982592	3.753688051	0.00204651745472258	0.126421303	8.57770941577748
High-income Asia Pacific	1990	0.767803785666359	3.950422451	14.1957533113413	0.880840715	6.91499435915154	2.638970566	0.00244376749400059	0.43061117	7.27831518469575
High-income Asia Pacific	1991	0.773332907931811	4.007967494	13.822513076298	0.903225715	6.91631572041567	2.668492448	0.00246240699365283	0.436249331	6.90373494888864
High-income Asia Pacific	1992	0.778834454121466	4.076214239	13.7724285112393	0.937596008	6.99546485852788	2.696936681	0.00248072030077156	0.44168155	6.77448293241063
High-income Asia Pacific	1993	0.783967343562873	4.127194521	13.4015786865364	0.956945215	6.91597545780558	2.723533446	0.00249809381382171	0.44671586	6.48310513491699
High-income Asia Pacific	1994	0.789028456505294	4.157826195	13.0501693549908	0.95811708	6.6719334230463	2.748420405	0.00251458926228382	0.45128871	6.37572134268225
High-income Asia Pacific	1995	0.794099241761951	4.393155004	14.3441609931212	1.167398487	7.51702063298102	2.7705668	0.00252955301837841	0.455189717	6.82461080712183
High-income	1996	0.79886294	4.507049836	14.71714676079	1.25499159	7.628515487950	2.793142389	0.002545346803	0.458915857	7.086085926044

Asia Pacific		2733738		83		99		06113		2
High-income Asia Pacific	1997	0.803127763476871	4.560098177	14.3385252244269	1.279677473	7.49596767200764	2.817426616	0.00256270129365235	0.462994088	6.83999485112565
High-income Asia Pacific	1998	0.806831344523233	4.598499591	14.1899106134234	1.29043906	7.37372077668412	2.841003181	0.00257981962864667	0.46705735	6.81361001711065
High-income Asia Pacific	1999	0.810468965547939	4.599976886	13.9288196025301	1.26772728	7.19782817785515	2.861432089	0.00259481329075189	0.470817517	6.72839661138422
High-income Asia Pacific	2000	0.814089018045995	4.650000276	13.8702925726965	1.299319176	7.29779402228845	2.876676904	0.00260627038109659	0.474004196	6.56989228002693
High-income Asia Pacific	2001	0.817544763901531	4.730646039	13.9800504248404	1.363063381	7.55734171447573	2.890684427	0.00261699782017428	0.47689823	6.42009171254454
High-income Asia Pacific	2002	0.82111049092479	4.723208129	13.5160557952704	1.335714942	7.36108806194941	2.907406344	0.00263011459713055	0.480086843	6.15233761872384
High-income Asia Pacific	2003	0.824635809128573	4.783136717	13.5523276070745	1.374967206	7.44735865029315	2.924759554	0.00264353608207478	0.483409957	6.1023254206993
High-income Asia Pacific	2004	0.828073761162773	4.881240632	13.5061435020045	1.45039322	7.50829711740334	2.943745432	0.00265775658703695	0.48710198	5.99518862801416
High-income Asia Pacific	2005	0.831062144544689	5.023894969	13.5848216302367	1.570473694	7.67851813643151	2.962417188	0.00267083275873587	0.491004087	5.90363266104644
High-income Asia Pacific	2006	0.83376632572486	5.152715397	13.5585260310508	1.672368633	7.76180746058506	2.984379118	0.00268515134493258	0.495967646	5.79403341912085
High-income Asia Pacific	2007	0.836546108092258	5.255218744	13.3806168159532	1.741005322	7.82876378439952	3.011958804	0.00270323165508508	0.502254618	5.54914979989862
High-income Asia Pacific	2008	0.839279640869941	5.324982112	13.4239800914893	1.774155921	7.83997129430622	3.041933942	0.00272283026507448	0.508892249	5.58128596691804
High-income	2009	0.84177063	5.346510942	13.29717138553	1.760776488	7.690710230208	3.070815834	0.002741573397	0.514918621	5.603719581931

Asia Pacific		4619589		67		14		33043		22
High-income Asia Pacific	2010	0.844682035657586	5.416422006	13.3638687155884	1.800735548	7.80044760422183	3.096169365	0.00275785771000366	0.519517093	5.56066325365655
High-income Asia Pacific	2011	0.847721964559105	5.487014514	13.4441038998953	1.844090679	7.88102860015407	3.119904904	0.00277392811258275	0.523018932	5.56030137162865
High-income Asia Pacific	2012	0.850693447826051	5.51481934	13.3708369531882	1.842658394	7.7262600326237	3.145712947	0.00279289133678634	0.526447999	5.64178402922771
High-income Asia Pacific	2013	0.853671414808462	5.509155742	13.1089812516017	1.804485476	7.4624671959757	3.174641349	0.00281520974705682	0.530028917	5.64369884587896
High-income Asia Pacific	2014	0.856611560874697	5.616995397	13.1711321909597	1.873485163	7.58040394821573	3.209244163	0.00284234219694271	0.534266071	5.58788590054706
High-income Asia Pacific	2015	0.859602611267632	5.575046618	12.5246954239128	1.78410311	7.1169135260325	3.251379076	0.00287530772131741	0.539564432	5.40490659
High-income Asia Pacific	2016	0.862733182568617	5.659509497	12.2714158437502	1.745232899	6.92581847658079	3.359457979	0.00296302936304596	0.554818618	5.34263433780641
High-income Asia Pacific	2017	0.865870239741164	5.87253406	12.1709860202477	1.75461291	6.94235273322474	3.537044014	0.0031087904132622	0.580877137	5.22552449660971
High-income Asia Pacific	2018	0.869072595436971	6.104297078	12.2845575966151	1.790351978	7.0499598965834	3.707984125	0.00324783504097347	0.605960975	5.23134986499075
High-income Asia Pacific	2019	0.872218835372088	6.209884202	12.3081024142171	1.793715928	6.98745026305636	3.797388419	0.00331649561441931	0.618779855	5.31733565554632
High-income Asia Pacific	2020	0.874437254664445	6.197569774	12.1376119940342	1.79093178	6.92500941876832	3.786738488	0.00330153312429777	0.619899506	5.20930104214157
High-income Asia Pacific	2021	0.876767000230308	6.209293264	12.168942614942	1.795727287	6.90579262919901	3.792490177	0.00330408648859548	0.621075801	5.25984589925437
High-income	1990	0.76565986	332.52165521778	101.9242307640	17.10326798	78.80951478149	208.6719425	0.090592701211	106.74644477254	23.02412328139

North America		1501338		95		27		0322		09
High-income North America	1991	0.768049984041072	336.30144643924	101.628904915846	17.37075162	78.5006818818744	207.58760769085	0.0899106670551201	111.34308712773	23.0383123669165
High-income North America	1992	0.77154981887738	340.01022834049	101.144693834603	17.80417297	78.1764272798971	206.94201934265	0.089502092673479	115.26403602702	22.8787644620323
High-income North America	1993	0.77497762630805	343.47385596578	101.323575952789	18.43932402	78.6820792561212	206.63715148275	0.0894620679257827	118.39738046555	22.5520346287417
High-income North America	1994	0.7782067547068	346.05232293156	100.013214393518	18.92937901	77.8917001524672	206.53532564426	0.0891093425355512	120.58761828107	22.0324048985149
High-income North America	1995	0.781348237094478	348.27809929611	100.014665289126	19.9298932	78.6965044140008	206.59076814094	0.089069629474541	121.75743795207	21.2290912456506
High-income North America	1996	0.784104747086797	350.3295381	99.8622608722394	20.80807233	78.9895486574462	208.00197682223	0.0896626227163003	121.5194889	20.7830495920769
High-income North America	1997	0.786347610069372	352.98455201359	97.7369136402911	20.87741668	77.366020528193	211.93128628912	0.0910657265211795	120.17584904207	20.2798273855769
High-income North America	1998	0.788569028213136	357.70260153501	96.721565842425	21.06344328	76.7029591399867	218.09422331843	0.093441689402647	118.54493494011	19.9251650130356

High-income North America	1999	0.791196294182564	364.72975395933	96.6220678302507	21.19034718	76.0369280402361	226.1441131	0.0965966020804239	117.39529366379	20.4885431879342
High-income North America	2000	0.794997034341345	374.91123655156	96.1305052782371	21.47701737	75.6929056953957	235.85164519889	0.100245220841945	117.58257397801	20.3373543619995
High-income North America	2001	0.799274822192144	416.7236212	97.0295916915588	21.83798155	75.645858320308	265.11053346899	0.112173514225049	129.77510622092	21.2715598570258
High-income North America	2002	0.802961331201887	499.42003042589	96.9251864969127	21.82005099	74.6274166064059	320.14207423855	0.134113489976608	157.45790520023	22.1636564005302
High-income North America	2003	0.805821250693291	595.3781171	97.306482425384	21.90567872	73.978575685605	382.95227010502	0.15935396525637	190.5201682	23.1685527745226
High-income North America	2004	0.807991190858455	676.12537789178	97.5351021455408	22.02007703	73.2847946693156	435.38483628679	0.180524051310198	218.72046457308	24.069783424915
High-income North America	2005	0.808886690716908	713.92499323012	99.2811009138222	22.49937096	74.3587297893045	459.49077423772	0.190012972215428	231.934848	24.7323581523023
High-income North America	2006	0.809721162464824	719.1773586	99.507736209475	22.42367677	74.1128712456162	462.84469990186	0.190959020514237	233.9089819	25.2039059433446
High-income North	2007	0.812486105704039	723.26643219434	98.4147796781744	22.21571451	72.7327924124014	465.54528480146	0.191881291177265	235.50543288328	25.4901059745957

America										
High-income North America	2008	0.817083931447835	726.81959306018	98.1071879042789	22.37093713	72.4538938679521	467.66800246672	0.192673693548613	236.78065346675	25.4606203427782
High-income North America	2009	0.822470006399231	729.89978637073	99.7257586184461	23.15166727	73.8782724550191	469.10255527066	0.193048834708103	237.64556382937	25.6544373287189
High-income North America	2010	0.827814138426882	731.14098130226	97.5622140736227	22.76056778	71.6872657057881	470.11469777572	0.19322983439574	238.26571574927	25.6817185334389
High-income North America	2011	0.832060879441146	731.57388276534	95.8396416039678	22.28849815	69.8325260963805	470.66499520847	0.19339303176769	238.62038940216	25.8137224758196
High-income North America	2012	0.83566893715831	731.79176459672	94.6020161995484	21.77239755	68.3203541462554	471.09059953248	0.193608550965009	238.92876751553	26.088053502328
High-income North America	2013	0.839058684688836	731.9167986	93.5691374948136	21.24085638	66.9604895520103	471.45479698384	0.193689995672133	239.22114527719	26.4149579471312
High-income North America	2014	0.842340952974672	731.54708019827	91.8777154549274	20.44151139	64.5600326196836	471.6820192	0.193673009028305	239.42354956744	27.1240098262155
High-income North America	2015	0.845876702238159	731.03426266055	88.4819349937254	19.32729005	60.7902917735973	472.02626378137	0.193927453197129	239.68070882921	27.497715766931
High-income	2016	0.84942336	730.50521134739	85.06234910325	18.36363996	57.43936504045	472.28930878383	0.193703959526	239.85226260233	27.42928010327

North America		8063407		98		46		563		86
High-income North America	2017	0.852960114928033	730.27310984158	82.7657745407204	17.62004626	55.1559454133219	472.60604661483	0.193554904049609	240.04701696906	27.4162742233489
High-income North America	2018	0.856316178002531	730.32359899371	80.8421669298755	17.08992524	53.3547761268824	472.96082332782	0.193245125883646	240.27285042626	27.2941456771095
High-income North America	2019	0.859749396624046	730.27693716417	79.0803927659363	16.67915805	51.887803985562	473.18027676876	0.193326358562664	240.4175023	26.9992624218116
High-income North America	2020	0.861874247369976	730.3746503	78.1296560212148	16.53032634	51.1567550734857	473.31337750075	0.193431891077892	240.53094644435	26.7794690566512
High-income North America	2021	0.86346547399092	730.15773850159	77.9578292196145	16.60682444	51.0623427993397	473.13995273573	0.193095017086637	240.41096132131	26.7023914031882
North Africa and Middle East	1990	0.437420668280208	17.51887907	27.0212262738947	0.936961386	8.97381078287351	7.032368464	0.00449190633436764	0.790109687	4.53231044773945
North Africa and Middle East	1991	0.445920934165917	18.71132477	26.6749630513073	0.971095661	8.92035332741747	7.510309412	0.00475465542285672	0.874257313	4.41237354281333
North Africa and Middle East	1992	0.453971432229708	19.79385282	26.6322547570959	1.013008171	8.91848184224921	7.936114063	0.00498886454809024	0.947804178	4.39265667175063

North Africa and Middle East	1993	0.462102156430768	20.68642796	26.6367178562693	1.052275147	8.91154771326496	8.284345698	0.00517855217409996	1.006593133	4.40163266269558
North Africa and Middle East	1994	0.470393818546454	21.31952481	26.6248194914521	1.082619443	8.89453878112611	8.530870798	0.00529812713189212	1.046272163	4.41257283746804
North Africa and Middle East	1995	0.478553399262448	21.65309687	26.7692830741705	1.112583515	8.91137183596906	8.651512891	0.00535726455316889	1.062452031	4.467912437
North Africa and Middle East	1996	0.486462857930485	21.72357004	26.6245321160833	1.138623802	8.86288893159141	8.665911888	0.00536524902844279	1.057249329	4.4440118774218
North Africa and Middle East	1997	0.493980049403206	21.66923636	26.4472257399025	1.167080478	8.80099405118017	8.627175985	0.00535845344332958	1.040361718	4.41726036532777
North Africa and Middle East	1998	0.501445054368231	21.56794235	26.1698826277772	1.198466765	8.71442947472837	8.56570569	0.00532673364854496	1.019798718	4.36518510551171
North Africa and Middle East	1999	0.508955783726057	21.50566924	25.8801180184643	1.237815222	8.63639663820451	8.511980022	0.00530924876628689	1.003039375	4.29835312226137
North Africa and Middle East	2000	0.516824214897251	21.56768464	25.5607205929194	1.287454956	8.55652203571019	8.498125412	0.00530280406945889	0.99826195	4.21853545668007
North Africa and Middle	2001	0.524091271102884	21.72099983	25.2471835527257	1.335723029	8.49520336848323	8.521526654	0.00531379958666341	1.003250233	4.12307460829298

East										
North Africa and Middle East	2002	0.531149448763886	21.85940373	24.9143092544744	1.370365506	8.35965077842253	8.550187014	0.00532891743334368	1.009149347	4.09217493138134
North Africa and Middle East	2003	0.538292126445339	21.98183887	24.6841168686793	1.394375089	8.22873875198951	8.581016565	0.00534598999900335	1.015527778	4.10797369235114
North Africa and Middle East	2004	0.545678162603322	22.05699348	24.2180161741012	1.394297119	8.01317393149797	8.612407377	0.00536013709109236	1.021792246	4.09047401846154
North Africa and Middle East	2005	0.553047386497167	22.15955596	24.2069931767712	1.414257231	7.91482159458439	8.638203122	0.0053728945984143	1.027317624	4.18330209920279
North Africa and Middle East	2006	0.560088958980031	22.27645371	24.2967166563385	1.447608653	7.84265257598734	8.658317471	0.00538324931955813	1.03230073	4.30032250286237
North Africa and Middle East	2007	0.566516098606474	22.42444714	24.5735941459939	1.500939992	7.80505356119765	8.674212886	0.00539044039588348	1.037070692	4.4763530714034
North Africa and Middle East	2008	0.572339255445104	22.60987888	25.04397097	1.578293192	7.84943675184831	8.685274954	0.00539403567221221	1.041371292	4.66715469876447
North Africa and Middle East	2009	0.577062164058613	22.80734652	25.747731453733	1.667615154	7.94747792036328	8.691306068	0.00539296270547554	1.044752038	4.92099484379777
North Africa	2010	0.58184904	22.93865158	25.81375385189	1.731836699	7.920574343804	8.690801753	0.005388893174	1.046687336	4.980913688968

and Middle East		0875827		53		36		89064		38
North Africa and Middle East	2011	0.587593075148442	23.03075963	25.9134951369293	1.787516716	7.89578527959098	8.681264727	0.00537955002673973	1.046598371	5.05558273884694
North Africa and Middle East	2012	0.594111701404195	23.06709718	26.0746922604139	1.829681476	7.87630478602016	8.65925562	0.00535446255590512	1.044611494	5.15568688163087
North Africa and Middle East	2013	0.601127065840355	23.06563718	26.4393793316012	1.86897593	7.87388309850295	8.622571181	0.00532806606429322	1.04127148	5.34047850123337
North Africa and Middle East	2014	0.608418687260886	23.04584498	26.661197090781	1.914901248	7.88487991796272	8.570730765	0.0052949557107236	1.037290476	5.44042367171706
North Africa and Middle East	2015	0.615777419646042	22.97912957	26.7005301165666	1.95827063	7.85942134577484	8.498745931	0.00524328797917042	1.032548223	5.48560042452928
North Africa and Middle East	2016	0.623142324826384	22.42560838	26.588010051528	2.021325592	7.85522096373967	8.202461129	0.00510361972091411	0.989017471	5.4336804423034
North Africa and Middle East	2017	0.630560772719337	21.2153258	26.2137605622924	2.08693236	7.83568806195363	7.621991433	0.00479894131549292	0.898739107	5.26639327787707
North Africa and Middle East	2018	0.637917914957086	19.77859255	25.7232212889885	2.122138896	7.77191398145383	6.961544072	0.00444482477219282	0.805613308	5.08525183826822

North Africa and Middle East	2019	0.645094792390707	18.70319499	25.6045177957232	2.177332188	7.78613951451595	6.42104663	0.00414934423847333	0.753218676	5.01197003910718
North Africa and Middle East	2020	0.651673492602445	17.64180156	25.2750602798576	2.166529693	7.63819167253696	5.910046821	0.00385395283516591	0.744324267	4.99548451455668
North Africa and Middle East	2021	0.658224715222995	17.82885853	25.0130435400145	2.190745709	7.59773605168713	5.982397032	0.00389040530518809	0.741286525	4.90489531301494
Oceania	1990	0.39119508179817	0.469595705	18.5777780876238	0.322278064	8.04745679309921	0.131554912	8.21923157503823e-05	0.015762729	10.5302391022088
Oceania	1991	0.39441882219909	0.470057391	18.6873574417053	0.323048488	8.0718274493268	0.131274643	8.19676315240495e-05	0.015734259	10.615448024747
Oceania	1992	0.397572263576645	0.468656709	18.5996603316139	0.32191481	8.0287742338121	0.131030886	8.17706271027452e-05	0.015711013	10.5708043271747
Oceania	1993	0.401113098804311	0.467818915	18.5930163740134	0.321285485	8.00678334969073	0.130839744	8.16093086427601e-05	0.015693686	10.586151415014
Oceania	1994	0.404892361419349	0.466551074	18.5814736740392	0.320155309	7.98140545052603	0.130713128	8.14932845053812e-05	0.015682638	10.5999867302287
Oceania	1995	0.408507250552117	0.46562584	18.589169439716	0.319284121	7.96464783562119	0.13066343	8.14337594739071e-05	0.015678289	10.6244401703353
Oceania	1996	0.412437065015011	0.466491592	18.7465431283982	0.32013285	8.00724299653645	0.130677361	8.14323072318772e-05	0.015681381	10.7392186995545
Oceania	1997	0.415893126821602	0.464104685	18.7547728472361	0.317689822	7.96361376268663	0.130724707	8.14733260559203e-05	0.015690156	10.7910776112234
Oceania	1998	0.41922484	0.46220944	18.88651476148	0.31571647	7.938276042252	0.130790739	8.153707089372	0.015702232	10.94815718216

		4145035		5		08		24e-05		2
Oceania	1999	0.42252052688375	0.463238079	19.0071470617734	0.316666614	7.98303464244505	0.130856739	8.15989183231509e-05	0.015714726	11.02403082
Oceania	2000	0.425069658649089	0.463858105	19.1570518100269	0.317231248	8.01496904362245	0.130902287	8.16276699814441e-05	0.01572457	11.1420011387345
Oceania	2001	0.427004026028397	0.464869017	19.2810426510954	0.31818451	8.02950910096659	0.13094905	8.16174210403823e-05	0.015735457	11.2514519327078
Oceania	2002	0.428534910024124	0.466188152	19.474288233431	0.319411029	8.06017290777905	0.131026202	8.15828853561728e-05	0.015750921	11.4140337427666
Oceania	2003	0.430059670403891	0.464962224	19.6413164083265	0.318083527	8.032954195	0.131111203	8.15473564791813e-05	0.015767494	11.608280666436
Oceania	2004	0.431581332566915	0.4618258	19.5491539838996	0.314861474	7.96322322540082	0.13118344	8.15253312778648e-05	0.015780886	11.5858492331675
Oceania	2005	0.433151844647519	0.461746537	19.7405720545295	0.314737237	7.98920060446105	0.131222631	8.15169306243867e-05	0.015786668	11.7512899331378
Oceania	2006	0.434584099146577	0.464484781	19.8655438974615	0.317385922	8.03788004473898	0.131321423	8.15531095677448e-05	0.015777436	11.827582299613
Oceania	2007	0.436101754919749	0.465530364	20.007518158902	0.318240956	8.06206476801594	0.131534461	8.16429739423328e-05	0.015754947	11.9453717479121
Oceania	2008	0.437392440444106	0.466185674	19.847817837872	0.318673467	8.04955598948685	0.13178453	8.17481230225873e-05	0.015727678	11.7981801002621
Oceania	2009	0.438807984145715	0.464202523	19.6139537474228	0.316503949	7.96946611313479	0.131994595	8.18306523417547e-05	0.015703979	11.6444058036357
Oceania	2010	0.440751869062464	0.466189599	19.8449767742853	0.318409979	8.04656195536247	0.13208702	8.18543122718088e-05	0.015692601	11.7983329646106
Oceania	2011	0.44261776	0.46870616	20.01481040270	0.32091564	8.117695764361	0.132096895	8.183550333059	0.015693625	11.89703280283

		924088		07		21		73e-05		62
Oceania	2012	0.444524398872964	0.468879214	19.9737734936224	0.321072917	8.12503105281167	0.132106857	8.18188176913098e-05	0.01569944	11.848660621993
Oceania	2013	0.446593513975053	0.465962564	19.8518612690564	0.318139421	8.03735011282791	0.132115762	8.18037943231659e-05	0.015707381	11.8144293524342
Oceania	2014	0.449363523837029	0.46642942	19.9939740036489	0.318591184	8.05804636193842	0.132123761	8.1789921970702e-05	0.015714475	11.9358458517885
Oceania	2015	0.452397260715871	0.467338591	20.2203429204573	0.319490034	8.10086571837673	0.132130907	8.17768021462161e-05	0.01571765	12.1193954252784
Oceania	2016	0.455453805026941	0.466202347	20.1544601971043	0.318502326	8.0442697390428	0.131984421	8.16707476096119e-05	0.0157156	12.1101087873139
Oceania	2017	0.458377791874918	0.467783165	20.4038222003374	0.320421278	8.0959983792227	0.131650892	8.14480789734219e-05	0.015710995	12.3077423730357
Oceania	2018	0.460818102527177	0.466020856	20.3912146332402	0.319001209	8.05514209709086	0.131313282	8.122256222198e-05	0.015706365	12.3359913135871
Oceania	2019	0.463333934467991	0.467210128	20.5771507233615	0.3203474	8.10291732608832	0.131158489	8.11114525334629e-05	0.015704239	12.4741522858206
Oceania	2020	0.46553464291317	0.465474986	20.4446103184789	0.318612849	8.04669436431955	0.131158	8.10989110135018e-05	0.015704137	12.3978348552483
Oceania	2021	0.46744512593133	0.46123445	20.2684898578064	0.314372143	7.92487128639829	0.131158683	8.10877191820752e-05	0.015703624	12.3435374836889
South Asia	1990	0.319796516550129	3.237490779	25.1308946999399	0.218357368	5.78171573624267	1.250808292	0.00069944010136659	0.149579729	6.78303217362592
South Asia	1991	0.325854134215593	3.218097492	25.2421055163405	0.219949193	5.8107432492034	1.241256691	0.000694382695096697	0.147842862	6.80961512627173
South Asia	1992	0.33194096	3.207570351	25.57173666596	0.223132615	5.859047747816	1.23424293	0.000690738529	0.146409631	6.926129846636

		0847559		51		93		00995		6
South Asia	1993	0.33801338 4272727	3.201637571	25.72544541202 5	0.225511872	5.895192804935 54	1.229967161	0.000688621370 794319	0.145339753	6.966841279706 18
South Asia	1994	0.34432290 1064293	3.211840156	26.14321037716 15	0.23262316	6.008969082606 54	1.228610763	0.000688143668 702899	0.144686155	7.061947962305 51
South Asia	1995	0.35072645 2621116	3.22004554	26.66748615092 32	0.23533414	6.051125807855 83	1.230173091	0.000689325452 470795	0.14451554	7.281927942153 29
South Asia	1996	0.35728656 8884949	3.240640579	26.67386074495 08	0.236952439	6.097880730338 64	1.238320102	0.000694162719 375381	0.145047748	7.238355479417 36
South Asia	1997	0.36371921 5289632	3.276592815	26.55899647610 61	0.238760272	6.143834628249 94	1.253377889	0.000702902666 13718	0.146158247	7.134960707136 96
South Asia	1998	0.37028757 6153282	3.318104295	26.64526516549 01	0.240458797	6.166148108182 64	1.271082104	0.000713060626 030357	0.147511245	7.155771413936 37
South Asia	1999	0.37697932 1648286	3.352660573	26.07146755795 58	0.239995081	6.139090339034 61	1.287453137	0.000722297127 630581	0.148882068	6.895921142815 65
South Asia	2000	0.38338845 6937894	3.378051129	25.89301664558 96	0.240761122	6.123618330959 26	1.29832585	0.000728167842 553759	0.149938593	6.822161823992 98
South Asia	2001	0.38953334 0797327	3.392592936	25.93910346604 33	0.240633997	6.071472903078 4	1.304968582	0.000731204448 87695	0.150693889	6.897347625494 39
South Asia	2002	0.39519406 9188782	3.404034951	25.89799881177 4	0.239599816	5.999084743981 17	1.310985089	0.000733485255 392353	0.151432571	6.949181176650 42
South Asia	2003	0.40098342 3681299	3.413775516	26.03987671558 96	0.238961617	5.929440218693 47	1.315873731	0.000735077964 169626	0.15205241	7.089763061137 17
South Asia	2004	0.40718055 4084282	3.418224944	26.00343572908 88	0.237701038	5.851758139371 39	1.318977339	0.000735955185 068871	0.152434095	7.149223769987 93
South Asia	2005	0.41398665	3.420765396	25.81687640798	0.237497335	5.797653687100	1.320274117	0.000736432677	0.152611246	7.110048084213

		7867736		19		28		532288		13
South Asia	2006	0.421367360629466	3.432542081	26.1066833434215	0.24319469	5.87419581416949	1.320373589	0.000736756835985829	0.152702762	7.17840910070529
South Asia	2007	0.429261848389977	3.438634184	26.4628448547699	0.247275358	5.89471244680815	1.319323227	0.000736637349052218	0.152718507	7.33597334322773
South Asia	2008	0.43701185864372	3.448372039	26.708855374119	0.253830193	5.9804610729488	1.317711064	0.000736307593366937	0.152644763	7.37323030651734
South Asia	2009	0.445197354775419	3.452905943	26.5313906560509	0.257657696	6.00372689494461	1.31625896	0.000736069598539957	0.152536315	7.2612323634823
South Asia	2010	0.454075791932099	3.46362731	26.6065806660128	0.263898275	6.06014210917579	1.315519981	0.000736133298730531	0.152395399	7.24241209053189
South Asia	2011	0.463339272558672	3.472291012	26.6889245025546	0.268019723	6.07401935353813	1.315919421	0.000736796646654524	0.152206362	7.26970610109253
South Asia	2012	0.473080558458938	3.484160636	26.8442287351185	0.273038367	6.11481990200371	1.317055646	0.000737999777519776	0.151986305	7.306556466
South Asia	2013	0.483434269072904	3.501867926	27.6911562928457	0.280821327	6.1975424159762	1.318412749	0.000739352527957861	0.151699887	7.64729637791867
South Asia	2014	0.494084361660021	3.511610625	28.2409040541523	0.285010951	6.17831015843477	1.319501106	0.000740486056438427	0.151293256	7.94140138258494
South Asia	2015	0.50489146137776	3.528948759	28.1960234493215	0.293085755	6.24277623355356	1.320411354	0.00074130503347512	0.150977271	7.85449418607372
South Asia	2016	0.515310996838947	3.554609371	28.1708548350477	0.299995549	6.29101620106106	1.325774647	0.000743849974391572	0.15153449	7.79366736648839
South Asia	2017	0.525128437427304	3.594717994	28.5091955168946	0.307703132	6.37123317756337	1.33662801	0.000748717605112432	0.153027855	7.88261586327882
South Asia	2018	0.53447740	3.632272781	28.63396872488	0.313124348	6.394786832371	1.348285192	0.000754069829	0.15472685	7.9214434602400

		8353411		23		05		733777		39
South Asia	2019	0.543245695778794	3.660649402	28.7063525670196	0.318480228	6.4024202369013	1.355943392	0.000757995381894033	0.155901081	7.94999805122659
South Asia	2020	0.550585566473338	3.689866531	28.7593426120321	0.325467654	6.43508528809306	1.362888965	0.000764508653760029	0.156576646	7.94382150926923
South Asia	2021	0.55786465678337	3.704403611	28.6447159187755	0.330930298	6.42510275338881	1.364875794	0.000764951902232572	0.156395714	7.8964902540967
Southeast Asia	1990	0.464103640336702	2.610344687	18.0271274063354	0.183997469	4.87642197597771	2.188014663	0.00138431504006902	0.238332555	13.1493211153176
Southeast Asia	1991	0.471749870885322	2.612873449	18.2179903943672	0.185793982	4.90557049638601	2.188194802	0.0013829633196185	0.238884666	13.3110369346616
Southeast Asia	1992	0.479490405523752	2.61475648	18.4612786401791	0.188320166	4.95186890445439	2.187357821	0.00138115612676858	0.239078493	13.5080285795979
Southeast Asia	1993	0.487349417627551	2.617602025	18.7238319020195	0.191054882	5.00454142053057	2.187280793	0.00138002492730125	0.239266351	13.7179104565616
Southeast Asia	1994	0.495348107426376	2.621537734	18.9406964291162	0.19311039	5.04089271109864	2.188835452	0.00138017445343951	0.239591892	13.8984235435641
Southeast Asia	1995	0.503351140117794	2.628172229	19.1589137939734	0.195281217	5.0824597473786	2.192685818	0.00138206250380223	0.240205194	14.075071984091
Southeast Asia	1996	0.511232528083358	2.63671778	19.3579137427961	0.196468688	5.11020073406145	2.199087877	0.00138576501314772	0.241161215	14.2463272437215
Southeast Asia	1997	0.518664154757138	2.645357046	19.4671007617688	0.196491915	5.10627475226064	2.206606965	0.00139047186857992	0.242258166	14.3594355376396
Southeast Asia	1998	0.524439380068146	2.656049303	19.6328591135796	0.197647383	5.13182119409322	2.214906589	0.00139573423833532	0.243495331	14.499642185248
Southeast	1999	0.52966778	2.667012248	19.84773810282	0.199465463	5.176001436604	2.222839821	0.001400654300	0.244706963	14.67033601192

Asia		1344127		5		09		01966		09
Southeast Asia	2000	0.534566608095096	2.676683053	19.9508023959746	0.200246298	5.18922297935223	2.230334747	0.00140495549379679	0.246102008	14.7601744611286
Southeast Asia	2001	0.53901043306637	2.684951108	20.0072610437882	0.201342682	5.19971147318594	2.235616774	0.00140753896270679	0.247991652	14.8061420316396
Southeast Asia	2002	0.54347854772398	2.690088056	20.0974260178116	0.203168047	5.22479396628866	2.236542715	0.00140720545717019	0.250377294	14.8712248460658
Southeast Asia	2003	0.548051005534058	2.693131977	20.1479475509383	0.205251981	5.25675133889691	2.234812056	0.0014050306684086	0.25306794	14.889791181373
Southeast Asia	2004	0.552707418079134	2.695468213	20.1099324869221	0.206984886	5.27911212528831	2.232585734	0.00140244195097183	0.255897592	14.8294179196828
Southeast Asia	2005	0.557391298357281	2.698061193	20.0989580781029	0.208369896	5.28830059553376	2.231079466	0.00140030271149852	0.258611831	14.8092571798576
Southeast Asia	2006	0.562302170530521	2.700623814	20.0529113054842	0.209950738	5.28003770389692	2.228879545	0.00139795202677906	0.261793531	14.7714756495605
Southeast Asia	2007	0.567654258919948	2.703007574	20.0241143562243	0.211280201	5.27017619240575	2.225612995	0.00139474924561194	0.266114378	14.7525434145729
Southeast Asia	2008	0.573208388642394	2.708422359	19.9632267347706	0.212696777	5.25840399710966	2.224644626	0.00139298126661348	0.271080957	14.7034297563943
Southeast Asia	2009	0.578497394141535	2.717022697	19.8846674968914	0.213680481	5.24182435708556	2.227528438	0.0013938821872427	0.275813778	14.6414492576186
Southeast Asia	2010	0.58426847001674	2.727168275	19.8589809100029	0.215639711	5.24019556988478	2.232686588	0.00139675064293349	0.278841977	14.6173885894752
Southeast Asia	2011	0.590414617673014	2.697324249	19.8260109167747	0.217090229	5.24489522883778	2.205043776	0.0013808623017964	0.275190244	14.5797348256351
Southeast	2012	0.59678028	2.61005328	19.63191601123	0.217208961	5.216732945403	2.128506123	0.001336303529	0.264338196	14.41384676229

Asia		5962537		09		53		24972		81
Southeast Asia	2013	0.603144919786686	2.495150223	19.5297332210085	0.218492343	5.2195334046227	2.026220774	0.00127661710149774	0.250437106	14.3089231992843
Southeast Asia	2014	0.609438680634718	2.379449459	19.4741997414036	0.220473734	5.23464243200932	1.921313417	0.00121528634457828	0.237662308	14.2383420230497
Southeast Asia	2015	0.615689065438732	2.293594916	19.5166795295073	0.224431663	5.28709369110452	1.83847097	0.00116671909143495	0.230692283	14.2284191193113
Southeast Asia	2016	0.621839858315707	2.205660481	19.5443497447832	0.226808997	5.3075210435814	1.750628788	0.00111442678517253	0.228222697	14.2357142744166
Southeast Asia	2017	0.627978128819177	2.095773953	19.5603775006291	0.229101629	5.32501722360619	1.640271411	0.00104836536634831	0.226400914	14.2343119116566
Southeast Asia	2018	0.634095876321264	2.00221145	19.6514983394311	0.231633917	5.33632314133002	1.545307522	0.000991311467429521	0.22527001	14.3141838866336
Southeast Asia	2019	0.640106755457432	1.9645013	19.7895232589257	0.234823241	5.3648669872842	1.504739918	0.000966699950413188	0.224938141	14.4236895716911
Southeast Asia	2020	0.645069663124998	1.962876253	19.5895372514821	0.232969347	5.29611535838008	1.504715004	0.000966538190493277	0.225191902	14.2924553549115
Southeast Asia	2021	0.649777295166023	1.966991537	19.7558298486716	0.237374848	5.35161805448335	1.504461092	0.000966345053221457	0.225155598	14.403245449135
Southern Latin America	1990	0.587308121460173	26.99843029	40.4404669817382	1.473726577	25.7482670552094	22.45446334	0.0137892245154832	3.070240368	14.6784107020133
Southern Latin America	1991	0.592118693682922	26.57651946	40.8822860758437	1.529670745	26.3092569500199	22.01312834	0.0136341935684659	3.033720382	14.5593949322553
Southern	1992	0.59807976	26.30828525	43.68840193961	1.701827409	28.70880095105	21.60632301	0.013392740323	3.000134833	14.96620824823

Latin America		0510357		66		79		8144		49
Southern Latin America	1993	0.603588571329245	26.08387291	45.5755896868525	1.84572915	30.5669759141118	21.26667237	0.0132792130338393	2.971471391	14.9953345597069
Southern Latin America	1994	0.609392015876043	26.01442112	48.2394219483509	2.035871294	32.9148290879139	21.02820731	0.0131567422866964	2.950342508	15.3114361181503
Southern Latin America	1995	0.614372848615658	26.04413905	49.8121017934129	2.180626852	34.3299306653822	20.9243494	0.0130711778041618	2.939162803	15.4690999502265
Southern Latin America	1996	0.619260048456637	26.12019797	51.0124481051132	2.282241364	35.0474102030604	20.90334282	0.0130639134113934	2.934613784	15.9519739886414
Southern Latin America	1997	0.624756486772831	26.10025028	51.173834913688	2.278726328	34.1258631632164	20.88962298	0.0130863617489713	2.931900967	17.0348853887226
Southern Latin America	1998	0.630349086083858	26.16384918	52.1976321807696	2.353530444	34.761339919688	20.88012517	0.0130908478320977	2.930193563	17.4232014132495
Southern Latin America	1999	0.635748461217051	26.17148292	51.3865376183497	2.371746285	34.3222126701819	20.87118745	0.0130575638023812	2.928549188	17.0512673843654
Southern Latin America	2000	0.641237434068023	26.28533183	52.9925588641363	2.499051854	35.1434034881676	20.85990006	0.0130497060420196	2.926379911	17.8361056699267

Southern Latin America	2001	0.646040370652539	26.46297232	54.6810572553168	2.656680194	36.8891147654585	20.88009868	0.0130677327932353	2.926193451	17.7788747570651
Southern Latin America	2002	0.649825844483279	26.57846602	54.793267842858	2.705401929	37.1530638417168	20.94396666	0.0130975549079597	2.929097425	17.6271064462332
Southern Latin America	2003	0.652552402911148	26.7815328	55.4812273313497	2.829271932	38.3949071927867	21.0194673	0.0131369192239954	2.932793566	17.073183219339
Southern Latin America	2004	0.655718875570674	26.87070065	55.4574718596396	2.857752672	38.2770834986088	21.07724419	0.0131618712872515	2.935703784	17.1672264897435
Southern Latin America	2005	0.660519129292011	26.89785746	55.0890274563971	2.873390908	37.8110048501571	21.0884061	0.0131707390915209	2.936060444	17.2648518671485
Southern Latin America	2006	0.664386241421523	26.87429903	55.6362230153541	2.978129171	38.3253979423252	20.96597983	0.0131287938784132	2.930190031	17.2976962791505
Southern Latin America	2007	0.667392500398787	26.52190903	54.4211467985685	2.900464626	36.7501627890263	20.7032295	0.0129573457755653	2.918214903	17.6580266637666
Southern Latin America	2008	0.670688619145044	26.21229411	53.9803204628057	2.911513955	36.5379139271921	20.39625881	0.0128160978713249	2.904521348	17.4295904377423
Southern Latin	2009	0.674152390174445	26.03691917	55.1784839740682	3.003397727	37.0433035473685	20.13987633	0.0126014125564788	2.893645118	18.1225790141432

America										
Southern Latin America	2010	0.678761221772206	25.98863623	55.7974057431928	3.073836051	37.0609141126418	20.02530348	0.0125439378367042	2.889496692	18.7239476927143
Southern Latin America	2011	0.684134275404321	26.0331845	55.1898624463195	3.108769241	36.8006047103913	20.0320781	0.0125596528414439	2.892337156	18.3766980830868
Southern Latin America	2012	0.688427908380671	26.00262323	54.0673398279185	3.033496482	35.4680366640204	20.07037659	0.0125804800477288	2.898750153	18.5867226838504
Southern Latin America	2013	0.691715991602521	26.07688645	53.8645958949605	3.036130438	35.3725977412049	20.13266111	0.0126323119961811	2.908094894	18.4793658417594
Southern Latin America	2014	0.696039552428508	26.28849932	54.9079463046234	3.15817033	36.5090989111524	20.21060338	0.0126896441024178	2.919725614	18.3861577493686
Southern Latin America	2015	0.703287228771999	26.4951513	55.0007690181224	3.268550198	36.587648075202	20.29402436	0.012732830508484	2.932576751	18.4003881124119
Southern Latin America	2016	0.710787734658366	26.7923743	54.2251730938148	3.246211442	35.6989737414961	20.57070433	0.0129018827269137	2.975458528	18.5132974695918
Southern Latin America	2017	0.717548417872934	27.31958443	52.3842422622096	3.193437413	34.1787373036861	21.07349907	0.0130974745571921	3.052647946	18.1924074839663
Southern	2018	0.72495215	27.8574156	50.72381692095	3.169150222	33.28838698450	21.56153633	0.013306659300	3.126729044	17.42212327715

Latin America		6546809		85		63		7129		15
Southern Latin America	2019	0.731133816536993	28.13588453	50.4308397413535	3.18571697	32.862524625354	21.79017389	0.0134575250605815	3.159993663	17.5548575909389
Southern Latin America	2020	0.733963356843606	27.97628808	48.0098646283961	3.060976999	31.2150041721579	21.75613928	0.0134522589371094	3.159171794	16.7814081973011
Southern Latin America	2021	0.735984716622088	27.89918496	45.9651515950287	2.992964879	29.9056294682463	21.7491315	0.0133844377325516	3.157088576	16.0461376890498
Southern Sub-Saharan Africa	1990	0.506946633101549	23.01623112	41.3312999135445	1.379344044	30.530270846076	15.97794905	0.0082352227737147	5.658938023	10.7927938446948
Southern Sub-Saharan Africa	1991	0.512377785688971	25.9122173	41.4685303198165	1.388784782	30.575500496103	17.61223962	0.00899531504408951	6.911192899	10.8840345086694
Southern Sub-Saharan Africa	1992	0.517569473057803	28.61700912	42.3560866318549	1.4234063	31.2003743423925	19.13418149	0.00968562394258979	8.059421327	11.1460266655198
Southern Sub-Saharan Africa	1993	0.522772503354064	30.93329349	41.5539320545624	1.397332175	30.5004279238872	20.49510728	0.0102490291836332	9.040854032	11.0432551014916
Southern Sub-Saharan Africa	1994	0.528063316644431	32.84174602	42.3425078061455	1.417777154	30.9907834513312	21.63792766	0.0107534518419376	9.786041202	11.3409709029724

Southern Sub-Saharan Africa	1995	0.533379780605518	34.12433251	42.1263612066066	1.396871165	30.6997556766015	22.50306132	0.0111339121271618	10.22440002	11.4154716178779
Southern Sub-Saharan Africa	1996	0.538780401523438	35.11481322	42.5072550573845	1.401062115	30.7881225480782	23.23794371	0.0114788085132978	10.4758074	11.707653700793
Southern Sub-Saharan Africa	1997	0.544134278750192	36.1469825	44.2439607276823	1.451849742	31.9198590391993	23.98720624	0.0118148445852083	10.70792652	12.3122868438978
Southern Sub-Saharan Africa	1998	0.549141665219989	37.02718635	44.4993725065898	1.460694797	32.1226848429923	24.66129374	0.0120671798431283	10.90519782	12.3646204837544
Southern Sub-Saharan Africa	1999	0.553884659117594	37.70273251	44.7590568127657	1.474936521	32.4224835522986	25.1729159	0.0122899659531233	11.05488009	12.324283294514
Southern Sub-Saharan Africa	2000	0.558490622219779	38.09825401	46.4989961779162	1.534160461	33.8406469093636	25.42621227	0.0123818796658141	11.13788128	12.6459673888868
Southern Sub-Saharan Africa	2001	0.5625112707742	38.15291033	47.3535506440027	1.563929354	34.4284744791233	25.42919299	0.012382221912989	11.15978799	12.9126939429664
Southern Sub-Saharan Africa	2002	0.566049840134913	38.12219362	48.8563435968674	1.615116569	35.5426014044917	25.33540507	0.0123288206497582	11.17167198	13.3014133717259
Southern Sub-Saharan	2003	0.569215186211791	38.06325718	50.5912633688461	1.664244938	36.6994259705002	25.21438306	0.012280176027223	11.18462918	13.8795572223187

Africa										
Southern Sub-Saharan Africa	2004	0.57238969939439	38.02991087	51.9889504136494	1.69618357	37.5605621495234	25.12829104	0.0122257083123694	11.20543626	14.4161625558136
Southern Sub-Saharan Africa	2005	0.576115686359393	38.08539342	52.8039613885483	1.70371261	37.7626908505969	25.13792149	0.0122380431331999	11.24375932	15.0290324948182
Southern Sub-Saharan Africa	2006	0.580684833056164	38.21220161	53.3280884250346	1.71719529	37.9506624308241	25.20747098	0.0122934490827933	11.28753534	15.3651325451277
Southern Sub-Saharan Africa	2007	0.585672998368962	38.34785331	53.2145775494324	1.718674029	37.6786293022299	25.28948396	0.012337013853648	11.33969532	15.5236112333489
Southern Sub-Saharan Africa	2008	0.590405318780599	38.5101388	53.8304049184158	1.739622055	37.9361350303863	25.37466647	0.0123335648439186	11.39585027	15.8819363231856
Southern Sub-Saharan Africa	2009	0.594585067687362	38.6445985	54.0833928394203	1.751926229	37.9509337183634	25.44625125	0.012358992282414	11.44642102	16.1201001287745
Southern Sub-Saharan Africa	2010	0.598835552808155	38.73649244	54.183955316684	1.760379016	37.8103365151944	25.49147565	0.0123558655721816	11.48463778	16.3612629359174
Southern Sub-Saharan Africa	2011	0.603346853362237	38.6423411	54.1668649344011	1.772772185	37.654832256386	25.42099963	0.0123379385017641	11.44856928	16.4996947395133
Southern Sub-	2012	0.60803776	38.3071719	54.18937479685	1.787620865	37.49350414929	25.19962709	0.012306330382	11.31992395	16.68356431717

Saharan Africa		9585606		27		81		813		18
Southern Sub-Saharan Africa	2013	0.612843814463776	37.79253182	54.294085956707	1.806319535	37.3388496959623	24.86090879	0.0121655216700071	11.1253035	16.9430707390747
Southern Sub-Saharan Africa	2014	0.617452594236263	37.16144451	54.78712222	1.840699431	37.5220111489438	24.43314694	0.0120277023378225	10.88759814	17.2530833661084
Southern Sub-Saharan Africa	2015	0.621837579721306	36.45392886	55.413082133291	1.874044999	37.7502416467348	23.94779485	0.011815612116618	10.63208901	17.6510248744396
Southern Sub-Saharan Africa	2016	0.625919059165734	34.97648664	55.8234920164581	1.892111646	37.7846151198136	22.94003645	0.0113779067167803	10.14433854	18.0274989899277
Southern Sub-Saharan Africa	2017	0.629761503242042	32.70353201	54.9443954419169	1.899659197	37.5774153204734	21.38878673	0.0107362800507477	9.415086085	17.3562438413928
Southern Sub-Saharan Africa	2018	0.63341065599326	30.64037745	54.6988474561776	1.917804859	37.5430365096294	19.9661748	0.0101227615388391	8.756397795	17.1456881850094
Southern Sub-Saharan Africa	2019	0.636940189944773	29.75424474	53.9575738169094	1.925506858	37.2886076065871	19.34887651	0.00983628504326598	8.479861368	16.659129925279
Southern Sub-Saharan Africa	2020	0.63988182654798	29.78475974	54.4795019040254	1.958915124	37.672100456435	19.33408109	0.00982867261473618	8.491763522	16.7975727749757

Southern Sub-Saharan Africa	2021	0.642200282056091	29.73044619	54.3623779135065	1.963796955	37.6137140035558	19.29239535	0.00980849733493305	8.474253884	16.7388554126158
Tropical Latin America	1990	0.499587794144496	68.6473652	47.3480139437838	1.259519658	26.9877710081914	64.19512086	0.028340028955741	3.192724682	20.3319029066367
Tropical Latin America	1991	0.504290478406825	69.48669819	47.2671112261835	1.279091694	27.1688389156341	65.08364308	0.028560975828677	3.123963412	20.0697113347207
Tropical Latin America	1992	0.508056827152166	70.3987282	47.6589867649631	1.325966163	27.7079918843277	66.00100947	0.0289569151712052	3.071752562	19.9220379654642
Tropical Latin America	1993	0.511622219151591	71.30557802	48.9372548391068	1.379289732	28.5663149005565	66.89039704	0.0292998505162027	3.035891252	20.3416400880341
Tropical Latin America	1994	0.515330113176832	72.16023868	50.266508353077	1.433532748	29.1131104944321	67.70950153	0.0295944301087093	3.017204406	21.1238034285362
Tropical Latin America	1995	0.519290875116018	72.87987711	49.4373280041798	1.447269821	28.9478880380933	68.41592069	0.0297180525136958	3.016686601	20.4597219135728
Tropical Latin America	1996	0.52349853894041	73.91185392	50.2338263783301	1.482559436	29.1807372053421	69.3718898	0.0302239698483261	3.057404691	21.0228652031397
Tropical Latin America	1997	0.527841480530975	75.39815324	50.7899225571751	1.479442531	28.9347716281919	70.77417037	0.0306907004321078	3.144540336	21.8244602285511
Tropical Latin America	1998	0.53216492084733	77.06822846	51.5866517531413	1.53123555	29.4519690643361	72.28812017	0.031180981378767	3.248872743	22.1035017074264
Tropical Latin America	1999	0.536658546418818	78.49948119	51.3470900742265	1.585989188	30.0093405213941	73.5725444	0.031947715774081	3.34094761	21.3058018370583
Tropical Latin America	2000	0.541715646072007	79.33599139	53.1959370404721	1.661434338	30.9223199827887	74.283853	0.0320938702716515	3.390704055	22.2415231874117
Tropical Latin	2001	0.54693151	79.68446544	54.62538254341	1.687106798	30.98350046655	74.59834092	0.032211084142	3.399017725	23.60967099271

America		4529212		52		99		1382		32
Tropical Latin America	2002	0.552364617546266	79.97422447	55.4557987878175	1.663013497	30.1558744589806	74.91736758	0.0322731615522697	3.3938434	25.2676511672846
Tropical Latin America	2003	0.557691442061791	80.25267405	53.5362819191377	1.659636469	29.6760733753929	75.20998414	0.0324092979033305	3.383053432	23.8277992458415
Tropical Latin America	2004	0.563268657303027	80.48236382	52.6578711921976	1.654517381	29.5028708306831	75.45273846	0.0324499569515443	3.375107978	23.122550404563
Tropical Latin America	2005	0.568897321190288	80.68496638	53.0628220237852	1.674905891	29.6467022585867	75.6313453	0.0326470062567752	3.378715193	23.3834727589417
Tropical Latin America	2006	0.574720305592822	80.92291126	53.6358482385489	1.712032101	30.1682901842762	75.82067128	0.0325749697039955	3.390207879	23.4349830845687
Tropical Latin America	2007	0.580915353341816	81.19918488	53.4658860078049	1.722252187	29.8808220777747	76.07453004	0.032793570233268	3.402402652	23.5522703597969
Tropical Latin America	2008	0.587353637591901	81.48759529	53.6196319378429	1.738109063	29.8187507834421	76.33201376	0.0327908419250679	3.417472469	23.7680903124757
Tropical Latin America	2009	0.593303117121853	81.75844261	53.7680343480328	1.786752879	30.1900463748081	76.53402528	0.0328684037901356	3.437664451	23.5451195694346
Tropical Latin America	2010	0.599646004958967	81.92387789	53.8429858798161	1.83383775	30.199232620417	76.62444531	0.0328456969551175	3.465594827	23.610907562444
Tropical Latin America	2011	0.605956974978664	81.90096188	53.0009259732614	1.822327198	29.4621876809255	76.56688057	0.0329462426037943	3.511754112	23.5057920497321
Tropical Latin America	2012	0.611787310339311	81.74693936	52.0216254468364	1.798620995	28.6725104915942	76.37020375	0.0328220961858041	3.578114619	23.3162928590564
Tropical Latin America	2013	0.617401723560476	81.48380531	51.6394763629738	1.793110069	28.170419024775	76.03619951	0.0327782094791474	3.654495729	23.4362791287196
Tropical Latin	2014	0.62264611	81.11685866	51.53655221897	1.82306149	28.08541680864	75.56343011	0.032594267705	3.730367061	23.41854114262

America		7925496		14		39		1222		24
Tropical Latin America	2015	0.627399632366814	80.635726	52.2414896557034	1.882722159	28.5169787024827	74.95732241	0.0324370402839802	3.795681433	23.6920739129367
Tropical Latin America	2016	0.631533363932431	79.90910242	52.8255554936169	1.898441512	28.4428968934443	74.13741984	0.0321144192073128	3.873241074	24.3505441809653
Tropical Latin America	2017	0.63576517839481	78.71023605	52.4426091789138	1.865091215	27.4873550181938	72.8769493	0.0317599496473608	3.968195539	24.9234942110726
Tropical Latin America	2018	0.640179901301094	76.80488665	51.3566421117404	1.816146293	26.3720812916914	70.9446909	0.0309426788833843	4.044049453	24.9536181411656
Tropical Latin America	2019	0.644693634309773	74.05072583	52.7621385191168	1.876229901	26.8399927734536	68.10991771	0.0300290371426709	4.064578214	25.8921167085205
Tropical Latin America	2020	0.6486028512164	63.56429484	52.797273951395	1.895218659	26.8712438171325	57.89304358	0.0259454614087094	3.776032598	25.9000846728538
Tropical Latin America	2021	0.652442393942196	42.81136618	52.3023520961882	1.920561729	26.9929123114998	37.82428377	0.0181568991113879	3.066520683	25.291282885577
Western Europe	1990	0.746400479135504	48.53402085	65.6856142183335	7.031392283	53.3651458576597	35.95979287	0.0220433219316088	5.542835694	12.2984250387422
Western Europe	1991	0.751670033731766	49.62020319	65.4225393222355	7.232850565	53.549326976727	36.72059824	0.0224236114111932	5.666754388	11.8507887340973
Western Europe	1992	0.757021934404097	50.77846756	66.1593594529308	7.554330676	54.4933215528329	37.43867303	0.022782895210182	5.785463848	11.6432550048877
Western Europe	1993	0.762056782995762	51.99927326	67.1252268104639	8.01995541	55.684345542419	38.08481278	0.0230864437812607	5.894505063	11.4177948242636
Western Europe	1994	0.766693114391754	53.23492061	68.4412386812959	8.632794009	56.9861900904525	38.6158702	0.0233628142186071	5.986256398	11.4316857766248
Western	1995	0.77062172	54.24734895	68.37287162305	9.184398977	57.06103197102	39.00650522	0.023470974725	6.056444748	11.28836867730

Europe		0690411		2		46		9919		14
Western Europe	1996	0.774079854763763	55.14525599	67.9063879985665	9.686674845	56.8817588157809	39.34062808	0.0236648622932348	6.117953067	11.0009643204924
Western Europe	1997	0.777577690685476	56.01805233	68.191708465625	10.14476182	57.4760492518983	39.69121867	0.023785871248779	6.182071842	10.6918733424779
Western Europe	1998	0.780866892314584	56.70556785	68.4932283515831	10.43002392	57.9530925813902	40.03008336	0.0239658292323778	6.245460577	10.5161699409605
Western Europe	1999	0.78401919706477	57.20352112	68.6043349930627	10.5896357	58.4256655995329	40.31295382	0.0241047742147732	6.300931596	10.154564619315
Western Europe	2000	0.787400010949281	57.74355913	70.2172240479971	10.87658663	59.4518652924561	40.52014613	0.0241359239429789	6.34682636	10.741222831598
Western Europe	2001	0.790904402656041	58.15075396	70.9215307672554	11.10510992	59.8241973447599	40.64542165	0.0242194878498071	6.400222382	11.0731139346457
Western Europe	2002	0.794165178043194	58.40734196	70.8633666995257	11.23836255	59.5230266362606	40.70123871	0.0242639926979164	6.467740698	11.3160760705672
Western Europe	2003	0.796961505412726	58.77913128	70.9841802004737	11.53970724	59.8784635729997	40.70203771	0.0243416878779315	6.537386331	11.0813749395961
Western Europe	2004	0.799715719870364	59.21888845	70.995127523846	11.9738352	59.9199728287095	40.64962121	0.02429962876017	6.595432039	11.0508550663763
Western Europe	2005	0.802414037675735	59.83461364	71.0982731865222	12.63743383	60.2491407637494	40.56523239	0.0242346207305698	6.631947422	10.8248978020422
Western Europe	2006	0.805066849571524	60.42604297	71.767735697968	13.37027505	61.0122177689694	40.40521355	0.024156550223779	6.650554373	10.7313613787748
Western Europe	2007	0.807723333559471	60.91642959	73.1453015676453	14.10881982	62.3776535137534	40.14658039	0.0240125656205036	6.661029381	10.7436354882714
Western	2008	0.81048437	61.0961515	73.95952551074	14.58708029	63.24477225263	39.84244008	0.023763537101	6.666631132	10.69098972100

Europe		73863		35		56		7235		62
Western Europe	2009	0.813110221690425	61.12057786	74.6157938729305	14.91674475	63.8192946244594	39.53390077	0.0235560977392657	6.669932341	10.7729431507318
Western Europe	2010	0.816142165327844	61.17412707	75.0718211470087	15.21982616	64.3215334007163	39.27840531	0.0233517778744179	6.675895601	10.726935968418
Western Europe	2011	0.819375238773098	61.45515869	74.0633827535144	15.15358796	63.3411627644121	39.55161239	0.0235281673039136	6.749958343	10.6986918217984
Western Europe	2012	0.82263372582807	62.25871878	72.0109045418281	14.84721635	61.2772301649341	40.49810328	0.0240028702842921	6.913399155	10.7096715066097
Western Europe	2013	0.825786900905849	63.81267833	71.8859687304569	15.0197928	61.0357510194849	41.68659729	0.0245849922819719	7.106288238	10.82563272
Western Europe	2014	0.828609512677857	65.57190554	73.7277051725222	15.61619454	62.6224898144458	42.68620329	0.0251372115995048	7.269507711	11.0800781464769
Western Europe	2015	0.831321798743052	66.10875408	73.5612550186934	15.70040689	62.304568919196	43.06595019	0.0252429767265796	7.342396993	11.2314431227708
Western Europe	2016	0.834216018576141	64.40694796	72.6709902532003	15.37378662	60.5475100661142	41.80826426	0.0246576332449536	7.224897085	12.0988225538411
Western Europe	2017	0.837522174062445	61.18969846	71.0779882298152	15.09345372	58.8759863454264	39.13916098	0.0233346608013035	6.957083753	12.1786672235875
Western Europe	2018	0.840833550124224	57.98365734	69.3346352069336	14.79649203	57.538136129269	36.49702971	0.0220007687222702	6.690135602	11.7744983089423
Western Europe	2019	0.844212108580339	56.46227731	67.9273607911456	14.56549629	56.2439663433227	35.3215088	0.0214415431325281	6.575272217	11.6619529046904
Western Europe	2020	0.846550359387909	55.735936	64.0944213171343	13.73181646	52.8315296458124	35.45100042	0.0215517112491921	6.553119118	11.2413399600727
Western	2021	0.84872631	55.57969276	63.72150685019	13.70657983	52.59393676232	35.31162201	0.021449256559	6.56149092	11.10612083131

Europe		58736		49		26		0732		32
Western Sub-Saharan Africa	1990	0.273700003911079	3.396178989	14.3495154610548	0.466180325	12.2587631581536	2.881684293	0.0016752866567292	0.048314371	2.08907701624445
Western Sub-Saharan Africa	1991	0.277410731953587	3.403179954	14.4709958214982	0.471134519	12.3631596490076	2.88405885	0.00167615686201246	0.047986585	2.10616001562854
Western Sub-Saharan Africa	1992	0.281024680970774	3.409699573	14.6216832302601	0.475884671	12.4939597470286	2.886200979	0.0016769748397619	0.047613924	2.12604650839171
Western Sub-Saharan Africa	1993	0.284431723352711	3.415513914	14.7206063447494	0.47980049	12.5758339927943	2.88848748	0.00167796658815592	0.047225944	2.14309438536691
Western Sub-Saharan Africa	1994	0.287699582930621	3.420484285	14.7901348473253	0.482771098	12.6231060446938	2.890820163	0.00167907374589725	0.046893025	2.16534972888564
Western Sub-Saharan Africa	1995	0.290983200465808	3.423230954	14.8587457971126	0.483769519	12.663236872893	2.892858217	0.00168022927168127	0.046603217	2.19382869494791
Western Sub-Saharan Africa	1996	0.294618466469239	3.425971435	14.8560256892364	0.48294813	12.622580801623	2.896543231	0.00168217598866844	0.046480074	2.23176271162477
Western Sub-Saharan Africa	1997	0.298302443150554	3.428712664	14.8581616018754	0.481105502	12.5608980073485	2.901212021	0.00168450022082804	0.046395141	2.29557909430611
Western Sub-	1998	0.30202579	3.433447114	14.89615005019	0.480781349	12.50080938823	2.906340158	0.001686968467	0.046325607	2.393653693494

Saharan Africa		6872401		77		55		42333		79
Western Sub-Saharan Africa	1999	0.305809778954492	3.436571583	14.994818423832	0.479275271	12.4552586843908	2.910970303	0.00168909028543038	0.046326008	2.53787064915573
Western Sub-Saharan Africa	2000	0.309780958279431	3.438323588	15.103540718771	0.47848455	12.3522657836463	2.913515668	0.00169018344297475	0.04632337	2.74958475168169
Western Sub-Saharan Africa	2001	0.314028091995735	3.442104656	15.4117637424088	0.481292115	12.3815385345802	2.914366044	0.0016899256732298	0.046446497	3.02853528215535
Western Sub-Saharan Africa	2002	0.318880998672762	3.441976356	15.4211424537079	0.482094718	12.3174778826555	2.913220305	0.00168791304837697	0.046661334	3.10197665800405
Western Sub-Saharan Africa	2003	0.324232893209818	3.439650984	15.3779980653677	0.481238767	12.2271877437691	2.91148372	0.00168530578287976	0.046928496	3.14912501581568
Western Sub-Saharan Africa	2004	0.330228268469955	3.438788966	15.3584832917839	0.481554273	12.1567627300685	2.909999086	0.00168290179925612	0.047235608	3.20003765991618
Western Sub-Saharan Africa	2005	0.336779707492596	3.436450805	15.2972859268137	0.48015258	12.0451395234223	2.908824291	0.0016811970858177	0.047473934	3.25046520630559
Western Sub-Saharan Africa	2006	0.343248174515047	3.437576264	15.2385127060873	0.480951236	11.9395181656391	2.90886668	0.00168019063964494	0.047758348	3.29731434980853

Western Sub-Saharan Africa	2007	0.350040166308299	3.43854095	15.2422021500259	0.48226195	11.908547020445	2.908200382	0.00167835384167204	0.048078618	3.3319767757392
Western Sub-Saharan Africa	2008	0.356716457285134	3.43868407	15.1144088891437	0.483060126	11.7589732529156	2.9072222	0.00167624828437378	0.048401743	3.35375938794372
Western Sub-Saharan Africa	2009	0.363491542715889	3.444787565	15.2618396011514	0.490028593	11.8476380101717	2.906037036	0.00167420398549034	0.048721936	3.41252738699416
Western Sub-Saharan Africa	2010	0.370608753	3.453997522	15.4404091928959	0.501211685	11.97480042	2.903842408	0.00167215121489056	0.048943429	3.46393662201105
Western Sub-Saharan Africa	2011	0.37760914387546	3.460427269	15.6093000360988	0.509409433	12.0845769866087	2.901859596	0.00167055634951792	0.04915824	3.52305249314058
Western Sub-Saharan Africa	2012	0.384753385790014	3.463777833	15.7831153856267	0.515454994	12.1995327847697	2.898986835	0.00166840312864799	0.049336004	3.58191419772838
Western Sub-Saharan Africa	2013	0.391890442560589	3.466869577	15.9712272376933	0.521551169	12.3343298051966	2.895857014	0.00166613792487977	0.049461394	3.63523129457179
Western Sub-Saharan Africa	2014	0.399162089672576	3.467420702	16.0770087129989	0.524908545	12.410590761273	2.892905852	0.00166398790953414	0.049606305	3.66475396381635
Western Sub-Saharan	2015	0.406238200614069	3.463878996	15.976592177461	0.524741265	12.2974546460075	2.889412399	0.00166159290665405	0.049725332	3.67747593854684

Africa										
Western Sub-Saharan Africa	2016	0.412927286930464	3.468051127	16.086777342964	0.532150931	12.3816558250613	2.886028152	0.00165920696387141	0.049872044	3.70346231093887
Western Sub-Saharan Africa	2017	0.419615018790548	3.46786494	16.0710644346719	0.53625187	12.3623735705899	2.881562024	0.00165606865438103	0.050051046	3.70703479542759
Western Sub-Saharan Africa	2018	0.426437347108723	3.465597231	15.9912894428083	0.538350104	12.2894886125187	2.877027636	0.00165292332126896	0.050219492	3.70014790696835
Western Sub-Saharan Africa	2019	0.433383889320038	3.464781736	15.9390635406757	0.541141451	12.2371388168948	2.87329826	0.00165034900747961	0.050342025	3.70027437477337
Western Sub-Saharan Africa	2020	0.439822327028239	3.464060318	15.9008446691399	0.545291546	12.1936276039592	2.868435397	0.00164725866028453	0.050333375	3.70556980652038
Western Sub-Saharan Africa	2021	0.446022978508802	3.465180044	15.9284854451105	0.552766326	12.2179007500477	2.862178491	0.00164341874251066	0.050235227	3.7089412763203

Total skin cancer includes malignant skin melanoma, non-melanoma skin cancer (basal-cell carcinoma) and non-melanoma skin cancer (squamous-cell carcinoma). DALYs, disability-adjusted life-years; SDI, socio-demographic index.

TABLE S23 Age-standardized rates of incidence and DALYs of total skin cancer for global and SDI regions, by year (1990, 2021), age group and sex, with age-standardized rates and 95% uncertainty intervals.

Location name	Age group	Incidence				DALYs			
		Male		Female		Male		Female	
		Age-standardized rate (95% UI), 1990	Age-standardized rate (95% UI), 2021	Age-standardized rate (95% UI), 1990	Age-standardized rate (95% UI), 2021	Age-standardized rate (95% UI), 1990	Age-standardized rate (95% UI), 2021	Age-standardized rate (95% UI), 1990	Age-standardized rate (95% UI), 2021
Global	15-19 years	0.12 (0.1 to 0.13)	0.13 (0.11 to 0.14)	0.19 (0.17 to 0.21)	0.23 (0.18 to 0.27)	2.16 (1.63 to 2.51)	1.6 (1.12 to 2.05)	2.27 (1.62 to 2.74)	2.12 (1.32 to 3.12)
Global	20-24 years	1.9 (1.2 to 2.82)	2.08 (1.36 to 3)	2.36 (1.55 to 3.31)	2.62 (1.82 to 3.62)	6.73 (5.53 to 7.68)	5.35 (4 to 6.42)	6.94 (5.47 to 8.33)	6.63 (4.57 to 8.86)
Global	25-29 years	3.7 (2.39 to 5.49)	3.96 (2.59 to 5.77)	5.03 (3.38 to 7.18)	5.45 (3.79 to 7.52)	12.42 (10.89 to 13.99)	9.25 (7.18 to 10.94)	12.27 (10.27 to 14.14)	9.75 (7.36 to 12.51)
Global	30-34 years	7.17 (5.34 to 9.71)	7.85 (5.96 to 10.47)	9.63 (7.31 to 13.01)	11.14 (8.74 to 14.34)	24.15 (21.07 to 26.51)	17.82 (12.88 to 21.18)	19.74 (16.94 to 22.23)	14.52 (11.24 to 17.78)
Global	35-39 years	11.63 (7.57 to 17.12)	13.5 (9.77 to 18.69)	15.21 (9.97 to 21.88)	19.36 (14.12 to 25.97)	35.95 (31.41 to 39.54)	24.45 (19.05 to 28.9)	28.5 (24.82 to 32.19)	20.79 (16.58 to 25.36)
Global	40-44 years	21.25 (15.8 to 27.92)	26.29 (20.65 to 32.8)	25.57 (18.98 to 33.68)	33.25 (26.56 to 40.83)	52.94 (45.74 to 57.95)	36.39 (28.57 to 42.9)	39.56 (34.92 to 44.18)	27.71 (22.96 to 32.69)
Global	45-49 years	32.91 (21.51 to 47.36)	45.89 (32.96 to 62.36)	36.41 (23.74 to 52.46)	52.83 (38.59 to 70.98)	60.71 (53.41 to 66.26)	42.96 (34.85 to 50.13)	47.29 (41.85 to 52.48)	34.53 (28.85 to 41.19)
Global	50-54 years	48.87 (35.81 to 66.39)	85.46 (67.28 to 106.29)	47.09 (34.43 to 64.13)	81.05 (64.63 to 102.45)	82.99 (72.91 to 91.04)	62.64 (51.25 to 71.54)	59.61 (53.05 to 66.98)	46.28 (38.12 to 53.76)
Global	55-59 years	74.92 (47.93 to 110.59)	148.59 (108.04 to 194.5)	63.42 (40.84 to 92.3)	117.39 (86.62 to 152.99)	104.85 (91.79 to 115.67)	86 (72.74 to 97.81)	70.11 (62.02 to 79.66)	59.84 (49.8 to 69.42)
Global	60-64	137.04 (102.43 to	285.89 (232.84 to	101 (73 to 138.57)	185.64 (145.76 to	139.19 (124.08 to	124.5 (107.25 to	82.78 (74.66 to	73.72 (63.29 to

	years	182.81)	347.48)		230.13)	152.48)	140.51)	92.19)	84.15)
Global	65-69 years	255.88 (199.36 to 323.79)	474.95 (406.05 to 545.01)	165.92 (127.22 to 208.07)	271.19 (231.33 to 313.58)	171.79 (153 to 191.82)	158.31 (135.48 to 180.44)	99.57 (90.73 to 109.46)	90.72 (77.91 to 103.04)
Global	70-74 years	387.44 (286.24 to 512.74)	720.49 (608.96 to 849.69)	237.77 (177.78 to 315.38)	382.66 (320.76 to 451.18)	196.97 (175.63 to 215.46)	203.31 (177.75 to 226.91)	114.62 (103.95 to 127.36)	114.42 (99.5 to 129.98)
Global	75-79 years	529.07 (376.32 to 716.47)	942.53 (776.79 to 1132.77)	316.04 (224.18 to 435.34)	479.99 (392.3 to 577.89)	234.49 (212.68 to 255.93)	246.66 (216 to 275.24)	143.72 (131.46 to 155.92)	136.93 (119.22 to 154.24)
Global	80-84 years	697.64 (535.24 to 890.9)	1118.5 (952.15 to 1296.26)	419.24 (317.35 to 547.86)	531.29 (444.3 to 624.44)	273.08 (247.15 to 298.76)	318.45 (274.84 to 351.67)	178.43 (158.49 to 194.4)	177.72 (149.79 to 199.71)
Global	85-89 years	943.77 (704.48 to 1245.52)	1405 (1193.21 to 1653.56)	573.49 (428.31 to 754.45)	630.43 (527.77 to 751.43)	348.85 (312.05 to 387.68)	437.34 (370.3 to 482.9)	242.14 (209.04 to 266.47)	238.95 (190.2 to 272)
Global	90-94 years	1298.85 (843.15 to 1897.75)	1860.18 (1480.93 to 2327.76)	812.73 (535.56 to 1177.32)	805.49 (631.07 to 1026.7)	477.29 (416.08 to 527.26)	634.66 (534.69 to 700.89)	344.47 (282.95 to 383.33)	365.47 (284.13 to 414.43)
Global	95+ years	1906.2 (1087.44 to 3025.5)	2174.37 (1584.44 to 2876.47)	1149.86 (644.63 to 1823.47)	999.21 (703.93 to 1355.04)	615.79 (500.79 to 690.84)	810.18 (638.91 to 908.96)	493.21 (379.51 to 562.56)	610.45 (443.26 to 702.72)
High SDI	15-19 years	0.56 (0.52 to 0.59)	0.62 (0.57 to 0.67)	0.9 (0.85 to 0.95)	1.06 (0.98 to 1.16)	4.86 (4.6 to 5.1)	2.98 (2.78 to 3.22)	4.39 (4.15 to 4.66)	2.93 (2.67 to 3.25)
High SDI	20-24 years	6.68 (4.5 to 9.31)	7.85 (6.02 to 9.83)	9.15 (6.31 to 12.39)	11.74 (9.36 to 14.26)	14.77 (14.04 to 15.68)	10.1 (9.5 to 10.91)	12.83 (12.07 to 13.75)	8.81 (8.12 to 9.61)
High SDI	25-29 years	13.35 (8.72 to 19.24)	15.11 (11.43 to 19.8)	20.13 (13.7 to 28.63)	24.5 (19 to 30.81)	31.31 (29.89 to 32.98)	20.32 (19.1 to 21.78)	26.47 (24.88 to 28.21)	16.56 (15.12 to 18.34)
High SDI	30-34 years	25.84 (19.36 to 34.81)	32.45 (26.65 to 40.42)	36.93 (28.33 to 49.81)	51.28 (42.52 to 62.55)	57.8 (55.38 to 60.36)	32.17 (30.13 to 34.65)	41.96 (39.85 to 44.02)	26.68 (24.65 to 29.14)
High SDI	35-39 years	42.49 (27.29 to 62.8)	58.06 (44.95 to 75.1)	58.19 (37.62 to 85.41)	89.22 (69.06 to 113.33)	84.94 (81.83 to 88.48)	43.89 (41.37 to 47.44)	59.35 (56.59 to 62.61)	36.28 (33.39 to 39.31)
High	40-44	71.45 (52.31 to	114.64 (94.52 to	87.36 (63.92 to	150.36 (125.12 to	112.35 (108.09 to	60.54 (57.02 to	76.75 (73.04 to	47.65 (44.02 to

SDI	years	94.32)	137.7)	115.68)	179.42)	117.15)	65.02)	80.81)	51.63)
High SDI	45-49 years	112.56 (72.25 to 164.41)	197.02 (150.86 to 255.25)	123.28 (78.85 to 180.77)	227.99 (176.9 to 292.26)	137.69 (132.42 to 143.48)	78.8 (74.28 to 84.49)	91.65 (87.3 to 96.51)	59.64 (55.3 to 64.84)
High SDI	50-54 years	161.87 (116.75 to 222.18)	351.14 (284.97 to 423.26)	151.76 (110.03 to 207.36)	328.41 (270.02 to 398.55)	170.14 (163.47 to 177.36)	115.59 (108.73 to 123.74)	103.35 (98.46 to 108.34)	77 (71.83 to 84)
High SDI	55-59 years	239.87 (147.79 to 362.56)	577.07 (434.15 to 734.3)	194.64 (121.5 to 288.23)	447.9 (341.04 to 562.81)	216.18 (207.24 to 226.57)	166.88 (156.61 to 180.44)	115.39 (109.9 to 121.63)	94.1 (87.26 to 102.7)
High SDI	60-64 years	432 (317.93 to 581.95)	1062.68 (880.64 to 1256.45)	292.65 (206.5 to 406.84)	670.78 (540.87 to 806.65)	268.23 (257.38 to 280.52)	226.48 (211.77 to 246.09)	132.06 (125.14 to 139.59)	112.56 (102.93 to 123.99)
High SDI	65-69 years	772.55 (592.1 to 989.64)	1730.05 (1495.8 to 1966.08)	440.38 (332.77 to 559.13)	955.48 (830.3 to 1088.99)	314.29 (299.95 to 330.1)	289.26 (266.97 to 316.27)	150.56 (141.83 to 159.78)	131.98 (118.71 to 145.8)
High SDI	70-74 years	1165.53 (843.98 to 1562.3)	2307.07 (1969.2 to 2681.5)	622.55 (458.29 to 835.94)	1189.6 (1011.43 to 1376.68)	351.57 (333.79 to 373.16)	347.43 (317.88 to 382.11)	166.31 (154.38 to 178.42)	153.56 (135.71 to 171.45)
High SDI	75-79 years	1378.93 (963.43 to 1890.06)	2899.29 (2410.21 to 3420.86)	713.79 (495.88 to 998.31)	1421.09 (1176.1 to 1673.12)	379.27 (356.96 to 404.16)	435.7 (393.42 to 479.18)	191.73 (175.6 to 205.92)	188.59 (164.55 to 211.77)
High SDI	80-84 years	1705.03 (1292.03 to 2186.98)	3076.15 (2661.67 to 3519.46)	864.45 (645.22 to 1140.51)	1404.12 (1196.82 to 1619.49)	412.59 (380.91 to 443.81)	508.76 (446.65 to 567.88)	221.98 (193.72 to 241.4)	216.69 (176.05 to 249.12)
High SDI	85-89 years	2204.41 (1626.43 to 2924.65)	3453.73 (2955.79 to 4015.77)	1092.35 (807.38 to 1445.94)	1446.77 (1232.8 to 1688.11)	498.64 (448.96 to 544.73)	630.65 (541.63 to 699.39)	276.77 (232.77 to 304.87)	273.36 (211.25 to 312.16)
High SDI	90-94 years	2858.95 (1836.07 to 4201.75)	4099.65 (3299.83 to 5048.15)	1390.86 (908.93 to 2027.72)	1604.16 (1282.98 to 1995.04)	648.5 (563.55 to 715.12)	852.8 (718.28 to 945.51)	373.76 (300.56 to 419.91)	397.81 (302.67 to 457.05)
High SDI	95+ years	4020.75 (2278.18 to 6397.04)	4302.56 (3170.45 to 5603.61)	1860.76 (1036.8 to 2959.4)	1717.44 (1237.3 to 2283.54)	842.08 (681.06 to 964.4)	1028.93 (816.9 to 1153.79)	521.86 (398.1 to 601.73)	646.26 (460.75 to 750.74)
High-middle SDI	15-19 years	0.13 (0.11 to 0.15)	0.22 (0.18 to 0.27)	0.22 (0.19 to 0.25)	0.42 (0.35 to 0.5)	2.96 (2.36 to 3.46)	2.12 (1.71 to 2.56)	2.95 (2.37 to 3.44)	2.36 (1.86 to 2.88)

High-middle SDI	20-24 years	1.4 (0.85 to 2.13)	2.71 (1.55 to 4.16)	1.81 (1.19 to 2.64)	3.35 (2.08 to 4.85)	8.43 (7.15 to 10.27)	6.97 (5.83 to 8.59)	8.17 (6.97 to 9.54)	7.06 (5.86 to 8.6)
High-middle SDI	25-29 years	2.39 (1.5 to 3.53)	4.76 (2.7 to 7.51)	3.3 (2.23 to 4.65)	6.62 (4.09 to 9.92)	14.51 (12.84 to 17.24)	12 (10.01 to 14.29)	14.83 (13 to 16.57)	11.84 (9.93 to 14.06)
High-middle SDI	30-34 years	3.92 (2.87 to 5.31)	7.64 (4.99 to 10.96)	5.35 (4.05 to 7.04)	11.18 (7.84 to 15.61)	26.97 (23.75 to 30.87)	21.71 (18.08 to 25.75)	24.29 (21.3 to 27.06)	18.07 (15.09 to 21.65)
High-middle SDI	35-39 years	5.89 (4.07 to 8.37)	11.12 (6.6 to 17.2)	7.57 (5.38 to 10.46)	16.94 (10.8 to 24.9)	39.8 (34.52 to 45.66)	33.61 (28.22 to 39.38)	34.27 (30.06 to 38.23)	26.59 (22.75 to 31.35)
High-middle SDI	40-44 years	10.77 (8.34 to 13.81)	19.18 (13.4 to 26.2)	12.65 (9.81 to 16.18)	26.02 (19.16 to 34.68)	59.91 (51.21 to 68.32)	49.79 (41.83 to 58.82)	48 (42.75 to 53.04)	37.71 (32.19 to 43.51)
High-middle SDI	45-49 years	17.83 (12.86 to 24.11)	31.59 (19.68 to 46.87)	19.34 (13.81 to 26.01)	39.63 (25.97 to 57.02)	66.66 (59.18 to 74.47)	53.08 (45.6 to 62.54)	58.33 (51.49 to 65.27)	46.39 (39.07 to 54.42)
High-middle SDI	50-54 years	28.29 (21.99 to 36.25)	53.03 (38.6 to 72.12)	26.98 (20.76 to 35.04)	54.74 (40.33 to 73.73)	91.96 (82.15 to 102.84)	70.04 (59.89 to 82.98)	70.15 (62.71 to 78.54)	57.18 (47.15 to 67.09)
High-middle SDI	55-59 years	44.06 (32 to 59.12)	81.33 (52.75 to 117.44)	35.99 (25.92 to 48.93)	69.72 (46.91 to 99.31)	111.38 (97.84 to 124.94)	92.73 (79.7 to 109.49)	79.17 (70.08 to 89.05)	71.73 (59.1 to 85.48)
High-middle	60-64 years	71.06 (57.06 to 89.11)	125.3 (93.21 to 166.43)	53.3 (41.27 to 68.97)	91.2 (66.36 to 123.57)	143.96 (130.47 to 161.23)	133.67 (117.78 to 157.54)	88.99 (80.25 to 98.36)	93.01 (80.26 to 106.97)

SDI									
High-middle SDI	65-69 years	113.46 (95.31 to 133.64)	194.4 (154.1 to 240)	78.37 (64.64 to 93.84)	123.32 (95.38 to 153.01)	172.27 (153.49 to 198.97)	162.51 (139.51 to 198.38)	104.39 (94.91 to 115.19)	107.85 (90.87 to 124.17)
High-middle SDI	70-74 years	147.38 (120.05 to 179.79)	267.99 (205.51 to 346.03)	99.55 (80.53 to 124.26)	156.73 (118.3 to 205.63)	190.23 (169.49 to 220.34)	202.71 (175.59 to 243.01)	115.97 (104.98 to 128.97)	132.47 (112.38 to 152.9)
High-middle SDI	75-79 years	211.3 (165.91 to 266.16)	348.38 (255.53 to 464.08)	134.05 (103.45 to 174.77)	192.57 (138.24 to 266.17)	231.67 (208.51 to 259.31)	232.28 (201.13 to 272.08)	150.59 (139.17 to 163.32)	151.11 (126.54 to 174.68)
High-middle SDI	80-84 years	269.69 (221.44 to 326.81)	399.2 (304.15 to 516.22)	163.43 (132.45 to 203.29)	219.89 (164.65 to 293.02)	287.48 (258.72 to 325.3)	313.36 (269.48 to 360.85)	195.71 (176.43 to 213.48)	206.77 (174.48 to 233.32)
High-middle SDI	85-89 years	327.22 (263.84 to 407.47)	454.4 (341.46 to 597.33)	190.59 (152.72 to 238.6)	251.32 (185.94 to 335.53)	400.8 (353.61 to 465.41)	438.98 (367.47 to 508.97)	271.43 (234.78 to 298.67)	261.55 (207.72 to 301.25)
High-middle SDI	90-94 years	391.52 (287.53 to 528.54)	495.75 (325.82 to 729.84)	222.05 (162.32 to 300.41)	280.11 (183.68 to 412.25)	584.7 (508.68 to 655.7)	629.76 (524.62 to 708.22)	413.64 (346.77 to 460.29)	394.87 (309.09 to 452.15)
High-middle SDI	95+ years	446.82 (292.53 to 642.94)	557.76 (305.31 to 904.1)	251.13 (161.82 to 365.94)	324.02 (175.66 to 530.41)	754.38 (630.58 to 840.82)	874.16 (686.78 to 998.2)	672.41 (532.2 to 764.63)	684.51 (507.5 to 789.9)
Middle SDI	15-19 years	0.05 (0.03 to 0.06)	0.08 (0.05 to 0.1)	0.06 (0.03 to 0.07)	0.11 (0.07 to 0.15)	1.67 (1 to 2.13)	1.29 (0.87 to 1.67)	1.58 (0.93 to 2.01)	1.33 (0.79 to 1.85)
Middle SDI	20-24 years	1.34 (0.74 to 2.15)	1.74 (0.94 to 2.78)	1.48 (0.85 to 2.33)	1.91 (1.05 to 2.96)	5.62 (3.81 to 6.8)	5.41 (3.8 to 6.49)	5.29 (3.77 to 6.61)	5.13 (3.66 to 6.47)

Middle SDI	25-29 years	2.07 (1.13 to 3.3)	2.92 (1.51 to 4.86)	2.25 (1.25 to 3.55)	3.48 (1.88 to 5.62)	8.03 (5.83 to 9.66)	7.94 (5.58 to 9.74)	8.07 (5.95 to 10.12)	7.71 (5.6 to 9.96)
Middle SDI	30-34 years	3.34 (2.24 to 4.74)	4.82 (2.95 to 7.13)	3.58 (2.44 to 5.03)	6.35 (4.1 to 9.29)	14.64 (10.25 to 17.66)	14.13 (9.26 to 17.48)	11.99 (8.67 to 14.8)	10.87 (7.66 to 13.78)
Middle SDI	35-39 years	4.95 (3.07 to 7.49)	6.9 (3.74 to 11.27)	5.27 (3.38 to 7.82)	9.75 (5.64 to 15.27)	22.2 (15.12 to 26.87)	20.13 (13.51 to 24.93)	17.39 (13.1 to 22.17)	15.59 (11.42 to 19.71)
Middle SDI	40-44 years	7.91 (5.62 to 10.67)	11.55 (7.7 to 16.62)	8.26 (5.96 to 11.04)	15.08 (10.4 to 21.04)	33.95 (22.7 to 41.5)	30.65 (20.93 to 38.47)	24.43 (18.76 to 30.45)	21.31 (16 to 26.8)
Middle SDI	45-49 years	11.63 (7.46 to 16.74)	19.92 (11.35 to 30.35)	12.08 (7.86 to 17.51)	24.64 (14.88 to 37.01)	34.56 (24.77 to 41.84)	33.04 (23.15 to 41.84)	29.79 (23.21 to 37)	27.23 (20.29 to 34.66)
Middle SDI	50-54 years	18.05 (13.16 to 24.42)	35.54 (25.01 to 49.19)	16.77 (12.19 to 22.59)	36.72 (25.81 to 51.28)	50.71 (35.66 to 62.38)	47.63 (33.11 to 58.96)	41.87 (32.35 to 52.37)	37.61 (26.79 to 46.91)
Middle SDI	55-59 years	26.21 (17.19 to 37.3)	57.27 (36.06 to 83.96)	21.8 (14.41 to 31.18)	50.27 (32.88 to 73.12)	64.98 (46.03 to 79.31)	62.29 (44.59 to 78.05)	52.7 (40.7 to 66.43)	49.39 (35.45 to 62.46)
Middle SDI	60-64 years	40.25 (30.12 to 53.73)	80.97 (58.72 to 109.37)	32.19 (24.03 to 43.43)	60.65 (41.94 to 83.62)	89.6 (66.32 to 108.86)	91.56 (65.42 to 114.4)	61.85 (48.58 to 77.45)	58.5 (43.32 to 72.42)
Middle SDI	65-69 years	59.12 (47.29 to 72.76)	131.94 (103.87 to 163.15)	45.29 (36.29 to 55.7)	86.1 (65.01 to 108.13)	116.02 (83.15 to 144.73)	119.68 (83.99 to 150.65)	73.6 (59.81 to 91.73)	76.15 (55.54 to 93.14)
Middle SDI	70-74 years	79.75 (62.23 to 101.27)	178.78 (137.25 to 229.21)	59.33 (46.94 to 75.11)	107.86 (80.8 to 139.92)	143.36 (103.27 to 175.73)	153.14 (107.6 to 189.2)	92.04 (75.08 to 114.96)	99.51 (71.88 to 120.5)
Middle SDI	75-79 years	105.47 (79.8 to 138.88)	224.66 (163.98 to 297.59)	77.28 (58.7 to 100.73)	129.18 (91.41 to 179.4)	161.09 (120.24 to 195.54)	174.72 (125.26 to 210.87)	104.33 (85.28 to 128.29)	117.9 (89.03 to 141.31)
Middle SDI	80-84 years	126.48 (99.85 to 159.92)	243.45 (184.2 to 313.78)	92.95 (73.44 to 116.36)	139.89 (102.49 to 188.88)	188.91 (149.11 to 233.86)	221.61 (159.67 to 264.45)	127.16 (103.19 to 155.56)	152.59 (114.59 to 180.47)
Middle SDI	85-89 years	145.47 (113.43 to 187.11)	227.41 (172.53 to 299.89)	105.98 (82.53 to 136.73)	142.73 (105.05 to 191.73)	247.36 (197.1 to 311.98)	325.92 (234.9 to 380.31)	191.31 (156.31 to 225.02)	214.2 (157.74 to 254.65)

Middle SDI	90-94 years	159.98 (109.48 to 228.79)	208.21 (138.73 to 306.88)	106.77 (73.65 to 150.85)	143.87 (94.73 to 212.79)	341.16 (280 to 429.89)	474.91 (354.04 to 560.72)	259.19 (205.22 to 310.75)	328.12 (248.29 to 383.68)
Middle SDI	95+ years	172.5 (96.27 to 277.26)	201 (105.93 to 331.9)	91.57 (52.81 to 144.35)	146.33 (75.97 to 245.14)	410.93 (324.35 to 519.49)	547.69 (413.77 to 642.71)	317.56 (223.56 to 424.13)	541.35 (400.96 to 639.45)
Low-middle SDI	15-19 years	0.02 (0.01 to 0.02)	0.03 (0.02 to 0.04)	0.04 (0.02 to 0.05)	0.07 (0.04 to 0.11)	0.85 (0.53 to 1.19)	1.03 (0.58 to 1.42)	1.34 (0.76 to 1.93)	1.74 (0.89 to 2.85)
Low-middle SDI	20-24 years	0.65 (0.3 to 1.23)	0.78 (0.38 to 1.37)	0.69 (0.33 to 1.27)	0.84 (0.4 to 1.52)	2.78 (1.89 to 3.77)	3.39 (2.16 to 4.48)	4.45 (2.7 to 6.79)	5.76 (3.17 to 8.6)
Low-middle SDI	25-29 years	0.96 (0.48 to 1.66)	1.19 (0.57 to 2.06)	1.03 (0.51 to 1.74)	1.3 (0.64 to 2.23)	4.57 (3.17 to 6.19)	5.41 (3.53 to 7.41)	6.1 (3.85 to 9.23)	7.47 (4.63 to 10.89)
Low-middle SDI	30-34 years	1.5 (0.92 to 2.28)	1.79 (1.04 to 2.74)	1.58 (0.94 to 2.37)	1.98 (1.17 to 2.96)	8.6 (5.71 to 11.25)	11.76 (6.48 to 15.85)	8.19 (5.07 to 12.34)	10.4 (6.46 to 14.32)
Low-middle SDI	35-39 years	2.22 (1.27 to 3.54)	2.57 (1.36 to 4.28)	2.34 (1.33 to 3.75)	2.87 (1.56 to 4.65)	11.89 (8.09 to 15.54)	14.17 (9.2 to 18.99)	11.72 (7.78 to 17.34)	14.79 (9.81 to 20.01)
Low-middle SDI	40-44 years	3.56 (2.38 to 5.1)	3.92 (2.49 to 5.9)	3.59 (2.42 to 5.17)	4.07 (2.6 to 6.1)	17.24 (11.58 to 22.86)	21.74 (14.39 to 28.71)	15.24 (10.47 to 22.67)	17.87 (12.2 to 23.75)
Low-middle SDI	45-49 years	5.33 (3.23 to 8.08)	5.61 (3.12 to 8.74)	5.25 (3.23 to 7.9)	5.54 (3.12 to 8.56)	21.35 (14.44 to 28.46)	26.51 (17.49 to 34.76)	18.02 (12.35 to 26.9)	20.07 (14.35 to 27.18)
Low-	50-54	8.35 (5.76 to 11.68)	8.76 (5.74 to 12.78)	7.57 (5.2 to 10.62)	7.98 (5.21 to 11.63)	32.33 (21.48 to	39.56 (26.27 to	24.49 (17.39 to	26.91 (18.81 to

middle SDI	years					43.23)	51.17)	35.54)	35.23)
Low-middle SDI	55-59 years	12.07 (7.43 to 18.08)	13.09 (7.52 to 20.66)	10.15 (6.27 to 15.25)	11.39 (6.67 to 17.93)	38.87 (26.58 to 53.2)	50.16 (34.75 to 64.87)	29.4 (21.02 to 43.79)	35.13 (25.88 to 45.78)
Low-middle SDI	60-64 years	18.44 (13.06 to 25.81)	18.3 (12.08 to 26.83)	14.54 (10.1 to 20.7)	14.98 (9.66 to 22.26)	53.95 (36.93 to 72.76)	66.75 (46.82 to 86.33)	34.19 (24.91 to 49.27)	38.13 (28.13 to 50.35)
Low-middle SDI	65-69 years	28.29 (21.8 to 35.31)	24.47 (17.8 to 31.8)	20.83 (15.93 to 26.41)	19.17 (13.8 to 25.23)	63.66 (42.48 to 87.11)	79.04 (54.24 to 103.7)	40.98 (29.33 to 57.87)	47.35 (35.77 to 61.07)
Low-middle SDI	70-74 years	39.27 (30.12 to 50.18)	31.25 (22.45 to 42.27)	27.85 (21.03 to 35.97)	23.72 (16.95 to 32.36)	74 (50.19 to 100.23)	98.25 (71.29 to 130.33)	51.35 (36.97 to 71.52)	61.17 (47.64 to 78.94)
Low-middle SDI	75-79 years	52.69 (39.03 to 69)	37.54 (25.85 to 52.97)	38 (27.68 to 50.76)	27.97 (18.72 to 40.34)	89.92 (60.76 to 120.91)	119.37 (83.92 to 156.11)	63.22 (46.58 to 85.14)	74.64 (58.92 to 93.61)
Low-middle SDI	80-84 years	56.58 (43.68 to 71.38)	44.02 (31.62 to 59.87)	43.11 (32.61 to 54.84)	32.78 (22.68 to 45.11)	101.36 (67.08 to 140.04)	153.28 (114.47 to 199.32)	74.65 (52.62 to 100.32)	99.32 (80.03 to 121.87)
Low-middle SDI	85-89 years	61.06 (47.42 to 79.14)	49.21 (36.59 to 65.48)	51.33 (39.16 to 67.72)	38.61 (28.23 to 51.62)	116.05 (77.46 to 161.41)	185.53 (140.72 to 241.39)	105.71 (71.82 to 144.05)	146.81 (113.77 to 182.05)
Low-middle SDI	90-94 years	69.38 (47.29 to 98.9)	55.87 (37.62 to 80.83)	63.67 (43.16 to 91.5)	48.16 (32.17 to 70.55)	157.29 (104.52 to 223.34)	272.65 (209.14 to 355.02)	156.08 (105.84 to 209.1)	233.43 (176.6 to 284.32)

Low-middle SDI	95+ years	84.25 (45.57 to 138.38)	83.26 (45.63 to 137.31)	80.62 (43.44 to 130.49)	73.93 (38.29 to 119.32)	190.26 (124.77 to 273.47)	388.59 (278.83 to 491.54)	216.27 (140.08 to 302)	378.47 (266.03 to 475.77)
Low SDI	15-19 years	0.03 (0.02 to 0.05)	0.04 (0.02 to 0.06)	0.07 (0.03 to 0.09)	0.1 (0.05 to 0.17)	1.94 (1.08 to 2.67)	1.94 (1.02 to 2.85)	2.95 (1.5 to 4.18)	3.28 (1.65 to 5.6)
Low SDI	20-24 years	0.58 (0.24 to 1.12)	0.64 (0.27 to 1.2)	0.65 (0.27 to 1.24)	0.77 (0.33 to 1.46)	3.71 (1.94 to 5.08)	4.19 (2.11 to 6.23)	7.83 (4.04 to 11.52)	8.95 (4.42 to 14.1)
Low SDI	25-29 years	0.91 (0.4 to 1.65)	1 (0.41 to 1.82)	1 (0.43 to 1.74)	1.15 (0.5 to 2.06)	5.79 (3.19 to 7.75)	6.89 (3.55 to 10.33)	10.37 (5.61 to 15.35)	10.89 (5.75 to 17.7)
Low SDI	30-34 years	1.36 (0.73 to 2.13)	1.5 (0.78 to 2.4)	1.41 (0.73 to 2.19)	1.59 (0.81 to 2.55)	12.56 (6.4 to 16.79)	18.56 (7.25 to 27.95)	14.28 (7.44 to 20.2)	15.24 (7.8 to 23.07)
Low SDI	35-39 years	1.95 (0.97 to 3.28)	2.13 (1.05 to 3.71)	1.93 (0.93 to 3.32)	2.13 (1.05 to 3.66)	16.73 (8.91 to 22.13)	18.55 (9.82 to 27.11)	20.04 (10.92 to 29.57)	20.81 (11.54 to 32.28)
Low SDI	40-44 years	2.9 (1.74 to 4.37)	3.16 (1.88 to 4.88)	2.58 (1.45 to 4)	2.78 (1.57 to 4.34)	23.83 (12.59 to 31.79)	26.49 (13.99 to 39.2)	24.08 (12.98 to 35.15)	23.9 (13.09 to 35)
Low SDI	45-49 years	4.29 (2.35 to 6.72)	4.54 (2.46 to 7.3)	3.44 (1.82 to 5.55)	3.66 (1.94 to 5.91)	30.5 (16.12 to 40.63)	32.16 (16.45 to 47.35)	27.43 (14.82 to 40.33)	26.39 (14.43 to 40.2)
Low SDI	50-54 years	6.74 (4.17 to 9.74)	6.97 (4.29 to 10.15)	5.18 (3.1 to 7.64)	5.37 (3.15 to 7.97)	48.68 (25.41 to 67.85)	49.3 (25.2 to 70.48)	38.92 (21.54 to 55.39)	36.51 (18.91 to 53.96)
Low SDI	55-59 years	9.49 (5.12 to 14.84)	9.75 (5.26 to 15.27)	7.41 (4.13 to 11.64)	7.58 (4.22 to 11.97)	50.76 (26.21 to 69.16)	53.39 (27.43 to 75.5)	45.7 (25.51 to 67.81)	44.87 (25.44 to 66.74)
Low SDI	60-64 years	13.08 (8.04 to 19.35)	13.19 (8.11 to 19.54)	9.48 (5.75 to 14.24)	9.52 (5.69 to 14.58)	67.49 (34.15 to 91.07)	74.15 (35.68 to 106.3)	50.6 (27.52 to 72.38)	48.88 (26.74 to 75.33)
Low SDI	65-69 years	17.55 (12.11 to 23.24)	17.06 (11.9 to 22.81)	11.49 (7.91 to 15.65)	11.56 (7.8 to 15.7)	76.69 (38.28 to 105.01)	78.45 (38.77 to 112.87)	58.86 (34.5 to 84.46)	58.99 (33.79 to 85.26)
Low SDI	70-74	21.91 (15.2 to	20.4 (14.07 to	13.32 (9.07 to 18.6)	13.27 (8.75 to	85.03 (42.89 to	92.92 (46.96 to	64.24 (36.76 to	69.7 (40.47 to

	years	29.75)	28.13)		18.95)	115.49)	131.86)	90.95)	100.47)
Low SDI	75-79 years	25.54 (16.69 to 36.33)	24.33 (15.88 to 35.07)	14.99 (9.17 to 22.17)	15.31 (9.03 to 23.02)	95.61 (48.87 to 132.14)	109.99 (52.07 to 159.34)	70.44 (40.46 to 102.13)	77.56 (45.15 to 112.86)
Low SDI	80-84 years	26.53 (18.08 to 36.41)	27.18 (18.33 to 37.48)	16.16 (10.51 to 23.04)	17.49 (11 to 24.98)	99.42 (52.04 to 142.33)	132.45 (63.69 to 192.31)	72.44 (38.88 to 109.71)	91.44 (53.22 to 132.83)
Low SDI	85-89 years	27.55 (19.64 to 37.39)	28.97 (20.46 to 39.35)	18.25 (12.5 to 26.19)	19.89 (13.26 to 28.54)	97.47 (48.96 to 145.36)	141.41 (67.69 to 202.14)	89.69 (44 to 143.15)	120.67 (63.94 to 182.25)
Low SDI	90-94 years	28.46 (18.09 to 42.19)	30.76 (19.32 to 45.43)	20.14 (12.45 to 30.65)	22.76 (13.65 to 34.4)	122.06 (58.12 to 192.21)	186.72 (89.61 to 283.89)	105.09 (45.8 to 171.5)	157.42 (75.5 to 242.79)
Low SDI	95+ years	27.2 (13.61 to 45.32)	31.42 (15.51 to 53.06)	21.97 (11.01 to 37.82)	25.55 (12.85 to 42.52)	145.75 (68.23 to 237.27)	199.15 (95.63 to 307.59)	134.6 (54.06 to 240.66)	203.31 (88.5 to 339.31)

Total skin cancer includes malignant skin melanoma, non-melanoma skin cancer (basal-cell carcinoma) and non-melanoma skin cancer (squamous-cell carcinoma). DALYs, disability-adjusted life-years; SDI, socio-demographic index; UI, uncertainty interval.

TABLE S24 Age-standardized rates of incidence and DALYs of malignant skin melanoma for global and SDI regions, by year (1990, 2021), age group and sex, with age-standardized rates and 95% uncertainty intervals.

Location name	Age group	Incidence				DALYs			
		Male		Female		Male		Female	
		Age-standardized rate (95% UI), 1990	Age-standardized rate (95% UI), 2021	Age-standardized rate (95% UI), 1990	Age-standardized rate (95% UI), 2021	Age-standardized rate (95% UI), 1990	Age-standardized rate (95% UI), 2021	Age-standardized rate (95% UI), 1990	Age-standardized rate (95% UI), 2021
Global	15-19 years	0.12 (0.1 to 0.13)	0.13 (0.11 to 0.14)	0.19 (0.17 to 0.21)	0.23 (0.18 to 0.27)	2.16 (1.63 to 2.51)	1.6 (1.12 to 2.05)	2.27 (1.62 to 2.74)	2.12 (1.32 to 3.12)

Global	20-24 years	0.32 (0.3 to 0.34)	0.35 (0.31 to 0.39)	0.5 (0.46 to 0.54)	0.61 (0.51 to 0.7)	4.49 (3.76 to 4.96)	3.52 (2.68 to 4.31)	4.95 (3.76 to 5.85)	4.99 (3.14 to 7)
Global	25-29 years	0.88 (0.83 to 0.93)	0.85 (0.76 to 0.92)	1.4 (1.32 to 1.49)	1.31 (1.16 to 1.46)	9.03 (8.04 to 9.86)	6.36 (5.04 to 7.6)	9.5 (7.92 to 10.72)	7.36 (5.34 to 9.76)
Global	30-34 years	1.64 (1.55 to 1.73)	1.41 (1.24 to 1.55)	2.26 (2.14 to 2.39)	1.99 (1.77 to 2.17)	18.33 (16.24 to 19.85)	12 (9.45 to 14.09)	16.03 (13.74 to 17.77)	11.16 (8.33 to 13.9)
Global	35-39 years	2.34 (2.21 to 2.46)	1.95 (1.72 to 2.16)	2.87 (2.72 to 3.02)	2.63 (2.33 to 2.91)	27.89 (24.46 to 30.29)	18.06 (14.36 to 21.45)	22.72 (19.73 to 25.23)	15.63 (12.04 to 19.57)
Global	40-44 years	3.41 (3.22 to 3.58)	2.78 (2.46 to 3.04)	3.91 (3.69 to 4.11)	3.37 (3.03 to 3.69)	40.23 (35.24 to 43.25)	25.88 (20.82 to 30.45)	31.28 (27.61 to 34.24)	20.35 (16.43 to 24.46)
Global	45-49 years	4.06 (3.87 to 4.27)	3.48 (3.13 to 3.8)	4.39 (4.17 to 4.61)	4.08 (3.7 to 4.45)	45.13 (40.12 to 48.59)	29.6 (24.64 to 34.55)	36.04 (31.81 to 39.52)	24.08 (19.82 to 29.17)
Global	50-54 years	5.11 (4.85 to 5.4)	5.16 (4.67 to 5.59)	4.87 (4.59 to 5.14)	5.08 (4.58 to 5.54)	59.15 (52.55 to 64.23)	41.62 (35.02 to 47.22)	43.52 (38.57 to 48.33)	30.76 (24.92 to 35.9)
Global	55-59 years	6.83 (6.43 to 7.19)	7.81 (7.13 to 8.44)	5.62 (5.28 to 5.92)	6.5 (5.95 to 7.03)	71.3 (62.79 to 78.3)	55.21 (46.99 to 62.98)	48.96 (43.07 to 54.64)	37.87 (31.13 to 44.02)
Global	60-64 years	8.99 (8.57 to 9.4)	11.27 (10.56 to 12.03)	6.83 (6.43 to 7.22)	8.45 (7.79 to 9.11)	81.98 (74.93 to 87.37)	68.61 (61.3 to 75.41)	55.43 (50.08 to 61.37)	45.98 (39.17 to 52.43)
Global	65-69 years	12.54 (11.98 to 13.15)	15.76 (14.76 to 16.82)	9.1 (8.56 to 9.56)	10.46 (9.57 to 11.27)	96.2 (88.67 to 103.6)	82.22 (73.38 to 90.64)	64.7 (59.38 to 70.32)	52.27 (44.73 to 59.16)
Global	70-74 years	15.82 (15.01 to 16.62)	23.07 (21.53 to 24.66)	10.76 (10.02 to 11.42)	14.02 (12.73 to 15.23)	102.47 (94.58 to 109.35)	102.51 (93.26 to 111.29)	68.38 (62.11 to 75.27)	62.63 (54.68 to 71.22)
Global	75-79 years	19.12 (17.97 to 20.12)	29.34 (27.06 to 31.53)	13.31 (12.19 to 14.12)	16.87 (14.63 to 18.49)	115.85 (107.67 to 122.93)	119.2 (108.73 to 130.37)	82.2 (74.87 to 88.37)	71.91 (62.23 to 81.21)
Global	80-84 years	21.07 (19.51 to 22.37)	38.48 (33.18 to 42.03)	15.84 (13.79 to 17.2)	20.96 (17.12 to 23.53)	118.05 (108.99 to 126.12)	144.17 (127.65 to 157.27)	89.61 (79.17 to 97.58)	86.1 (72.81 to 97.96)

Global	85-89 years	26.97 (23.92 to 28.94)	53.79 (45.37 to 59.31)	19.54 (15.86 to 21.56)	27.55 (20.87 to 31.49)	128.84 (115.93 to 138.7)	175.32 (149.53 to 192.24)	98.34 (82.97 to 108.01)	99.08 (77.53 to 113.75)
Global	90-94 years	27.41 (23.15 to 29.77)	56.49 (45.42 to 63)	20.28 (15.89 to 22.74)	28.82 (21.5 to 33.07)	153.39 (132.63 to 165.71)	219.29 (181.65 to 242.12)	119.09 (95.12 to 132.44)	126.86 (96.24 to 144.25)
Global	95+ years	26.73 (20.95 to 29.96)	51.86 (40.18 to 59.12)	22.75 (16.99 to 26.13)	35.65 (25.27 to 41.77)	159.79 (127.84 to 177.22)	219.4 (170.93 to 246.4)	140.88 (106.16 to 161.35)	165.58 (118.21 to 191.28)
High SDI	15-19 years	0.56 (0.52 to 0.59)	0.62 (0.57 to 0.67)	0.9 (0.85 to 0.95)	1.06 (0.98 to 1.16)	4.86 (4.6 to 5.1)	2.98 (2.78 to 3.22)	4.39 (4.15 to 4.66)	2.93 (2.67 to 3.25)
High SDI	20-24 years	1.59 (1.49 to 1.69)	1.88 (1.74 to 2.01)	2.47 (2.31 to 2.64)	3.01 (2.8 to 3.23)	12.85 (12.25 to 13.57)	8.69 (8.18 to 9.31)	11.64 (10.97 to 12.45)	7.93 (7.3 to 8.64)
High SDI	25-29 years	3.96 (3.73 to 4.2)	4.34 (4.02 to 4.66)	6.37 (5.99 to 6.76)	6.55 (6.13 to 7.01)	26.6 (25.37 to 27.98)	17.39 (16.33 to 18.62)	24.83 (23.36 to 26.41)	15.22 (13.89 to 16.87)
High SDI	30-34 years	6.71 (6.36 to 7.1)	6.27 (5.82 to 6.77)	9.02 (8.58 to 9.49)	9.45 (8.87 to 10.07)	49.02 (46.96 to 51.07)	27.74 (25.93 to 29.84)	39.69 (37.75 to 41.55)	24.85 (22.96 to 27.1)
High SDI	35-39 years	9.34 (8.85 to 9.84)	7.81 (7.34 to 8.38)	11.04 (10.53 to 11.63)	11.26 (10.57 to 12.06)	74.12 (71.45 to 77.05)	38.35 (36.17 to 41.27)	55.92 (53.4 to 58.82)	33.44 (30.82 to 36.09)
High SDI	40-44 years	11.98 (11.43 to 12.58)	10.3 (9.6 to 11.02)	13.21 (12.53 to 13.92)	13.25 (12.43 to 14.19)	96.76 (93.22 to 100.64)	51.68 (48.82 to 55.01)	71.61 (68.26 to 75.13)	43.01 (39.85 to 46.3)
High SDI	45-49 years	14.65 (13.96 to 15.43)	13.22 (12.33 to 14.23)	14.69 (14 to 15.41)	15.29 (14.41 to 16.28)	115.64 (111.36 to 120.3)	65.1 (61.68 to 69.23)	83.38 (79.65 to 87.28)	51.91 (48.49 to 55.79)
High SDI	50-54 years	17.31 (16.43 to 18.33)	18.76 (17.57 to 20.06)	15.22 (14.45 to 16.16)	18.2 (17.02 to 19.59)	137.15 (131.73 to 142.69)	91.1 (86.04 to 96.61)	90.61 (86.51 to 94.42)	64.91 (61.08 to 69.75)
High SDI	55-59 years	22.6 (21.5 to 23.84)	27.63 (25.95 to 29.54)	16.69 (15.84 to 17.52)	21.78 (20.48 to 23.09)	166.39 (159.76 to 173.57)	126.09 (119.39 to 134.7)	99.08 (94.73 to 103.52)	76.6 (71.9 to 81.83)
High SDI	60-64 years	28.72 (27.42 to 30.12)	37.54 (35.1 to 39.99)	18.93 (17.88 to 20.06)	25.2 (23.61 to 27.13)	194.67 (187.23 to 202.76)	158.57 (150.09 to 169.1)	108.87 (103.55 to 113.92)	87.44 (81.36 to 93.69)

High SDI	65-69 years	37.41 (35.62 to 39.3)	52.66 (49.45 to 56.19)	22.75 (21.34 to 24)	30.29 (27.94 to 32.59)	219.73 (210.95 to 228.75)	196.7 (185.28 to 209.43)	120.06 (113.83 to 126.2)	98.19 (90.28 to 105.01)
High SDI	70-74 years	46.82 (44.49 to 49.36)	67.88 (62.78 to 72.31)	26.37 (24.52 to 28.04)	36.11 (32.5 to 38.71)	235.18 (225.69 to 245.7)	227.64 (213.02 to 243.58)	125.64 (117.51 to 132.85)	110.15 (99.7 to 118.82)
High SDI	75-79 years	48.3 (45.31 to 50.91)	84.81 (77.49 to 90.73)	27.54 (25.08 to 29.36)	42.32 (36.41 to 46.41)	232.85 (221.3 to 243.54)	273.73 (252.1 to 292.64)	133.37 (122.38 to 141.39)	129.06 (114.07 to 140.05)
High SDI	80-84 years	48.89 (44.99 to 52.01)	98.99 (84.64 to 109.21)	29.77 (25.59 to 32.51)	45.85 (36.51 to 52.05)	221.01 (204.46 to 234.85)	301.53 (264.21 to 330.26)	137.06 (118.82 to 147.66)	139.91 (112.86 to 158.24)
High SDI	85-89 years	58.8 (51.96 to 63.38)	120.57 (99.77 to 133.95)	33.69 (26.94 to 37.35)	53.92 (39.31 to 61.74)	227.14 (203.35 to 244.07)	330.03 (280.6 to 364.34)	141.66 (116.25 to 155.92)	154.01 (117.15 to 175.13)
High SDI	90-94 years	54.14 (45.37 to 59.06)	112.29 (89.82 to 126.55)	30.81 (23.79 to 34.69)	48.84 (35.42 to 56.53)	253.27 (215.54 to 273.06)	375.15 (306.18 to 417.5)	158.38 (122.75 to 177.09)	179.51 (133.3 to 205.47)
High SDI	95+ years	49.63 (38.53 to 55.97)	91.66 (71.53 to 104.75)	32.52 (24.06 to 37.39)	53 (37.28 to 62.52)	249.85 (194.26 to 278.85)	334.06 (259.08 to 374.54)	179.29 (134.15 to 205.45)	214.19 (151.03 to 248.04)
High-middle SDI	15-19 years	0.13 (0.11 to 0.15)	0.22 (0.18 to 0.27)	0.22 (0.19 to 0.25)	0.42 (0.35 to 0.5)	2.96 (2.36 to 3.46)	2.12 (1.71 to 2.56)	2.95 (2.37 to 3.44)	2.36 (1.86 to 2.88)
High-middle SDI	20-24 years	0.29 (0.25 to 0.33)	0.51 (0.43 to 0.6)	0.49 (0.43 to 0.56)	0.94 (0.79 to 1.11)	5.58 (4.72 to 6.2)	4.56 (3.86 to 5.37)	5.59 (4.76 to 6.41)	5.06 (4.19 to 6.22)
High-middle SDI	25-29 years	0.7 (0.62 to 0.81)	1.14 (0.96 to 1.31)	1.2 (1.07 to 1.35)	1.95 (1.68 to 2.24)	10.82 (9.62 to 11.86)	8.73 (7.32 to 9.88)	11.3 (9.95 to 12.43)	9.07 (7.68 to 10.67)
High-middle SDI	30-34 years	1.15 (1.02 to 1.29)	1.82 (1.5 to 2.15)	1.91 (1.71 to 2.13)	2.63 (2.26 to 3.01)	20.92 (18.39 to 23)	16.79 (13.99 to 19.3)	19.55 (17.15 to 21.43)	14.2 (11.95 to 16.88)

High-middle SDI	35-39 years	1.56 (1.41 to 1.73)	2.56 (2.15 to 2.95)	2.37 (2.13 to 2.62)	3.6 (3.11 to 4.05)	30.46 (26.26 to 33.79)	26.01 (21.95 to 29.71)	27.32 (23.94 to 30.13)	20.86 (17.9 to 24.5)
High-middle SDI	40-44 years	2.34 (2.06 to 2.6)	3.72 (3.07 to 4.26)	3.27 (2.93 to 3.6)	4.87 (4.26 to 5.44)	44.36 (37.67 to 48.97)	37.37 (31.7 to 42.61)	37.92 (33.9 to 41.24)	29.28 (25.17 to 33.42)
High-middle SDI	45-49 years	2.83 (2.53 to 3.16)	4.24 (3.61 to 4.9)	3.82 (3.38 to 4.27)	5.73 (4.93 to 6.42)	49.13 (43.43 to 53.98)	40 (34.6 to 45.35)	44.15 (38.96 to 48.9)	34.17 (29.15 to 39.51)
High-middle SDI	50-54 years	3.77 (3.39 to 4.2)	5.52 (4.67 to 6.41)	4.26 (3.82 to 4.7)	6.26 (5.33 to 7.02)	64.37 (57.15 to 71.23)	49.44 (42.16 to 56.48)	52.05 (46.53 to 57.66)	40.11 (33.53 to 46.26)
High-middle SDI	55-59 years	4.77 (4.26 to 5.33)	7.45 (6.29 to 8.69)	4.71 (4.25 to 5.22)	7.4 (6.43 to 8.4)	74.23 (64.36 to 83.29)	62.84 (53.65 to 71.99)	55.58 (49.07 to 61.79)	47.95 (40.04 to 56.33)
High-middle SDI	60-64 years	5.71 (5.2 to 6.27)	9.58 (8.35 to 10.88)	5.25 (4.72 to 5.77)	9.44 (8.24 to 10.41)	80.31 (73.71 to 86.72)	76.08 (68.13 to 83.75)	59.1 (53.31 to 64.62)	60.85 (53.06 to 68.73)
High-middle SDI	65-69 years	7.11 (6.39 to 7.92)	11.94 (10.3 to 13.45)	6.59 (5.96 to 7.24)	10.92 (9.28 to 12.19)	86.86 (79.23 to 94.8)	84.41 (74.48 to 92.08)	65.79 (59.97 to 71.68)	65.69 (56.03 to 73.85)
High-middle SDI	70-74 years	7.73 (6.93 to 8.49)	16.76 (14.48 to 19.15)	7.15 (6.47 to 7.92)	13.67 (11.73 to 15.64)	83.72 (75.57 to 92.34)	102.28 (90.96 to 112.72)	65.25 (59.42 to 71.63)	76.14 (65.23 to 86.73)
High-middle	75-79 years	10.26 (9.29 to 11.33)	19.44 (16.59 to 22.09)	9.19 (8.3 to 10)	15.29 (12.7 to 17.93)	98.9 (89.45 to 107.55)	105.63 (93.76 to 116.84)	78.87 (72.3 to 85.82)	80.74 (67.59 to 92.9)

SDI									
High-middle SDI	80-84 years	11.84 (10.46 to 13.12)	24.48 (20.57 to 28.19)	10.47 (9.36 to 11.66)	19.55 (16.14 to 22.59)	105.56 (94.63 to 116.57)	128.21 (113.24 to 140.92)	83.18 (74.82 to 91.86)	100.24 (84.82 to 113.56)
High-middle SDI	85-89 years	14.9 (12.87 to 16.66)	33.81 (27.63 to 39.65)	11.79 (10.05 to 13.21)	22.75 (17.54 to 27.04)	118.16 (103.98 to 131.85)	149.57 (127.06 to 167.37)	84.72 (72.31 to 94.46)	101.16 (80.16 to 117.22)
High-middle SDI	90-94 years	16.77 (14.43 to 18.79)	32.53 (26.43 to 38.08)	12.68 (10.27 to 14.33)	22.04 (17.01 to 25.98)	146.64 (126.69 to 162.6)	177.47 (149.41 to 200.66)	105.09 (87.52 to 117.59)	122.74 (94.8 to 140.68)
High-middle SDI	95+ years	16.49 (13.57 to 18.65)	33.46 (25.43 to 39.75)	15.23 (11.57 to 17.65)	28.43 (20.78 to 34.6)	154.58 (129.09 to 173.46)	199.4 (155 to 230.72)	136.76 (107.23 to 156.94)	166.23 (122.44 to 195.19)
Middle SDI	15-19 years	0.05 (0.03 to 0.06)	0.08 (0.05 to 0.1)	0.06 (0.03 to 0.07)	0.11 (0.07 to 0.15)	1.67 (1 to 2.13)	1.29 (0.87 to 1.67)	1.58 (0.93 to 2.01)	1.33 (0.79 to 1.85)
Middle SDI	20-24 years	0.07 (0.04 to 0.08)	0.15 (0.1 to 0.18)	0.1 (0.06 to 0.12)	0.24 (0.15 to 0.3)	2.54 (1.63 to 3.07)	2.58 (1.9 to 3.15)	2.65 (1.59 to 3.37)	2.77 (1.72 to 3.68)
Middle SDI	25-29 years	0.13 (0.09 to 0.15)	0.27 (0.19 to 0.34)	0.2 (0.13 to 0.24)	0.43 (0.29 to 0.56)	3.98 (2.8 to 4.78)	3.94 (2.85 to 4.88)	4.31 (2.88 to 5.54)	4.24 (2.88 to 5.79)
Middle SDI	30-34 years	0.27 (0.18 to 0.32)	0.51 (0.34 to 0.64)	0.29 (0.19 to 0.35)	0.59 (0.37 to 0.75)	8.71 (5.92 to 10.6)	8.04 (5.49 to 9.98)	6.95 (4.54 to 8.79)	6.36 (4.03 to 8.27)
Middle SDI	35-39 years	0.4 (0.26 to 0.49)	0.71 (0.48 to 0.9)	0.38 (0.26 to 0.48)	0.78 (0.51 to 1.03)	12.98 (8.31 to 16)	11.77 (8.09 to 14.71)	9.52 (6.44 to 12.62)	8.94 (5.97 to 11.96)
Middle SDI	40-44 years	0.6 (0.4 to 0.73)	0.98 (0.67 to 1.3)	0.49 (0.34 to 0.62)	0.94 (0.62 to 1.25)	19.4 (12.69 to 23.72)	17.27 (12.11 to 22.2)	12.6 (8.72 to 16.1)	11.77 (8.02 to 15.57)
Middle	45-49	0.62 (0.44 to 0.74)	1 (0.7 to 1.29)	0.58 (0.4 to 0.73)	1.07 (0.7 to 1.43)	18.35 (12.77 to	16.98 (12.07 to	13.99 (9.73 to	13.43 (9.21 to

SDI	years					22.24)	21.95)	18.26)	17.94)
Middle SDI	50-54 years	0.99 (0.68 to 1.2)	1.48 (1.04 to 1.9)	0.81 (0.57 to 1.03)	1.32 (0.84 to 1.71)	27.31 (18.33 to 33.57)	24.4 (17.42 to 30.55)	19.15 (13.38 to 24.96)	17.4 (11.11 to 22.5)
Middle SDI	55-59 years	1.33 (0.89 to 1.63)	2.01 (1.4 to 2.6)	1.02 (0.7 to 1.33)	1.67 (1.1 to 2.24)	33.2 (22.25 to 41.11)	30.93 (22.05 to 40.28)	22.76 (15.46 to 30.1)	21.65 (14.09 to 28.77)
Middle SDI	60-64 years	1.33 (0.96 to 1.59)	2.21 (1.64 to 2.82)	1.13 (0.81 to 1.49)	1.96 (1.28 to 2.55)	28.58 (20.93 to 34.88)	31 (23.76 to 38.77)	23.18 (16.58 to 31.07)	23.96 (16.03 to 30.4)
Middle SDI	65-69 years	1.75 (1.28 to 2.14)	2.83 (2.04 to 3.73)	1.41 (1.02 to 1.87)	2.41 (1.53 to 3.08)	33 (24.42 to 41.39)	34.73 (25.84 to 44.52)	25.53 (18.8 to 34.64)	27.02 (17.21 to 34.17)
Middle SDI	70-74 years	2.28 (1.61 to 2.71)	3.71 (2.7 to 4.87)	1.81 (1.29 to 2.35)	3.13 (2 to 3.98)	37.52 (27.26 to 46.19)	40.5 (30.44 to 50.6)	29.55 (21.38 to 39.75)	32.46 (20.62 to 41.16)
Middle SDI	75-79 years	2.8 (2.07 to 3.3)	4.58 (3.33 to 5.88)	2.31 (1.66 to 2.97)	3.99 (2.62 to 4.93)	41.86 (31.02 to 49.83)	44.89 (34 to 55.56)	34.73 (25.2 to 46.36)	39.32 (25.99 to 48.97)
Middle SDI	80-84 years	3.35 (2.57 to 4.19)	5.91 (4.32 to 7.24)	2.92 (2.16 to 3.77)	4.89 (3.2 to 5.97)	43.81 (33.88 to 54.67)	51.48 (39.02 to 60.72)	38.07 (28.51 to 51.06)	44.16 (29.8 to 54.37)
Middle SDI	85-89 years	5.18 (3.76 to 6.04)	9.05 (6.41 to 11.02)	4.37 (3.11 to 5.43)	6.65 (4.32 to 8.22)	56.5 (42.45 to 67.97)	66.06 (49.4 to 78.29)	47.95 (34.84 to 61.67)	51.76 (34.94 to 63.75)
Middle SDI	90-94 years	6.53 (4.6 to 7.7)	9.97 (7.22 to 11.95)	5.11 (3.53 to 6.21)	7.35 (5.02 to 8.81)	71.09 (52.78 to 83.72)	82.33 (63.31 to 96.81)	55.72 (39.91 to 70.42)	64.87 (46.08 to 78.1)
Middle SDI	95+ years	7.34 (5.51 to 8.64)	9.79 (7.15 to 11.89)	4.85 (3.19 to 6.54)	8.74 (6.23 to 10.57)	85.1 (65.38 to 100.01)	90.41 (67.64 to 106.66)	54.12 (37.27 to 76.71)	81.84 (59.56 to 99.1)
Low-middle SDI	15-19 years	0.02 (0.01 to 0.02)	0.03 (0.02 to 0.04)	0.04 (0.02 to 0.05)	0.07 (0.04 to 0.11)	0.85 (0.53 to 1.19)	1.03 (0.58 to 1.42)	1.34 (0.76 to 1.93)	1.74 (0.89 to 2.85)
Low-middle	20-24 years	0.03 (0.02 to 0.05)	0.06 (0.04 to 0.08)	0.08 (0.05 to 0.12)	0.18 (0.09 to 0.28)	1.67 (1.08 to 2.28)	2.05 (1.2 to 2.85)	3.08 (1.69 to 4.46)	4.27 (1.97 to 6.76)

SDI									
Low-middle SDI	25-29 years	0.07 (0.05 to 0.09)	0.12 (0.07 to 0.16)	0.13 (0.08 to 0.19)	0.27 (0.15 to 0.4)	2.82 (1.87 to 3.82)	3.27 (1.95 to 4.66)	4.14 (2.44 to 5.9)	5.34 (2.91 to 8.31)
Low-middle SDI	30-34 years	0.13 (0.09 to 0.17)	0.22 (0.13 to 0.29)	0.18 (0.1 to 0.26)	0.37 (0.2 to 0.5)	5.35 (3.57 to 7.13)	6.3 (3.69 to 8.64)	5.6 (3.15 to 8.08)	7.42 (4 to 10.77)
Low-middle SDI	35-39 years	0.22 (0.14 to 0.28)	0.33 (0.2 to 0.45)	0.25 (0.15 to 0.35)	0.47 (0.27 to 0.65)	8.03 (5.2 to 10.46)	9.37 (5.63 to 13.08)	7.52 (4.48 to 10.75)	9.87 (5.63 to 14.15)
Low-middle SDI	40-44 years	0.31 (0.2 to 0.4)	0.48 (0.31 to 0.64)	0.31 (0.2 to 0.45)	0.54 (0.32 to 0.73)	11.01 (7.28 to 14.51)	13.45 (8.49 to 18.44)	9.49 (5.94 to 13.74)	11.24 (6.64 to 15.86)
Low-middle SDI	45-49 years	0.37 (0.24 to 0.49)	0.56 (0.36 to 0.75)	0.38 (0.24 to 0.56)	0.61 (0.38 to 0.83)	12.42 (8.05 to 16.47)	14.96 (9.43 to 20.41)	10.93 (6.79 to 16.49)	12.2 (7.79 to 17.81)
Low-middle SDI	50-54 years	0.59 (0.37 to 0.78)	0.87 (0.56 to 1.14)	0.53 (0.34 to 0.75)	0.79 (0.48 to 1.06)	18.39 (11.39 to 24.38)	21.58 (13.61 to 28.69)	14.2 (9.13 to 20.7)	15.43 (9.45 to 21.59)
Low-middle SDI	55-59 years	0.71 (0.46 to 0.96)	1.08 (0.7 to 1.39)	0.63 (0.4 to 0.95)	1 (0.63 to 1.35)	20.18 (12.95 to 27.58)	24.15 (15.48 to 32.05)	15.69 (10.15 to 24.31)	18.24 (11.66 to 25.69)
Low-middle SDI	60-64 years	0.91 (0.57 to 1.21)	1.33 (0.86 to 1.73)	0.77 (0.51 to 1.13)	1.12 (0.69 to 1.6)	23.03 (14.48 to 31.04)	26.58 (17.17 to 35.32)	17.75 (11.58 to 26.39)	19.43 (11.96 to 28.67)
Low-	65-69	1.2 (0.75 to 1.58)	1.74 (1.09 to 2.27)	1 (0.65 to 1.42)	1.41 (0.9 to 1.97)	26.5 (16.53 to	30.24 (19.5 to	20.19 (13.37 to	22.16 (14.33 to

middle SDI	years					35.18)	40.82)	29.43)	31.95)
Low-middle SDI	70-74 years	1.45 (0.93 to 1.83)	2.16 (1.38 to 2.8)	1.22 (0.79 to 1.68)	1.67 (1.06 to 2.36)	27.62 (17.6 to 35.37)	32.93 (21.53 to 43.86)	21.87 (14.49 to 31.1)	24.02 (15.68 to 35.73)
Low-middle SDI	75-79 years	1.75 (1.15 to 2.18)	2.54 (1.61 to 3.21)	1.47 (0.99 to 2.07)	1.92 (1.25 to 2.66)	28.88 (18.96 to 36.17)	34.24 (22.18 to 43.72)	23.14 (15.75 to 33.94)	24.91 (16.34 to 35.87)
Low-middle SDI	80-84 years	1.93 (1.27 to 2.44)	2.99 (1.95 to 3.72)	1.68 (1.12 to 2.4)	2.27 (1.51 to 3.05)	27.26 (17.94 to 34.6)	35.11 (23.25 to 44.25)	22.95 (15.53 to 33.89)	25.97 (17.52 to 36.7)
Low-middle SDI	85-89 years	2.18 (1.43 to 2.76)	3.63 (2.35 to 4.46)	2.07 (1.38 to 2.99)	2.71 (1.82 to 3.77)	26.15 (17.26 to 33.24)	36.09 (24.51 to 44.86)	24.34 (16.59 to 35.7)	26.93 (18.52 to 39.19)
Low-middle SDI	90-94 years	2.64 (1.72 to 3.25)	4.19 (2.87 to 5.18)	2.36 (1.62 to 3.23)	3.06 (2.12 to 3.86)	31.14 (20.89 to 38.44)	43.64 (30.89 to 54.44)	27.59 (19.1 to 38.17)	32.04 (22.62 to 41.45)
Low-middle SDI	95+ years	2.57 (1.81 to 3.25)	4.4 (3.14 to 5.36)	2.77 (1.89 to 3.73)	3.75 (2.53 to 4.76)	31.32 (22.26 to 39.42)	47.8 (35.16 to 57.6)	33.13 (22.78 to 45.27)	40.08 (27.45 to 51.32)
Low SDI	15-19 years	0.03 (0.02 to 0.05)	0.04 (0.02 to 0.06)	0.07 (0.03 to 0.09)	0.1 (0.05 to 0.17)	1.94 (1.08 to 2.67)	1.94 (1.02 to 2.85)	2.95 (1.5 to 4.18)	3.28 (1.65 to 5.6)
Low SDI	20-24 years	0.06 (0.03 to 0.08)	0.08 (0.04 to 0.12)	0.16 (0.08 to 0.23)	0.26 (0.12 to 0.42)	3.17 (1.72 to 4.26)	3.47 (1.85 to 5.07)	7.01 (3.53 to 10.18)	8.02 (3.83 to 12.82)
Low SDI	25-29 years	0.1 (0.06 to 0.14)	0.15 (0.08 to 0.22)	0.25 (0.13 to 0.36)	0.38 (0.19 to 0.61)	4.77 (2.75 to 6.23)	5.46 (3.02 to 8.12)	9.27 (4.84 to 13.53)	9.73 (5 to 16.16)

Low SDI	30-34 years	0.2 (0.11 to 0.25)	0.27 (0.15 to 0.4)	0.34 (0.17 to 0.48)	0.5 (0.24 to 0.77)	8.67 (4.87 to 11.21)	9.79 (5.53 to 14.3)	12.57 (6.31 to 17.59)	13.33 (6.57 to 20.52)
Low SDI	35-39 years	0.36 (0.2 to 0.47)	0.45 (0.25 to 0.66)	0.48 (0.25 to 0.72)	0.65 (0.34 to 1.01)	14.56 (8 to 18.83)	15.55 (8.75 to 22.33)	16.86 (8.89 to 24.73)	17.53 (9.32 to 27.9)
Low SDI	40-44 years	0.52 (0.29 to 0.67)	0.64 (0.36 to 0.94)	0.59 (0.3 to 0.86)	0.74 (0.39 to 1.09)	20.05 (11.1 to 26.1)	21.36 (12.15 to 31.62)	20.11 (10.4 to 28.95)	19.86 (10.46 to 29.61)
Low SDI	45-49 years	0.7 (0.39 to 0.9)	0.8 (0.45 to 1.15)	0.73 (0.38 to 1.07)	0.87 (0.47 to 1.3)	25.02 (13.86 to 32.5)	25.1 (13.91 to 36.67)	23.37 (12.24 to 34.14)	22.34 (11.89 to 34.64)
Low SDI	50-54 years	1.2 (0.65 to 1.64)	1.29 (0.71 to 1.8)	1.09 (0.58 to 1.55)	1.22 (0.6 to 1.74)	39.63 (21.2 to 54.5)	37.77 (20.89 to 53.73)	32.53 (17.58 to 45.84)	30.18 (15 to 44.89)
Low SDI	55-59 years	1.33 (0.72 to 1.75)	1.44 (0.8 to 1.99)	1.33 (0.72 to 1.98)	1.48 (0.81 to 2.2)	39.64 (21.2 to 52.72)	38.48 (21.34 to 53.47)	36.66 (19.86 to 54.24)	34.51 (19.07 to 52.41)
Low SDI	60-64 years	1.75 (0.91 to 2.27)	1.86 (1.02 to 2.6)	1.62 (0.87 to 2.34)	1.74 (0.9 to 2.7)	46.6 (24.17 to 60.3)	44.64 (24.33 to 62.89)	40.58 (21.6 to 58.32)	37.58 (19.57 to 59.7)
Low SDI	65-69 years	2.28 (1.21 to 3)	2.31 (1.25 to 3.19)	2.07 (1.22 to 2.99)	2.25 (1.22 to 3.23)	53.26 (28.12 to 69.49)	48.26 (26.4 to 67.68)	45.6 (27.03 to 65.97)	42.93 (23.61 to 62.93)
Low SDI	70-74 years	2.63 (1.44 to 3.4)	2.64 (1.41 to 3.61)	2.3 (1.33 to 3.24)	2.55 (1.37 to 3.77)	53.46 (29.11 to 69.11)	48.05 (26.28 to 65.8)	44.67 (25.94 to 63.49)	43.36 (23.52 to 65.47)
Low SDI	75-79 years	2.95 (1.62 to 3.68)	3.03 (1.65 to 4.01)	2.59 (1.55 to 3.8)	2.88 (1.57 to 4.41)	51.77 (28.3 to 65)	47.87 (26.49 to 64.03)	44.04 (26.52 to 64.78)	43.8 (23.95 to 66.96)
Low SDI	80-84 years	3.1 (1.76 to 3.96)	3.5 (1.94 to 4.61)	2.61 (1.55 to 3.91)	3.23 (1.76 to 4.82)	46.47 (26.36 to 59.5)	47.94 (26.82 to 63.13)	38.28 (22.9 to 57.54)	43.22 (23.82 to 65.38)
Low SDI	85-89 years	3.18 (1.76 to 4.16)	3.87 (2.04 to 4.99)	2.74 (1.57 to 4.36)	3.42 (1.73 to 5.39)	40.44 (22.53 to 53.13)	45.2 (23.95 to 58.19)	34.38 (19.92 to 55.43)	39.64 (20.21 to 63.82)
Low SDI	90-94 years	3.57 (2.05 to 4.69)	4.37 (2.44 to 5.61)	2.65 (1.53 to 4.02)	3.6 (1.78 to 5.28)	44 (25.19 to 58.14)	50.99 (28.68 to 65.39)	32.37 (18.86 to 49.36)	41.86 (20.9 to 62.63)

Low SDI	95+ years	3.27 (1.9 to 4.62)	3.61 (2.02 to 4.93)	2.79 (1.54 to 4.8)	3.82 (1.85 to 5.78)	42.16 (25.51 to 59.49)	44.82 (25.44 to 61.31)	35.59 (19.67 to 63.21)	46.93 (23.26 to 72.97)

DALYs, disability-adjusted life-years; SDI, socio-demographic index; UI, uncertainty interval.

TABLE S25 Age-standardized rates of incidence and DALYs of non-melanoma skin cancer (basal-cell carcinoma) for global and SDI regions, by year (1990, 2021), age group and sex, with age-standardized rates and 95% uncertainty intervals.

Location name	Age group	Incidence				DALYs			
		Male		Female		Male		Female	
		Age-standardized rate (95% UI), 1990	Age-standardized rate (95% UI), 2021	Age-standardized rate (95% UI), 1990	Age-standardized rate (95% UI), 2021	Age-standardized rate (95% UI), 1990	Age-standardized rate (95% UI), 2021	Age-standardized rate (95% UI), 1990	Age-standardized rate (95% UI), 2021
Global	15-19 years	0 (0 to 0)	0 (0 to 0)	0 (0 to 0)	0 (0 to 0)	0 (0 to 0)	0 (0 to 0)	0 (0 to 0)	0 (0 to 0)
Global	20-24 years	1.24 (0.72 to 1.92)	1.47 (0.91 to 2.19)	1.57 (0.93 to 2.31)	1.83 (1.21 to 2.62)	0 (0 to 0)	0 (0 to 0)	0 (0 to 0)	0 (0 to 0)
Global	25-29 years	2.33 (1.31 to 3.74)	2.74 (1.62 to 4.2)	3.2 (1.84 to 4.99)	3.84 (2.45 to 5.61)	0 (0 to 0)	0 (0 to 0)	0 (0 to 0)	0 (0 to 0.01)
Global	30-34 years	4.67 (3.25 to 6.64)	5.65 (4.17 to 7.8)	6.57 (4.67 to 9.39)	8.49 (6.49 to 11.27)	0 (0 to 0.01)	0 (0 to 0.01)	0 (0 to 0.01)	0.01 (0 to 0.01)
Global	35-39 years	7.91 (4.66 to 12.09)	10.06 (7.05 to 14.21)	11.02 (6.58 to 16.44)	15.42 (10.9 to 21.08)	0 (0 to 0.01)	0.01 (0 to 0.01)	0.01 (0 to 0.01)	0.01 (0 to 0.02)
Global	40-44 years	14.84 (10.7 to 19.83)	19.83 (15.44 to 24.88)	18.79 (13.44 to 25.31)	26.43 (20.9 to 32.64)	0.01 (0 to 0.02)	0.01 (0 to 0.02)	0.01 (0 to 0.02)	0.01 (0.01 to 0.03)
Global	45-49	23.57 (14.68 to	35 (24.56 to 48.01)	26.99 (16.56 to	41.61 (29.78 to	0.01 (0.01 to 0.03)	0.02 (0.01 to 0.04)	0.01 (0.01 to 0.03)	0.02 (0.01 to 0.05)

	years	34.47)		39.86)	56.38)				
Global	50-54 years	34.64 (25.1 to 47.35)	63.68 (49.84 to 79.26)	35.4 (25.4 to 49.03)	63.18 (50.02 to 80.56)	0.02 (0.01 to 0.04)	0.03 (0.01 to 0.06)	0.02 (0.01 to 0.04)	0.03 (0.01 to 0.07)
Global	55-59 years	52.05 (33.59 to 76.12)	107.67 (77.82 to 139.93)	48.44 (31.09 to 70.38)	89.9 (65.44 to 117.22)	0.03 (0.01 to 0.05)	0.05 (0.02 to 0.1)	0.03 (0.01 to 0.05)	0.04 (0.02 to 0.09)
Global	60-64 years	91.41 (68.84 to 122.95)	193.91 (155.42 to 237.05)	75.65 (54.63 to 104.4)	133.98 (102.64 to 167.96)	0.04 (0.02 to 0.08)	0.08 (0.04 to 0.16)	0.04 (0.02 to 0.08)	0.06 (0.03 to 0.12)
Global	65-69 years	163.77 (129.05 to 201.41)	308.52 (263.5 to 352.53)	119.41 (91.79 to 146.99)	187.73 (159.69 to 217.28)	0.07 (0.03 to 0.14)	0.13 (0.06 to 0.25)	0.06 (0.03 to 0.11)	0.09 (0.04 to 0.17)
Global	70-74 years	241.83 (182.78 to 312.06)	455.5 (386.88 to 532.98)	166.57 (126.79 to 217.52)	255.82 (214.02 to 300.95)	0.11 (0.05 to 0.21)	0.19 (0.09 to 0.36)	0.08 (0.04 to 0.16)	0.12 (0.05 to 0.23)
Global	75-79 years	330.73 (240.59 to 437.35)	589.01 (487.6 to 703.46)	220.61 (159.72 to 300.48)	315.35 (257.31 to 378.45)	0.15 (0.07 to 0.29)	0.24 (0.11 to 0.47)	0.11 (0.05 to 0.22)	0.14 (0.06 to 0.28)
Global	80-84 years	419.91 (325.94 to 523.84)	677.91 (572.03 to 783.94)	277.83 (212.14 to 359.67)	339.78 (281.56 to 399.21)	0.19 (0.08 to 0.38)	0.28 (0.12 to 0.56)	0.14 (0.06 to 0.28)	0.15 (0.07 to 0.31)
Global	85-89 years	531.73 (412.17 to 687.15)	809.37 (690.12 to 962.43)	348.24 (269.16 to 446.67)	382.36 (321.35 to 462.85)	0.23 (0.1 to 0.45)	0.33 (0.16 to 0.64)	0.16 (0.07 to 0.31)	0.17 (0.08 to 0.32)
Global	90-94 years	694.74 (462.53 to 1007.48)	1038.47 (830.26 to 1318.66)	453.96 (310.44 to 645.09)	467.26 (365.17 to 610.95)	0.3 (0.13 to 0.57)	0.42 (0.18 to 0.78)	0.2 (0.08 to 0.39)	0.2 (0.09 to 0.39)
Global	95+ years	970.68 (542.68 to 1547.37)	1193.42 (852.35 to 1619.84)	587.6 (324.74 to 940.55)	552.92 (375.81 to 779.82)	0.41 (0.16 to 0.83)	0.48 (0.2 to 0.94)	0.25 (0.1 to 0.5)	0.23 (0.1 to 0.44)
High SDI	15-19 years	0 (0 to 0)	0 (0 to 0)	0 (0 to 0)	0 (0 to 0)	0 (0 to 0)	0 (0 to 0)	0 (0 to 0)	0 (0 to 0)
High SDI	20-24 years	3.59 (2.14 to 5.21)	4.95 (3.6 to 6.35)	5.26 (3.17 to 7.5)	7.77 (5.9 to 9.7)	0 (0 to 0)	0 (0 to 0.01)	0 (0 to 0.01)	0 (0 to 0.01)
High	25-29	7.24 (3.87 to 11.53)	9.08 (6.3 to 12.6)	11.62 (6.59 to	16.2 (11.69 to	0 (0 to 0.01)	0.01 (0 to 0.01)	0.01 (0 to 0.02)	0.01 (0 to 0.02)

SDI	years			18.38)	21.32)				
High SDI	30-34 years	15.44 (10.7 to 22.08)	22.32 (17.83 to 28.67)	24.15 (17.41 to 34.68)	37.81 (30.49 to 47.38)	0.01 (0 to 0.02)	0.01 (0 to 0.02)	0.01 (0.01 to 0.03)	0.02 (0.01 to 0.04)
High SDI	35-39 years	27.26 (15.48 to 42.09)	42.99 (32.37 to 56.53)	41.15 (24 to 62.98)	70.39 (52.99 to 90.74)	0.01 (0.01 to 0.03)	0.02 (0.01 to 0.04)	0.02 (0.01 to 0.05)	0.04 (0.02 to 0.07)
High SDI	40-44 years	47.58 (33.49 to 64.01)	85.63 (70.18 to 102.76)	62.86 (44.18 to 85.05)	118.54 (97.86 to 141.88)	0.02 (0.01 to 0.05)	0.04 (0.02 to 0.08)	0.03 (0.01 to 0.07)	0.06 (0.02 to 0.11)
High SDI	45-49 years	76.41 (46.1 to 114.06)	146.43 (111.16 to 190)	89.14 (53.33 to 134.39)	176.78 (136.22 to 227.68)	0.04 (0.02 to 0.08)	0.07 (0.03 to 0.14)	0.04 (0.02 to 0.1)	0.08 (0.04 to 0.17)
High SDI	50-54 years	107.42 (76.4 to 148.73)	251.68 (203.54 to 302.93)	109.88 (78.21 to 152.66)	245.94 (200.99 to 300.72)	0.05 (0.02 to 0.1)	0.11 (0.05 to 0.22)	0.05 (0.02 to 0.12)	0.11 (0.05 to 0.23)
High SDI	55-59 years	154.29 (95.37 to 231.28)	398.79 (299.24 to 502.75)	141.33 (88.37 to 208.59)	321.29 (243.61 to 399.85)	0.07 (0.03 to 0.14)	0.17 (0.08 to 0.32)	0.07 (0.03 to 0.13)	0.14 (0.06 to 0.27)
High SDI	60-64 years	266.76 (197.54 to 364.09)	688.49 (561.7 to 811.31)	206.25 (145.09 to 288.96)	453.57 (359.84 to 545.71)	0.12 (0.05 to 0.23)	0.28 (0.13 to 0.53)	0.1 (0.04 to 0.2)	0.2 (0.09 to 0.38)
High SDI	65-69 years	463.17 (357.92 to 574.76)	1068.63 (925.51 to 1208.21)	298.83 (226.34 to 370.93)	614.45 (535 to 696.66)	0.2 (0.08 to 0.37)	0.43 (0.2 to 0.82)	0.14 (0.06 to 0.26)	0.26 (0.12 to 0.51)
High SDI	70-74 years	685.74 (505.78 to 896.06)	1395.64 (1197.33 to 1608.73)	411.8 (309.08 to 543.24)	743.89 (635.21 to 853.32)	0.29 (0.13 to 0.58)	0.56 (0.26 to 1.07)	0.2 (0.08 to 0.38)	0.32 (0.15 to 0.62)
High SDI	75-79 years	814.41 (581.22 to 1090.55)	1736.05 (1452.77 to 2028.33)	471.13 (334.36 to 650.88)	874.56 (725.52 to 1014.42)	0.35 (0.16 to 0.69)	0.69 (0.31 to 1.35)	0.23 (0.09 to 0.45)	0.37 (0.17 to 0.74)
High SDI	80-84 years	970.19 (743.63 to 1213.42)	1791.06 (1554.04 to 2040.56)	541.18 (407.5 to 707.45)	839.96 (717.7 to 961.95)	0.42 (0.18 to 0.83)	0.71 (0.33 to 1.41)	0.26 (0.11 to 0.51)	0.36 (0.16 to 0.72)
High SDI	85-89 years	1175.84 (901.78 to 1525)	1928.29 (1664.45 to 2258.26)	626.56 (480.31 to 806.48)	825.65 (712.58 to 973.07)	0.5 (0.22 to 0.97)	0.76 (0.36 to 1.47)	0.29 (0.13 to 0.54)	0.35 (0.16 to 0.67)
High	90-94	1457.65 (958.53 to	2232.37 (1805.16 to	741.1 (503.28 to	883.55 (709.09 to	0.61 (0.26 to 1.17)	0.87 (0.38 to 1.64)	0.32 (0.14 to 0.61)	0.37 (0.16 to 0.7)

SDI	years	2130.17)	2780.79)	1060.45)	1120.71)				
High SDI	95+ years	1968.56 (1088.9 to 3149.12)	2302.62 (1667.04 to 3062.35)	915.66 (501.3 to 1472.04)	910.63 (639.47 to 1254.56)	0.81 (0.31 to 1.65)	0.9 (0.39 to 1.76)	0.38 (0.15 to 0.77)	0.37 (0.16 to 0.71)
High-middle SDI	15-19 years	0 (0 to 0)	0 (0 to 0)	0 (0 to 0)	0 (0 to 0)	0 (0 to 0)	0 (0 to 0)	0 (0 to 0)	0 (0 to 0)
High-middle SDI	20-24 years	0.95 (0.53 to 1.52)	1.87 (1.01 to 2.95)	1.23 (0.72 to 1.89)	2.28 (1.25 to 3.44)	0 (0 to 0)	0 (0 to 0)	0 (0 to 0)	0 (0 to 0)
High-middle SDI	25-29 years	1.5 (0.8 to 2.38)	3.24 (1.6 to 5.45)	2.01 (1.13 to 3.12)	4.53 (2.37 to 7.37)	0 (0 to 0)	0 (0 to 0)	0 (0 to 0)	0 (0 to 0.01)
High-middle SDI	30-34 years	2.51 (1.7 to 3.58)	5.22 (3.19 to 7.77)	3.29 (2.27 to 4.64)	8.25 (5.46 to 11.99)	0 (0 to 0)	0 (0 to 0.01)	0 (0 to 0.01)	0.01 (0 to 0.01)
High-middle SDI	35-39 years	3.92 (2.46 to 5.87)	7.59 (4.05 to 12.32)	4.93 (3.13 to 7.3)	12.74 (7.48 to 19.45)	0 (0 to 0.01)	0.01 (0 to 0.01)	0 (0 to 0.01)	0.01 (0 to 0.02)
High-middle SDI	40-44 years	7.65 (5.8 to 10.02)	13.62 (9.27 to 18.96)	8.56 (6.34 to 11.38)	19.34 (13.8 to 26.42)	0.01 (0 to 0.01)	0.01 (0 to 0.02)	0.01 (0 to 0.01)	0.01 (0.01 to 0.03)
High-middle SDI	45-49 years	13.64 (9.57 to 18.69)	23.99 (14.43 to 35.9)	13.69 (9.2 to 19.01)	29.74 (18.55 to 43.76)	0.01 (0 to 0.02)	0.01 (0.01 to 0.03)	0.01 (0 to 0.02)	0.02 (0.01 to 0.04)
High-middle	50-54 years	22.02 (16.94 to 28.27)	40.94 (29.87 to 55.38)	20.67 (15.57 to 27.32)	43.54 (31.86 to 58.68)	0.01 (0.01 to 0.03)	0.02 (0.01 to 0.05)	0.01 (0.01 to 0.03)	0.03 (0.01 to 0.06)

SDI									
High-middle SDI	55-59 years	34.83 (25.3 to 46.52)	62.7 (41.23 to 88.94)	29.78 (20.84 to 40.94)	58.44 (38.66 to 83.19)	0.02 (0.01 to 0.04)	0.03 (0.01 to 0.07)	0.02 (0.01 to 0.04)	0.03 (0.01 to 0.07)
High-middle SDI	60-64 years	55.9 (44.95 to 70.14)	94.86 (70.86 to 126.19)	45.4 (34.83 to 59.23)	76.32 (55.04 to 104.37)	0.03 (0.01 to 0.06)	0.05 (0.02 to 0.1)	0.03 (0.01 to 0.06)	0.04 (0.02 to 0.09)
High-middle SDI	65-69 years	87.87 (74.53 to 101.99)	144.47 (115.89 to 175.35)	66.15 (54.5 to 79.1)	102.39 (79.39 to 126.34)	0.05 (0.02 to 0.09)	0.07 (0.03 to 0.14)	0.04 (0.02 to 0.08)	0.06 (0.02 to 0.11)
High-middle SDI	70-74 years	113.26 (93.1 to 136.51)	195.73 (151.84 to 248.59)	83.94 (67.86 to 105.02)	128.55 (97.36 to 168.6)	0.06 (0.03 to 0.14)	0.1 (0.04 to 0.19)	0.05 (0.02 to 0.11)	0.07 (0.03 to 0.14)
High-middle SDI	75-79 years	161.76 (128 to 202.21)	253.13 (187.83 to 332.97)	112.6 (86.43 to 148.06)	157.66 (113.33 to 218.5)	0.09 (0.04 to 0.2)	0.12 (0.05 to 0.25)	0.07 (0.03 to 0.14)	0.09 (0.04 to 0.18)
High-middle SDI	80-84 years	204.32 (169.3 to 243.39)	283.28 (217.66 to 361.25)	135.95 (109.93 to 169.77)	175.16 (130.7 to 234.62)	0.12 (0.05 to 0.23)	0.14 (0.06 to 0.27)	0.08 (0.04 to 0.17)	0.1 (0.04 to 0.2)
High-middle SDI	85-89 years	242.6 (197.72 to 299.44)	307.85 (235.23 to 402.86)	155.97 (125.51 to 195.07)	193.04 (144.81 to 257.11)	0.13 (0.06 to 0.25)	0.15 (0.07 to 0.3)	0.09 (0.04 to 0.18)	0.1 (0.05 to 0.2)
High-middle SDI	90-94 years	287.08 (211.84 to 386.85)	335.04 (221.41 to 490.75)	179.99 (131.84 to 244.11)	213.57 (140.36 to 314.62)	0.15 (0.06 to 0.29)	0.16 (0.07 to 0.32)	0.09 (0.04 to 0.19)	0.11 (0.05 to 0.22)
High-	95+	326.74 (212.03 to	377.52 (202.34 to	201.03 (127.95 to	241.25 (126.69 to	0.16 (0.06 to 0.33)	0.18 (0.07 to 0.39)	0.1 (0.04 to 0.2)	0.11 (0.04 to 0.24)

middle SDI	years	469.56)	614.94)	295.1)	403.19)				
Middle SDI	15-19 years	0 (0 to 0)	0 (0 to 0)	0 (0 to 0)	0 (0 to 0)	0 (0 to 0)	0 (0 to 0)	0 (0 to 0)	0 (0 to 0)
Middle SDI	20-24 years	1.06 (0.6 to 1.69)	1.35 (0.73 to 2.16)	1.24 (0.73 to 1.95)	1.53 (0.85 to 2.38)	0 (0 to 0)	0 (0 to 0)	0 (0 to 0)	0 (0 to 0)
Middle SDI	25-29 years	1.71 (0.94 to 2.74)	2.37 (1.21 to 3.99)	1.93 (1.06 to 3.06)	2.9 (1.54 to 4.78)	0 (0 to 0)	0 (0 to 0)	0 (0 to 0)	0 (0 to 0)
Middle SDI	30-34 years	2.78 (1.9 to 3.94)	3.87 (2.38 to 5.74)	3.12 (2.16 to 4.39)	5.52 (3.62 to 8.1)	0 (0 to 0)	0 (0 to 0.01)	0 (0 to 0)	0 (0 to 0.01)
Middle SDI	35-39 years	4.13 (2.61 to 6.23)	5.52 (2.97 to 9)	4.65 (3.01 to 6.86)	8.55 (4.97 to 13.32)	0 (0 to 0)	0 (0 to 0.01)	0 (0 to 0.01)	0.01 (0 to 0.01)
Middle SDI	40-44 years	6.66 (4.85 to 8.93)	9.39 (6.35 to 13.39)	7.28 (5.33 to 9.67)	13.17 (9.21 to 18.21)	0 (0 to 0.01)	0.01 (0 to 0.01)	0 (0 to 0.01)	0.01 (0 to 0.02)
Middle SDI	45-49 years	10.07 (6.54 to 14.37)	16.7 (9.61 to 25.05)	10.63 (6.97 to 15.35)	21.37 (13.01 to 31.81)	0.01 (0 to 0.01)	0.01 (0 to 0.02)	0.01 (0 to 0.01)	0.01 (0.01 to 0.03)
Middle SDI	50-54 years	15.53 (11.53 to 20.85)	29.9 (21.49 to 40.52)	14.92 (10.96 to 19.92)	32.48 (23.25 to 44.63)	0.01 (0 to 0.02)	0.02 (0.01 to 0.03)	0.01 (0 to 0.02)	0.02 (0.01 to 0.04)
Middle SDI	55-59 years	22.55 (15.2 to 31.54)	48.41 (31.72 to 68.62)	19.8 (13.21 to 28.02)	45.79 (30.51 to 65.18)	0.01 (0 to 0.02)	0.02 (0.01 to 0.05)	0.01 (0 to 0.02)	0.02 (0.01 to 0.05)
Middle SDI	60-64 years	34.83 (26.51 to 46.24)	67.51 (49.98 to 89.99)	29.46 (22.22 to 39.54)	54.94 (38.54 to 74.9)	0.02 (0.01 to 0.04)	0.03 (0.01 to 0.07)	0.02 (0.01 to 0.03)	0.03 (0.01 to 0.06)
Middle SDI	65-69 years	50.56 (41.01 to 61.46)	107.02 (86.11 to 128.87)	41.11 (33.28 to 50.07)	76.59 (58.88 to 94.78)	0.02 (0.01 to 0.05)	0.05 (0.02 to 0.1)	0.02 (0.01 to 0.04)	0.04 (0.02 to 0.08)
Middle SDI	70-74 years	67.81 (53.88 to 85.12)	143.02 (112.96 to 178.19)	53.49 (42.88 to 67.14)	94.48 (72.38 to 120.76)	0.03 (0.01 to 0.06)	0.07 (0.03 to 0.13)	0.03 (0.01 to 0.05)	0.05 (0.02 to 0.09)

Middle SDI	75-79 years	90.4 (69.47 to 118)	178.57 (134.11 to 229.85)	69.7 (53.54 to 90.23)	111.97 (80.9 to 153.92)	0.04 (0.02 to 0.08)	0.08 (0.04 to 0.15)	0.03 (0.01 to 0.07)	0.06 (0.02 to 0.11)
Middle SDI	80-84 years	108.35 (86.38 to 135.47)	188.64 (145.62 to 237.15)	82.98 (66.08 to 102.91)	118.06 (87.87 to 157.86)	0.05 (0.02 to 0.1)	0.08 (0.04 to 0.16)	0.04 (0.02 to 0.08)	0.06 (0.03 to 0.11)
Middle SDI	85-89 years	124.44 (98.26 to 159.49)	168.24 (132.25 to 217.46)	92.55 (72.98 to 118.83)	114.69 (87.44 to 151.08)	0.06 (0.03 to 0.12)	0.08 (0.03 to 0.15)	0.04 (0.02 to 0.08)	0.06 (0.02 to 0.11)
Middle SDI	90-94 years	137.39 (94.51 to 197.11)	153.82 (106.94 to 221.21)	90.94 (63.45 to 128.27)	112.12 (77.09 to 162.14)	0.07 (0.03 to 0.13)	0.08 (0.03 to 0.15)	0.04 (0.02 to 0.09)	0.06 (0.02 to 0.11)
Middle SDI	95+ years	151.96 (83.84 to 246.22)	161.93 (85.66 to 265.89)	76.27 (43.84 to 120.65)	112.77 (59.34 to 187.42)	0.08 (0.03 to 0.16)	0.08 (0.03 to 0.18)	0.04 (0.01 to 0.08)	0.06 (0.02 to 0.13)
Low-middle SDI	15-19 years	0 (0 to 0)	0 (0 to 0)	0 (0 to 0)	0 (0 to 0)	0 (0 to 0)	0 (0 to 0)	0 (0 to 0)	0 (0 to 0)
Low-middle SDI	20-24 years	0.53 (0.25 to 1.03)	0.64 (0.32 to 1.14)	0.56 (0.27 to 1.04)	0.61 (0.3 to 1.15)	0 (0 to 0)	0 (0 to 0)	0 (0 to 0)	0 (0 to 0)
Low-middle SDI	25-29 years	0.81 (0.4 to 1.41)	1 (0.47 to 1.75)	0.85 (0.42 to 1.45)	0.99 (0.48 to 1.74)	0 (0 to 0)	0 (0 to 0)	0 (0 to 0)	0 (0 to 0)
Low-middle SDI	30-34 years	1.27 (0.79 to 1.93)	1.48 (0.87 to 2.27)	1.35 (0.82 to 2.01)	1.56 (0.95 to 2.36)	0 (0 to 0)	0 (0 to 0)	0 (0 to 0)	0 (0 to 0)
Low-middle SDI	35-39 years	1.88 (1.07 to 3.01)	2.11 (1.11 to 3.57)	2.02 (1.16 to 3.24)	2.32 (1.26 to 3.84)	0 (0 to 0)	0 (0 to 0)	0 (0 to 0)	0 (0 to 0)
Low-	40-44	3.05 (2.07 to 4.36)	3.24 (2.08 to 4.9)	3.13 (2.15 to 4.47)	3.39 (2.21 to 5.13)	0 (0 to 0)	0 (0 to 0)	0 (0 to 0)	0 (0 to 0)

middle SDI	years								
Low-middle SDI	45-49 years	4.66 (2.85 to 7.03)	4.73 (2.63 to 7.41)	4.62 (2.87 to 6.9)	4.69 (2.63 to 7.29)	0 (0 to 0.01)	0 (0 to 0.01)	0 (0 to 0.01)	0 (0 to 0.01)
Low-middle SDI	50-54 years	7.25 (5.08 to 10.06)	7.35 (4.88 to 10.74)	6.75 (4.68 to 9.37)	6.89 (4.56 to 10.04)	0 (0 to 0.01)	0 (0 to 0.01)	0 (0 to 0.01)	0 (0 to 0.01)
Low-middle SDI	55-59 years	10.54 (6.61 to 15.58)	11.14 (6.46 to 17.58)	9.22 (5.73 to 13.7)	10.07 (5.9 to 15.89)	0.01 (0 to 0.01)	0.01 (0 to 0.01)	0 (0 to 0.01)	0.01 (0 to 0.01)
Low-middle SDI	60-64 years	16.19 (11.67 to 22.53)	15.58 (10.4 to 22.94)	13.31 (9.35 to 18.84)	13.39 (8.72 to 19.87)	0.01 (0 to 0.02)	0.01 (0 to 0.02)	0.01 (0 to 0.01)	0.01 (0 to 0.01)
Low-middle SDI	65-69 years	24.97 (19.52 to 30.83)	20.65 (15.24 to 26.65)	19.07 (14.75 to 23.93)	17.01 (12.42 to 22.18)	0.01 (0.01 to 0.02)	0.01 (0 to 0.02)	0.01 (0 to 0.02)	0.01 (0 to 0.02)
Low-middle SDI	70-74 years	34.88 (27.19 to 44.17)	26.25 (19.18 to 35.45)	25.51 (19.48 to 32.71)	20.98 (15.22 to 28.45)	0.02 (0.01 to 0.03)	0.01 (0.01 to 0.03)	0.01 (0.01 to 0.03)	0.01 (0 to 0.02)
Low-middle SDI	75-79 years	47.15 (35.46 to 61.28)	31.46 (22.01 to 44.55)	34.96 (25.67 to 46.39)	24.65 (16.63 to 35.56)	0.02 (0.01 to 0.04)	0.02 (0.01 to 0.03)	0.02 (0.01 to 0.04)	0.01 (0.01 to 0.03)
Low-middle SDI	80-84 years	50.61 (39.58 to 63.2)	37.01 (26.94 to 50.39)	39.65 (30.24 to 49.91)	28.83 (20.07 to 39.55)	0.02 (0.01 to 0.05)	0.02 (0.01 to 0.04)	0.02 (0.01 to 0.04)	0.02 (0.01 to 0.03)

Low-middle SDI	85-89 years	54.87 (43.08 to 70.78)	41.48 (31.34 to 55.2)	47.29 (36.39 to 62)	34.02 (25.13 to 45.15)	0.03 (0.01 to 0.05)	0.02 (0.01 to 0.04)	0.02 (0.01 to 0.05)	0.02 (0.01 to 0.04)
Low-middle SDI	90-94 years	62.74 (43.02 to 89.8)	47.42 (32.02 to 69.35)	59.06 (40.16 to 84.85)	42.89 (28.72 to 63.18)	0.03 (0.01 to 0.07)	0.02 (0.01 to 0.05)	0.03 (0.01 to 0.06)	0.02 (0.01 to 0.05)
Low-middle SDI	95+ years	77.51 (41.72 to 127.7)	72.95 (39.57 to 121.2)	75.17 (40.35 to 121.88)	67.21 (34.48 to 108.87)	0.04 (0.01 to 0.08)	0.04 (0.01 to 0.08)	0.04 (0.01 to 0.08)	0.04 (0.01 to 0.08)
Low SDI	15-19 years	0 (0 to 0)	0 (0 to 0)	0 (0 to 0)	0 (0 to 0)	0 (0 to 0)	0 (0 to 0)	0 (0 to 0)	0 (0 to 0)
Low SDI	20-24 years	0.46 (0.19 to 0.93)	0.5 (0.2 to 0.97)	0.43 (0.17 to 0.91)	0.45 (0.18 to 0.93)	0 (0 to 0)	0 (0 to 0)	0 (0 to 0)	0 (0 to 0)
Low SDI	25-29 years	0.75 (0.32 to 1.4)	0.79 (0.31 to 1.49)	0.69 (0.28 to 1.27)	0.71 (0.28 to 1.34)	0 (0 to 0)	0 (0 to 0)	0 (0 to 0)	0 (0 to 0)
Low SDI	30-34 years	1.11 (0.59 to 1.76)	1.17 (0.6 to 1.89)	1.01 (0.53 to 1.6)	1.03 (0.54 to 1.67)	0 (0 to 0)	0 (0 to 0)	0 (0 to 0)	0 (0 to 0)
Low SDI	35-39 years	1.52 (0.74 to 2.67)	1.61 (0.77 to 2.9)	1.37 (0.65 to 2.47)	1.41 (0.67 to 2.51)	0 (0 to 0)	0 (0 to 0)	0 (0 to 0)	0 (0 to 0)
Low SDI	40-44 years	2.29 (1.41 to 3.52)	2.43 (1.47 to 3.76)	1.88 (1.09 to 2.97)	1.94 (1.13 to 3.08)	0 (0 to 0)	0 (0 to 0)	0 (0 to 0)	0 (0 to 0)
Low SDI	45-49 years	3.46 (1.91 to 5.56)	3.61 (1.95 to 5.89)	2.57 (1.36 to 4.22)	2.64 (1.4 to 4.34)	0 (0 to 0)	0 (0 to 0)	0 (0 to 0)	0 (0 to 0)
Low SDI	50-54 years	5.33 (3.42 to 7.74)	5.49 (3.48 to 8)	3.92 (2.42 to 5.79)	3.96 (2.45 to 5.92)	0 (0 to 0.01)	0 (0 to 0.01)	0 (0 to 0)	0 (0 to 0)
Low SDI	55-59	7.87 (4.28 to 12.49)	8.03 (4.34 to 12.69)	5.89 (3.31 to 9.29)	5.9 (3.31 to 9.39)	0 (0 to 0.01)	0 (0 to 0.01)	0 (0 to 0.01)	0 (0 to 0.01)

	years								
Low SDI	60-64 years	10.91 (6.9 to 16.35)	10.91 (6.87 to 16.21)	7.63 (4.75 to 11.5)	7.54 (4.66 to 11.46)	0.01 (0 to 0.01)	0.01 (0 to 0.01)	0 (0 to 0.01)	0 (0 to 0.01)
Low SDI	65-69 years	14.68 (10.51 to 19.41)	14.12 (10.23 to 18.72)	9.13 (6.5 to 12.23)	8.98 (6.37 to 11.97)	0.01 (0 to 0.02)	0.01 (0 to 0.01)	0 (0 to 0.01)	0 (0 to 0.01)
Low SDI	70-74 years	18.56 (13.31 to 25.32)	16.89 (12.11 to 23.27)	10.65 (7.51 to 14.8)	10.29 (7.12 to 14.53)	0.01 (0 to 0.02)	0.01 (0 to 0.02)	0.01 (0 to 0.01)	0.01 (0 to 0.01)
Low SDI	75-79 years	21.7 (14.56 to 31.28)	20.22 (13.6 to 29.41)	11.94 (7.38 to 17.64)	11.91 (7.17 to 17.8)	0.01 (0.01 to 0.02)	0.01 (0 to 0.02)	0.01 (0 to 0.01)	0.01 (0 to 0.01)
Low SDI	80-84 years	22.34 (15.6 to 30.82)	22.45 (15.58 to 31.07)	12.97 (8.6 to 18.24)	13.6 (8.82 to 19.17)	0.01 (0.01 to 0.03)	0.01 (0.01 to 0.03)	0.01 (0 to 0.02)	0.01 (0 to 0.02)
Low SDI	85-89 years	23.19 (17.08 to 31.54)	23.84 (17.57 to 32.55)	14.79 (10.45 to 20.79)	15.66 (10.99 to 22)	0.01 (0.01 to 0.03)	0.01 (0.01 to 0.03)	0.01 (0 to 0.02)	0.01 (0 to 0.02)
Low SDI	90-94 years	23.71 (15.33 to 35.63)	25.12 (16.12 to 37.79)	16.68 (10.44 to 25.34)	18.21 (11.32 to 27.6)	0.01 (0.01 to 0.03)	0.01 (0.01 to 0.03)	0.01 (0 to 0.02)	0.01 (0 to 0.02)
Low SDI	95+ years	22.7 (11.11 to 38.51)	26.62 (12.89 to 46.04)	18.32 (9.04 to 31.55)	20.62 (10.42 to 34.84)	0.01 (0 to 0.03)	0.02 (0.01 to 0.03)	0.01 (0 to 0.02)	0.01 (0 to 0.03)

DALYs, disability-adjusted life-years; SDI, socio-demographic index; UI, uncertainty interval.

TABLE S26 Age-standardized rates of incidence and DALYs of non-melanoma skin cancer (squamous-cell carcinoma) for global and SDI regions, by year (1990, 2021), age group and sex, with age-standardized rates and 95% uncertainty intervals.

Location name	Age group	Incidence				DALYs			
		Male		Female		Male		Female	

		Age-standardized rate (95% UI), 1990	Age-standardized rate (95% UI), 2021	Age-standardized rate (95% UI), 1990	Age-standardized rate (95% UI), 2021	Age-standardized rate (95% UI), 1990	Age-standardized rate (95% UI), 2021	Age-standardized rate (95% UI), 1990	Age-standardized rate (95% UI), 2021
Global	15-19 years	0 (0 to 0)	0 (0 to 0)	0 (0 to 0)	0 (0 to 0)	0 (0 to 0)	0 (0 to 0)	0 (0 to 0)	0 (0 to 0)
Global	20-24 years	0.35 (0.19 to 0.56)	0.26 (0.14 to 0.42)	0.28 (0.15 to 0.45)	0.19 (0.11 to 0.3)	2.24 (1.77 to 2.72)	1.83 (1.32 to 2.1)	1.99 (1.7 to 2.47)	1.64 (1.43 to 1.86)
Global	25-29 years	0.49 (0.26 to 0.83)	0.38 (0.21 to 0.64)	0.42 (0.22 to 0.7)	0.29 (0.18 to 0.45)	3.38 (2.86 to 4.13)	2.89 (2.15 to 3.34)	2.77 (2.35 to 3.42)	2.39 (2.02 to 2.74)
Global	30-34 years	0.86 (0.54 to 1.34)	0.79 (0.55 to 1.12)	0.8 (0.5 to 1.23)	0.65 (0.48 to 0.9)	5.81 (4.83 to 6.66)	5.82 (3.43 to 7.09)	3.71 (3.2 to 4.44)	3.35 (2.91 to 3.87)
Global	35-39 years	1.39 (0.69 to 2.56)	1.49 (1 to 2.32)	1.32 (0.68 to 2.41)	1.31 (0.9 to 1.98)	8.06 (6.95 to 9.25)	6.39 (4.69 to 7.44)	5.77 (5.08 to 6.94)	5.15 (4.54 to 5.77)
Global	40-44 years	3.01 (1.88 to 4.51)	3.68 (2.76 to 4.88)	2.88 (1.84 to 4.26)	3.45 (2.63 to 4.51)	12.71 (10.5 to 14.68)	10.5 (7.75 to 12.42)	8.26 (7.3 to 9.92)	7.34 (6.52 to 8.21)
Global	45-49 years	5.27 (2.95 to 8.62)	7.41 (5.27 to 10.56)	5.03 (3.01 to 7.99)	7.15 (5.11 to 10.16)	15.56 (13.29 to 17.65)	13.34 (10.21 to 15.55)	11.24 (10.03 to 12.93)	10.42 (9.02 to 11.98)
Global	50-54 years	9.12 (5.86 to 13.65)	16.62 (12.77 to 21.44)	6.82 (4.45 to 9.96)	12.79 (10.03 to 16.35)	23.83 (20.35 to 26.78)	21 (16.21 to 24.26)	16.08 (14.47 to 18.61)	15.48 (13.18 to 17.79)
Global	55-59 years	16.04 (7.92 to 27.28)	33.11 (23.09 to 46.14)	9.36 (4.46 to 16)	20.98 (15.24 to 28.74)	33.52 (28.98 to 37.32)	30.74 (25.73 to 34.73)	21.13 (18.94 to 24.97)	21.92 (18.65 to 25.31)
Global	60-64 years	36.63 (25.02 to 50.46)	80.71 (66.86 to 98.39)	18.52 (11.95 to 26.95)	43.21 (35.33 to 53.05)	57.17 (49.13 to 65.03)	55.8 (45.91 to 64.94)	27.32 (24.56 to 30.75)	27.68 (24.09 to 31.59)
Global	65-69 years	79.57 (58.33 to 109.23)	150.67 (127.79 to 175.66)	37.41 (26.86 to 51.52)	73.01 (62.07 to 85.02)	75.52 (64.3 to 88.09)	75.96 (62.04 to 89.56)	34.8 (31.32 to 39.02)	38.36 (33.14 to 43.71)
Global	70-74 years	129.8 (88.45 to 184.06)	241.92 (200.55 to 292.05)	60.44 (40.97 to 86.44)	112.82 (94.01 to 135)	94.39 (81 to 105.9)	100.61 (84.4 to 115.26)	46.16 (41.8 to 51.93)	51.68 (44.77 to 58.53)

Global	75-79 years	179.22 (117.75 to 259)	324.17 (262.13 to 397.78)	82.12 (52.27 to 120.74)	147.77 (120.36 to 180.96)	118.48 (104.94 to 132.7)	127.22 (107.16 to 144.4)	61.41 (56.54 to 67.33)	64.88 (56.93 to 72.75)
Global	80-84 years	256.65 (189.79 to 344.69)	402.11 (346.94 to 470.29)	125.58 (91.42 to 170.98)	170.55 (145.63 to 201.7)	154.84 (138.08 to 172.27)	174 (147.06 to 193.85)	88.69 (79.26 to 96.54)	91.47 (76.91 to 101.43)
Global	85-89 years	385.08 (268.39 to 529.43)	541.84 (457.71 to 631.81)	205.71 (143.29 to 286.22)	220.52 (185.56 to 257.09)	219.77 (196.02 to 248.53)	261.69 (220.61 to 290.02)	143.63 (126 to 158.14)	139.7 (112.59 to 157.92)
Global	90-94 years	576.7 (357.47 to 860.51)	765.22 (605.24 to 946.1)	338.48 (209.23 to 509.5)	309.41 (244.39 to 382.68)	323.6 (283.32 to 360.97)	414.96 (352.85 to 457.99)	225.18 (187.74 to 250.5)	238.41 (187.81 to 269.79)
Global	95+ years	908.79 (523.82 to 1448.17)	929.09 (691.91 to 1197.51)	539.52 (302.9 to 856.79)	410.64 (302.85 to 533.45)	455.59 (372.79 to 512.8)	590.3 (467.78 to 661.62)	352.08 (273.25 to 400.7)	444.64 (324.96 to 511.01)
High SDI	15-19 years	0 (0 to 0)	0 (0 to 0)	0 (0 to 0)	0 (0 to 0)	0 (0 to 0)	0 (0 to 0)	0 (0 to 0)	0 (0 to 0)
High SDI	20-24 years	1.5 (0.86 to 2.41)	1.03 (0.68 to 1.47)	1.42 (0.83 to 2.26)	0.97 (0.66 to 1.33)	1.91 (1.79 to 2.1)	1.4 (1.32 to 1.59)	1.19 (1.1 to 1.3)	0.88 (0.82 to 0.96)
High SDI	25-29 years	2.15 (1.12 to 3.52)	1.7 (1.11 to 2.55)	2.14 (1.12 to 3.49)	1.75 (1.18 to 2.48)	4.71 (4.53 to 5)	2.92 (2.77 to 3.14)	1.63 (1.52 to 1.78)	1.32 (1.23 to 1.45)
High SDI	30-34 years	3.69 (2.3 to 5.63)	3.86 (2.99 to 4.98)	3.76 (2.34 to 5.64)	4.02 (3.16 to 5.1)	8.78 (8.42 to 9.28)	4.42 (4.2 to 4.79)	2.25 (2.1 to 2.45)	1.81 (1.68 to 2)
High SDI	35-39 years	5.89 (2.96 to 10.87)	7.27 (5.25 to 10.19)	5.99 (3.1 to 10.8)	7.57 (5.5 to 10.53)	10.81 (10.37 to 11.4)	5.51 (5.19 to 6.13)	3.4 (3.18 to 3.74)	2.8 (2.55 to 3.15)
High SDI	40-44 years	11.88 (7.39 to 17.73)	18.72 (14.74 to 23.91)	11.3 (7.21 to 16.71)	18.57 (14.82 to 23.36)	15.57 (14.86 to 16.47)	8.83 (8.19 to 9.92)	5.1 (4.77 to 5.61)	4.58 (4.14 to 5.22)
High SDI	45-49 years	21.5 (12.2 to 34.92)	37.37 (27.36 to 51.03)	19.46 (11.52 to 30.97)	35.92 (26.28 to 48.29)	22.02 (21.05 to 23.1)	13.63 (12.57 to 15.12)	8.22 (7.63 to 9.13)	7.65 (6.77 to 8.89)
High SDI	50-54 years	37.14 (23.92 to 55.12)	80.7 (63.87 to 100.27)	26.66 (17.36 to 38.54)	64.27 (52 to 78.23)	32.95 (31.72 to 34.57)	24.38 (22.64 to 26.91)	12.69 (11.93 to 13.8)	11.98 (10.7 to 14.02)

High SDI	55-59 years	62.98 (30.92 to 107.44)	150.64 (108.96 to 202.01)	36.63 (17.28 to 62.12)	104.83 (76.94 to 139.87)	49.73 (47.45 to 52.85)	40.62 (37.15 to 45.42)	16.24 (15.15 to 17.97)	17.36 (15.3 to 20.6)
High SDI	60-64 years	136.52 (92.98 to 187.74)	336.66 (283.84 to 405.16)	67.47 (43.52 to 97.81)	192.02 (157.42 to 233.81)	73.45 (70.1 to 77.53)	67.63 (61.55 to 76.45)	23.09 (21.55 to 25.47)	24.93 (21.49 to 29.92)
High SDI	65-69 years	271.97 (198.55 to 375.58)	608.76 (520.83 to 701.68)	118.8 (85.1 to 164.2)	310.74 (267.35 to 359.74)	94.36 (88.91 to 100.99)	92.13 (81.49 to 106.02)	30.36 (27.94 to 33.31)	33.52 (28.31 to 40.28)
High SDI	70-74 years	432.98 (293.71 to 616.88)	843.56 (709.09 to 1000.46)	184.38 (124.69 to 264.66)	409.6 (343.72 to 484.65)	116.09 (107.98 to 126.88)	119.23 (104.61 to 137.46)	40.47 (36.79 to 45.19)	43.09 (35.86 to 52.02)
High SDI	75-79 years	516.22 (336.9 to 748.61)	1078.43 (879.95 to 1301.8)	215.12 (136.44 to 318.07)	504.21 (414.17 to 612.29)	146.06 (135.51 to 159.93)	161.28 (141.01 to 185.2)	58.14 (53.12 to 64.08)	59.17 (50.31 to 70.98)
High SDI	80-84 years	685.95 (503.41 to 921.56)	1186.1 (1022.98 to 1369.68)	293.5 (212.13 to 400.55)	518.31 (442.61 to 605.49)	191.16 (176.27 to 208.12)	206.52 (182.11 to 236.2)	84.67 (74.78 to 93.24)	76.42 (63.02 to 90.17)
High SDI	85-89 years	969.77 (672.69 to 1336.26)	1404.87 (1191.58 to 1623.55)	432.11 (300.12 to 602.11)	567.2 (480.92 to 653.3)	271 (245.39 to 299.69)	299.86 (260.66 to 333.58)	134.83 (116.39 to 148.41)	119 (93.94 to 136.36)
High SDI	90-94 years	1347.16 (832.17 to 2012.52)	1754.98 (1404.86 to 2140.81)	618.95 (381.86 to 932.58)	671.78 (538.47 to 817.81)	394.62 (347.75 to 440.88)	476.77 (411.72 to 526.38)	215.06 (177.67 to 242.21)	217.94 (169.2 to 250.88)
High SDI	95+ years	2002.56 (1150.75 to 3191.95)	1908.29 (1431.87 to 2436.51)	912.58 (511.43 to 1449.96)	753.8 (560.56 to 966.47)	591.41 (486.5 to 683.91)	693.97 (557.43 to 777.48)	342.18 (263.8 to 395.51)	431.69 (309.56 to 501.99)
High-middle SDI	15-19 years	0 (0 to 0)	0 (0 to 0)	0 (0 to 0)	0 (0 to 0)	0 (0 to 0)	0 (0 to 0)	0 (0 to 0)	0 (0 to 0)
High-middle SDI	20-24 years	0.16 (0.07 to 0.28)	0.32 (0.12 to 0.6)	0.09 (0.04 to 0.19)	0.14 (0.04 to 0.3)	2.85 (2.43 to 4.07)	2.4 (1.97 to 3.21)	2.57 (2.21 to 3.13)	2 (1.67 to 2.37)
High-middle	25-29 years	0.18 (0.08 to 0.35)	0.37 (0.14 to 0.75)	0.09 (0.03 to 0.19)	0.14 (0.04 to 0.31)	3.69 (3.22 to 5.37)	3.27 (2.69 to 4.41)	3.53 (3.05 to 4.13)	2.77 (2.25 to 3.38)

SDI									
High-middle SDI	30-34 years	0.27 (0.15 to 0.44)	0.6 (0.3 to 1.04)	0.15 (0.07 to 0.26)	0.3 (0.12 to 0.61)	6.04 (5.36 to 7.87)	4.92 (4.09 to 6.44)	4.74 (4.14 to 5.62)	3.87 (3.14 to 4.76)
High-middle SDI	35-39 years	0.41 (0.2 to 0.77)	0.97 (0.39 to 1.93)	0.27 (0.12 to 0.55)	0.61 (0.21 to 1.4)	9.33 (8.26 to 11.86)	7.59 (6.26 to 9.66)	6.95 (6.12 to 8.09)	5.72 (4.85 to 6.83)
High-middle SDI	40-44 years	0.78 (0.48 to 1.19)	1.84 (1.05 to 2.98)	0.82 (0.54 to 1.19)	1.82 (1.1 to 2.83)	15.55 (13.54 to 19.33)	12.41 (10.12 to 16.19)	10.07 (8.85 to 11.78)	8.42 (7.01 to 10.06)
High-middle SDI	45-49 years	1.36 (0.76 to 2.26)	3.36 (1.63 to 6.06)	1.83 (1.22 to 2.72)	4.15 (2.49 to 6.83)	17.52 (15.75 to 20.48)	13.07 (11 to 17.16)	14.17 (12.53 to 16.35)	12.2 (9.91 to 14.86)
High-middle SDI	50-54 years	2.5 (1.65 to 3.77)	6.57 (4.06 to 10.33)	2.05 (1.38 to 3.02)	4.95 (3.15 to 8.03)	27.58 (25 to 31.59)	20.58 (17.71 to 26.45)	18.09 (16.18 to 20.85)	17.05 (13.6 to 20.77)
High-middle SDI	55-59 years	4.46 (2.44 to 7.27)	11.17 (5.23 to 19.8)	1.51 (0.83 to 2.77)	3.88 (1.82 to 7.71)	37.13 (33.47 to 41.61)	29.85 (26.04 to 37.42)	23.58 (21.01 to 27.22)	23.75 (19.05 to 29.08)
High-middle SDI	60-64 years	9.46 (6.91 to 12.7)	20.85 (14 to 29.36)	2.65 (1.72 to 3.96)	5.43 (3.08 to 8.78)	63.62 (56.75 to 74.45)	57.53 (49.63 to 73.7)	29.86 (26.93 to 33.68)	32.12 (27.19 to 38.16)
High-middle SDI	65-69 years	18.48 (14.39 to 23.72)	37.98 (27.91 to 51.2)	5.63 (4.19 to 7.5)	10.01 (6.7 to 14.48)	85.37 (74.24 to 104.07)	78.03 (65 to 106.15)	38.56 (34.92 to 43.43)	42.1 (34.82 to 50.22)
High-	70-74	26.39 (20.02 to	55.49 (39.19 to	8.46 (6.2 to 11.32)	14.51 (9.21 to	106.45 (93.9 to	100.32 (84.59 to	50.67 (45.53 to	56.25 (47.12 to

middle SDI	years	34.79)	78.29)		21.39)	127.87)	130.1)	57.23)	66.03)
High-middle SDI	75-79 years	39.28 (28.62 to 52.61)	75.81 (51.12 to 109.01)	12.26 (8.73 to 16.7)	19.63 (12.21 to 29.75)	132.68 (119.02 to 151.56)	126.53 (107.32 to 154.99)	71.65 (66.83 to 77.35)	70.27 (58.91 to 81.6)
High-middle SDI	80-84 years	53.54 (41.68 to 70.3)	91.44 (65.93 to 126.78)	17.01 (13.16 to 21.86)	25.18 (17.82 to 35.8)	181.8 (164.04 to 208.49)	185.01 (156.18 to 219.65)	112.44 (101.58 to 121.45)	106.44 (89.62 to 119.56)
High-middle SDI	85-89 years	69.72 (53.25 to 91.37)	112.74 (78.6 to 154.83)	22.83 (17.16 to 30.32)	35.53 (23.6 to 51.37)	282.51 (249.57 to 333.31)	289.26 (240.34 to 341.3)	186.62 (162.44 to 204.02)	160.29 (127.51 to 183.83)
High-middle SDI	90-94 years	87.68 (61.25 to 122.9)	128.18 (77.98 to 201.01)	29.38 (20.21 to 41.97)	44.5 (26.31 to 71.65)	437.92 (381.93 to 492.8)	452.12 (375.14 to 507.24)	308.46 (259.22 to 342.51)	272.02 (214.25 to 311.26)
High-middle SDI	95+ years	103.59 (66.93 to 154.73)	146.78 (77.54 to 249.4)	34.88 (22.3 to 53.19)	54.34 (28.19 to 92.62)	599.64 (501.44 to 667.03)	674.57 (531.72 to 767.1)	535.55 (424.93 to 607.49)	518.17 (385.03 to 594.47)
Middle SDI	15-19 years	0 (0 to 0)	0 (0 to 0)	0 (0 to 0)	0 (0 to 0)	0 (0 to 0)	0 (0 to 0)	0 (0 to 0)	0 (0 to 0)
Middle SDI	20-24 years	0.22 (0.1 to 0.38)	0.25 (0.1 to 0.44)	0.14 (0.06 to 0.26)	0.14 (0.05 to 0.28)	3.07 (2.18 to 3.73)	2.83 (1.9 to 3.33)	2.64 (2.18 to 3.23)	2.35 (1.94 to 2.79)
Middle SDI	25-29 years	0.22 (0.1 to 0.41)	0.28 (0.12 to 0.53)	0.13 (0.06 to 0.25)	0.14 (0.06 to 0.28)	4.05 (3.04 to 4.88)	4 (2.72 to 4.86)	3.75 (3.07 to 4.58)	3.47 (2.73 to 4.16)
Middle SDI	30-34 years	0.29 (0.16 to 0.47)	0.44 (0.23 to 0.75)	0.16 (0.09 to 0.28)	0.24 (0.11 to 0.44)	5.93 (4.33 to 7.05)	6.09 (3.77 to 7.49)	5.04 (4.13 to 6)	4.5 (3.63 to 5.51)
Middle	35-39	0.41 (0.2 to 0.77)	0.68 (0.3 to 1.36)	0.25 (0.11 to 0.49)	0.42 (0.16 to 0.92)	9.22 (6.81 to 10.87)	8.35 (5.42 to 10.21)	7.88 (6.65 to 9.54)	6.64 (5.44 to 7.73)

SDI	years								
Middle SDI	40-44 years	0.64 (0.37 to 1.02)	1.19 (0.67 to 1.93)	0.49 (0.29 to 0.76)	0.97 (0.56 to 1.59)	14.54 (10 to 17.77)	13.37 (8.82 to 16.25)	11.82 (10.04 to 14.34)	9.53 (7.97 to 11.21)
Middle SDI	45-49 years	0.94 (0.48 to 1.62)	2.22 (1.05 to 4.01)	0.87 (0.5 to 1.43)	2.2 (1.17 to 3.77)	16.21 (12 to 19.59)	16.05 (11.07 to 19.87)	15.78 (13.47 to 18.73)	13.79 (11.08 to 16.69)
Middle SDI	50-54 years	1.52 (0.95 to 2.37)	4.15 (2.47 to 6.77)	1.04 (0.66 to 1.63)	2.91 (1.72 to 4.94)	23.38 (17.32 to 28.79)	23.21 (15.68 to 28.38)	22.71 (18.96 to 27.39)	20.19 (15.67 to 24.38)
Middle SDI	55-59 years	2.33 (1.1 to 4.13)	6.85 (2.94 to 12.74)	0.99 (0.5 to 1.84)	2.81 (1.27 to 5.7)	31.76 (23.78 to 38.18)	31.34 (22.52 to 37.72)	29.93 (25.23 to 36.31)	27.72 (21.35 to 33.63)
Middle SDI	60-64 years	4.09 (2.66 to 5.9)	11.25 (7.1 to 16.57)	1.6 (0.99 to 2.39)	3.76 (2.12 to 6.17)	61.01 (45.38 to 73.95)	60.53 (41.64 to 75.56)	38.65 (31.99 to 46.35)	34.51 (27.28 to 41.96)
Middle SDI	65-69 years	6.8 (5 to 9.16)	22.1 (15.72 to 30.55)	2.77 (1.99 to 3.76)	7.1 (4.6 to 10.27)	82.99 (58.72 to 103.29)	84.9 (58.13 to 106.04)	48.05 (41 to 57.05)	49.1 (38.31 to 58.9)
Middle SDI	70-74 years	9.67 (6.74 to 13.44)	32.04 (21.59 to 46.15)	4.03 (2.77 to 5.62)	10.24 (6.42 to 15.17)	105.8 (76 to 129.48)	112.58 (77.13 to 138.47)	62.45 (53.68 to 75.15)	67 (51.24 to 79.25)
Middle SDI	75-79 years	12.28 (8.26 to 17.57)	41.51 (26.54 to 61.86)	5.26 (3.5 to 7.52)	13.22 (7.89 to 20.55)	119.18 (89.2 to 145.62)	129.75 (91.22 to 155.16)	69.56 (60.06 to 81.86)	78.53 (63.01 to 92.22)
Middle SDI	80-84 years	14.78 (10.9 to 20.26)	48.89 (34.27 to 69.38)	7.05 (5.21 to 9.68)	16.95 (11.42 to 25.05)	145.05 (115.21 to 179.08)	170.05 (120.61 to 203.56)	89.06 (74.66 to 104.41)	108.37 (84.77 to 125.99)
Middle SDI	85-89 years	15.86 (11.41 to 21.58)	50.12 (33.87 to 71.42)	9.05 (6.43 to 12.47)	21.38 (13.28 to 32.42)	190.79 (154.63 to 243.89)	259.78 (185.47 to 301.86)	143.32 (121.46 to 163.26)	162.39 (122.77 to 190.79)
Middle SDI	90-94 years	16.05 (10.38 to 23.98)	44.42 (24.57 to 73.71)	10.72 (6.67 to 16.36)	24.4 (12.61 to 41.84)	270 (227.2 to 346.04)	392.51 (290.7 to 463.75)	203.43 (165.29 to 240.24)	263.2 (202.18 to 305.48)
Middle SDI	95+ years	13.2 (6.92 to 22.41)	29.28 (13.11 to 54.12)	10.44 (5.78 to 17.16)	24.82 (10.4 to 47.14)	325.75 (258.94 to 419.32)	457.2 (346.11 to 535.87)	263.39 (186.27 to 347.34)	459.46 (341.38 to 540.22)
Low-	15-19	0 (0 to 0)	0 (0 to 0)	0 (0 to 0)	0 (0 to 0)	0 (0 to 0)	0 (0 to 0)	0 (0 to 0)	0 (0 to 0)

middle SDI	years								
Low-middle SDI	20-24 years	0.08 (0.03 to 0.15)	0.07 (0.03 to 0.15)	0.05 (0.02 to 0.11)	0.04 (0.01 to 0.1)	1.11 (0.81 to 1.49)	1.35 (0.96 to 1.63)	1.38 (1.01 to 2.33)	1.49 (1.2 to 1.84)
Low-middle SDI	25-29 years	0.08 (0.03 to 0.16)	0.07 (0.03 to 0.15)	0.04 (0.01 to 0.1)	0.04 (0.01 to 0.09)	1.74 (1.3 to 2.37)	2.15 (1.57 to 2.74)	1.95 (1.41 to 3.33)	2.13 (1.72 to 2.58)
Low-middle SDI	30-34 years	0.1 (0.04 to 0.18)	0.09 (0.04 to 0.17)	0.05 (0.02 to 0.1)	0.05 (0.02 to 0.1)	3.24 (2.14 to 4.12)	5.46 (2.79 to 7.2)	2.59 (1.91 to 4.26)	2.97 (2.46 to 3.55)
Low-middle SDI	35-39 years	0.13 (0.06 to 0.25)	0.13 (0.06 to 0.26)	0.08 (0.03 to 0.16)	0.07 (0.03 to 0.15)	3.86 (2.89 to 5.08)	4.8 (3.57 to 5.9)	4.21 (3.3 to 6.58)	4.92 (4.18 to 5.86)
Low-middle SDI	40-44 years	0.2 (0.11 to 0.35)	0.2 (0.11 to 0.35)	0.14 (0.08 to 0.25)	0.14 (0.07 to 0.24)	6.23 (4.3 to 8.35)	8.29 (5.9 to 10.27)	5.75 (4.53 to 8.93)	6.63 (5.56 to 7.88)
Low-middle SDI	45-49 years	0.3 (0.14 to 0.56)	0.31 (0.13 to 0.59)	0.25 (0.12 to 0.44)	0.24 (0.11 to 0.44)	8.93 (6.4 to 11.99)	11.54 (8.07 to 14.34)	7.08 (5.56 to 10.4)	7.87 (6.56 to 9.37)
Low-middle SDI	50-54 years	0.51 (0.3 to 0.84)	0.54 (0.31 to 0.9)	0.3 (0.18 to 0.5)	0.3 (0.17 to 0.53)	13.93 (10.09 to 18.84)	17.98 (12.66 to 22.47)	10.28 (8.26 to 14.83)	11.47 (9.36 to 13.64)
Low-middle SDI	55-59 years	0.82 (0.36 to 1.54)	0.87 (0.37 to 1.69)	0.3 (0.14 to 0.6)	0.32 (0.14 to 0.69)	18.69 (13.63 to 25.61)	26 (19.27 to 32.81)	13.71 (10.87 to 19.47)	16.89 (14.22 to 20.07)

Low-middle SDI	60-64 years	1.34 (0.81 to 2.06)	1.39 (0.81 to 2.16)	0.45 (0.25 to 0.73)	0.47 (0.25 to 0.79)	30.91 (22.44 to 41.71)	40.16 (29.64 to 51)	16.43 (13.33 to 22.87)	18.69 (16.17 to 21.67)
Low-middle SDI	65-69 years	2.13 (1.52 to 2.9)	2.08 (1.47 to 2.88)	0.77 (0.53 to 1.06)	0.75 (0.48 to 1.08)	37.16 (25.95 to 51.91)	48.79 (34.74 to 62.86)	20.79 (15.96 to 28.42)	25.17 (21.43 to 29.1)
Low-middle SDI	70-74 years	2.94 (2.01 to 4.17)	2.84 (1.88 to 4.02)	1.12 (0.75 to 1.58)	1.07 (0.67 to 1.55)	46.36 (32.58 to 64.82)	65.31 (49.75 to 86.43)	29.47 (22.47 to 40.4)	37.14 (31.96 to 43.18)
Low-middle SDI	75-79 years	3.78 (2.42 to 5.54)	3.55 (2.23 to 5.21)	1.58 (1.01 to 2.29)	1.4 (0.84 to 2.12)	61.02 (41.79 to 84.69)	85.11 (61.74 to 112.35)	40.06 (30.82 to 51.16)	49.72 (42.57 to 57.71)
Low-middle SDI	80-84 years	4.03 (2.83 to 5.75)	4.02 (2.72 to 5.76)	1.78 (1.25 to 2.53)	1.68 (1.1 to 2.5)	74.07 (49.13 to 105.39)	118.16 (91.21 to 155.03)	51.67 (37.08 to 66.4)	73.33 (62.5 to 85.14)
Low-middle SDI	85-89 years	4.01 (2.92 to 5.6)	4.1 (2.9 to 5.82)	1.96 (1.4 to 2.72)	1.88 (1.28 to 2.71)	89.88 (60.19 to 128.11)	149.41 (116.19 to 196.48)	81.34 (55.22 to 108.31)	119.86 (95.24 to 142.82)
Low-middle SDI	90-94 years	4 (2.54 to 5.85)	4.27 (2.73 to 6.29)	2.25 (1.39 to 3.42)	2.21 (1.32 to 3.52)	126.12 (83.62 to 184.83)	228.99 (178.24 to 300.53)	128.46 (86.73 to 170.87)	201.36 (153.98 to 242.81)
Low-middle SDI	95+ years	4.18 (2.04 to 7.43)	5.92 (2.92 to 10.75)	2.67 (1.2 to 4.88)	2.97 (1.27 to 5.69)	158.9 (102.5 to 233.97)	340.75 (243.65 to 433.86)	183.1 (117.29 to 256.64)	338.35 (238.57 to 424.36)
Low SDI	15-19 years	0 (0 to 0)	0 (0 to 0)	0 (0 to 0)	0 (0 to 0)	0 (0 to 0)	0 (0 to 0)	0 (0 to 0)	0 (0 to 0)

Low SDI	20-24 years	0.05 (0.02 to 0.11)	0.06 (0.02 to 0.11)	0.06 (0.02 to 0.11)	0.06 (0.03 to 0.11)	0.54 (0.22 to 0.82)	0.72 (0.26 to 1.16)	0.82 (0.5 to 1.34)	0.93 (0.59 to 1.29)
Low SDI	25-29 years	0.05 (0.02 to 0.11)	0.06 (0.02 to 0.11)	0.05 (0.02 to 0.11)	0.06 (0.03 to 0.11)	1.02 (0.43 to 1.52)	1.43 (0.53 to 2.21)	1.09 (0.77 to 1.82)	1.16 (0.75 to 1.54)
Low SDI	30-34 years	0.06 (0.02 to 0.11)	0.06 (0.02 to 0.12)	0.06 (0.03 to 0.1)	0.06 (0.03 to 0.11)	3.89 (1.54 to 5.58)	8.78 (1.72 to 13.65)	1.71 (1.12 to 2.61)	1.91 (1.23 to 2.55)
Low SDI	35-39 years	0.07 (0.03 to 0.14)	0.07 (0.03 to 0.15)	0.07 (0.03 to 0.13)	0.07 (0.03 to 0.14)	2.17 (0.91 to 3.3)	3.01 (1.08 to 4.77)	3.17 (2.04 to 4.84)	3.27 (2.22 to 4.38)
Low SDI	40-44 years	0.1 (0.04 to 0.18)	0.09 (0.04 to 0.18)	0.1 (0.05 to 0.17)	0.1 (0.06 to 0.17)	3.78 (1.49 to 5.69)	5.12 (1.84 to 7.58)	3.98 (2.57 to 6.2)	4.04 (2.64 to 5.38)
Low SDI	45-49 years	0.13 (0.06 to 0.26)	0.13 (0.06 to 0.26)	0.15 (0.07 to 0.27)	0.15 (0.08 to 0.27)	5.48 (2.27 to 8.12)	7.05 (2.55 to 10.68)	4.06 (2.57 to 6.19)	4.05 (2.54 to 5.56)
Low SDI	50-54 years	0.2 (0.11 to 0.36)	0.2 (0.11 to 0.35)	0.18 (0.1 to 0.31)	0.18 (0.1 to 0.31)	9.05 (4.21 to 13.34)	11.52 (4.3 to 16.74)	6.38 (3.95 to 9.54)	6.33 (3.91 to 9.07)
Low SDI	55-59 years	0.3 (0.12 to 0.59)	0.29 (0.12 to 0.58)	0.19 (0.1 to 0.37)	0.19 (0.1 to 0.37)	11.11 (5 to 16.43)	14.91 (6.09 to 22.03)	9.04 (5.65 to 13.56)	10.36 (6.37 to 14.32)
Low SDI	60-64 years	0.43 (0.23 to 0.73)	0.42 (0.22 to 0.73)	0.23 (0.12 to 0.4)	0.24 (0.12 to 0.42)	20.89 (9.98 to 30.75)	29.5 (11.35 to 43.4)	10.02 (5.91 to 14.05)	11.3 (7.18 to 15.62)
Low SDI	65-69 years	0.58 (0.39 to 0.84)	0.63 (0.42 to 0.9)	0.3 (0.19 to 0.43)	0.33 (0.21 to 0.49)	23.42 (10.16 to 35.51)	30.18 (12.37 to 45.18)	13.25 (7.47 to 18.48)	16.06 (10.18 to 22.31)
Low SDI	70-74 years	0.72 (0.46 to 1.04)	0.87 (0.55 to 1.25)	0.37 (0.23 to 0.56)	0.43 (0.26 to 0.65)	31.56 (13.78 to 46.35)	44.87 (20.68 to 66.03)	19.57 (10.83 to 27.45)	26.33 (16.95 to 34.99)
Low SDI	75-79 years	0.89 (0.5 to 1.37)	1.08 (0.63 to 1.65)	0.45 (0.25 to 0.72)	0.51 (0.28 to 0.81)	43.83 (20.57 to 67.11)	62.11 (25.58 to 95.29)	26.39 (13.93 to 37.33)	33.76 (21.2 to 45.89)
Low SDI	80-84 years	1.09 (0.72 to 1.62)	1.23 (0.82 to 1.8)	0.58 (0.36 to 0.89)	0.65 (0.41 to 0.99)	52.93 (25.67 to 82.8)	84.5 (36.87 to 129.16)	34.16 (15.97 to 52.15)	48.22 (29.39 to 67.43)

Low SDI	85-89 years	1.19 (0.8 to 1.69)	1.27 (0.86 to 1.81)	0.72 (0.48 to 1.04)	0.81 (0.54 to 1.15)	57.01 (26.42 to 92.2)	96.19 (43.73 to 143.92)	55.3 (24.07 to 87.7)	81.03 (43.73 to 118.41)
Low SDI	90-94 years	1.18 (0.71 to 1.87)	1.27 (0.76 to 2.03)	0.81 (0.48 to 1.28)	0.94 (0.55 to 1.52)	78.05 (32.93 to 134.05)	135.72 (60.93 to 218.47)	72.7 (26.94 to 122.11)	115.55 (54.6 to 180.14)
Low SDI	95+ years	1.23 (0.6 to 2.19)	1.19 (0.59 to 2.1)	0.86 (0.44 to 1.48)	1.1 (0.58 to 1.9)	103.57 (42.71 to 177.75)	154.31 (70.18 to 246.25)	99 (34.39 to 177.43)	156.36 (65.23 to 266.31)

DALYs, disability-adjusted life-years; SDI, socio-demographic index; UI, uncertainty interval.

TABLE S27 Changes in population-level determinants, including aging, population growth, and epidemiological factors, on the incidence and DALYs of total skin cancer globally and across different SDI regions from 1990 to 2021.

Location name	Incidence				DALYs			
	Overall difference	Change due to Population-level determinants (% contribute to the total changes)			Overall difference	Change due to Population-level determinants (% contribute to the total changes)		
		Aging	Population	Epidemiological change		Aging	Population	Epidemiological change
Global	6606545.9	2080362.923 （31.49%）	2076211.661 （31.43%）	2449971.314 （37.08%）	1690609.7	866371.95 (51.25%)	1139392.881 (67.4%)	-315155.129 (-18.64%)
High SDI	6126870.69	1616493.552 （26.38%）	1252198.057 （20.44%）	3258179.078 （53.18%）	575505.09	306045.847 (53.18%)	322410.487 (56.02%)	-52951.246 (-9.2%)
High-middle SDI	1115491.69	378950.843	239708.11	496832.739	761192.72	452321.729	313370.879	-4499.885

		（33.97%）	（21.49%）	（44.54%）		(59.42%)	(41.17%)	(-0.59%)
Low SDI	22066.22	1102.574<br>（5%）	20114.708<br>（91.16%）	848.943<br>（3.85%）	151590.35	7944.57<br>(5.24%)	129054.699<br>(85.13%)	14591.083<br>(9.63%)
Low-middle SDI	85463.33	35230.841<br>（41.22%）	64672.1<br>（75.67%）	-14439.608<br>（-16.9%）	408065.18	96249.558<br>(23.59%)	204246.841(50.05%)	107568.781<br>(26.36%)
Middle SDI	643975.26	224790.981<br>（34.91%）	152385.499<br>（23.66%）	266798.775<br>（41.43%）	530421.01	290785.48<br>(54.82%)	225963.507<br>(42.6%)	13672.025<br>(2.58%)

Total skin cancer includes malignant skin melanoma, non-melanoma skin cancer (basal-cell carcinoma) and non-melanoma skin cancer (squamous-cell carcinoma). A positive value for each component indicates a corresponding positive contribution in incidence or DALYs, and a negative value indicates a corresponding negative contribution in incidence or DALYs. DALYs, disability-adjusted life-years; SDI, socio-demographic index.

TABLE S28 Changes in population-level determinants, including aging, population growth, and epidemiological factors, on the incidence and DALYs of malignant skin melanoma globally and across different SDI regions from 1990 to 2021.

Location name	Incidence				DALYs			
	Overall difference	Change due to Population-level determinants (% contribute to the total changes)			Overall difference	Change due to Population-level determinants (% contribute to the total changes)		
		Aging	Population	Epidemiological change		Aging	Population	Epidemiological change

Global	258505.47	35190.757(13.61%)	94774.046(36.66%)	128540.663(49.72%)	633058.77	416653.492(65.82%)	527053.137(83.26%)	-310647.856(-49.07%)
High SDI	31340.1	-16771.713(-53.52%)	18781.301(59.93%)	29330.507(93.59%)	152576.3	88399.718(57.94%)	68741.612(45.05%)	-4565.034(-2.99%)
High-middle SDI	69304.11	20678.557(29.84%)	15562.155(22.45%)	33063.395(47.71%)	-88437.24	117967.544(-133.39%)	98292.504(-111.14%)	-304697.292(344.54%)
Low SDI	115375.73	1635.767(1.42%)	35463.197(30.74%)	78276.763(67.85%)	222722.65	158419.613(71.13%)	184072.342(82.65%)	-119769.305(-53.78%)
Low-middle SDI	13838.94	-3865.788(-27.93%)	7993.623(57.76%)	9711.102(70.17%)	124636.68	31689.076(25.43%)	69722.038(55.94%)	23225.568(18.63%)
Middle SDI	2340.88	142.892(6.1%)	2358.862(100.77%)	-160.872(-6.87%)	73821.5	5312.616(7.2%)	68644.359(92.99%)	-135.473(-0.18%)

A positive value for each component indicates a corresponding positive contribution in incidence or DALYs, and a negative value indicates a corresponding negative contribution in incidence or DALYs. DALYs, disability-adjusted life-years; SDI, socio-demographic index.

TABLE S29 Changes in population-level determinants, including aging, population growth, and epidemiological factors, on the incidence and DALYs of non-melanoma skin cancer (basal-cell carcinoma) globally and across different SDI regions from 1990 to 2021.

Location name	Incidence		DALYs	
	Overall difference	Change due to Population-level determinants (% contribute to the total changes)	Overall difference	Change due to Population-level determinants (% contribute to the total changes)

		Aging	Population	Epidemiological change		Aging	Population	Epidemiological change
Global	6063349.97	1020287.771(16.83%)	2801189.504(46.2%)	2241872.691(36.97%)	1407.58	467.352(33.2%)	453.378(32.21%)	486.845(34.59%)
High SDI	11990.18	636.911(5.31%)	11383.873(94.94%)	-30.602(-0.26%)	6.5	0.352(5.42%)	6.215(95.62%)	-0.066(-1.02%)
High-middle SDI	412337.1	155895.204(37.81%)	95099.23(23.06%)	161342.662(39.13%)	208.66	78.405(37.58%)	49.359(23.66%)	80.892(38.77%)
Low SDI	3290872.05	847830.774(25.76%)	621875.16(18.9%)	1821166.118(55.34%)	1312.09	355.919(27.13%)	267.988(20.42%)	688.188(52.45%)
Low-middle SDI	744086.38	272671.336(36.65%)	160360.742(21.55%)	311054.307(41.8%)	371.92	144.336(38.81%)	88.625(23.83%)	138.96(37.36%)
Middle SDI	57333.68	27097.996(47.26%)	42737.324(74.54%)	-12501.64(-21.81%)	31.81	13.919(43.76%)	22.314(70.15%)	-4.425(-13.91%)

A positive value for each component indicates a corresponding positive contribution in incidence or DALYs, and a negative value indicates a corresponding negative contribution in incidence or DALYs. DALYs, disability-adjusted life-years; SDI, socio-demographic index.

TABLE S30 Changes in population-level determinants, including aging, population growth, and epidemiological factors, on the incidence and DALYs of non-melanoma skin cancer (squamous-cell carcinoma) globally and across different SDI regions from 1990 to 2021.

Location name	Incidence				DALYs			
	Overall difference	Change due to Population-level determinants (% contribute to the total changes)			Overall difference	Change due to Population-level determinants (% contribute to the total changes)		
		Aging	Population	Epidemiological change		Aging	Population	Epidemiological change
Global	3423404.97	-180387.579(-5.27%)	3192183.038(93.25%)	411609.509(12.02%)	665900.84	329603.446(49.5%)	325729.818(48.92%)	10567.581(1.59%)
High SDI	124746.41	52684.488(42.23%)	26652.075(21.37%)	45409.843(36.4%)	377909.3	258459.046(68.39%)	141175.366(37.36%)	-21725.117(-5.75%)
High-middle SDI	2387.26	1587.484(66.5%)	2427.751(101.7%)	-1627.98(-68.19%)	196114.56	56572.9(28.85%)	86057.973(43.88%)	53483.69(27.27%)
Low SDI	2782.91	200.653(7.21%)	2796.652(100.49%)	-214.399(-7.7%)	35408.85	1618.208(4.57%)	24478.546(69.13%)	9312.096(26.3%)
Low-middle SDI	88004.05	3725.056(4.23%)	17555.131(19.95%)	66723.861(75.82%)	259909.1	158044.415(60.81%)	98062.063(37.73%)	3802.62(1.46%)
Middle SDI	1428102.45	514585.401(36.03%)	302029.899(21.15%)	611487.147(42.82%)	211111.89	118922.871(56.33%)	80272.132(38.02%)	11916.886(5.64%)

A positive value for each component indicates a corresponding positive contribution in incidence or DALYs, and a negative value indicates a corresponding negative contribution in incidence or DALYs. DALYs, disability-adjusted life-years; SDI, socio-demographic index.

TABLE S31 Predicated age-standardized rates of incidence and DALYs of total skin cancer by sex from 2022 to 2040, with 95% uncertainty intervals.

Year	Incidence			DALYs		
	Age-standardized rate (95% UI), male	Age-standardized rate (95% UI), female	Age-standardized rate (95% UI), both	Age-standardized rate (95% UI), male	Age-standardized rate (95% UI), female	Age-standardized rate (95% UI), both
2022	109.14 (102.13 to 116.15)	64.74 (60.7 to 68.77)	83.87 (78.56 to 89.18)	41.21 (40.5 to 41.91)	27 (26.52 to 27.48)	33.45 (32.93 to 33.98)
2023	113.76 (101.26 to 126.27)	67.57 (59.9 to 75.23)	87.41 (77.74 to 97.09)	40.62 (39.5 to 41.74)	26.7 (25.93 to 27.47)	33.02 (32.16 to 33.88)
2024	118.52 (98.6 to 138.43)	70.5 (57.98 to 83.03)	91.06 (75.53 to 106.59)	40.03 (38.4 to 41.67)	26.41 (25.27 to 27.55)	32.59 (31.31 to 33.87)
2025	123.4 (94.35 to 152.44)	73.54 (55.03 to 92.05)	94.81 (72.08 to 117.55)	39.43 (37.22 to 41.65)	26.11 (24.56 to 27.67)	32.15 (30.41 to 33.9)
2026	128.4 (88.53 to 168.27)	76.66 (51.04 to 102.28)	98.65 (67.37 to 129.94)	38.84 (35.99 to 41.68)	25.82 (23.81 to 27.82)	31.71 (29.46 to 33.96)
2027	133.54 (81.11 to 185.98)	79.88 (45.97 to 113.78)	102.59 (61.39 to 143.79)	38.24 (34.72 to 41.76)	25.52 (23.03 to 28.02)	31.28 (28.48 to 34.07)
2028	138.79 (72.01 to 205.57)	83.19 (39.79 to 126.59)	106.62 (54.1 to 159.14)	37.66 (33.43 to 41.89)	25.25 (22.23 to 28.26)	30.86 (27.49 to 34.22)
2029	144.12 (61.18 to 227.06)	86.6 (32.44 to 140.77)	110.73 (45.43 to 176.03)	37.08 (32.12 to 42.04)	24.97 (21.42 to 28.53)	30.44 (26.48 to 34.4)
2030	149.52 (48.54 to 250.51)	90.11 (23.85 to 156.36)	114.92 (35.34 to 194.49)	36.5 (30.79 to 42.21)	24.7 (20.59 to 28.81)	30.02 (25.44 to 34.6)
2031	154.98 (34.03 to 275.93)	93.67 (13.97 to 173.38)	119.15 (23.76 to 214.53)	35.92 (29.44 to 42.4)	24.43 (19.74 to 29.11)	29.6 (24.39 to 34.81)
2032	160.49 (17.57 to 303.4)	97.3 (2.72 to 191.87)	123.42 (10.63 to 236.2)	35.34 (28.09 to 42.6)	24.16 (18.88 to 29.44)	29.19 (23.34 to 35.04)
2033	166.03 (-0.89 to 332.95)	101 (-9.95 to 211.95)	127.74 (-4.1 to 259.58)	34.78 (26.73 to 42.83)	23.9 (18.01 to 29.79)	28.79 (22.28 to 35.29)
2034	171.63 (-21.41 to 364.67)	104.82 (-24.1 to 233.73)	132.14 (-20.49 to 284.77)	34.22 (25.38 to 43.07)	23.65 (17.14 to 30.17)	28.39 (21.22 to 35.56)
2035	177.28 (-44.06 to 398.62)	108.73 (-39.82 to 257.29)	136.61 (-38.59 to 311.81)	33.66 (24.02 to 43.31)	23.4 (16.26 to 30.55)	28 (20.15 to 35.84)
2036	182.95 (-68.89 to 434.78)	112.7 (-57.17 to 282.56)	141.11 (-58.45 to 340.67)	33.11 (22.66 to 43.55)	23.16 (15.37 to 30.94)	27.61 (19.09 to 36.12)
2037	188.59 (-95.94 to 473.13)	116.7 (-76.19 to 309.58)	145.61 (-80.13 to 371.35)	32.56 (21.31 to 43.81)	22.91 (14.48 to 31.35)	27.22 (18.02 to 36.42)
2038	194.26 (-125.27 to 513.8)	120.78 (-96.97 to 338.53)	150.16 (-103.69 to 404.01)	32.02 (19.97 to 44.08)	22.68 (13.58 to 31.78)	26.85 (16.96 to 36.74)
2039	199.99 (-156.96 to 556.93)	124.99 (-119.63 to 369.62)	154.8 (-129.2 to 438.8)	31.49 (18.64 to 44.35)	22.46 (12.69 to 32.22)	26.48 (15.9 to 37.06)
2040	205.76 (-191.08 to 602.6)	129.32 (-144.26 to 402.89)	159.51 (-156.75 to 475.78)	30.97 (17.32 to 44.61)	22.23 (11.79 to 32.67)	26.12 (14.85 to 37.38)

Total skin cancer includes malignant skin melanoma, non-melanoma skin cancer (basal-cell carcinoma) and non-melanoma skin cancer (squamous-cell carcinoma). DALYs, disability-adjusted life-years; UI, uncertainty interval.

TABLE S32 Predicated age-standardized rates of incidence and DALYs of malignant skin melanoma by sex from 2022 to 2040, with 95% uncertainty intervals.

Year	Incidence			DALYs		
	Age-standardized rate (95% UI), male	Age-standardized rate (95% UI), female	Age-standardized rate (95% UI), both	Age-standardized rate (95% UI), male	Age-standardized rate (95% UI), female	Age-standardized rate (95% UI), both
2022	4.04 (3.92 to 4.17)	3.11 (3.02 to 3.2)	3.5 (3.4 to 3.59)	22.78 (22.35 to 23.21)	16.29 (15.99 to 16.58)	19.27 (18.93 to 19.6)
2023	3.99 (3.78 to 4.2)	3.07 (2.91 to 3.23)	3.45 (3.28 to 3.61)	22.35 (21.64 to 23.06)	16.09 (15.59 to 16.59)	18.95 (18.39 to 19.51)
2024	3.93 (3.62 to 4.24)	3.03 (2.79 to 3.27)	3.4 (3.14 to 3.66)	21.93 (20.88 to 22.97)	15.9 (15.16 to 16.65)	18.64 (17.81 to 19.48)
2025	3.87 (3.44 to 4.3)	2.99 (2.66 to 3.33)	3.35 (2.99 to 3.71)	21.5 (20.07 to 22.92)	15.71 (14.69 to 16.74)	18.33 (17.2 to 19.47)
2026	3.81 (3.26 to 4.37)	2.95 (2.51 to 3.39)	3.3 (2.83 to 3.77)	21.07 (19.24 to 22.9)	15.52 (14.18 to 16.85)	18.02 (16.55 to 19.48)
2027	3.75 (3.06 to 4.44)	2.91 (2.36 to 3.47)	3.25 (2.67 to 3.84)	20.65 (18.39 to 22.91)	15.32 (13.66 to 16.98)	17.71 (15.89 to 19.53)
2028	3.69 (2.86 to 4.53)	2.87 (2.2 to 3.54)	3.2 (2.49 to 3.92)	20.25 (17.54 to 22.96)	15.15 (13.14 to 17.16)	17.42 (15.23 to 19.61)
2029	3.63 (2.65 to 4.62)	2.84 (2.04 to 3.63)	3.16 (2.32 to 4)	19.86 (16.68 to 23.03)	14.98 (12.61 to 17.36)	17.14 (14.57 to 19.71)
2030	3.57 (2.44 to 4.71)	2.8 (1.88 to 3.72)	3.11 (2.14 to 4.08)	19.46 (15.82 to 23.1)	14.82 (12.07 to 17.57)	16.86 (13.9 to 19.82)
2031	3.51 (2.22 to 4.8)	2.76 (1.71 to 3.81)	3.06 (1.95 to 4.17)	19.07 (14.95 to 23.19)	14.64 (11.51 to 17.78)	16.58 (13.22 to 19.94)
2032	3.45 (2.01 to 4.9)	2.72 (1.54 to 3.9)	3.01 (1.77 to 4.26)	18.69 (14.09 to 23.29)	14.48 (10.94 to 18.01)	16.3 (12.53 to 20.07)
2033	3.39 (1.79 to 4.99)	2.69 (1.37 to 4)	2.96 (1.58 to 4.35)	18.32 (13.23 to 23.41)	14.33 (10.38 to 18.27)	16.04 (11.86 to 20.23)
2034	3.33 (1.57 to 5.09)	2.65 (1.2 to 4.1)	2.92 (1.39 to 4.44)	17.96 (12.39 to 23.54)	14.18 (9.82 to 18.55)	15.79 (11.19 to 20.4)
2035	3.27 (1.35 to 5.19)	2.61 (1.02 to 4.21)	2.87 (1.2 to 4.54)	17.6 (11.54 to 23.67)	14.04 (9.24 to 18.84)	15.54 (10.51 to 20.57)

2036	3.21 (1.13 to 5.29)	2.58 (0.85 to 4.31)	2.82 (1.01 to 4.63)	17.25 (10.71 to 23.8)	13.89 (8.66 to 19.12)	15.29 (9.84 to 20.74)
2037	3.15 (0.91 to 5.38)	2.54 (0.67 to 4.41)	2.78 (0.83 to 4.72)	16.9 (9.88 to 23.93)	13.75 (8.08 to 19.42)	15.04 (9.17 to 20.92)
2038	3.09 (0.7 to 5.48)	2.51 (0.49 to 4.52)	2.73 (0.64 to 4.82)	16.57 (9.07 to 24.08)	13.62 (7.49 to 19.74)	14.81 (8.51 to 21.12)
2039	3.03 (0.48 to 5.58)	2.47 (0.32 to 4.63)	2.68 (0.45 to 4.92)	16.25 (8.27 to 24.23)	13.5 (6.91 to 20.09)	14.59 (7.85 to 21.33)
2040	2.97 (0.27 to 5.67)	2.44 (0.14 to 4.74)	2.64 (0.27 to 5.02)	15.93 (7.48 to 24.38)	13.37 (6.32 to 20.43)	14.37 (7.2 to 21.54)

DALYs, disability-adjusted life-years; UI, uncertainty interval.

TABLE S33 Predicated age-standardized rates of incidence and DALYs of non-melanoma skin cancer (basal-cell carcinoma) by sex from 2022 to 2040, with 95% uncertainty intervals.

Year	Incidence			DALYs		
	Age-standardized rate (95% UI), male	Age-standardized rate (95% UI), female	Age-standardized rate (95% UI), both	Age-standardized rate (95% UI), male	Age-standardized rate (95% UI), female	Age-standardized rate (95% UI), both
2022	70.04 (65.19 to 74.89)	45.23 (42.29 to 48.18)	55.96 (52.2 to 59.72)	0.03 (0.03 to 0.03)	0.02 (0.02 to 0.02)	0.02 (0.02 to 0.03)
2023	73.29 (64.7 to 81.87)	47.76 (42.05 to 53.48)	58.8 (51.86 to 65.74)	0.03 (0.02 to 0.03)	0.02 (0.02 to 0.02)	0.02 (0.02 to 0.03)
2024	76.65 (63 to 90.3)	50.42 (40.95 to 59.89)	61.75 (50.49 to 73.01)	0.03 (0.02 to 0.03)	0.02 (0.02 to 0.02)	0.02 (0.02 to 0.03)
2025	80.12 (60.19 to 100.04)	53.21 (39.04 to 67.38)	64.83 (48.2 to 81.46)	0.03 (0.02 to 0.04)	0.02 (0.02 to 0.03)	0.03 (0.02 to 0.03)
2026	83.71 (56.3 to 111.12)	56.13 (36.27 to 76)	68.02 (44.94 to 91.1)	0.03 (0.02 to 0.04)	0.02 (0.01 to 0.03)	0.03 (0.02 to 0.03)
2027	87.41 (51.27 to 123.56)	59.2 (32.59 to 85.8)	71.34 (40.69 to 102)	0.03 (0.02 to 0.04)	0.02 (0.01 to 0.03)	0.03 (0.02 to 0.04)
2028	91.22 (45.05 to 137.39)	62.4 (27.92 to 96.88)	74.78 (35.37 to 114.19)	0.03 (0.02 to 0.05)	0.02 (0.01 to 0.03)	0.03 (0.01 to 0.04)
2029	95.13 (37.59 to 152.67)	65.76 (22.2 to 109.32)	78.34 (28.91 to 127.76)	0.03 (0.01 to 0.05)	0.02 (0.01 to 0.03)	0.03 (0.01 to 0.04)
2030	99.12 (28.81 to 169.44)	69.27 (15.32 to 123.22)	82.02 (21.26 to 142.78)	0.03 (0.01 to 0.05)	0.02 (0.01 to 0.04)	0.03 (0.01 to 0.05)
2031	103.21 (18.64 to 187.77)	72.93 (7.19 to 138.66)	85.81 (12.32 to 159.3)	0.03 (0.01 to 0.06)	0.02 (0.01 to 0.04)	0.03 (0.01 to 0.05)
2032	107.37 (7.03 to 207.72)	76.73 (-2.28 to 155.75)	89.71 (2 to 177.42)	0.03 (0 to 0.06)	0.02 (0.01 to 0.04)	0.03 (0 to 0.06)

2033	111.62 (-6.1 to 229.34)	80.7 (-13.21 to 174.62)	93.74 (-9.76 to 197.23)	0.03 (0 to 0.07)	0.02 (0 to 0.04)	0.03 (0 to 0.06)
2034	115.95 (-20.82 to 252.73)	84.86 (-25.71 to 195.43)	97.89 (-23.06 to 218.84)	0.03 (0 to 0.07)	0.02 (0 to 0.05)	0.03 (0 to 0.07)
2035	120.37 (-37.2 to 277.95)	89.2 (-39.92 to 218.32)	102.19 (-38 to 242.37)	0.03 (-0.01 to 0.08)	0.02 (0 to 0.05)	0.03 (-0.01 to 0.07)
2036	124.87 (-55.31 to 305.06)	93.72 (-55.94 to 243.39)	106.6 (-54.66 to 267.87)	0.03 (-0.01 to 0.08)	0.02 (0 to 0.05)	0.03 (-0.01 to 0.08)
2037	129.43 (-75.22 to 334.09)	98.41 (-73.92 to 270.75)	111.13 (-73.15 to 295.41)	0.03 (-0.02 to 0.09)	0.02 (-0.01 to 0.05)	0.03 (-0.01 to 0.08)
2038	134.07 (-97.01 to 365.15)	103.3 (-94.02 to 300.62)	115.79 (-93.58 to 325.17)	0.04 (-0.02 to 0.09)	0.02 (-0.01 to 0.06)	0.03 (-0.02 to 0.09)
2039	138.81 (-120.76 to 398.38)	108.43 (-116.41 to 333.26)	120.62 (-116.08 to 357.32)	0.04 (-0.03 to 0.1)	0.02 (-0.01 to 0.06)	0.03 (-0.02 to 0.09)
2040	143.65 (-146.56 to 433.85)	113.8 (-141.27 to 368.87)	125.61 (-140.77 to 391.99)	0.04 (-0.03 to 0.1)	0.03 (-0.01 to 0.06)	0.04 (-0.03 to 0.1)

DALYs, disability-adjusted life-years; UI, uncertainty interval.

TABLE S34 Predicated age-standardized rates of incidence and DALYs of non-melanoma skin cancer (squamous-cell carcinoma) by sex from 2022 to 2040, with 95% uncertainty intervals.

Year	Incidence			DALYs		
	Age-standardized rate (95% UI), male	Age-standardized rate (95% UI), female	Age-standardized rate (95% UI), both	Age-standardized rate (95% UI), male	Age-standardized rate (95% UI), female	Age-standardized rate (95% UI), both
2022	36.13 (32.54 to 39.72)	17.22 (15.2 to 19.24)	25.46 (22.67 to 28.25)	18.4 (17.98 to 18.82)	10.73 (10.48 to 10.97)	14.18 (13.9 to 14.46)
2023	37.84 (31.54 to 44.14)	17.73 (14.24 to 21.23)	26.48 (21.65 to 31.3)	18.28 (17.62 to 18.95)	10.65 (10.28 to 11.02)	14.09 (13.64 to 14.53)
2024	39.6 (29.59 to 49.61)	18.25 (12.8 to 23.71)	27.51 (19.94 to 35.08)	18.16 (17.18 to 19.13)	10.57 (10.05 to 11.09)	13.99 (13.34 to 14.64)
2025	41.42 (26.81 to 56.04)	18.78 (10.94 to 26.62)	28.57 (17.62 to 39.52)	18.03 (16.7 to 19.36)	10.49 (9.79 to 11.19)	13.89 (13 to 14.78)
2026	43.3 (23.18 to 63.42)	19.3 (8.68 to 29.92)	29.64 (14.69 to 44.59)	17.9 (16.18 to 19.62)	10.41 (9.51 to 11.3)	13.79 (12.63 to 14.95)
2027	45.24 (18.68 to 71.8)	19.83 (6.05 to 33.6)	30.73 (11.16 to 50.3)	17.77 (15.63 to 19.91)	10.32 (9.21 to 11.44)	13.69 (12.25 to 15.13)
2028	47.23 (13.28 to 81.18)	20.35 (3.03 to 37.66)	31.83 (7.02 to 56.64)	17.64 (15.05 to 20.22)	10.24 (8.91 to 11.58)	13.59 (11.84 to 15.33)
2029	49.25 (6.92 to 91.58)	20.86 (-0.37 to 42.09)	32.94 (2.26 to 63.62)	17.5 (14.44 to 20.56)	10.16 (8.59 to 11.73)	13.48 (11.42 to 15.54)

2030	51.3 (-0.43 to 103.02)	21.36 (-4.14 to 46.87)	34.05 (-3.15 to 71.24)	17.36 (13.81 to 20.91)	10.07 (8.25 to 11.89)	13.37 (10.98 to 15.77)
2031	53.36 (-8.81 to 115.54)	21.85 (-8.29 to 51.99)	35.14 (-9.21 to 79.49)	17.23 (13.17 to 21.28)	9.98 (7.91 to 12.05)	13.27 (10.53 to 16)
2032	55.45 (-18.25 to 129.15)	22.32 (-12.81 to 57.45)	36.23 (-15.92 to 88.38)	17.09 (12.51 to 21.67)	9.9 (7.57 to 12.23)	13.16 (10.07 to 16.25)
2033	57.55 (-28.79 to 143.89)	22.77 (-17.68 to 63.23)	37.3 (-23.3 to 97.9)	16.94 (11.83 to 22.06)	9.81 (7.21 to 12.41)	13.05 (9.59 to 16.5)
2034	59.65 (-40.47 to 159.78)	23.21 (-22.92 to 69.33)	38.36 (-31.35 to 108.07)	16.8 (11.13 to 22.47)	9.72 (6.85 to 12.59)	12.93 (9.11 to 16.76)
2035	61.77 (-53.3 to 176.83)	23.63 (-28.5 to 75.76)	39.41 (-40.08 to 118.89)	16.66 (10.43 to 22.89)	9.63 (6.48 to 12.78)	12.82 (8.62 to 17.02)
2036	63.87 (-67.3 to 195.03)	24.02 (-34.4 to 82.44)	40.43 (-49.46 to 130.31)	16.51 (9.71 to 23.31)	9.54 (6.1 to 12.97)	12.71 (8.12 to 17.3)
2037	65.95 (-82.49 to 214.39)	24.39 (-40.62 to 89.39)	41.41 (-59.5 to 142.32)	16.37 (8.99 to 23.75)	9.45 (5.73 to 13.16)	12.59 (7.61 to 17.57)
2038	68.02 (-98.88 to 234.92)	24.73 (-47.13 to 96.59)	42.37 (-70.18 to 154.93)	16.22 (8.26 to 24.18)	9.35 (5.34 to 13.36)	12.48 (7.1 to 17.85)
2039	70.08 (-116.51 to 256.68)	25.06 (-53.95 to 104.06)	43.32 (-81.53 to 168.17)	16.07 (7.52 to 24.63)	9.26 (4.96 to 13.55)	12.36 (6.59 to 18.13)
2040	72.15 (-135.41 to 279.7)	25.37 (-61.06 to 111.79)	44.25 (-93.53 to 182.03)	15.92 (6.77 to 25.08)	9.16 (4.57 to 13.74)	12.24 (6.07 to 18.41)

DALYs, disability-adjusted life-years; UI, uncertainty interval.
